# Supplementary material for: Diastereodivergent chiral aldehyde catalysis for asymmetric 1,6-conjugated addition and Mannich reactions
Source: Nat Commun. 2020 Oct 23;11:5372. doi: 10.1038/s41467-020-19245-3 (PMC7584650; doi:10.1038/s41467-020-19245-3)
Supplement: Supplementary file 1 — Supplementary Information [file 41467_2020_19245_MOESM1_ESM.pdf]

Supplementary Information

for

Diastereodivergent Chiral Aldehyde Catalytsis for Asymmetric 1,6-Conjugated Addition and Mannich Reactions

Wei Wen[*a], Ming-Jing Luo[a], Yi Yuan[b], Jian-Hua Liu[a], Zhu-Lian Wu[a], Tian Cai[a], Zhao-Wei Wu[a], Qin Ouyang[*b], Qi-Xiang Guo[*a]

[a] Key Laboratory of Applied Chemistry of Chongqing Municipality, and Chongqing Key Laboratory of Soft-Matter Material Chemistry and Function Manufacturing, School of Chemistry and Chemical Engineering, Southwest University, Chongqing, 400715, China. wenwei1989@swu.edu.cn; qxguo@swu.edu.cn

[b] College of Pharmacy, Third Military Medical University, Chongqing 400038, China. ouyangq@tmmu.edu.cn

Table of Contents:

1. General data...S2

2. Procedure for the preparation of catalyst 3d...S2

3. Reaction condition optimization of the 1,6-conjugated addition....................S3

4. Reaction condition optimization of the Mannich reaction..............................S5

5. General procedure for the catalytic asymmetric 1,6-conjugated addition...S10

6. General procedure for the catalytic asymmetric Mannich reaction............S36

7. Determination of the absolute configurations of *anti*-5g, *syn*-5g, *anti*-8a and *syn*-8a..S65

8. The spectra of ^{1}H NMR and ^{13}C NMR..S71

9. DFT computational calculation studies...S159

10. Supplementary References...S170

1. General data.

Solvents for reactions were dried appropriately before use: toluene, THF and Et_2O were dried by refluxing with sodium and benzophenone as indicator, CH_2Cl_2 and $CHCl_3$ were dried by refluxing with CaH_2. All other reagents were directly used as purchased from either Adamas-beta® or Energy Chemical.

Unless otherwise noted, commercial reagents were used as received and all reactions were carried out directly in air atmosphere. All reactions were monitored by TLC with silica gel coated plates. ^{1}H NMR (600 or 400 MHz) and ^{13}C NMR (150 or 100 MHz) spectra were recorded on Bruker Avance 600 MHz spectrometer or Bruker Avance 400 MHz spectrometer. Chemical shifts (δ) are reported in ppm from tetramethyl silane (TMS) with the solvent resonance as the internal standard. Proton signal multiplicities are given as s (singlet), d (doublet), t (triplet), q (quartet), m (multiplet), br (broad) or a combination of them. *J*-values are in Hz. HRMS (ESI-Q-TOF) spectra were recorded on Bruker Impact-II. Enantiomer ratios were determined by HPLC (Chiralpak IA-H, IC-H, AD-H, OD-H, OJ-H columns were purchased from Daicel Chemical Industries, LTD.). Optical rotations were determined at λ = 589 nm (sodium D line) by using a Rudolph-API automatic polarimeter. The catalysts **3**[1-2] and **4**[3], *para*-quinone methides **1**[4-5] and imines **7**[6] were prepared according to the literatures.

2. Procedure for the preparation of catalyst 3d.

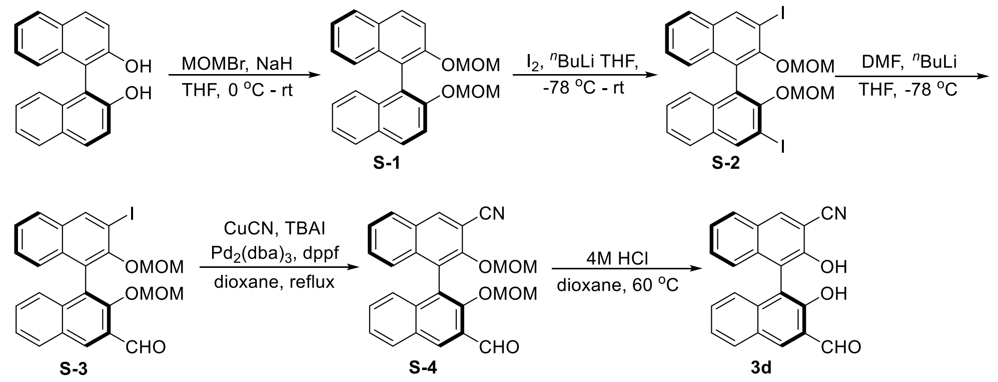

(S)-3'-formyl-2,2'-bis(methoxymethoxy)-[1,1'-binaphthalene]-3-carbonitrile (S-4):

A mixture of **S-3**[2,7] (0.73 g, 1.39 mmol), CuCN (0.933 g, 10.425 mmol), $Pd_2(dba)_3$ (0.063 g, 0.069 mmol), dppf (0.077 g, 0.139 mmol) and tetrabutylammonium iodide (0.514 g, 1.39 mmol) in dioxane (15 mL) was effectively stirred at 80 °C and monitored by TLC. After the compound **S-3** was completely consumed, saturated $NaHCO_3$ (5 mL) was added and the resulted solution was filtered with silica. The filtrate was washed with water (3 × 15 mL), and the organic layer was dried over anhydrous Na_2SO_4, filtered and concentrated *in vacuo* to give crude product **S-4**. This crude product was used for the next step without further purification.

(S)-3'-formyl-2,2'-dihydroxy-[1,1'-binaphthalene]-3-carbonitrile (3d):

A HCl solution (4M, 1.39 mL) was added to the mixture of **S-4** (0.73 g, 1.39 mmol) in dioxane (20 mL). The reaction was stirred at 60 °C for 1 hour and then cooled to room temperature. After the reaction solution was extracted with CH_2Cl_2 (3×10 mL), the organic layer was combined and washed with water (3×15 mL), then dried over anhydrous Na_2SO_4 and concentrated in vacuo., The residue was purified by column chromatography on silica gel (petroleum ether: EtOAc = 10:1) to afford catalyst **3d** as a yellow soild (0.44 g, 1.3 mmol, 94%).

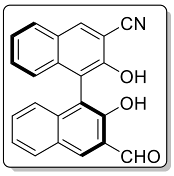

3d: yellow soild; m.p. = 320-321 °C; R_f = 0.38 (petroleum ether/EtOAc = 3:1); **^{1}H NMR (600 MHz, DMSO-*d*$_6$)** δ 10.38 (s, 1H), 10.24 (s, 1H), 9.75 (s, 1H), 8.70 (s, 1H), 8.63 (s, 1H), 8.18 (dd, *J* = 6.4, 2.7 Hz, 1H), 8.04 (d, *J* = 7.7 Hz, 1H), 7.53 – 7.35 (m, 4H), 7.02 – 6.89 (m, 2H); **^{13}C NMR (151 MHz, DMSO-*d*$_6$)** δ 196.81, 154.67, 152.82, 137.66, 137.47, 136.72, 136.13, 130.87, 130.03, 129.39, 128.02, 127.79, 124.77, 124.70, 124.68, 124.41, 123.84, 117.64, 116.20, 115.18, 103.52; **HRMS(ESI):** calcd. for $C_{22}H_{13}N_2O_2$ $(M+H)^+$: 340.0968, found: 340.0968.

3. Reaction condition optimization of 1,6-conjugated addition.

1) Reaction condition optimization of the *anti*-selective 1,6-conjugated addition.

Supplementary Table 1. The screening of base equivalent and catalyst loading

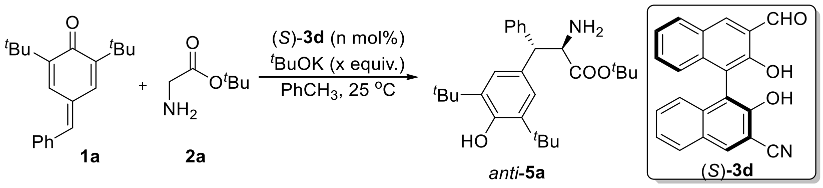

entry	n	x (equiv.)	t (h)	yield (%)[b]	ee (%)[c]	dr (*syn:anti*)[c]
1	20	1	5	64	96	10:90
2	20	0.3	10	72	98	10:90
3	10	0.3	18	69	98	9:91
4[d]	10	0.3	67	74	97	10:90

[a] **1a** (0.10 mmol), **2a** (0.20 mmol), catalyst **3d** (n mol%), ^{t}BuOK (x equiv.) in toluene (0.5 mL) at 25 °C. [b] Isolated yield. [c] Determined by chiral HPLC analysis. [d] Using 1 mL $PhCH_3$.

2) Reaction condition optimization of the *syn*-selective 1,6-conjugated addition.

Supplementary Table 2. Solvent screening

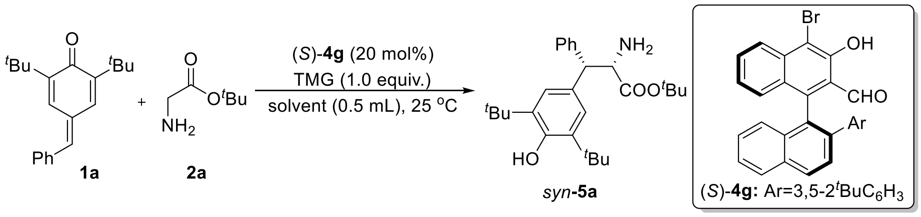

entry	solvent	t (h)	yield (%)[b]	ee (%)[c]	dr (*syn*:*anti*)[c]
1	toluene	3	36	79	76:24
2	xylenes	5	27	79	79:21
3	mesitylene	5	37	83	79:21
4	$PhCF_3$	5	33	51	62:38
5	Ph^{i}Pr	2	14	73	85:15
6	CH_2Cl_2	4	15	61	65:35
7	THF	4	13	13/34	54:46
8	Et_2O	4	24	64	70:30

[a] **1a** (0.10 mmol), **2a** (0.20 mmol), catalyst **4g** (0.02 mmol), TMG (0.1 mmol) in solvent (0.5 mL) at 25 °C. [b] Isolated yield. [c] Determined by chiral HPLC analysis.

Supplementary Table 3. Base screening

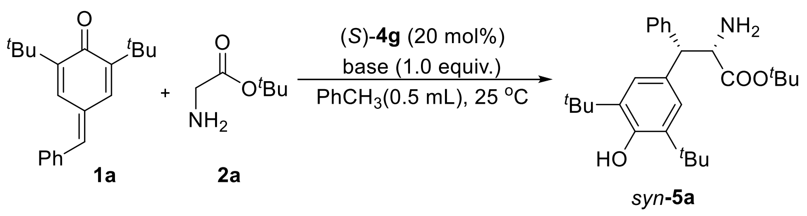

entry	base	t (h)	yield (%)[b]	ee (%)[c]	dr (*syn*:*anti*)[c]
1	DBU	4	32	43	59:41
2	DBN	2	24	30	50:50
3	TMG	3	36	79	76:24
4	BTMG	4	35	56	80:20
5	MTBD	6	23	44	60:40
6[d]	^{t}BuOK	3	69	86	94:6
7[d]	MeONa	22	41	84	86:14
8[d]	^{t}BuONa	22	28	64	79:21
9[d]	KOH	5	64	84	94:6

[a] **1a** (0.10 mmol), **2a** (0.20 mmol), catalyst **4g** (0.02 mmol), base (0.10 mmol) in $PhCH_3$ (0.5 mL) at 25 °C. [b] Isolated yield. [c] Determined by chiral HPLC analysis. [d] Using 0.5 mL mesitylene as solvent.

Supplementary Table 4. Base equivalent screening

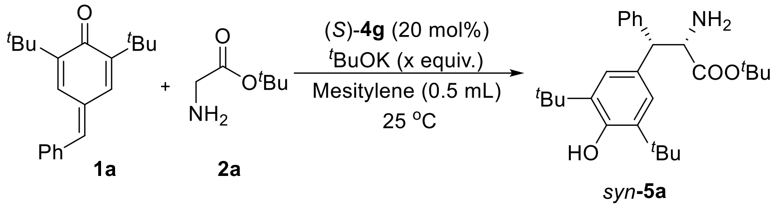

entry	x (equiv.)	t (h)	yield (%)[b]	ee (%)[c]	dr (*syn*:*anti*)[c]
1	1	3	69	86	94:6
2	0.7	5	70	87	95:5
3	0.5	6	66	82	93:7
4	0.3	7	70	90	95:5
5	0.2	11	65	90	96:4
6[d]	0.3	11	61	88	95:5
7[e]	0.3	7	56	84	94:6

[a] **1a** (0.10 mmol), **2a** (0.20 mmol), catalyst **4g** (0.02 mmol), ^{t}BuOK (x equiv.) in mesitylene (0.5 mL) at 25 °C. [b] Isolated yield. [c] Determined by chiral HPLC analysis. [d] Using 15 mol % **4g**. [e] Using 10 mol % **4g**.

4. Reaction condition optimization of the Mannich reaction.

1) Reaction condition optimization of the *syn*-selective Mannich reaction

Supplementary Table 5. Solvent screening

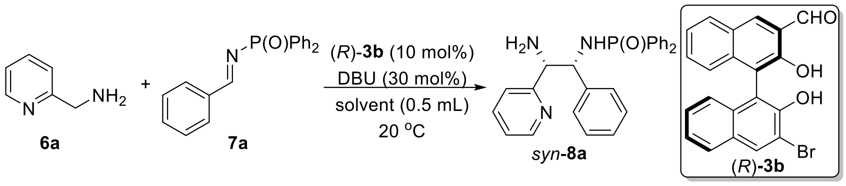

entry[a]	solvent	t (h)	yield (%)[b]	ee (%)[c]	dr (*syn*:*anti*)[c]
1	$PhCH_3$	12	95	66	93:7
2	PhCl	12	94	68	94:6
3	EtOAc	12	63	65	99:1
4	THF	12	92	55	98:2
5	Et_2O	48	68	55	98:2
6	CH_2Cl_2	12	87	76	95:5
7	$CHCl_3$	48	64	19	92:8
8	CCl_4	49	57	60	81:19
9	CH_2ClCH_2Cl	12	77	8	48:52

[a] **6a** (0.1 mmol), **7a** (0.13 mmol), **3b** (0.01 mmol), DBU (0.03 mmol) and solvent (0.5 mL), at 20 °C; [b] Isolated yield. [c] Determined by chiral HPLC.

Supplementary Table 6. Base screening

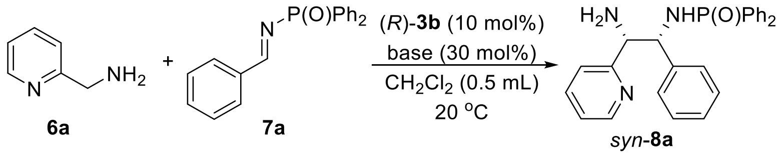

entry[a]	base	t (h)	yield (%)[b]	ee (%)[c]	dr (*syn:anti*)[c]
1	DBU	12	87	76	95:5
2	DBN[d]	12.5	87	67	92:8
3	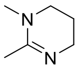	12.5	92	73	98:2
4	Et_3N	48	trace	n.d.[g]	n.d.
5	TBD[e]	12	24	23	n.d.
6	TMG[f]	12	28	-19/-29	77:23
7	K_2CO_3	42	trace	n.d.	n.d.

[a] **6a** (0.1 mmol), **7a** (0.13 mmol), **3b** (0.01 mmol), base (0.03 mmol) and CH_2Cl_2 (0.5 mL), at 20 °C; [b] Isolated yield. [c] Determined by chiral HPLC. [d] 1,5-Diazabicyclo[4.3.0]non-5-ene. [e] 1,5,7-Triazabicylo[4.4.0]dec-5-ene. [f] 1,1,3,3-Tetramethylguanidine. [g] n.d. = Not determined.

Supplementary Table 7. Base equivalent screening

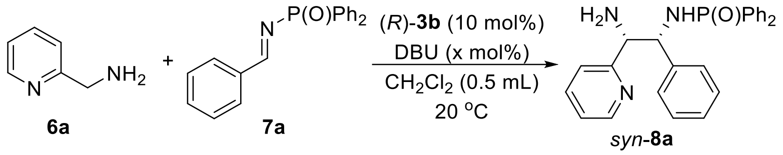

entry[a]	x (mol%)	t (h)	yield (%)[b]	ee (%)[c]	dr (*syn:anti*)[c]
1	10	48	79	18	90:10
2	30	12	87	76	95:5
3	50	12	87	79	96:4
4	70	12	85	81	94:6
5	100	12	82	78	93:7

[a] **6a** (0.1 mmol), **7a** (0.13 mmol), **3b** (0.01 mmol), DBU (x mol%) and CH_2Cl_2 (0.5 mL), at 20 °C; [b] Isolated yield. [c] Determined by chiral HPLC.

Supplementary Table 8. Additive screening

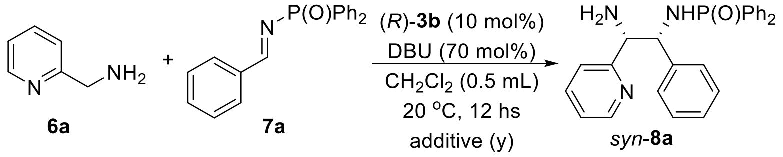

entry[a]	additive (y)	yield (%)[b]	ee (%)[c]	dr (*syn:anti*)[c]
1	-	85	81	94:6
2	PhCOOH (5 mol%)	71	81	95:5
3	Picolinic acid (5 mol%)	85	81	95:5

4	H_2O (3 equiv.)	56	79	96:4
5	anhydrous Na_2SO_4 (50 mg)	88	78	94:6

[a] **6a** (0.1 mmol), **7a** (0.13 mmol), **3b** (0.01 mmol), DBU (0.07 mmol), CH_2Cl_2 (0.5 mL) and specified additive, at 20 °C; [b] Isolated yield. [c] Determined by chiral HPLC.

Supplementary Table 9. Optimization of reaction temperature and concentration

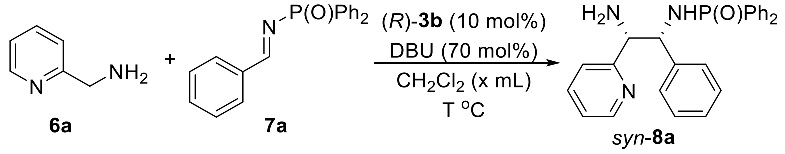

entry[a]	T (°C)	x (mL)	t (h)	yield (%)[b]	ee (%)[c]	dr (*syn:anti*)[c]
1	10	0.25	28.5	88	83	96:4
2	10	0.5	24	83	87	96:4
3	10	1	38	89	87	97:3
4	0	0.25	60	83	86	97:3
5	0	0.5	66	83	90	97:3
6	0	1	72	72	90	98:2
7	-10	0.5	72	40	90.5	98:2

[a] **6a** (0.1 mmol), **7a** (0.13 mmol), **3b** (0.01 mmol), DBU (0.07 mmol) and CH_2Cl_2 (x mL), at T °C; [b] Isolated yield. [c] Determined by chiral HPLC.

Supplementary Table 10. *N*-Protection group screening

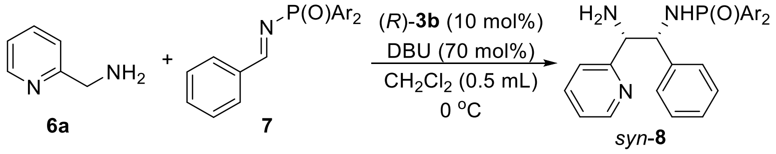

entry[a]	Ar	t (h)	yield (%)[b]	ee (%)[c]	dr (*syn:anti*)[d]
1	Ph	66	83	90	97:3
2	2-MeC_6H_4	71	83	85	97:3
3	3,5-$Me_2C_6H_3$	77	84	87	98:2
4	2,6-$Me_2C_6H_3$	75	74	76	95:5

[a] **6a** (0.1 mmol), **7** (0.13 mmol), **3b** (0.01 mmol), DBU (0.07 mmol) and CH_2Cl_2 (0.5 mL), at 0 °C; [b] Isolated yield. [c] Determined by chiral HPLC. [d] Determined by ^1H NMR.

2) Reaction condition optimization of the *anti*-selective Mannich reaction.

Supplementary Table 11. Solvent screening

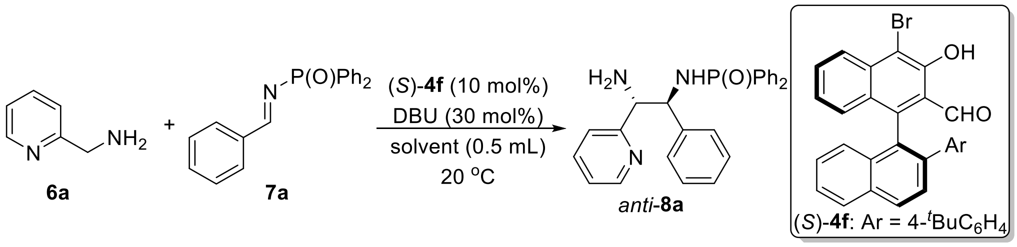

entry[a]	solvent	t (h)	yield (%)[b]	ee (%)[c]	dr (*syn*:*anti*)[c]
1	CH_2Cl_2	12	25	61	51:49
2	$CHCl_3$	24	29	60	22:78
3	CH_2ClCH_2Cl	12	55	43	48:52
4	cyclohexane	12.5	38	69	49:51
5	EtOAc	13	57	78	40:60
6	^{i}PrOH	13	47	79	32:68
7	THF	12.5	75	79	31:69
8	Et_2O	12	81	83	38:62
9	CPME	12	78	87	27:73
10	MeO^{t}Bu	12	84	88	30:70
11	$PhCH_3$	12	64	88	25:75
12	PhCl	12	51	62	22:76
13	PhOMe	12	62	82	32:68
14	PhCN	12	59	70	66:34
15	benzene	12	47	85	23:77
16	PhEt	12	57	88	34:66
17	Ph^{n}Bu	12	71	89	20:80
18	*o*-xylene	12	72	89	21:79
19	*m*-xylene	12	83	88	23:77
20	*p*-xylene	12	80	90	24:76
21	mesitylene	12	80	88	21:79

[a] **6a** (0.1 mmol), **7a** (0.13 mmol), **4f** (0.01 mmol), DBU (0.03 mmol) and solvent (0.5 mL), at 20 °C; [b] Isolated yield. [c] Determined by chiral HPLC.

Supplementary Table 12. Base screening

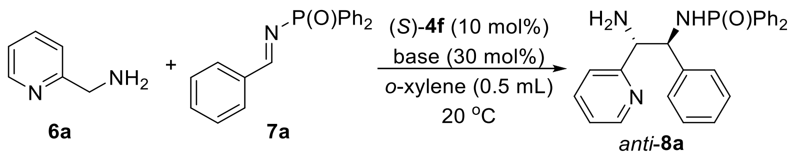

entry[a]	base	t (h)	yield (%)[b]	ee (%)[c]	dr (*syn*:*anti*)[c]
1	DBU	12	72	89	21:79
2	DBN[d]	12	79	88	20:80
3	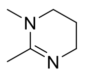	12	74	89	22:78
4	TBD[e]	22	35	63	45:55

[a] **6a** (0.1 mmol), **7a** (0.13 mmol), **4f** (0.01 mmol), base (0.03 mmol) and *o*-xylene (0.5 mL), at 20 °C; [b] Isolated yield. [c] Determined by chiral HPLC. [d] 1,5-Diazabicyclo[4.3.0]non-5-ene. [e] 1,5,7-Triazabicylo[4.4.0]dec-5-ene.

Supplementary Table 13. Base equivalent screening

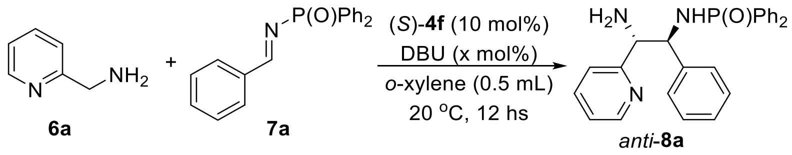

entry[a]	x (mol%)	yield (%)[b]	ee (%)[c]	dr (*syn:anti*)[c]
1	10	74	88	20:80
2	30	72	89	21:79
3	50	73	89	21:79
5	70	73	90	22:78
6	100	74	89	21:79

[a] **6a** (0.1 mmol), **7a** (0.13 mmol), **4f** (0.01 mmol), DBU (x mol%) and *o*-xylene (0.5 mL), at 20 °C; [b] Isolated yield. [c] Determined by chiral HPLC.

Supplementary Table 14. Optimization of the reaction temperature and concentration

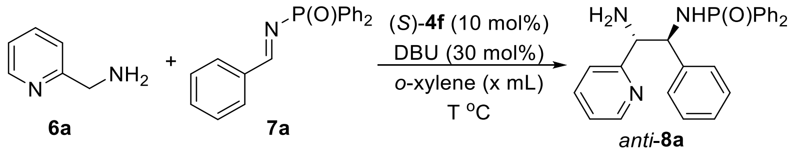

entry[a]	T (°C)	x (mL)	t (h)	yield (%)[b]	ee (%)[c]	dr (*syn:anti*)[c]
1	20	0.5	12	72	89	21:79
2	20	1	13	74	87	19:81
3	10	0.5	17	80	89	24:76
4	10	1	21	66	87	21:79
5	0	0.5	22	81	91	23:77
6	0	1	44	74	91	20:80
7	-10	0.5	44	72	93	17:83
8	-10	1	44	71	93	16:84
9	-20	0.5	77	30	95	16:84
10	-20	1	77	33	94	17:83

[a] **6a** (0.1 mmol), **7a** (0.13 mmol), **4f** (0.01 mmol), DBU (0.03 mmol) and *o*-xylene (x mL), at T °C; [b] Isolated yield. [c] Determined by chiral HPLC.

Supplementary Table 15. Additive screening

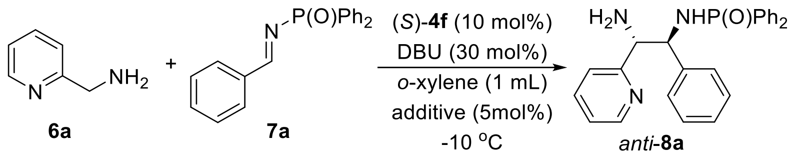

entry[a]	additive	t (h)	yield (%)[b]	ee (%)[c]	dr (*syn:anti*)[c]
1	-	44	71	93	16:84
2	2-Carboxypyridine	51	75	92	16:84
3	Salicylic acid	45	75	92	16:84
4	PhCOOH	45	56	93	15:85

[a] Reaction conditions: **6a** (0.1 mmol), **7a** (0.13 mmol), **4f** (0.01 mmol), DBU (0.03 mmol), *o*-xylene (1 mL) and additive (5 mol%), at -10 °C; [b] Isolated yield. [c] Determined by chiral HPLC.

Supplementary Table 16. *N*-Protection group screening

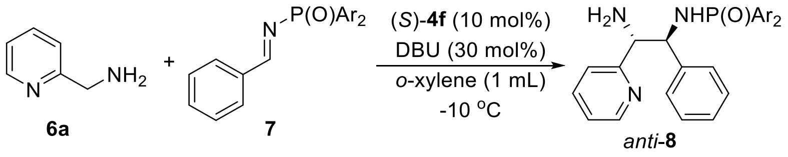

entry[a]	Ar	t (hs)	yield (%)[b]	ee (%)[c]	dr (*syn:anti*)[d]
1	Ph	44	71	93	16:84
2	2-MeC_6H_4	46	69	88	4:96
3	3,5-$Me_2C_6H_3$	46	65	90	12:88
4	2,6-$Me_2C_6H_3$	76	trace	n.d.[e]	n.d.

[a] **6a** (0.1 mmol), **7a** (0.13 mmol), **4f** (0.01 mmol), DBU (0.03 mmol) and *o*-xylene (1 mL), at -10 °C; [b] Isolated yield. [c] Determined by chiral HPLC. [d] Determined by ^{1}HNMR. [e] n.d. = not determined.

5. General procedure for the catalytic asymmetric 1,6-conjugated addition reaction.

1) General procedure for the catalytic asymmetric *anti*-1,6-conjugated addition reaction

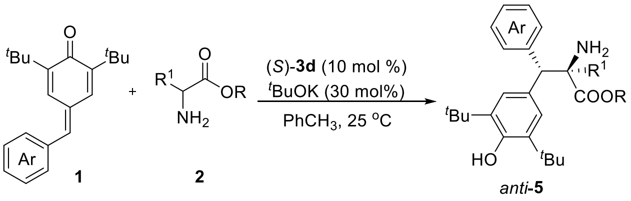

A dry Schlenk tube was charged with *para*-quinone methides **1** (0.1 mmol), catalyst **3d** (0.01 mmol), ^{t}BuOK (0.03 mmol) and α-amino acid derivatives **2** (0.2 mmol). After the addition of dry toluene (1.0 mL), the reaction mixture was effectively stirred at 25 ℃ and monitored by TLC. After the complete consumption of *para*-quinone methides **1**, the mixture was concentrated *in vacuo* and purified by flash chromatography on silica gel (petroleum ether: EtOAc = 15:1 to 2:1) to afford the compounds *anti*-**5**.

2) General procedure for catalytic asymmetric *syn*-1,6-conjugated addition reaction

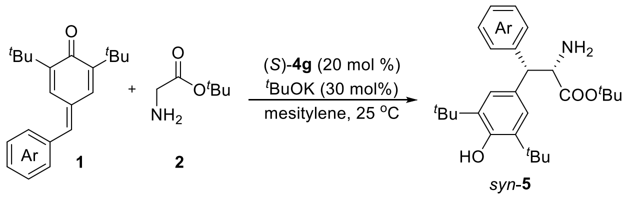

A dry Schlenk tube was charged with *para*-quinone methides **1** (0.1 mmol), catalyst **4g** (0.02 mmol), ^{t}BuOK (0.03 mmol) and glycine *tert*-butyl ester **2** (0.2 mmol). After addition of dry mesitylene (0.5 mL), the reaction mixture was effectively stirred at 25 ℃ and monitored by TLC. After the complete consumption of *para*-quinone methides **1**, the mixture was concentrated *in vacuo* and purified by flash chromatography on silica gel (petroleum ether: EtOAc = 15:1 to 2:1) to afford the compounds *syn*-**5.**

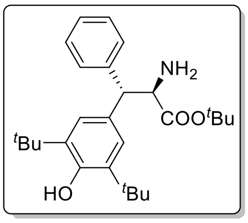

***tert*-butyl (2*R*,3*S*)-2-amino-3-(3,5-di-*tert*-butyl-4-hydroxyphenyl)-3-phenylpropanoa (*anti*-5a):** white soild (31.3 mg, 74%); m.p. = 145-146 ^{o}C; R_f = 0.30 (petroleum ether/EtOAc =4:1); the enantiomeric excess was determined to be 97% by HPLC analysis on Daicel Chirapak IA-H column (hexane/isopropanol = 98/2, flow rate 1.0 mL/min, T = 30°C), UV 220 nm, t_R(major) 11.78 min, t_R(minor) 13.33 min; $[a]_D^{25}$ = -47.4 (c = 0.43, $CHCl_3$); **^{1}H NMR (600 MHz, $CDCl_3$)** δ 7.37 (d, *J* = 7.3 Hz, 2H), 7.31 (t, *J* = 7.6 Hz, 2H), 7.20 (t, *J* = 7.3 Hz, 1H), 7.11 (s, 2H), 5.05 (s, 1H), 3.99 (dd, *J* = 78.2, 9.9 Hz, 2H), 1.40 (s, 18H), 1.15 (s, 9H); **^{13}C NMR (151 MHz, $CDCl_3$)** δ 173.87, 152.54, 142.00, 135.59, 132.02, 128.71, 128.41, 126.68, 124.97, 80.71, 59.90, 58.01, 34.29, 30.29, 27.64; **HRMS(ESI)**: calcd. for $C_{27}H_{42}NO_3(M+H)^+$: 426.3003, found: 426.3003.

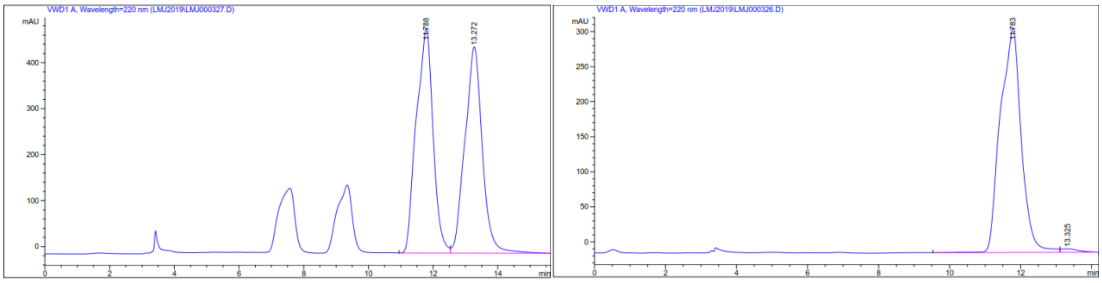

Peak #	RetTime [min]	Type	Width [min]	Area [mAU*s]	Height [mAU]	Area %
1	11.788	BV	0.5032	1.75507e4	487.38632	51.2041
2	13.272	VBA	0.5330	1.67252e4	448.27365	48.7959

Peak #	RetTime [min]	Type	Width [min]	Area [mAU*s]	Height [mAU]	Area %
1	11.783	BV	0.5554	1.30792e4	319.10431	98.7341
2	13.325	VBA	0.4894	167.69379	4.93932	1.2659

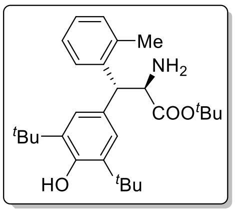

***tert*-butyl (2*R*,3*R*)-2-amino-3-(3,5-di-*tert*-butyl-4-hydroxyphenyl)-3-(o-tolyl)propanoate (*anti*-5b):** white solid (23 mg, 52%); m.p. = 129-130 ^{o}C; R_f = 0.33 (petroleum ether/EtOAc = 4:1); the enantiomeric excess was determined to be 98% by HPLC analysis on Daicel Chirapak AD-H column (hexane/isopropanol = 98/2, flow rate 1.0 mL/min, T = 30°C), UV 220 nm, t_R(major) 13.29 min, t_R(minor) 14.57 min; $[a]_D^{25}$ = -262.8 (c = 0.40, $CHCl_3$); **^{1}H NMR (400 MHz, $CDCl_3$)** δ 7.52 (d, *J* = 7.7 Hz, 1H), 7.26 – 7.21 (m, 1H), 7.15 – 7.03 (m, 4H), 5.02 (s, 1H), 4.20 – 4.10 (m, 2H), 2.37 (s, 3H), 1.38 (s, 18H), 1.12 (s, 9H); **^{13}C NMR (101 MHz, $CDCl_3$)** δ 174.13, 152.42, 140.16, 137.33, 135.39, 130.97, 130.75, 126.46, 126.35, 125.52, 125.48, 80.67, 76.70, 59.81, 53.17, 34.23, 30.26, 27.65, 20.23; **HRMS (ESI)**: calcd. for $C_{28}H_{41}NO_3(M+H)^+$:

440.3159, found: 440.3159.

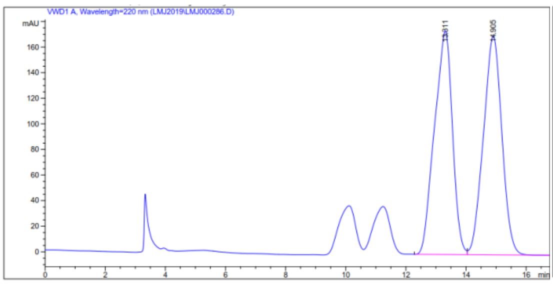

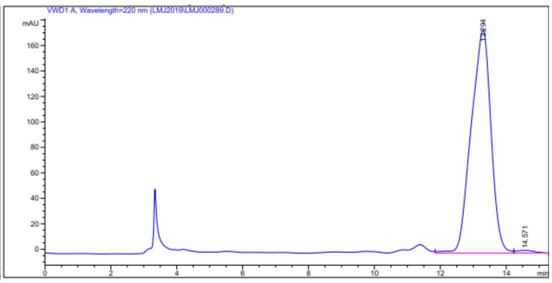

Peak #	RetTime [min]	Type	Width [min]	Area [mAU*s]	Height [mAU]	Area %
1	13.311	BV	0.5977	7249.24512	174.75742	49.8141
2	14.905	VBA	0.6438	7303.34961	171.07726	50.1859

Peak #	RetTime [min]	Type	Width [min]	Area [mAU*s]	Height [mAU]	Area %
1	13.294	VV	0.6040	7316.77100	175.14671	98.9419
2	14.571	VBA	0.5679	78.24556	2.06280	1.0581

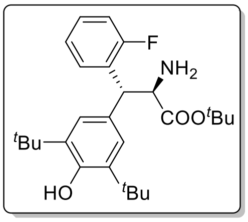

***tert*-butyl (2*R*,3*R*)-2-amino-3-(3,5-di-*tert*-butyl-4-hydroxyphenyl)-3-(2-fluorophenyl)propanoate (*syn*-5c):** white soild (37.2 mg, 84%); m.p. = 128-129 ^{o}C; R_f = 0.21(petroleum ether/EtOAc = 4:1); the enantiomeric excess was determined to be 99% by HPLC analysis on Daicel Chirapak IA-H column (hexane/isopropanol = 98/2, flow rate 1.0 mL/min, T = 30°C), UV 220 nm, t_R(major) 16.43 min, t_R(minor) 13.64 min; $[a]_D^{25}$ **=** -137.1 (c = 0.63, $CHCl_3$); **^{1}H NMR (600 MHz, $CDCl_3$)** δ 7.45 (td, *J* = 7.6, 1.4 Hz, 1H), 7.20 – 7.08 (m, 4H), 7.02 (dd, *J* = 13.9, 4.6 Hz, 1H), 5.06 (s, 1H), 4.21 (dd, *J* = 131.3, 10.1 Hz,2H), 1.40 (s,18H), 1.15 (s,9H); **^{13}C NMR (151 MHz, $CDCl_3$)** δ 173.88, 161.95, 160.33, 152.90, 152.66, 135.63, 133.57, 131.18, 130.86, 129.38, 129.25, 129.16, 129.09, 129.06, 128.35, 128.06, 128.01, 127.35, 125.33, 124.31, 124.29, 123.90, 117.93, 115.87, 115.71, 80.77, 59.07, 50.20, 34.29, 30.28, 27.61; **HRMS(ESI)**: calcd. for $C_{27}H_{39}FNO_3(M+H)^+$: 444.2908, found: 444.2916.

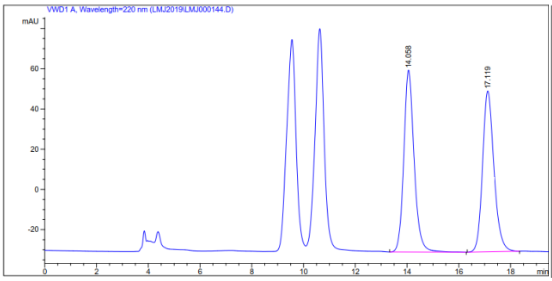

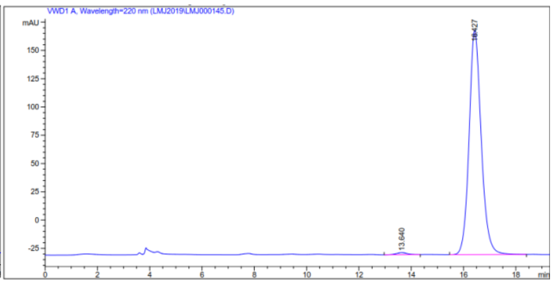

Peak #	RetTime [min]	Type	Width [min]	Area [mAU*s]	Height [mAU]	Area %
1	14.058	BB	0.4172	2509.66113	90.40210	50.5909
2	17.119	BB	0.4685	2451.03223	79.93825	49.4091

Peak #	RetTime [min]	Type	Width [min]	Area [mAU*s]	Height [mAU]	Area %
1	13.640	BB	0.4362	51.34750	1.76958	0.7996
2	16.427	BB	0.4886	6370.09668	198.25345	99.2004

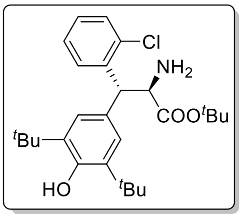

***tert*-butyl (2*R*,3*R*)-2-amino-3-(2-chlorophenyl)-3-(3,5-di-*tert*-butyl-4-hydroxyphenyl)propanoate (*anti*-5d):** light yellow oil (38.3mg, 52%); R_f = 0.23 (petroleum ether/EtOAc = 4:1); the enantiomeric excess was determined to be 98% by HPLC analysis on Daicel Chirapak IA-H column (hexane/isopropanol = 98/2, flow rate 1.0 mL/min, T = 30°C), UV 220 nm, t_R(major) 13.30 min, t_R(minor) 11.63 min; $[a]_D^{25}$ = -96.3 (c = 0.57, $CHCl_3$); **^{1}H NMR**

(400 MHz, $CDCl_3$) δ 7.61 (d, *J* = 7.7 Hz, 1H), 7.31 (dd, *J* = 23.3, 7.7 Hz, 2H), 7.16 – 7.08 (m, 3H), 5.07 (s, 1H), 4.36 (dd, *J* = 201.3, 10.4 Hz,2H), 1.39 (s, 18H), 1.13 (s, 9H); **^{13}C NMR (101 MHz, $CDCl_3$)** δ 173.78, 152.63, 139.76, 135.46, 135.26, 130.24, 130.05, 127.80, 127.58, 127.15, 125.56, 80.82, 59.58, 52.51, 34.28, 30.28, 27.61; **HRMS(ESI)**: calcd. for $C_{27}H_{38}ClNO_3(M+H)^+$: 460.2613, found: 460.2611.

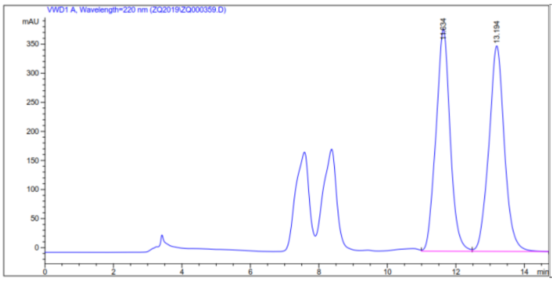

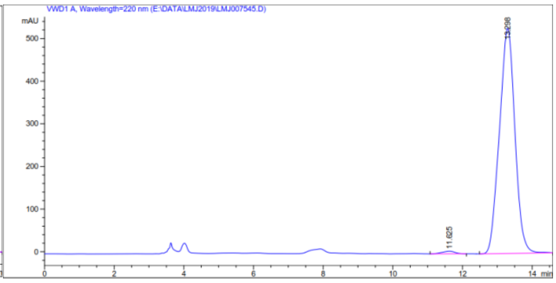

Peak #	RetTime [min]	Type	Width [min]	Area [mAU*s]	Height [mAU]	Area %
1	11.634	VV	0.4268	1.10233e4	382.27499	49.7318
2	13.194	VBA	0.4667	1.11422e4	353.62903	50.2682

Peak #	RetTime [min]	Type	Width [min]	Area [mAU*s]	Height [mAU]	Area %
1	11.625	BB	0.3860	154.24440	5.96720	0.9375
2	13.298	BBA	0.4592	1.62993e4	528.01105	99.0625

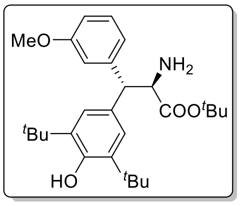

***tert*-butyl (2*R*,3*R*)-2-amino-3-(3,5-di-*tert*-butyl-4-hydroxyphenyl)-3-(3-methoxyphenyl)propanoate (*anti*-5e):** white soild (31.3 mg, 69%); m.p. = 96-97 °C; R_f = 0.33 (petroleum ether/EtOAc = 4:1); the enantiomeric excess was determined to be 96% by HPLC analysis on Daicel Chirapak IA-H column (hexane/isopropanol = 98/2, flow rate 1.0 mL/min, T = 30°C), UV 220 nm, t_R(major) 16.28 min, t_R(minor) 18.19 min; $[a]_D^{25}$ = -101.3 (c = 0.45, $CHCl_3$); **^{1}H NMR (400 MHz, $CDCl_3$)** δ 7.22 (d, *J* = 7.9 Hz, 1H), 7.11 (s, 2H), 6.97 (d, *J* = 7.6 Hz, 1H), 6.92 (s, 1H), 6.75 (dd, *J* = 8.2, 2.2 Hz, 1H), 5.05 (s, 1H), 3.95 (dd, *J* = 65.9, 10.1 Hz, 2H), 3.79 (s, 3H), 1.40 (s, 18H), 1.14 (s, 9H); **^{13}C NMR (101 MHz, $CDCl_3$)** δ 173.84, 159.79, 152.59, 143.61, 135.52, 131.82, 129.72, 124.95, 120.55, 114.44, 111.88, 80.73, 59.91, 58.24, 55.14, 34.28, 30.28, 27.62; **HRMS(ESI)**: calcd. for $C_{28}H_{42}NO_4(M+H)^+$: 456.3108, found: 456.3107.

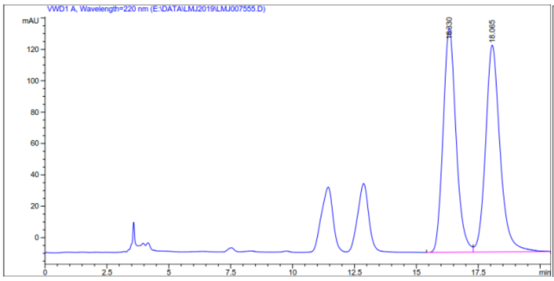

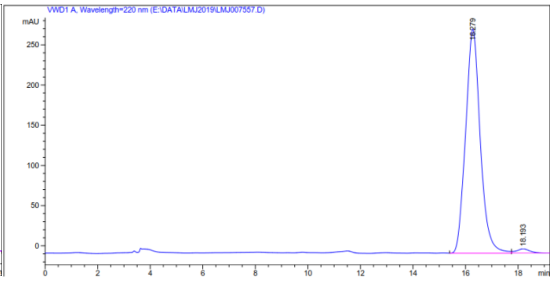

Peak #	RetTime [min]	Type	Width [min]	Area [mAU*s]	Height [mAU]	Area %
1	16.279	VV	0.5767	1.07891e4	278.80362	98.1793
2	18.193	VBA	0.5103	200.07544	5.63503	1.8207

Peak #	RetTime [min]	Type	Width [min]	Area [mAU*s]	Height [mAU]	Area %
1	16.330	BV	0.5518	5185.84717	142.80801	49.2133
2	18.065	VBA	0.6076	5351.63818	131.81631	50.7867

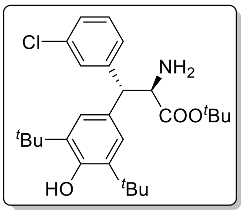

***tert*-butyl (2*R*,3*R*)-2-amino-3-(3-chlorophenyl)-3-(3,5-di-*tert*-butyl-4-hydroxyphenyl)propanoate (*anti*-5f):** light yellow oil (38.3 mg, 83%); R_f = 0.39 (petroleum ether/EtOAc = 4:1); tthe enantiomeric excess was determined to be 96% by HPLC analysis on Daicel Chirapak IA-H column (hexane/isopropanol = 98/2, flow rate 1.0 mL/min, T = 30 ^{o}C), UV 220 nm, t_R(major) 17.16 min, t_R(minor) 18.87 min; $[a]_D^{25}$ = -131.7 (c = 0.48, $CHCl_3$); **^{1}H NMR (600 MHz, $CDCl_3$)** δ 7.35 (s, 1H), 7.25 (dt, *J* = 18.5, 7.7 Hz, 2H), 7.18 (d, *J* = 7.7 Hz, 1H), 7.08 (s, 2H), 5.08 (s, 1H), 3.99 (dd, *J* = 58.6, 9.5 Hz, 2H), 1.40 (s, 18H), 1.16 (s, 9H); **^{13}C NMR (151 MHz, $CDCl_3$)** δ 173.61, 152.73, 144.13, 135.78, 134.39, 131.29, 129.83, 128.87, 126.82, 126.45, 125.00, 80.91, 59.66, 57.17, 34.32, 30.27, 27.64; **HRMS(ESI)**: calcd. for $C_{27}H_{39}ClNO_3(M+H)^+$: 460.2613, found: 460.2612.

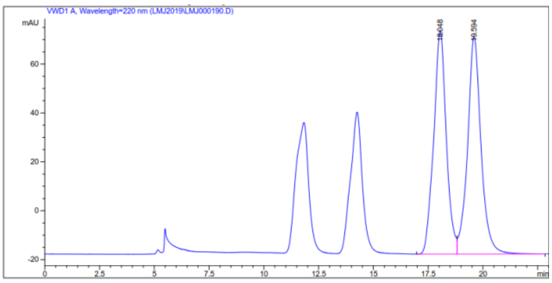

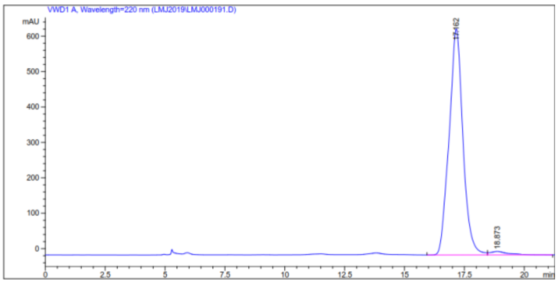

Peak #	RetTime [min]	Type	Width [min]	Area [mAU*s]	Height [mAU]	Area %
1	17.162	BV	0.5822	2.56257e4	638.10223	98.0235
2	18.873	VB	0.7289	516.70996	9.82198	1.9765

Peak #	RetTime [min]	Type	Width [min]	Area [mAU*s]	Height [mAU]	Area %
1	18.048	BV	0.5847	3651.53784	91.59148	48.9569
2	19.594	VB	0.6290	3807.13550	88.96942	51.0431

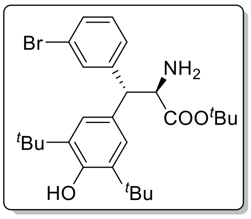

***tert*-butyl (2*R*,3*R*)-2-amino-3-(3-bromophenyl)-3-(3,5-di-*tert*-butyl-4-hydroxyphenyl)propanoate (*anti*-5g):** white soild (34.5 mg, 68%); m.p. = 101-102 ^{o}C; R_f = 0.39 (petroleum ether/EtOAc = 4:1); the enantiomeric excess was determined to be 96% by HPLC analysis on Daicel Chirapak IA-H column (hexane/isopropanol = 98/2, flow rate 1.0 mL/min, T = 30 ^{o}C), UV 220 nm, t_R(major) 12.10 min, t_R(minor) 13.46 min; $[a]_D^{25}$ = -43.4 (c = 0.44, $CHCl_3$); **^{1}H NMR (400 MHz, $CDCl_3$)** δ 7.51 (s, 1H), 7.33 (t, *J* = 7.3 Hz, 2H), 7.18 (t, *J* = 7.8 Hz, 1H), 7.08 (s, 2H), 5.09 (s, 1H), 3.97 (dd, *J* = 44.8, 9.6 Hz,2H), 1.40 (s, 18H), 1.16 (s, 9H); **^{13}C NMR (101 MHz, $CDCl_3$)** δ 173.64, 152.75, 144.40, 135.73, 131.78, 131.20, 130.19, 129.77, 126.85, 124.99, 122.72, 80.95,, 59.66, 57.22, 34.32, 30.26, 27.63; **HRMS(ESI)**: calcd. for $C_{27}H_{39}BrNO_3(M+H)^+$: 504.2108, found: 504.2103.

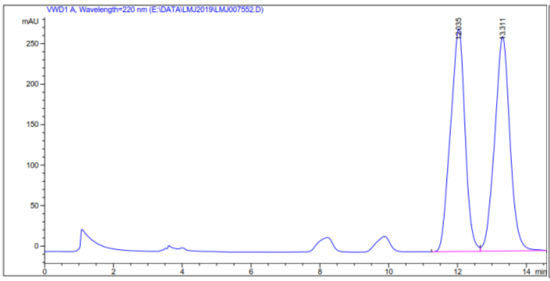

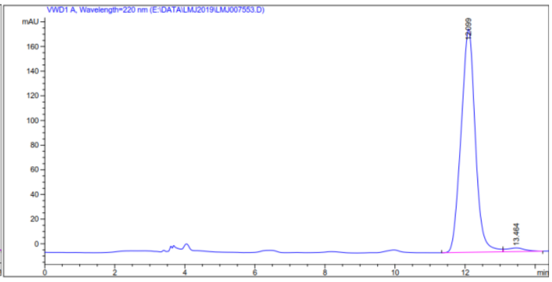

Peak #	RetTime [min]	Type	Width [min]	Area [mAU*s]	Height [mAU]	Area %
1	12.035	BV	0.4312	8073.69141	275.07635	49.8339
2	13.311	VBA	0.4573	8127.50098	264.64758	50.1661

Peak #	RetTime [min]	Type	Width [min]	Area [mAU*s]	Height [mAU]	Area %
1	12.099	BV	0.4216	5099.48535	181.15936	97.9159
2	13.464	VB	0.5226	108.54176	2.94870	2.0841

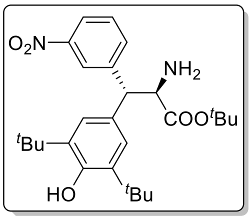

***tert*-butyl (2*R*,3*R*)-2-amino-3-(3,5-di-*tert*-butyl-4-hydroxyphenyl)-3-(3-nitrophenyl)propanoate (*anti*-5h):** white soild (31.8 mg,68%); m.p. = 165-166 ^{o}C; R_f = 0.25 (petroleum ether/EtOAc = 2:1); the enantiomeric excess was determined to be 94% by HPLC analysis on Daicel Chirapak AD-H column (hexane/isopropanol = 95/5, flow rate 1.0 mL/min, T = 30°C), UV 220 nm, t_R(major) 20.49 min, t_R(minor) 18.20 min; $[a]_D^{25}$ = -158.5 (c = 0.40, $CHCl_3$); **^{1}H NMR (400 MHz, $CDCl_3$)** δ 8.29 (t, *J* = 1.8 Hz, 1H), 8.07 (dd, *J* = 8.2, 1.3 Hz, 1H), 7.73 (d, *J* = 7.8 Hz, 1H), 7.48 (t, *J* = 8.0 Hz, 1H), 7.10 (s, 2H), 5.13 (s, 1H), 4.14 – 4.07 (m, 2H), 1.40 (s, 18H), 1.17 (s, 9H); **^{13}C NMR (101 MHz, $CDCl_3$)** δ 173.67, 152.94, 148.34, 144.36, 135.99, 134.85, 130.65, 129.35, 125.06, 123.52, 121.68, 81.19, 59.50, 56.36, 34.35, 30.24, 27.64; **HRMS(ESI)**: calcd. for $C_{27}H_{39}N_2O_5(M+H)^+$: 471.2853, found: 471.2852.

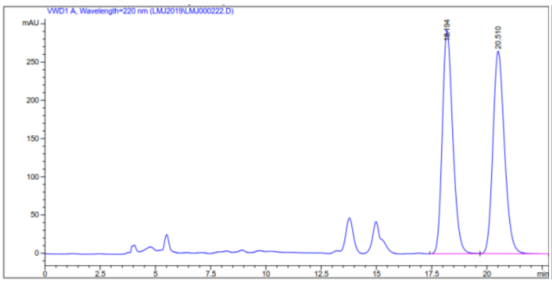

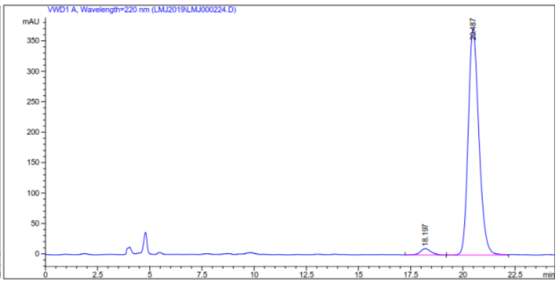

Peak #	RetTime [min]	Type	Width [min]	Area [mAU*s]	Height [mAU]	Area %
1	18.194	BB	0.5060	9607.99316	292.22449	50.0219
2	20.510	BBA	0.5630	9599.58594	264.70895	49.9781

Peak #	RetTime [min]	Type	Width [min]	Area [mAU*s]	Height [mAU]	Area %
1	18.197	BB	0.5271	361.77161	10.28290	2.6666
2	20.487	BV	0.5493	1.32049e4	371.06927	97.3334

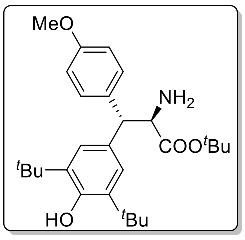

***tert*-butyl (2*R*,3*S*)-2-amino-3-(3,5-di-*tert*-butyl-4-hydroxyphenyl)-3-(4-methoxyphenyl)propanoate (*anti*-5i):** white soild (28.2 mg, 62%); m.p. = 197-198 ^{o}C; R_f = 0.39 (petroleum ether/EtOAc = 4:1); the enantiomeric excess was determined to be 98% by HPLC analysis on Daicel Chirapak IA-H column (hexane/isopropanol = 98/2, flow rate 1.0 mL/min, T = 30°C), UV 220 nm, t_R(major) 20.77 min, t_R(minor) 24.57 min; $[a]_D^{25}$ = -107.4 (c = 0.50, $CHCl_3$); **^{1}H NMR (600 MHz, $CDCl_3$)** δ 7.29 – 7.25 (m, 2H), 7.09 (s, 2H), 6.85 (d, *J* = 8.6 Hz,2H), 5.03 (s, 1H), 3.94 (dd, *J* = 71.1, 9.7 Hz, 2H), 3.77 (s, 3H), 1.40 (s, 18H), 1.15 (s, 9H); **^{13}C**

NMR (151 MHz, CDCl$_3$) δ 173.95, 158.38, 152.45, 135.56, 134.09, 132.39, 129.35, 124.86, 114.15, 80.66, 60.01, 57.08, 55.23, 34.28, 30.29, 27.65; **HRMS(ESI)**: calcd. for $C_{28}H_{42}NO_4(M+H)^+$: 456.3108, found: 456.3107.

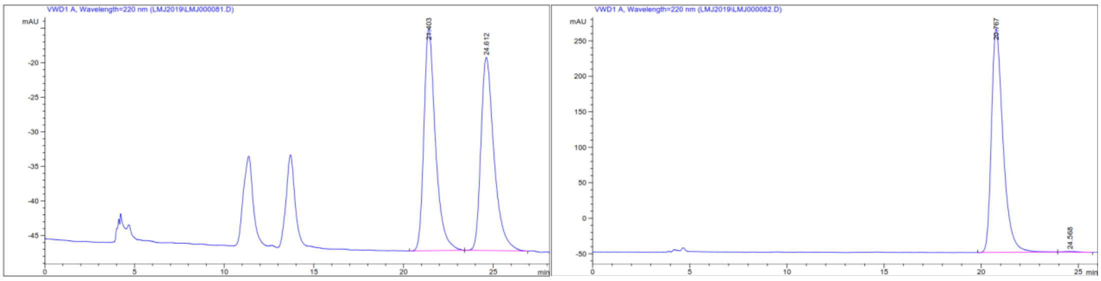

Peak #	RetTime [min]	Type	Width [min]	Area [mAU*s]	Height [mAU]	Area %
1	20.767	BB	0.6311	1.31101e4	315.14575	99.6280
2	24.568	BBA	0.6099	48.95606	1.13779	0.3720

Peak #	RetTime [min]	Type	Width [min]	Area [mAU*s]	Height [mAU]	Area %
1	21.403	BB	0.6707	1437.31372	32.12359	50.2770
2	24.612	BB	0.7656	1421.47778	27.93716	49.7230

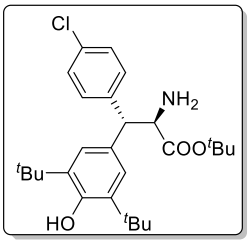

***tert*-butyl (2*R*,3*S*)-2-amino-3-(4-chlorophenyl)-3-(3,5-di-*tert*-butyl-4-hydroxyphenyl)propanoate (*anti*-5j):** light yellow oil (33.9 mg,74%); R_f = 0.24 (petroleum ether/EtOAc = 4:1); the enantiomeric excess was determined to be 97% by HPLC analysis on Daicel Chirapak IA-H column (hexane/isopropanol = 98/2, flow rate 1.0 mL/min, T = 30°C), UV 220 nm, t_R(major) 14.37 min, t_R(minor) 20.77 min; $[a]_D^{25}$ = -50.1 (c = 0.4, CHCl$_3$); **^{1}H NMR (400 MHz, CDCl$_3$)** δ 7.30 (t, *J* = 6.6 Hz, 4H), 7.06 (s, 2H), 5.07 (s, 1H), 3.98 (dd, *J* = 34.0, 9.6 Hz,2H), 1.40 (s,18H), 1.16 (s, 9H); **^{13}C NMR (101 MHz, CDCl$_3$)** δ 173.79, 152.66, 140.56, 135.70, 132.43, 131.53, 129.77, 128.77, 124.89, 80.91, 59.65, 56.81, 34.31, 30.26, 27.64; **HRMS(ESI)**: calcd. for $C_{27}H_{38}ClNO_3(M+H)^+$: 460.2613, found: 460.2611.

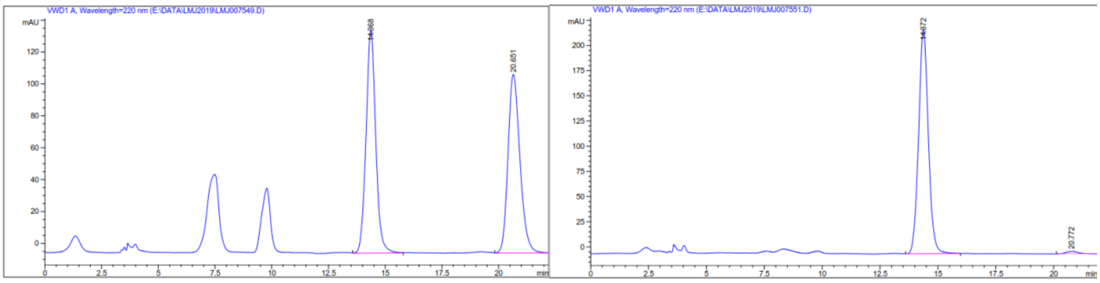

Peak #	RetTime [min]	Type	Width [min]	Area [mAU*s]	Height [mAU]	Area %
1	14.368	VV	0.4456	4161.02051	140.05519	50.4286
2	20.651	VBA	0.5658	4090.28979	112.01823	49.5714

Peak #	RetTime [min]	Type	Width [min]	Area [mAU*s]	Height [mAU]	Area %
1	14.372	VB	0.4438	6508.72656	223.06654	98.5697
2	20.772	BBA	0.5509	94.44389	2.50298	1.4303

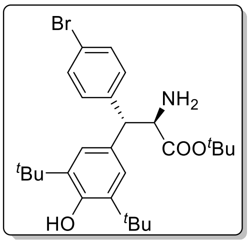

***tert*-butyl (2*R*,3*S*)-2-amino-3-(4-bromophenyl)-3-(3,5-di-*tert*-butyl-4-hydroxyphenyl)propanoate (*anti*-5k):** light yellow oil (37.9 mg, 75%); R_f = 0.18 (petroleum ether/EtOAc = 4:1); the enantiomeric excess was determined to be 97% by HPLC analysis on Daicel Chirapak IA-H column (hexane/isopropanol = 98/2, flow rate 1.0 mL/min, T = 30°C),

UV 220 nm, t_R(major) 20.840 min, t_R(minor) 29.79 min; $[a]_D^{25}$ = -125.7 (c = 0.44, $CHCl_3$); **^{1}H NMR (600 MHz, $CDCl_3$)** δ 7.43 (d, *J* = 8.4 Hz, 2H), 7.25 (d, *J* = 8.4 Hz, 2H), 7.07 (d, *J* = 8.0 Hz, 2H), 5.07 (s, 1H), 3.98 (dd, *J* = 57.1, 9.5 Hz, 2H), 1.39 (s, 18H), 1.16 (s, 9H); **^{13}C NMR (151 MHz, $CDCl_3$)** δ 173.71, 152.67, 141.11, 135.77, 131.70, 131.48, 130.18, 124.89, 120.53, 80.90, 59.58, 56.82, 34.30, 30.31, 30.27, 27.70, 27.65; **HRMS(ESI)**: calcd. for $C_{27}H_{39}BrNO_3(M+H)^+$: 504.2108, found: 504.2109.

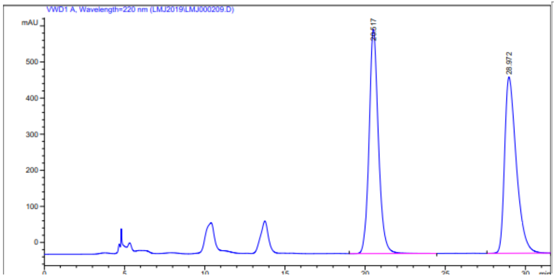

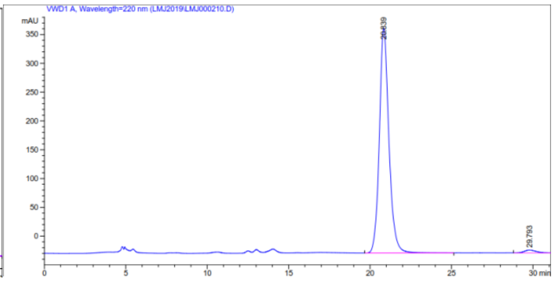

Peak #	RetTime [min]	Type	Width [min]	Area [mAU*s]	Height [mAU]	Area %
1	20.839	BB	0.6124	1.59012e4	390.24524	98.5173
2	29.793	BBA	0.7481	239.31302	4.79828	1.4827

Peak #	RetTime [min]	Type	Width [min]	Area [mAU*s]	Height [mAU]	Area %
1	20.517	BB	0.6052	2.50300e4	621.74762	50.2615
2	28.972	BBA	0.7723	2.47696e4	488.51520	49.7385

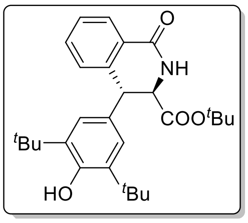

***tert*-butyl (3*R*,4*R*)-4-(3,5-di-*tert*-butyl-4-hydroxyphenyl)-1-oxo-1,2,3,4-tetrahydroisoquinoline-3-carboxylate (*anti*-5l):** light yellow oil (27.1 mg, 62%); R_f = 0.25 (petroleum ether/EtOAc = 4:1); the enantiomeric excess was determined to be 96% by HPLC analysis on Daicel Chirapak IA-H column (hexane/isopropanol = 80/20, flow rate 1.0 mL/min, T = 30°C), UV 220 nm, t_R(major) 13.05 min, t_R(minor) 8.040 min; $[a]_D^{25}$ = -172.2 (c = 0.44, $CHCl_3$); **^{1}H NMR (600 MHz, $CDCl_3$)** δ 8.15 (d, *J* = 7.6 Hz, 1H), 7.41 (dt, *J* = 26.4, 7.4 Hz, 2H), 7.06 (d, *J* = 7.4 Hz, 1H), 6.90 (s, 2H), 6.05 (s, 1H), 5.17 (s, 1H), 4.52 (d, *J* = 3.8 Hz, 1H), 4.27 (d, *J* = 3.7 Hz, 1H), 1.37 (s, 18H), 1.24 (s, 9H); **^{13}C NMR (151 MHz, $CDCl_3$)** δ 170.19, 165.24, 153.01, 139.31, 136.06, 132.57, 130.71, 128.64, 128.21, 127.74, 127.56, 125.02, 82.53, 61.19, 47.12, 34.34, 30.22, 27.68; **HRMS(ESI)**: calcd. for $C_{28}H_{38}NO_4(M+H)^+$: 452.2795, found: 452.2794.

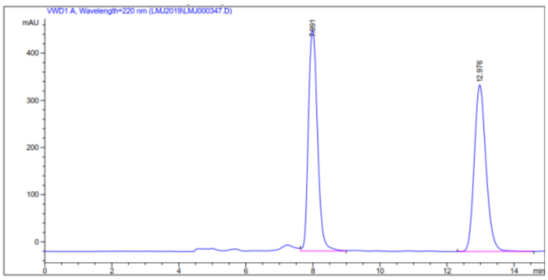

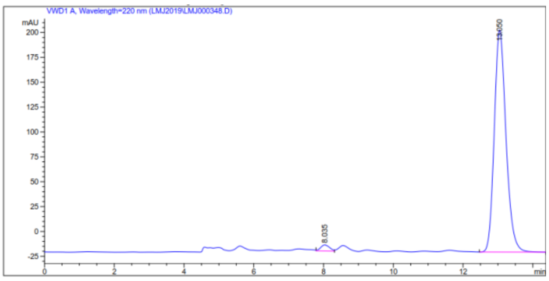

Peak #	RetTime [min]	Type	Width [min]	Area [mAU*s]	Height [mAU]	Area %
1	7.991	VV	0.3000	8891.75000	468.90411	50.8495
2	12.976	BB	0.3756	8594.65332	353.41989	49.1505

Peak #	RetTime [min]	Type	Width [min]	Area [mAU*s]	Height [mAU]	Area %
1	8.035	VV	0.2833	110.07631	6.10430	2.0657
2	13.050	BBA	0.3587	5218.62939	223.30499	97.9343

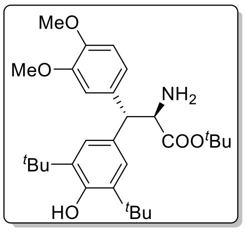

***tert*-butyl (2*R*,3*R*)-2-amino-3-(3,5-di-*tert*-butyl-4-hydroxyphenyl)-3-(3,4-dimethoxyphenyl)propanoate (*anti*-5m):** light yellow oil (27.1 mg, 56%); R_f = 0.15 (petroleum ether/EtOAc = 3:1); the enantiomeric excess was determined to be 98% by HPLC analysis after being converted to corresponding N-Boc derivatives. HPLC: Daicel Chirapak AD-H column (hexane/isopropanol = 95/5, flow rate 1.0 mL/min, T = 30°C), UV 220 nm, t_R(major) 10.19 min, t_R(minor) 19.93 min; $[a]_D^{25}$ = -82.5 (c = 0.24, $CHCl_3$); **^{1}H NMR (400 MHz, $CDCl_3$)** δ 7.12 (s, 2H), 6.95 – 6.88 (m, 2H), 6.82 (d, *J* = 8.8 Hz, 1H), 5.05 (s, 1H), 4.00 (d, *J* = 9.8 Hz, 1H), 3.86 (d, *J* = 13.6 Hz,7H), 1.40 (s,18H), 1.15 (s,9H); **^{13}C NMR (101 MHz, $CDCl_3$)** δ 173.88, 152.54, 149.00, 147.85, 135.53, 134.51, 132.14, 124.85, 120.49, 111.52, 111.43, 80.74, 60.11, 57.57, 55.89, 55.79, 34.29, 30.29, 27.64; **HRMS(ESI)**: calcd. for $C_{29}H_{44}NO_5(M+H)^+$: 486.3214, found: 486.1213.

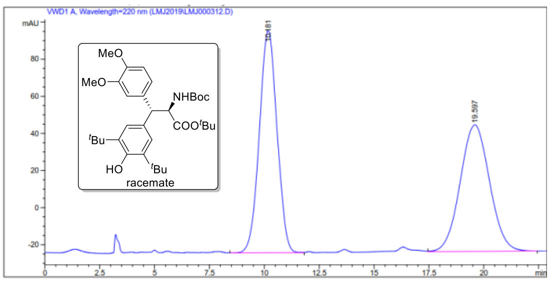

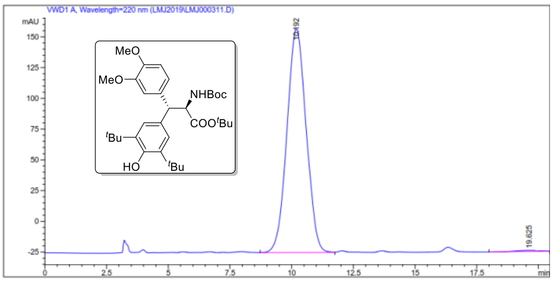

Peak #	RetTime [min]	Type	Width [min]	Area [mAU*s]	Height [mAU]	Area %
1	10.192	BV	0.8324	9832.12500	181.71533	99.3688
2	19.625	BBA	0.9057	62.45866	9.03392e-1	0.6312

Peak #	RetTime [min]	Type	Width [min]	Area [mAU*s]	Height [mAU]	Area %
1	10.181	BV	0.8567	6695.46436	119.68624	51.3767
2	19.597	VB	1.4628	6336.63965	68.21053	48.6233

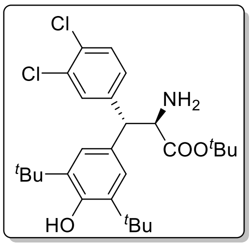

***tert*-butyl (2*R*,3*R*)-2-amino-3-(3,5-di-*tert*-butyl-4-hydroxyphenyl)-3-(3,4-dichlorophenyl)propanoate (*anti*-5n):** white soild (37.8 mg, 77%); m.p. = 129-130 ^{o}C; R_f = 0.25 (petroleum ether/EtOAc = 4:1); the enantiomeric excess was determined to be 97% by HPLC analysis on Daicel Chirapak IA-H column (hexane/isopropanol = 98/2, flow rate 1.0 mL/min, T = 30°C), UV 220 nm, t_R(major) 19.11 min, t_R(minor) 24.47 min; $[a]_D^{25}$ = -117.7 (c = 0.76, $CHCl_3$); **^{1}H NMR (600 MHz, $CDCl_3$)** δ 7.46 (d, *J* = 1.8 Hz, 1H), 7.37 (d, *J* = 8.3 Hz, 1H), 7.23 (dd, *J* = 8.3, 1.9 Hz, 1H), 7.06 (s, 2H), 5.11 (s, 1H), 3.98 (dd, *J* = 40.1, 9.4 Hz, 2H), 1.40 (s, 18H), 1.17 (s, 9H); **^{13}C NMR (151 MHz, $CDCl_3$)** δ 173.34, 152.59, 142.32, 135.65, 131.44, 127.77, 125.74, 125.10, 121.82, 80.87, 60.34, 52.89, 34.30, 30.30, 27.66; **HRMS(ESI)**: calcd. for $C_{27}H_{38}Cl_2NO_3(M+H)^+$: 494.2223, found: 494.2225.

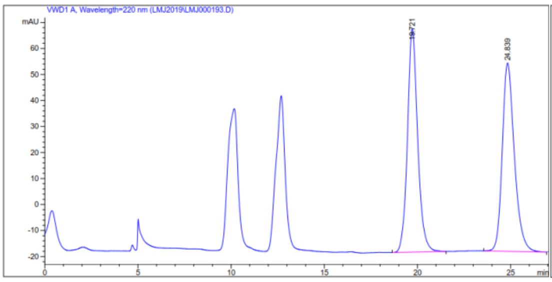

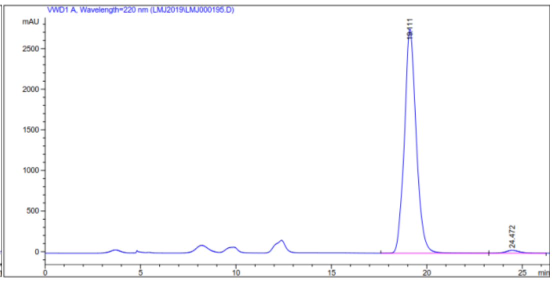

Peak #	RetTime [min]	Type	Width [min]	Area [mAU*s]	Height [mAU]	Area %
1	19.721	BB	0.5729	3270.45435	85.80759	50.0041
2	24.839	BBA	0.6851	3269.91650	72.49138	49.9959

Peak #	RetTime [min]	Type	Width [min]	Area [mAU*s]	Height [mAU]	Area %
1	19.111	BB	0.6386	1.18938e5	2758.53833	98.5877
2	24.472	BB	0.7385	1703.80212	34.82309	1.4123

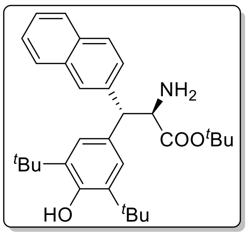

***tert*-butyl (2*R*,3*S*)-2-amino-3-(3,5-di-*tert*-butyl-4-hydroxyphenyl)-3-(naphthalen-2-yl)propanoate (*anti*-5o):** light yellow oil (37.9 mg, 80%); R_f = 0.41 (petroleum ether/EtOAc = 4:1); the enantiomeric excess was determined to be 94% by HPLC analysis on Daicel Chirapak IA-H column (hexane/isopropanol = 98/2, flow rate 1.0 mL/min, T = 30°C), UV 220 nm, t_R(major) 18.00 min, t_R(minor) 20.56 min; $[a]_D^{25}$ **=** -100.5 (c = 0.60, $CHCl_3$); **^{1}H NMR (600 MHz, $CDCl_3$)** δ 7.82 – 7.75 (m, 4H), 7.51 (dd, *J* = 8.5, 1.6 Hz, 1H), 7.47 – 7.39 (m, 2H), 7.17 (s, 2H), 5.04 (s, 1H), 4.15 (dd, *J* = 44.3, 9.8 Hz, 2H), 1.40 (s, 18H), 1.17 (s, 9H); **^{13}C NMR (151 MHz, $CDCl_3$)** δ 173.87, 152.59, 139.45, 135.67, 133.62, 132.45, 131.95, 128.43, 127.77, 127.56, 127.15, 126.47, 126.05, 125.58, 125.04, 80.77, 59.77, 58.04, 34.30,30.30,27.68; **HRMS(ESI)**: calcd. for $C_{31}H_{42}NO_3(M+H)^+$: 476.3159, found: 476.3158.

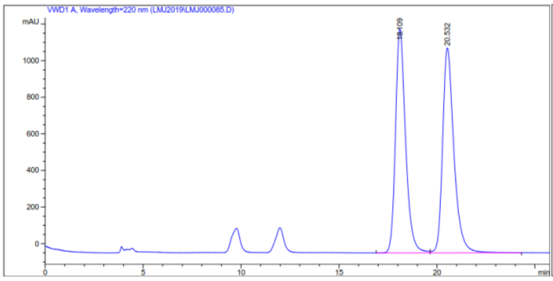

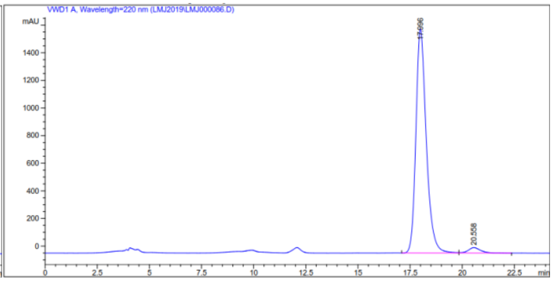

Peak #	RetTime [min]	Type	Width [min]	Area [mAU*s]	Height [mAU]	Area %
1	18.109	BV	0.5389	4.36746e4	1223.52966	49.2599
2	20.532	VV	0.6085	4.49870e4	1120.11243	50.7401

Peak #	RetTime [min]	Type	Width [min]	Area [mAU*s]	Height [mAU]	Area %
1	17.996	BV	0.5254	5.60882e4	1623.73035	97.1691
2	20.558	VB	0.6249	1634.05359	39.07453	2.8309

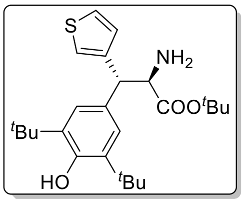

***tert*-butyl (2*R*,3*R*)-2-amino-3-(3,5-di-*tert*-butyl-4-hydroxyphenyl)-3-(thiophen-3-yl)propanoate (*anti*-5p):** white soild (28.7 mg, 67%); m.p. = 179-180 °C; R_f = 0.14 (petroleum ether/EtOAc = 4:1); the enantiomeric excess was determined to be 98% by HPLC analysis on Daicel Chirapak IA-H column (hexane/isopropanol = 98/2, flow rate 1.0 mL/min, T = 30°C), UV 220 nm, t_R(major) 13.06 min, t_R(minor) 15.92 min; $[a]_D^{25}$ **=** -106.1 (c = 0.34, $CHCl_3$); **^{1}H NMR (600 MHz, $CDCl_3$)** δ 7.29 – 7.25 (m, 1H), 7.18 (d, *J* = 1.5 Hz, 1H), 7.09 (d, *J* = 5.6 Hz,

3H), 5.07 (s, 1H), 4.04 (dd, J = 107.2, 8.9 Hz, 2H), 1.40 (s, 18H), 1.16 (s, 9H); **^{13}C NMR (151 MHz, CDCl$_3$)** δ 173.34, 152.59, 142.32, 135.65, 131.44, 127.77, 125.74, 125.10, 121.82, 80.87, 60.34, 52.89, 34.30, 30.30, 27.66; **HRMS(ESI)**: calcd. for $C_{25}H_{38}NO_3S(M+H)^+$: 432.2567, found: 432.2566.

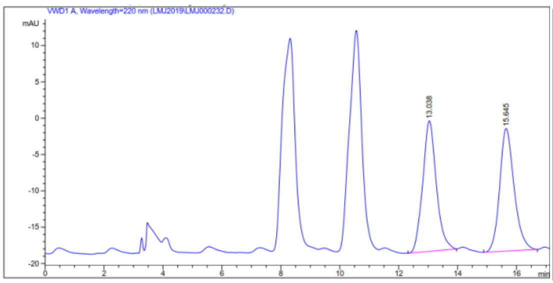

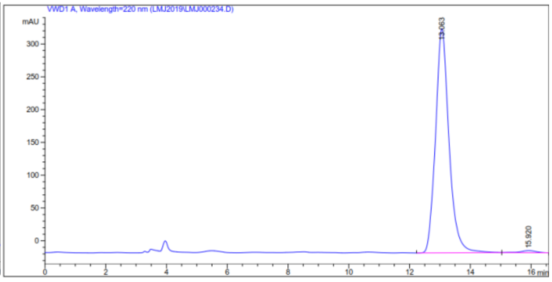

Peak #	RetTime [min]	Type	Width [min]	Area [mAU*s]	Height [mAU]	Area %
1	13.038	BB	0.4754	576.99164	17.95919	50.1543
2	15.645	BB	0.5157	573.44165	16.88731	49.8457

Peak #	RetTime [min]	Type	Width [min]	Area [mAU*s]	Height [mAU]	Area %
1	13.063	BB	0.4558	1.05225e4	341.44235	99.0030
2	15.920	BBA	0.4971	105.96410	3.15287	0.9970

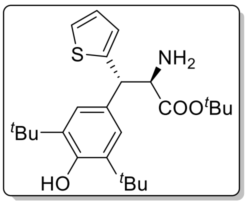

***tert*-butyl (2*R*,3*S*)-2-amino-3-(3,5-di-*tert*-butyl-4-hydroxyphenyl)-3-(thiophen-2-yl)propanoate (*anti*-5q):** white solid (30.7 mg, 71%); m.p. = 187-188 ^{o}C; R$_f$ = 0.33 (petroleum ether/EtOAc = 4:1); the enantiomeric excess was determined to be 58% by HPLC analysis on Daicel Chirapak IA-H column (hexane/isopropanol = 98/2, flow rate 1.0 mL/min, T = 30°C), UV 220 nm, t$_R$(major) 13.82 min, t$_R$(minor) 15.32 min; [a]$_D^{25}$ = -88.3 (c = 0.42, CHCl$_3$); **^{1}H NMR (600 MHz, CDCl$_3$)** δ 7.18 (d, J = 5.0 Hz, 1H), 7.15 (s, 2H), 6.96 (d, J = 3.1 Hz, 1H), 6.95 – 6.90 (m, 1H), 5.09 (s, 1H), 4.25 (d, J = 8.9 Hz, 1H), 3.88 (d, J = 8.9 Hz, 1H), 1.41 (s, 18H), 1.16 (s, 9H); **^{13}C NMR (151 MHz, CDCl$_3$)** δ 173.05, 152.78, 145.06, 135.67, 131.35, 126.49, 125.79, 125.10, 124.46, 80.93, 61.35, 53.33, 34.32, 30.29, 27.63; **HRMS(ESI)**: calcd. for $C_{25}H_{38}NO_3S(M+H)^+$: 432.2567, found: 432.2567.

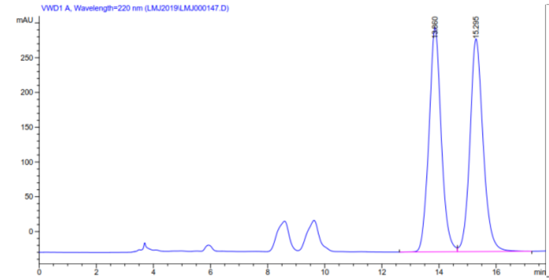

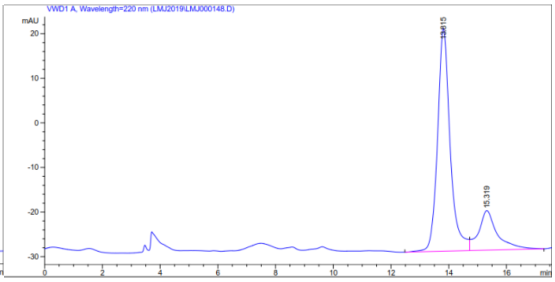

Peak #	RetTime [min]	Type	Width [min]	Area [mAU*s]	Height [mAU]	Area %
1	13.860	BV	0.4465	9651.51758	322.77133	49.6122
2	15.295	VB	0.4829	9802.40332	306.11749	50.3878

Peak #	RetTime [min]	Type	Width [min]	Area [mAU*s]	Height [mAU]	Area %
1	13.815	BV	0.4487	1517.16797	50.01452	79.2704
2	15.319	VB	0.6238	396.74756	8.87201	20.7296

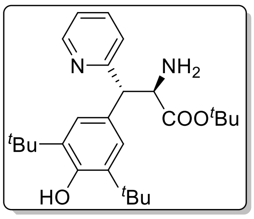

***tert*-butyl (2*R*,3*S*)-2-amino-3-(3,5-di-*tert*-butyl-4-hydroxyphenyl)-3-(pyridin-2-yl)propanoate (*anti*-5r):** white solid (24.7 mg, 58%); m.p. = 124-125 ^{o}C; R$_f$ = 0.30 (petroleum ether/EtOAc = 2:1); the enantiomeric excess was determined to be 89% by HPLC analysis after being

converted to the corresponding N-Boc derivatives. HPLC: Daicel Chirapak IA-H column (hexane/isopropanol = 80/20, flow rate 1.0 mL/min, T = 30°C), UV 220 nm, t_R(major) 4.20 min, t_R (minor) 5.02 min; $[a]_D^{25}$ = +16.9 (c = 0.48, $CHCl_3$); **^{1}H NMR (600 MHz, $CDCl_3$)** δ 8.55 (d, *J* = 4.1 Hz, 1H), 7.60 (td, *J* = 7.7, 1.7 Hz, 1H), 7.37 (d, *J* = 7.9 Hz, 1H), 7.20 (s,2H), 7.15 – 7.06 (m, 1H), 5.11 (s, 1H), 3.31 – 3.24 (m,2H), 1.45 (s, 18H), 1.40 (s,9H); **^{13}C NMR (151 MHz, $CDCl_3$)** δ 171.61, 162.70, 153.11, 149.05, 136.46, 135.87, 132.52, 124.53, 121.82, 121.77, 81.03, 68.14, 49.91, 34.36, 30.33, 28.15; **HRMS(ESI)**: calcd. for $C_{26}H_{39}N_2O_3(M+H)^+$: 427.2955, found: 427.2955.

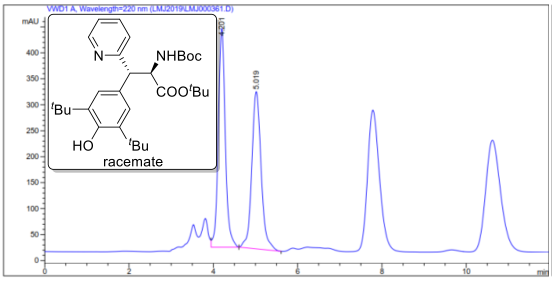

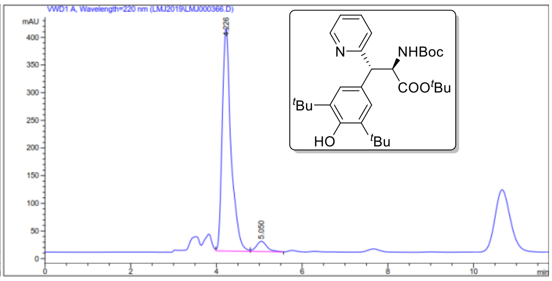

Peak #	RetTime [min]	Type	Width [min]	Area [mAU*s]	Height [mAU]	Area %
1	4.226	VV	0.2163	5746.61719	402.01196	94.7182
2	5.050	VB	0.2599	320.45261	18.68128	5.2818

Peak #	RetTime [min]	Type	Width [min]	Area [mAU*s]	Height [mAU]	Area %
1	4.201	VB	0.1788	4878.63330	421.33469	51.1068
2	5.019	BB	0.2366	4667.31494	302.89630	48.8932

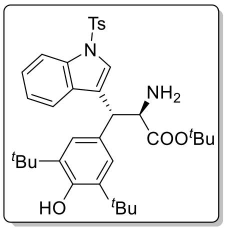

***tert*-butyl (2*R*,3*R*)-2-amino-3-(3,5-di-*tert*-butyl-4-hydroxyphenyl)-3-(1-tosyl-1H-inden-3-yl)propanoate (*anti*-5s):** light yellow oil (48.1 mg, 68%); R_f = 0.28 (petroleum ether/ EtOAc = 2:1); the enantiomeric excess was determined to be 93% by HPLC analysis on Daicel Chirapak IA-H column (hexane/isopropanol = 90/10, flow rate 1.0 mL/min, T = 30°C), UV 220 nm, t_R(major) 13.41 min, t_R(minor) 15.66 min ; $[a]_D^{25}$ = -188.4 (c = 0.35, $CHCl_3$); **^{1}H NMR (600 MHz, $CDCl_3$)** δ 7.97 (d, *J* = 8.3 Hz, 1H), 7.80 – 7.74 (m, 3H), 7.44 (d, *J* = 7.9 Hz, 1H), 7.29 – 7.23 (m, 2H), 7.17 (dd, *J* = 13.4, 7.7 Hz, 3H), 7.07 (s, 2H), 5.05 (s, 1H), 4.35 (d, *J* = 7.8 Hz, 1H), 4.06 (d, *J* = 7.8 Hz, 1H), 2.31 (s, 3H), 1.36 (s, 18H), 1.08 (s, 9H); **^{13}C NMR (151 MHz, $CDCl_3$)** δ 172.78, 152.70, 144.69, 135.66, 135.50, 135.07, 131.19, 130.06, 129.84, 126.76, 125.14, 124.67, 123.15, 122.99, 119.92, 113.62, 81.03, 59.76, 47.65, 34.27, 30.28, 27.60; **HRMS(ESI)**: calcd. for $C_{36}H_{47}N_2O_5S(M+H)^+$: 619.3200, found: 619.3205.

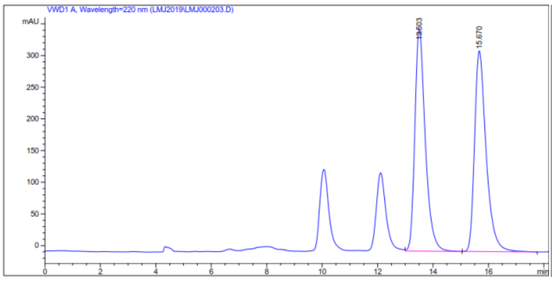

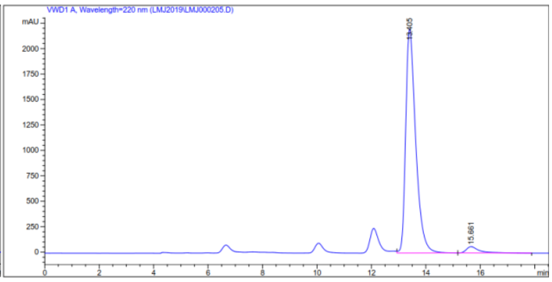

Peak #	RetTime [min]	Type	Width [min]	Area [mAU*s]	Height [mAU]	Area %	Peak #	RetTime [min]	Type	Width [min]	Area [mAU*s]	Height [mAU]	Area %
1	13.503	VB	0.3926	9179.39648	350.98264	49.8963	1	13.405	VB	0.3878	5.60865e4	2200.21802	96.4765
2	15.670	BB	0.4386	9217.53613	316.81357	50.1037	2	15.661	BB	0.4853	2048.40112	61.41495	3.5235

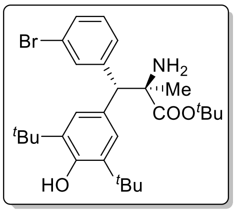

***tert*-butyl (2*R*,3*R*)-2-amino-3-(3-bromophenyl)-3-(3,5-di-*tert*-butyl-4-hydroxyphenyl)-2-methylpropanoate (*anti*-5t):** light yellow oil (31.1 mg, 60%); R_f = 0.40 (petroleum ether/ EtOAc = 10:1); the enantiomeric excess was determined to be 95% by HPLC analysis on Daicel Chirapak AD-H column (hexane/isopropanol = 99/1, flow rate 1.0 mL/min, T = 30°C), UV 220 nm, t_R(major) 9.98 min, t_R(minor) 11.53 min; $[a]_D^{25}$ = -96.5 (c = 0.40, $CHCl_3$); **^{1}H NMR (600 MHz, $CHCl_3$)** δ 7.70 (s, 1H), 7.66 (d, *J* = 7.8 Hz, 1H), 7.28 (dt, *J* = 13.4, 3.5 Hz, 3H), 7.12 (t, *J* = 7.9 Hz, 1H), 5.09 (s, 1H), 4.12 (s, 1H), 1.42 (s,18H), 1.32 (s, 3H), 1.25 (s,9H); **^{13}C NMR (151 MHz, $CDCl_3$)** δ 175.46, 152.68, 145.09, 135.29, 132.72, 129.73, 129.48, 129.22, 127.78, 126.96, 121.88, 81.22, 61.22, 57.98, 34.36, 30.39, 27.63; **HRMS(ESI)**: calcd. for $C_{28}H_{41}BrNO_3$ $(M+H)^+$: 518.2264, found:518.2263.

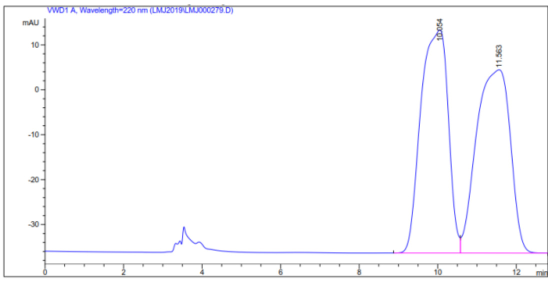

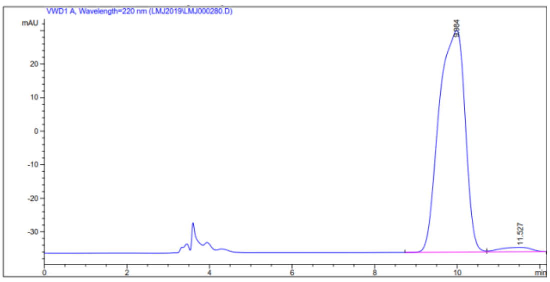

Peak #	RetTime [min]	Type	Width [min]	Area [mAU*s]	Height [mAU]	Area %	Peak #	RetTime [min]	Type	Width [min]	Area [mAU*s]	Height [mAU]	Area %
1	10.054	BV	0.6565	2405.41113	49.69618	49.6205	1	9.984	BV	0.6045	2880.89209	66.12141	97.6831
2	11.563	VBA	0.8205	2442.20532	40.81828	50.3795	2	11.527	VBA	0.8485	68.33029	1.32712	2.3169

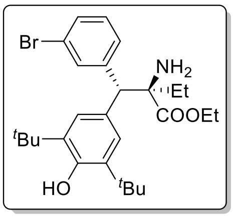

ethyl (*R*)-2-amino-2-((*R*)-(3-bromophenyl)(3,5-di-*tert*-butyl-4-hydroxyphenyl)methyl)butanoate (*anti*-5u): light yellow oil (23 mg, 46%); R_f = 0.46 (petroleum ether/EtOAc = 15:1); the enantiomeric excess was determined to be 88% by HPLC analysis on Daicel Chirapak IA-H column (hexane/isopropanol = 99.9/0.1, flow rate 1.0 mL/min, T = 30 ^{o}C), UV 220 nm, t_R(major) 8.01 min, t_R(minor) 7.60 min; $[a]_D^{25}$ = -64.7 (c =0.30, $CHCl_3$); **^{1}H NMR (600 MHz, $CDCl_3$)** δ 7.69 (s, 1H), 7.64 (d, J = 7.8 Hz, 1H), 7.32 (s, 2H), 7.27 (d, J = 8.1 Hz, 1H), 7.10 (t, J = 7.9 Hz, 1H), 5.08 (s, 1H), 4.17 (s, 1H), 4.07 – 3.97 (m, 2H), 1.85 (dq, J = 14.7, 7.4 Hz, 1H), 1.42 (s, 18H), 1.08 (t, J = 7.1 Hz, 3H), 0.78 (t, J = 7.5 Hz, 3H); **^{13}C NMR (151 MHz, $CDCl_3$)** δ 175.56, 152.67, 144.90, 135.29, 132.61, 129.96, 129.49, 129.29, 127.72, 126.97, 121.92, 65.23, 60.95, 58.36, 34.36, 33.91, 30.40, 14.03, 8.20; **HRMS(ESI)**: calcd. for $C_{27}H_{39}BrNO_3$ $(M+H)^+$: 504.2108, found : 504.2108.

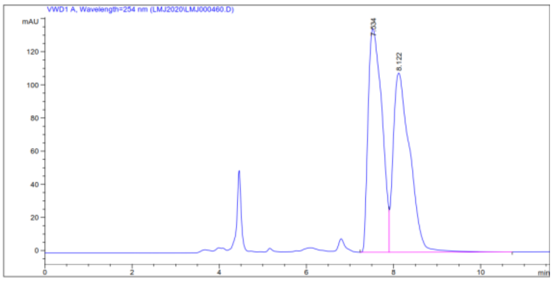

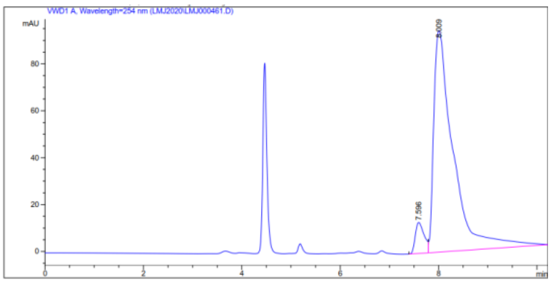

Peak #	RetTime [min]	Type	Width [min]	Area [mAU*s]	Height [mAU]	Area %
1	7.534	BV	0.3726	2963.77808	135.09338	50.6581
2	8.122	VB	0.3813	2886.77002	108.19257	49.3419

Peak #	RetTime [min]	Type	Width [min]	Area [mAU*s]	Height [mAU]	Area %
1	7.596	BV	0.1896	165.60176	13.24824	5.9944
2	8.009	VBA	0.3864	2597.00366	94.44202	94.0056

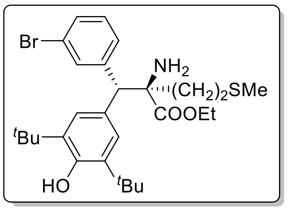

ethyl (*R*)-2-amino-2-((*R*)-(3-bromophenyl)(3,5-di-*tert*-butyl-4-hydroxyphenyl)methyl)-4-(methylthio)butanoate (*anti*-5v): light yellowoil (31.1 mg, 57%); R_f = 0.21 (petroleum ether/EtOAc = 12:1); the enantiomeric excess was determined to be 92% by HPLC analysis on Daicel Chirapak IA-H column (hexane/isopropanol = 99:1, flow rate 1.0 mL/min, T = 30°C), UV 220 nm, t_R(major) 21.73 min, t_R(minor) 24.14 min; $[a]_D^{25}$ = -77.1 (c =0.34, $CHCl_3$); **^{1}H NMR (600 MHz, $CDCl_3$)** δ 7.65 (s, 1H), 7.57 (d, *J* = 7.8 Hz, 1H), 7.33 (s, 2H), 7.29 (d, *J* = 7.9 Hz, 1H), 7.11 (t, *J* = 7.9 Hz, 1H), 5.11 (s, 1H), 4.18 (s, 1H), 4.08 – 3.97 (m, 2H), 2.47 (td, *J* = 12.4, 5.1 Hz, 1H), 2.33 – 2.24 (m, 1H), 2.16 – 2.08 (m, 1H), 1.72 (ddd, *J* = 13.6, 11.5, 5.1 Hz, 1H), 1.42 (s, 18H), 1.09 (t, *J* = 7.1 Hz, 3H); **^{13}C NMR (151 MHz, $CDCl_3$)** δ 175.18, 152.86, 144.18, 135.51, 132.59, 129.57, 129.50, 129.43, 127.71, 126.88, 122.03, 64.65, 61.28, 58.16, 40.29, 34.39, 30.40, 28.88, 15.44, 14.01; **HRMS(ESI)**: calcd. for $C_{28}H_{41}BrNO_3S$ $(M+H)^+$: 550.1985, found: 550.1986.

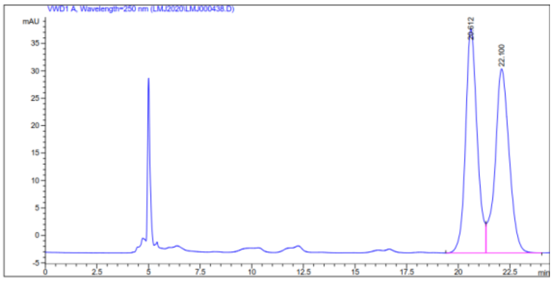

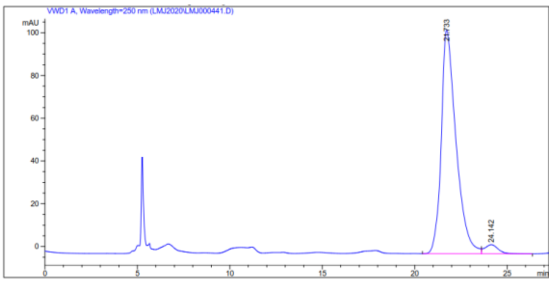

Peak #	RetTime [min]	Type	Width [min]	Area [mAU*s]	Height [mAU]	Area %
1	20.612	BV	0.5970	1596.00903	40.87291	50.0658
2	22.100	VB	0.7172	1591.81396	33.52708	49.9342

Peak #	RetTime [min]	Type	Width [min]	Area [mAU*s]	Height [mAU]	Area %
1	21.733	BV	0.8129	5815.24658	104.43163	96.3943
2	24.142	VB	0.7577	217.52457	4.26918	3.6057

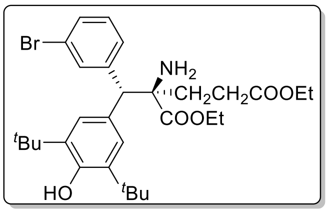

Diethyl (*R*)-2-amino-2-((*R*)-(3-bromophenyl)(3,5-di-*tert*-butyl-4-hydroxyphenyl)methyl)pentanedioate (*anti*-5w): light yellow oil (9.9 mg, 25%); R_f = 0.28 (petroleum ether/EtOAc = 6:1); the enantiomeric excess was determined to be 96% by HPLC analysis on Daicel Chirapak IA-H column (hexane/isopropanol = 99:1, flow rate 1.0 mL/min, T = 30°C), UV 220 nm, t_R(major) 13.61 min, t_R(minor) 15.82 min; $[a]_D^{25}$ = -65.2

(c =0.25, $CHCl_3$); **^{1}H NMR (600 MHz, $CDCl_3$)** δ 7.70 (s, 1H), 7.63 (d, *J* = 7.8 Hz, 1H), 7.28 (t, *J* = 9.7 Hz, 3H), 7.11 (t, *J* = 7.9 Hz, 1H), 5.11 (s, 1H), 4.18 (s, 1H), 4.11 – 4.06 (m, 2H), 4.04 – 3.95 (m, 2H), 2.38 – 2.28 (m, 1H), 2.19 – 2.07 (m, 2H), 1.81 (ddd, *J* = 16.2, 12.0, 5.3 Hz, 1H), 1.42 (s, 18H), 1.21 (t, *J* = 7.1 Hz,3H), 1.05 (t, *J* = 7.1 Hz, 3H); **^{13}C NMR (151 MHz, $CDCl_3$)** δ 174.83, 173.03, 152.86, 144.48, 135.47, 132.58, 129.55, 129.46, 129.25, 127.64, 126.93, 121.99, 64.07, 61.27, 60.46, 58.29, 36.12, 34.37, 30.36, 30.31, 29.41, 14.15, 13.90; **HRMS(ESI)**: calcd. for $C_{30}H_{43}BrNO_5$ (M+H)+: 504.2108, found: 504.2108.

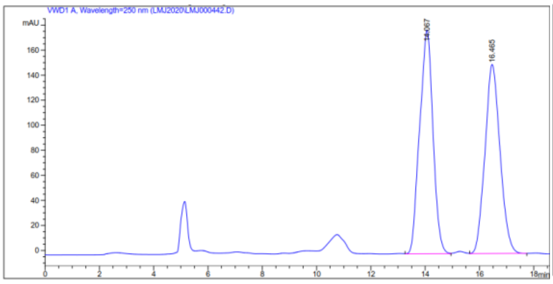

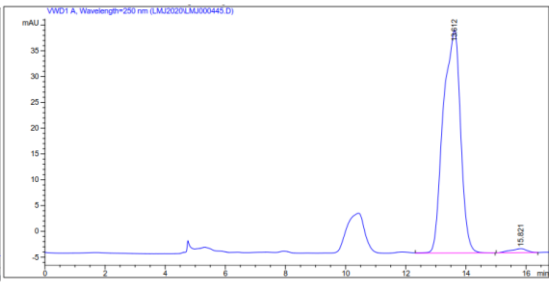

Peak #	RetTime [min]	Type	Width [min]	Area [mAU*s]	Height [mAU]	Area %
1	14.067	VV	0.4918	6105.13574	178.74518	51.1735
2	16.465	VB	0.5755	5825.12891	150.96336	48.8265

Peak #	RetTime [min]	Type	Width [min]	Area [mAU*s]	Height [mAU]	Area %
1	13.612	VB	0.5476	1723.84851	43.15649	98.3631
2	15.821	BB	0.5368	28.68792	7.54579e-1	1.6369

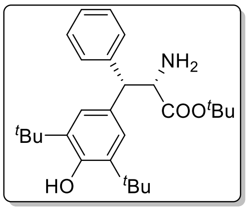

***tert*-butyl (2*S*,3*S*)-2-amino-3-(3,5-di-*tert*-butyl-4-hydroxyphenyl)-3-phenylpropanoate (*syn*-5a):** white soild (29.7mg, 70%); R_f = 0.38 (petroleum ether/EtOAc = 4:1); the enantiomeric excess was determined to be 90% by HPLC analysis on Daicel Chirapak IA-H column (hexane/isopropanol = 98/2, flow rate 1.0 mL/min, T = 30°C), UV 220 nm, t_R(major) 8.11 min, t_R(minor) 9.71 min; $[a]_D^{25}$ = +21.5 (c = 0.40, $CHCl_3$); **^{1}H NMR (600 MHz, $CDCl_3$)** δ 7.34 (d, *J* = 7.4 Hz, 2H), 7.26 (t, *J* = 7.6 Hz, 2H), 7.17 (t, *J* = 7.3 Hz, 1H), 7.13 (s, 2H), 5.08 (s, 1H), 4.01 (dd, *J* = 48.4, 9.4 Hz, 2H), 1.41 (s, 18H), 1.17 (s, 9H); **^{13}C NMR (151 MHz, $CDCl_3$)** δ 173.64, 152.68, 142.32, 136.03, 131.44, 128.47, 128.26, 126.44, 125.02, 80.94, 59.80, 57.66, 34.37, 30.33, 27.61; **HRMS(ESI)**: calcd. for $C_{27}H_{42}NO_3(M+H)^+$: 426.3003, found: 426.3005.

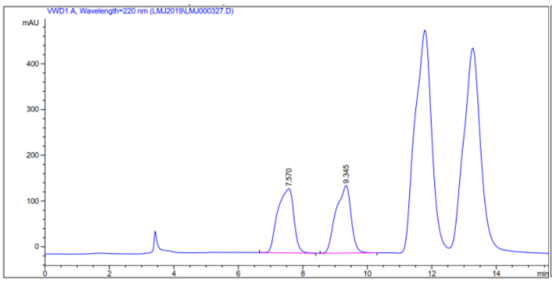

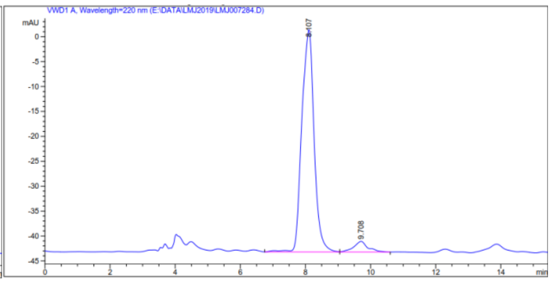

Peak #	RetTime [min]	Type	Width [min]	Area [mAU*s]	Height [mAU]	Area %
1	7.570	BB	0.5848	4842.44092	140.31772	50.4168
2	9.345	BB	0.4386	4762.37988	147.79085	49.5832

Peak #	RetTime [min]	Type	Width [min]	Area [mAU*s]	Height [mAU]	Area %
1	8.107	BV	0.4242	1171.23096	44.61942	94.7663
2	9.708	VB	0.4187	64.68436	2.14577	5.2337

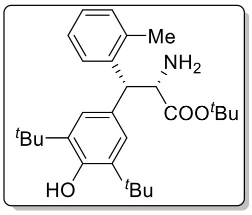

***tert*-butyl (2*S*,3*R*)-2-amino-3-(3,5-di-*tert*-butyl-4-hydroxyphenyl)-3-(o-tolyl)propanoate (*syn*-5b):** white soild (22.8mg, 52%); m.p. = 179-180 °C; R_f = 0.38 (petroleum ether/EtOAc = 4:1); the enantiomeric excess was determined to be 93% by HPLC analysis on Daicel Chirapak AD-H column (hexane/isopropanol = 98/2, flow rate 1.0 mL/min, T = 30°C), UV 220 nm, t_R(major) 9.90 min, t_R(minor) 11.05 min; $[a]_D^{25}$ = +29.6 (c = 0.29, $CHCl_3$); **^{1}H NMR (400 MHz, $CDCl_3$)** δ 7.50 (d, *J* = 7.7 Hz, 1H), 7.21 – 7.14 (m, 1H), 7.12 (s,2H), 7.06 (dd, *J* = 7.2, 5.5 Hz, 2H), 5.07 (s, 1H), 4.12 (dd, *J* = 54.7, 9.7 Hz,2H), 2.36 (s, 3H), 1.40 (s, 18H), 1.16 (s, 9H); **^{13}C NMR (101 MHz, $CDCl_3$)** δ 173.79, 152.60, 140.71, 135.92, 135.88, 130.93, 130.38, 127.31, 126.22, 125.85, 125.21, 80.85, 59.94, 52.36, 34.35, 30.31, 27.51, 20.18; **HRMS(ESI)**: calcd. for $C_{28}H_{42}NO_3$($M+H)^+$: 440.3159, found: 440.3159.

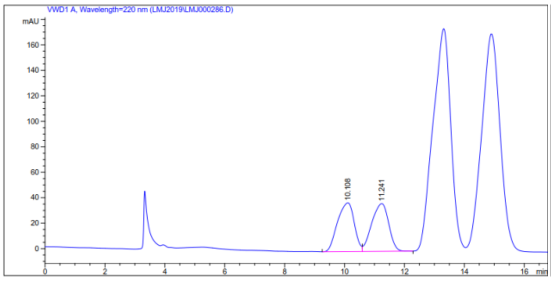

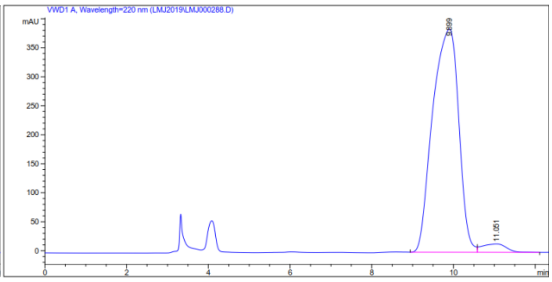

Peak #	RetTime [min]	Type	Width [min]	Area [mAU*s]	Height [mAU]	Area %
1	10.108	BV	0.6441	1483.69397	38.32311	50.1878
2	11.241	VB	0.6476	1472.58838	37.49974	49.8122

Peak #	RetTime [min]	Type	Width [min]	Area [mAU*s]	Height [mAU]	Area %
1	9.899	BV	0.7476	1.70570e4	384.73013	96.6780
2	11.051	VB	0.6491	586.10461	14.33236	3.3220

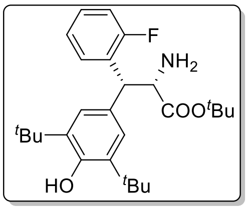

***tert*-butyl (2*S*,3*R*)-2-amino-3-(3,5-di-*tert*-butyl-4-hydroxyphenyl)-3-(2-fluorophenyl)propanoate (*syn*-5c):** white soild (25.6 mg, 58%); R_f = 0.55 (petroleum ether/EtOAc = 4:1); the enantiomeric excess was determined to be 92% by HPLC analysis on Daicel Chirapak IA-H column (hexane/isopropanol = 98/2, flow rate 1.0 mL/min, T = 30°C), UV 220 nm, t_R(major) 9.25 min, t_R(minor) 10.30 min; $[a]_D^{25}$ = +53.1 (c = 0.36, $CHCl_3$); **^{1}H NMR (600 MHz, $CDCl_3$)** δ 7.42 (td, *J* = 7.5, 1.4 Hz, 1H), 7.21 – 7.12 (m, 3H), 7.06 (td, *J* = 7.6, 1.0 Hz, 1H), 7.02 – 6.95 (m, 1H), 5.10 (s, 1H), 4.20 (dd, *J* = 63.9, 9.7 Hz, 2H), 1.41 (s,18H), 1.17 (s, 9H); **^{13}C NMR (151 MHz, $CDCl_3$)** δ 173.34, 161.31, 159.68, 152.78, 136.06, 130.51, 129.98, 129.95, 129.66, 129.56, 128.00, 127.95, 125.23, 123.90, 123.88, 115.59, 115.44, 81.00, 58.56, 50.63, 34.37, 30.31, 27.53; **HRMS(ESI)**: calcd. for $C_{27}H_{39}FNO_3(M+H)^+$: 444.2908, found: 444.2909.

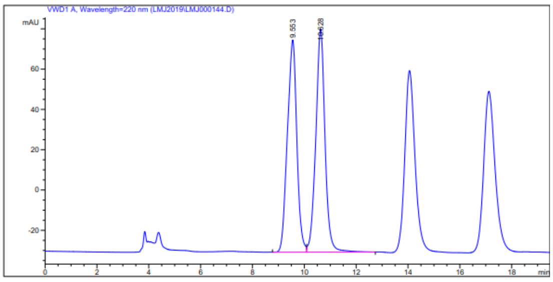

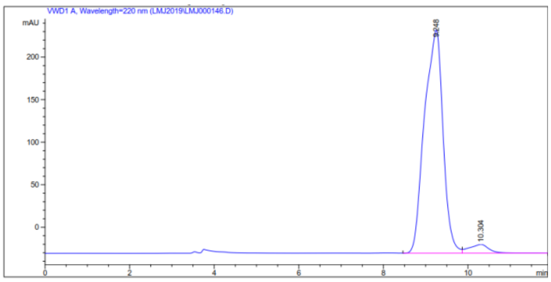

Peak #	RetTime [min]	Type	Width [min]	Area [mAU*s]	Height [mAU]	Area %
1	9.553	BV	0.3707	2661.43579	105.27404	49.3203
2	10.628	VB	0.3641	2734.79468	110.68400	50.6797

Peak #	RetTime [min]	Type	Width [min]	Area [mAU*s]	Height [mAU]	Area %
1	9.248	VV	0.4192	7978.59717	262.19858	95.9664
2	10.304	VBA	0.4493	335.34946	10.31478	4.0336

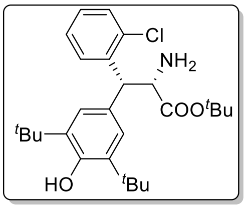

***tert*-butyl (2*S*,3*R*)-2-amino-3-(2-chlorophenyl)-3-(3,5-di-*tert*-butyl-4-hydroxyphenyl)propanoate (*syn*-5d):** white soild (32.4 mg, 71%); R_f = 0.55 (petroleum ether/EtOAc = 4:1); the enantiomeric excess was determined to be 93% by HPLC analysis on Daicel Chirapak IA-H column (hexane/isopropanol = 98/2, flow rate 1.0 mL/min, T = 30°C), UV 220 nm, t_R(major) 7.82 min, t_R(minor) 8.62 min); $[a]_D^{25}$ = +59.8 (c = 0.26, $CHCl_3$); **^{1}H NMR (600 MHz, $CDCl_3$)** δ 7.31 – 7.25 (m, 2H), 7.09 (s,2H), 6.85 (d, *J* = 8.6 Hz, 2H), 5.03 (s, 1H), 3.94 (dd, *J* = 71.1, 9.7 Hz, 2H), 3.77 (s, 3H), 1.40 (s, 18H), 1.15 (s, 9H); **^{13}C NMR (151 MHz, $CDCl_3$)** δ 173.95, 158.38, 152.45, 135.56, 134.09, 132.39, 129.35, 124.86, 114.15, 80.66, 60.01, 57.08, 55.23, 34.28, 30.29, 27.65; **HRMS(ESI)**: calcd. for $C_{27}H_{39}ClNO_3(M+H)^+$: 460.2613, found: 460.2612.

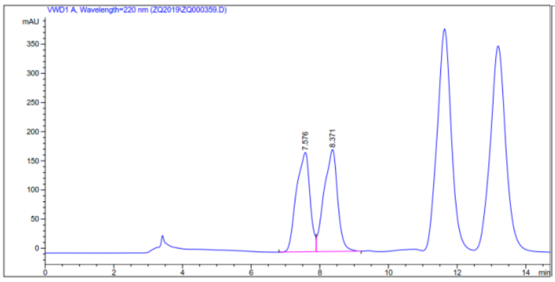

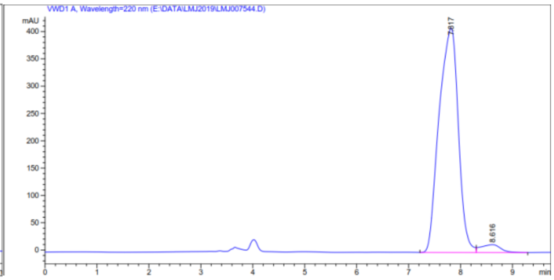

Peak #	RetTime [min]	Type	Width [min]	Area [mAU*s]	Height [mAU]	Area %
1	7.576	BV	0.3779	4664.19385	170.27283	49.2940
2	8.371	VB	0.3786	4797.79883	174.78056	50.7060

Peak #	RetTime [min]	Type	Width [min]	Area [mAU*s]	Height [mAU]	Area %
1	7.817	BV	0.4315	1.05776e4	410.79025	96.5124
2	8.616	VB	0.3767	382.23514	14.39984	3.4876

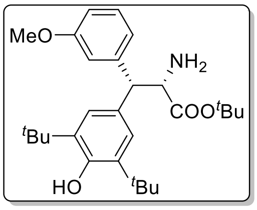

***tert*-butyl (2*S*,3*R*)-2-amino-3-(3,5-di-*tert*-butyl-4-hydroxyphenyl)-3-(3-methoxyphenyl)propanoate (*syn*-5e):** white soild (23.5 mg, 52%); m.p. = 112-113 ^{o}C; R_f = 0.31 (petroleum ether/EtOAc = 4:1); the enantiomeric excess was determined to be 82% by HPLC analysis on Daicel Chirapak IA-H column (hexane/isopropanol = 98/2, flow rate 1.0 mL/min, T = 30°C), UV 220 nm, t_R(major) 11.44 min, t_R(minor) 12.88 min; $[a]_D^{25}$ = +25.4 (c = 0.26, $CHCl_3$); **^{1}H NMR (400 MHz, $CDCl_3$)** δ 7.18 (t, *J* = 7.9 Hz, 1H), 7.13 (s, 2H), 6.94 (d, *J* = 7.7 Hz, 1H), 6.89 (s, 1H), 6.72 (dd, *J* = 8.1, 2.3 Hz, 1H), 5.09 (s, 1H), 3.98 (dd, *J* = 37.4, 9.4 Hz,

2H), 3.78 (s, 3H), 1.41 (s, 18H), 1.19 (s, 9H); **^{13}C NMR (101 MHz, $CDCl_3$)** δ 173.60, 159.44, 152.72, 143.78, 135.96, 131.22, 129.20, 125.01, 120.84, 114.38, 111.81, 80.98, 9.76, 57.65, 55.15, 34.37, 30.32, 27.63; **HRMS(ESI)**: calcd. for $C_{28}H_{42}NO_4(M+H)^+$: 456.3108, found: 456.3107.

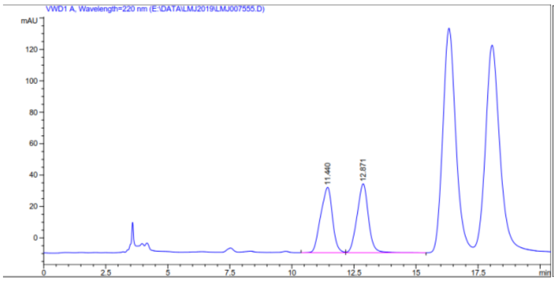

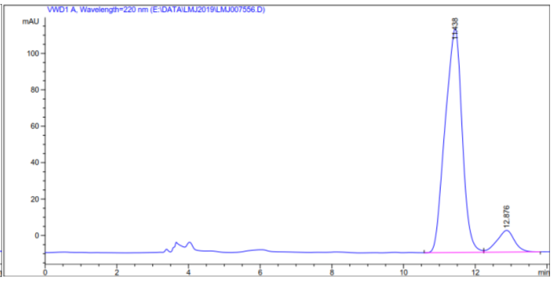

Peak #	RetTime [min]	Type	Width [min]	Area [mAU*s]	Height [mAU]	Area %
1	11.440	BV	0.5441	1362.75635	41.56360	49.5192
2	12.871	VB	0.4714	1389.22144	43.86101	50.4808

Peak #	RetTime [min]	Type	Width [min]	Area [mAU*s]	Height [mAU]	Area %
1	11.438	BV	0.5438	4038.20874	122.81121	90.9163
2	12.876	VB	0.4963	403.46655	11.93471	9.0837

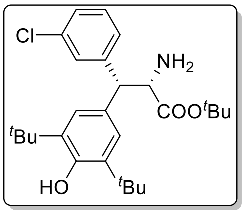

***tert*-butyl (2*S*,3*R*)-2-amino-3-(3-chlorophenyl)-3-(3,5-di-*tert*-butyl-4-hydroxyphenyl)propanoate (*syn*-5f):** white soild (38.3 mg, 83%); m.p.= 184-185 °C; R_f = 0.39 (petroleum ether/EtOAc = 4:1); the enantiomeric excess was determined to be 88% by HPLC analysis on Daicel Chirapak IA-H column (hexane/isopropanol = 98/2, flow rate 1.0 mL/min, T = 30°C), UV 220 nm, t_R(major) 11.16 min, t_R(minor) 13.47 min; $[a]_D^{25}$ = +30.4 (c = 0.38, $CHCl_3$); **^{1}H NMR (600 MHz, $CDCl_3$)** δ 7.34 (s, 1H), 7.19 (ddd, *J* = 25.0, 16.9, 7.7 Hz, 3H), 7.09 (s, 2H), 5.12 (s, 1H), 3.98 (dd, *J* = 30.0, 9.2 Hz, 2H), 1.41 (s, 18H), 1.21 (s, 9H); **^{13}C NMR (151 MHz, $CDCl_3$)** δ 173.29, 152.89, 144.41, 136.22, 133.99, 130.57, 129.51, 128.75, 126.62, 126.59, 125.02, 81.29, 59.50, 56.97, 34.39, 30.31, 27.67; **HRMS(ESI)**: calcd. for $C_{27}H_{39}ClNO_3(M+H)^+$: 460.2613, found: 460.2614.

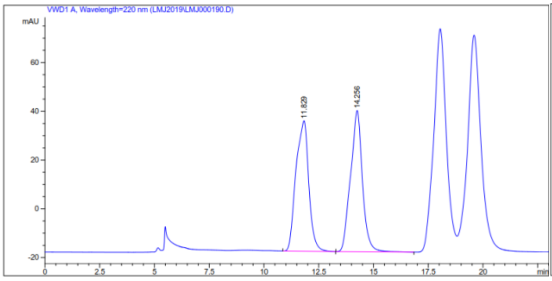

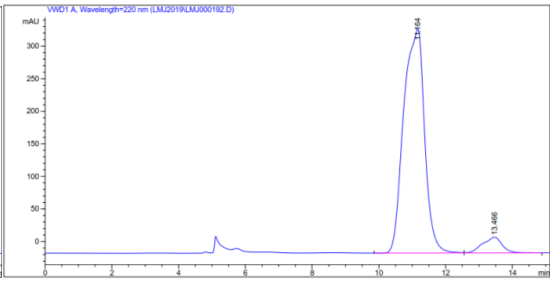

Peak #	RetTime [min]	Type	Width [min]	Area [mAU*s]	Height [mAU]	Area %
1	11.829	BB	0.5515	2175.71240	53.52769	49.8746
2	14.256	BB	0.5351	2186.65186	57.93169	50.1254

Peak #	RetTime [min]	Type	Width [min]	Area [mAU*s]	Height [mAU]	Area %
1	11.164	BV	0.5764	1.49374e4	344.13376	93.7930
2	13.466	VB	0.5613	988.51764	24.33915	6.2070

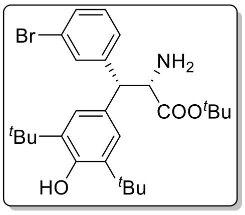

***tert*-butyl (2*S*,3*R*)-2-amino-3-(3-bromophenyl)-3-(3,5-di-*tert*-butyl-4-hydroxyphenyl)propanoate (*syn*-5g):** white soild (34.5 mg, 68%); R_f = 0.45 (petroleum ether/EtOAc = 4:1); the enantiomeric excess was determined to be 90% by HPLC analysis on Daicel Chirapak IA-H

column (hexane/isopropanol = 98/2, flow rate 1.0 mL/min, T = 30°C), UV 220 nm, t_R(major) 8.19 min, t_R(minor) 9.89 min; $[a]_D^{25}$ = +38.1 (c = 0.26, $CHCl_3$); **^{1}H NMR (400 MHz, $CDCl_3$)** δ 7.49 (s, 1H), 7.35 – 7.27 (m, 2H), 7.14 (t, *J* = 7.8 Hz, 1H), 7.09 (s, 2H), 5.13 (s, 1H), 3.96 (dd, *J* = 27.2, 9.3 Hz, 2H), 1.41 (s, 18H), 1.21 (s, 9H); **^{13}C NMR (101 MHz, $CDCl_3$)** δ 173.69, 152.73, 139.80, 136.03, 133.42, 132.26, 131.19, 127.79, 127.76, 127.48, 126.89, 126.84, 125.90, 125.46, 125.14, 81.05, 59.61, 57.59, 34.38, 30.33, 29.71, 27.58; **HRMS(ESI)**: calcd. for $C_{27}H_{39}BrNO_3(M+H)^+$: 504.2108, found: 504.2104.

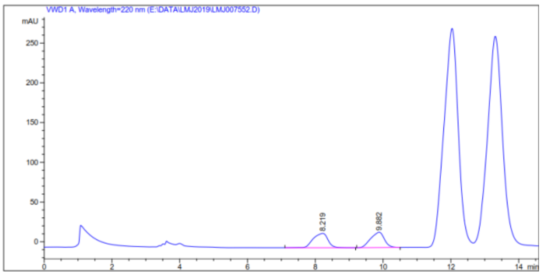

Peak #	RetTime [min]	Type	Width [min]	Area [mAU*s]	Height [mAU]	Area %
1	8.219	BB	0.5048	546.78033	18.04991	50.4148
2	9.882	BB	0.4676	537.78357	19.22228	49.5852

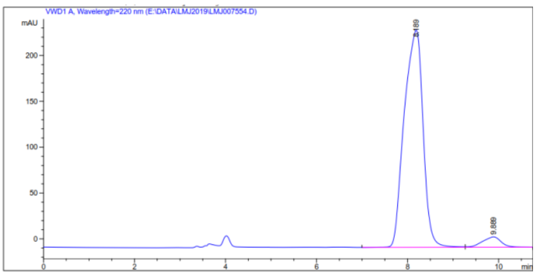

Peak #	RetTime [min]	Type	Width [min]	Area [mAU*s]	Height [mAU]	Area %
1	8.189	BV	0.4834	6801.81787	236.94421	95.4658
2	9.889	VBA	0.4670	323.05463	11.31836	4.5342

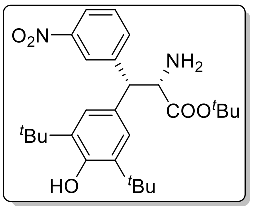

***tert*-butyl (2*S*,3*R*)-2-amino-3-(3,5-di-*tert*-butyl-4-hydroxyphenyl)-3-(3-nitrophenyl)propanoate (*syn*-5h):** white soild (23.5 mg, 50%); m.p. = 126-127 ^{o}C; R_f = 0.28 (petroleum ether/EtOAc = 4:1); the enantiomeric excess was determined to be 73% by HPLC analysis on Daicel Chirapak AD-H column (hexane/isopropanol = 95/5, flow rate 1.0 mL/min, T = 30°C), UV 220 nm, t_R(major) 13.72 min, t_R(minor) 15.14 min; $[a]_D^{25}$ = +7.5 (c = 0.32, $CHCl_3$); **^{1}H NMR (600 MHz, $CDCl_3$)** δ 8.28 (s, 1H), 8.06 (dd, *J* = 8.2, 1.3 Hz, 1H), 7.71 (d, *J* = 7.7 Hz, 1H), 7.45 (t, *J* = 8.0 Hz, 1H), 7.10 (s, 2H), 5.15 (s, 1H), 4.12 (dd, *J* = 63.7, 8.6 Hz,2H), 1.41 (s,18H), 1.22 (s, 9H); **^{13}C NMR (151 MHz, $CDCl_3$)** δ 173.07, 153.11, 148.22, 144.77, 136.42, 134.76, 129.83, 129.10, 125.12, 123.45, 121.50, 81.53, 59.33, 56.19, 34.41, 30.28, 27.73; **HRMS(ESI)**: calcd. for $C_{27}H_{39}N_2O_5(M+H)^+$: 471.2853, found: 471.2852.

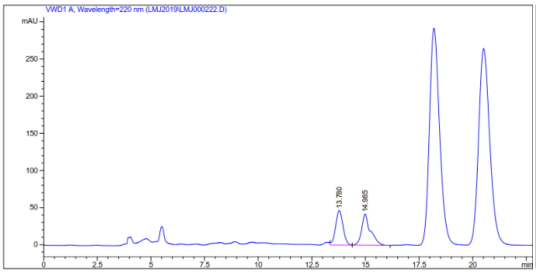

Peak #	RetTime [min]	Type	Width [min]	Area [mAU*s]	Height [mAU]	Area %
1	13.780	VB	0.3693	1110.50671	46.44325	48.3658
2	14.985	BB	0.3972	1185.55017	41.90076	51.6342

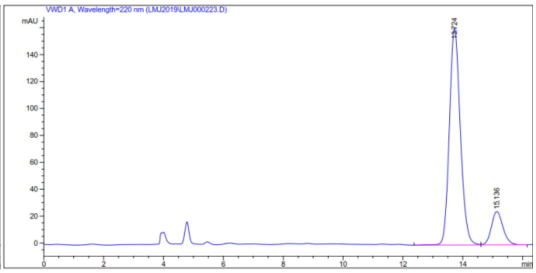

Peak #	RetTime [min]	Type	Width [min]	Area [mAU*s]	Height [mAU]	Area %
1	13.724	BV	0.3894	4087.69653	161.05357	86.0070
2	15.136	VB	0.4119	665.05035	24.80947	13.9930

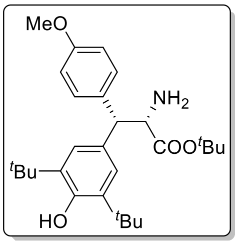

***tert*-butyl (2*S*,3*S*)-2-amino-3-(3,5-di-*tert*-butyl-4-hydroxyphenyl)-3-(4-methoxyphenyl)propanoate (*syn*-5i):** white soild (29.6 mg, 65%); R_f = 0.4 (petroleum ether/EtOAc = 4:1); the enantiomeric excess was determined to be 86% by HPLC analysis on Daicel Chirapak IA-H column (hexane/isopropanol = 98/2, flow rate 1.0 mL/min, T = 30°C), UV 220 nm, t_R(major) 11.13 min, t_R(minor) 13.51 min; $[a]_D^{25}$ = +32.5 (c = 0.40, $CHCl_3$); **^{1}H NMR (400 MHz, $CDCl_3$)** δ 7.24 (s, 2H), 7.10 (s, 2H), 6.81 (d, *J* = 8.7 Hz, 2H), 5.08 (s, 1H), 3.96 (dd, *J* = 31.0, 9.5 Hz, 2H), 3.76 (s, 3H), 1.41 (s,18H), 1.19 (s9H); **^{13}C NMR (101 MHz, $CDCl_3$)** δ 173.73, 158.23, 152.61, 135.96, 134.50, 131.73, 129.39, 124.89, 113.67, 80.94, , 59.95, 56.80, 55.29, 34.37, 30.32, 27.67; **HRMS(ESI)**: calcd. for $C_{28}H_{42}NO_4(M+H)^+$: 456.3108, found: 456.3106.

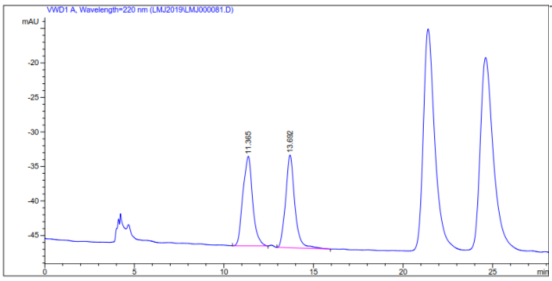

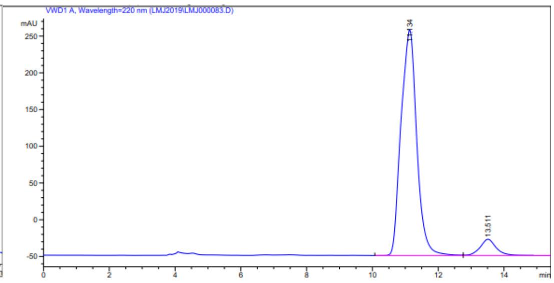

Peak #	RetTime [min]	Type	Width [min]	Area [mAU*s]	Height [mAU]	Area %
1	11.365	BB	0.5378	488.33536	12.98709	50.4019
2	13.692	BB	0.5191	480.54675	13.44356	49.5981

Peak #	RetTime [min]	Type	Width [min]	Area [mAU*s]	Height [mAU]	Area %
1	11.134	BV	0.5409	1.01595e4	306.78946	93.1799
2	13.511	VBA	0.4907	743.60504	22.07020	6.8201

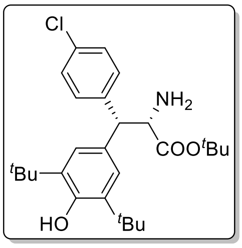

***tert*-butyl (2*S*,3*S*)-2-amino-3-(4-chlorophenyl)-3-(3,5-di-*tert*-butyl-4-hydroxyphenyl)propanoate (*syn*-5j):** white soild (31.8 mg, 69%); R_f = 0.29 (petroleum ether/EtOAc = 4:1); the enantiomeric excess was determined to be 90% by HPLC analysis on Daicel Chirapak IA-H column (hexane/isopropanol = 98/2, flow rate 1.0 mL/min, T = 30°C), UV 220 nm, t_R(major) 7.51 min, t_R(minor) 9.80 min; $[a]_D^{25}$ = +19.2 (c = 0.50, $CHCl_3$); **^{1}H NMR (400 MHz, $CDCl_3$)** δ 7.32 – 7.20 (m, 5H), 7.08 (s, 2H), 5.11 (s, 1H), 3.99 (q, *J* = 9.3 Hz, 2H), 1.41 (s, 18H), 1.21 (s, 9H); **^{13}C NMR (101 MHz, $CDCl_3$)** δ 173.44, 152.81, 140.92, 136.12, 132.21, 130.84, 129.80, 128.34, 124.92, 81.24, 59.56, 56.63, 34.38, 30.29, 27.68; **HRMS(ESI)**: calcd. for $C_{27}H_{39}ClNO_3(M+H)^+$: 460.2613, found: 460.2611.

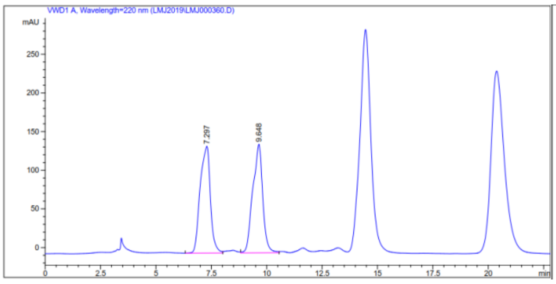

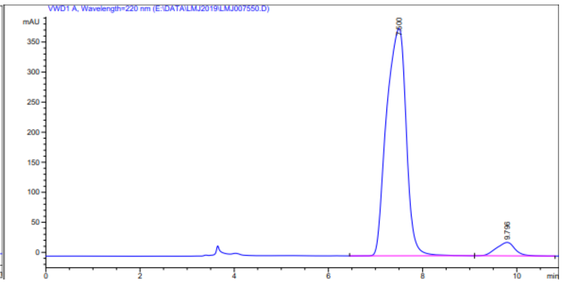

Peak #	RetTime [min]	Type	Width [min]	Area [mAU*s]	Height [mAU]	Area %
1	7.297	BV	0.5256	4430.11084	138.20865	50.1937
2	9.648	VV	0.4318	4395.91602	140.62312	49.8063

Peak #	RetTime [min]	Type	Width [min]	Area [mAU*s]	Height [mAU]	Area %
1	7.500	BB	0.4854	1.09258e4	378.35352	94.6782
2	9.796	BBA	0.3947	614.12970	22.46407	5.3218

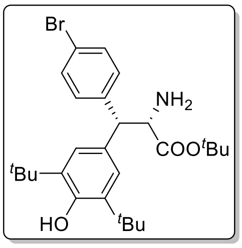

***tert*-butyl (2*S*,3*S*)-2-amino-3-(4-bromophenyl)-3-(3,5-di-*tert*-butyl-4-hydroxyphenyl)propanoate (*syn*-5k):** white soild (32.3 mg, 64%); R_f = 0.23 (petroleum ether/EtOAc = 4:1); the enantiomeric excess was determined to be 90% by HPLC analysis on Daicel Chirapak IA-H column (hexane/isopropanol = 98/2, flow rate 1.0 mL/min, T = 30°C), UV 220 nm, t_R(major) 5.62 min, t_R(minor) 7.88 min; $[a]_D^{25}$ = +15.3 (c = 0.6, $CHCl_3$); **^{1}H NMR (400 MHz, $CDCl_3$)** δ 7.39 (d, *J* = 8.4 Hz, 2H), 7.22 (d, *J* = 8.4 Hz, 2H), 7.07 (s, 2H), 5.12 (s, 1H), 3.98 (dd, *J* = 19.8, 9.2 Hz, 2H), 1.41 (s, 18H), 1.21 (s, 9H); **^{13}C NMR (101 MHz, $CDCl_3$)** δ 173.37, 152.84, 141.42, 136.15, 130.73, 130.19, 124.92, 120.29, 81.30, 59.46, 56.65, 34.39, 30.30, 27.68; **HRMS(ESI)**: calcd. for $C_{27}H_{39}BrNO_3(M+H)^+$: 504.2108, found: 504.2102.

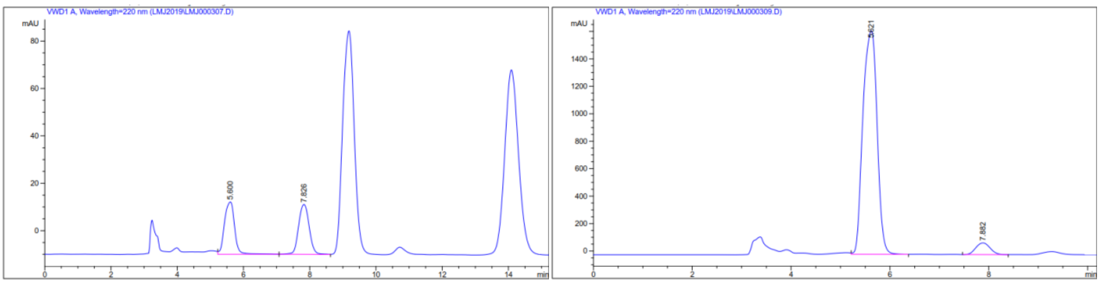

Peak #	RetTime [min]	Type	Width [min]	Area [mAU*s]	Height [mAU]	Area %
1	5.600	VB	0.3417	470.67319	22.10636	50.1263
2	7.826	BV	0.3527	468.30042	21.05824	49.8737

Peak #	RetTime [min]	Type	Width [min]	Area [mAU*s]	Height [mAU]	Area %
1	5.621	VB	0.3258	3.24273e4	1627.33435	94.9812
2	7.882	VV	0.3228	1713.46204	84.95148	5.0188

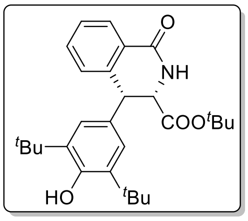

***tert*-butyl (3*S*,4*R*)-4-(3,5-di-*tert*-butyl-4-hydroxyphenyl)-1-oxo-1,2,3,4-tetrahydroisoquinoline-3-carboxylate (*syn*-5l):** white soild (24.7 mg, 57%); R_f = 0.38 (petroleum ether/EtOAc = 4:1); the enantiomeric excess was determined to be 92% by HPLC analysis on Daicel Chirapak IA-H column (hexane/isopropanol = 90/10, flow rate 1.0 mL/min, T = 30°C), UV 220 nm, t_R(major) 9.78 min, t_R(minor) 11.53 min; $[a]_D^{25}$ = -516.6 (c = 0.39, $CHCl_3$); **^{1}H NMR (600 MHz, $CDCl_3$)** δ 8.13 (d, *J* = 7.6 Hz, 1H), 7.44 (dd, *J* = 7.4, 6.6 Hz, 1H), 7.35 (t, *J* = 7.4 Hz, 1H), 7.30 – 7.25 (m, 1H), 7.09 (s, 2H), 6.46 (s, 1H), 5.09 (s, 1H), 4.74 (d, *J* = 5.2 Hz, 1H), 4.39 (d, *J* = 5.1 Hz, 1H), 1.36 (s, 18H), 1.23 (s, 9H); **^{13}C NMR (151 MHz, $CDCl_3$)** δ 167.74, 164.94, 153.24, 141.16, 135.80, 132.83, 129.45, 128.21, 128.01, 127.59, 127.40, 124.87, 82.87, 57.55, 46.36, 34.23, 30.16, 27.68; **HRMS(ESI)**: calcd. for C28H38NO4 $(M+H)^+$: 452.2795, found: 452.2791.

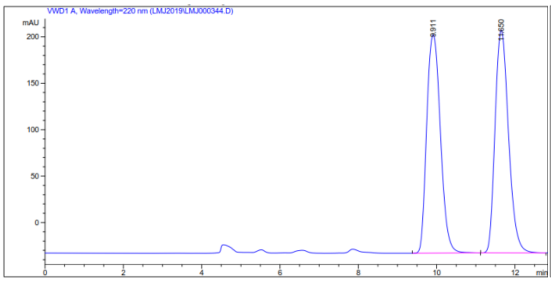

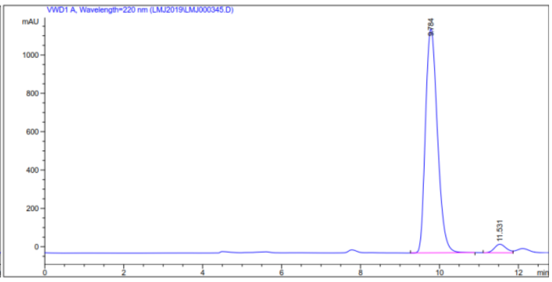

Peak #	RetTime [min]	Type	Width [min]	Area [mAU*s]	Height [mAU]	Area %
1	9.911	BB	0.3898	5710.45898	235.10269	49.9303
2	11.650	BB	0.3747	5726.40332	239.91267	50.0697

Peak #	RetTime [min]	Type	Width [min]	Area [mAU*s]	Height [mAU]	Area %
1	9.784	BB	0.3316	2.45675e4	1167.19727	96.3906
2	11.531	BV	0.3160	919.95490	44.68387	3.6094

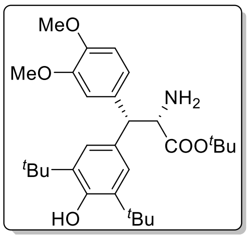

***tert*-butyl (2*S*,3*R*)-2-amino-3-(3,5-di-*tert*-butyl-4-hydroxyphenyl)-3-(3,4-dimethoxyphenyl)propanoate (*syn*-5m):** light yellow gum (26.5 mg, 55%); R_f = 0.35 (petroleum ether/EtOAc = 4:1); the enantiomeric excess was determined to be 59% by HPLC analysis on Daicel Chirapak AD-H column (hexane/isopropanol = 90/10 flow rate 1.0 mL/min, T = 30°C), UV 220 nm, t_R(major) 7.61 min, t_R(minor) 9.09 min; $[a]_D^{25}$ = +31.5 (c = 0.34, $CHCl_3$); **^{1}H NMR (600 MHz, $CDCl_3$)** δ 7.13 (s, 2H), 6.92 – 6.85 (m, 2H), 6.78 (d, *J* = 8.2 Hz, 1H), 5.08 (s, 1H), 3.96 (dd, *J* = 36.2, 9.2 Hz, 2H), 3.84 (d, *J* = 14.9 Hz, 18H), 1.41 (s, 18H), 1.21 (s, 9H); **^{13}C NMR (151 MHz, $CDCl_3$)** δ 173.69, 152.65, 148.68, 147.75, 135.99, 134.99, 131.56, 124.94, 120.61, 112.02, 111.23, 80.91, 60.05, 56.98, 55.97, 55.80, 34.37, 30.34, 27.71; **HRMS(ESI)**: calcd. for $C_{29}H_{44}NO_5(M+H)^+$: 486.3214, found: 486.3212.

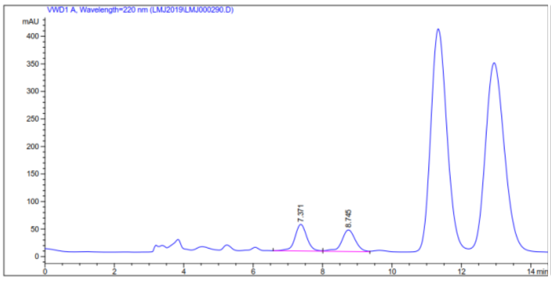

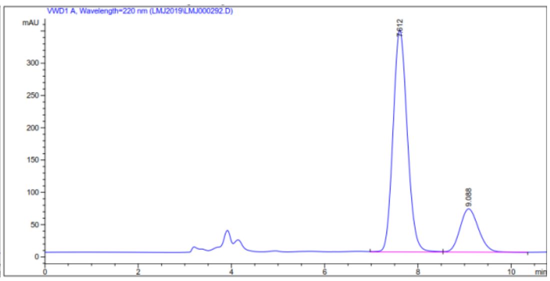

Peak #	RetTime [min]	Type	Width [min]	Area [mAU*s]	Height [mAU]	Area %
1	7.371	BV	0.3655	1152.10059	48.34307	52.1536
2	8.745	VV	0.4198	1056.95142	39.16884	47.8464

Peak #	RetTime [min]	Type	Width [min]	Area [mAU*s]	Height [mAU]	Area %
1	7.612	VV	0.3290	7289.97998	343.80743	79.7478
2	9.088	VB	0.4299	1851.31079	67.07123	20.2522

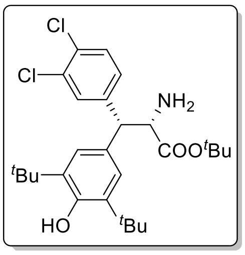

***tert*-butyl (2*S*,3*R*)-2-amino-3-(3,5-di-*tert*-butyl-4-hydroxyphenyl)-3-(3,4-dichlorophenyl)propanoate (*syn*-5n):** white soild (36.7 mg, 74%); R_f=0.38 (petroleum ether/EtOAc=4:1); the enantiomeric excess was determined to be 88% by HPLC analysis on Daicel Chirapak IA-H column (hexane/isopropanol = 98/2, flow rate 1.0 mL/min, T = 30°C), UV 220 nm, t_R(major) 9.90 min, t_R(minor) 12.43 min; $[a]_D^{25}$ = +25.7 (c = 0.60, $CHCl_3$); **^{1}H NMR (600 MHz, $CDCl_3$)** δ 7.45 (d, *J* = 1.5 Hz, 1H), 7.34 (d, *J* = 8.3 Hz, 1H), 7.19 (dd, *J* = 8.3, 1.6 Hz,

1H), 7.06 (s,2H), 5.14 (s, 1H), 3.98 (s, 2H), 1.41 (s, 18H), 1.24 (s, 9H); **^{13}C NMR (151 MHz, $CDCl_3$)** δ 173.14, 153.00, 142.80, 136.32, 132.11, 130.54, 130.39, 130.12, 127.86, 124.99, 81.49, 59.33, 55.97, 34.40, 30.30, 27.74; **HRMS(ESI)**: calcd. for $C_{27}H_{38}Cl_2NO_3(M+H)^+$: 494.2223, found: 494.2222.

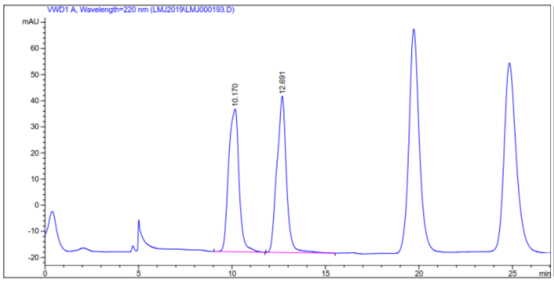

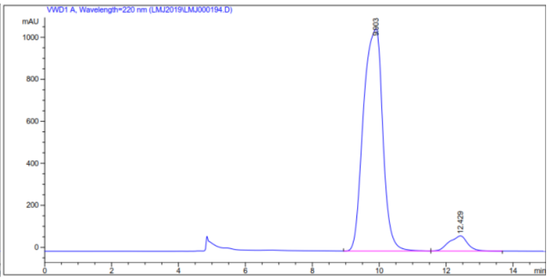

Peak #	RetTime [min]	Type	Width [min]	Area [mAU*s]	Height [mAU]	Area %
1	10.170	BB	0.6211	2113.12817	54.71460	50.0026
2	12.691	BB	0.4932	2112.90649	59.89526	49.9974

Peak #	RetTime [min]	Type	Width [min]	Area [mAU*s]	Height [mAU]	Area %
1	9.903	BV	0.6525	4.23647e4	1057.53186	93.9063
2	12.429	VB	0.5212	2749.09546	72.48861	6.0937

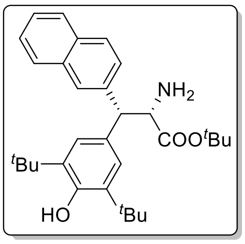

***tert*-butyl (2*S*,3*S*)-2-amino-3-(3,5-di-*tert*-butyl-4-hydroxyphenyl)-3-(naphthalen-2-yl)propanoate (*syn*-5o):** white soild (34.3mg, 72%); R_f = 0.51 (petroleum ether/EtOAc = 4:1); the enantiomeric excess was determined to be 84% by HPLC analysis on Daicel Chirapak IA-H column (hexane/isopropanol = 98/2, flow rate 1.0 mL/min, T = 30°C), UV 220 nm, t_R(major) 9.85 min, t_R(minor) 12.01 min; $[a]_D^{25}$ = +20.4 (c = 0.37, $CHCl_3$); **^{1}H NMR (400 MHz, $CDCl_3$)** δ 7.80-7.70(m, 4H), 7.51 – 7.36 (m, 3H), 7.18 (s, 2H), 5.09 (s, 1H), 4.17 (s,2H), 1.41 (s, 18H), 1.08 (s, 9H); **^{13}C NMR (101 MHz, $CDCl_3$)** δ 173.69, 152.73, 139.80, 136.03, 133.42, 132.26, 131.19, 127.79, 127.76, 127.48, 126.89, 126.84, 125.90, 125.46, 125.14, 81.05, 59.61, 57.59, 34.38, 30.33, 27.58; **HRMS(ESI)**: calcd. for $C_{31}H_{42}NO_3(M+H)^+$: 476.3159, found: 476.3158.

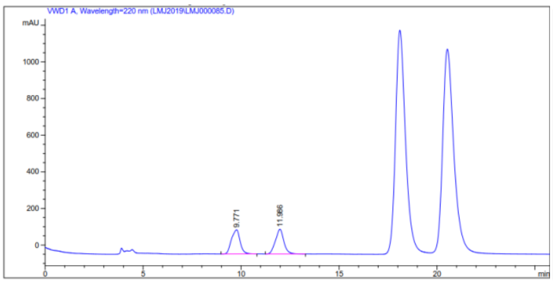

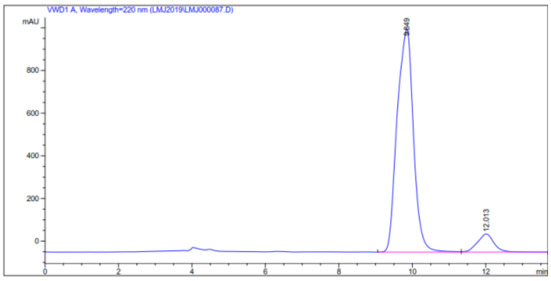

Peak #	RetTime [min]	Type	Width [min]	Area [mAU*s]	Height [mAU]	Area %
1	9.771	BB	0.4984	4027.98901	132.17239	49.9465
2	11.986	BB	0.4369	4036.62256	135.33258	50.0535

Peak #	RetTime [min]	Type	Width [min]	Area [mAU*s]	Height [mAU]	Area %
1	9.849	BV	0.5019	3.22032e4	1046.32324	92.4100
2	12.013	VBA	0.4530	2644.96973	85.09753	7.5900

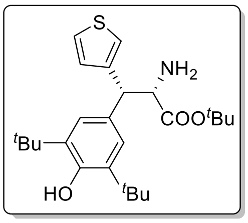

***tert*-butyl (2*S*,3*R*)-2-amino-3-(3,5-di-*tert*-butyl-4-hydroxyphenyl)-3-(thiophen-3-yl)propanoate (*syn*-5p):** white soild (31.6 mg, 73%); m.p. = 127-128 °C; R_f = 0.23 (petroleum ether/EtOAc = 4:1); the enantiomeric excess was determined to be 88% by HPLC analysis on Daicel Chirapak IA-H column (hexane/isopropanol = 98/2, flow rate 1.0 mL/min, T = 30°C), UV 220 nm, t_R(major) 8.42 min, t_R(minor) 10.73 min; $[a]_D^{25}$ = +23.9 (c = 0.48, $CHCl_3$); **^{1}H NMR (600 MHz, $CDCl_3$)** δ 7.22 (dd, *J* = 4.7, 3.0 Hz, 1H), 7.12 (s, 1H), 7.08 (s, 2H), 7.06 (d, *J* = 4.9 Hz, 1H), 5.10 (s, 1H), 4.06 (dd, *J* = 126.3, 8.5 Hz, 2H), 1.41 (s, 18H), 1.26 (s, 9H); **^{13}C NMR (151 MHz, $CDCl_3$)** δ 173.51, 152.74, 142.86, 135.97, 130.83, 128.02, 125.18, 125.05, 121.49, 81.03, 60.13, 52.40, 34.36, 30.33, 27.75; **HRMS(ESI)**: calcd. for $C_{25}H_{38}NO_3S(M+H)^+$: 432.2567, found: 432.2567.

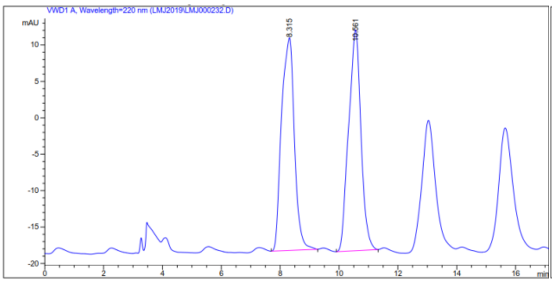

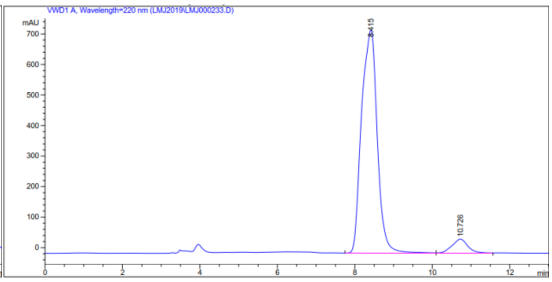

Peak #	RetTime [min]	Type	Width [min]	Area [mAU*s]	Height [mAU]	Area %
1	8.315	BB	0.4168	886.02478	29.19190	50.1360
2	10.561	BB	0.4180	881.21771	30.30588	49.8640

Peak #	RetTime [min]	Type	Width [min]	Area [mAU*s]	Height [mAU]	Area %
1	8.415	BV	0.4591	2.05414e4	730.84320	94.0692
2	10.726	VV	0.4111	1295.07263	45.86024	5.9308

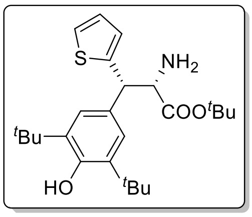

***tert*-butyl (2*S*,3*S*)-2-amino-3-(3,5-di-*tert*-butyl-4-hydroxyphenyl)-3-(thiophen-2-yl)propanoate (*syn*-5q):** white soild (33.6 mg, 78%); R_f = 0.46 (petroleum ether/EtOAc = 4:1); the enantiomeric excess wasdetermined to be 94% by HPLC analysis on Daicel Chirapak IA-H column (hexane/isopropanol = 98/2, flow rate 1.0 mL/min, T = 30°C), UV 220 nm, t_R(major) 8.49 min, t_R(minor) 9.53 min; $[a]_D^{25}$ = +74.2 (c = 0.48, $CHCl_3$); **^{1}H NMR (400 MHz, $CDCl_3$)** δ 7.15 (d, *J* = 4.2 Hz, 3H), 6.95 – 6.86 (m,2H), 5.12 (s, 1H), 4.14 (dd, *J* = 165.8, 8.2 Hz, 2H), 1.42 (s, 18H), 1.28 (s, 9H); **^{13}C NMR (101 MHz, $CDCl_3$)** δ 173.11, 152.96, 145.71, 135.94, 130.62, 126.26, 125.20, 125.17, 124.05, 81.21, 61.19, 52.25, 34.38, 30.31, 27.78; **HRMS(ESI)**: calcd. for $C_{25}H_{38}NO_3S(M+H)^+$: 432.2567, found: 432.2566.

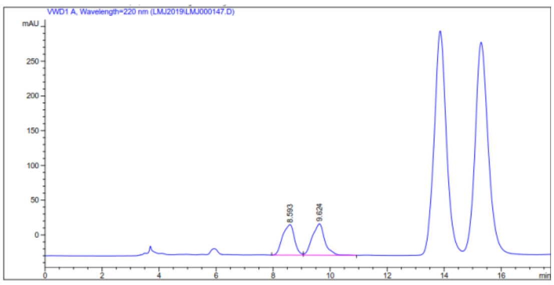

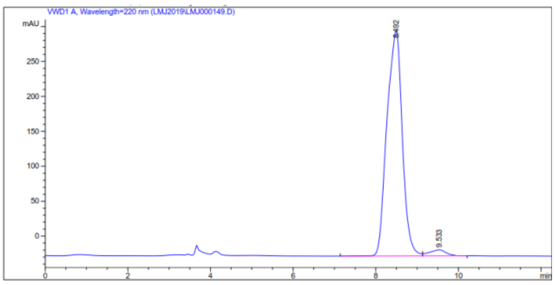

Peak #	RetTime [min]	Type	Width [min]	Area [mAU*s]	Height [mAU]	Area %
1	8.593	BV	0.4752	1261.90491	43.91479	48.0210
2	9.624	VB	0.4162	1365.91650	45.26609	51.9790

Peak #	RetTime [min]	Type	Width [min]	Area [mAU*s]	Height [mAU]	Area %
1	8.492	BV	0.4231	8292.38379	321.74127	97.2438
2	9.533	VB	0.3907	235.03587	8.51122	2.7562

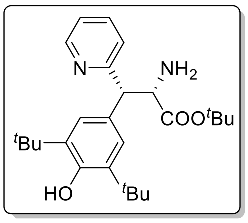

***tert*-butyl (2*S*,3*S*)-2-*amino*-3-(3,5-*di*-*tert*-butyl-4-hydroxyphenyl)-3-(pyridin-2-yl)propanoate (*syn*-5r):** white soild (25 mg, 73%); R_f = 0.26 (petroleum ether/EtOAc = 2:1);the enantiomeric excess was determined to be 86% by HPLC analysis after being converted to the corresponding N-Boc derivatives. HPLC: Daicel Chirapak IA-H column (hexane/isopropanol = 80/20, flow rate 1.0 mL/min, T = 30°C), UV 220 nm, t_R(major) 7.70 min, t_R(minor) 10.60 min; $[a]_D^{25}$ = -21.7 (c = 0.24, $CHCl_3$); **^{1}H NMR (600 MHz, $CDCl_3$)** δ 8.55 (d, *J* = 4.3 Hz, 1H), 7.60 (td, *J* = 7.7, 1.5 Hz, 1H), 7.37 (d, *J* = 7.9 Hz, 1H), 7.20 (s, 2H), 7.11 (dd, *J* = 6.8, 5.3 Hz, 1H), 5.11 (s, 1H), 3.31 – 3.23 (m, 2H), 1.45 (s, 9H), 1.40 (s, 18H); **^{13}C NMR (151 MHz, $CDCl_3$)** δ 171.61, 162.70, 153.11, 149.05, 136.47, 135.87, 132.52, 124.53, 121.82, 121.77, 81.03, 68.13, 49.91, 34.37, 30.33, 28.14; **HRMS(ESI)**: calcd. for $C_{26}H_{39}N_2O_3(M+H)^+$: 427.2955, found: 427.2954.

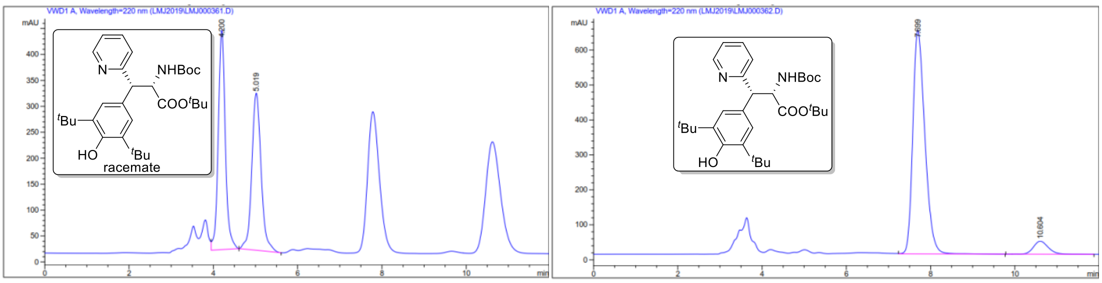

Peak #	RetTime [min]	Type	Width [min]	Area [mAU*s]	Height [mAU]	Area %
1	4.200	VB	0.1829	4939.02148	423.01434	51.4134
2	5.019	BB	0.2352	4667.46924	302.81317	48.5866

Peak #	RetTime [min]	Type	Width [min]	Area [mAU*s]	Height [mAU]	Area %
1	7.699	VB	0.3082	1.27314e4	639.16992	93.2795
2	10.604	BBA	0.3804	917.25964	37.27408	6.7205

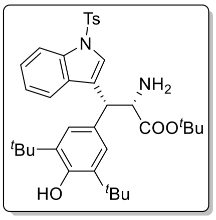

***tert*-butyl (2*S*,3*R*)-2-amino-3-(3,5-di-*tert*-butyl-4-hydroxyphenyl)-3-(1-tosyl-1H-indol-3-yl)propanoate (*syn*-5s):** light yellow oil (46 mg, 65%); R_f = 0.28 (petroleum ether/EtOAc = 2:1); the enantiomeric excess was determined to be 88% by HPLC analysis on Daicel Chirapak IA-H column (hexane/isopropanol = 90/10, flow rate 1.0 mL/min, T = 30°C), UV 220 nm, t_R(major) 10.05 min, t_R(minor) 12.11 min; $[a]_D^{25}$ = +34.1 (c = 0.45, $CHCl_3$);**^{1}H NMR (600 MHz, $CDCl_3$)** δ 7.96 (d, *J* = 8.3 Hz, 1H), 7.82 – 7.67 (m, 3H), 7.40 (d, *J* = 7.8 Hz, 1H), 7.30 – 7.22 (m, 3H), 7.23 – 7.13 (m, 3H), 7.05 (s, 2H), 5.06 (s, 1H), 4.15 (dd, *J* = 207.9, 7.5 Hz, 2H), 2.32 (s, 3H), 1.36 (s, 18H), 1.23 (s, 9H); **^{13}C NMR (151 MHz, $CDCl_3$)** δ 173.37, 152.86, 144.58, 135.81, 134.96, 134.66, 130.56, 129.82, 129.31, 126.74, 125.30, 124.57, 123.48, 122.92, 120.06, 113.53, 81.28, 59.45, 46.79, 34.28, 30.29, 27.77, 21.48; **HRMS(ESI)**: calcd. for $C_{36}H_{47}N_2O_5S(M+H)^+$: 619.3200, found: 619.3203.

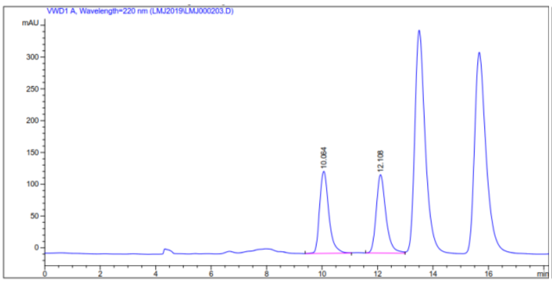

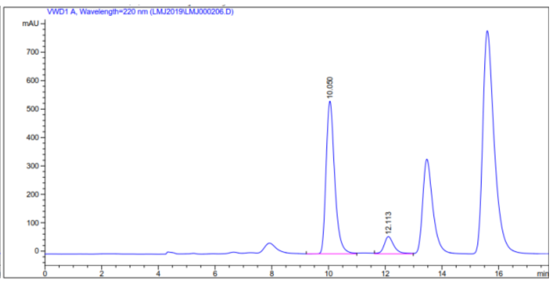

Peak #	RetTime [min]	Type	Width [min]	Area [mAU*s]	Height [mAU]	Area %
1	10.050	BV	0.3256	1.13628e4	536.93329	88.5187
2	12.113	VV	0.3615	1473.80481	60.80655	11.4813

Peak #	RetTime [min]	Type	Width [min]	Area [mAU*s]	Height [mAU]	Area %
1	10.064	BV	0.3582	2979.44629	129.09920	49.9552
2	12.108	BV	0.3672	2984.78467	123.21734	50.0448

6. General procedure for the catalytic asymmetric Mannich reaction.

1) General procedure for catalytic asymmetric synthesis of compounds *syn*-8.

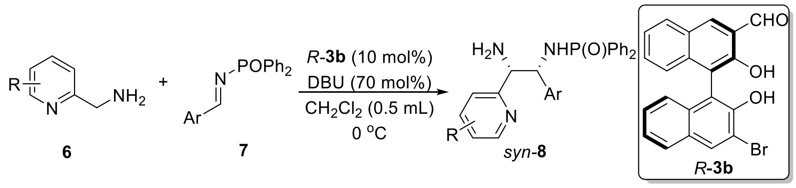

Imines **7** (0.13 mmol) and catalyst *R*-**3b** (0.01mmol) were placed in a dry Schlenk tube. Dry CH_2Cl_2 (0.5 mL) was added and the solution was stirred at 0 ℃ for 10 min. Then aminomethylpyridines **6** (0.1 mmol) and DBU (0.07 mmol) were added and the resulted mixture was effectively stirred at 0 ℃ and monitored by TLC. After the complete consumption of aminomethylpyridines **6**, the mixture was concentrated *in vacuo* and purified by flash chromatography on silica gel (CH_2Cl_2: MeOH: Et_3N = 30:1:0 to 30:1:0.15) to afford the compound *syn*-**8**.

2) General procedure for catalytic asymmetric synthesis of compounds *anti*-8.

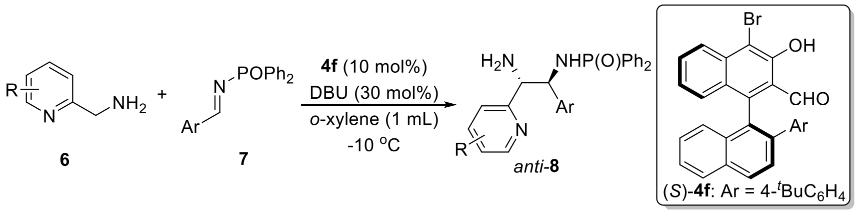

Imines **7** (0.13 mmol) and catalyst **4f** (0.01mmol) were placed in a dry Schlenk tube. Dry *o*-xylene (1 mL) was added and the solution was stirred at -10 ℃ for 10 min. Then aminomethylpyridines **6** (0.1 mmol), and DBU (0.01 mmol) were added and the resulted mixture was effectively stirred at -10 ℃ and monitored by TLC. After the complete consumption of aminomethylpyridines **6**, the mixture was concentrated *in vacuo* and purified by flash chromatography on silica gel (CH_2Cl_2: MeOH: Et_3N = 30:1:0 to 30:1:0.15) to afford the compound *anti*-**8**.

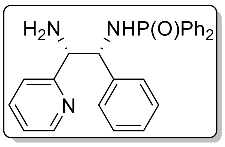

N-((1*R*,2*R*)-2-amino-1-phenyl-2-(pyridin-2-yl)ethyl)-P,P-diphenylphosphinic amide (*syn*-8a): white solid (34.3 mg, 83%); m.p. = 201-202 ^{o}C; R_f = 0.25 (CH_2Cl_2/MeOH = 25:1); the enantiomeric excess was determined to be 90% by HPLC analysis after being converted to the corresponding *N*-Boc derivatives. **HPLC**: Daicel Chirapak IA-H column (hexane/isopropanol = 70/30, flow rate 1.0 mL/min, T = 30°C), UV 210 nm, t_R(major) 13.94 min, t_R(minor) 12.64 min; $[\alpha]_D^{25}$ = +185.5 (c = 0.22, MeOH); **^{1}H NMR (600 MHz, $CDCl_3$)** δ 8.43 (d, *J* = 4.5 Hz, 1H), 7.85 (dd, *J* = 11.9, 7.3 Hz, 2H), 7.69 (dd, *J* = 12.0, 7.8 Hz, 2H), 7.52 – 7.41 (m, 4H), 7.37 (t, *J* = 7.4 Hz, 1H), 7.24 (td, *J* = 7.5, 2.7 Hz, 2H), 7.14 – 7.08 (m, 4H), 6.91 – 6.86 (m, 2H), 6.85 (d, *J* = 7.8 Hz, 1H), 5.44 – 5.37 (m, 1H), 4.58 (td, *J* = 11.1,

4.4 Hz, 1H), 4.35 (d, *J* = 3.8 Hz, 1H); **^{13}C NMR (151 MHz, $CDCl_3$)** δ 160.56, 148.71, 140.61, 140.59, 136.31, 133.76, 132.92, 132.59, 132.53, 132.42, 131.75, 131.69, 131.58, 128.51, 128.43, 128.21, 128.13, 127.88, 126.97, 126.89, 123.29, 122.39, 61.37, 60.73; **HRMS (ESI)**: calcd. for $C_{25}H_{25}N_3OP(M+H)^+$: 414.1730, found: 414.1724.

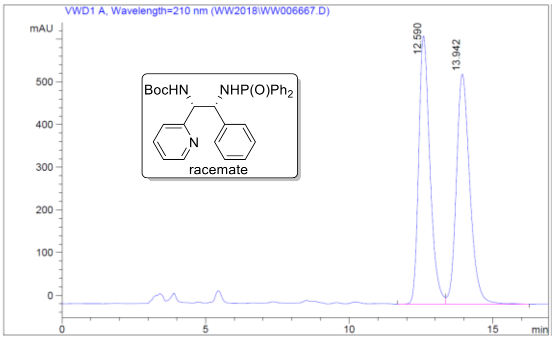

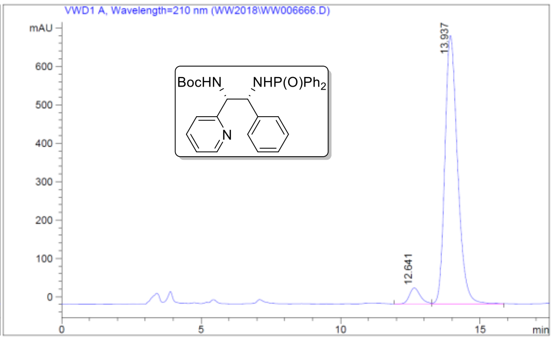

Meas.RT min	Area mAU*sec	Height mAU	Area %	Width min	Symm.
12.59	17128.0	628.1	49.3	0.417	0.708
13.94	17581.2	538.7	50.7	0.503	0.742

Meas.RT min	Area mAU*sec	Height mAU	Area %	Width min	Symm.
12.64	1136.2	41.8	4.9	0.410	0.767
13.94	22071.8	698.4	95.1	0.485	0.722

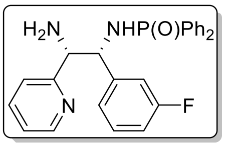

N-((1*R*,2*R*)-2-amino-1-(3-fluorophenyl)-2-(pyridin-2-yl)ethyl)-P,P-diphenylphosphinic amide (*syn*-8b): white solid (27.4 mg, 64%); m.p. = 145-146 ^{o}C; R_f = 0.28 (CH_2Cl_2/MeOH = 25:1); the enantiomeric excess was determined to be 83% by HPLC analysis after being converted to the corresponding *N*-Boc derivatives. **HPLC**: Daicel Chirapak IA-H column (hexane/isopropanol = 70/30, flow rate 1.0 mL/min, T = 30°C), UV 210 nm, t_R(major) 10.22 min, t_R(minor) 8.10 min; $[\alpha]_D^{25}$ = +216.0 (c = 0.20, MeOH); **^{1}H NMR (600 MHz, $CDCl_3$)** δ 8.43 (d, *J* = 4.3 Hz,1H), 7.93 – 7.82 (m, 2H), 7.75 – 7.66 (m, 2H), 7.55 – 7.43 (m, 4H), 7.39 (t, *J* = 6.7 Hz, 1H), 7.29 – 7.24 (m, 2H), 7.15 – 7.11 (m, 1H), 7.08 (dd, *J* = 13.0, 6.5 Hz, 1H), 6.88 (d, *J* = 7.3 Hz, 1H), 6.83 (t, *J* = 7.8 Hz, 1H), 6.68 (d, *J* = 7.1 Hz, 1H), 6.61 (d, *J* = 9.6 Hz, 1H), 5.58 – 5.49 (m, 1H), 4.65 – 4.56 (m, 1H), 4.36 (d, *J* = 3.6 Hz, 1H); **^{13}C NMR (151 MHz, $CDCl_3$)** δ 163.33, 161.70, 159.84, 148.83, 143.34, 143.30, 143.26, 136.56, 133.43, 132.56, 132.50, 132.10, 131.92, 131.91, 131.80, 131.78, 131.73, 131.67, 131.23, 129.44, 129.39, 128.64, 128.56, 128.34, 128.26, 123.33, 122.67, 122.65, 114.01, 113.91, 113.87, 113.77, 60.97, 60.96, 60.06; **HRMS (ESI)**: calcd. for $C_{25}H_{24}FN_3OP(M+H)^+$: 432.1636, found: 432.1633.

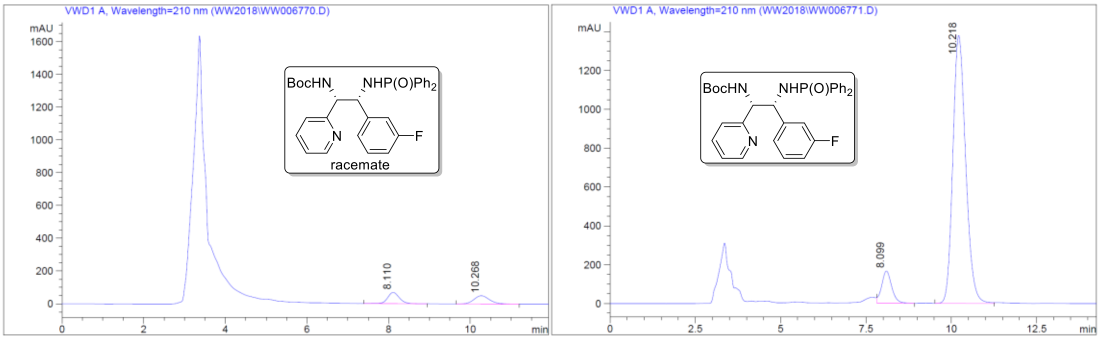

Meas.RT min	Area mAU*sec	Height mAU	Area %	Width min	Symm.
8.11	1358.1	68.3	50.7	0.332	0.825
10.27	1323.2	49.9	49.3	0.414	0.852

Meas.RT min	Area mAU*sec	Height mAU	Area %	Width min	Symm.
8.10	3371.9	165.6	8.6	0.339	0.804
10.22	35973.3	1377.8	91.4	0.404	0.786

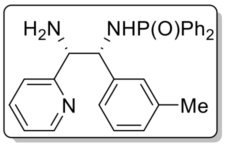

N-((1*R*,2*R*)-2-amino-2-(pyridin-2-yl)-1-(*m*-tolyl)ethyl)-P,P-diphenylphosphinic amide (*syn*-8c): white solid (25.1 mg, 59%); m.p. = 119-120 ^oC; R_f = 0.23 (CH_2Cl_2/MeOH = 25:1); the enantiomeric excess was determined to be 87% by HPLC analysis after being converted to the corresponding *N*-Boc derivatives. **HPLC**: Daicel Chirapak IA-H column (hexane/isopropanol = 80/20, flow rate 1.0 mL/min, T = 30°C), UV 210 nm, t_R(major) 14.83 min, t_R(minor) 11.93 min; $[\alpha]_D^{25}$ = +171.3 (c = 0.30, MeOH); **^{1}H NMR (600 MHz, $CDCl_3$)** δ 8.43 (d, *J* = 4.6 Hz, 1H), 7.81 (dd, *J* = 11.9, 7.4 Hz, 2H), 7.69 (dd, *J* = 12.0, 7.6 Hz, 2H), 7.49 (q, *J* = 6.5 Hz, 2H), 7.44 – 7.37 (m, 3H), 7.29 – 7.24 (m, 2H), 7.14 (dd, *J* = 7.0, 5.2 Hz, 1H), 7.03 (t, *J* = 7.5 Hz, 1H), 6.96 (d, *J* = 7.5 Hz, 1H), 6.87 (d, *J* = 7.7 Hz, 1H), 6.72 (d, *J* = 7.5 Hz, 1H), 6.68 (s, 1H), 5.35 (dd, *J* = 10.3, 6.3 Hz, 1H), 4.65 (td, *J* = 11.0, 4.2 Hz, 1H), 4.57 (d, *J* = 4.1 Hz, 1H), 2.19 (s, 3H); **^{13}C NMR (151 MHz, $CDCl_3$)** δ 158.34, 148.70, 139.41, 139.37, 137.60, 136.49, 133.22, 132.64, 132.58, 132.36, 131.90, 131.89, 131.84, 131.82, 131.69, 131.63, 130.79, 128.59, 128.51, 128.36, 128.28, 128.04, 127.94, 127.73, 123.91, 123.75, 122.81, 60.66, 59.90, 21.33; **HRMS (ESI)**: calcd. for $C_{26}H_{27}N_3OP(M+H)^+$: 428.1886, found: 428.1880.

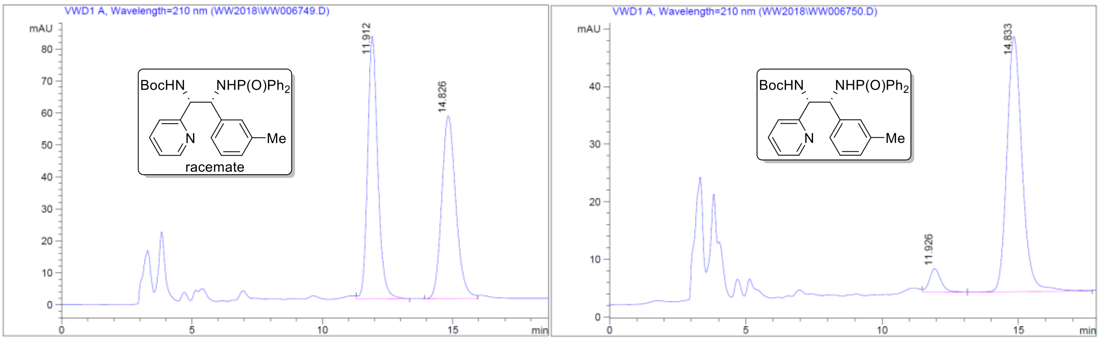

Meas.RT min	Area mAU*sec	Height mAU	Area %	Width min	Symm.
11.91	2221.7	82.2	50.7	0.414	0.790
14.83	2160.2	57.1	49.3	0.581	0.815

Meas.RT min	Area mAU*sec	Height mAU	Area %	Width min	Symm.
11.93	118.5	4.1	6.4	0.485	0.845
14.83	1728.7	44.3	93.6	0.650	0.788

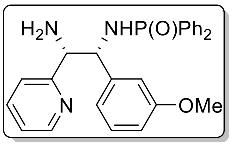

N-((1*R*,2*R*)-2-amino-1-(3-methoxyphenyl)-2-(pyridin-2-yl)ethyl)-P,P-diphenylphosphinic amide (*syn*-8d): white solid (29.7 mg, 67%); m.p. =

123-124 ^{o}C; R_f = 0.20 (CH_2Cl_2/MeOH = 25:1); the enantiomeric excess was determined to be 84% by HPLC analysis after being converted to the corresponding *N*-Boc derivatives. **HPLC**: Daicel Chirapak IA-H column (hexane/isopropanol = 80/20, flow rate 1.0 mL/min, T = 30°C), UV 210 nm, t_R(major) 18.48 min, t_R(minor) 12.73 min; $[\alpha]_D^{25}$ = +157.0 (c = 0.30, MeOH); **^{1}H NMR (600 MHz, $CDCl_3$)** δ 8.43 (d, *J* = 4.4 Hz, 1H), 7.84 (dd, *J* = 11.8, 7.4 Hz, 2H), 7.70 (dd, *J* = 12.0, 7.6 Hz, 2H), 7.51 – 7.46 (m, 2H), 7.45 – 7.40 (m, 2H), 7.38 (t, *J* = 7.1 Hz, 1H), 7.27 – 7.22 (m, 2H), 7.12 (dd, *J* = 7.0, 5.2 Hz, 1H), 7.05 (t, *J* = 7.9 Hz, 1H), 6.89 (d, *J* = 7.8 Hz, 1H), 6.68 (dd, *J* = 8.1, 2.1 Hz, 1H), 6.50 (d, *J* = 7.5 Hz, 1H), 6.43 (s, 1H), 5.42 (dd, *J* = 9.9, 6.5 Hz, 1H), 4.58 (td, *J* = 11.2, 4.4 Hz, 1H), 4.38 (d, *J* = 4.5 Hz, 1H), 3.63 (s, 3H); **^{13}C NMR (151 MHz, $CDCl_3$)** δ 160.21, 159.25, 148.71, 142.07, 142.03, 136.41, 133.62, 132.77, 132.60, 132.54, 132.25, 131.77, 131.76, 131.73, 131.67, 131.63, 131.37, 128.93, 128.53, 128.45, 128.26, 128.18, 123.37, 122.48, 119.36, 112.76, 112.55, 77.24, 61.20, 61.18, 60.55, 55.12; **HRMS (ESI)**: calcd. for $C_{26}H_{27}N_3O_2P(M+H)^+$: 444.1835, found: 444.1839.

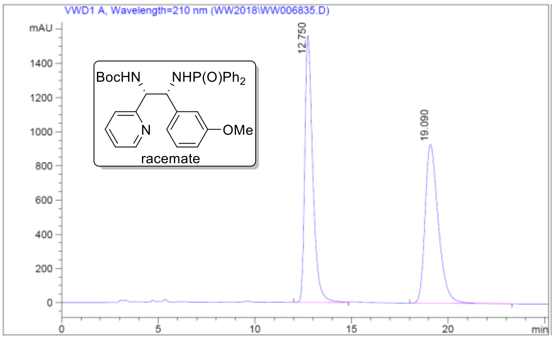

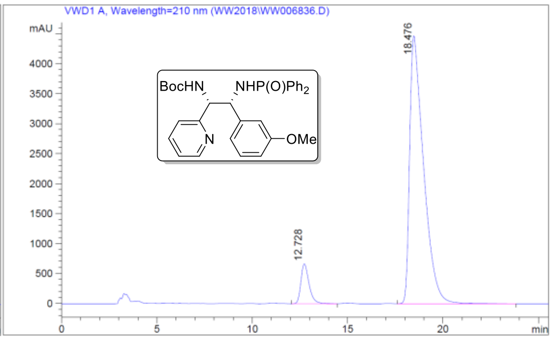

Meas.RT min	Area mAU*sec	Height mAU	Area %	Width min	Symm.
12.75	44967.2	1562.1	50.4	0.435	0.586
19.09	44260.4	929.5	49.6	0.730	0.639

Meas.RT min	Area mAU*sec	Height mAU	Area %	Width min	Symm.
12.73	19844.3	666.8	8.0	0.452	0.659
18.48	227401.4	4474.2	92.0	0.721	0.407

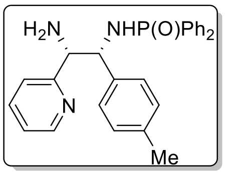

N-((1*R*,2*R*)-2-amino-2-(pyridin-2-yl)-1-(p-tolyl)ethyl)-P,P-diphenylphosphinic amide (*syn*-8e): white solid (32.7 mg, 77%); m.p. = 109-110 ^{o}C; R_f = 0.26 (CH_2Cl_2/MeOH = 25:1); the enantiomeric excess was determined to be 85% by HPLC analysis after being converted to the corresponding *N*-Boc derivatives. **HPLC**: Daicel Chirapak IC-H column (hexane/isopropanol = 70/30, flow rate 1.0 mL/min, T = 30°C), UV 210 nm, t_R(major) 18.64 min, t_R(minor) 9.98 min; $[\alpha]_D^{25}$ = +195.0 (c = 0.40, MeOH); **^{1}H NMR (600 MHz, $CDCl_3$)** δ 8.42 (d, *J* = 3.6 Hz, 1H), 7.82 (dd, *J* = 11.5, 7.7 Hz, 2H), 7.68 (dd, *J* = 11.6, 7.9 Hz, 2H), 7.51 – 7.46 (m, 2H), 7.44 – 7.36 (m, 3H), 7.27 – 7.23 (m, 2H), 7.16 – 7.09 (m, 1H), 6.94 (d, *J* = 7.6 Hz, 2H), 6.88 (d, *J* = 7.7 Hz, 1H), 6.79 (d, *J* = 7.7 Hz, 2H), 5.36 (dd, *J* = 9.7, 6.2 Hz, 1H), 4.59 (td, *J* = 10.9, 3.6 Hz, 1H), 4.46 (d, *J* = 3.7 Hz, 1H), 2.26 (s, 3H); **^{13}C NMR (151 MHz, $CDCl_3$)** δ 159.35, 148.71, 136.92, 136.88, 136.70, 136.46, 133.41, 132.62, 132.55, 131.96, 131.83, 131.81, 131.75, 131.69, 131.09, 128.72, 128.56, 128.48, 128.33, 128.25, 126.80, 123.57, 122.62, 61.01, 61.00, 60.06, 21.05; **HRMS (ESI)**: calcd. for

$C_{26}H_{27}N_3OP(M+H)^+$: 428.1886, found: 428.1880.

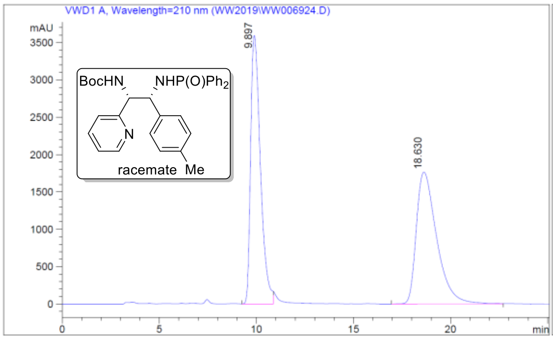

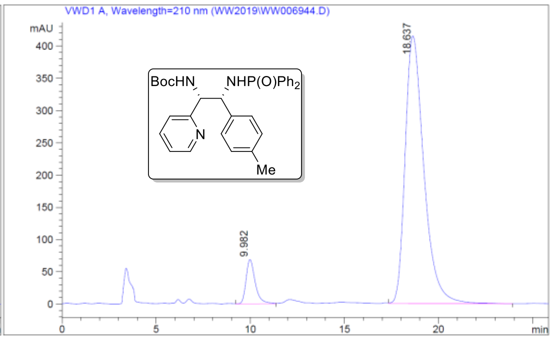

Meas.RT min	Area mAU*sec	Height mAU	Area %	Width min	Symm.
9.90	125391.1	3593.0	49.4	0.582	0.580
18.63	128404.7	1764.8	50.6	1.111	0.550

Meas.RT min	Area mAU*sec	Height mAU	Area %	Width min	Symm.
9.98	2332.2	68.8	7.5	0.512	0.687
18.64	28743.0	414.9	92.5	1.059	0.649

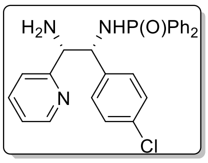

N-((1*R*,2*R*)-2-amino-1-(4-chlorophenyl)-2-(pyridin-2-yl)ethyl)-P,P-diphenylphosphinic amide (*syn*-8f): white solid (27.4 mg, 61%); m.p. = 128-129 °C; R_f = 0.24 (CH_2Cl_2/MeOH = 25:1); the enantiomeric excess was determined to be 82% by HPLC analysis after being converted to the corresponding *N*-Boc derivatives. **HPLC**: Daicel Chirapak IA-H column (hexane/isopropanol = 80/20, flow rate 1.0 mL/min, T = 30°C), UV 210 nm, t_R(major) 12.44 min, t_R(minor) 22.61 min; $[\alpha]_D^{25}$ = +195.0 (c = 0.30, MeOH); **^{1}H NMR (600 MHz, $CDCl_3$)** δ 8.41 (d, *J* = 4.5 Hz, 1H), 7.83 (dd, *J* = 11.9, 7.3 Hz, 2H), 7.67 (dd, *J* = 12.0, 7.4 Hz, 2H), 7.52 – 7.47 (m, 2H), 7.46 – 7.37 (m, 3H), 7.29 – 7.24 (m, 2H), 7.13 (dd, *J* = 7.1, 5.3 Hz, 1H), 7.09 (d, *J* = 8.3 Hz, 2H), 6.90 (d, *J* = 7.8 Hz, 1H), 6.83 (d, *J* = 8.3 Hz, 2H), 5.56 (dd, *J* = 10.0, 6.3 Hz, 1H), 4.61 (td, *J* = 11.1, 4.2 Hz, 1H), 4.42 (d, *J* = 4.2 Hz, 1H); **^{13}C NMR (151 MHz, $CDCl_3$)** δ 159.43, 148.81, 138.82, 138.79, 136.60, 133.27, 132.90, 132.51, 132.45, 131.91, 131.90, 131.81, 131.79, 131.70, 131.63, 131.11, 128.61, 128.53, 128.36, 128.28, 128.09, 123.41, 122.69, 60.84, 60.82, 59.73; **HRMS (ESI)**: calcd. for $C_{25}H_{24}ClN_3OP(M+H)^+$: 448.1340, found: 448.1333.

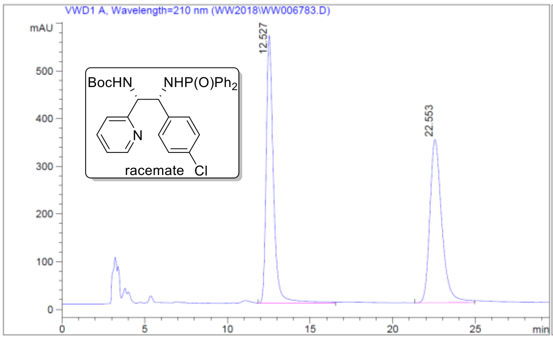

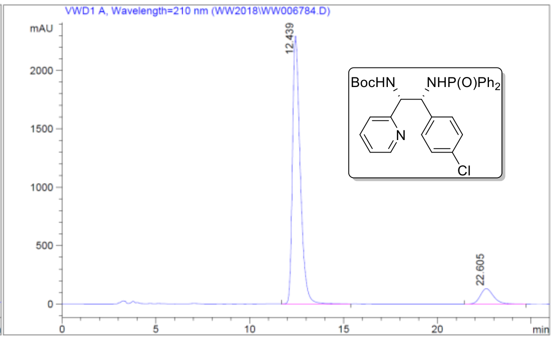

Meas.RT min	Area mAU*sec	Height mAU	Area %	Width min	Symm.
12.53	17034.4	560.9	49.8	0.453	0.627
22.55	17163.7	342.0	50.2	0.760	0.691

Meas.RT min	Area mAU*sec	Height mAU	Area %	Width min	Symm.
12.44	65632.0	2296.2	91.1	0.432	0.589
22.61	6436.4	131.7	8.9	0.751	0.778

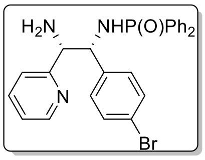

N-((1*R*,2*R*)-2-amino-1-(4-bromophenyl)-2-(pyridin-2-yl)ethyl)-P,P-diphenylphosphinic amide (*syn*-8g): white solid (32.6 mg, 66%); m.p. = 178-179 ^{o}C; R_f = 0.24 (CH_2Cl_2/MeOH = 25:1); R_f = 0.23 (CH_2Cl_2/MeOH = 25:1); the enantiomeric excess was determined to be 84% by HPLC analysis after being converted to the corresponding *N*-Boc derivatives. **HPLC**: Daicel Chirapak IA-H column (hexane/isopropanol = 80/20, flow rate 1.0 mL/min, T = 30°C), UV 210 nm, t_R(major) 13.66 min, t_R(minor) 24.89 min; $[\alpha]_D^{25}$ = +237.5 (c = 0.24, MeOH); **^{1}H NMR (600 MHz, $CDCl_3$)** δ 8.42 (d, *J* = 3.4 Hz, 1H), 7.84 (dd, *J* = 11.0, 8.1 Hz, 2H), 7.69 (dd, *J* = 11.1, 8.1 Hz, 2H), 7.55 – 7.37 (m, 5H), 7.28 – 7.22 (m, 4H), 7.15 – 7.09 (m, 1H), 6.90 (d, *J* = 7.6 Hz, 1H), 6.76 (d, *J* = 8.1 Hz, 2H), 5.56 – 5.47 (m, 1H), 4.55 (td, *J* = 11.1, 3.8 Hz, 1H), 4.35 (d, *J* = 3.6 Hz, 1H); **^{13}C NMR (151 MHz, $CDCl_3$)** δ 159.92, 148.83, 139.59, 139.55, 136.59, 133.40, 132.53, 132.47, 132.17, 131.90, 131.88, 131.79, 131.78, 131.74, 131.68, 131.29, 131.02, 128.69, 128.62, 128.54, 128.37, 128.29, 123.32, 122.63, 121.00, 60.98, 60.96, 60.03; **HRMS (ESI)**: calcd. for $C_{25}H_{24}BrN_3OP(M+H)^+$: 492.0835, found: 492.0831.

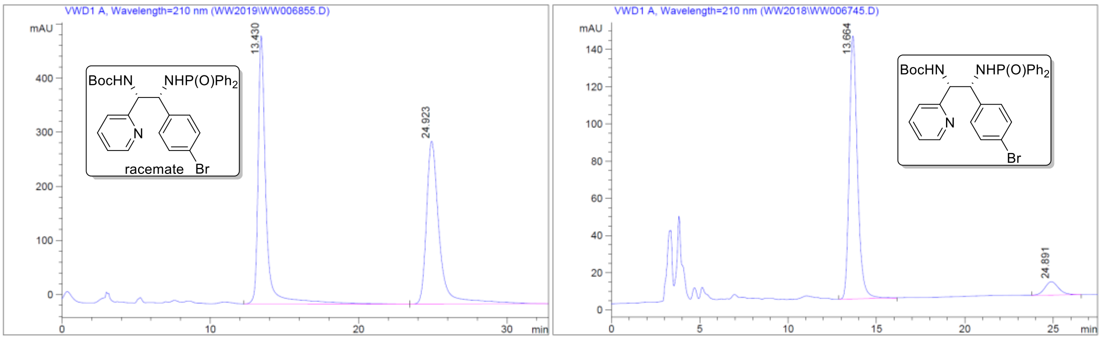

Meas.RT min	Area mAU*sec	Height mAU	Area %	Width min	Symm.
13.43	18426.1	494.8	50.4	0.545	0.561
24.92	18157.7	300.8	49.6	0.902	0.600

Meas.RT min	Area mAU*sec	Height mAU	Area %	Width min	Symm.
13.66	4572.6	141.3	92.1	0.494	0.732
24.89	394.9	7.2	7.9	0.908	0.870

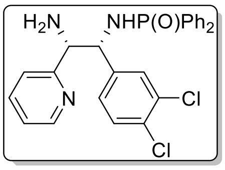

N-((1*R*,2*R*)-2-amino-1-(3,4-dichlorophenyl)-2-(pyridin-2-yl)ethyl)-P,P-diphenylphosphinic amide (*syn*-8h): white solid (27.2 mg, 56%); m.p. = 102-103 ^{o}C; R_f = 0.20 (CH_2Cl_2/MeOH = 25:1); the enantiomeric excess was determined to be 74% by HPLC analysis after being converted to the corresponding *N*-Boc derivatives. **HPLC**: Daicel Chirapak IA-H column (hexane/isopropanol = 80/20, flow rate 1.0 mL/min, T = 30°C), UV 210 nm, t_R(major) 17.17 min, t_R(minor) 21.37 min; $[\alpha]_D^{25}$ = +78.9 (c = 0.35, MeOH); **^{1}H NMR (600 MHz, $CDCl_3$)** δ 8.42 (d, *J* = 4.0 Hz, 1H), 7.85 (dd, *J* = 11.7, 7.5 Hz, 2H), 7.68 (dd, *J* = 11.8, 7.7 Hz, 2H), 7.56 – 7.50 (m, 2H), 7.48 – 7.38 (m, 3H), 7.32 – 7.28 (m, 2H), 7.18 – 7.13 (m, 2H), 6.97 – 6.90 (m, 2H), 6.70 (d, *J* = 8.2 Hz, 1H), 5.54 (dd, *J* = 9.1, 6.9 Hz, 1H), 4.56 – 4.48 (m, 1H), 4.31 (d, *J* = 4.3 Hz, 1H); **^{13}C NMR (151 MHz, $CDCl_3$)** δ 160.16, 148.88, 141.04, 141.00, 136.68, 132.44, 132.38, 131.98, 131.97, 131.92, 131.86, 131.84, 131.72, 131.66, 130.97, 129.79, 129.06, 128.67, 128.58, 128.39, 128.31, 126.47, 123.12,

122.70, 60.98, 60.96, 59.73; **HRMS (ESI)**: calcd. for $C_{25}H_{23}Cl_2N_3OP(M+H)^+$: 482.0950, found: 482.0942.

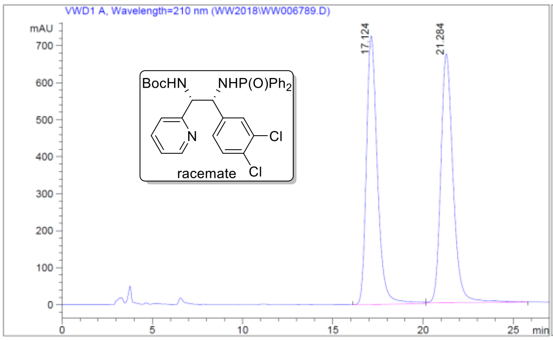

Meas.RT min	Area mAU*sec	Height mAU	Area %	Width min	Symm.
17.12	30766.4	725.4	49.3	0.646	0.692
21.28	31669.6	672.3	50.7	0.785	0.775

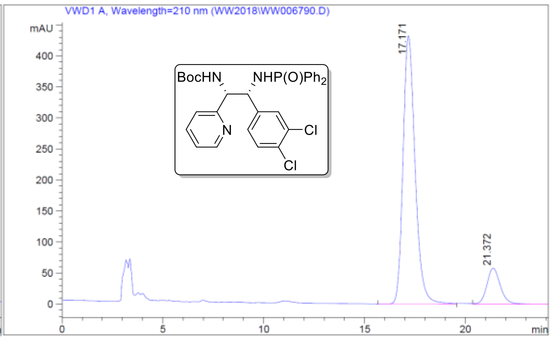

Meas.RT min	Area mAU*sec	Height mAU	Area %	Width min	Symm.
17.17	17945.8	431.9	87.1	0.636	0.744
21.37	2652.1	57.8	12.9	0.704	0.839

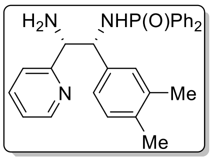

N-((1*R*,2*R*)-2-amino-1-(3,4-dimethylphenyl)-2-(pyridin-2-yl)ethyl)-P,P-diphenylphosphinic amide (*syn*-8i): white solid (32.0 mg, 73%); m.p. = 122-123 °C; R_f = 0.27 (CH_2Cl_2/MeOH = 25:1); the enantiomeric excess was determined to be 87% by HPLC analysis after being converted to the corresponding *N*-Boc derivatives. **HPLC**: Daicel Chirapak IA-H column (hexane/isopropanol = 80/20, flow rate 1.0 mL/min, T = 30°C), UV 210 nm, t_R(major) 18.54 min, t_R(minor) 12.47 min; $[\alpha]_D^{25}$ = +198.1 (c = 0.36, MeOH); **^{1}H NMR (600 MHz, $CDCl_3$)** δ 8.43 (d, *J* = 4.4 Hz, 1H), 7.84 – 7.77 (m, 2H), 7.71 – 7.65 (m, 2H), 7.51 – 7.46 (m, 2H), 7.44 – 7.36 (m, 3H), 7.27 – 7.24 (m, 2H), 7.13 (dd, *J* = 7.0, 5.3 Hz, 1H), 6.90 (dd, *J* = 15.4, 7.6 Hz, 2H), 6.64 – 5.56 (m, 2H), 5.22 (dd, *J* = 10.2, 6.4 Hz, 1H), 4.52 (td, *J* = 10.9, 4.6 Hz, 1H), 4.39 (d, *J* = 4.6 Hz, 1H), 2.17 (s, 3H), 2.09 (s, 3H); **^{13}C NMR (151 MHz, $CDCl_3$)** δ 160.18, 148.64, 137.44, 137.41, 136.34, 135.94, 135.23, 133.59, 132.74, 132.59, 132.53, 132.29, 131.76, 131.70, 131.60, 131.59, 131.41, 129.22, 128.49, 128.41, 128.33, 128.25, 128.16, 124.30, 123.42, 122.45, 61.34, 61.31, 60.37, 19.66, 19.33; **HRMS (ESI)**: calcd. for $C_{27}H_{29}N_3OP(M+H)^+$: 442.2043, found: 442.2037.

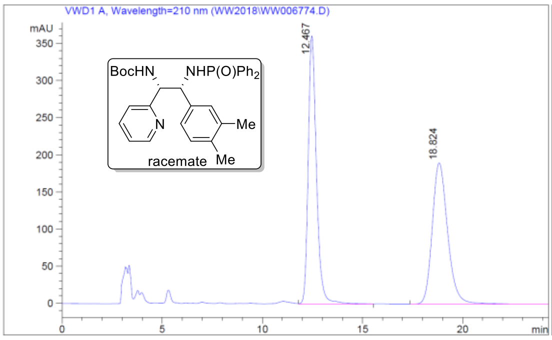

Meas.RT min	Area mAU*sec	Height mAU	Area %	Width min	Symm.
12.47	10285.7	360.9	50.4	0.432	0.713
18.82	10137.3	190.2	49.6	0.820	0.764

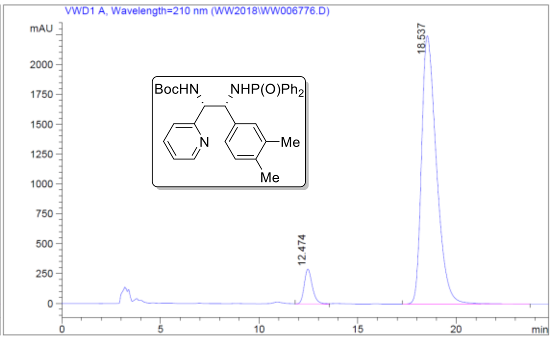

Meas.RT min	Area mAU*sec	Height mAU	Area %	Width min	Symm.
12.47	8326.1	291.0	6.6	0.433	0.751
18.54	118023.1	2244.3	93.4	0.800	0.589

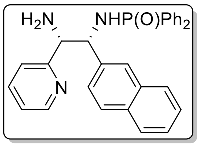

N-((1*R*,2*R*)-2-amino-1-(naphthalen-2-yl)-2-(pyridin-2-yl)ethyl)-P,P-diphenylphosphinic amide (*syn*-8j): white solid (36.4 mg, 79%); m.p. = 174-175 ^{o}C; R_f = 0.27 (CH_2Cl_2/MeOH = 25:1); the enantiomeric excess was determined to be 81% by HPLC analysis after being converted to the corresponding *N*-Boc derivatives. **HPLC**: Daicel Chirapak IA-H column (hexane/isopropanol = 80/20, flow rate 1.0 mL/min, T = 30°C), UV 210 nm, t_R(major) 25.68 min, t_R(minor) 21.37 min; $[\alpha]_D^{25}$ = +291.0 (c = 0.30, MeOH); **^{1}H NMR (600 MHz, $CDCl_3$)** δ 8.42 (d, *J* = 3.9 Hz, 1H), 7.87 (dd, *J* = 11.6, 7.6 Hz, 2H), 7.77 – 7.72 (m, 1H), 7.69 (dd, *J* = 11.7, 7.8 Hz, 2H), 7.65 – 7.62 (m, 1H), 7.60 (d, *J* = 8.4 Hz, 1H), 7.50 (t, *J* = 7.1 Hz, 1H), 7.46 – 7.38 (m, 5H), 7.35 – 7.29 (m, 2H), 7.21 – 7.14 (m, 2H), 7.12 – 7.06 (m, 1H), 6.99 (d, *J* = 8.4 Hz, 1H), 6.85 (d, *J* = 7.7 Hz, 1H), 5.54 (dd, *J* = 9.2, 6.4 Hz, 1H), 4.74 (td, *J* = 11.1, 4.4 Hz, 1H), 4.43 (d, *J* = 4.3 Hz, 1H); **^{13}C NMR (151 MHz, $CDCl_3$)** δ 160.63, 148.76, 138.13, 138.09, 136.41, 133.72, 132.98, 132.87, 132.58, 132.52, 132.31, 131.78, 131.71, 131.62, 131.60, 131.43, 128.56, 128.47, 128.24, 128.16, 127.90, 127.59, 127.53, 125.97, 125.89, 125.68, 124.99, 123.26, 122.44, 61.44, 61.43, 60.97; **HRMS (ESI)**: calcd. for $C_{29}H_{27}N_3OP(M+H)^+$: 464.1886, found: 428.1877.

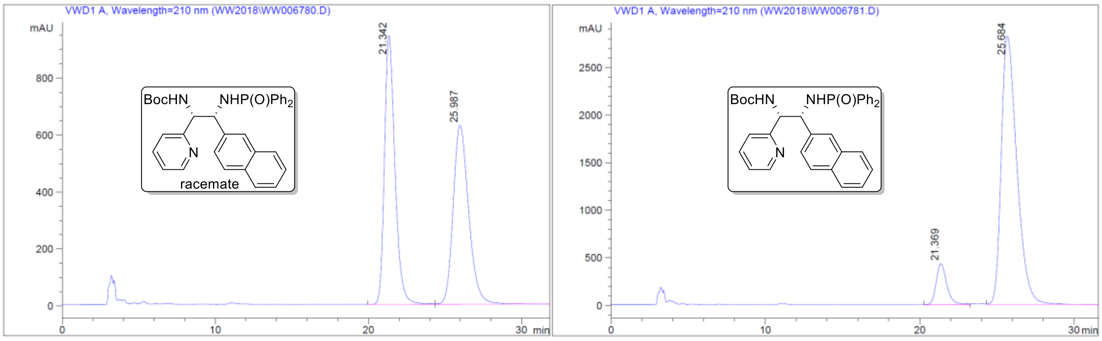

Meas.RT min	Area mAU*sec	Height mAU	Area %	Width min	Symm.
21.34	44862.4	942.6	50.4	0.724	0.698
25.99	44071.6	627.4	49.6	1.076	0.750

Meas.RT min	Area mAU*sec	Height mAU	Area %	Width min	Symm.
21.37	20540.1	430.7	9.4	0.731	0.732
25.68	198419.8	2820.6	90.6	1.077	0.607

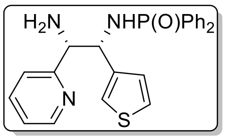

N-((1*R*,2*R*)-2-amino-2-(pyridin-2-yl)-1-(thiophen-3-yl)ethyl)-P,P-diphenylphosphinic amide (*syn*-8k): white solid (27.5 mg, 66%); m.p. = 177-178 ^{o}C; R_f = 0.34 (CH_2Cl_2/MeOH = 25:1); the enantiomeric excess was determined to be 89% by HPLC analysis after being converted to the corresponding *N*-Boc derivatives. **HPLC**: Daicel Chirapak IA-H column (hexane/isopropanol = 80/20, flow rate 1.0 mL/min, T = 30°C), UV 210 nm, t_R(major) 11.88 min, t_R(minor) 16.36 min; $[\alpha]_D^{25}$ = +174.0 (c = 0.40, MeOH); **^{1}H NMR (600 MHz, $CDCl_3$)** δ 8.43 (d, *J* = 4.3 Hz, 1H), 7.84 (dd, *J* = 11.8, 7.3 Hz, 2H), 7.74 (dd, *J* = 11.9, 7.4 Hz, 2H), 7.53 – 7.47 (m, 2H), 7.45 – 7.39 (m, 3H), 7.33 – 7.28 (m, 2H), 7.12 (dd, *J* = 6.9, 5.2 Hz, 1H), 7.09 (dd, *J* = 4.8, 3.0 Hz, 1H), 6.99 (d, *J* = 7.8 Hz, 1H), 6.76 (d, *J* = 1.9 Hz, 1H), 6.59 (d, *J* = 4.9 Hz, 1H), 5.31 (dd, *J* = 9.7, 7.2 Hz, 1H), 4.69 (td, *J* = 10.7, 4.3 Hz, 1H), 4.39 (d, *J* = 4.3 Hz, 1H); **^{13}C NMR (151 MHz, $CDCl_3$)** δ 160.84, 148.70, 141.92, 141.89,

136.41, 133.67, 132.82, 132.56, 132.49, 131.73, 131.67, 131.64, 128.53, 128.44, 128.30, 128.22, 126.38, 125.24, 122.94, 122.38, 121.72, 61.02, 61.01, 57.13; **HRMS (ESI)**: calcd. for $C_{23}H_{23}N_3OPS(M+H)^+$: 420.1294, found: 420.1292.

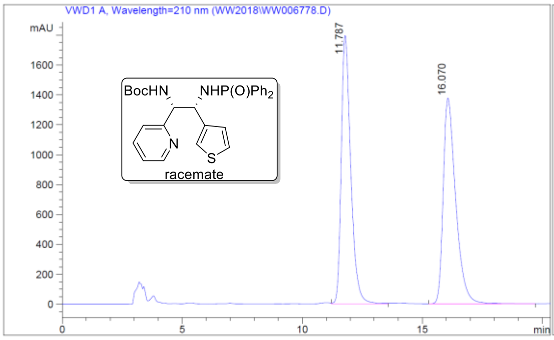

Meas.RT min	Area mAU*sec	Height mAU	Area %	Width min	Symm.
11.79	49279.5	1793.7	49.7	0.420	0.634
16.07	49836.9	1375.8	50.3	0.545	0.562

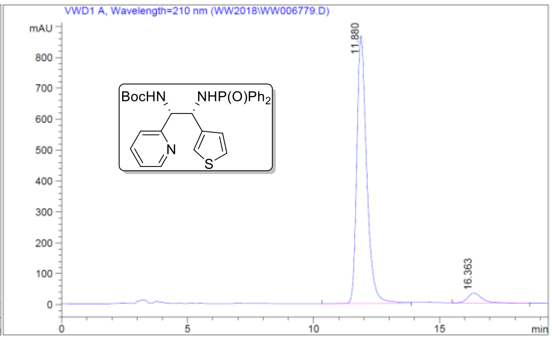

Meas.RT min	Area mAU*sec	Height mAU	Area %	Width min	Symm.
11.88	23824.8	865.4	94.6	0.414	0.654
16.36	1365.6	32.9	5.4	0.618	0.738

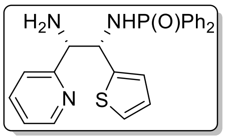

N-((1*S*,2*R*)-2-amino-2-(pyridin-2-yl)-1-(thiophen-2-yl)ethyl)-P,P-diphenylphosphinic amide (*syn*-8l): white solid (37.0 mg, 88%); m.p. = 125-126 ^oC; R_f = 0.34 (CH_2Cl_2/MeOH = 25:1); the enantiomeric excess was determined to be 83% by HPLC analysis after being converted to the corresponding *N*-Boc derivatives. **HPLC**: Daicel Chirapak IC-H column (hexane/isopropanol = 70/30, flow rate 1.0 mL/min, T = 30°C), UV 210 nm, t_R(major) 18.95 min, t_R(minor) 10.31 min; $[\alpha]^D_{25}$ = +105.0 (c = 0.30, MeOH); **^{1}H NMR (600 MHz, $CDCl_3$)** δ 8.42 (d, *J* = 3.6 Hz, 1H), 7.90 (dd, *J* = 11.5, 7.7 Hz, 2H), 7.82 (dd, *J* = 11.8, 7.6 Hz, 2H), 7.57 – 7.47 (m, 2H), 7.47 – 7.38 (m, 3H), 7.35 – 7.30 (m, 2H), 7.14 – 7.10 (m, 1H), 7.09 – 7.04 (m, 2H), 6.80 – 6.75 (m, 1H), 6.54 (s, 1H), 5.57 (dd, *J* = 9.5, 6.5 Hz, 1H), 4.89 (td, *J* = 10.9, 3.8 Hz, 1H), 4.51 (d, *J* = 3.8 Hz, 1H); **^{13}C NMR (151 MHz, $CDCl_3$)** δ 159.89, 148.75, 144.70, 144.65, 136.59, 133.40, 132.63, 132.57, 131.93, 131.91, 131.83, 131.81, 131.74, 131.06, 128.62, 128.53, 128.40, 128.32, 126.58, 124.66, 124.15, 123.30, 122.58, 61.07, 61.06, 56.86; **HRMS (ESI)**: calcd. for $C_{23}H_{23}N_3OPS(M+H)^+$: 420.1294, found: 420.1296.

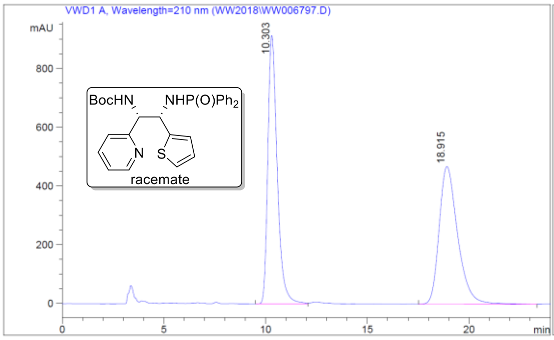

Meas.RT min	Area mAU*sec	Height mAU	Area %	Width min	Symm.
10.30	29337.6	913.8	49.8	0.491	0.661
18.92	29629.7	468.7	50.2	0.959	0.677

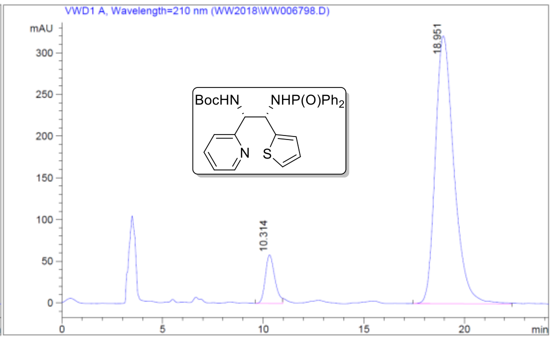

Meas.RT min	Area mAU*sec	Height mAU	Area %	Width min	Symm.
10.31	1900.2	58.2	8.4	0.545	0.769
18.95	20717.4	321.2	91.6	0.977	0.707

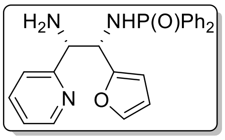

N-((1*S*,2*R*)-2-amino-1-(furan-2-yl)-2-(pyridin-2-yl)ethyl)-P,P-diphenylphosphinic amide (*syn*-8m): white solid (26.6 mg, 66%); m.p. = 103-104 ^{o}C; R_f = 0.32 (CH_2Cl_2/MeOH = 25:1); the enantiomeric excess was determined to be 84% by HPLC analysis after being converted to the corresponding *N*-Boc derivatives. **HPLC**: Daicel Chirapak IC-H column (hexane/isopropanol = 70/30, flow rate 1.0 mL/min, T = 30°C), UV 210 nm, t_R(major) 22.22 min, t_R(minor) 13.84 min; $[\alpha]_D^{25}$ = +119.7 (c = 0.31, MeOH); **^{1}H NMR (600 MHz, CDCl$_3$)** δ 8.43 (d, *J* = 4.4 Hz, 1H), 7.80 (td, *J* = 12.5, 7.8 Hz, 4H), 7.54 (td, *J* = 7.7, 1.3 Hz, 1H), 7.51 – 7.47 (m, 1H), 7.45 – 7.40 (m, 3H), 7.35 (td, *J* = 7.6, 3.1 Hz, 2H), 7.21 (s, 1H), 7.12 (dd, *J* = 6.9, 5.3 Hz, 1H), 7.06 (d, *J* = 7.8 Hz, 1H), 6.15 (dd, *J* = 2.6, 1.7 Hz, 1H), 5.93 (d, *J* = 3.0 Hz, 1H), 5.00 (dd, *J* = 9.8, 8.1 Hz, 1H), 4.65 (td, *J* = 10.6, 4.7 Hz, 1H), 4.53 (d, *J* = 4.6 Hz, 1H); **^{13}C NMR (151 MHz, CDCl$_3$)** δ 160.15, 153.56, 148.81, 141.64, 136.52, 133.28, 132.53, 132.46, 132.29, 131.89, 131.88, 131.84, 131.82, 131.79, 131.73, 131.41, 128.58, 128.50, 128.44, 128.36, 122.71, 122.50, 110.14, 107.53, 59.71, 55.04; **HRMS (ESI)**: calcd. for $C_{23}H_{23}N_3O_2P(M+H)^+$: 404.1522, found: 404.1515.

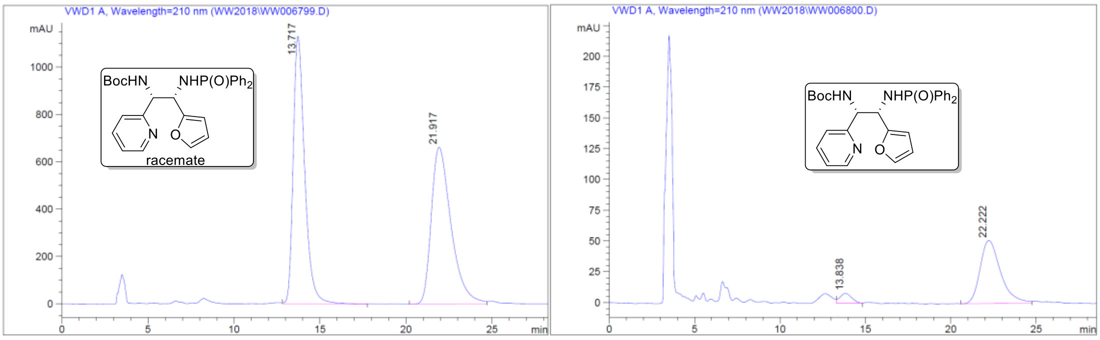

Meas.RT min	Area mAU*sec	Height mAU	Area %	Width min	Symm.
13.72	52425.7	1130.0	49.8	0.707	0.647
21.92	52908.8	662.3	50.2	1.209	0.601

Meas.RT min	Area mAU*sec	Height mAU	Area %	Width min	Symm.
13.84	383.1	8.0	8.2	0.715	0.907
22.22	4283.4	51.3	91.8	1.269	0.690

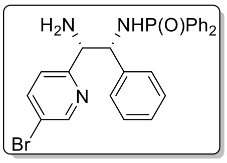

N-((1*R*,2*R*)-2-amino-2-(5-bromopyridin-2-yl)-1-phenylethyl)-P,P-diphenylphosphinic amide (*syn*-8n): white solid (41.5 mg, 84%); m.p. = 110-111 ^{o}C; R_f = 0.23 (CH_2Cl_2/MeOH = 25:1); the enantiomeric excess was determined to be 79% by HPLC analysis after being converted to the corresponding *N*-Boc derivatives. **HPLC**: Daicel Chirapak IA-H column (hexane/isopropanol = 70/30, flow rate 1.0 mL/min, T = 30°C), UV 210 nm, t_R(major) 20.20 min, t_R(minor) 8.38 min; $[\alpha]_D^{25}$ = +80.3 (c = 0.30, MeOH); **^{1}H NMR (600 MHz, CDCl$_3$)** δ 8.46 (s, 1H), 7.90 (dd, *J* = 11.9, 7.8 Hz, 2H), 7.83 (dd, *J* = 11.6, 7.6 Hz, 2H), 7.67 (dd, *J* = 11.7, 7.8 Hz, 2H), 7.58 (dd, *J* = 8.2, 1.7 Hz, 1H), 7.50 (dd, *J* = 16.2, 7.8 Hz, 2H), 7.38 (t, *J* = 7.2 Hz, 1H), 7.26 – 7.22 (m, 2H), 7.19 – 7.14 (m, 3H), 6.91 (d, *J* = 3.4 Hz, 2H), 6.79 (d, *J* = 8.2 Hz, 1H), 5.16 (dd, *J* = 9.9, 6.1 Hz, 1H), 4.59 (td, *J* = 11.1, 4.2 Hz, 1H), 4.40 (d, *J* = 3.9 Hz, 1H); **^{13}C NMR (151 MHz, CDCl$_3$)** δ 158.69, 149.85, 139.97, 139.94, 138.94, 133.81, 133.43, 132.95, 132.59, 132.52, 131.98, 131.91, 131.89, 131.87,

131.83, 131.79, 131.76, 131.71, 131.64, 131.10, 128.61, 128.55, 128.53, 128.47, 128.34, 128.26, 128.12, 127.25, 126.88, 124.59, 119.50, 60.88, 60.87, 60.26; **HRMS (ESI)**: calcd. for $C_{25}H_{24}BrN_3OP(M+H)^+$: 492.0835, found: 492.0821.

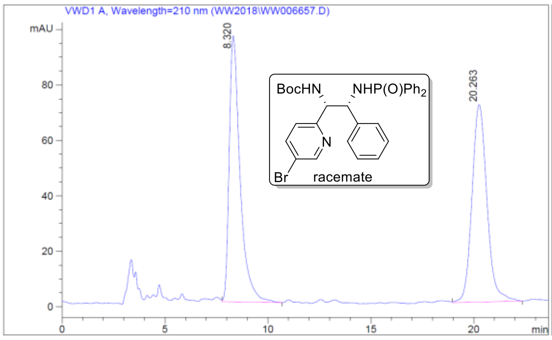

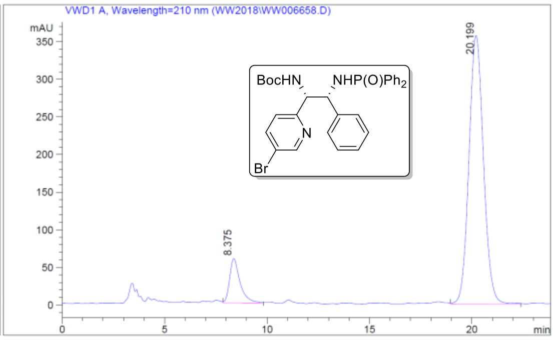

Meas.RT min	Area mAU*sec	Height mAU	Area %	Width min	Symm.
8.32	3518.8	95.9	49.5	0.550	0.545
20.26	3594.9	71.2	50.5	0.781	0.869

Meas.RT min	Area mAU*sec	Height mAU	Area %	Width min	Symm.
8.38	2082.3	58.8	10.6	0.524	0.604
20.20	17588.8	357.1	89.4	0.821	0.885

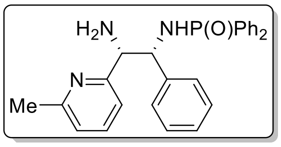

N-((1*R*,2*R*)-2-amino-2-(6-methylpyridin-2-yl)-1-phenylethyl)-P,P-diphenylphosphinic amide (*syn*-8o): white solid (33.3 mg, 80%); m.p. = 89-90 ^oC; R_f = 0.23 (CH_2Cl_2/MeOH = 25:1); the enantiomeric excess was determined to be 89% by HPLC analysis after being converted to the corresponding *N*-Boc derivatives. **HPLC**: Daicel Chirapak IC-H column (hexane/isopropanol = 70/30, flow rate 1.0 mL/min, T = 30°C), UV 210 nm, t_R(major) 14.64 min, t_R(minor) 7.54 min; $[\alpha]_D^{25}$ = +289.0 (c = 0.30, MeOH); **^{1}H NMR (600 MHz, $CDCl_3$)** δ 7.84 (dd, *J* = 11.6, 7.7 Hz, 2H), 7.69 (dd, *J* = 11.9, 7.7 Hz, 2H), 7.50 (t, *J* = 7.1 Hz, 1H), 7.46 – 7.41 (m, 2H), 7.39 – 7.32 (m, 2H), 7.27 –7.22 (m, 2H), 7.15 – 7.12 (m, 3H), 6.98 (d, *J* = 7.6 Hz, 1H), 6.95 – 6.91 (m, 2H), 6.59 (d, *J* = 7.5 Hz, 1H), 5.81 (dd, *J* = 8.6, 7.3 Hz, 1H), 4.64 (td, *J* = 11.3, 3.9 Hz, 1H), 4.38 (d, *J* = 3.7 Hz, 1H), 2.38 (s, 3H); **^{13}C NMR (151 MHz, $CDCl_3$)** δ 158.26, 157.55, 140.46, 140.42, 136.86, 133.60, 132.76, 132.66, 132.60, 131.97, 131.85, 131.83, 131.76, 131.74, 131.58, 131.52, 131.09, 128.56, 128.48, 128.30, 128.21, 127.97, 127.08, 126.89, 122.28, 120.73, 60.57, 60.55, 60.28, 24.23; **HRMS (ESI)**: calcd. for $C_{26}H_{27}N_3OP(M+H)^+$: 428.1886, found: 428.1882.

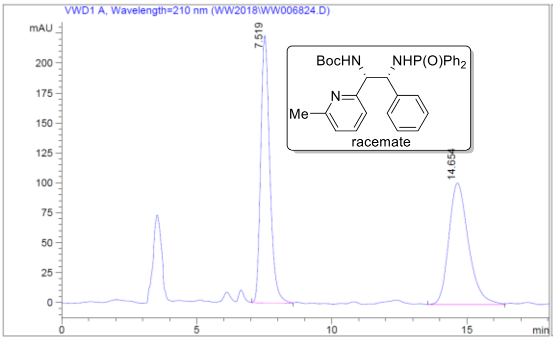

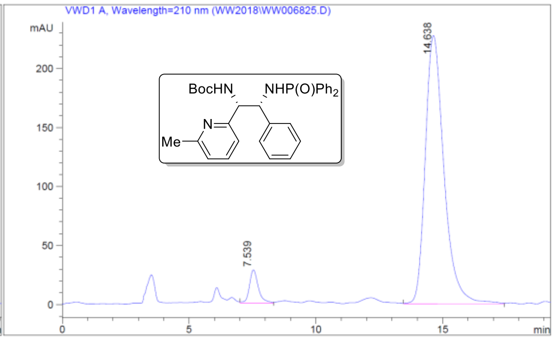

Meas.RT min	Area mAU*sec	Height mAU	Area %	Width min	Symm.
7.52	5226.5	223.3	50.4	0.360	0.742
14.65	5138.1	101.5	49.6	0.771	0.737

Meas.RT min	Area mAU*sec	Height mAU	Area %	Width min	Symm.
7.54	664.5	27.9	5.4	0.366	0.765
14.64	11665.3	227.3	94.6	0.779	0.713

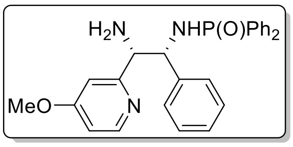

N-((1*R*,2*R*)-2-amino-2-(4-methoxypyridin-2-yl)-1-phenylethyl)-P,P-diphenylphosphinic amide (*syn*-8p): white solid (39.9 mg, 90%); m.p. = 152-153 °C; R_f = 0.20 (CH_2Cl_2/MeOH = 25:1); the enantiomeric excess was determined to be 83% by HPLC analysis after being converted to the corresponding *N*-Boc derivatives. **HPLC**: Daicel Chirapak IC-H column (hexane/isopropanol = 70/30, flow rate 1.0 mL/min, T = 30°C), UV 210 nm, t_R(major) 14.91 min, t_R(minor) 8.83 min; $[\alpha]_D^{25}$ = +88.9 (c = 0.21, MeOH); **^{1}H NMR (600 MHz, $CDCl_3$)** δ 8.23 (d, *J* = 5.7 Hz, 1H), 7.84 (dd, *J* = 11.6, 7.5 Hz, 2H), 7.69 (dd, *J* = 11.7, 7.6 Hz, 2H), 7.50 (t, *J* = 6.9 Hz, 1H), 7.46 – 7.40 (m, 2H), 7.37 (t, *J* = 7.0 Hz, 1H), 7.26 – 7.20 (m, 2H), 7.18 – 7.11 (m, 3H), 6.97 – 6.91 (m, 2H), 6.64 (dd, *J* = 5.6, 2.2 Hz, 1H), 6.34 (s, 1H), 5.53 – 5.45 (m, 1H), 4.57 (td, *J* = 11.3, 4.4 Hz, 1H), 4.28 (d, *J* = 4.4 Hz, 1H), 3.66 (s, 3H); **^{13}C NMR (151 MHz, $CDCl_3$)** δ 166.00, 161.17, 149.71, 140.32, 140.29, 133.45, 132.63, 132.56, 132.02, 131.84, 131.83, 131.74, 131.68, 131.14, 128.58, 128.49, 128.32, 128.24, 128.04, 127.15, 126.95, 109.64, 108.92, 61.16, 61.15, 60.28, 55.17; **HRMS (ESI)**: calcd. for $C_{26}H_{27}N_3O_2P(M+H)^+$: 444.1835, found: 444.1835.

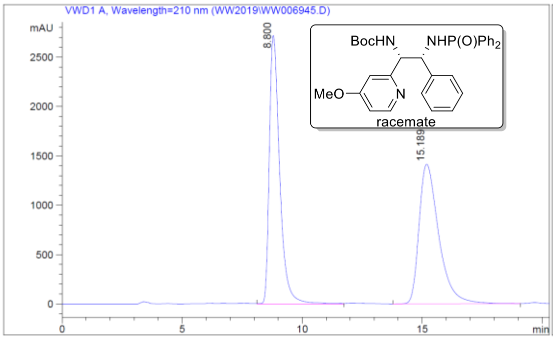

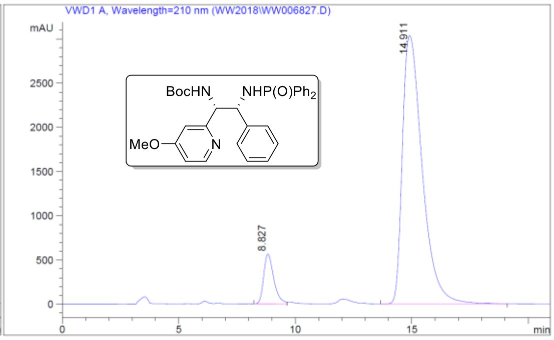

Meas.RT min	Area mAU*sec	Height mAU	Area %	Width min	Symm.
8.80	81604.1	2716.7	50.3	0.461	0.578
15.19	80694.8	1413.8	49.7	0.876	0.585

Meas.RT min	Area mAU*sec	Height mAU	Area %	Width min	Symm.
8.83	16913.5	563.4	8.6	0.500	0.682
14.91	179615.6	3034.4	91.4	0.912	0.528

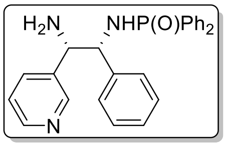

N-((1*R*,2*S*)-2-amino-1-phenyl-2-(pyridin-3-yl)ethyl)-P,P-diphenylphosphinic amide (*syn*-8q): Using (*S*)-**3d** as catalyst: white solid (18.6 mg, 45%); m.p. = 244-245 °C; R_f = 0.23 (CH_2Cl_2/MeOH = 25:1); the enantiomeric excess was determined to be 84% by HPLC analysis after being converted to the corresponding *N*-Boc derivatives. **HPLC**: Daicel Chirapak IA-H column (hexane/isopropanol = 70/30, flow rate 1.0 mL/min, T = 30°C), UV 210 nm, t_R(major) 6.97 min, t_R(minor) 15.04 min; $[\alpha]_D^{25}$ = -28.5 (c = 0.20, MeOH); **^{1}H NMR (600 MHz, CD_3OD)** δ 8.62 (d, *J* = 4.1 Hz, 1H), 8.54 (s, 1H), 7.92 (d, *J* = 7.9 Hz, 1H), 7.56 – 7.47 (m, 5H), 7.41 – 7.32 (m, 11H), 4.76 (d, *J* = 7.6 Hz, 1H), 4.73 – 4.68 (m, 1H); **^{13}C NMR (151 MHz, CD_3OD)** δ 149.61, 149.13, 138.16, 138.14, 136.58, 132.29, 132.28, 132.18, 132.17, 131.93, 131.86, 131.37, 131.28, 131.21, 131.11, 130.86, 130.52, 130.25, 128.95, 128.57, 128.38, 128.33, 128.30, 128.25, 127.25, 124.19, 58.06, 57.60, 57.56; **HRMS (ESI)**: calcd. for $C_{25}H_{25}N_3OP(M+H)^+$: 414.1730, found: 414.1729.

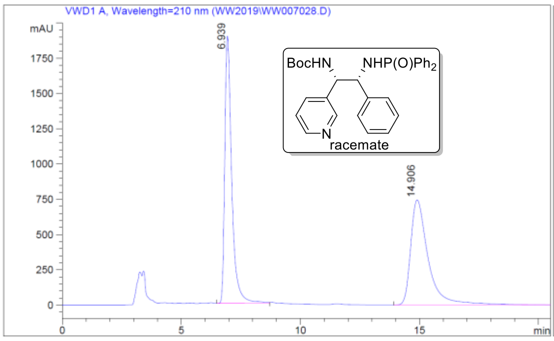

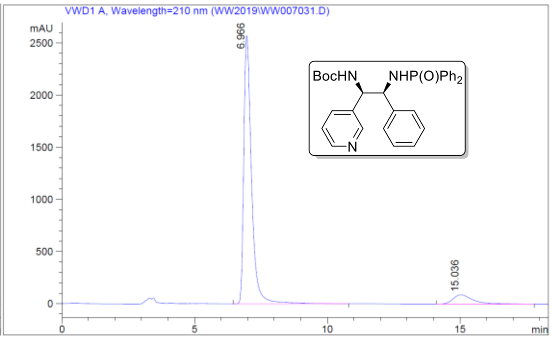

Meas.RT min	Area mAU*sec	Height mAU	Area %	Width min	Symm.
6.94	38324.3	1897.1	50.1	0.337	0.574
14.91	38183.6	744.9	49.9	0.761	0.524

Meas.RT min	Area mAU*sec	Height mAU	Area %	Width min	Symm.
6.97	52234.5	2568.2	91.9	0.307	0.557
15.04	4629.1	89.0	8.1	0.776	0.597

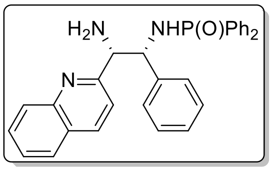

N-((1*R*,2*R*)-2-amino-1-phenyl-2-(quinolin-2-yl)ethyl)-P,P-diphenylphosphinic amide (*syn*-8r): white solid (41.1 mg, 89%); m.p. = 148-149 ^oC; R_f = 0.24 (CH_2Cl_2/MeOH = 25:1); the enantiomeric excess was determined to be 63% by HPLC analysis after being converted to the corresponding *N*-Boc derivatives. **HPLC**: Daicel Chirapak IA-H column (hexane/isopropanol = 70/30, flow rate 1.0 mL/min, T = 30°C), UV 210 nm, t_R(major) 15.34 min, t_R(minor) 7.61 min; $[\alpha]_D^{25}$ = +252.0 (c = 0.13, MeOH); **^{1}H NMR (600 MHz, CDCl$_3$)** δ 7.95 – 7.89 (m, 2H), 7.86 – 7.81 (m, 3H), 7.75 (d, *J* = 8.0 Hz, 1H), 7.72 – 7.64 (m, 3H), 7.52 – 7.49 (m, 1H), 7.52 – 7.41 (m, 2H), 7.37 (t, *J* = 7.0 Hz, 1H), 7.25 – 7.21 (m, 2H), 7.13 – 7.08 (m, 3H), 6.97 –6.90 (m, 3H), 5.70 – 5.61 (m, 1H), 4.70 (td, *J* = 11.1, 4.2 Hz, 1H), 4.54 (d, *J* = 4.0 Hz, 1H); **^{13}C NMR (151 MHz, CDCl$_3$)** δ 160.97, 147.16, 140.58, 140.55, 136.37, 133.76, 132.91, 132.64, 132.58, 132.30, 131.93, 131.92, 131.85, 131.80, 131.78, 131.68, 131.66, 131.63, 131.56, 131.41, 129.60, 129.01, 128.58, 128.55, 128.49, 128.47, 128.26, 128.17, 128.00, 127.64, 127.41, 127.06, 126.89, 126.44, 121.54, 61.81, 61.79, 60.58; **HRMS (ESI)**: calcd. for $C_{26}H_{27}N_3OP(M+H)^+$: 464.1886, found: 428.1872.

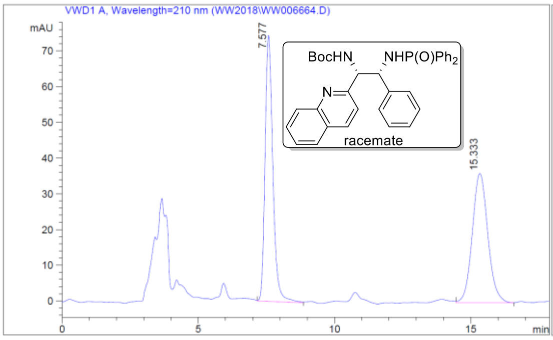

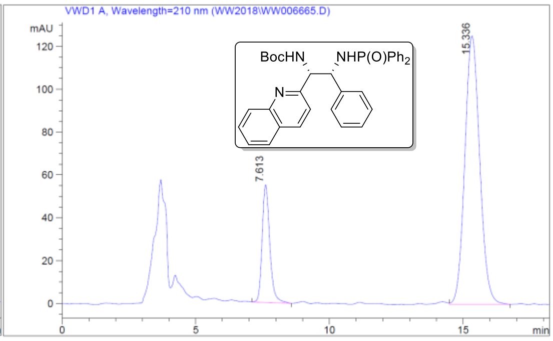

Meas.RT min	Area mAU*sec	Height mAU	Area %	Width min	Symm.
7.58	1480.8	74.4	50.8	0.308	0.730
15.33	1433.5	36.2	49.2	0.619	0.912

Meas.RT min	Area mAU*sec	Height mAU	Area %	Width min	Symm.
7.61	1092.8	54.9	18.4	0.302	0.765
15.34	4850.8	125.2	81.6	0.603	0.892

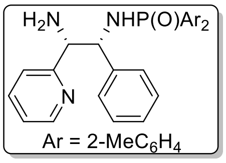

N-((1*R*,2*R*)-2-amino-1-phenyl-2-(pyridin-2-yl)ethyl)-P,P-di-*o*-tolylphosphinic amide(*syn*-8s): white solid (36.7 mg, 83%); m.p. = 76-77 ^oC; R_f = 0.29 (CH_2Cl_2/MeOH = 25:1); the enantiomeric excess was determined to be

85% by HPLC analysis after being converted to the corresponding *N*-Boc derivatives. **HPLC**: Daicel Chirapak AD-H column (hexane/isopropanol = 80/20, flow rate 1.0 mL/min, T = 30°C), UV 210 nm, t_R(major) 9.85 min, t_R(minor) 7.93 min; $[\alpha]_D^{25}$ = +110.4 (c = 0.53, MeOH); **^{1}H NMR (600 MHz, $CDCl_3$)** δ 8.41 (d, *J* = 4.1 Hz, 1H), 7.71 (dd, *J* = 13.5, 7.6 Hz, 1H), 7.63 (dd, *J* = 13.4, 7.7 Hz, 1H), 7.47 (t, *J* = 7.6 Hz, 1H), 7.38 (t, *J* = 7.4 Hz, 1H), 7.29 – 7.17 (m, 3H), 7.14 – 7.08 (m, 4H), 7.08 – 7.04 (m, 1H), 7.00 (t, *J* = 7.1 Hz, 1H), 6.93 (d, *J* = 7.7 Hz, 1H), 6.87 (d, *J* = 6.6 Hz, 2H), 5.33 – 5.25 (m, 1H), 4.72 (td, *J* = 10.6, 4.4 Hz, 1H), 4.39 (d, *J* = 4.4 Hz, 1H), 2.45 (s, 3H), 2.21 (s, 3H); **^{13}C NMR (151 MHz, $CDCl_3$)** δ 160.75, 148.63, 142.37, 142.30, 141.70, 141.64, 140.41, 140.37, 136.33, 133.46, 133.39, 132.83, 132.76, 131.88, 131.75, 131.73, 131.68, 131.61, 131.58, 131.56, 131.53, 131.49, 131.45, 131.08, 130.64, 127.87, 127.19, 127.00, 125.49, 125.40, 125.18, 125.09, 123.28, 122.37, 61.65, 61.63, 60.72, 21.52, 21.49, 21.44, 21.41; **HRMS (ESI)**: calcd. for $C_{27}H_{29}N_3OP(M+H)^+$: 442.2043, found: 442.2038.

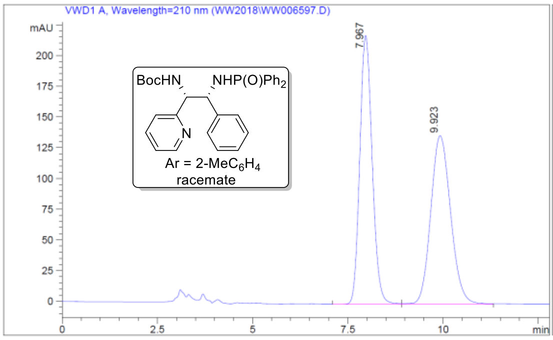

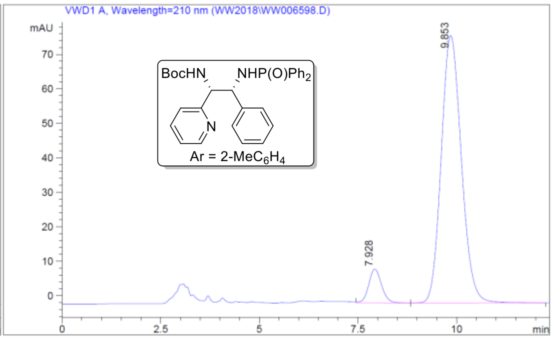

Meas.RT min	Area mAU*sec	Height mAU	Area %	Width min	Symm.
7.97	4934.4	218.4	49.8	0.351	0.838
9.92	4976.6	137.0	50.2	0.563	0.852

Meas.RT min	Area mAU*sec	Height mAU	Area %	Width min	Symm.
7.93	222.4	9.8	7.5	0.352	0.870
9.85	2746.8	77.6	92.5	0.547	0.845

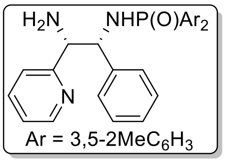

N-((1*R*,2*R*)-2-amino-1-phenyl-2-(pyridin-2-yl)ethyl)-P,P-bis(3,5-dimethylphenyl)phosphinic amide(*syn*-8t): white solid (39.4 mg, 84%); m.p. = 97-98 ^{o}C; R_f = 0.26 (CH_2Cl_2/MeOH = 25:1); the enantiomeric excess was determined to be 91% by HPLC analysis after being converted to the corresponding *N*-Boc derivatives. **HPLC**: Daicel Chirapak AD-H column (hexane/isopropanol = 80/20, flow rate 1.0 mL/min, T = 30°C), UV 210 nm, t_R(major) 6.10 min, t_R(minor) 4.89 min; $[\alpha]_D^{25}$ = +176.3 (c = 0.40, MeOH); **^{1}H NMR (600 MHz, $CDCl_3$)** δ 8.43 (d, *J* = 4.1 Hz, 1H), 7.49 – 7.41 (m, 3H), 7.24 (d, *J* = 12.3 Hz, 2H), 7.17 – 7.09 (m, 5H), 6.96 (s, 1H), 6.91 – 6.86 (m, 3H), 5.27 (dd, *J* = 9.4, 7.0 Hz, 1H), 4.55 (td, *J* = 11.9, 4.4 Hz, 1H), 4.35 (d, *J* = 4.3 Hz, 1H), 2.33 (s, 6H), 2.10 (s, 6H); **^{13}C NMR (151 MHz, $CDCl_3$)** δ 160.48, 148.68, 140.88, 140.84, 138.15, 138.07, 137.78, 137.69, 136.31, 133.56, 133.49, 133.47, 133.29, 133.27, 132.72, 131.95, 131.08, 130.41, 130.35, 129.22, 129.16, 127.83, 127.05, 126.92, 123.32, 122.41, 61.36, 61.34, 60.62, 21.32, 21.01; **HRMS (ESI)**: calcd. for $C_{29}H_{33}N_3OP(M+H)^+$: 470.2356, found: 470.2345.

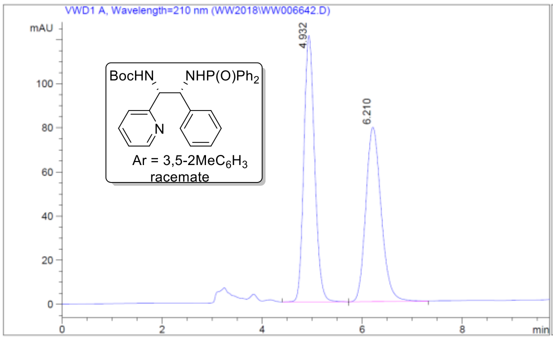

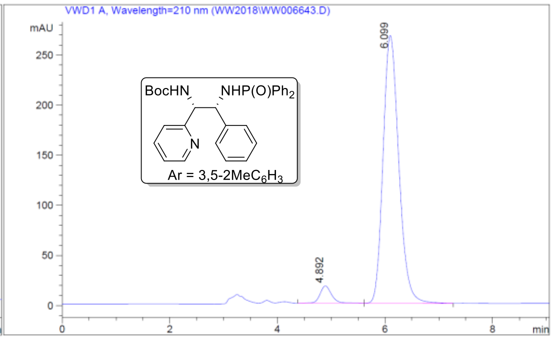

Meas.RT min	Area mAU*sec	Height mAU	Area %	Width min	Symm.
4.93	1806.8	120.5	51.7	0.233	0.830
6.21	1689.6	79.0	48.3	0.331	0.797

Meas.RT min	Area mAU*sec	Height mAU	Area %	Width min	Symm.
4.89	261.6	17.2	4.6	0.236	0.791
6.10	5432.4	267.0	95.4	0.313	0.787

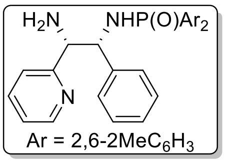

N-((1*R*,2*R*)-2-amino-1-phenyl-2-(pyridin-2-yl)ethyl)-P,P-bis(2,6-dimethylphenyl)phosphinic amide(*syn*-8u): white solid (34.6 mg, 74%); m.p. = 85-86 ^{o}C; R_f = 0.33 (CH_2Cl_2/MeOH = 25:1); the enantiomeric excess was determined to be 77% by HPLC analysis after being converted to the corresponding *N*-Boc derivatives. **HPLC**: Daicel Chirapak AD-H column (hexane/isopropanol = 90/10, flow rate 0.8 mL/min, T = 30°C), UV 210 nm, t_R(major) 12.80 min, t_R(minor) 11.50 min; $[\alpha]_D^{25}$ = +121.1 (c = 0.41, MeOH); **^{1}H NMR (600 MHz, $CDCl_3$)** δ 8.48 (d, *J* = 3.9 Hz, 1H), 7.46 (t, *J* = 6.9 Hz, 1H), 7.18 (t, *J* = 7.3 Hz, 1H), 7.15 – 7.12 (m, 1H), 7.10 (t, *J* = 7.5 Hz, 1H), 7.04 (t, *J* = 7.2 Hz, 1H), 7.02 – 6.95 (m, 4H), 6.83 (dd, *J* = 7.2, 3.9 Hz, 2H), 6.80 (d, *J* = 7.6 Hz, 1H), 6.61 (d, *J* = 7.4 Hz, 2H), 5.72 (d, *J* = 5.7 Hz, 1H), 4.50 – 4.41 (m, 1H), 4.23 (d, *J* = 4.1 Hz, 1H), 2.40 (s, 6H), 2.25 (s, 6H); **^{13}C NMR (151 MHz, $CDCl_3$)** δ 160.86, 148.69, 142.02, 141.95, 141.10, 141.03, 140.53, 140.49, 136.33, 133.75, 132.94, 132.65, 131.84, 130.44, 130.42, 130.06, 129.98, 129.87, 129.79, 127.50, 127.21, 126.76, 123.49, 122.49, 62.79, 61.42, 61.40, 23.04, 23.02, 22.89, 22.86; **HRMS (ESI)**: calcd. for $C_{29}H_{33}N_3OP(M+H)^+$: 470.2356, found: 470.2351.

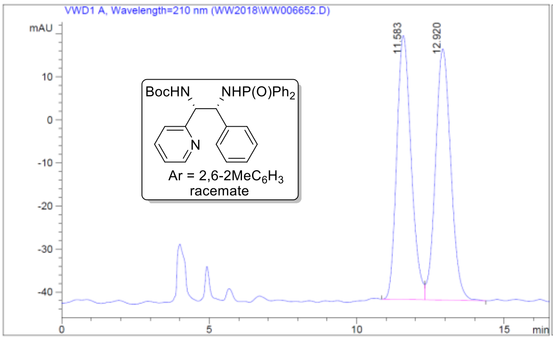

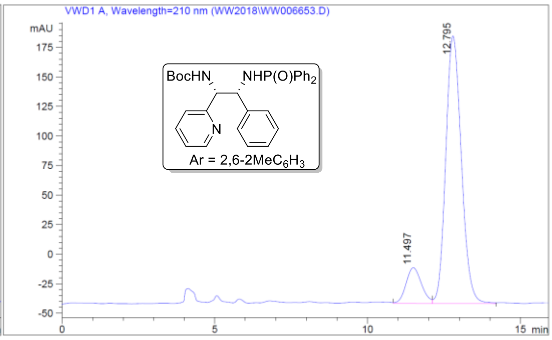

Meas.RT min	Area mAU*sec	Height mAU	Area %	Width min	Symm.
11.58	2077.0	61.3	49.6	0.529	0.796
12.92	2111.5	58.3	50.4	0.562	0.868

Meas.RT min	Area mAU*sec	Height mAU	Area %	Width min	Symm.
11.50	1059.2	30.2	11.7	0.543	0.853
12.80	8019.6	226.3	88.3	0.559	0.820

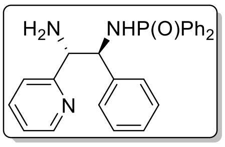

N-((1*S*,2*R*)-2-amino-1-phenyl-2-(pyridin-2-yl)ethyl)-P,P-diphenylphosphinic amide (*anti*-8a): white solid (29.3 mg, 71%); m.p. = 181-182 ^{o}C; R_f = 0.22 (CH_2Cl_2/MeOH = 25:1); the enantiomeric excess was determined to

be 92% by HPLC analysis after being converted to the corresponding *N*-Boc derivatives. **HPLC**: Daicel Chirapak IA-H column (hexane/isopropanol = 70/30, flow rate 1.0 mL/min, T = 30°C), UV 210 nm, t_R(major) 10.39 min, t_R(minor) 18.06 min; $[\alpha]_D^{25}$ = -38.1 (c = 0.21, MeOH); **^{1}H NMR (600 MHz, CD_3OD)** δ 8.46 (d, *J* = 4.3 Hz, 1H), 7.69 (dd, *J* = 12.1, 7.4 Hz, 2H), 7.63 (dd, *J* = 12.2, 7.5 Hz, 2H), 7.57 – 7.53 (m, 1H), 7.52 – 7.43 (m, 4H), 7.34 (td, *J* = 7.7, 3.2 Hz, 2H), 7.18 (dd, *J* = 6.9, 5.2 Hz, 1H), 7.15 – 7.11 (m, 3H), 6.99 – 6.94 (m, 2H), 6.91 (d, *J* = 7.7 Hz, 1H), 4.35 – 4.30 (m, 1H), 4.26 (d, *J* = 8.2 Hz, 1H); **^{13}C NMR (151 MHz, CD_3OD)** δ 158.05, 148.68, 140.57, 140.54, 136.50, 132.25, 132.18, 132.07, 132.05, 132.00, 131.97, 131.95, 131.51, 131.44, 131.37, 131.13, 130.50, 128.41, 128.32, 128.21, 128.13, 127.97, 127.11, 126.86, 123.57, 122.77, 62.08, 62.05, 61.86; **HRMS (ESI)**: calcd. for $C_{25}H_{25}N_3OP(M+H)^+$: 414.1730, found: 414.1728.

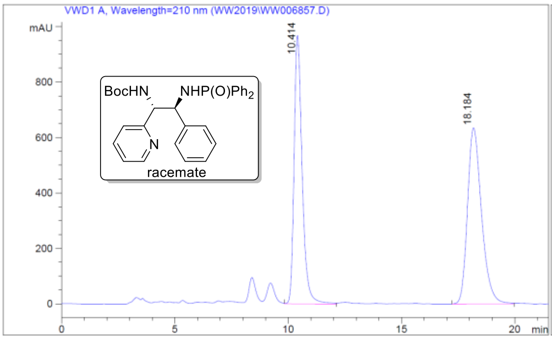

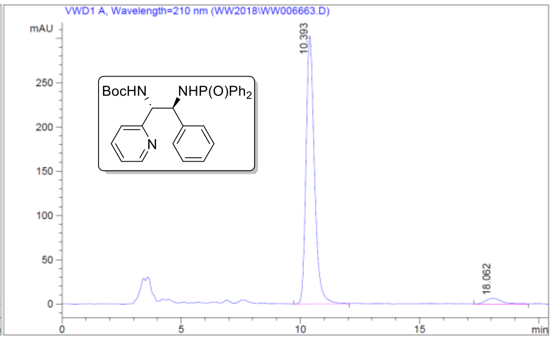

Meas.RT min	Area mAU*sec	Height mAU	Area %	Width min	Symm.
10.41	23871.5	968.3	46.7	0.411	0.684
18.18	27288.0	635.0	53.3	0.716	0.747

Meas.RT min	Area mAU*sec	Height mAU	Area %	Width min	Symm.
10.39	7405.7	302.6	96.2	0.373	0.726
18.06	292.4	6.4	3.8	0.701	0.703

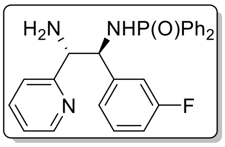

N-((1*S*,2*R*)-2-amino-1-(3-fluorophenyl)-2-(pyridin-2-yl)ethyl)-P,P-diphenylphosphinic amide (*anti*-8b): white solid (24.8 mg, 58%); m.p. = 69-70 ^{o}C; R_f = 0.24 (CH_2Cl_2/MeOH = 25:1); the enantiomeric excess was determined to be 93% by HPLC analysis after being converted to the corresponding *N*-Boc derivatives. **HPLC**: Daicel Chirapak IA-H column (hexane/isopropanol = 70/30, flow rate 1.0 mL/min, T = 30°C), UV 210 nm, t_R(major) 10.49 min, t_R(minor) 14.47 min; $[\alpha]_D^{25}$ = +7.0 (c = 0.30, MeOH); **^{1}H NMR (600 MHz, CD_3OD)** δ 8.47 (d, *J* = 4.3 Hz, 1H), 7.68 (dd, *J* = 12.2, 7.5 Hz, 2H), 7.64 (dd, *J* = 12.2, 7.7 Hz, 2H), 7.56 (t, *J* = 7.5 Hz, 2H), 7.51 – 7.44 (m, 3H), 7.36 (td, *J* = 7.6, 3.1 Hz, 2H), 7.21 (dd, *J* = 7.0, 5.3 Hz, 1H), 7.12 (dd, *J* = 13.9, 7.8 Hz, 1H), 6.99 (d, *J* = 7.8 Hz, 1H), 6.88 – 6.84 (m, 1H), 6.78 (d, *J* = 9.9 Hz, 1H), 6.74 (d, *J* = 7.6 Hz, 1H), 4.38 – 4.33 (m, 1H), 4.22 (d, *J* = 8.0 Hz, 1H); **^{13}C NMR (151 MHz, CD_3OD)** δ 163.46, 161.84, 158.30, 148.75, 143.62, 136.66, 132.15, 132.12, 132.10, 132.08, 132.02, 132.00, 131.52, 131.46, 130.96, 130.54, 129.69, 129.63, 128.45, 128.36, 128.23, 128.14, 123.42, 122.86, 122.79, 122.77, 113.81, 113.75, 113.66, 113.60, 62.01, 61.98, 61.46; **HRMS (ESI)**: calcd. for $C_{25}H_{24}FN_3OP(M+H)^+$: 432.1636, found: 432.1631.

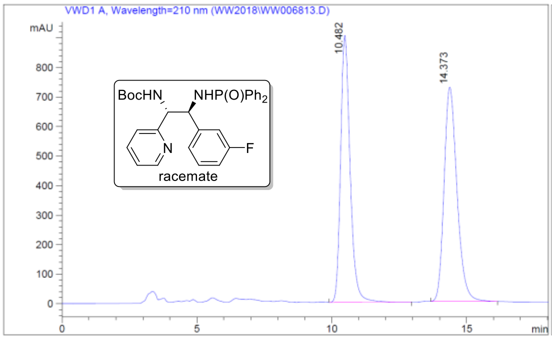

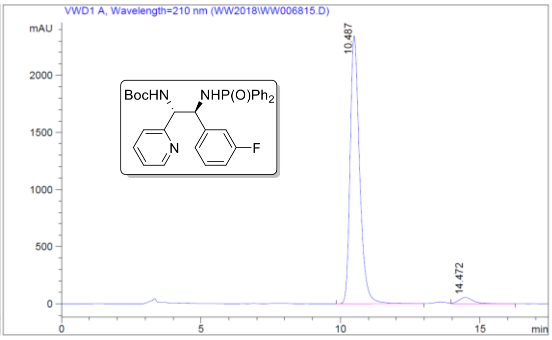

Meas.RT min	Area mAU*sec	Height mAU	Area %	Width min	Symm.
10.48	22113.2	903.2	47.8	0.379	0.727
14.37	24166.3	726.3	52.2	0.555	0.749

Meas.RT min	Area mAU*sec	Height mAU	Area %	Width min	Symm.
10.49	55370.4	2344.0	96.7	0.363	0.673
14.47	1860.9	54.3	3.3	0.522	0.806

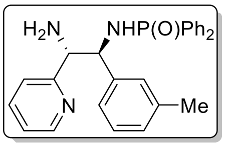

N-((1*S*,2*R*)-2-amino-2-(pyridin-2-yl)-1-(m-tolyl)ethyl)-P,P-diphenylphosphinic amide (*anti*-8c): white solid (35.3 mg, 83%); m.p. = 83-84 °C; R_f = 0.19 (CH_2Cl_2/MeOH = 25:1); the enantiomeric excess was determined to be 96% by HPLC analysis after being converted to the corresponding *N*-Boc derivatives. **HPLC**: Daicel Chirapak IA-H column (hexane/isopropanol = 80/20, flow rate 1.0 mL/min, T = 30°C), UV 210 nm, t_R(major) 13.20 min, t_R(minor) 20.05 min; $[\alpha]_D^{25}$ = -25.3 (c = 0.24, MeOH); **^{1}H NMR (600 MHz, $CDCl_3$)** δ 8.55 (d, *J* = 4.1 Hz, 1H), 7.64 (dd, *J* = 11.9, 7.5 Hz, 2H), 7.56 (t, *J* = 7.0 Hz, 1H), 7.51 (dd, *J* = 11.9, 7.5 Hz, 2H), 7.41 (dd, *J* = 13.0, 6.3 Hz, 2H), 7.32 – 7.27 (m, 4H), 7.20 – 7.16 (m, 1H), 7.12 – 7.06 (m, 2H), 6.98 (t, *J* = 6.0 Hz, 2H), 6.90 (s, 1H), 4.93 (t, *J* = 8.5 Hz, 1H), 4.45 – 4.39 (m, 2H), 2.24 (s, 3H); **^{13}C NMR (151 MHz, $CDCl_3$)** δ 160.14, 148.86, 141.08, 137.77, 136.26, 132.45, 132.38, 131.87, 131.80, 131.61, 131.59, 128.36, 128.28, 128.22, 128.15, 128.14, 128.04, 127.90, 124.20, 122.53, 122.46, 61.99, 61.95, 60.84, 21.37; **HRMS (ESI)**: calcd. for $C_{26}H_{27}N_3OP(M+H)^+$: 428.1886, found: 428.1886.

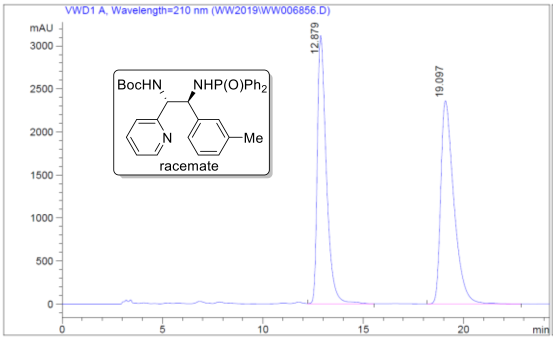

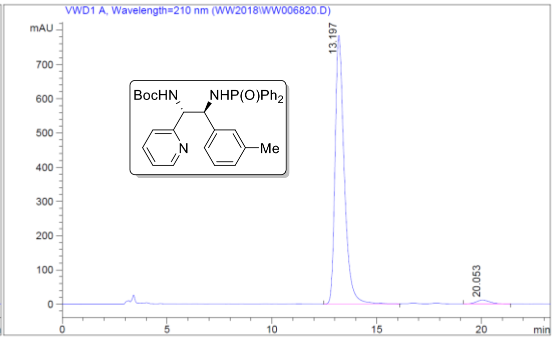

Meas.RT min	Area mAU*sec	Height mAU	Area %	Width min	Symm.
12.88	97582.8	3114.8	47.6	0.470	0.532
19.10	107594.0	2360.4	52.4	0.683	0.515

Meas.RT min	Area mAU*sec	Height mAU	Area %	Width min	Symm.
13.20	24040.2	783.9	97.9	0.463	0.631
20.05	510.5	11.1	2.1	0.700	0.811

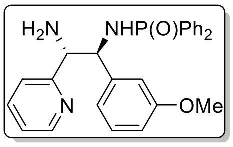

N-((1*S*,2*R*)-2-amino-1-(3-methoxyphenyl)-2-(pyridin-2-yl)ethyl)-P,P-diphenylphosphinic amide (*anti*-8d): white solid (27.9 mg, 63%); m.p. = 138-139 °C; R_f = 0.15 (CH_2Cl_2/MeOH = 25:1); the enantiomeric excess was determined to be 93% by HPLC analysis after being converted to the

corresponding *N*-Boc derivatives. **HPLC**: Daicel Chirapak IA-H column (hexane/isopropanol = 70/30, flow rate 1.0 mL/min, T = 30°C), UV 210 nm, t_R(major) 9.50 min, t_R(minor) 12.51 min; $[\alpha]_D^{25}$ = -31.0 (c = 0.20, MeOH); **^{1}H NMR (600 MHz, CD_3OD)** δ 8.48 (d, *J* = 4.2 Hz, 1H), 7.71 (dd, *J* = 12.1, 7.6 Hz, 2H), 7.64 (dd, *J* = 12.2, 7.6 Hz, 2H), 7.59 – 7.46 (m, 5H), 7.38 – 7.33 (m, 2H), 7.22 – 7.18 (m, 1H), 7.05 (t, *J* = 7.9 Hz, 1H), 6.93 (d, *J* = 7.8 Hz, 1H), 6.69 (dd, *J* = 8.1, 1.9 Hz, 1H), 6.55 (d, *J* = 7.4 Hz, 1H), 6.50 (s, 1H), 4.35 – 4.29 (m, 1H), 3.63 (s, 3H); **^{13}C NMR (151 MHz, CD_3OD)** δ 159.67, 157.29, 148.76, 141.65, 136.58, 132.27, 132.21, 132.16, 132.15, 132.03, 132.02, 131.86, 131.48, 131.41, 131.19, 131.00, 130.32, 129.09, 128.45, 128.36, 128.23, 128.14, 123.71, 122.97, 119.04, 112.99, 112.41, 61.76, 61.73, 61.51, 54.24; **HRMS (ESI)**: calcd. for $C_{26}H_{27}N_3O_2P(M+H)^+$: 444.1835, found: 444.1834.

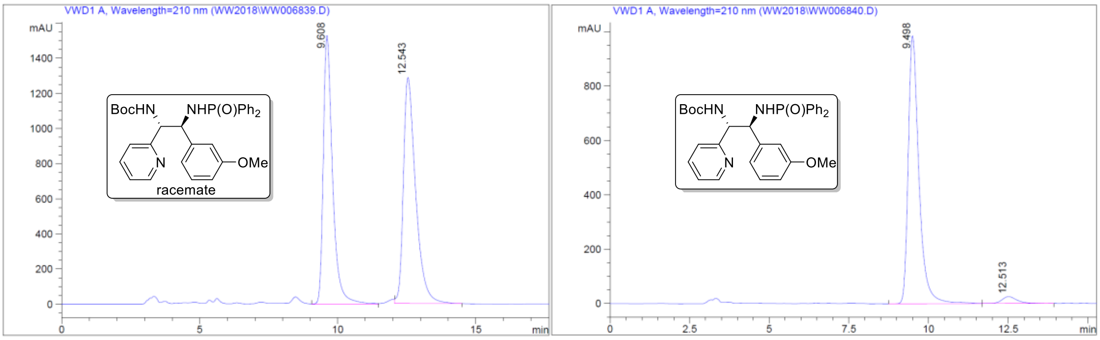

Meas.RT min	Area mAU*sec	Height mAU	Area %	Width min	Symm.
9.61	35887.0	1530.7	47.5	0.391	0.581
12.54	39660.6	1287.5	52.5	0.513	0.572

Meas.RT min	Area mAU*sec	Height mAU	Area %	Width min	Symm.
9.50	23083.9	984.8	96.6	0.349	0.613
12.51	806.5	24.7	3.4	0.544	0.756

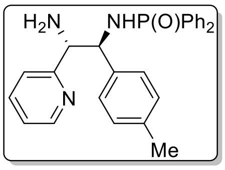

N-((1*S*,2*R*)-2-amino-2-(pyridin-2-yl)-1-(p-tolyl)ethyl)-P,P-diphenylphosphinic amide (*anti*-8e): white solid (22.4 mg, 52%); m.p. = 113-114 ^{o}C; R_f = 0.22 (CH_2Cl_2/MeOH = 25:1); the enantiomeric excess was determined to be 92% by HPLC analysis after being converted to the corresponding *N*-Boc derivatives. **HPLC**: Daicel Chirapak IA-H column (hexane/isopropanol = 70/30, flow rate 0.8 mL/min, T = 30°C), UV 210 nm, t_R(major) 14.18 min, t_R(minor) 35.22 min; $[\alpha]_D^{25}$ = -33.0 (c = 0.20, MeOH); **^{1}H NMR (400 MHz, CD_3OD)** δ 8.47 (d, *J* = 4.5 Hz, 1H), 7.74 – 7.68 (m, 2H), 7.66 – 7.60 (m, 2H), 7.58 – 7.48 (m, 5H), 7.35 (td, *J* = 7.5, 3.2 Hz, 2H), 7.19 (dd, *J* = 7.4, 5.1 Hz, 1H), 6.97 – 6.80 (m, 5H), 4.35 – 4.31 (m, 2H), 2.23 (s, 3H); **^{13}C NMR (101 MHz, CD_3OD)** δ 156.86, 148.79, 137.16, 136.55, 132.32, 132.22, 132.18, 132.16, 132.05, 132.02, 131.49, 131.39, 128.65, 128.46, 128.34, 128.28, 128.15, 126.79, 123.82, 123.01, 61.71, 61.67, 61.07 19.67; **HRMS (ESI)**: calcd. for $C_{26}H_{27}N_3OP(M+H)^+$: 428.1886, found: 428.1885.

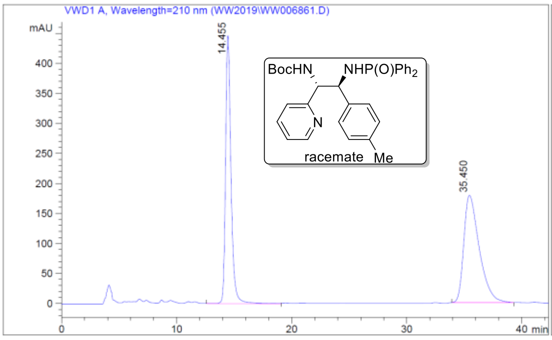

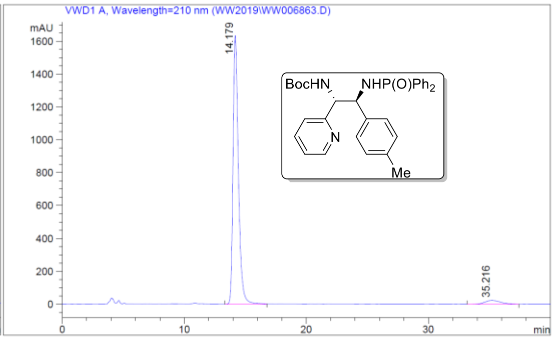

Meas.RT min	Area mAU*sec	Height mAU	Area %	Width min	Symm.
14.46	14772.1	447.1	47.0	0.551	0.684
35.45	16652.5	178.7	53.0	1.553	0.558

Meas.RT min	Area mAU*sec	Height mAU	Area %	Width min	Symm.
14.18	50026.9	1634.2	96.0	0.456	0.644
35.22	2083.4	23.3	4.0	1.272	0.772

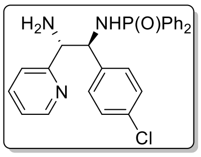

N-((1*S*,2*R*)-2-amino-1-(4-chlorophenyl)-2-(pyridin-2-yl)ethyl)-P,P-diphenylphosphinic amide (*anti*-8f): white solid (27.8 mg, 62%); m.p. = 99-100 ^{o}C; R_f = 0.21 (CH_2Cl_2/MeOH = 25:1); the enantiomeric excess was determined to be 92% by HPLC analysis after being converted to the corresponding *N*-Boc derivatives. **HPLC**: Daicel Chirapak IA-H column (hexane/isopropanol = 70/30, flow rate 1.0 mL/min, T = 30°C), UV 210 nm, t_R(major) 15.40 min, t_R(minor) 46.90 min; $[\alpha]_D^{25}$ = +13.5 (c = 0.40, MeOH); **^{1}H NMR (400 MHz, CD_3OD)** δ 8.46 (d, *J* = 4.5 Hz, 1H), 7.73 – 7.60 (m, 4H), 7.56 (td, *J* = 7.6, 1.4 Hz, 2H), 7.50 – 7.43 (m, 3H), 7.36 (td, *J* = 7.7, 3.3 Hz, 2H), 7.20 (dd, *J* = 7.0, 5.0 Hz, 1H), 7.12 (d, *J* = 8.4 Hz, 2H), 7.01 – 6.91 (m, 3H), 4.37 – 4.30 (m, 1H), 4.23 (d, *J* = 8.1 Hz, 1H); **^{13}C NMR (101 MHz, CD_3OD)** δ 158.15, 148.79, 139.56, 136.71, 132.81, 132.18, 132.15, 132.12, 132.09, 132.03, 132.00, 131.56, 131.46, 130.70, 130.27, 128.53, 128.48, 128.36, 128.29, 128.16, 127.98, 123.53, 122.90, 61.98, 61.93, 61.23; **HRMS (ESI)**: calcd. for $C_{25}H_{24}ClN_3OP(M+H)^+$: 448.1340, found: 448.1339.

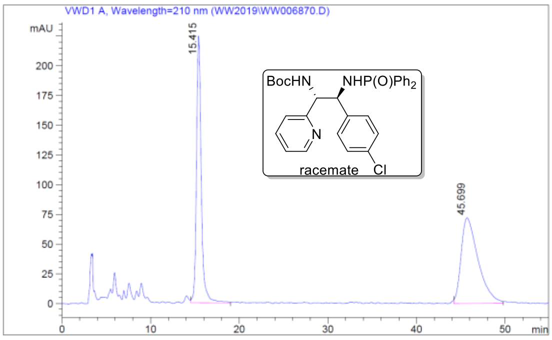

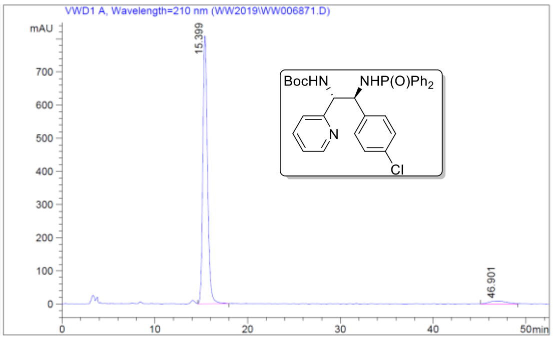

Meas.RT min	Area mAU*sec	Height mAU	Area %	Width min	Symm.
15.42	8427.3	224.6	46.8	0.625	0.767
45.70	9599.0	72.1	53.2	2.219	0.528

Meas.RT min	Area mAU*sec	Height mAU	Area %	Width min	Symm.
15.40	28290.4	808.3	95.8	0.536	0.759
46.90	1238.7	9.2	4.2	1.629	0.794

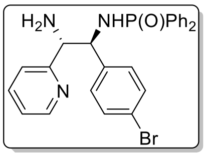

N-((1*S*,2*R*)-2-amino-1-(4-bromophenyl)-2-(pyridin-2-yl)ethyl)-P,P-diphenylphosphinic amide (*anti*-8g): white solid (31.0 mg, 63%); m.p. = 94-95 ^{o}C; R_f = 0.21 (CH_2Cl_2/MeOH = 25:1); the enantiomeric excess was determined to be 92% by HPLC analysis after being converted to the

corresponding *N*-Boc derivatives. **HPLC**: Daicel Chirapak IA-H column (hexane/isopropanol = 70/30, flow rate 1.0 mL/min, T = 30°C), UV 210 nm, t_R(major) 14.92 min, t_R(minor) 44.29 min; $[\alpha]_D^{25}$ = +14.6 (c = 0.39, MeOH); **^{1}H NMR (600 MHz, CD_3OD)** δ 8.47 (d, *J* = 4.2 Hz, 1H), 7.70 (dd, *J* = 12.2, 7.5 Hz, 2H), 7.64 (dd, *J* = 12.1, 7.7 Hz, 2H), 7.56 (t, *J* = 7.3 Hz, 2H), 7.52 – 7.43 (m, 3H), 7.36 (td, *J* = 7.6, 3.0 Hz, 2H), 7.27 (d, *J* = 8.3 Hz, 2H), 7.21 (dd, *J* = 6.9, 5.3 Hz, 1H), 6.99 (d, *J* = 7.7 Hz, 1H), 6.90 (d, *J* = 8.2 Hz, 2H), 4.34 (t, *J* = 9.1 Hz, 1H), 4.26 (d, *J* = 8.1 Hz, 1H); **^{13}C NMR (151 MHz, CD_3OD)** δ 158.02, 148.82, 139.96, 139.94, 136.70, 132.16, 132.14, 132.12, 132.09, 132.01, 131.99, 131.81, 131.54, 131.47, 131.40, 131.06, 131.00, 130.95, 130.53, 128.90, 128.46, 128.38, 128.27, 128.18, 123.54, 122.93, 120.87, 61.84, 61.80, 61.18; **HRMS (ESI)**: calcd. for $C_{25}H_{24}BrN_3OP(M+H)^+$: 492.0835, found: 492.0834.

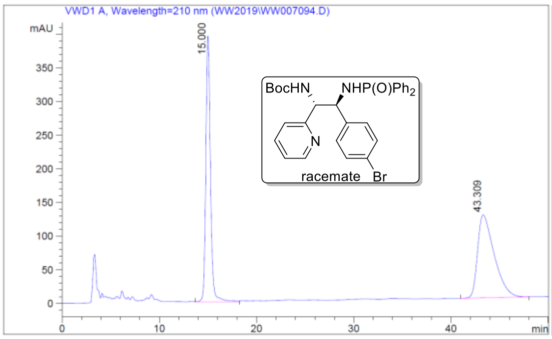

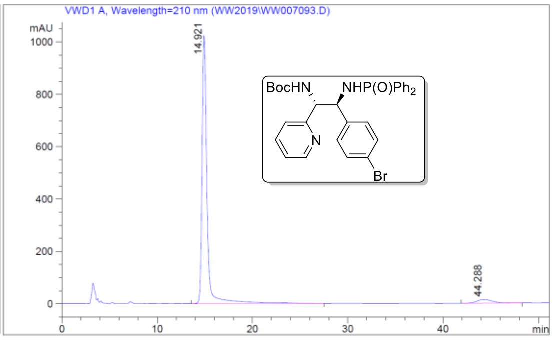

Meas.RT min	Area mAU*sec	Height mAU	Area %	Width min	Symm.
15.00	13109.6	395.4	47.3	0.553	0.811
43.31	14587.5	123.3	52.7	1.972	0.491

Meas.RT min	Area mAU*sec	Height mAU	Area %	Width min	Symm.
14.92	37327.0	1023.4	95.8	0.542	0.598
44.29	1643.6	14.2	4.2	1.733	0.711

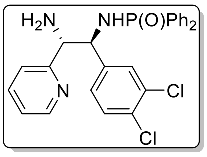

N-((1*S*,2*R*)-2-amino-1-(3,4-dichlorophenyl)-2-(pyridin-2-yl)ethyl)-P,P-diphenylphosphinic amide (*anti*-8h): white solid (33.3 mg, 69%); m.p. = 82-83 ^{o}C; R_f = 0.16 (CH_2Cl_2/MeOH = 25:1); the enantiomeric excess was determined to be 94% by HPLC analysis after being converted to the corresponding *N*-Boc derivatives. **HPLC**: Daicel Chirapak IC-H column (hexane/isopropanol = 80/20, flow rate 0.8 mL/min, T = 30°C), UV 210 nm, t_R(major) 16.21 min, t_R(minor) 13.86 min; $[\alpha]_D^{25}$ = -12.9 (c = 0.28, MeOH); **^{1}H NMR (600 MHz, CD_3OD)** δ 8.51 (d, *J* = 4.4 Hz, 1H), 7.76 – 7.70 (m, 2H), 7.68 – 7.58 (m, 4H), 7.54 – 7.48 (m, 3H), 7.40 (td, *J* = 7.5, 3.0 Hz, 2H), 7.29 – 7.24 (m, 2H), 7.12 (d, *J* = 1.5 Hz, 1H), 7.06 (d, *J* = 7.8 Hz, 1H), 6.93 (d, *J* = 8.3 Hz, 1H), 4.40 (t, *J* = 9.0 Hz, 1H), 4.30 (d, *J* = 7.8 Hz, 1H); **^{13}C NMR (151 MHz, CD_3OD)** δ 157.66, 148.94, 141.33, 136.80, 132.21, 132.19, 132.05, 132.04, 131.99, 131.72, 131.59, 131.49, 131.43, 131.31, 130.88, 130.73, 130.45, 129.95, 129.24, 128.47, 128.39, 128.24, 128.15, 126.80, 123.49, 123.08, 61.59, 61.55, 60.52; **HRMS (ESI)**: calcd. for $C_{25}H_{23}Cl_2N_3OP(M+H)^+$: 482.0950, found: 482.0948.

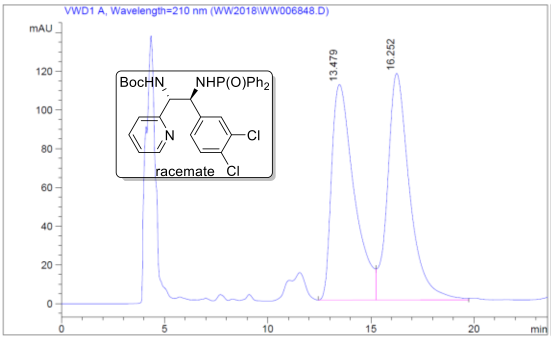

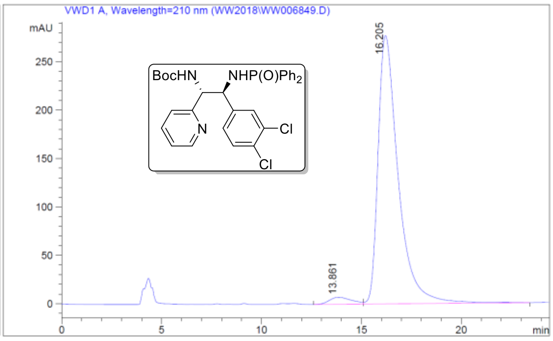

Meas.RT min	Area mAU*sec	Height mAU	Area %	Width min	Symm.
13.48	8467.3	111.2	49.3	1.150	0.495
16.25	8715.9	116.9	50.7	1.103	0.654

Meas.RT min	Area mAU*sec	Height mAU	Area %	Width min	Symm.
13.86	551.2	7.2	2.8	1.162	0.685
16.21	19456.2	277.2	97.2	1.052	0.529

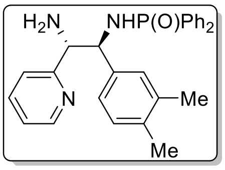

N-((1*S*,2*R*)-2-amino-1-(3,4-dimethylphenyl)-2-(pyridin-2-yl)ethyl)-P,P-diphenylphosphinic amide (*anti*-8i): white solid (21.2 mg, 48%); m.p. = 90-91 ^{o}C; R_f = 0.22 (CH_2Cl_2/MeOH = 25:1); the enantiomeric excess was determined to be 96% by HPLC analysis after being converted to the corresponding *N*-Boc derivatives. **HPLC**: Daicel Chirapak IA-H column (hexane/isopropanol = 80/20, flow rate 0.8 mL/min, T = 30°C), UV 210 nm, t_R(major) 18.32 min, t_R(minor) 31.38 min; $[\alpha]_D^{25}$ = -7.6 (c = 0.33, MeOH); **^{1}H NMR (600 MHz, $CDCl_3$)** δ 8.51 (d, *J* = 4.1 Hz, 1H), 7.64 (dd, *J* = 11.9, 7.5 Hz, 2H), 7.55 (dd, *J* = 11.9, 7.8 Hz, 2H), 7.50 (t, *J* = 7.6 Hz, 1H), 7.43 –7.39 (m, 2H), 7.32 –7.27 (m, 4H), 7.17 – 7.13 (m, 1H), 6.97 (dd, *J* = 13.8, 7.9 Hz, 2H), 6.86 (d, *J* = 7.6 Hz, 1H), 6.83 (s, 1H), 5.16 (t, *J* = 9.1 Hz, 1H), 4.56 (d, *J* = 6.1 Hz, 1H), 4.36 (dd, *J* = 16.2, 9.8 Hz, 1H), 2.18 (s, 3H), 2.13 (s, 3H); **^{13}C NMR (151 MHz, $CDCl_3$)** δ 159.05, 148.83, 138.12, 138.09, 136.38, 136.29, 135.63, 132.56, 132.49, 131.85, 131.78, 131.71, 131.69, 131,66, 131.64, 129.57, 128.45, 128.40, 128.32, 128.26, 128.17, 124.58, 122.97, 122.60, 61.57, 61.54, 60.51, 19.69, 19.38; **HRMS (ESI)**: calcd. for $C_{27}H_{29}N_3OP(M+H)^+$: 442.2043, found: 442.2042.

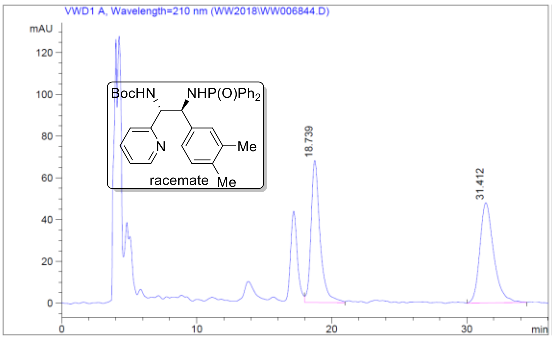

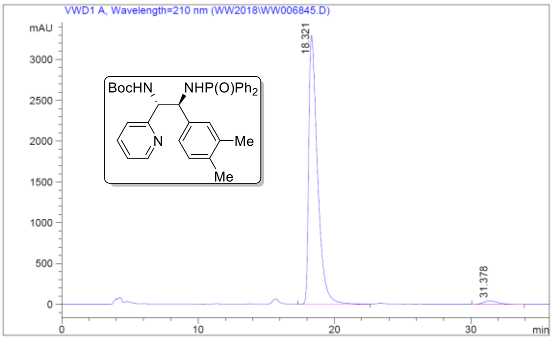

Meas.RT min	Area mAU*sec	Height mAU	Area %	Width min	Symm.
18.74	3175.9	68.0	48.1	0.778	0.691
31.41	3426.4	48.0	51.9	1.072	0.724

Meas.RT min	Area mAU*sec	Height mAU	Area %	Width min	Symm.
18.32	146843.7	3292.5	98.0	0.666	0.430
31.38	3048.9	42.2	2.0	1.098	0.681

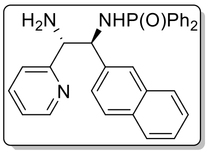

N-((1*S*,2*R*)-2-amino-1-(naphthalen-2-yl)-2-(pyridin-2-yl)ethyl)-P,P-diphenylphosphinic amide (*anti*-8j): white solid (25.1 mg, 54%); m.p. = 118-119 ^{o}C; R_f = 0.23 (CH_2Cl_2/MeOH = 25:1); the enantiomeric excess was

determined to be 96% by HPLC analysis after being converted to the corresponding *N*-Boc derivatives. **HPLC**: Daicel Chirapak IA-H column (hexane/isopropanol = 70/30, flow rate 1.0 mL/min, T = 30°C), UV 210 nm, t_R(major) 11.73 min, t_R(minor) 26.94 min; $[\alpha]_D^{25}$ = -24.0 (c = 0.20, MeOH); **^{1}H NMR (400 MHz, CD_3OD)** δ 8.45 (d, *J* = 4.7 Hz, 1H), 7.75 – 7.59 (m, 7H), 7.56 – 7.51 (m, 1H), 7.46 – 7.38 (m, 6H), 7.32 (s, 1H), 7.28 – 7.19 (m, 3H), 7.15 – 7.10 (m, 1H), 6.92 (d, *J* = 7.8 Hz, 1H), 4.53 – 4.46 (m, 1H), 4.37 (d, *J* = 8.1 Hz, 1H); **^{13}C NMR (101 MHz, CD_3OD)** δ 158.37, 148.69, 138.02, 136.54, 133.05, 132.72, 132.25, 132.18, 132.15, 132.09, 132.06, 131.94, 131.92, 131.60, 131.56, 131.47, 130.88, 130.29, 128.45, 128.33, 128.19, 128.06, 127.79, 127.48, 127.18, 126.10, 125.82, 125.68, 124.49, 123.51, 122.75, 62.15, 62.01, 61.96; **HRMS (ESI)**: calcd. for $C_{29}H_{27}N_3OP(M+H)^+$: 464.1886, found: 464.1885.

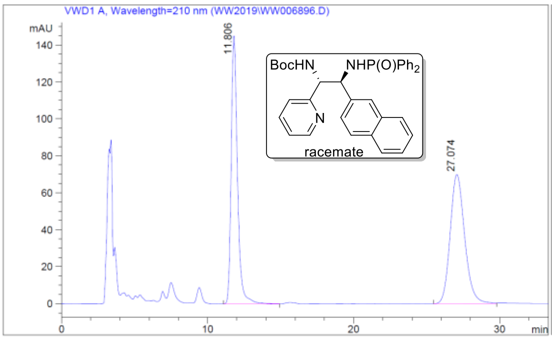

Meas.RT min	Area mAU*sec	Height mAU	Area %	Width min	Symm.
11.81	4341.9	145.0	47.3	0.454	0.701
27.07	4838.1	69.9	52.7	1.153	0.840

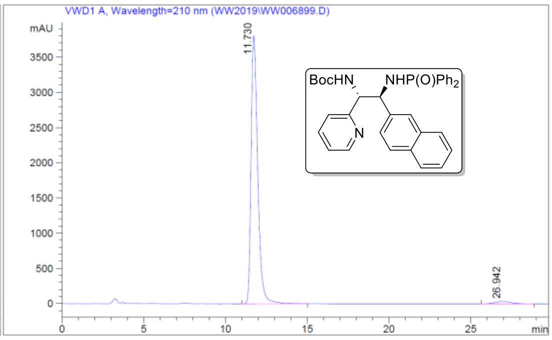

Meas.RT min	Area mAU*sec	Height mAU	Area %	Width min	Symm.
11.73	107314.7	3803.5	97.9	0.428	0.638
26.94	2312.9	34.2	2.1	1.034	0.821

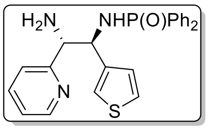

N-((1*S*,2*R*)-2-amino-2-(pyridin-2-yl)-1-(thiophen-3-yl)ethyl)-P,P-diphenyl phosphinic amide (*anti*-8k): white solid (24.7 mg, 59%); m.p. = 96-97 ^{o}C; R_f = 0.31 (CH_2Cl_2/MeOH = 25:1); the enantiomeric excess was determined to be 90% by HPLC analysis after being converted to the corresponding *N*-Boc derivatives. **HPLC**: Daicel Chirapak IA-H column (hexane/isopropanol = 70/30, flow rate 1.0 mL/min, T = 30°C), UV 210 nm, t_R(major) 11.52 min, t_R(minor) 25.88 min; $[\alpha]_D^{25}$ = +37.5 (c = 0.20, MeOH); **^{1}H NMR (600 MHz, CD_3OD)** δ 8.50 (d, *J* = 4.5 Hz, 1H), 7.70 (ddd, *J* = 15.8, 6.9, 4.8 Hz, 4H), 7.61 – 7.51 (m, 3H), 7.49 – 7.46 (m, 2H), 7.42 – 7.39 (m, 2H), 7.27 – 7.23 (m, 2H), 7.04 (d, *J* = 7.8 Hz, 1H), 6.88 (d, *J* = 1.8 Hz, 1H), 6.85 (d, *J* = 4.9 Hz, 1H), 4.53 (t, *J* = 9.1 Hz, 1H), 4.35 (dd, *J* = 7.8, 3.8 Hz, 1H); **^{13}C NMR (151 MHz, CD_3OD)** δ 157.71, 148.71, 141.34, 136.60, 132.19, 132.12, 132.08, 132.01, 131.99, 131.93, 131.87, 131.43, 131.37, 128.40, 128.31, 128.26, 128.18, 125.85, 125.68, 123.44, 122.91, 122.15, 61.57, 61.54, 57.35; **HRMS (ESI)**: calcd. for $C_{23}H_{23}N_3OPS(M+H)^+$: 420.1294, found: 420.1293.

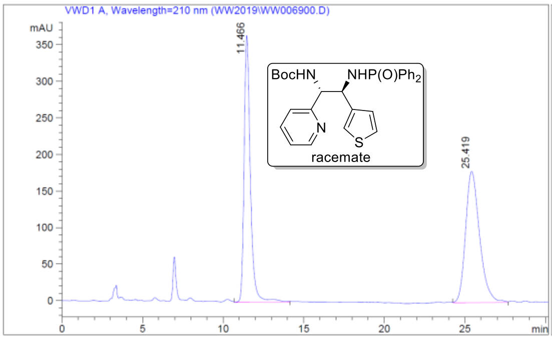

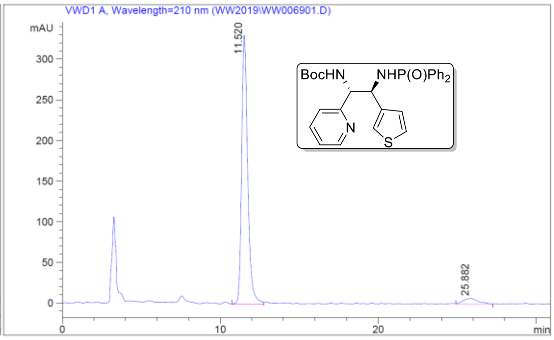

Meas.RT min	Area mAU*sec	Height mAU	Area %	Width min	Symm.
11.47	9887.2	365.1	47.7	0.451	0.664
25.42	10841.8	179.5	52.3	1.007	0.757

Meas.RT min	Area mAU*sec	Height mAU	Area %	Width min	Symm.
11.52	8783.0	330.1	95.0	0.398	0.711
25.88	457.5	6.8	5.0	0.862	1.071

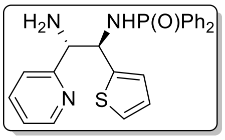

N-((1*R*,2*R*)-2-amino-2-(pyridin-2-yl)-1-(thiophen-2-yl)ethyl)-P,P-diphenylphosphinic amide (*anti*-8l): white solid (29.4 mg, 70%); m.p. = 83-84 ^{o}C; R_f = 0.31 (CH_2Cl_2/MeOH = 25:1); the enantiomeric excess was determined to be 92% by HPLC analysis after being converted to the corresponding *N*-Boc derivatives. **HPLC**: Daicel Chirapak IA-H column (hexane/isopropanol = 70/30, flow rate 1.0 mL/min, T = 30°C), UV 210 nm, t_R(major) 11.04 min, t_R(minor) 16.50 min; $[\alpha]^D_{25}$ = +42.0 (c = 0.30, MeOH); **^{1}H NMR (600 MHz, CDCl$_3$)** δ 8.54 (d, *J* = 3.9 Hz, 1H), 7.70 (dd, *J* = 11.9, 7.6 Hz, 2H), 7.59 – 7.56 (m, 3H), 7.42 (dd, *J* = 14.7, 7.3 Hz, 2H), 7.34 – 7.29 (m, 4H), 7.21 – 7.17 (m, 2H), 7.12 (d, *J* = 4.4 Hz, 1H), 6.83 – 6.79 (m, 2H), 5.10 (t, *J* = 8.6 Hz, 1H), 4.76 (td, *J* = 9.6, 5.1 Hz, 1H), 4.49 (d, *J* = 4.8 Hz, 1H); **^{13}C NMR (151 MHz, CDCl$_3$)** δ 160.16, 148.82, 145.50, 136.47, 132.88, 132.62, 132.42, 132.35, 132.03, 131.86, 131.79, 131.75, 131.68, 131.66, 128.43, 128.34, 128.29, 128.21, 126.60, 125.52, 124.47, 122.53, 122.42, 62.05, 62.02, 57.21; **HRMS (ESI)**: calcd. for $C_{23}H_{23}N_3OPS(M+H)^+$: 420.1294, found: 420.1296.

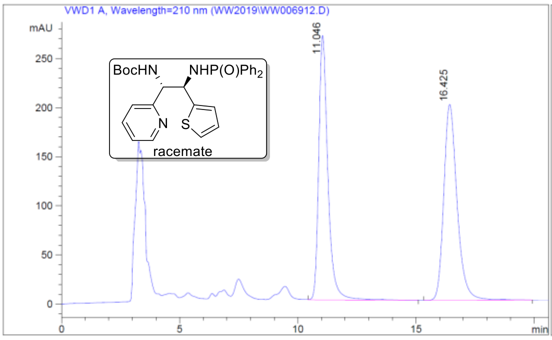

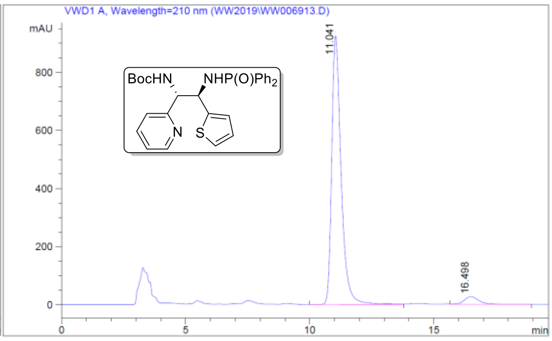

Meas.RT min	Area mAU*sec	Height mAU	Area %	Width min	Symm.
11.05	7257.1	268.9	48.2	0.408	0.661
16.42	7793.6	199.2	51.8	0.596	0.740

Meas.RT min	Area mAU*sec	Height mAU	Area %	Width min	Symm.
11.04	24338.6	923.0	95.8	0.401	0.625
16.50	1072.6	26.3	4.2	0.679	0.832

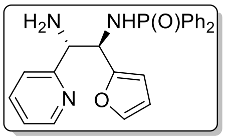

N-((1*R*,2*R*)-2-amino-1-(furan-2-yl)-2-(pyridin-2-yl)ethyl)-P,P-diphenylphosphinic amide (*anti*-8m): white solid (16.0 mg, 40%); m.p. = 96-97 ^{o}C; R_f = 0.29 (CH_2Cl_2/MeOH = 25:1); the enantiomeric excess was determined to be 68% by HPLC analysis after being converted to the corresponding *N*-Boc

derivatives. **HPLC**: Daicel Chirapak IC-H column (hexane/isopropanol = 80/20, flow rate 0.8 mL/min, T = 30°C), UV 210 nm, t_R(major) 29.05 min, t_R(minor) 22.27 min; $[\alpha]_D^{25}$ = +35.0 (c = 0.30, MeOH); **^{1}H NMR (600 MHz, CD_3OD)** δ 8.46 (d, *J* = 4.6 Hz, 1H), 7.70 (dd, *J* = 12.3, 7.4 Hz, 2H), 7.65 – 7.62 (m, 3H), 7.55 – 7.51 (m, 2H), 7.45 – 7.41 (m, 4H), 7.33 (d, *J* = 0.8 Hz, 1H), 7.24 (dd, *J* = 7.2, 5.2 Hz, 1H), 7.14 (d, *J* = 7.8 Hz, 1H), 6.15 (dd, *J* = 2.9, 2.0 Hz, 1H), 5.87 (d, *J* = 3.2 Hz, 1H), 4.46 – 4.40 (m, 2H); **^{13}C NMR (151 MHz, CD_3OD)** δ 158.27, 152.90, 152.88, 148.67, 147.26, 141.82, 136.70, 132.06, 132.03, 132.01, 131.99, 131.97, 131.43, 131.36, 128.38, 128.29, 128.28, 128.19, 122.84, 109.77, 107.51, 59.93, 59.90, 55.35; **HRMS (ESI)**: calcd. for $C_{23}H_{23}N_3O_2P(M+H)^+$: 404.1522, found: 404.1523.

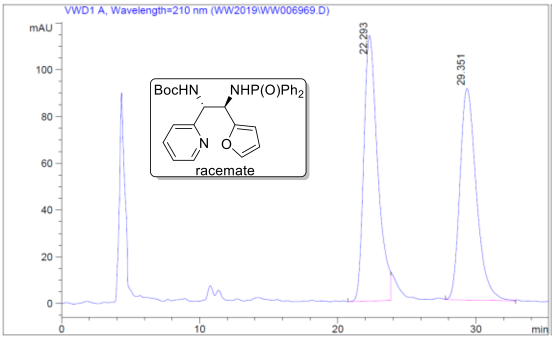

Meas.RT min	Area mAU*sec	Height mAU	Area %	Width min	Symm.
22.29	7944.0	113.6	51.4	1.165	0.669
29.35	7509.6	90.5	48.6	1.383	0.716

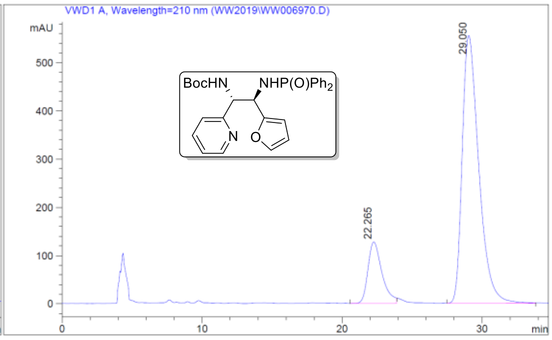

Meas.RT min	Area mAU*sec	Height mAU	Area %	Width min	Symm.
22.27	8826.6	127.3	16.2	1.156	0.697
29.05	45603.0	555.0	83.8	1.256	0.605

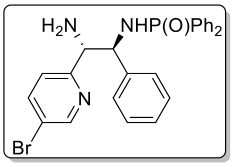

N-((1*S*,2*R*)-2-amino-2-(5-bromopyridin-2-yl)-1-phenylethyl)-P,P-diphenylphosphinic amide (*anti*-8n): white solid (44.3 mg, 90%); m.p. = 84-85 °C; R_f = 0.20 (CH_2Cl_2/MeOH = 25:1); the enantiomeric excess was determined to be 88% by HPLC analysis after being converted to the corresponding *N*-Boc derivatives. **HPLC**: Daicel Chirapak IA-H column (hexane/isopropanol = 70/30, flow rate 1.0 mL/min, T = 30°C), UV 210 nm, t_R(major) 13.74 min, t_R(minor) 12.39 min; $[\alpha]_D^{25}$ = -68.8 (c = 0.34, MeOH); **^{1}H NMR (600 MHz, $CDCl_3$)** δ 8.53 (s, 1H), 7.62 – 7.55 (m, 5H), 7.44 – 7.38 (m, 2H), 7.34 – 7.30 (m, 2H), 7.29 – 7.26 (m, 2H), 7.21 – 7.16 (m, 3H), 7.11 (d, *J* = 6.9 Hz, 2H), 6.87 (d, *J* = 8.3 Hz, 1H), 5.21 – 5.16 (m, 1H), 4.44 – 4.38 (m, 2H); **^{13}C NMR (151 MHz, $CDCl_3$)** δ 158.66, 149.95, 141.00, 140.98, 138.69, 132.78, 132.45, 132.38, 132.32, 131.93, 131.78, 131.72, 131.70, 131.68, 131.59, 128.42, 128.34, 128.32, 128.28, 128.19, 127.35, 127.14, 123.95, 119.26, 61.58, 61.55, 60.86; **HRMS (ESI)**: calcd. for $C_{25}H_{24}BrN_3OP(M+H)^+$: 492.0835, found: 492.0840.

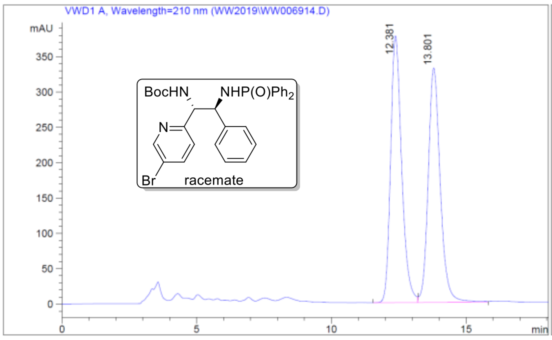

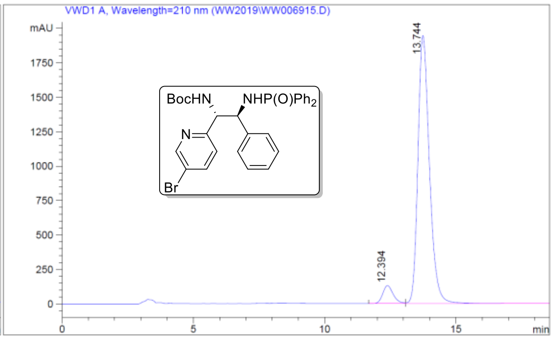

Meas.RT min	Area mAU*sec	Height mAU	Area %	Width min	Symm.
12.38	10714.8	377.1	51.0	0.436	0.736
13.80	10299.9	331.5	49.0	0.479	0.787

Meas.RT min	Area mAU*sec	Height mAU	Area %	Width min	Symm.
12.39	3655.0	130.8	5.8	0.425	0.781
13.74	59151.4	1944.5	94.2	0.466	0.705

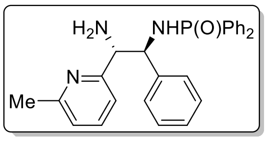

N-((1*S*,2*R*)-2-amino-2-(6-methylpyridin-2-yl)-1-phenylethyl)-P,P-diphenylphosphinic amide (*anti*-8o): white solid (28.3 mg, 68%); m.p. = 79-80 ^{o}C; R_f = 0.19 (CH_2Cl_2/MeOH = 25:1); the enantiomeric excess was determined to be 91% by HPLC analysis after being converted to the corresponding *N*-Boc derivatives. **HPLC**: Daicel Chirapak IC-H column (hexane/isopropanol = 70/30, flow rate 1.0 mL/min, T = 30°C), UV 210 nm, t_R(major) 10.18 min, t_R(minor) 6.71 min; $[\alpha]_D^{25}$ = -54.2 (c = 0.28, MeOH); **^{1}H NMR (600 MHz, $CDCl_3$)** δ 7.62 (td, *J* = 12.5, 7.6 Hz, 4H), 7.43 (t, *J* = 6.9 Hz, 1H), 7.39 (t, *J* = 7.0 Hz, 1H), 7.36 – 7.30 (m, 3H), 7.26 – 7.24 (m, 1H), 7.18 – 7.15 (m, 3H), 7.11 – 7.06 (m, 2H), 6.99 (d, *J* = 7.6 Hz, 1H), 6.65 (d, *J* = 7.6 Hz, 1H), 5.73 (t, *J* = 9.0 Hz, 1H), 4.60 (d, *J* = 6.6 Hz, 1H), 4.41 (dd, *J* = 16.7, 9.9 Hz, 1H), 2.47 (s, 3H); **^{13}C NMR (151 MHz, $CDCl_3$)** δ 157.66, 140.75, 140.72, 136.56, 132.55, 132.49, 131.83, 131.78, 131.76, 131.69, 131.67, 128.43, 128.34, 128.25, 128.22, 128.17, 127.43, 127.35, 122.23, 119.95, 61.11, 61.08, 60.67, 24.32 **HRMS (ESI)**: calcd. for $C_{26}H_{27}N_3OP(M+H)^+$: 428.1886, found: 428.1877.

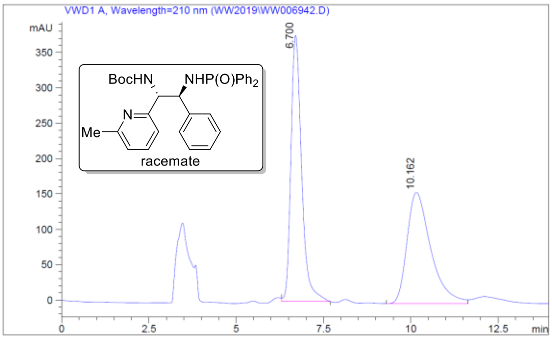

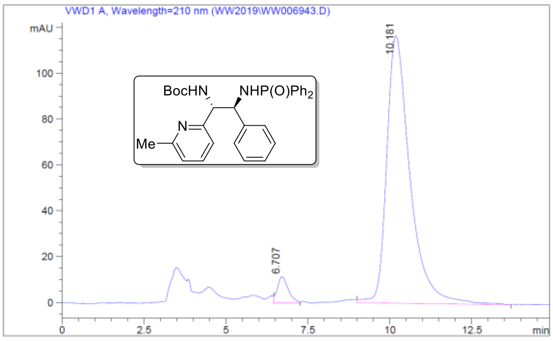

Meas.RT min	Area mAU*sec	Height mAU	Area %	Width min	Symm.
6.70	7997.9	377.0	51.8	0.354	0.683
10.16	7438.5	157.2	48.2	0.709	0.606

Meas.RT min	Area mAU*sec	Height mAU	Area %	Width min	Symm.
6.71	267.8	11.5	4.4	0.387	0.682
10.18	5760.9	116.5	95.6	0.740	0.603

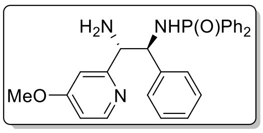

N-((1*S*,2*R*)-2-amino-2-(4-methoxypyridin-2-yl)-1-phenylethyl)-P,P-diphenylphosphinic amide (*anti*-8p): white solid (34.6 mg, 78%); m.p. = 172-173 ^{o}C; R_f = 0.24 (CH_2Cl_2/MeOH = 25:1); the enantiomeric excess was determined to be 90% by HPLC analysis after being converted to the corresponding

N-Boc derivatives. **HPLC**: Daicel Chirapak IA-H column (hexane/isopropanol = 70/30, flow rate 1.0 mL/min, T = 30°C), UV 210 nm, t_R(major) 12.10 min, t_R(minor) 33.19 min; $[\alpha]_D^{25}$ = +65.6 (c = 0.31, MeOH); **^{1}H NMR (600 MHz, CD_3OD)** δ 8.28 (d, *J* = 5.7 Hz, 1H), 7.71 (dd, *J* = 12.2, 7.5 Hz, 2H), 7.64 (dd, *J* = 12.3, 7.4 Hz, 2H), 7.58 – 7.55 (m, 1H), 7.51 – 7.45 (m, 4H), 7.36 – 7.34 (m, 1H), 7.18 – 7.16 (m, 3H), 7.00 – 6.98 (m, 2H), 6.76 (dd, *J* = 5.7, 2.3 Hz, 1H), 6.45 (d, *J* = 2.1 Hz, 1H), 4.39 – 4.32 (m, 2H), 3.65 (s, 3H); **^{13}C NMR (151 MHz, CD_3OD)** δ 166.34, 158.06, 149.94, 139.85, 132.26, 132.20, 132.09, 132.07, 131.47, 131.40, 128.44, 128.36, 128.27, 128.19, 128.09, 127.41, 126.96, 109.72, 109.31, 61.64, 61.61, 60.93, 54.51; **HRMS (ESI)**: calcd. for $C_{26}H_{27}N_3O_2P(M+H)^+$: 444.1835, found: 444.1836.

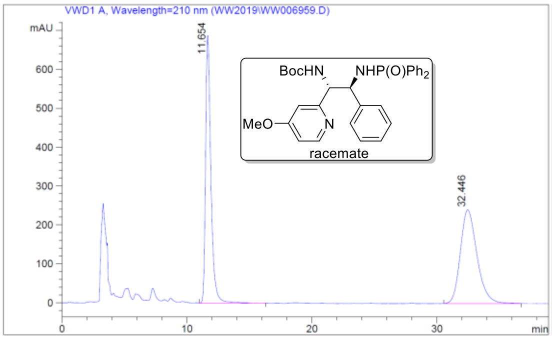

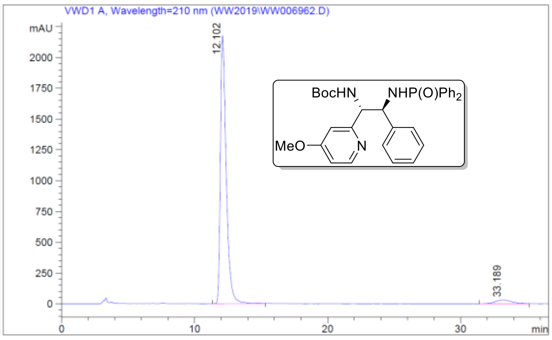

Meas.RT min	Area mAU*sec	Height mAU	Area %	Width min	Symm.
11.65	20641.6	688.8	48.1	0.499	0.645
32.45	22252.7	240.9	51.9	1.433	0.739

Meas.RT min	Area mAU*sec	Height mAU	Area %	Width min	Symm.
12.10	65780.5	2173.7	96.2	0.452	0.547
33.19	2598.8	30.3	3.8	1.319	0.821

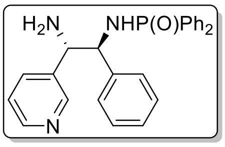

N-((1*S*,2*S*)-2-amino-1-phenyl-2-(pyridin-3-yl)ethyl)-P,P-diphenylphosphinic amide (*anti*-8q): faint yellow semi-solid (27.3 mg, 66%); R_f = 0.20 (CH_2Cl_2/MeOH = 25:1); the enantiomeric excess was determined to be 75% by HPLC analysis after being converted to the corresponding *N*-Boc derivatives. **HPLC**: Daicel Chirapak IC-H column (hexane/isopropanol = 80/20, flow rate 1.0 mL/min, T = 30°C), UV 210 nm, t_R(major) 10.72 min, t_R(minor) 9.25 min; $[\alpha]_D^{25}$ = -25.6 (c = 0.51, MeOH); **^{1}H NMR (600 MHz, $CDCl_3$)** δ 8.33 (s, 1H), 8.24 – 8.22 (m, 1H), 7.82 (d, *J* = 7.4 Hz, 1H), 7.76 (dd, *J* = 12.0, 7.8 Hz, 2H), 7.42 (dd, *J* = 12.0, 7.8 Hz, 2H), 7.37 (dd, *J* = 12.4, 6.5 Hz, 2H), 7.31 – 7.27 (m, 2H), 7.17 – 7.12 (m, 2H), 7.10 – 7.03 (m, 3H), 6.98 – 6.92 (m, 3H), 5.06 (d, *J* = 10.1 Hz, 1H), 4.58 (dd, *J* = 21.4, 10.7 Hz, 1H); **^{13}C NMR (151 MHz, $CDCl_3$)** δ 149.81, 149.34, 139.32, 136.14, 132.85, 132.78, 132.23, 131.90, 131.83, 131.48, 131.27, 130.43, 129.56, 128.70, 128.61, 128.47, 128.29, 128.20, 127.73, 127.50, 123.28, 60.24, 58.28; **HRMS (ESI)**: calcd. for $C_{25}H_{25}N_3OP(M+H)^+$: 414.1730, found: 414.1728.

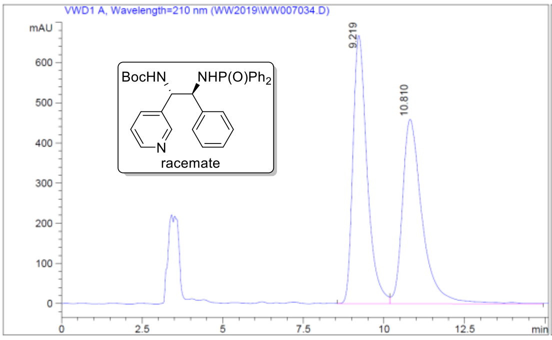

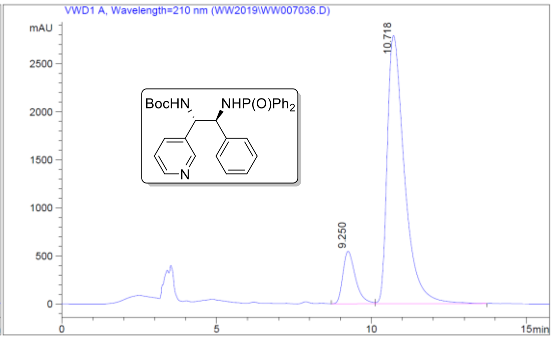

Meas.RT min	Area mAU*sec	Height mAU	Area %	Width min	Symm.
9.22	20421.9	667.5	50.9	0.510	0.714
10.81	19697.4	458.7	49.1	0.716	0.627

Meas.RT min	Area mAU*sec	Height mAU	Area %	Width min	Symm.
9.25	15370.4	545.4	12.5	0.428	0.657
10.72	108041.7	2789.9	87.5	0.586	0.522

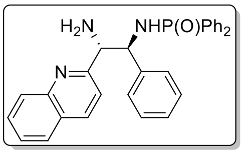

N-((1*S*,2*R*)-2-amino-1-phenyl-2-(quinolin-2-yl)ethyl)-P,P-diphenylphosphinic amide (*anti*-8r): white solid (40.6 mg, 88%); m.p. = 103-104 ^{o}C; R_f = 0.20 (CH_2Cl_2/MeOH = 25:1); the enantiomeric excess was determined to be 96% by HPLC analysis after being converted to the corresponding *N*-Boc derivatives. **HPLC**: Daicel Chirapak IA-H column (hexane/isopropanol = 70/30, flow rate 1.0 mL/min, T = 30°C), UV 210 nm, t_R(major) 10.00 min, t_R(minor) 6.52 min; $[\alpha]_D^{25}$ = -140.2 (c = 0.24, MeOH); **^{1}H NMR (600 MHz, $CDCl_3$)** δ 8.05 (d, *J* = 8.4 Hz, 1H), 7.96 (d, *J* = 8.4 Hz, 1H), 7.79 (d, *J* = 8.0 Hz, 1H), 7.73 (t, *J* = 7.6 Hz, 1H), 7.62 (dd, *J* = 11.9, 7.6 Hz, 2H), 7.55 (t, *J* = 7.4 Hz, 1H), 7.41 – 7.31 (m, 4H), 7.26 – 7.18 (m, 7H), 7.13 – 7.07 (m, 3H), 5.35 (t, *J* = 9.1 Hz, 1H), 4.71 (d, *J* = 5.2 Hz, 1H), 4.54 (td, *J* = 9.5, 5.5 Hz, 1H); **^{13}C NMR (151 MHz, $CDCl_3$)** δ 159.93, 147.25, 141.24, 136.24, 132.39, 132.33, 131.76, 131.69, 131.65, 131.63, 131.55, 131.54, 129.66, 129.15, 128.33, 128.24, 128.23, 128.16, 128.14, 127.63, 127.49, 127.36, 127.31, 126.52, 120.53, 61.93, 61.89, 60.67; **HRMS (ESI)**: calcd. for $C_{26}H_{27}N_3OP(M+H)^+$: 464.1886, found: 428.1878.

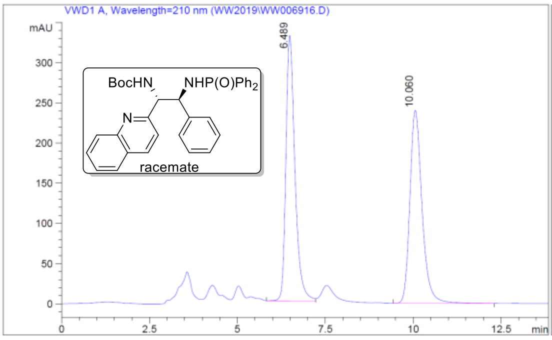

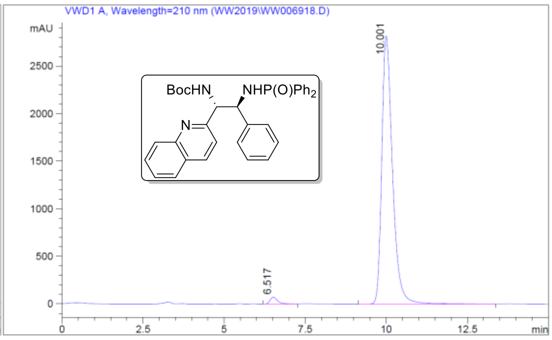

Meas.RT min	Area mAU*sec	Height mAU	Area %	Width min	Symm.
6.49	5880.0	330.8	50.5	0.296	0.703
10.06	5757.8	239.9	49.5	0.367	0.735

Meas.RT min	Area mAU*sec	Height mAU	Area %	Width min	Symm.
6.52	1199.4	72.5	1.8	0.246	0.632
10.00	64871.0	2816.7	98.2	0.350	0.645

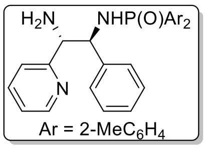

N-((1*S*,2*R*)-2-amino-1-phenyl-2-(pyridin-2-yl)ethyl)-P,P-di-o-tolylphosphinic amide (*anti*-8s): white solid (30.4 mg, 69%); m.p. = 92-93 ^{o}C; R_f = 0.24 (CH_2Cl_2/MeOH = 25:1); the enantiomeric excess was determined to be 88% by HPLC analysis after being converted to the corresponding *N*-Boc

derivatives. **HPLC**: Daicel Chirapak OJ-H column (hexane/isopropanol = 80/20, flow rate 1.0 mL/min, T = 30°C), UV 210 nm, t_R(major) 3.68 min, t_R(minor) 5.24 min; $[\alpha]_D^{25}$ = -49.4 (c = 0.65, MeOH); **^{1}H NMR (600 MHz, $CDCl_3$)** δ 8.47 (d, *J* = 4.1 Hz, 1H), 7.58 (ddd, *J* = 24.8, 13.4, 7.6 Hz, 2H), 7.43 (t, *J* = 7.6 Hz, 1H), 7.32 – 7.25 (m, 2H), 7.15 – 7.01 (m, 10H), 6.91 (d, *J* = 7.6 Hz, 1H), 5.29 – 5.21 (m, 1H), 4.55 (dd, *J* = 15.2, 9.1 Hz, 1H), 4.44 (d, *J* = 5.7 Hz, 1H), 2.23 (s, 3H), 2.11 (s, 3H); **^{13}C NMR (151 MHz, $CDCl_3$)** δ 160.19, 148.71, 141.99, 141.92, 141.76, 141.69, 141.41, 141.39, 136.11, 133.30, 133.24, 132.83, 132.76, 131.89, 131.58, 131.56, 131.52, 131.47, 131.45, 131.44, 131.42, 131.34, 131.05, 130.73, 128.03, 127.37, 127.08, 125.35, 125.27, 125.13, 125.04, 122.58, 122.27, 61.97, 61.94, 61.33, 21.39, 21.37, 21.34, 21.31; **HRMS (ESI)**: calcd. for $C_{27}H_{29}N_3OP(M+H)^+$: 442.2043, found: 442.2038.

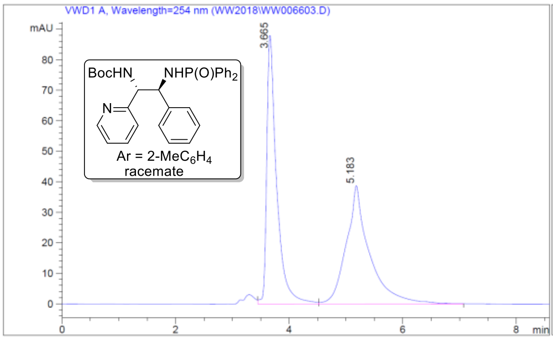

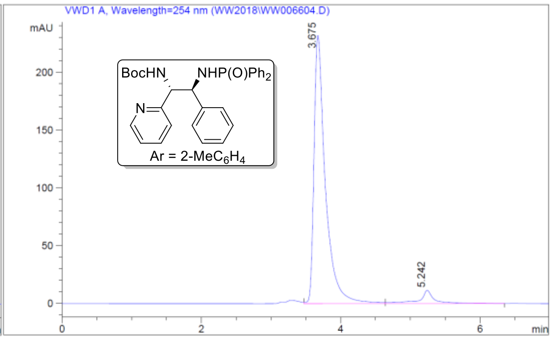

Meas.RT min	Area mAU*sec	Height mAU	Area %	Width min	Symm.
3.66	1066.3	88.1	49.1	0.176	0.452
5.18	1106.1	38.6	50.9	0.364	0.699

Meas.RT min	Area mAU*sec	Height mAU	Area %	Width min	Symm.
3.68	2638.2	230.6	93.9	0.168	0.488
5.24	172.1	11.3	6.1	0.200	1.043

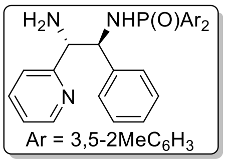

N-((1*S*,2*R*)-2-amino-1-phenyl-2-(pyridin-2-yl)ethyl)-P,P-bis(3,5-dimethylphenyl)phosphinic amide (*anti*-8t): white solid (30.5 mg, 65%); m.p. = 93-94 ^{o}C; R_f = 0.22 (CH_2Cl_2/MeOH = 25:1); the enantiomeric excess was determined to be 90% by HPLC analysis after being converted to the corresponding *N*-Boc derivatives. **HPLC**: Daicel Chirapak IC-H column (hexane/isopropanol = 85/15, flow rate 1.0 mL/min, T = 30°C), UV 210 nm, t_R(major) 11.26 min, t_R(minor) 8.76 min; $[\alpha]_D^{25}$ = -14.0 (c = 0.30, MeOH); **^{1}H NMR (600 MHz, $CDCl_3$)** δ 8.50 (d, *J* = 4.0 Hz, 1H), 7.46 – 7.41 (m, 1H), 7.27 – 7.23 (m, 2H), 7.21 – 7.15 (m, 5H), 7.12 – 7.08 (m, 3H), 7.03 (s, 1H), 6.98 (s, 1H), 6.83 (d, *J* = 7.7 Hz, 1H), 5.33 (t, *J* = 7.5 Hz, 1H), 4.54 (d, *J* = 6.7 Hz, 1H), 4.38 (dd, *J* = 17.1, 9.9 Hz, 1H), 2.25 (s, 6H), 2.15 (s, 6H); **^{13}C NMR (151 MHz, $CDCl_3$)** δ 159.19, 148.84, 141.19, 141.16, 138.05, 137.96, 137.80, 137.71, 136.11, 133.56, 133.54, 133.34, 133.32, 132.48, 132.04, 131.64, 131.17, 130.33, 130.26, 129.30, 129.24, 128.14, 127.40, 127.22, 123.00, 122.50, 61.74, 61.72, 61.02, 21.27, 21.08; **HRMS (ESI)**: calcd. for $C_{29}H_{33}N_3OP(M+H)^+$: 470.2356, found: 470.2358.

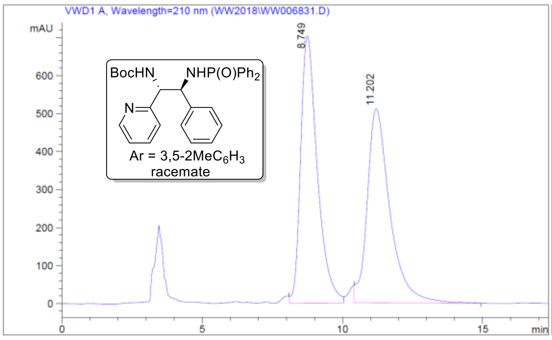

Meas.RT min	Area mAU*sec	Height mAU	Area %	Width min	Symm.
8.75	29095.8	703.6	50.3	0.622	0.627
11.20	28775.9	510.9	49.7	0.939	0.643

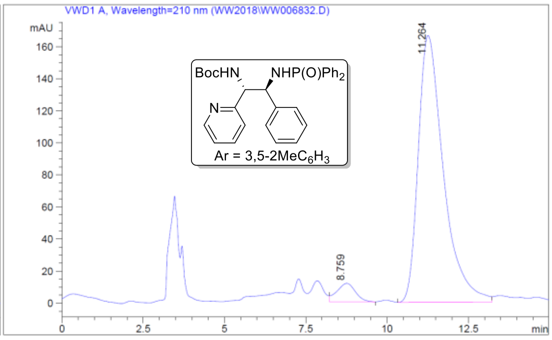

Meas.RT min	Area mAU*sec	Height mAU	Area %	Width min	Symm.
8.76	453.3	11.5	4.8	0.594	1.099
11.26	9016.7	166.6	95.2	0.902	0.607

7. Determination of the absolute configurations of *anti*-5g, *syn*-5g, *anti*-8a and *syn*-8a.

The absolute structure of *anti*-**5g** was determined by X-ray diffraction analysis.

Crystallographic data for *anti*-5g.

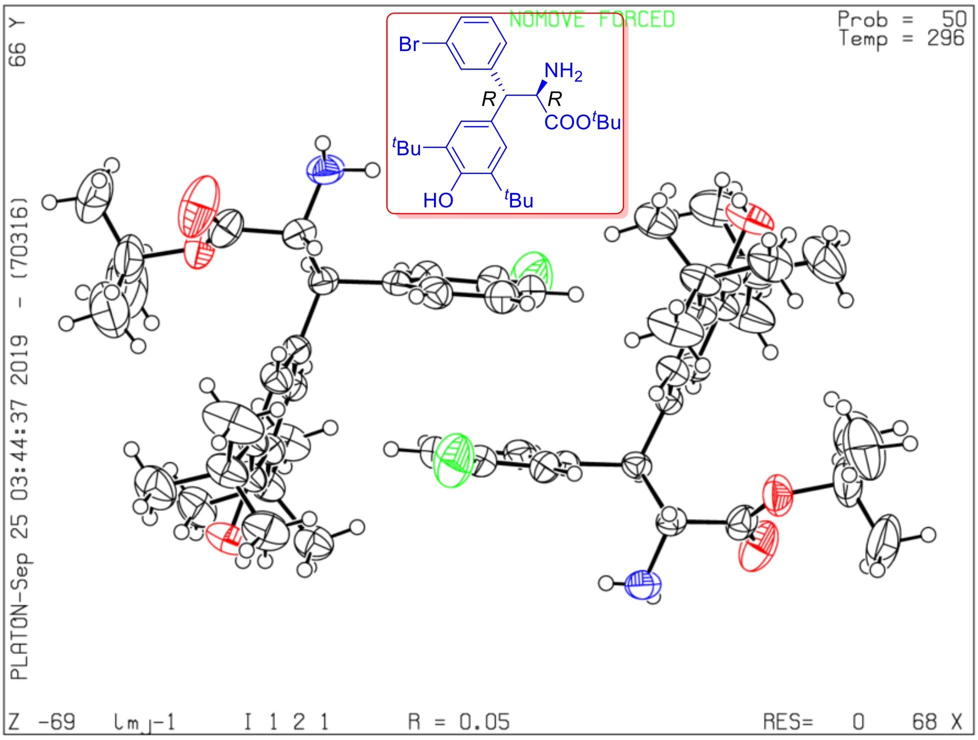

Chemical Formula	$C_{27}H_{38}BrNO_3$
Formula weight	504.49
Temperature	296 K
Wavelength	1.54184 A
Crystal system, space group	Monoclinic, I 1 2 1
a, Å	18.63267(17)
b, Å	17.29388(17)
c, Å	18.09742(15)
α, °	90.00
β, °	108.0969(9)
γ, °	90.00
V, $Å^3$	5543.08(9)
F(000)	2128.0
Z, Calculated density	8, 1.209 Mg/m^3

The absolute configuration of *syn*-**5g** was established on the basis of the comparison of the literature data with the specific rotation.

Literature:

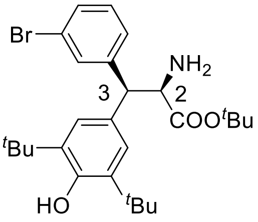

(2*R*, 3*S*)

This work:

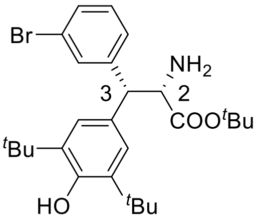

(2*S*, 3*R*)

specific rotation:

Literature[8]: $[\alpha]_D^{21}$ = -60 (c = 1, $CHCl_3$) at 97% *ee*;

This work: $[\alpha]_D^{25}$ = +21.5 (c = 0.4, $CHCl_3$) at 90% *ee.*

The absolute configuration of *syn*-**8a** and *anti*-**8a** were confirmed by the X-ray structure of their derivatized products **10** and **12**.

Procedure for the synthesis of compound 10.

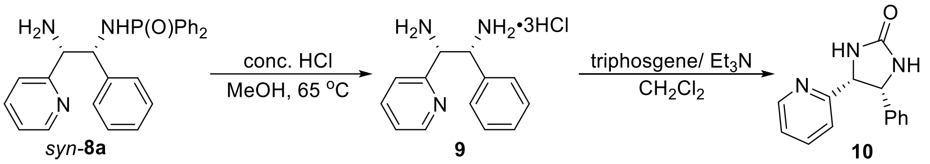

syn-**8a** **9** **10**

(1*R*,2*R*)-1-phenyl-2-(pyridin-2-yl)ethane-1,2-diamine hydrochloride (9):

To a solution of compound *syn*-**8a** (162 mg, 0.39 mmol) in MeOH (5 mL) was slowly added conc. HCl (3 mL) at room temperature. After that, the reaction mixture was stirred for 8 hours in reflux. The mixture was then concentrated *in vacuo* and redissolved in H_2O (20 mL). The aqueous phase was washed with EtOAc (10 mL × 3) and concentrated under reduced pressure to afford compound **9** (109 mg, 0.34 mmol, 88%).

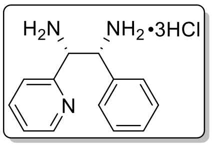

^{1}H NMR (600 MHz, D_2O) δ 8.70 (d, *J* = 4.1 Hz, 1H), 7.92 (t, *J* = 7.6 Hz, 1H), 7.57 (d, *J* = 7.6 Hz, 1H), 7.54 – 7.49 (m, 6H), 4.99 (dd, *J* = 35.1, 9.9 Hz, 2H); **^{13}C NMR (151 MHz, D_2O)** δ 151.12, 150.00, 139.30, 131.21, 131.17, 130.32, 128.09, 126.20, 125.28, 56.94, 56.62.

(4*R*,5*R*)-4-phenyl-5-(pyridin-2-yl)imidazolidin-2-one (10):

To a solution of compound **9** (109 mg, 0.34 mmol) and Et_3N (283 uL, 2.04 mmol) in CH_2Cl_2 (2 mL), triphosgene solution (35 mg, 0.12 mmol, dissolved in 0.5 mL CH_2Cl_2) was added in dropwise at 0 °C. After the temperature was raised to room temperature, an additional 1 hour of stirring was required. After **9** was completely consumed, the reaction was quenched by saturated NaCl (2 mL) and extracted by CH_2Cl_2 3 times (5 mL × 3). The combined organic layer was dried by anhydrous Na_2SO_4, filtered and concentrated *in vacuo*. Purification of the residue by flash column chromatography on silica gel (petroleum: AcOEt = 1:4) gave compound **10** (70 mg, 0.29 mmol, 86%).

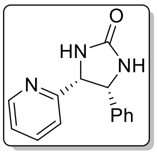

white solid; m.p. = 219-220 °C; R_f = 0.20 (CH_2Cl_2/MeOH = 25:1); $[\alpha]_D^{25}$ = -95.0 (c = 0.30, MeOH); **^{1}H NMR (400 MHz, $CDCl_3$)** δ 8.33 (d, *J* = 4.2 Hz, 1H), 7.36 (td, *J* = 7.7, 1.7 Hz, 1H), 7.08 – 7.04 (m, 3H), 7.03 – 6.93 (m, 4H), 5.48 (s, 1H), 5.38 – 5.27 (m, 3H); **^{13}C NMR (151 MHz, $CDCl_3$)** δ 164.07, 157.28, 148.67, 137.40, 136.08, 127.97, 127.71, 126.97, 122.34, 121.47, 62.89, 60.88; **HRMS (ESI)**: calcd. for $C_{14}H_{14}N_3O(M+H)^+$: 240.1131, found: 240.1135.

Crystallographic data for 10.

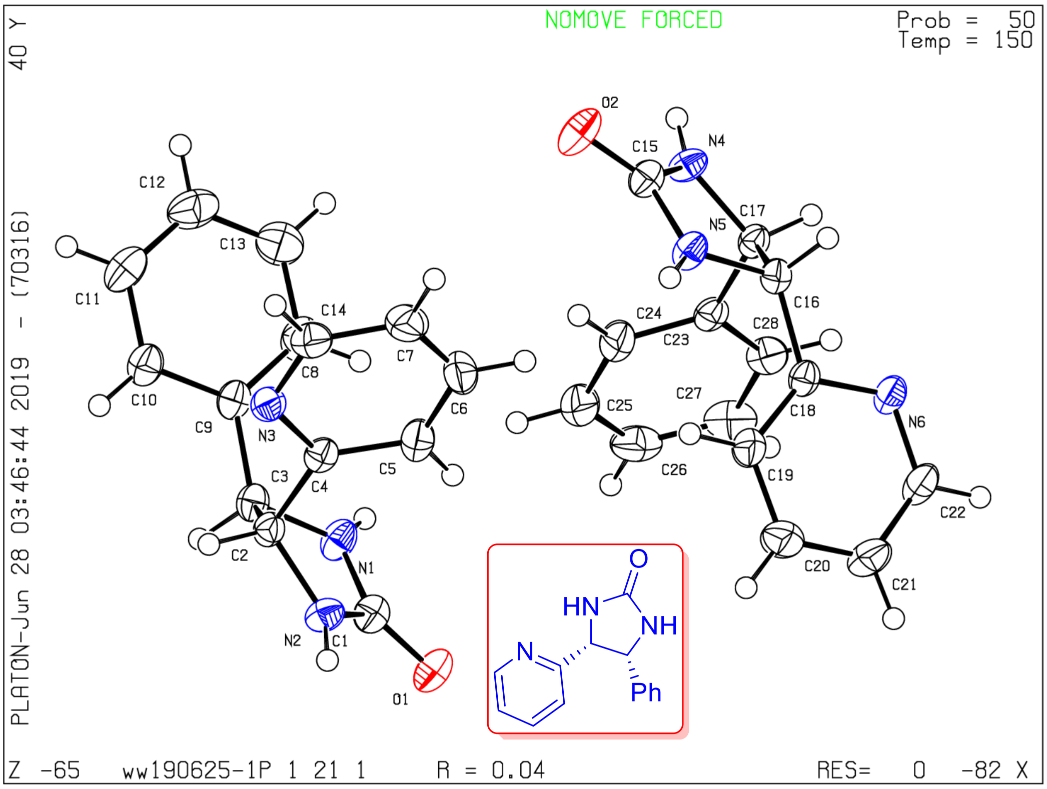

Chemical Formula	$2(C_{14}H_{13}N_3O)$
Formula weight	478.55
Temperature	150 K
Wavelength	1.54184
Crystal system, space group	Monoclinic, P 1 21 1
a, Ǻ	8.57090(14)
b, Ǻ	14.7478(2)
c, Ǻ	9.95745(12)
α, °	90.00
β, °	101.9926(14)
γ, °	90.00
V, Ǻ^3	1231.17(3)
F(000)	504.0
Z, Calculated density	2, 1.291 Mg/m^3

Procedure for the synthesis of compound 12.

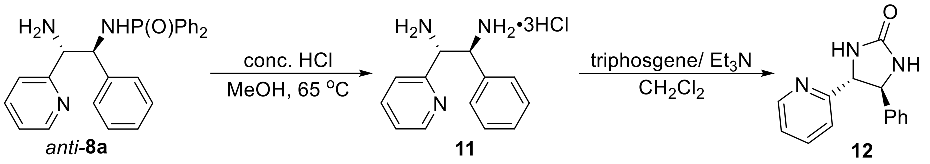

(1*S*,2*R*)-1-phenyl-2-(pyridin-2-yl)ethane-1,2-diamine hydrochloride (11):

To a solution of compound *anti*-**8a** (239 mg, 0.58 mmol) in MeOH (12 mL) was slowly added conc. HCl (4 mL) at room temperature. Then the reaction was stirred for 8 hours in reflux. The mixture was concentrated *in vacuo*, and redissolved in H_2O (20 mL). The resulted aqueous phase was washed with EtOAc (10 mL × 3). The aqueous layer was concentrated under reduced pressure to afford compound **11** (191 mg, 0.53 mmol, 92%).

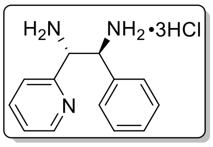

^{1}H NMR (400 MHz, D_2O) δ 8.47 (d, *J* = 4.7 Hz, 1H), 7.71 (t, *J* = 7.8 Hz, 1H), 7.37 –7.33 (m, 1H), 7.30 – 7.20 (m, 4H), 7.09 (d, *J* = 7.9 Hz, 2H), 5.03 (dd, *J* = 51.3, 7.9 Hz, 2H); **^{13}C NMR (101 MHz, D_2O)** δ 149.95, 149.25, 139.36, 130.61, 130.33, 129.53, 127.64, 125.46, 124.33, 56.91, 56.55.

(4*S*,5*R*)-4-phenyl-5-(pyridin-2-yl)imidazolidin-2-one (12):

To a solution of compound **11** (100 mg, 0.31 mmol) and Et_3N (258 uL, 1.86 mmol) in CH_2Cl_2 (1 mL), triphosgene solution (33 mg, 0.11 mmol, dissolved in 0.2 mL CH_2Cl_2) was added dropwise at 0 ^{o}C. Then the reaction temperature was warmed to room temperature and stirred for additional 1 hour. After **11** was completely consumed, the reaction was quenched by saturated NaCl (2 mL) and extracted using CH_2Cl_2 3 times (5 mL × 3). The combined organic layer was dried by anhydrous Na_2SO_4, filtered and concentrated *in vacuo*. Purification of the residue by flash column chromatography on silica gel (petroleum: AcOEt = 1:4) gave compound **12** (64 mg, 0.27 mmol, 87%).

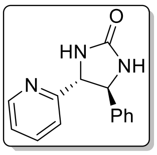

white solid; m.p. = 161-162 ^{o}C; R_f = 0.27 (CH_2Cl_2/MeOH = 25:1); $[\alpha]_D^{25}$ = -271.0 (c = 0.10, MeOH); **^{1}H NMR (600 MHz, $CDCl_3$)** δ 8.62 (d, *J* = 3.7 Hz, 1H), 7.73 (t, *J* = 7.2 Hz, 1H), 7.41 – 7.37 (m, 5H), 7.35 – 7.32 (m, 1H), 7.26 – 7.22 (m, 1H), 5.49 (s, 1H), 5.22 (s, 1H), 4.85 (d, *J* = 5.5 Hz, 1H), 4.75 (d, *J* = 5.4 Hz, 1H); **^{13}C NMR (151 MHz, $CDCl_3$)** δ 162.54, 159.80, 149.87, 141.02, 137.15, 128.99, 128.42, 126.31, 123.20, 120.76, 66.24, 63.50; **HRMS (ESI)**: calcd. for $C_{14}H_{14}N_3O(M+H)^+$: 240.1131, found: 240.1136.

Crystallographic data for 12.

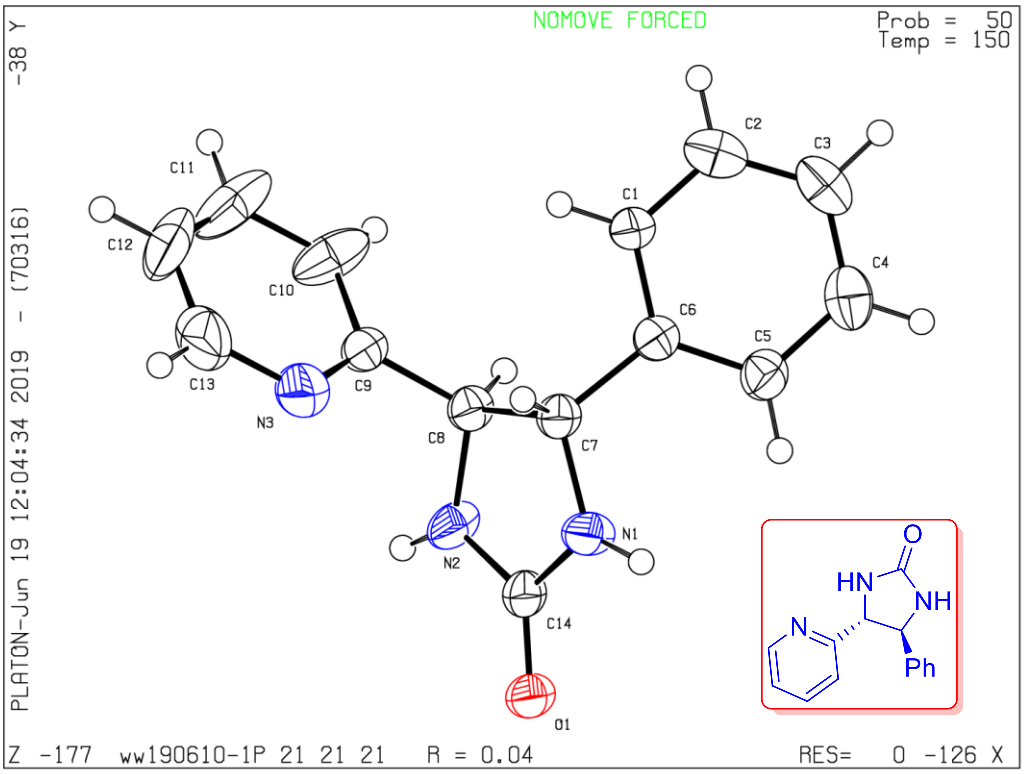

Chemical Formula	$C_{14}H_{13}N_3O$
Formula weight	239.27
Temperature	150 K
Wavelength	1.54184
Crystal system, space group	Orthorhombic, P 21 21 21
a, Å	7.18461(6)
b, Å	11.07992(13)
c, Å	15.75550(15)
α, °	90.00
β, °	90.00
γ, °	90.00
V, $Å^3$	1254.22(2)
F(000)	504.0
Z, Calculated density	4, 1.267 Mg/m^3

8. The spectra of ^{1}H NMR and ^{13}C NMR

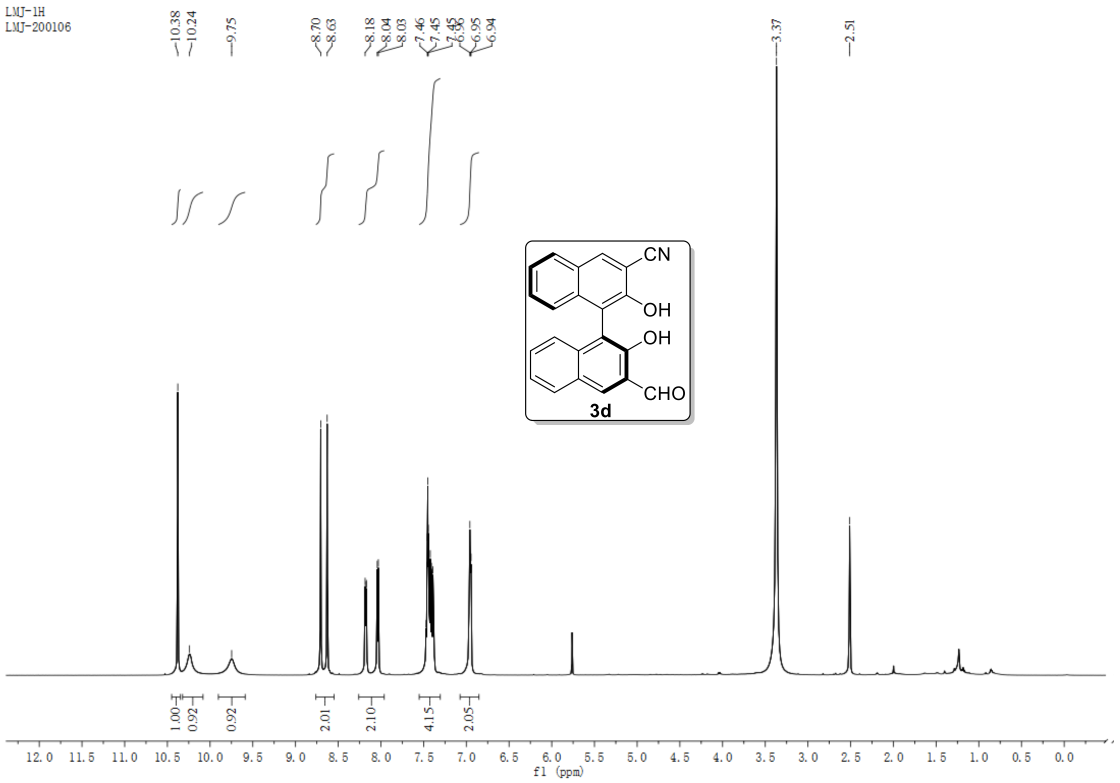

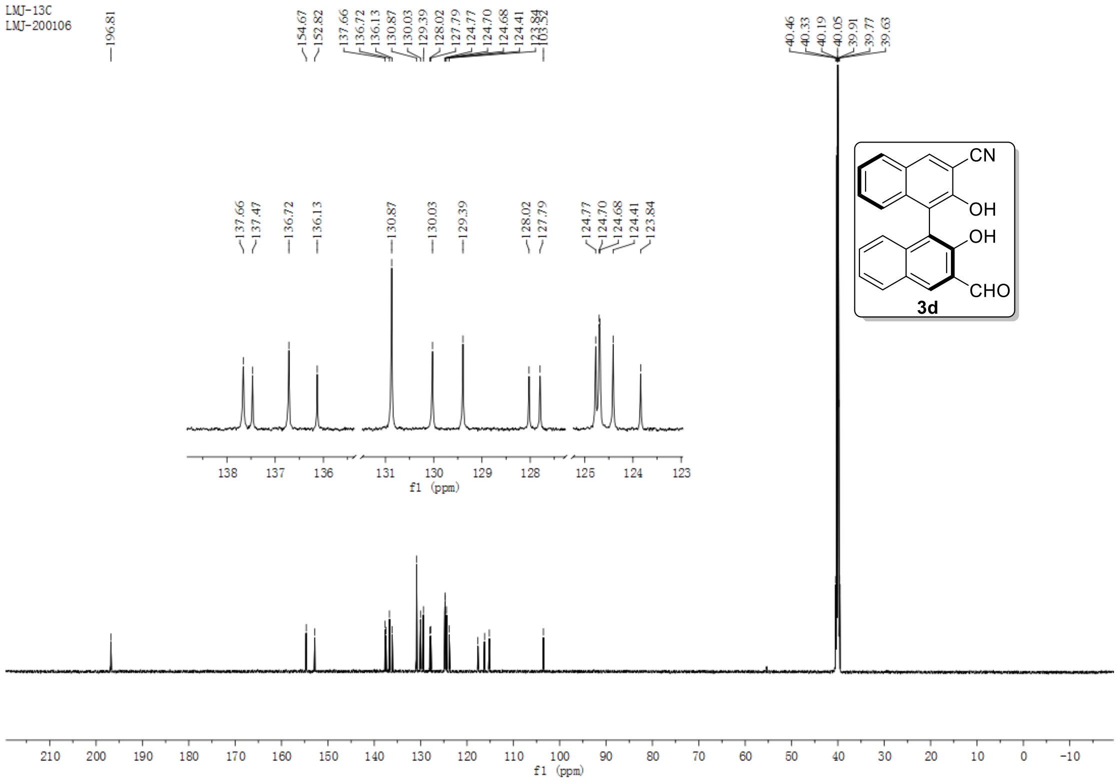

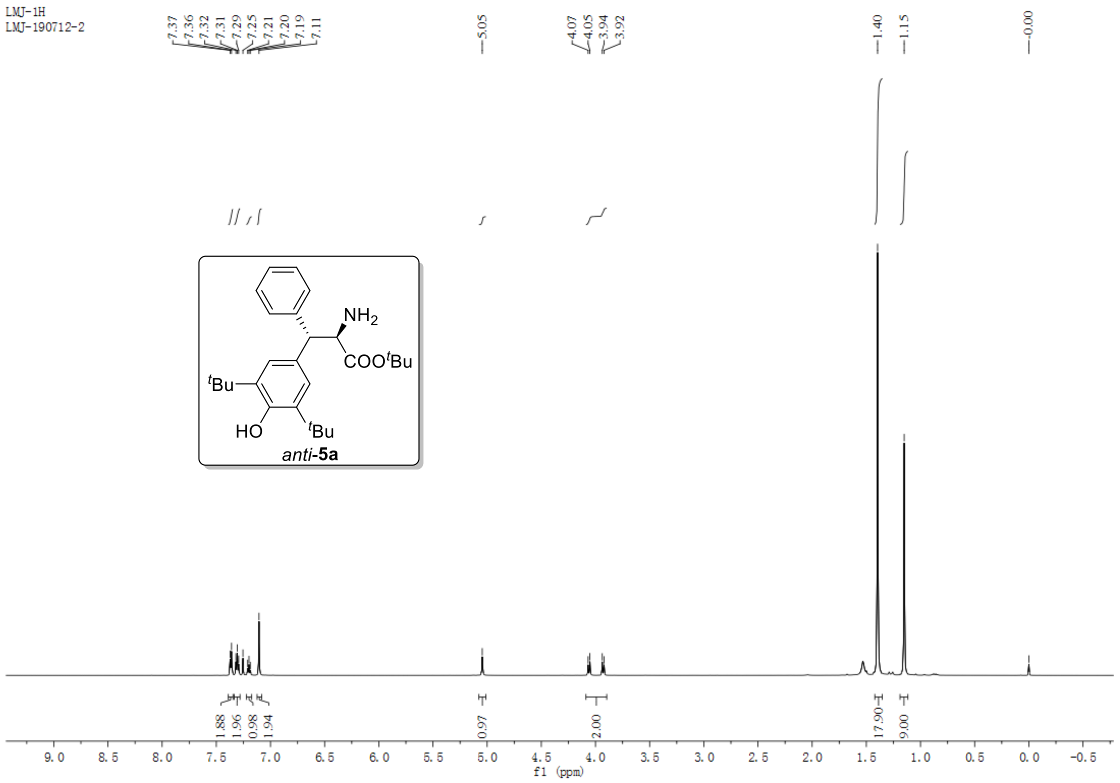
LMJ-1H
LMJ-190712-2
7.37 7.36 7.32 7.31 7.29 7.25 7.21 7.20 7.19 7.11
5.05
4.07 4.05 3.94 3.92
1.40 1.15
0.00
NH2
COOtBu
tBu
HO
tBu
anti-5a
1.88 1.96 0.98 1.94
0.97
2.00
17.90
9.00
f1 (ppm)

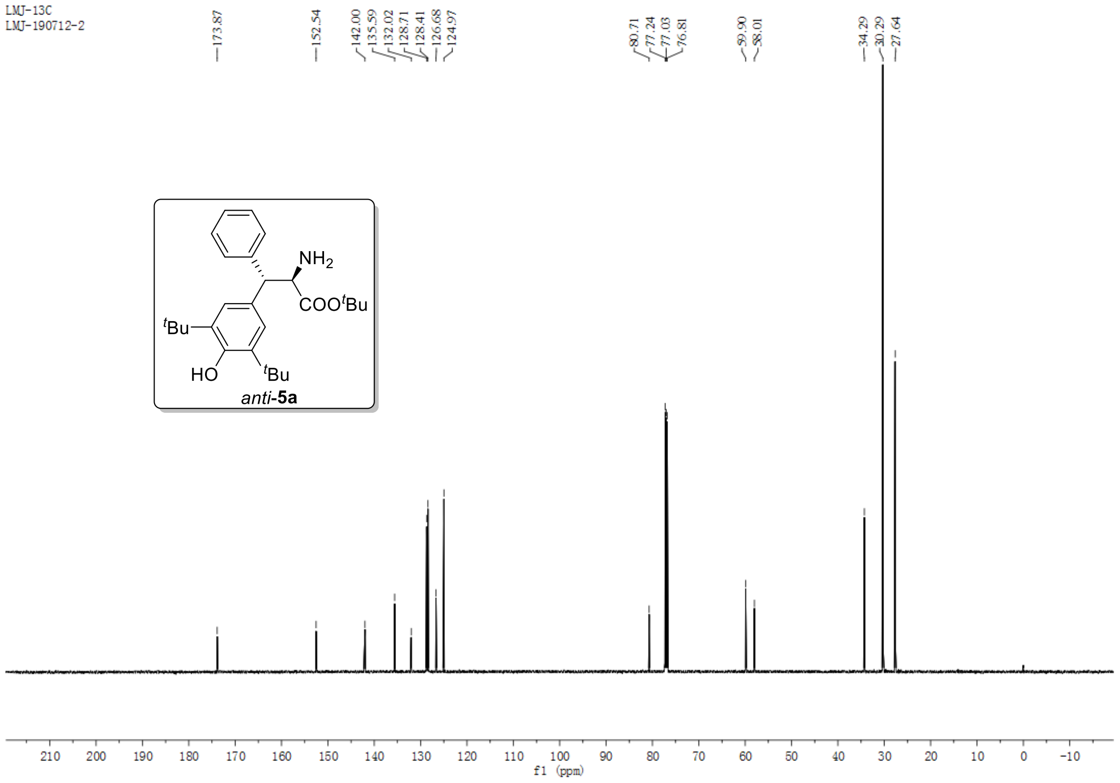
LMJ-13C
LMJ-190712-2
173.87
152.54
142.00 135.59 132.02 128.71 128.41 126.68 124.97
80.71 77.24 77.03 76.81
59.90 58.01
34.29 30.29 27.64
NH2
COOtBu
tBu
HO
tBu
anti-5a
f1 (ppm)

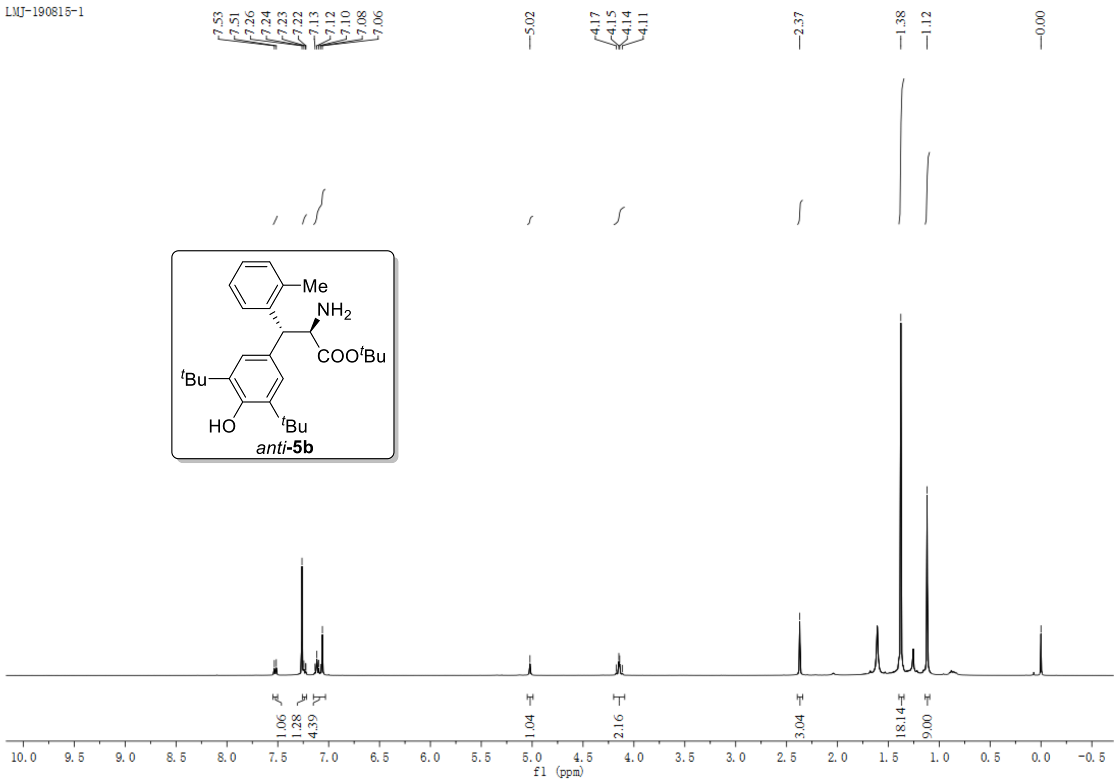

LMJ-190815-1
7.53 7.51 7.26 7.24 7.23 7.22 7.13 7.12 7.10 7.08 7.06
5.02
4.17 4.15 4.14 4.11
2.37
1.38
1.12
0.00
Me
NH2
COO^tBu
^tBu
HO
^tBu
anti-5b
1.06 1.28 4.39 1.04 2.16 3.04 18.14 9.00
f1 (ppm)

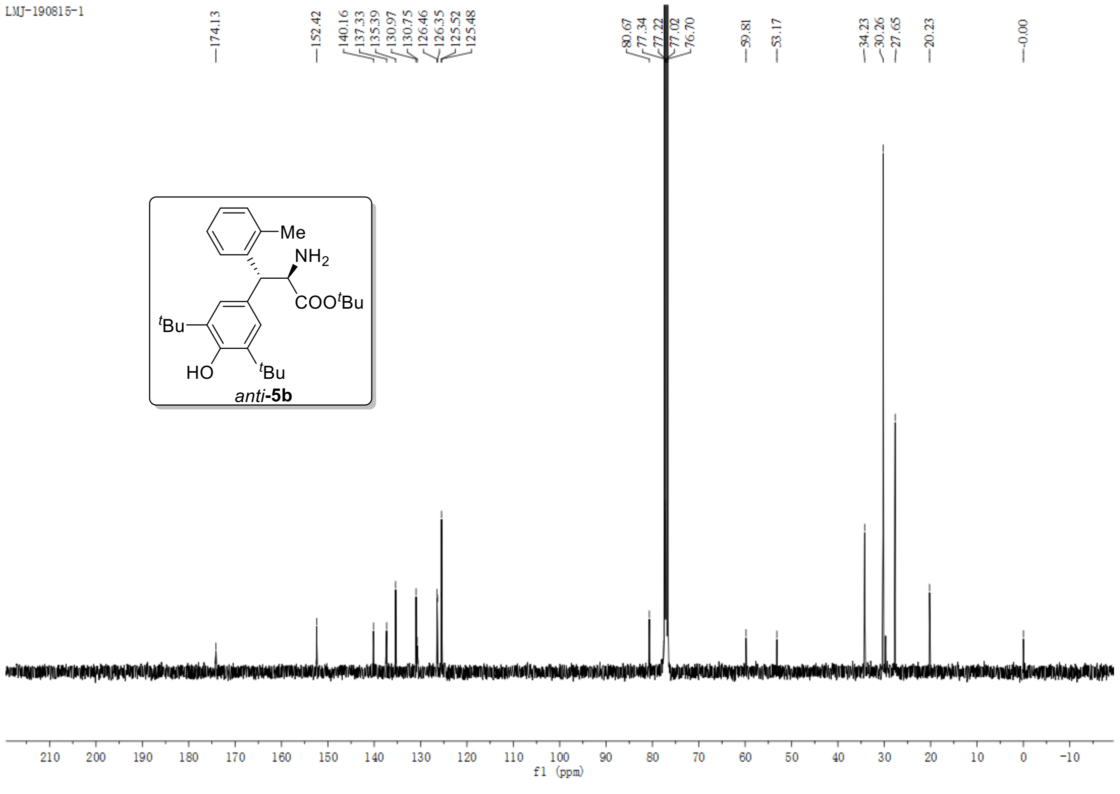

LMJ-190815-1
174.13
152.42
140.16 137.33 135.39 130.97 130.75 126.46 126.35 125.52 125.48
80.67 77.34 77.22 77.02 76.70
59.81
53.17
34.23 30.26 27.65
20.23
0.00
Me
NH2
COO^tBu
^tBu
HO
^tBu
anti-5b
f1 (ppm)

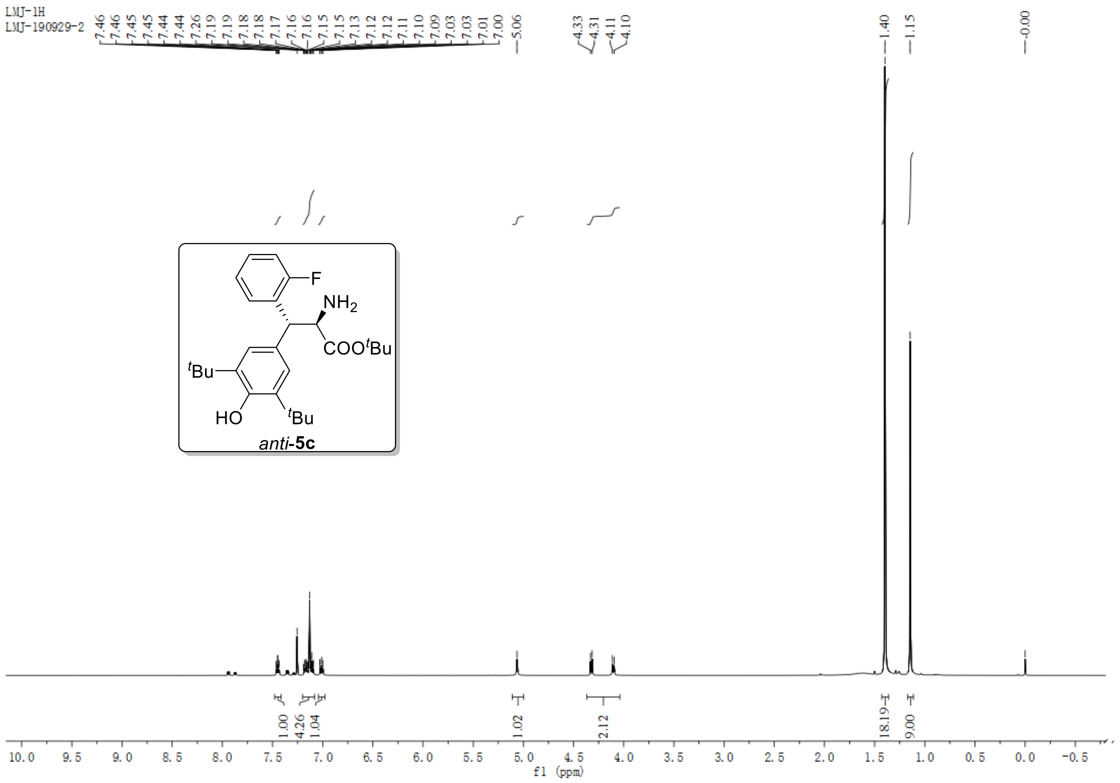
LMJ-1H
LMJ-190929-2
anti-5c
f1 (ppm)

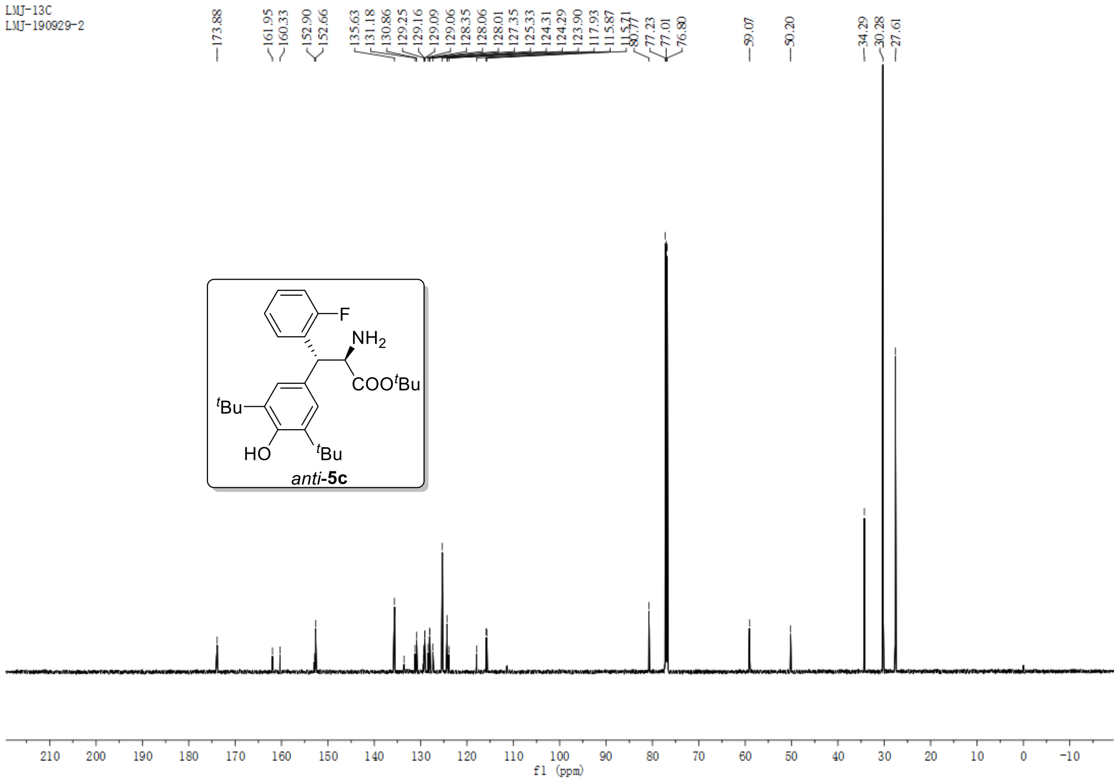
LMJ-13C
LMJ-190929-2
anti-5c
f1 (ppm)

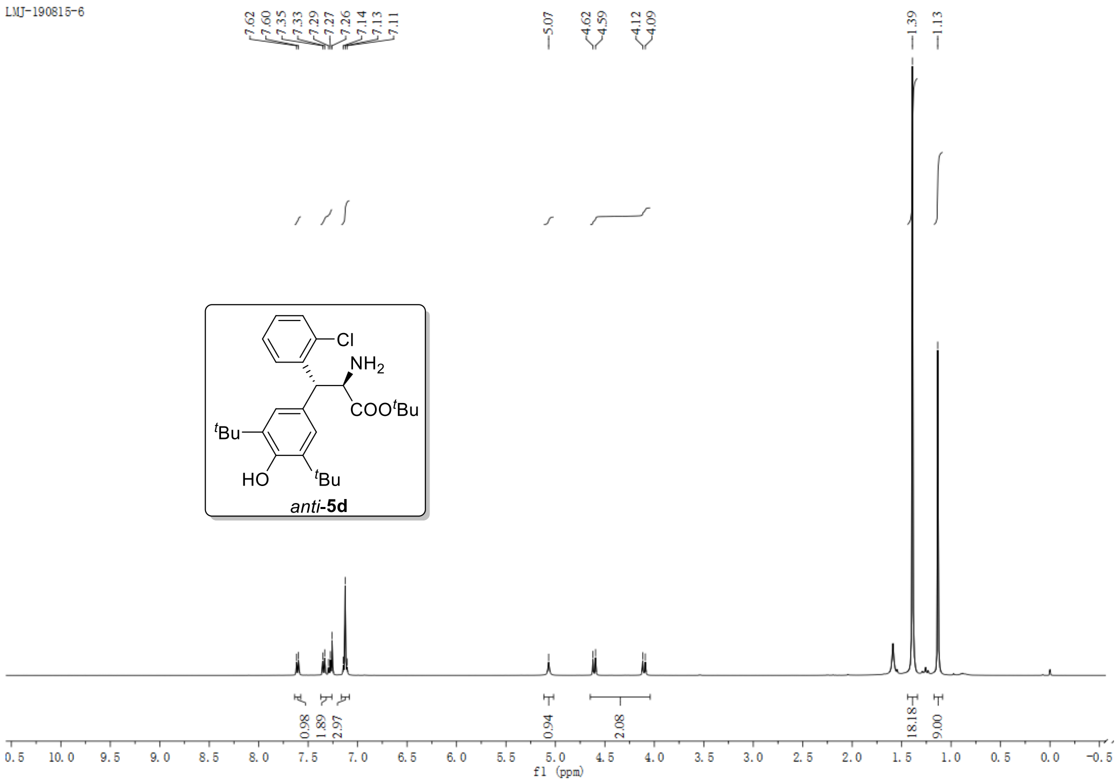
LMJ-190815-6
7.62
7.60
7.35
7.33
7.29
7.27
7.26
7.14
7.13
7.11
5.07
4.62
4.59
4.12
4.09
1.39
1.13
NH2
Cl
COOtBu
tBu
HO
tBu
anti-5d
0.98
1.89
2.97
0.94
2.08
18.18
9.00
f1 (ppm)

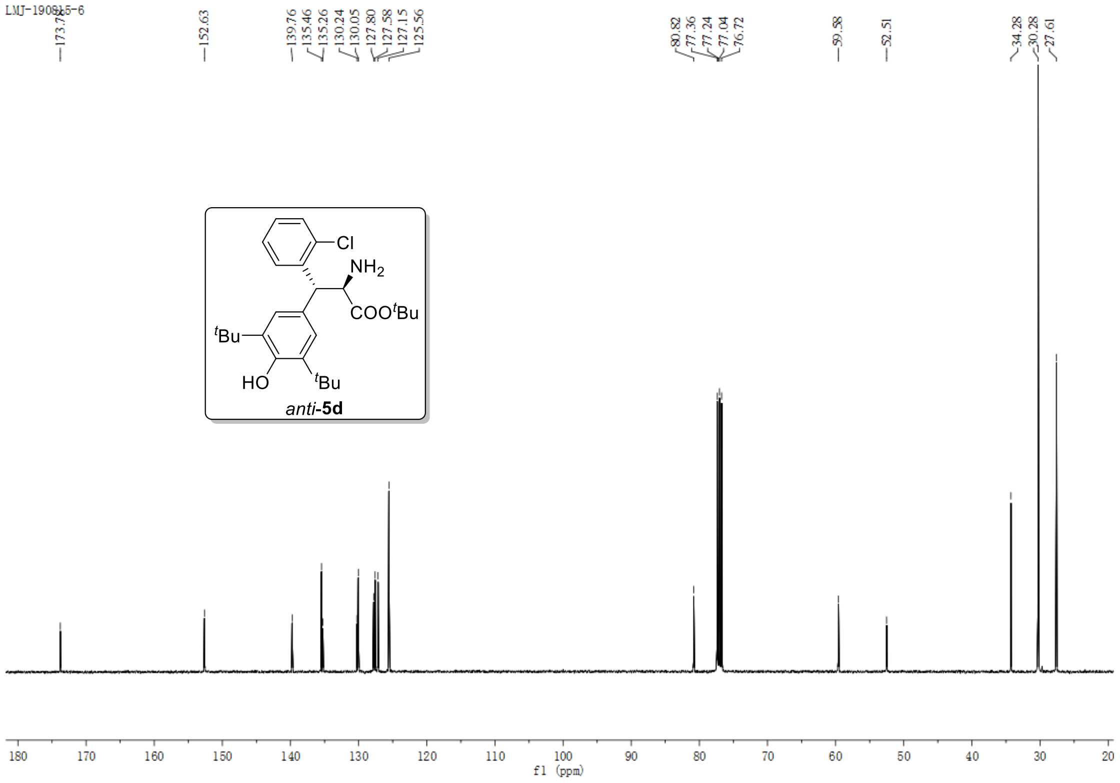
LMJ-190815-6
173.78
152.63
139.76
135.46
135.26
130.24
130.05
127.80
127.58
127.15
125.56
80.82
77.36
77.24
77.04
76.72
59.58
52.51
34.28
30.28
27.61
NH2
Cl
COOtBu
tBu
HO
tBu
anti-5d
f1 (ppm)

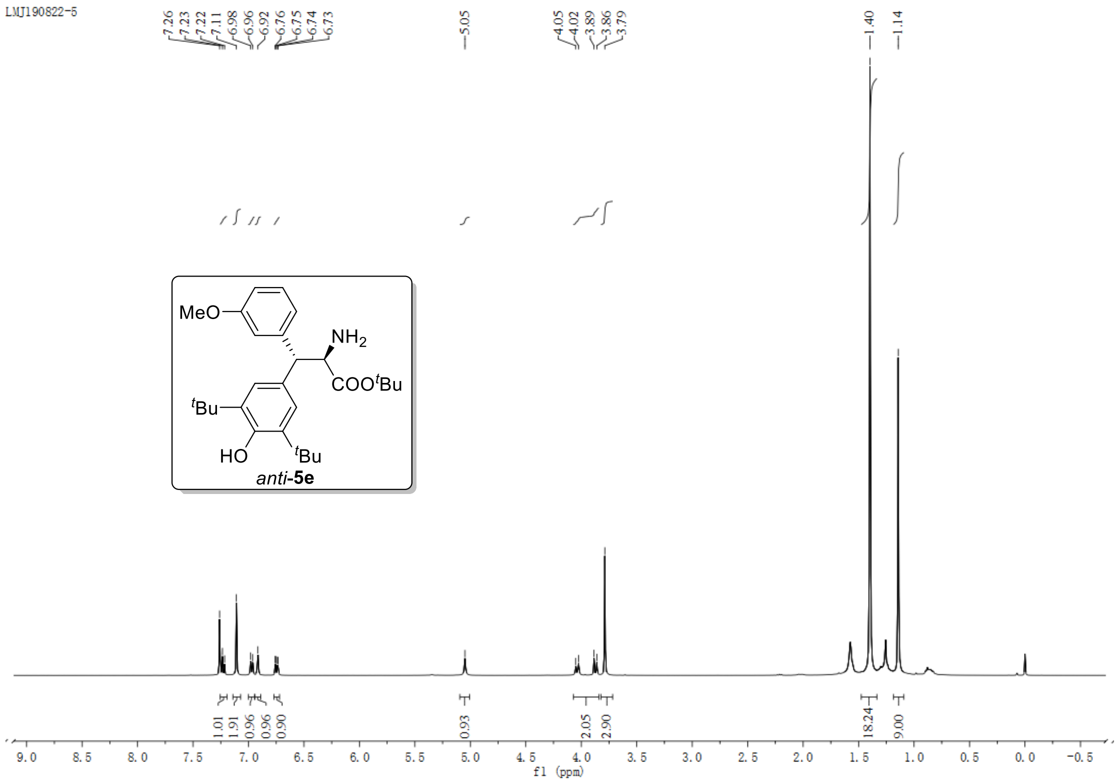

LMJ190822-5
7.26
7.23
7.22
7.11
6.98
6.96
6.92
6.76
6.75
6.74
6.73
5.05
4.05
4.02
3.89
3.86
3.79
1.40
1.14
MeO
NH2
COOtBu
tBu
HO
tBu
anti-5e
1.01
1.91
0.96
0.96
0.90
0.93
2.05
2.90
18.24
9.00
9.0 8.5 8.0 7.5 7.0 6.5 6.0 5.5 5.0 4.5 4.0 3.5 3.0 2.5 2.0 1.5 1.0 0.5 0.0 -0.5
f1 (ppm)

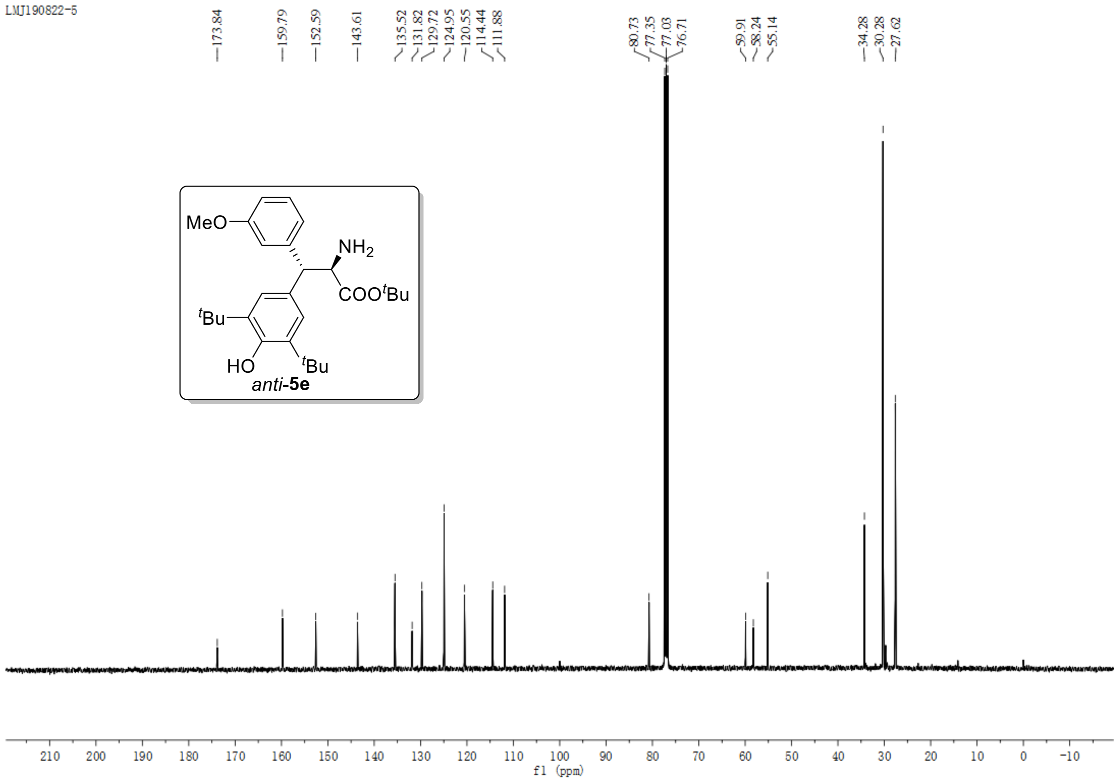

LMJ190822-5
173.84
159.79
152.59
143.61
135.52
131.82
129.72
124.95
120.55
114.44
111.88
80.73
77.35
77.03
76.71
59.91
58.24
55.14
34.28
30.28
27.62
MeO
NH2
COOtBu
tBu
HO
tBu
anti-5e
210 200 190 180 170 160 150 140 130 120 110 100 90 80 70 60 50 40 30 20 10 0 -10
f1 (ppm)

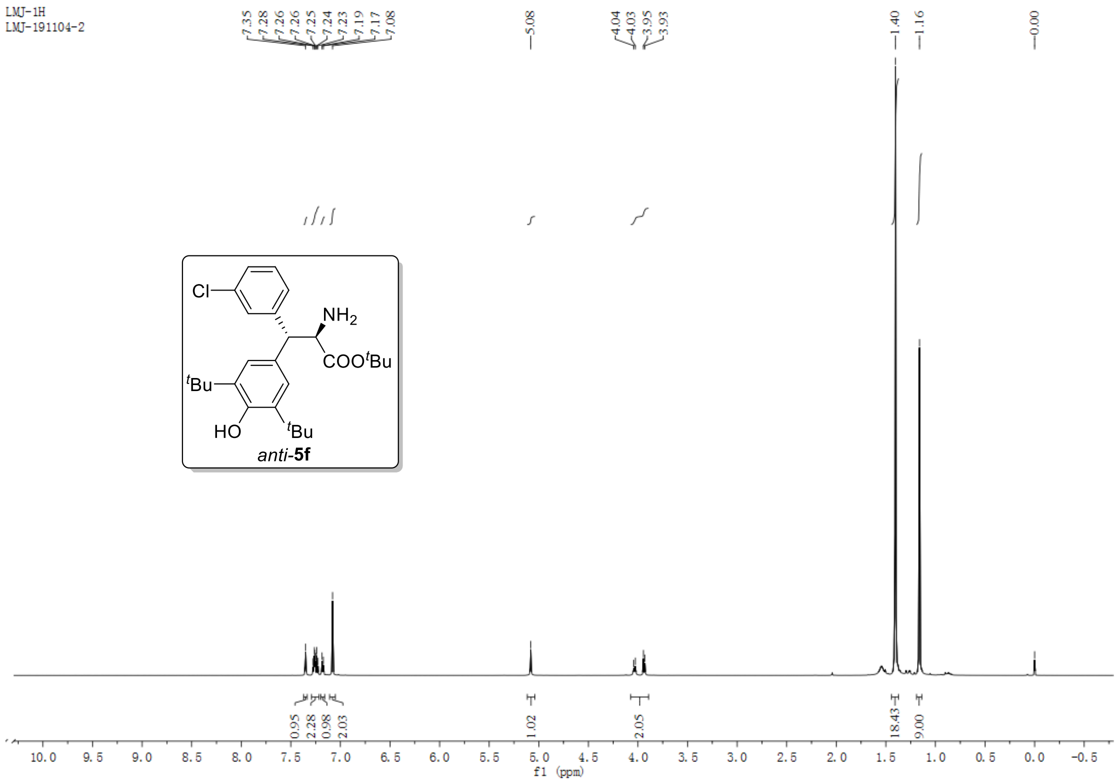
LMJ-1H
LMJ-191104-2
7.35
7.28
7.26
7.26
7.25
7.24
7.23
7.19
7.17
7.08
5.08
4.04
4.03
3.95
3.93
1.40
1.16
0.00
Cl
NH2
COO^{t}Bu
^{t}Bu
HO
^{t}Bu
anti-5f
0.95
2.28
0.98
2.03
1.02
2.05
18.43
9.00
f1 (ppm)

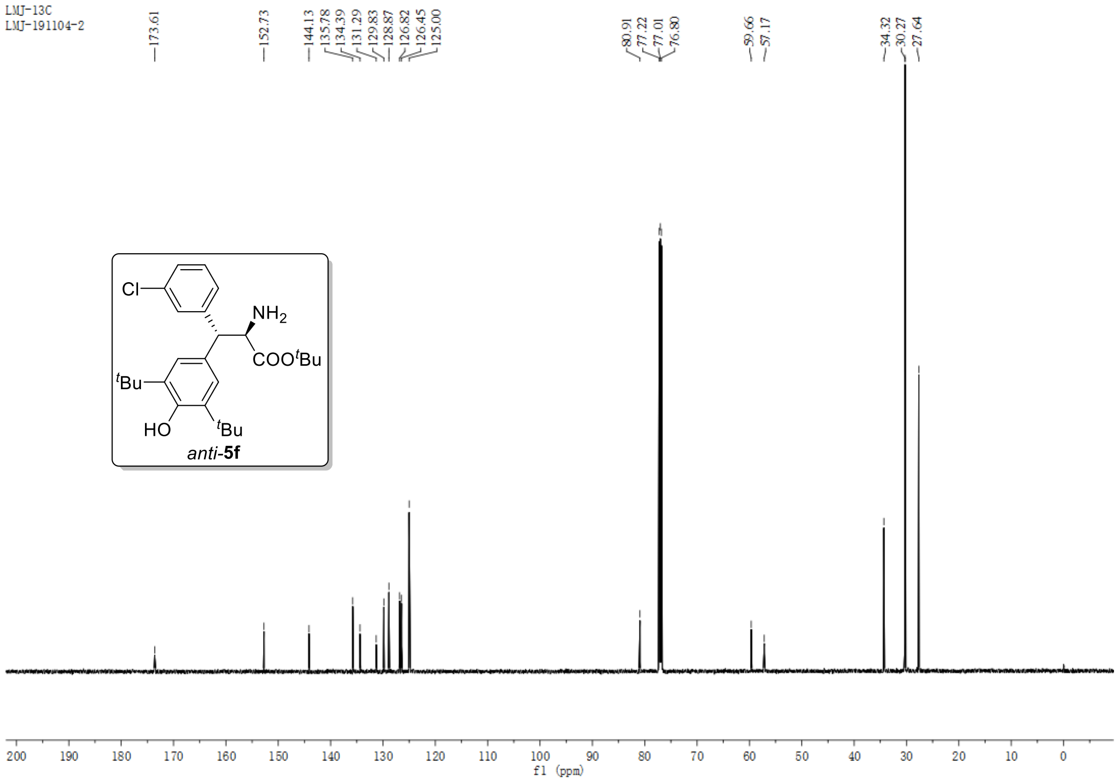
LMJ-13C
LMJ-191104-2
173.61
152.73
144.13
135.78
134.39
131.29
129.83
128.87
126.82
126.45
125.00
80.91
77.22
77.01
76.80
59.66
57.17
34.32
30.27
27.64
Cl
NH2
COO^{t}Bu
^{t}Bu
HO
^{t}Bu
anti-5f
f1 (ppm)

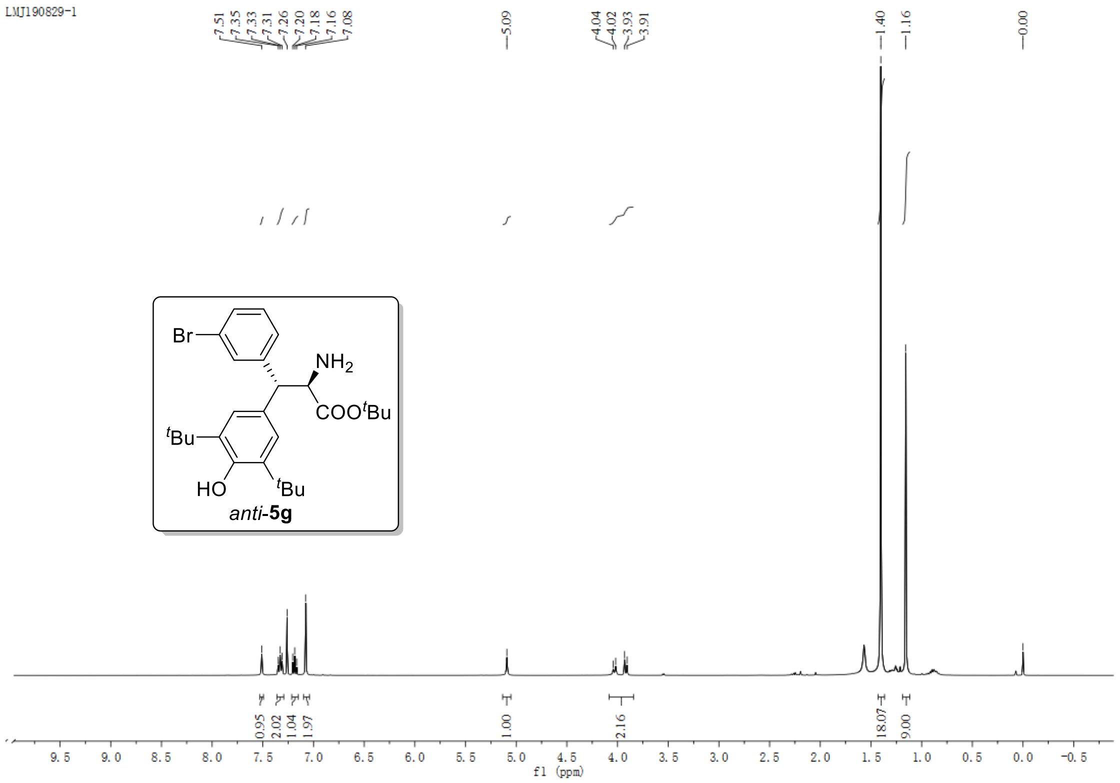
LMJ190829-1
7.51
7.35
7.33
7.31
7.26
7.20
7.18
7.16
7.08
5.09
4.04
4.02
3.93
3.91
1.40
1.16
0.00
Br
NH2
COOtBu
tBu
HO
tBu
anti-5g
0.95
2.02
1.04
1.97
1.00
2.16
18.07
9.00
f1 (ppm)

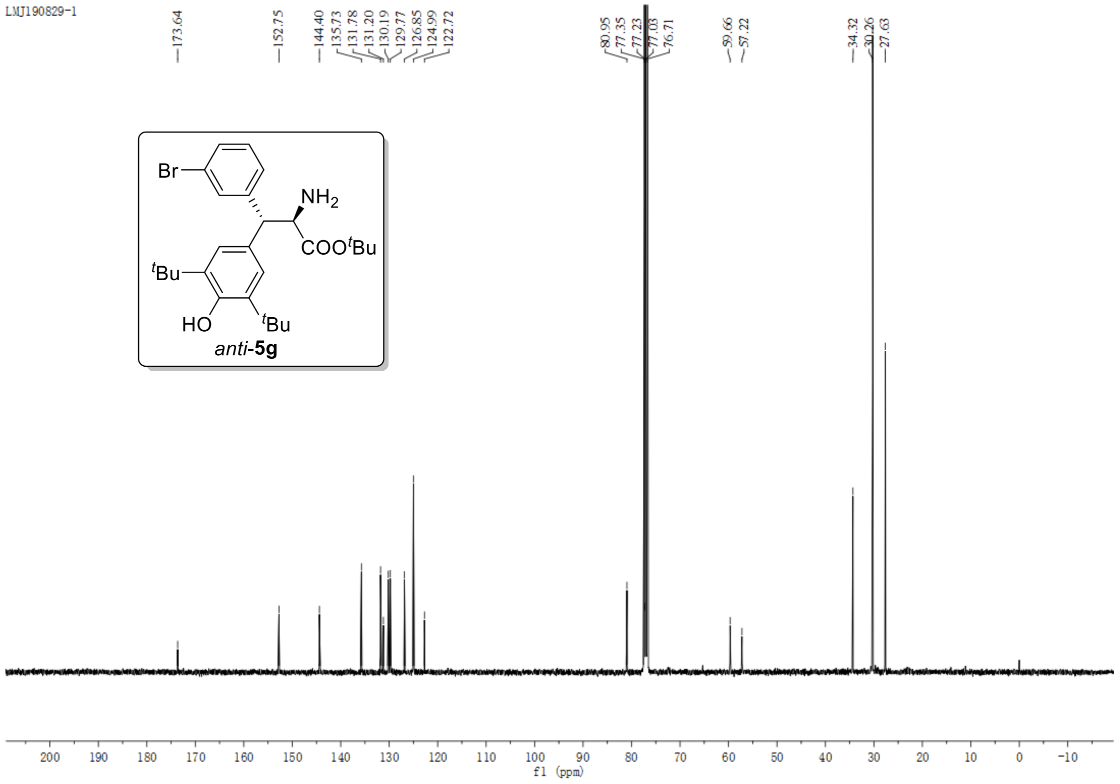
LMJ190829-1
173.64
152.75
144.40
135.73
131.78
131.20
130.19
129.77
126.85
124.99
122.72
80.95
77.35
77.23
77.03
76.71
59.66
57.22
34.32
30.26
27.63
Br
NH2
COOtBu
tBu
HO
tBu
anti-5g
f1 (ppm)

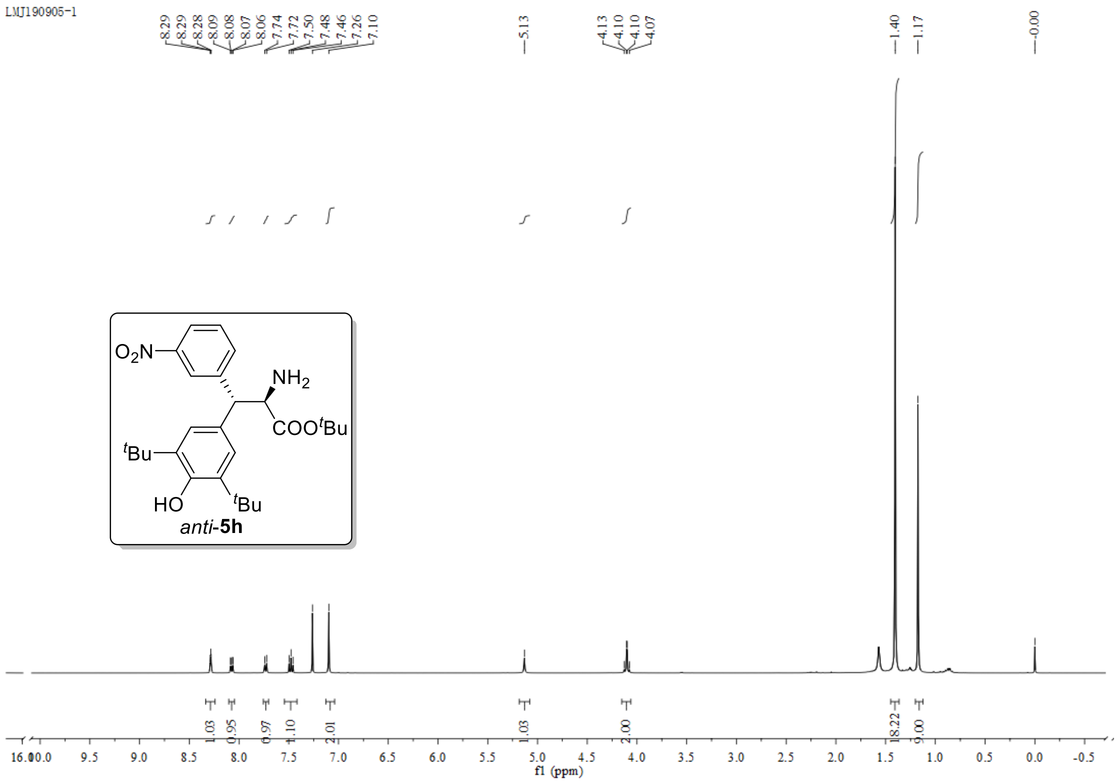
LMJ190905-1
8.29
8.29
8.28
8.09
8.08
8.07
8.06
7.74
7.72
7.50
7.48
7.46
7.26
7.10
5.13
4.13
4.10
4.10
4.07
1.40
1.17
0.00
O_2N
NH_2
COO^tBu
^tBu
HO
^tBu
anti-**5h**
1.03
0.95
0.97
1.10
2.01
1.03
2.00
18.22
9.00
f1 (ppm)

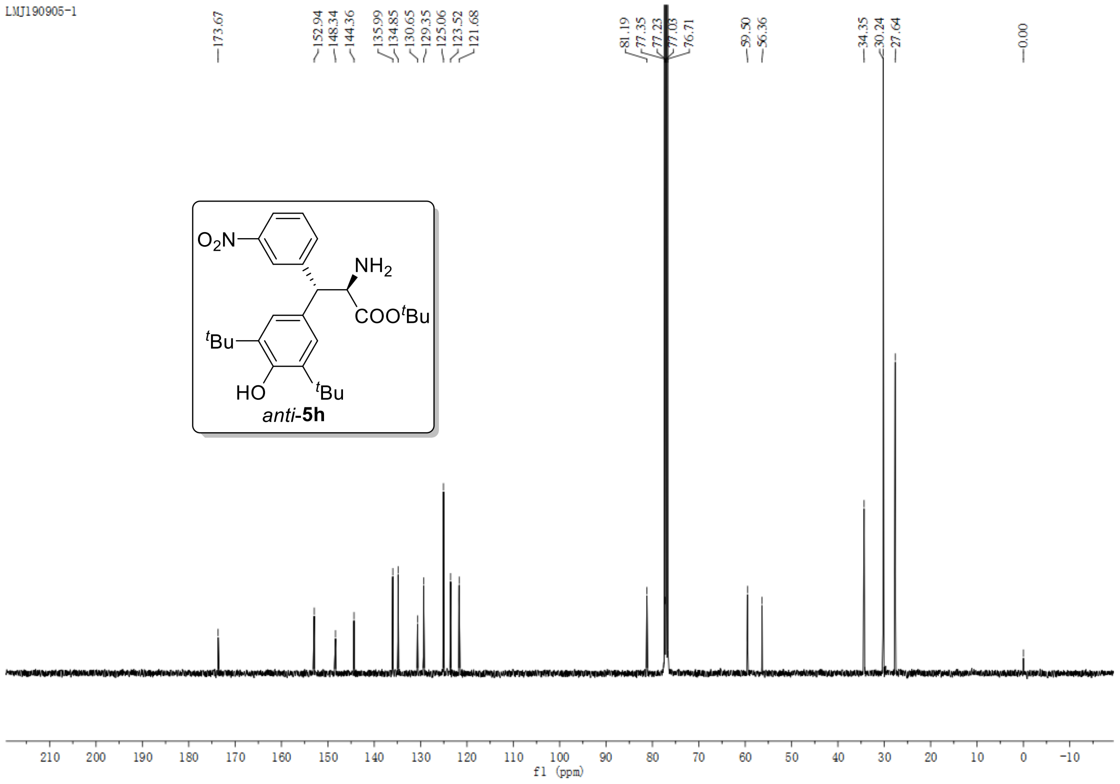
LMJ190905-1
173.67
152.94
148.34
144.36
135.99
134.85
130.65
129.35
125.06
123.52
121.68
81.19
77.35
77.23
77.03
76.71
59.50
56.36
34.35
30.24
27.64
0.00
O_2N
NH_2
COO^tBu
^tBu
HO
^tBu
anti-**5h**
f1 (ppm)

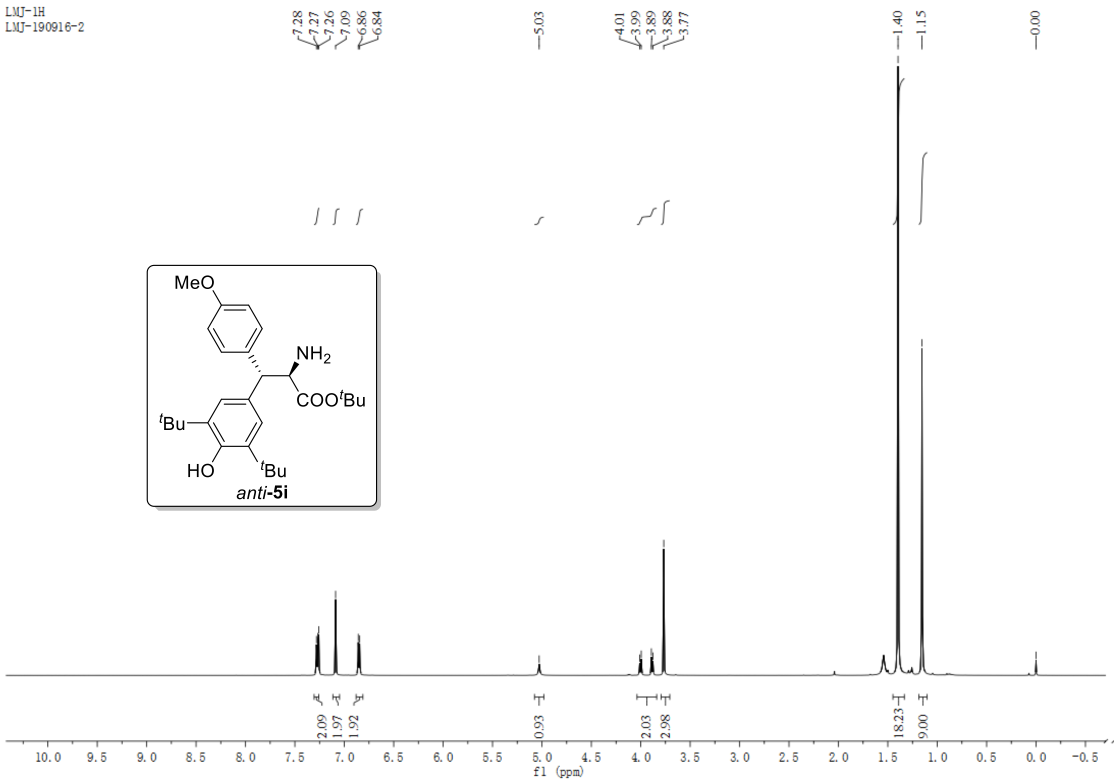
LMJ-1H
LMJ-190916-2
MeO
NH2
COOtBu
tBu
HO
tBu
anti-5i
f1 (ppm)

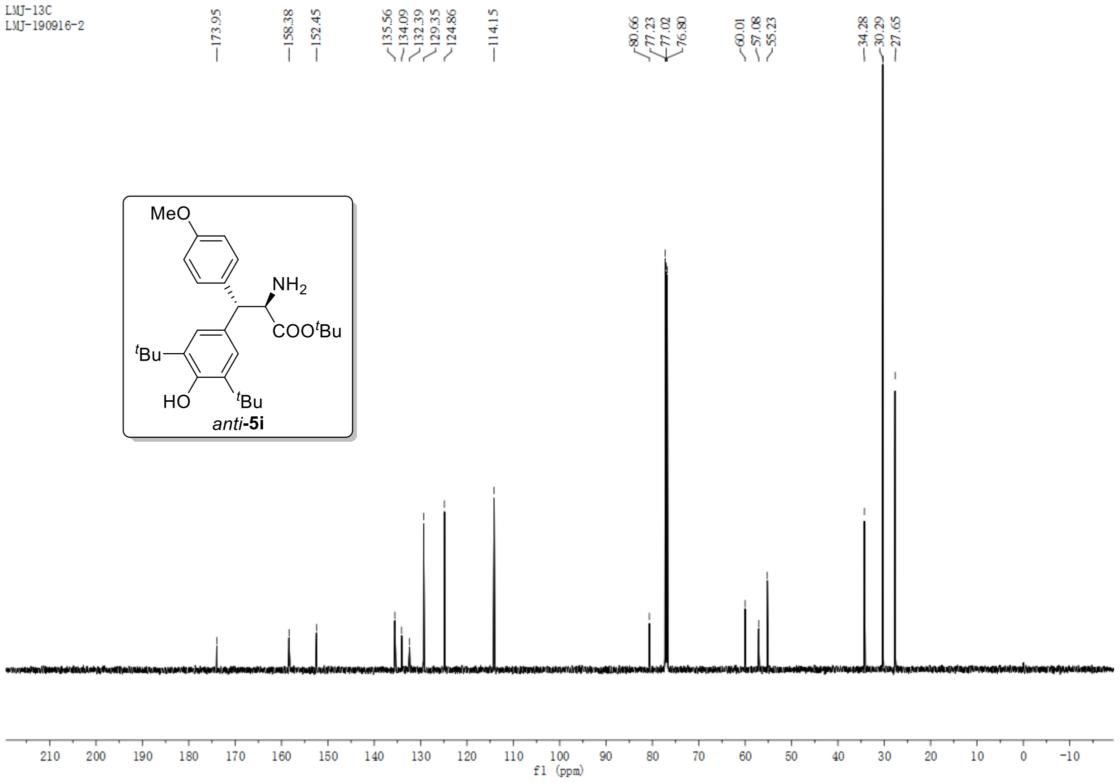
LMJ-13C
LMJ-190916-2
MeO
NH2
COOtBu
tBu
HO
tBu
anti-5i
f1 (ppm)

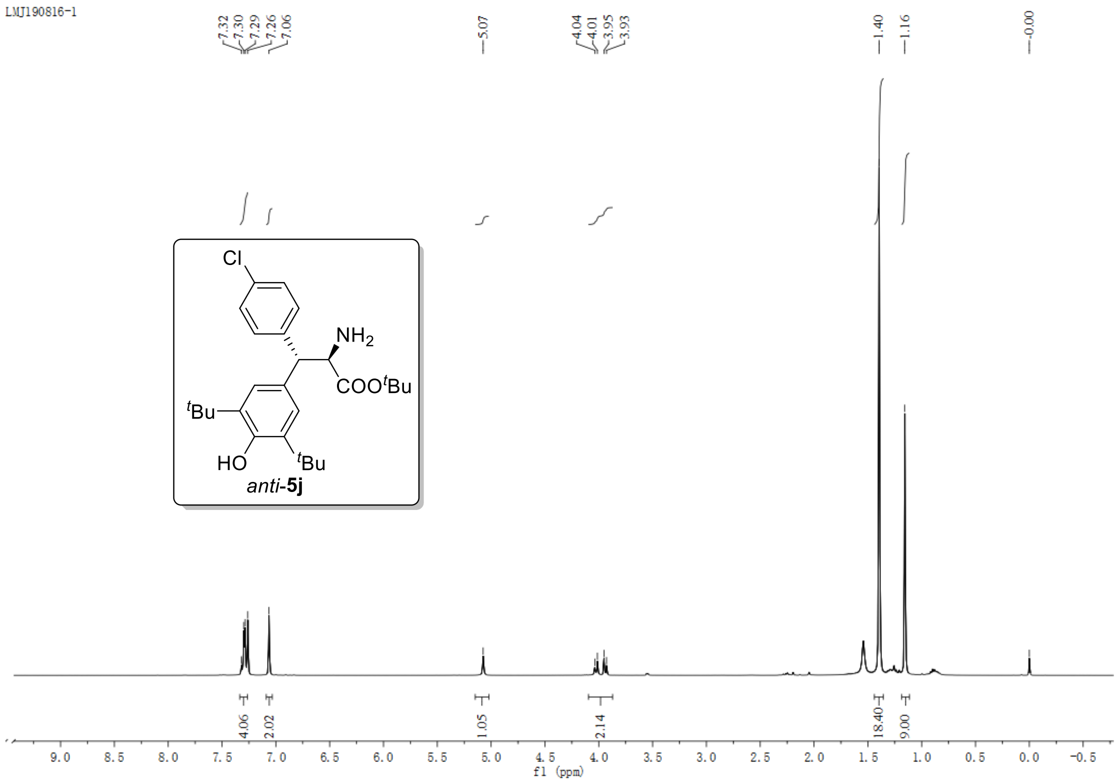
LMJ190816-1
7.32
7.30
7.29
7.26
7.06
5.07
4.04
4.01
3.95
3.93
1.40
1.16
0.00
Cl
NH2
COOtBu
tBu
HO
tBu
anti-5j
4.06
2.02
1.05
2.14
18.40
9.00
f1 (ppm)

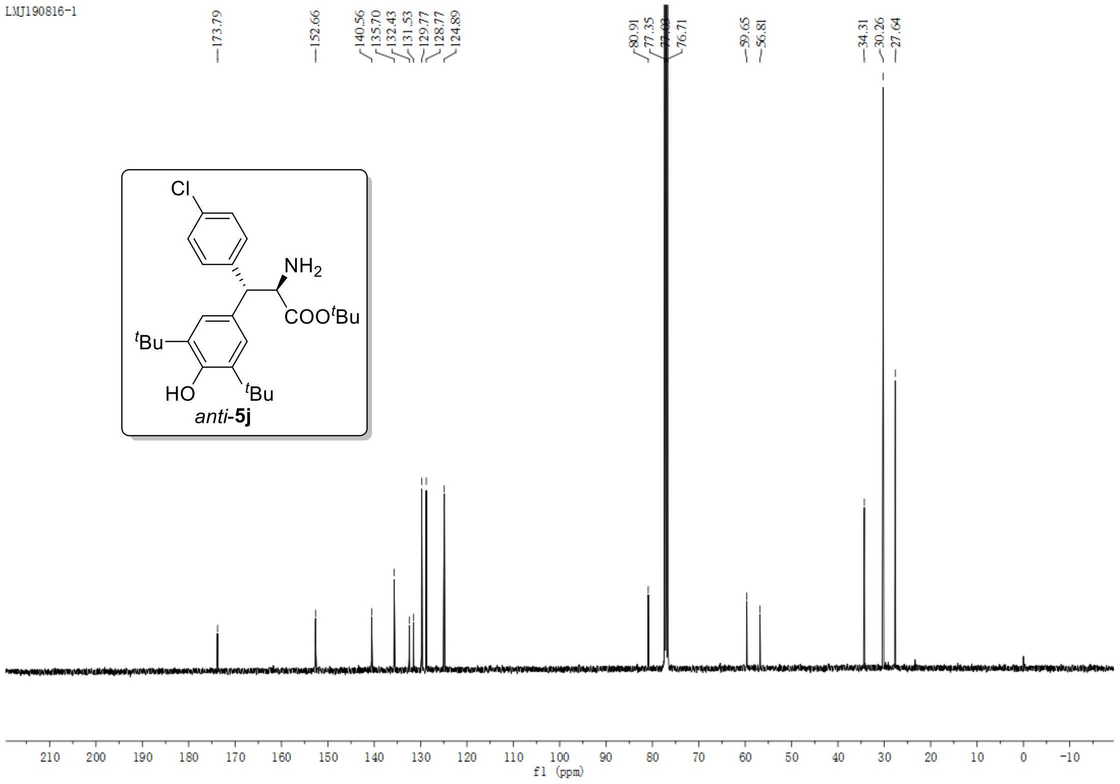
LMJ190816-1
173.79
152.66
140.56
135.70
132.43
131.53
129.77
128.77
124.89
80.91
77.35
76.71
59.65
56.81
34.31
30.26
27.64
Cl
NH2
COOtBu
tBu
HO
tBu
anti-5j
f1 (ppm)

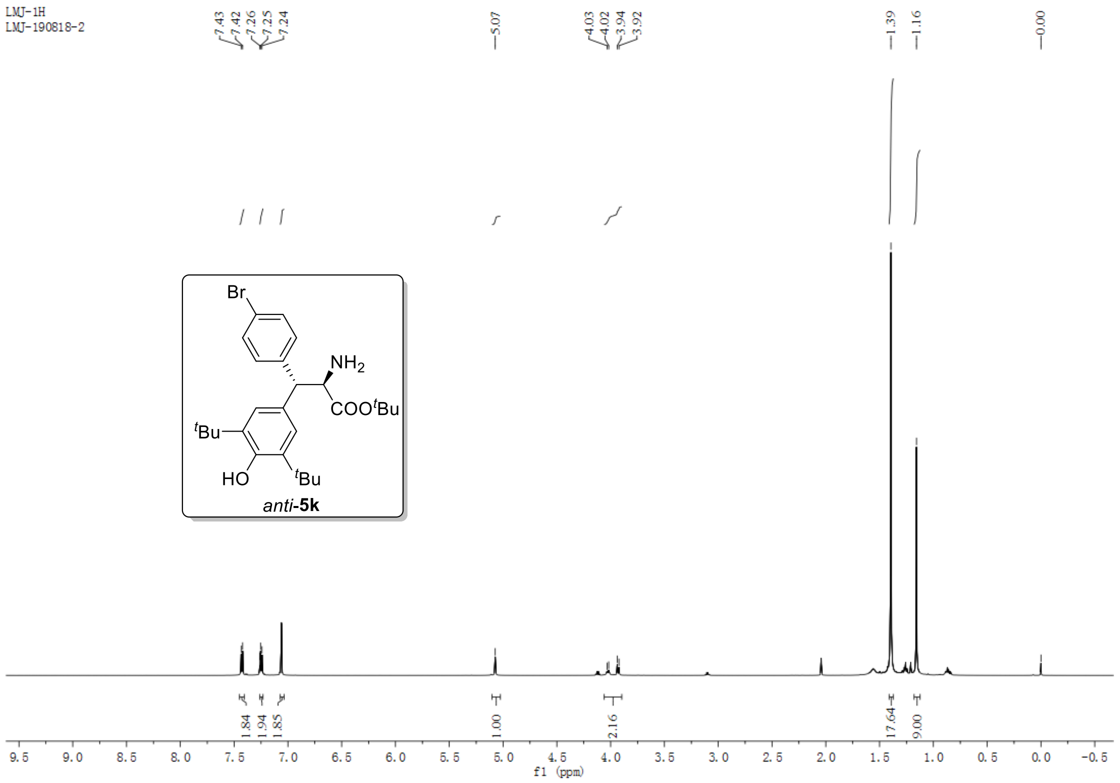
LMJ-1H
LMJ-190818-2
7.43
7.42
7.26
7.25
7.24
5.07
4.03
4.02
3.94
3.92
1.39
1.16
0.00
Br
NH2
COOtBu
tBu
HO
tBu
anti-5k
1.84
1.94
1.85
1.00
2.16
17.64
9.00
f1 (ppm)

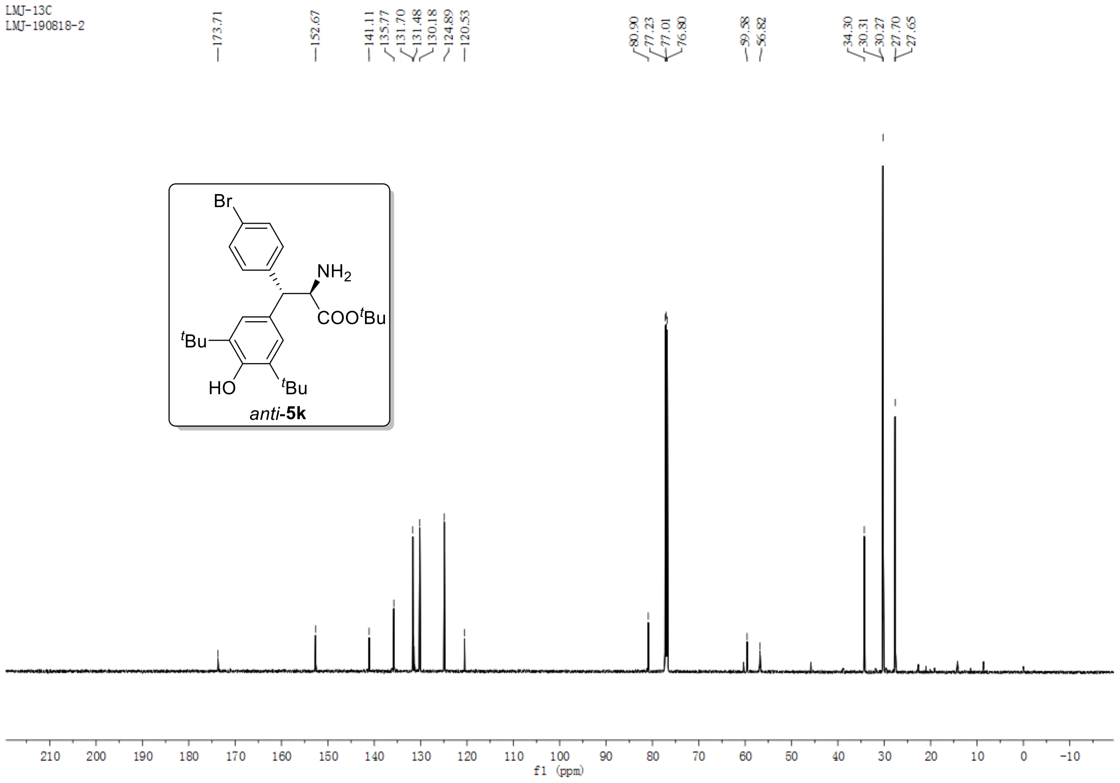
LMJ-13C
LMJ-190818-2
173.71
152.67
141.11
135.77
131.70
131.48
130.18
124.89
120.53
80.90
77.23
77.01
76.80
59.58
56.82
34.30
30.31
30.27
27.70
27.65
Br
NH2
COOtBu
tBu
HO
tBu
anti-5k
f1 (ppm)

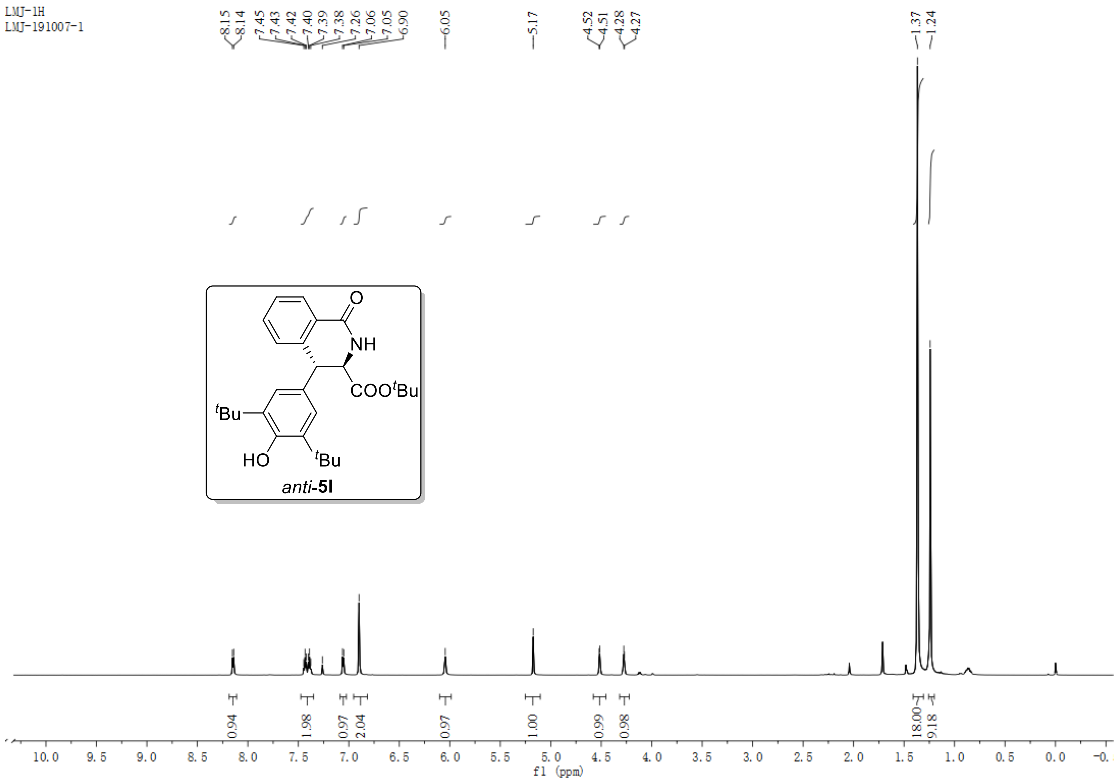
LMJ-1H
LMJ-191007-1
8.15
8.14
7.45
7.43
7.42
7.40
7.39
7.38
7.26
7.06
7.05
6.90
6.05
5.17
4.52
4.51
4.28
4.27
1.37
1.24
O
NH
COO^{t}Bu
^{t}Bu
HO
^{t}Bu
anti-**5l**
0.94
1.98
0.97
2.04
0.97
1.00
0.99
0.98
18.00
9.18
f1 (ppm)

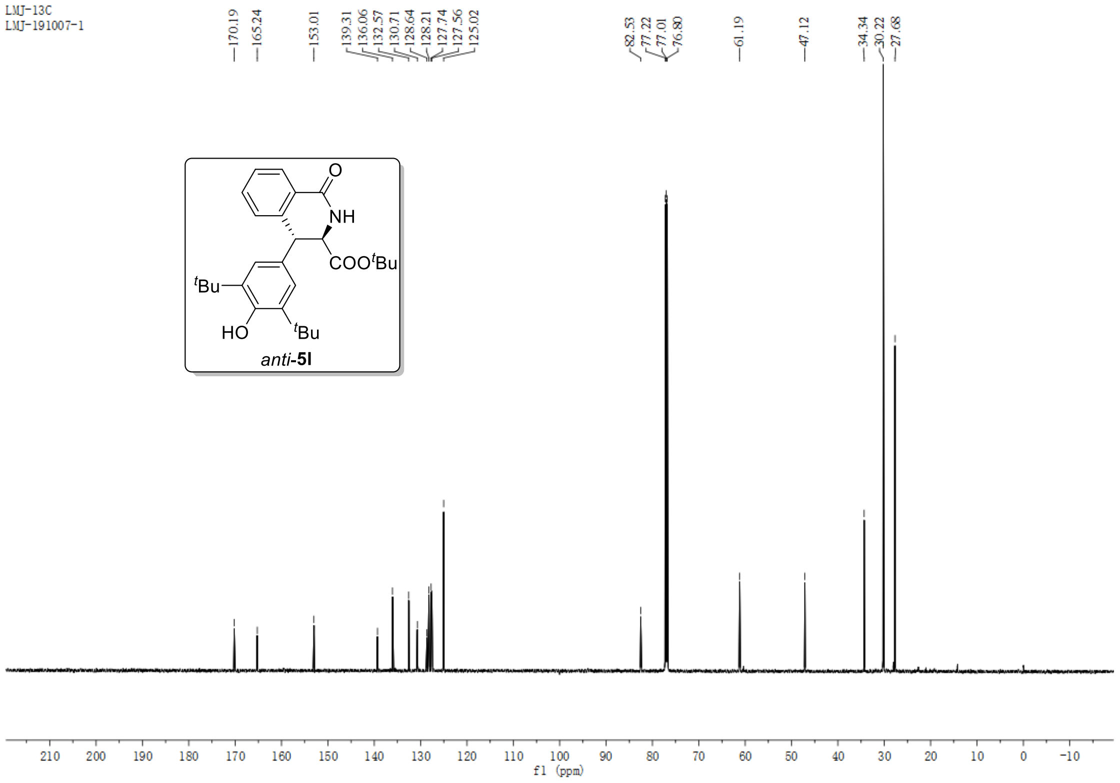
LMJ-13C
LMJ-191007-1
170.19
165.24
153.01
139.31
136.06
132.57
130.71
128.64
128.21
127.74
127.56
125.02
82.53
77.22
77.01
76.80
61.19
47.12
34.34
30.22
27.68
O
NH
COO^{t}Bu
^{t}Bu
HO
^{t}Bu
anti-**5l**
f1 (ppm)

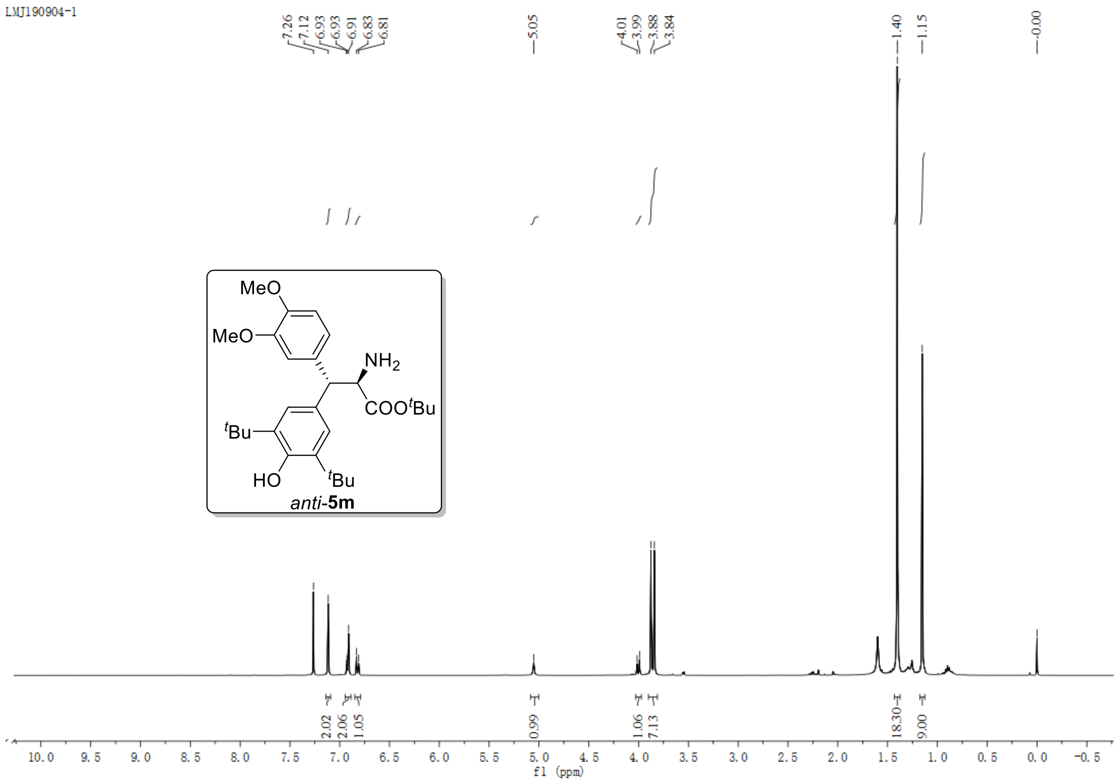
LMJ190904-1
7.26
7.12
6.93
6.93
6.91
6.83
6.81
5.05
4.01
3.99
3.88
3.84
1.40
1.15
0.00
MeO
MeO
NH2
COOtBu
tBu
HO
tBu
anti-5m
2.02
2.06
1.05
0.99
1.06
7.13
18.30
9.00
f1 (ppm)

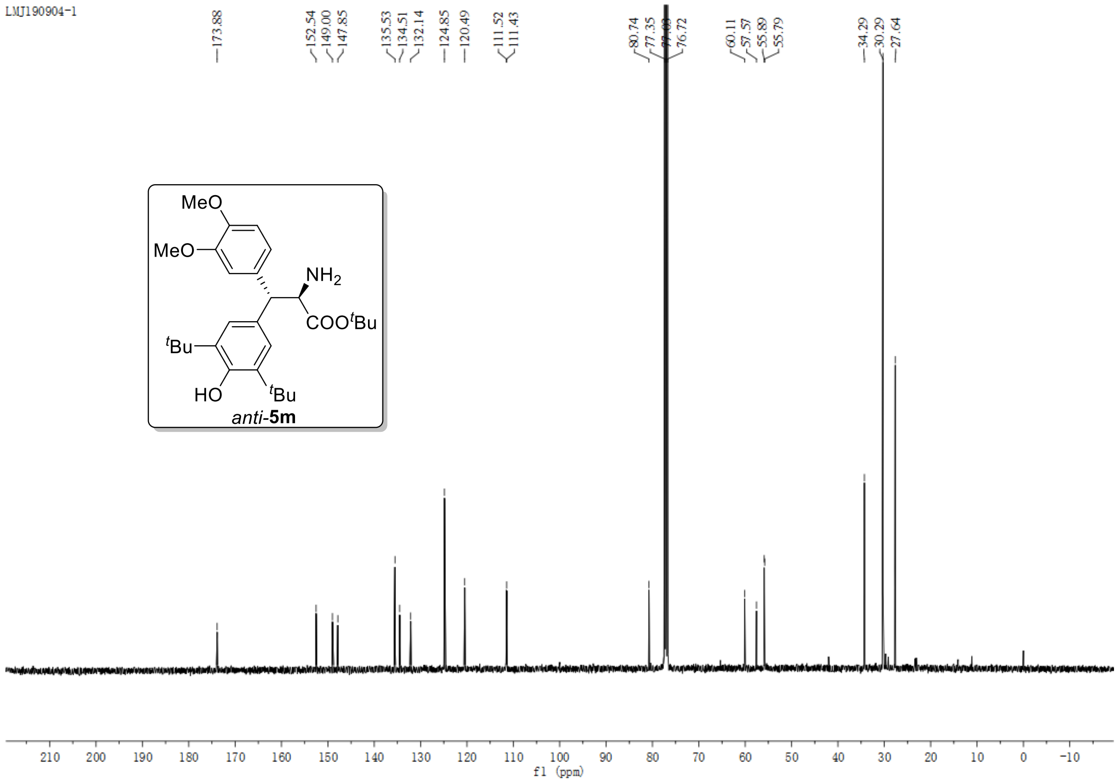
LMJ190904-1
173.88
152.54
149.00
147.85
135.53
134.51
132.14
124.85
120.49
111.52
111.43
80.74
77.35
76.72
60.11
57.57
55.89
55.79
34.29
30.29
27.64
MeO
MeO
NH2
COOtBu
tBu
HO
tBu
anti-5m
f1 (ppm)

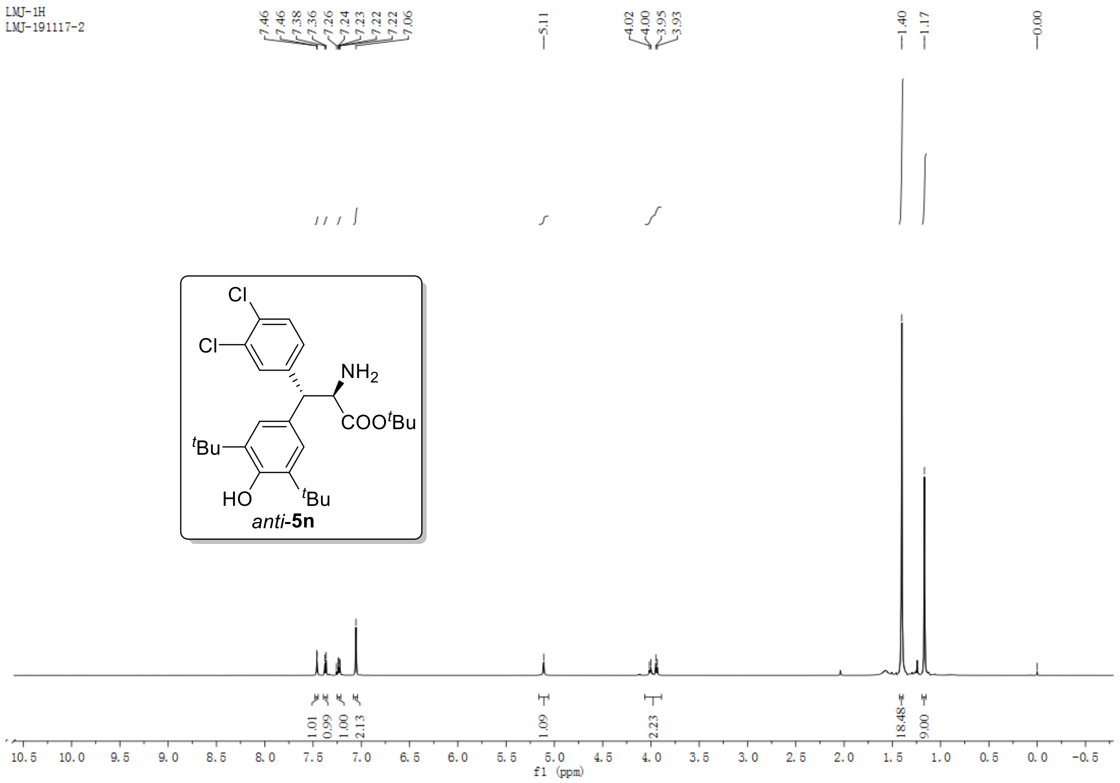
LMJ-1H
LMJ-191117-2
anti-5n
f1 (ppm)

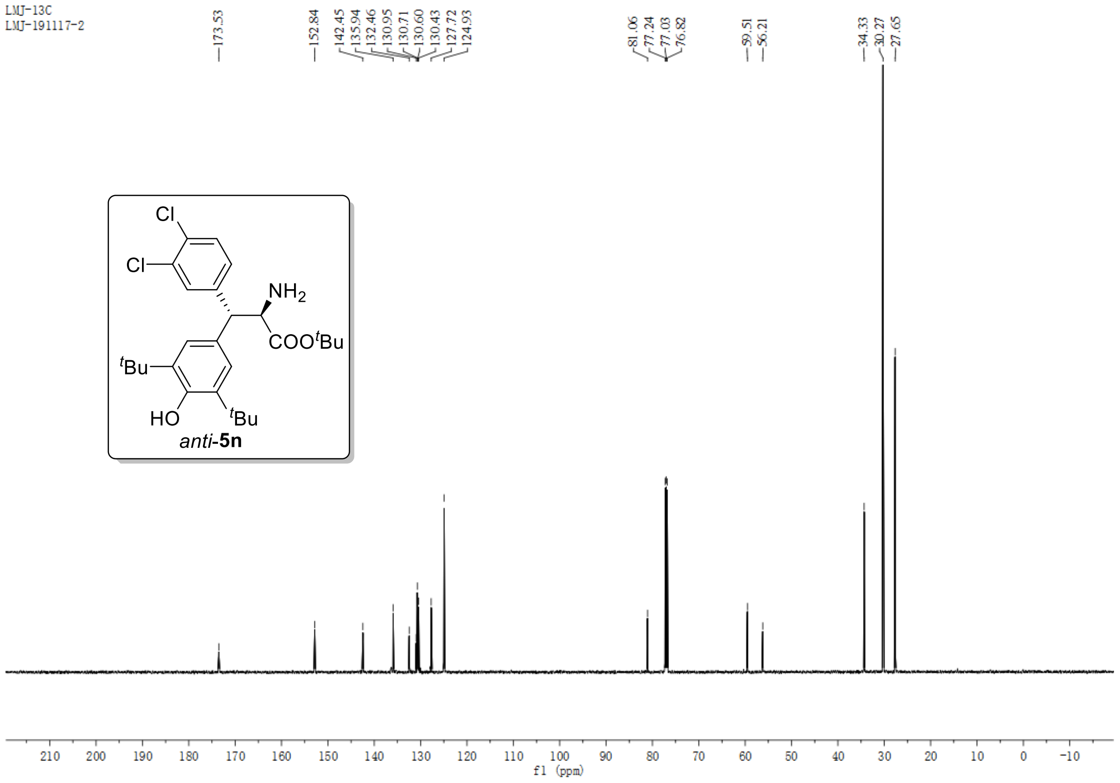
LMJ-13C
LMJ-191117-2
anti-5n
f1 (ppm)

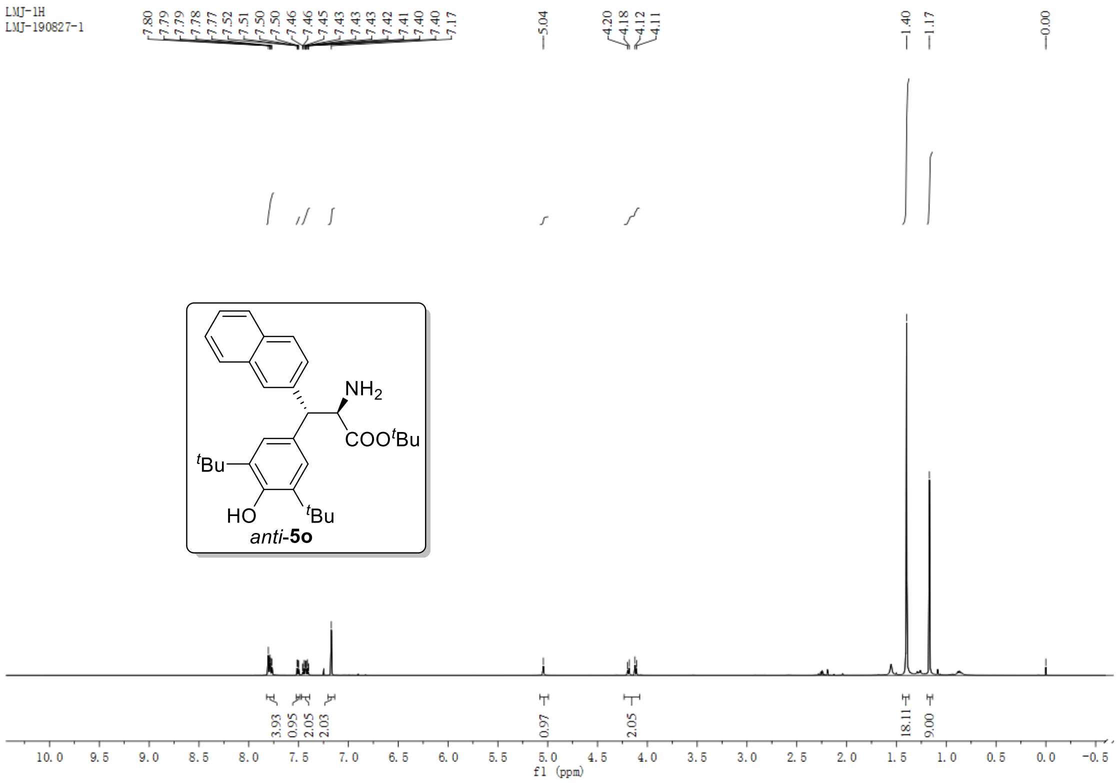
LMJ-1H
LMJ-190827-1
anti-5o
f1 (ppm)

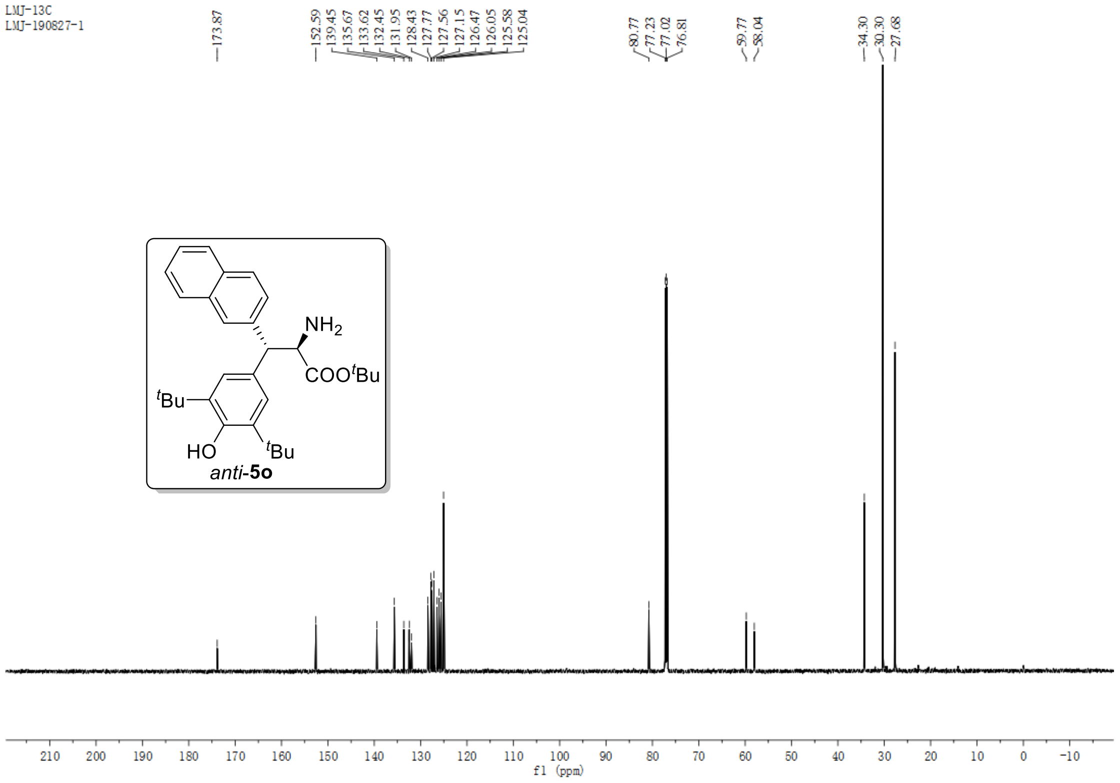
LMJ-13C
LMJ-190827-1
anti-5o
f1 (ppm)

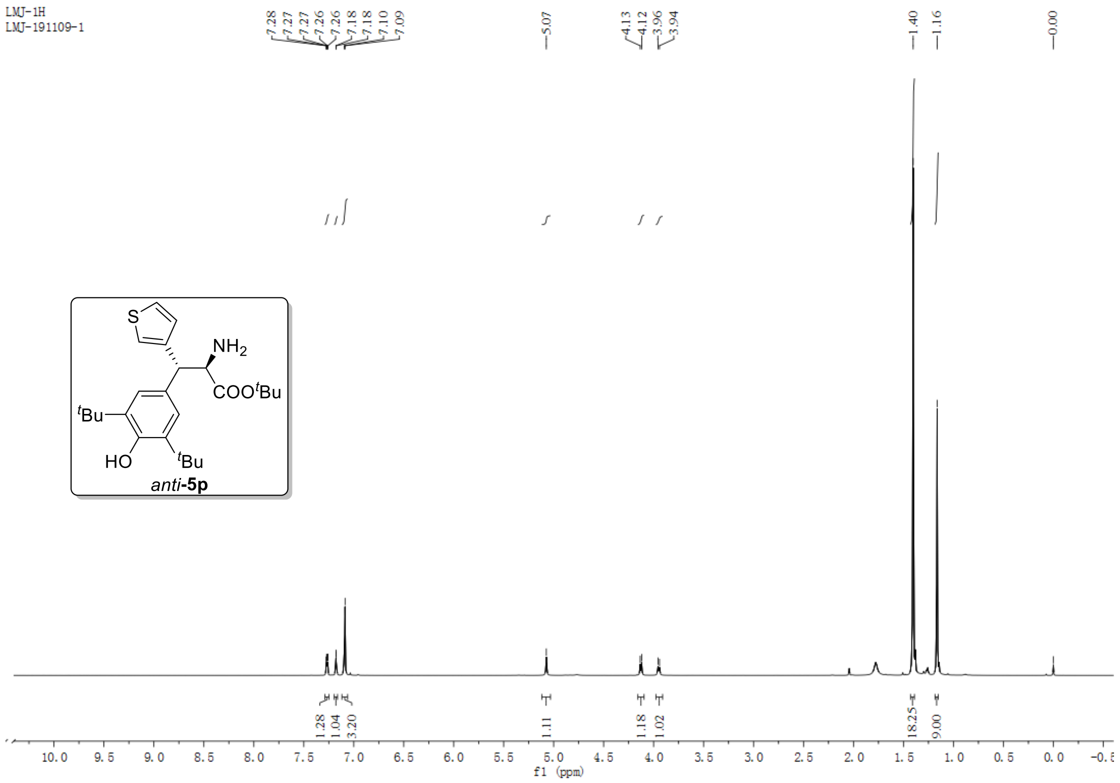

LMJ-1H
LMJ-191109-1
7.28
7.27
7.27
7.26
7.26
7.18
7.18
7.10
7.09
5.07
4.13
4.12
3.96
3.94
1.40
1.16
0.00
NH2
COOtBu
tBu
HO
tBu
S
anti-5p
1.28
1.04
3.20
1.11
1.18
1.02
18.25
9.00
f1 (ppm)

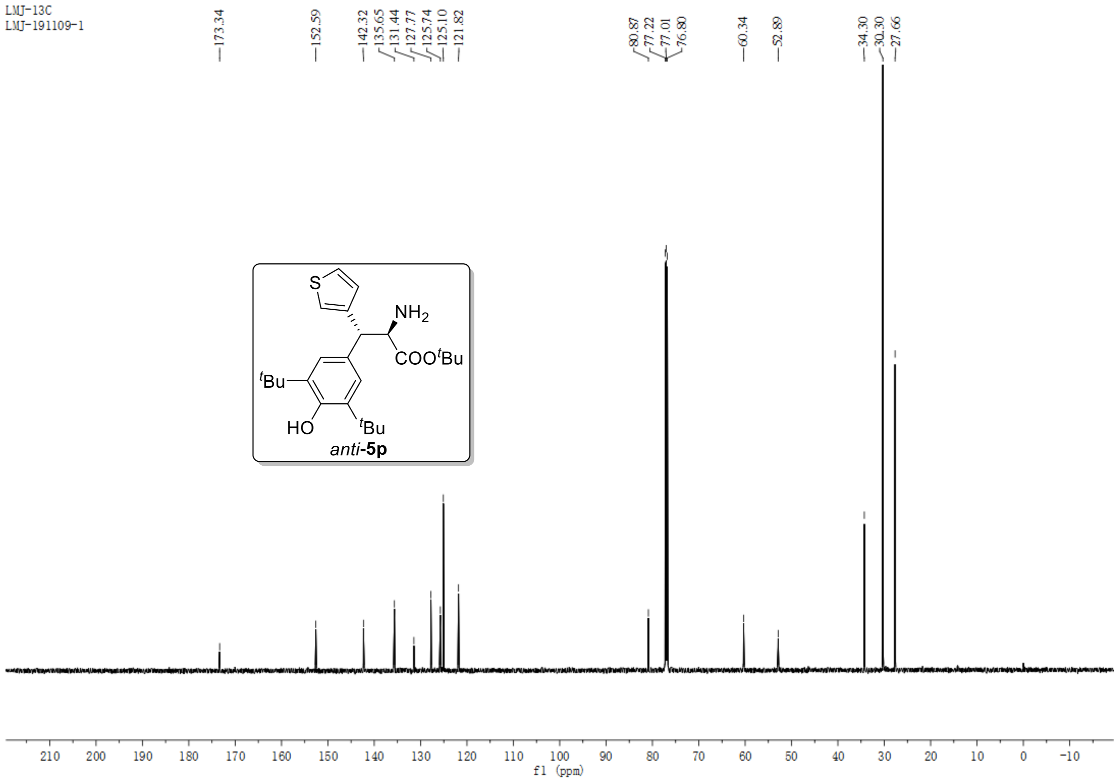

LMJ-13C
LMJ-191109-1
173.34
152.59
142.32
135.65
131.44
127.77
125.74
125.10
121.82
80.87
77.22
77.01
76.80
60.34
52.89
34.30
30.30
27.66
NH2
COOtBu
tBu
HO
tBu
S
anti-5p
f1 (ppm)

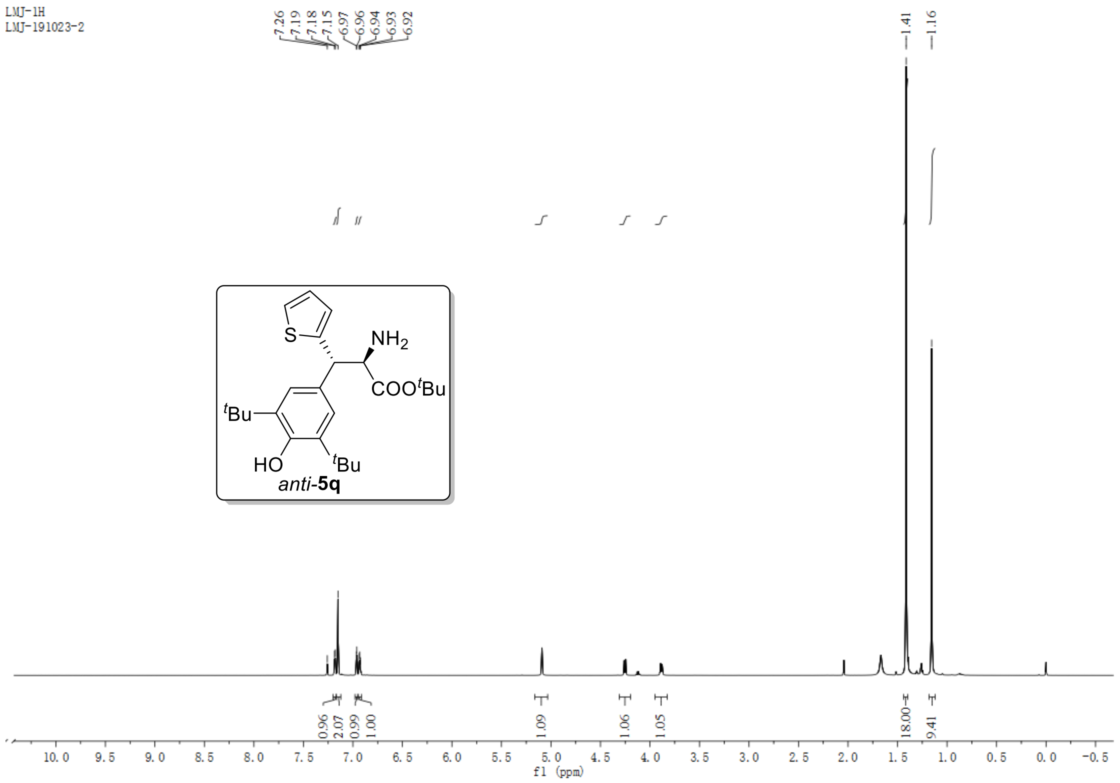
LMJ-1H
LMJ-191023-2
7.26 7.19 7.18 7.15 6.97 6.96 6.94 6.93 6.92
1.41 1.16
NH2
COO^{t}Bu
^{t}Bu
HO
^{t}Bu
S
anti-5q
0.96 2.07 0.99 1.00 1.09 1.06 1.05 18.00 9.41
f1 (ppm)

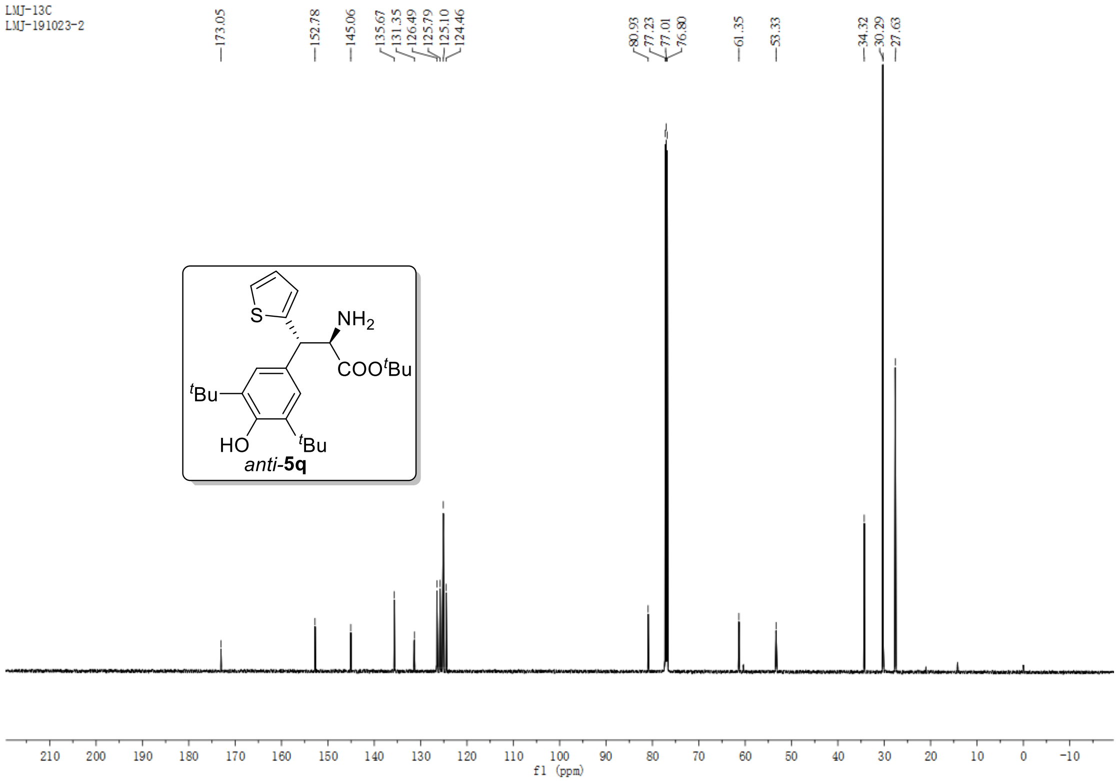
LMJ-13C
LMJ-191023-2
173.05 152.78 145.06 135.67 131.35 126.49 125.79 125.10 124.46 80.93 77.23 77.01 76.80 61.35 53.33 34.32 30.29 27.63
NH2
COO^{t}Bu
^{t}Bu
HO
^{t}Bu
S
anti-5q
f1 (ppm)

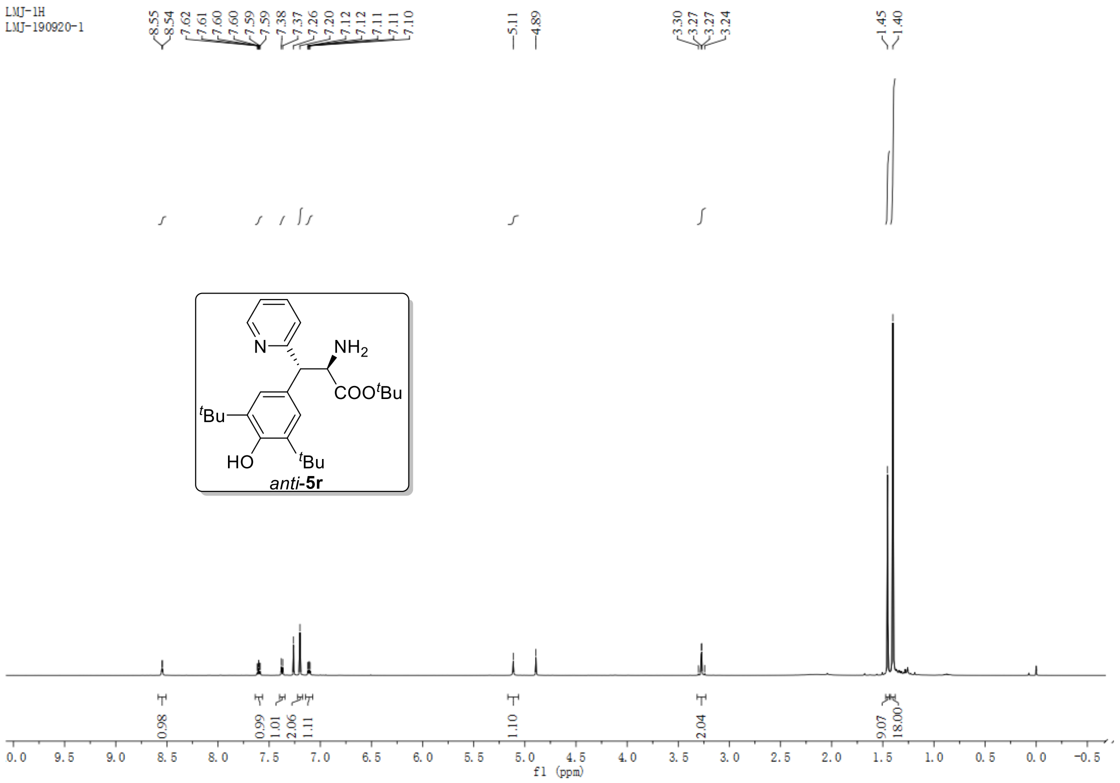
LMJ-1H
LMJ-190920-1
8.55
8.54
7.62
7.61
7.60
7.60
7.59
7.59
7.38
7.37
7.26
7.20
7.12
7.12
7.11
7.11
7.10
5.11
4.89
3.30
3.27
3.27
3.24
1.45
1.40
NH2
N
COO^{t}Bu
^{t}Bu
HO
^{t}Bu
anti-**5r**
0.98
0.99
1.01
2.06
1.11
1.10
2.04
9.07
18.00
f1 (ppm)

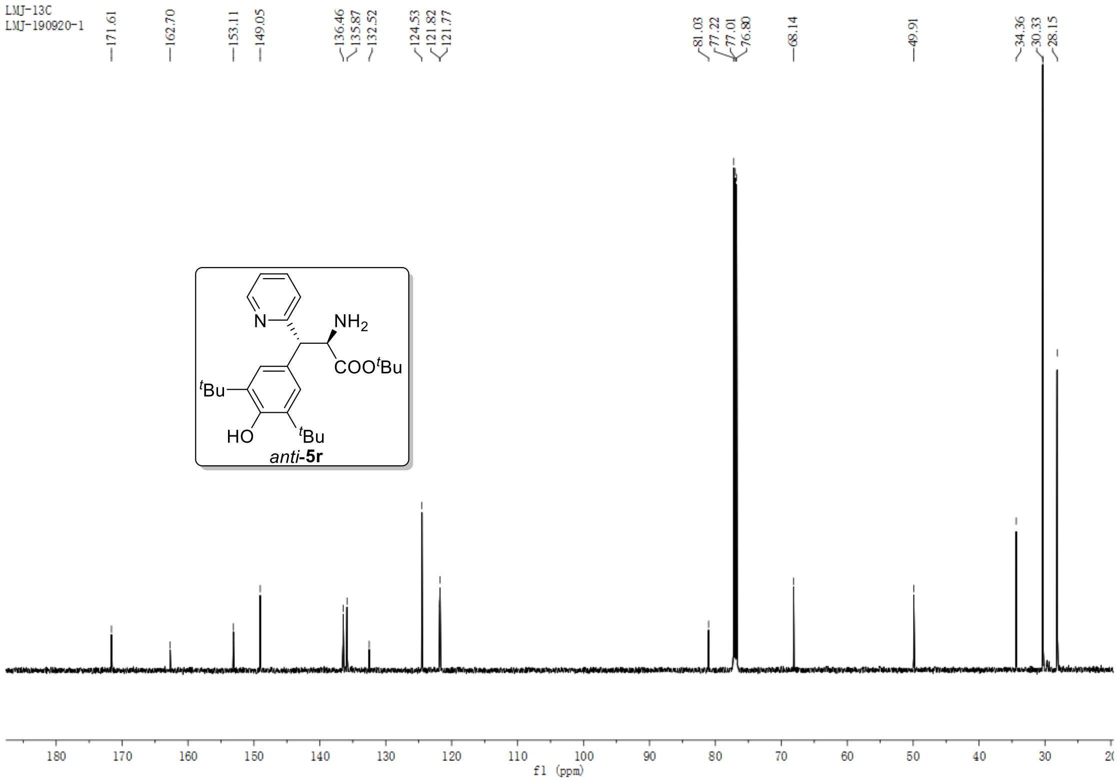
LMJ-13C
LMJ-190920-1
171.61
162.70
153.11
149.05
136.46
135.87
132.52
124.53
121.82
121.77
81.03
77.22
77.01
76.80
68.14
49.91
34.36
30.33
28.15
NH2
N
COO^{t}Bu
^{t}Bu
HO
^{t}Bu
anti-**5r**
f1 (ppm)

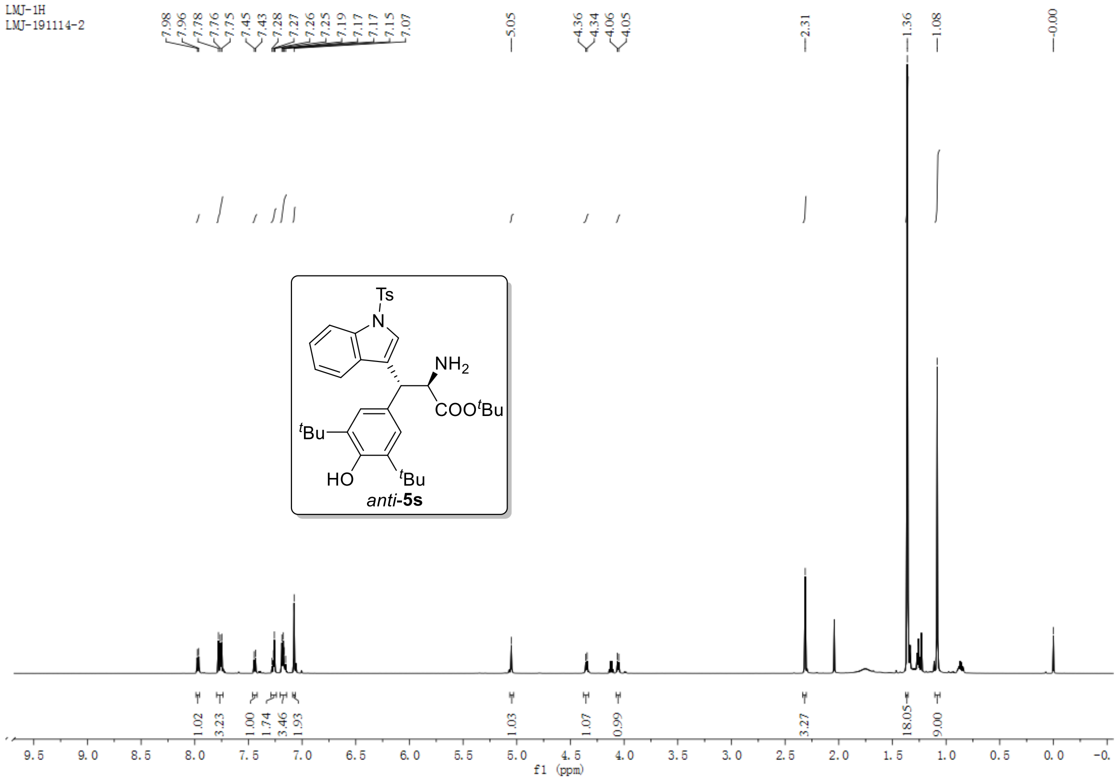
LMJ-1H
LMJ-191114-2
7.98 7.96 7.78 7.76 7.75 7.45 7.43 7.28 7.27 7.26 7.25 7.19 7.17 7.17 7.15 7.07
5.05
4.36 4.34 4.06 4.05
2.31
1.36
1.08
0.00
1.02 3.23 1.00 1.74 3.46 1.93 1.03 1.07 0.99 3.27 18.05 9.00
f1 (ppm)
Ts
N
NH2
COOtBu
tBu
HO
tBu
anti-5s

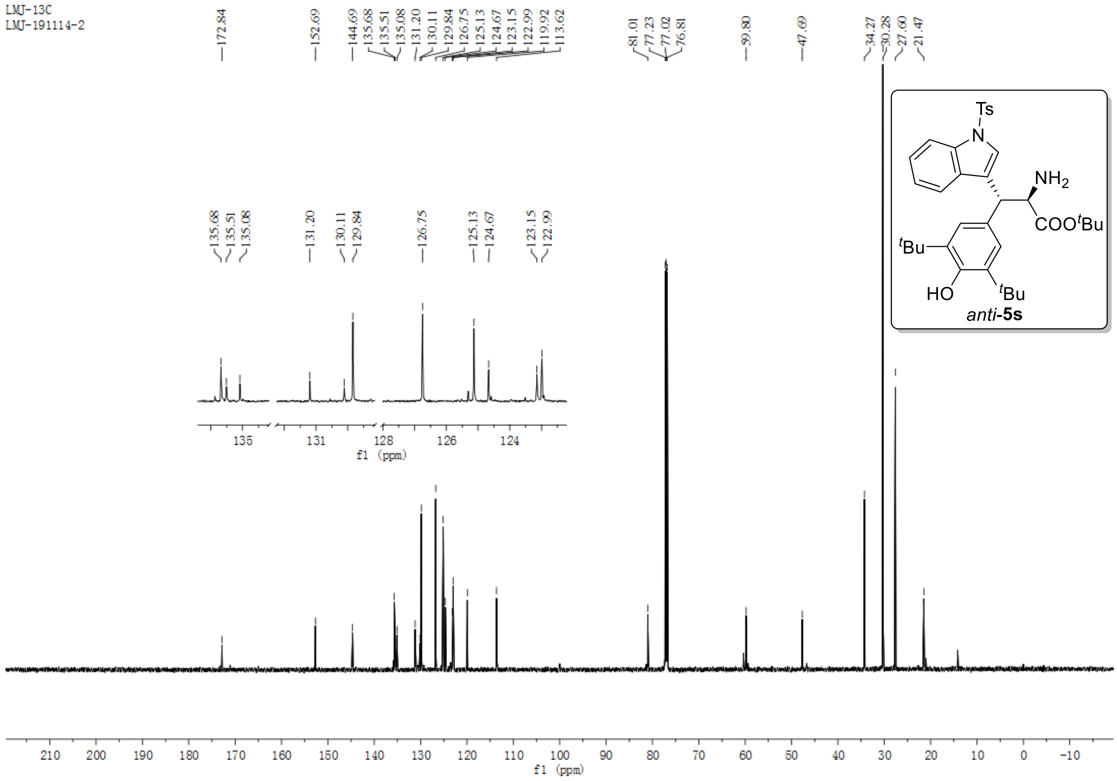
LMJ-13C
LMJ-191114-2
172.84
152.69
144.69 135.68 135.51 135.08 131.20 130.11 129.84 126.75 125.13 124.67 123.15 122.99 119.92 113.62
81.01 77.23 77.02 76.81
59.80
47.69
34.27 30.28 27.60 21.47
f1 (ppm)
Ts
N
NH2
COOtBu
tBu
HO
tBu
anti-5s

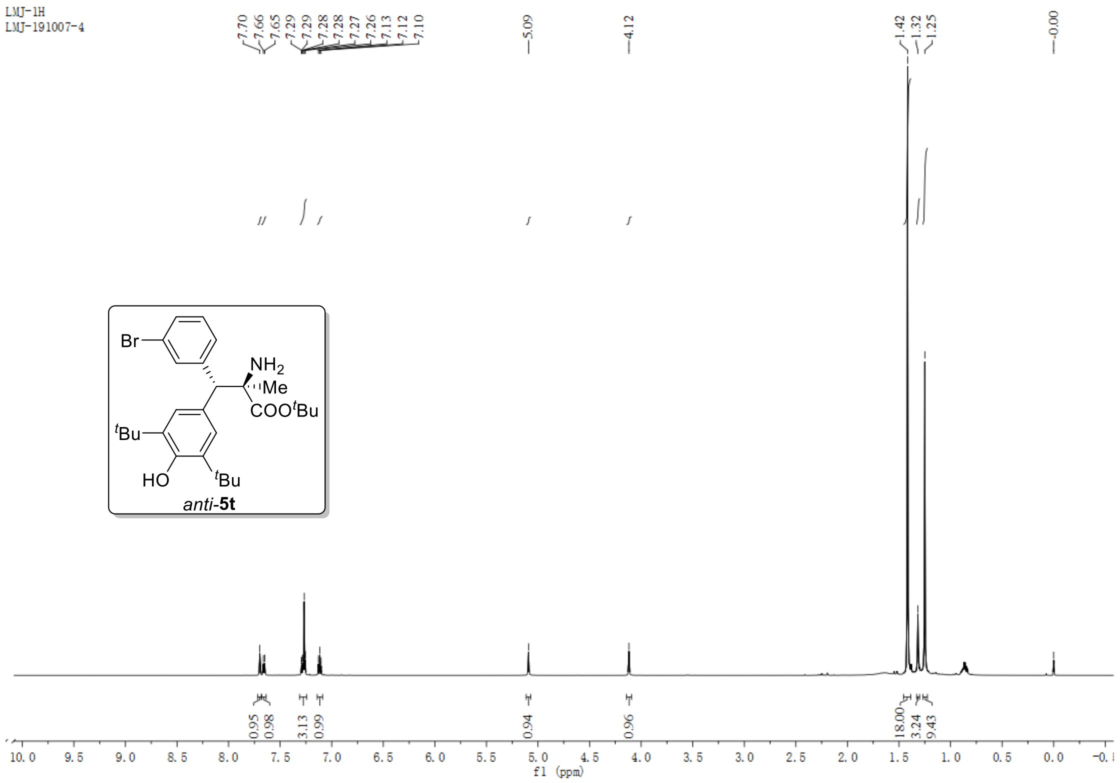
LMJ-1H
LMJ-191007-4
7.70 7.66 7.65 7.29 7.29 7.28 7.28 7.27 7.26 7.13 7.12 7.10
5.09
4.12
1.42 1.32 1.25
0.00
Br
NH2
Me
COOtBu
tBu
HO
tBu
anti-5t
0.95 0.98 3.13 0.99 0.94 0.96 18.00 3.24 9.43
f1 (ppm)

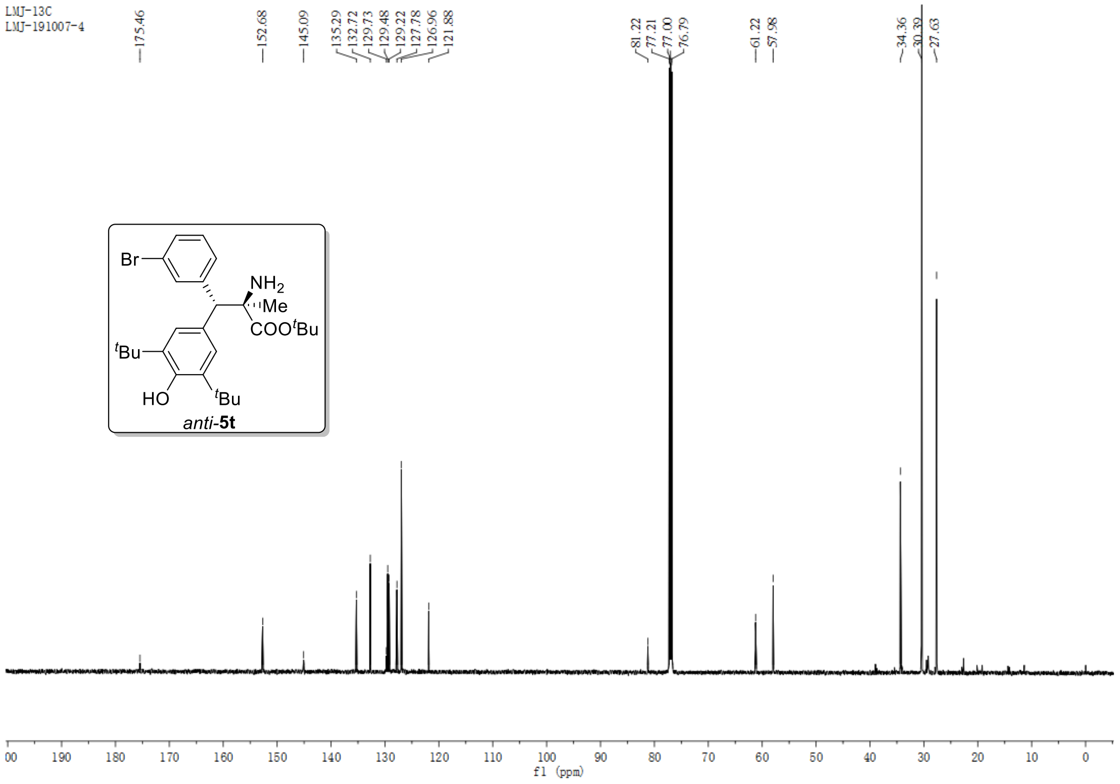
LMJ-13C
LMJ-191007-4
175.46
152.68
145.09
135.29 132.72 129.73 129.48 129.22 127.78 126.96 121.88
81.22 77.21 77.00 76.79
61.22 57.98
34.36 30.39 27.63
Br
NH2
Me
COOtBu
tBu
HO
tBu
anti-5t
f1 (ppm)

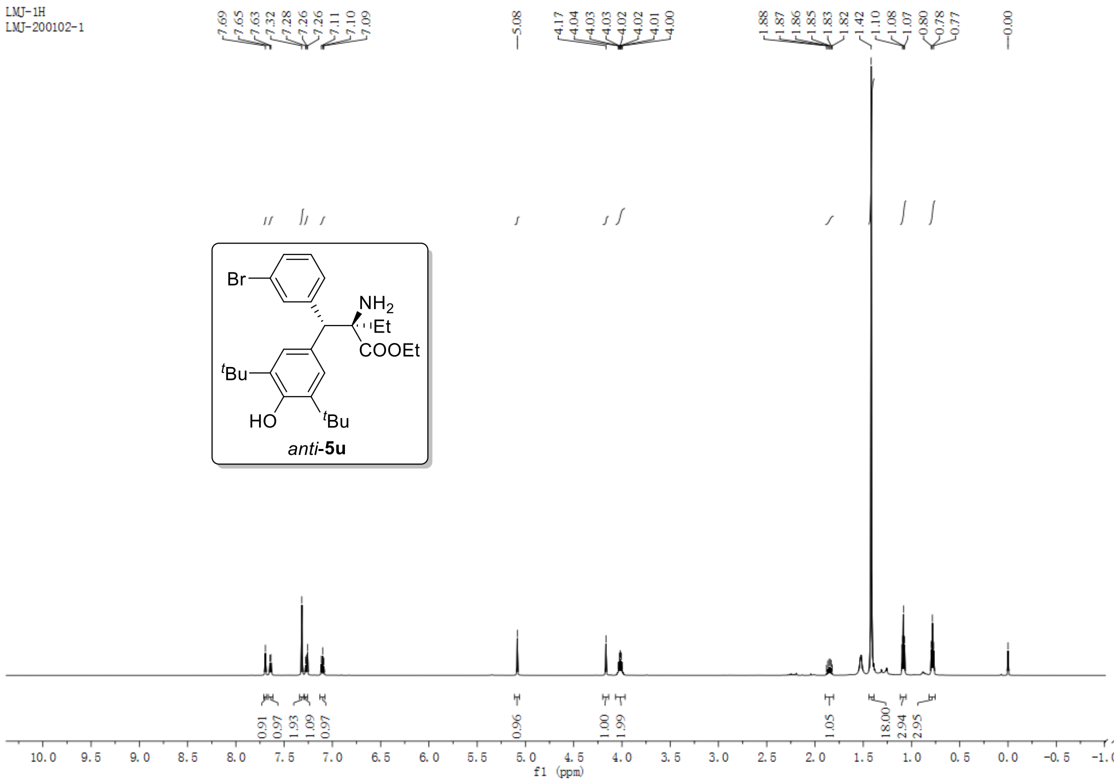
LMJ-1H
LMJ-200102-1
7.69
7.65
7.63
7.32
7.28
7.26
7.26
7.11
7.10
7.09
5.08
4.17
4.04
4.03
4.03
4.02
4.02
4.01
4.00
1.88
1.87
1.86
1.85
1.83
1.82
1.42
1.10
1.08
1.07
0.80
0.78
0.77
0.00
Br
NH2
Et
COOEt
tBu
HO
tBu
anti-5u
0.91
0.97
1.93
1.09
0.97
0.96
1.00
1.99
1.05
18.00
2.94
2.95
f1 (ppm)

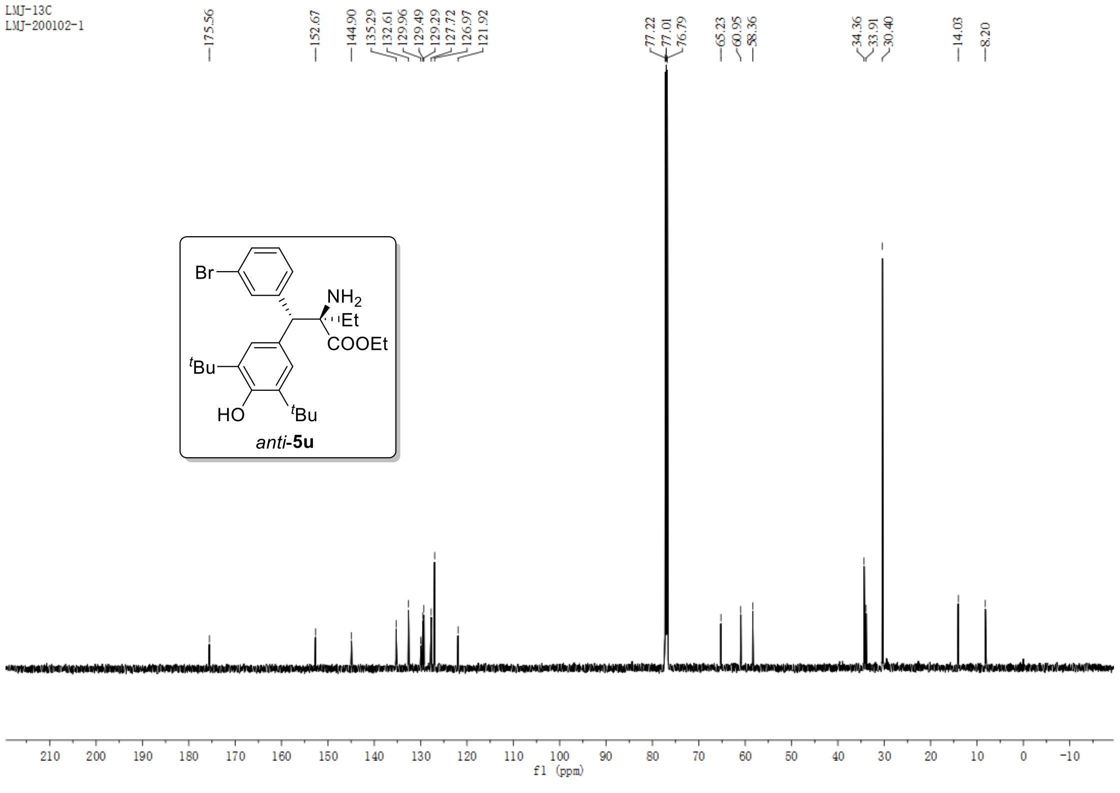
LMJ-13C
LMJ-200102-1
175.56
152.67
144.90
135.29
132.61
129.96
129.49
129.29
127.72
126.97
121.92
77.22
77.01
76.79
65.23
60.95
58.36
34.36
33.91
30.40
14.03
8.20
Br
NH2
Et
COOEt
tBu
HO
tBu
anti-5u
f1 (ppm)

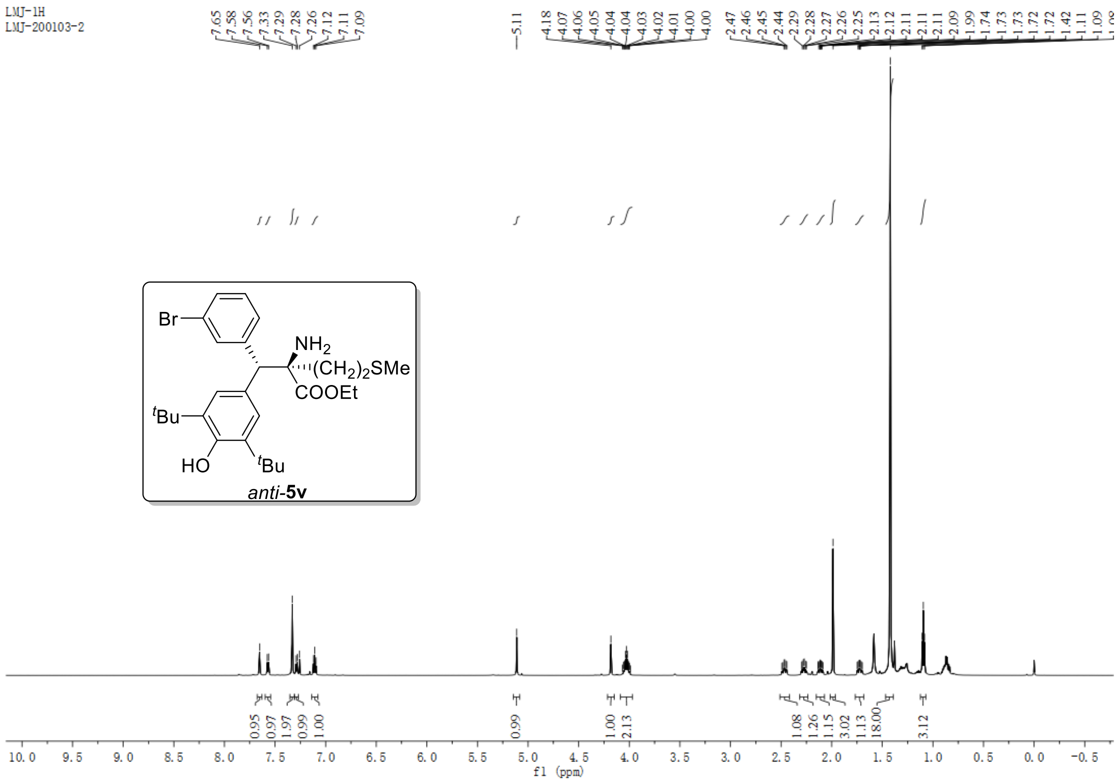
LMJ-1H
LMJ-200103-2
Br
NH2
(CH2)2SMe
COOEt
tBu
HO
tBu
anti-5v
f1 (ppm)

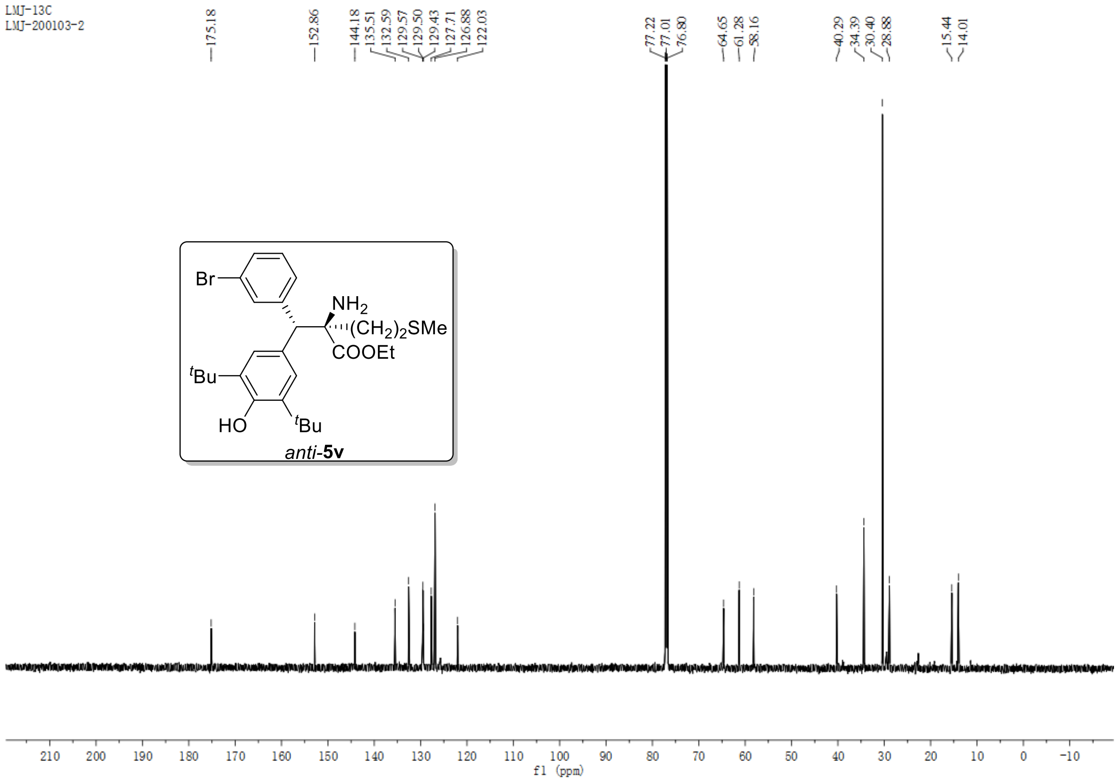
LMJ-13C
LMJ-200103-2
Br
NH2
(CH2)2SMe
COOEt
tBu
HO
tBu
anti-5v
f1 (ppm)

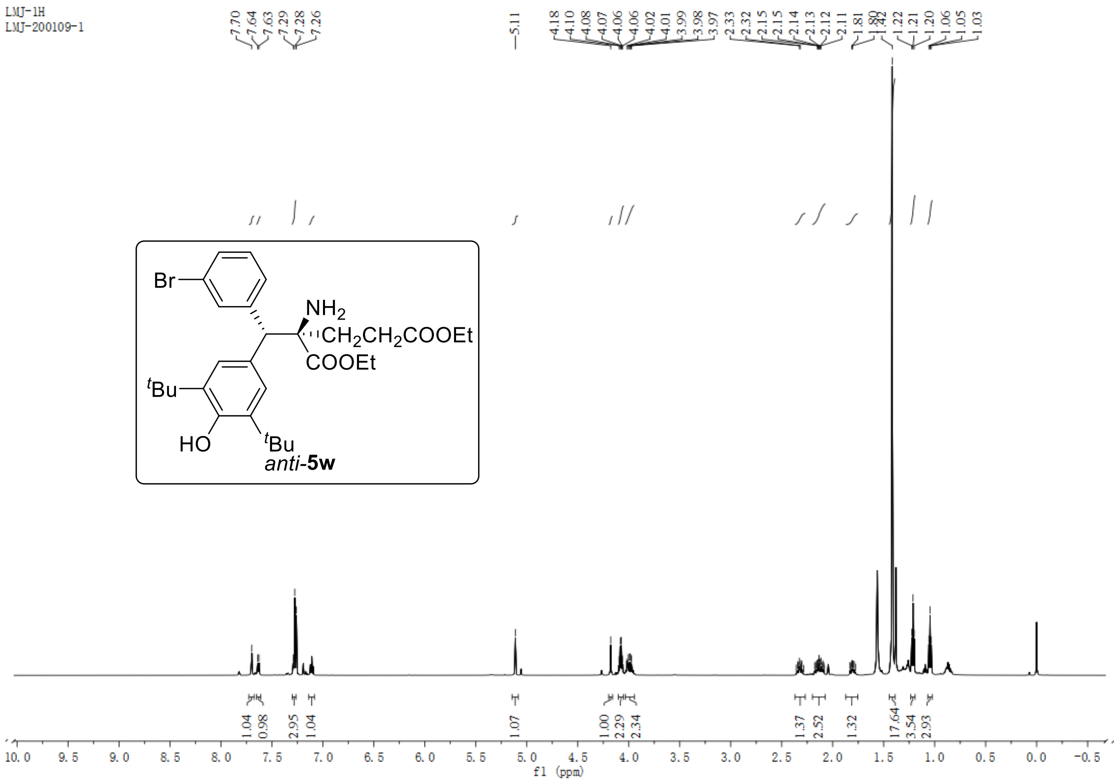
LMJ-1H
LMJ-200109-1
anti-5w
f1 (ppm)

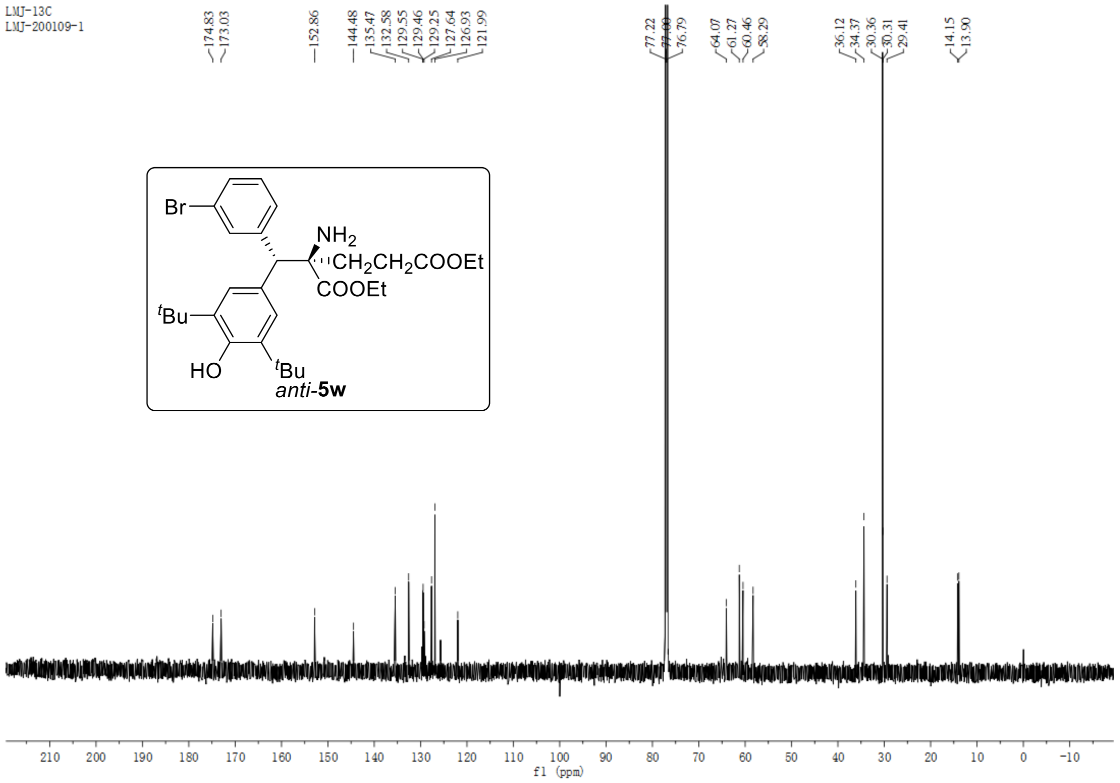
LMJ-13C
LMJ-200109-1
anti-5w
f1 (ppm)

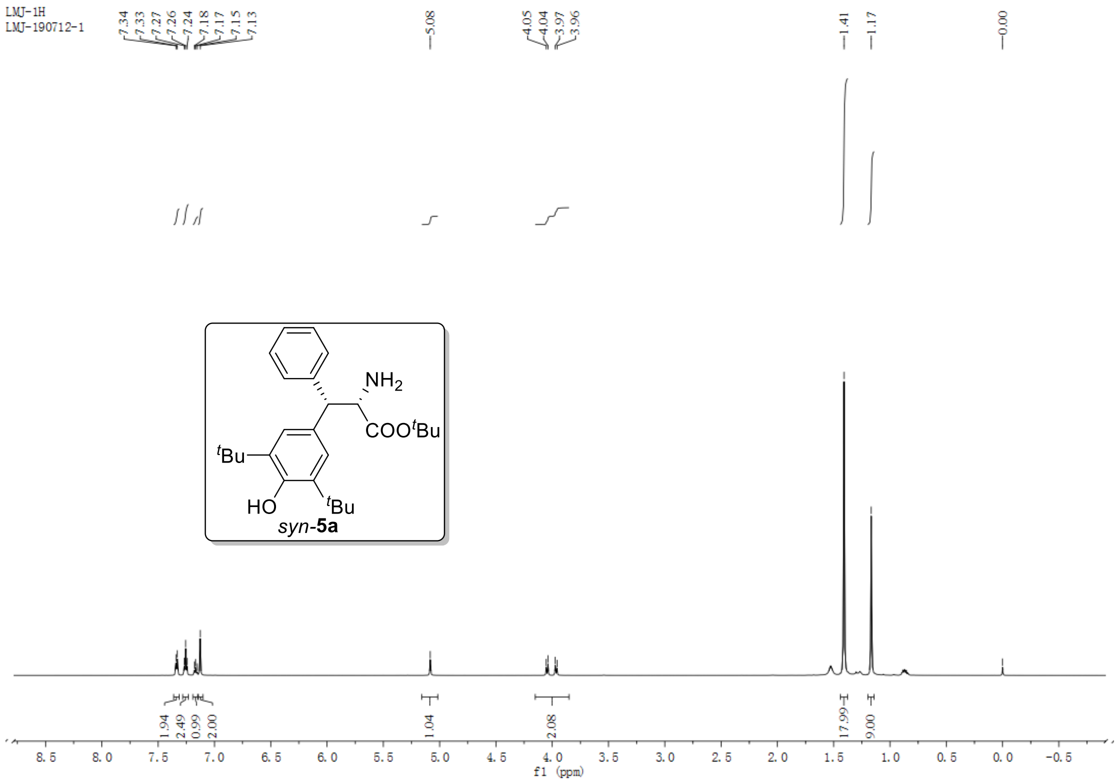
LMJ-1H
LMJ-190712-1
7.34
7.33
7.27
7.26
7.24
7.18
7.17
7.15
7.13
5.08
4.05
4.04
3.97
3.96
1.41
1.17
0.00
NH2
COOtBu
tBu
HO
tBu
syn-5a
1.94
2.49
0.99
2.00
1.04
2.08
17.99
9.00
f1 (ppm)

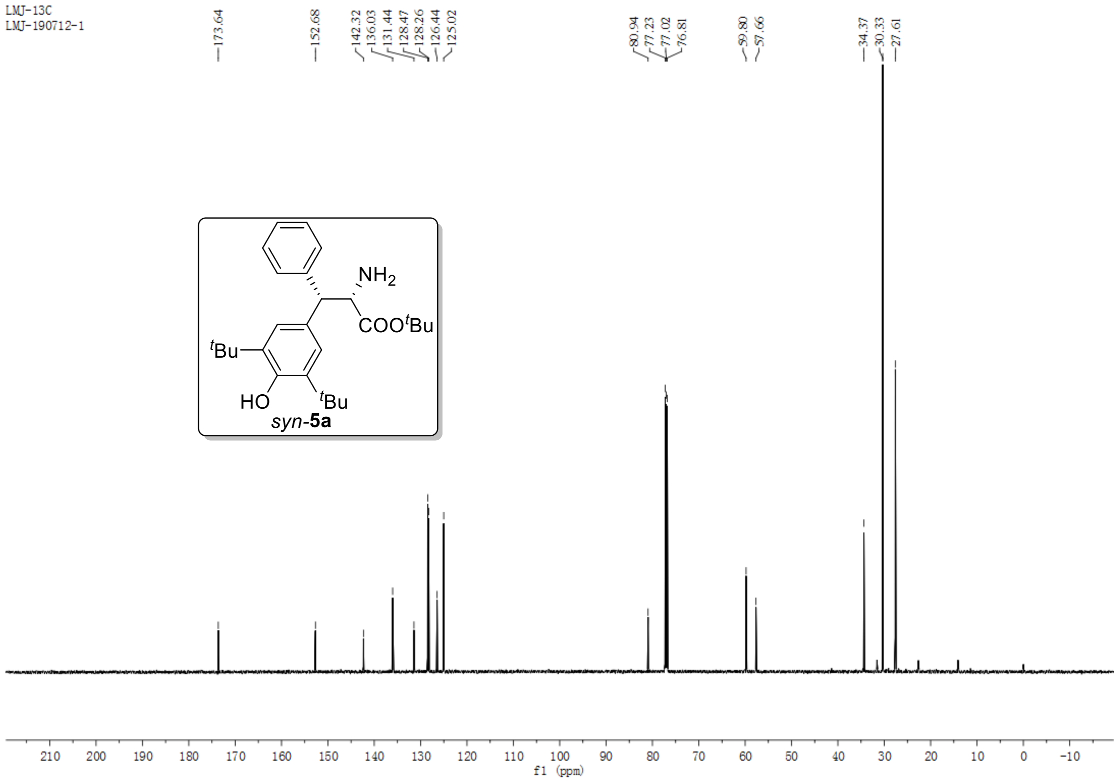
LMJ-13C
LMJ-190712-1
173.64
152.68
142.32
136.03
131.44
128.47
128.26
126.44
125.02
80.94
77.23
77.02
76.81
59.80
57.66
34.37
30.33
27.61
NH2
COOtBu
tBu
HO
tBu
syn-5a
f1 (ppm)

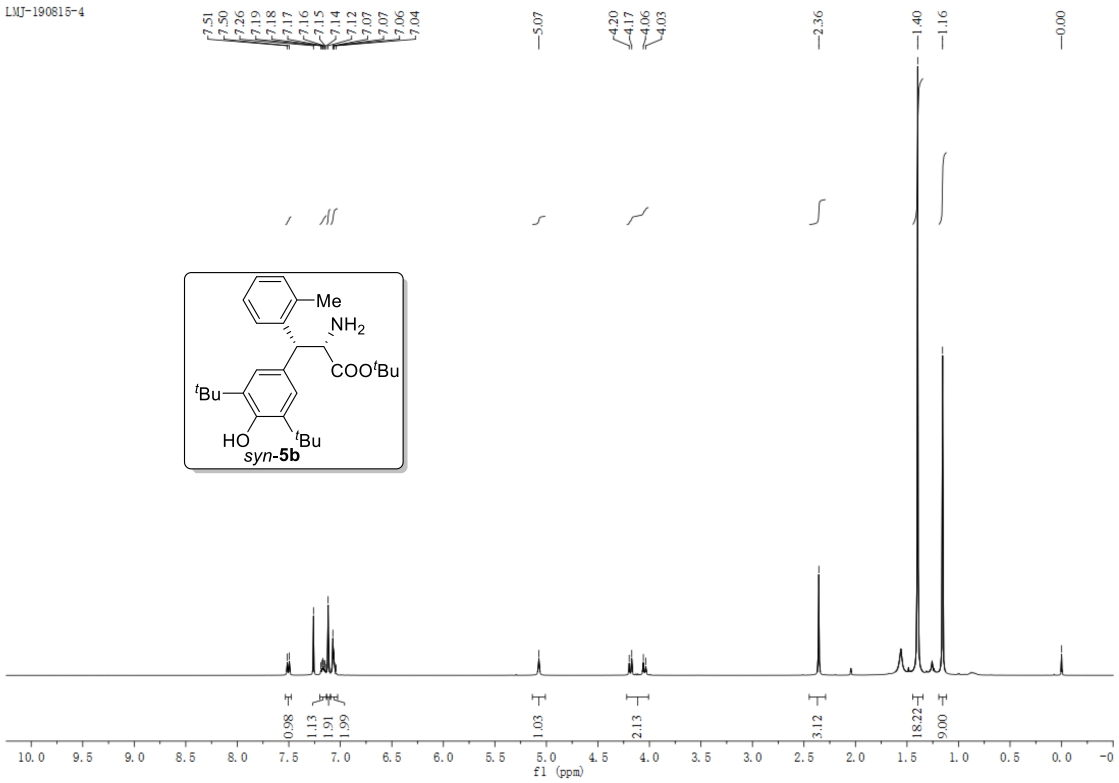
LMJ-190815-4
7.51
7.50
7.26
7.19
7.18
7.17
7.16
7.15
7.14
7.12
7.07
7.07
7.06
7.04
5.07
4.20
4.17
4.06
4.03
2.36
1.40
1.16
0.00
Me
NH2
COOtBu
tBu
HO
tBu
syn-5b
0.98
1.13
1.91
1.99
1.03
2.13
3.12
18.22
9.00
f1 (ppm)

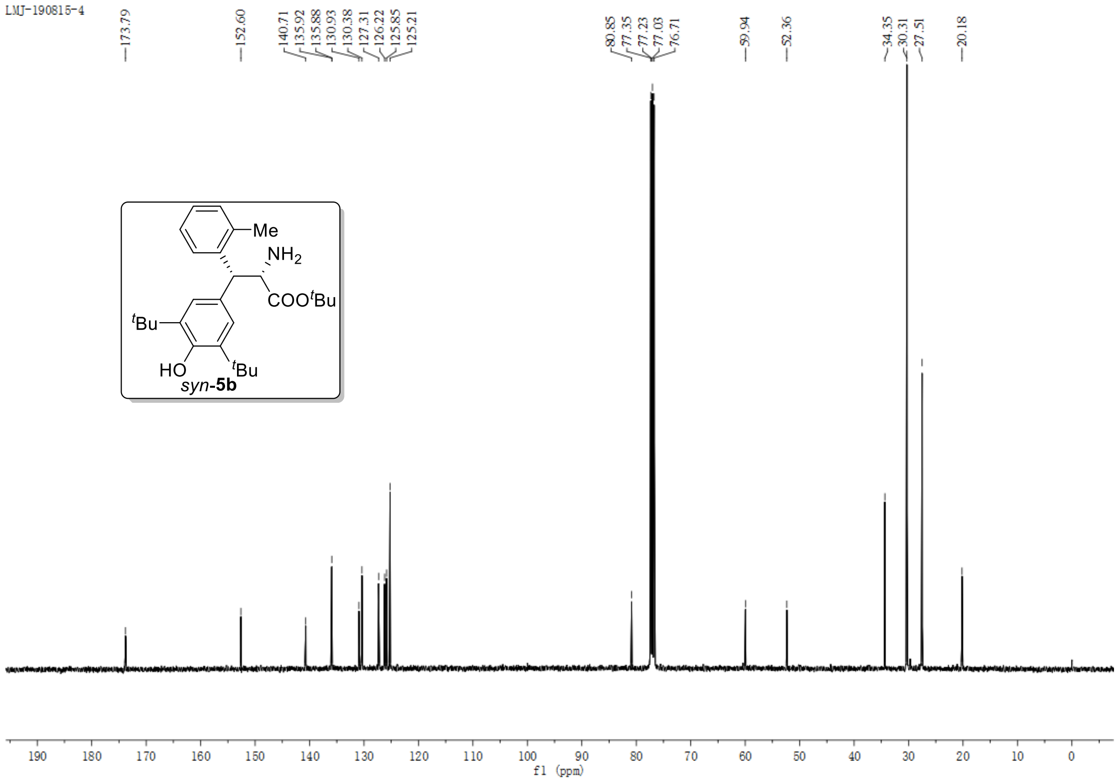
LMJ-190815-4
173.79
152.60
140.71
135.92
135.88
130.93
130.38
127.31
126.22
125.85
125.21
80.85
77.35
77.23
77.03
76.71
59.94
52.36
34.35
30.31
27.51
20.18
Me
NH2
COOtBu
tBu
HO
tBu
syn-5b
f1 (ppm)

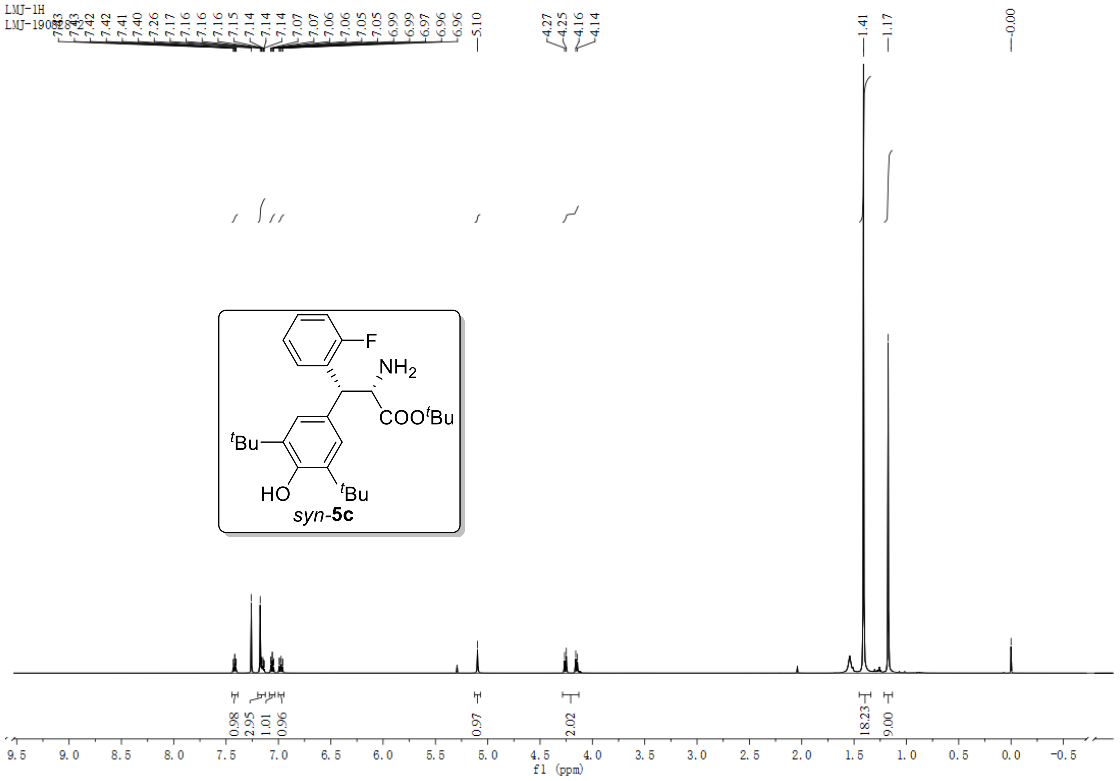
LMJ-1H
7.43
7.43
7.42
7.42
7.41
7.40
7.26
7.17
7.16
7.16
7.16
7.15
7.14
7.14
7.14
7.14
7.07
7.07
7.06
7.06
7.06
7.05
7.05
6.99
6.99
6.97
6.96
6.96
5.10
4.27
4.25
4.16
4.14
1.41
1.17
0.00
F
NH2
COOtBu
tBu
HO
tBu
syn-5c
0.98
2.95
1.01
0.96
0.97
2.02
18.23
9.00
f1 (ppm)

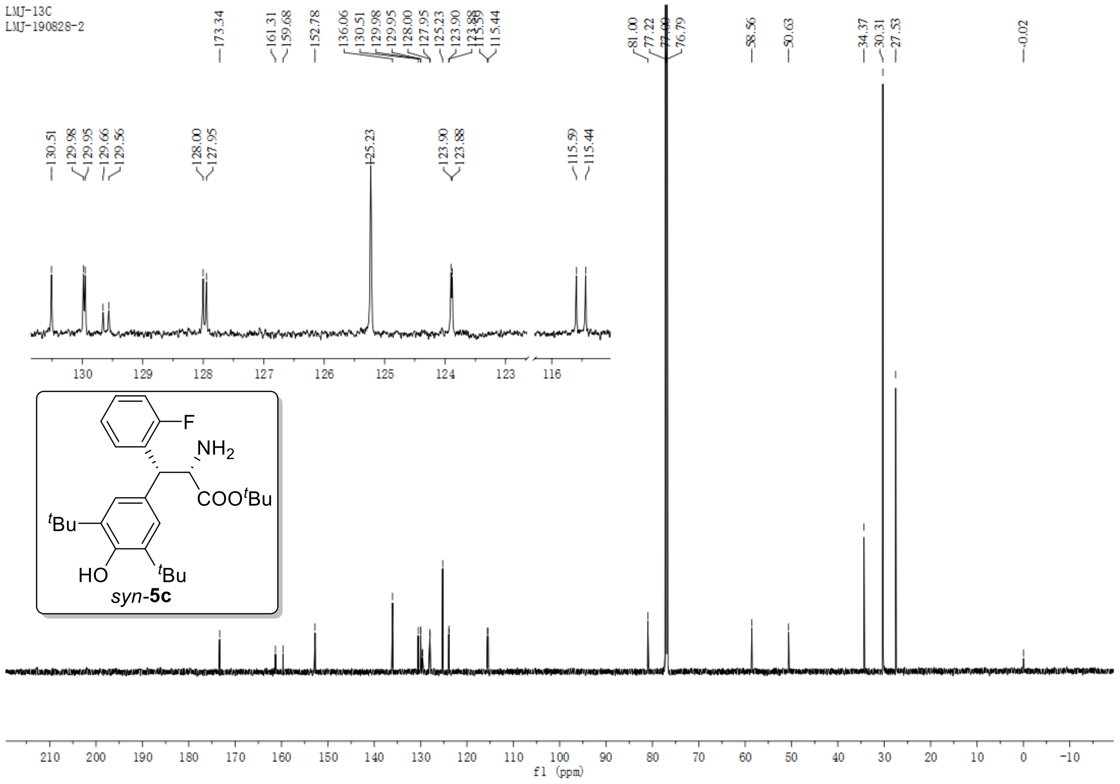
LMJ-13C
LMJ-190828-2
173.34
161.31
159.68
152.78
136.06
130.51
129.98
129.95
128.00
127.95
125.23
123.90
115.44
81.00
77.22
76.79
58.56
50.63
34.37
30.31
27.53
-0.02
130.51
129.98
129.95
129.66
129.56
128.00
127.95
125.23
123.90
123.88
115.59
115.44
F
NH2
COOtBu
tBu
HO
tBu
syn-5c
f1 (ppm)

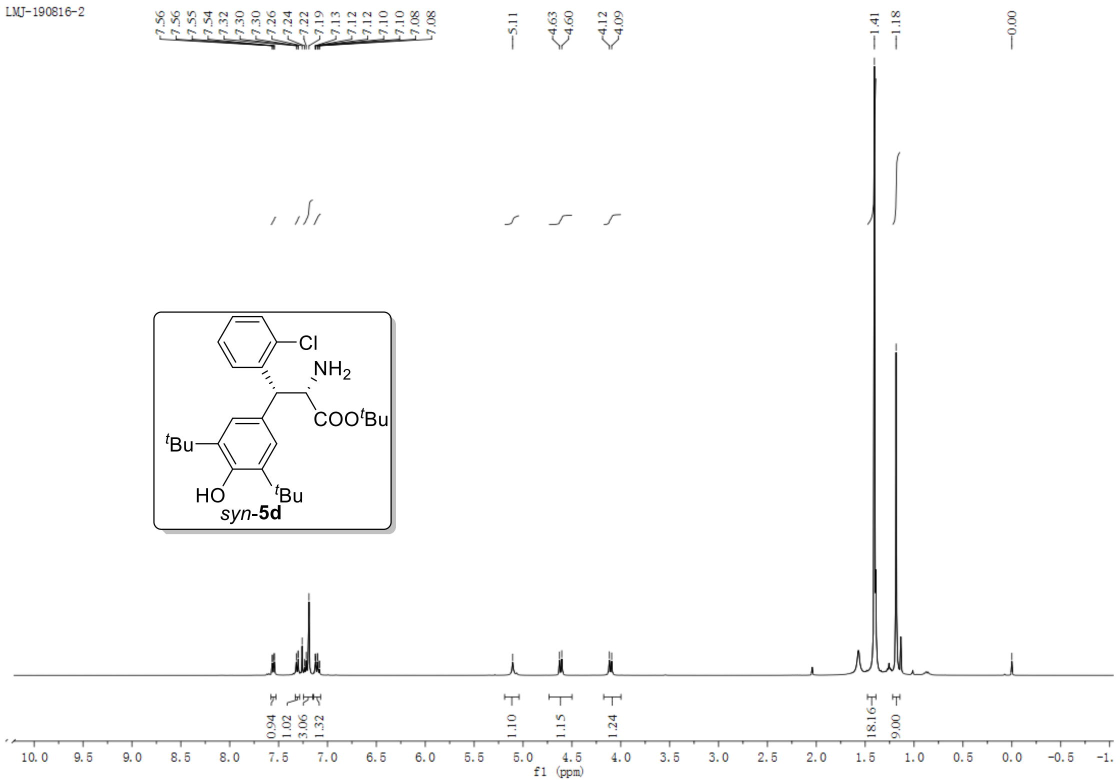
LMJ-190816-2
7.56
7.56
7.55
7.54
7.32
7.30
7.30
7.26
7.24
7.22
7.19
7.13
7.12
7.12
7.10
7.08
7.08
5.11
4.63
4.60
4.12
4.09
1.41
1.18
0.00
Cl
NH2
COOtBu
tBu
HO
tBu
syn-5d
0.94
1.02
3.06
1.32
1.10
1.15
1.24
18.16
9.00
f1 (ppm)

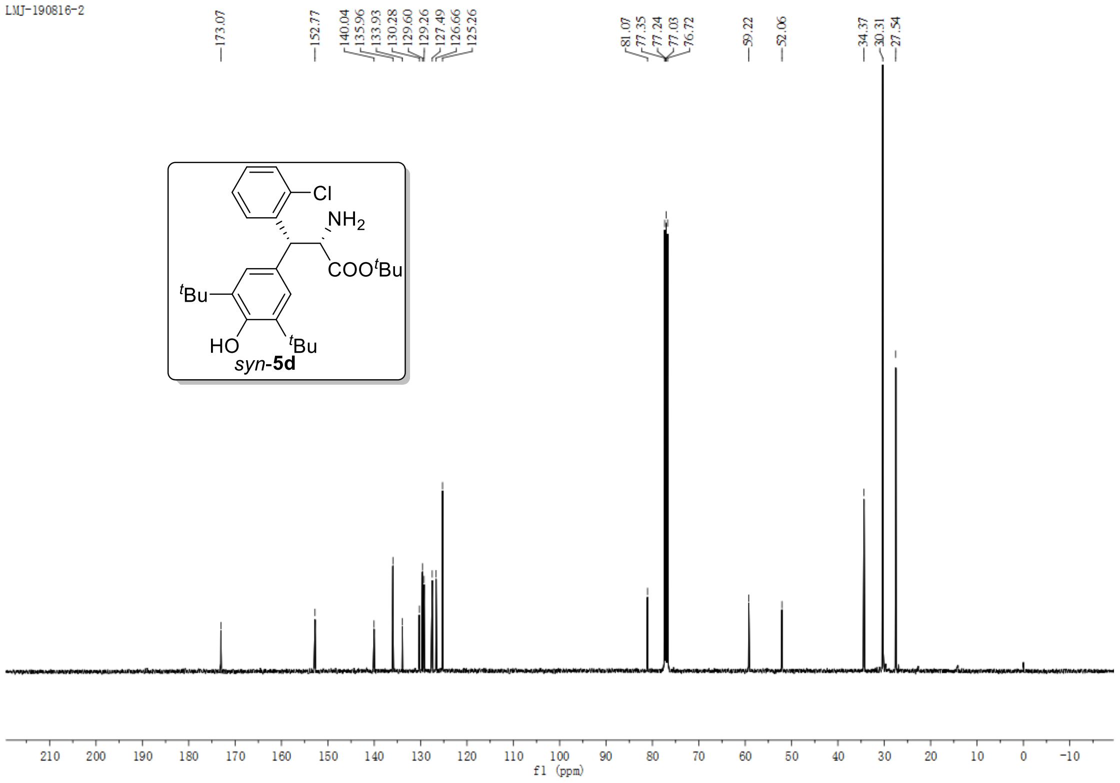
LMJ-190816-2
173.07
152.77
140.04
135.96
133.93
130.28
129.60
129.26
127.49
126.66
125.26
81.07
77.35
77.24
77.03
76.72
59.22
52.06
34.37
30.31
27.54
Cl
NH2
COOtBu
tBu
HO
tBu
syn-5d
f1 (ppm)

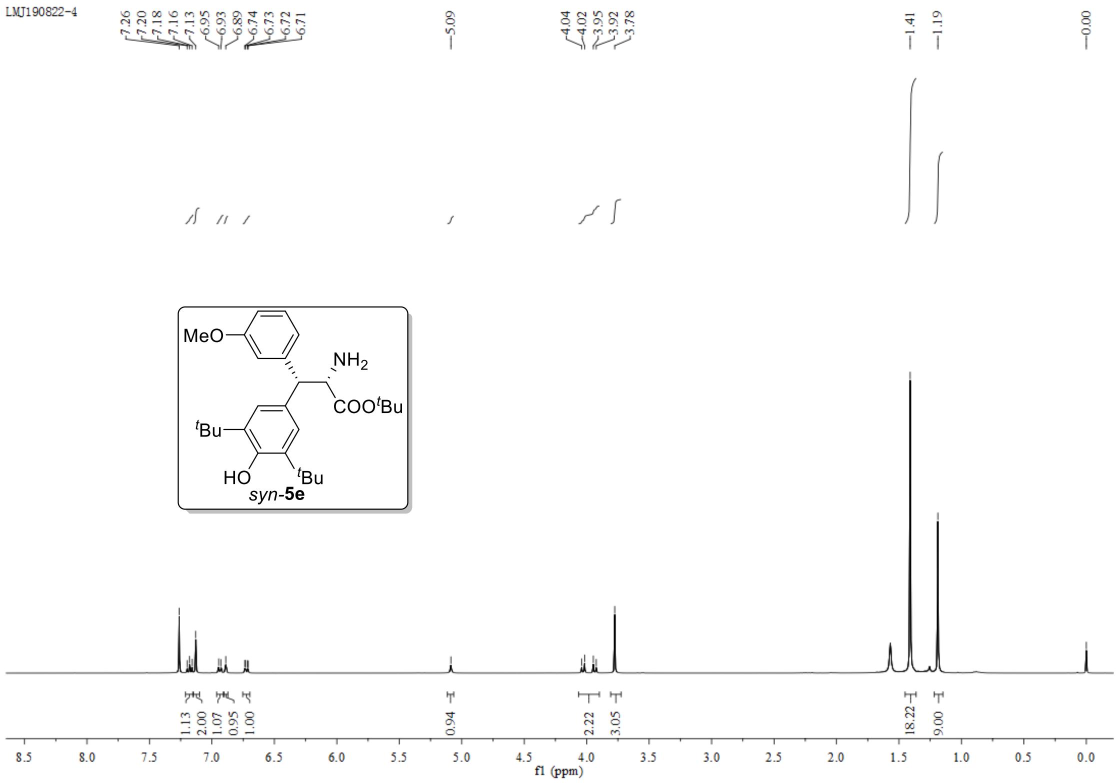
LMJ190822-4
7.26
7.20
7.18
7.16
7.13
6.95
6.93
6.89
6.74
6.73
6.72
6.71
5.09
4.04
4.02
3.95
3.92
3.78
1.41
1.19
0.00
MeO
NH2
COOtBu
tBu
HO
tBu
syn-5e
1.13
2.00
1.07
0.95
1.00
0.94
2.22
3.05
18.22
9.00
f1 (ppm)

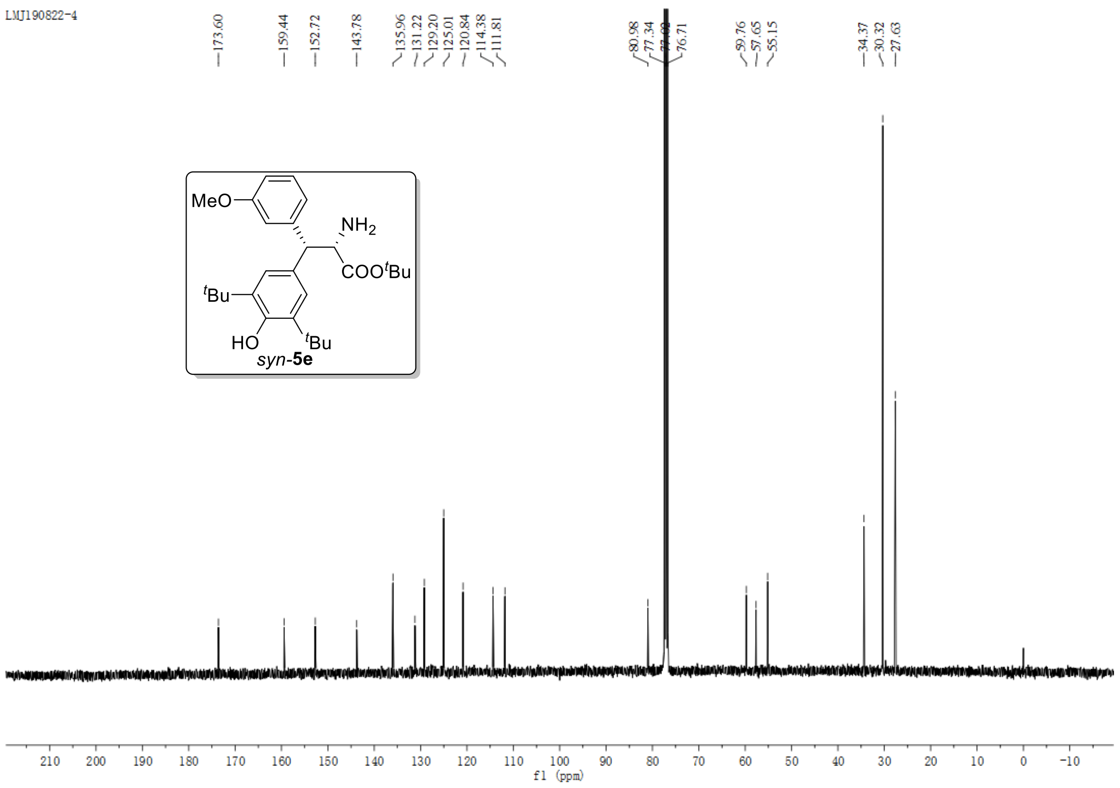
LMJ190822-4
173.60
159.44
152.72
143.78
135.96
131.22
129.20
125.01
120.84
114.38
111.81
80.98
77.34
76.71
59.76
57.65
55.15
34.37
30.32
27.63
MeO
NH2
COOtBu
tBu
HO
tBu
syn-5e
f1 (ppm)

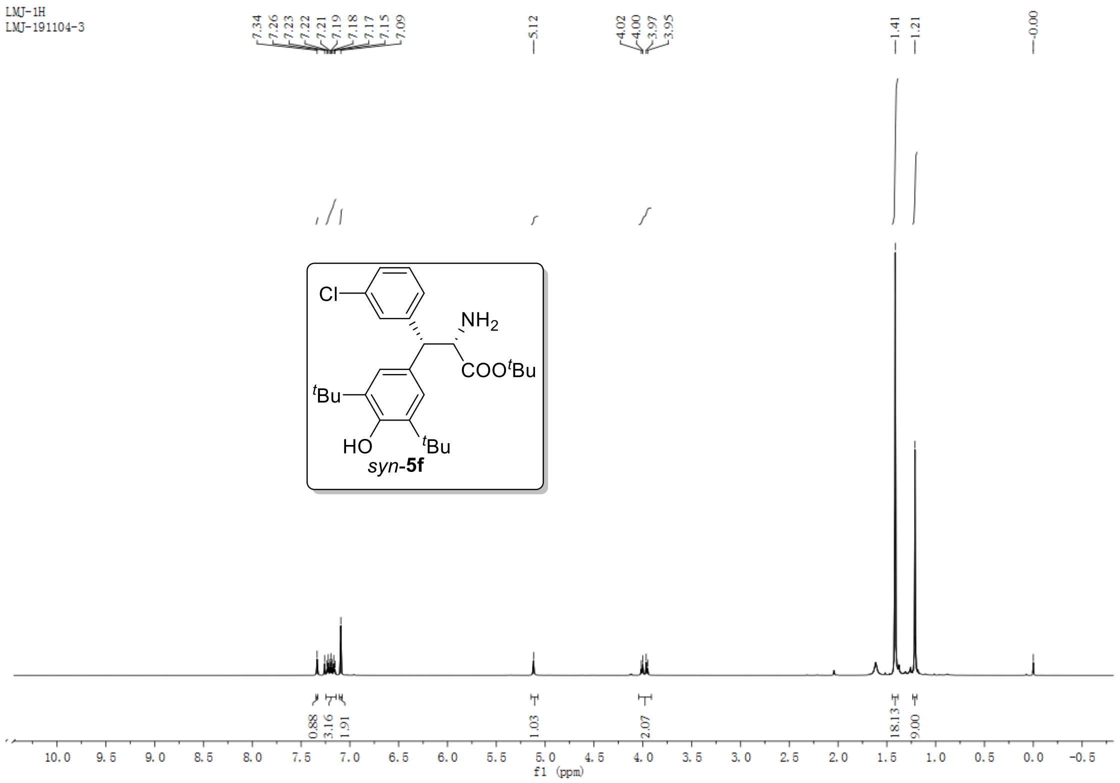
LMJ-1H
LMJ-191104-3
7.34
7.26
7.23
7.22
7.21
7.19
7.18
7.17
7.15
7.09
5.12
4.02
4.00
3.97
3.95
1.41
1.21
-0.00
Cl
NH2
COOtBu
tBu
HO
tBu
syn-5f
0.88
3.16
1.91
1.03
2.07
18.13
9.00
10.0 9.5 9.0 8.5 8.0 7.5 7.0 6.5 6.0 5.5 5.0 4.5 4.0 3.5 3.0 2.5 2.0 1.5 1.0 0.5 0.0 -0.5
f1 (ppm)

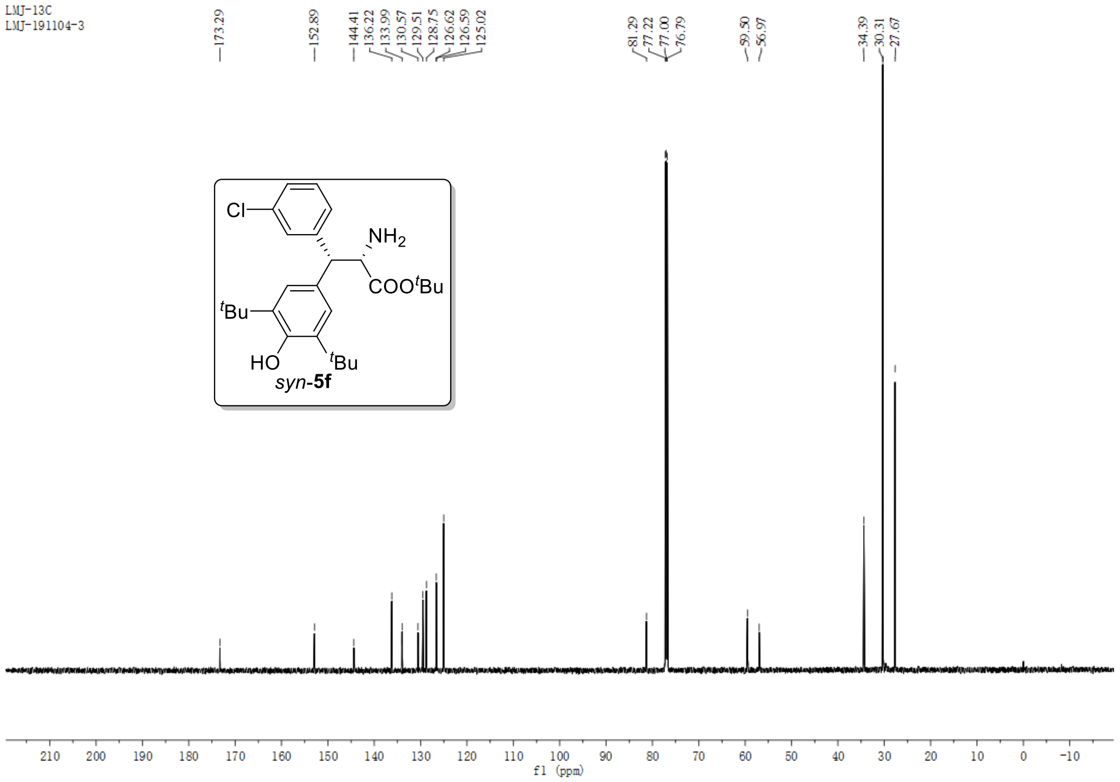
LMJ-13C
LMJ-191104-3
173.29
152.89
144.41
136.22
133.99
130.57
129.51
128.75
126.62
126.59
125.02
81.29
77.22
77.00
76.79
59.50
56.97
34.39
30.31
27.67
Cl
NH2
COOtBu
tBu
HO
tBu
syn-5f
210 200 190 180 170 160 150 140 130 120 110 100 90 80 70 60 50 40 30 20 10 0 -10
f1 (ppm)

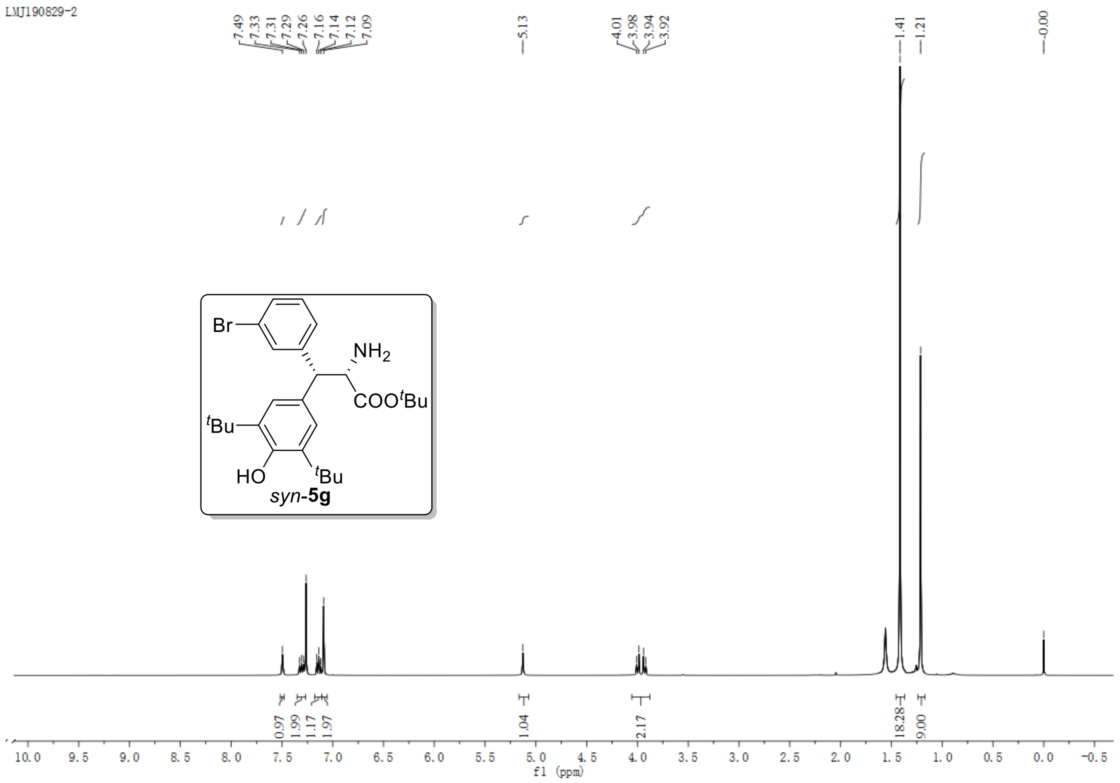
LMJ190829-2
7.49
7.33
7.31
7.29
7.26
7.16
7.14
7.12
7.09
5.13
4.01
3.98
3.94
3.92
1.41
1.21
-0.00
Br
NH2
COOtBu
tBu
HO
tBu
syn-5g
0.97
1.99
1.17
1.97
1.04
2.17
18.28
9.00
f1 (ppm)

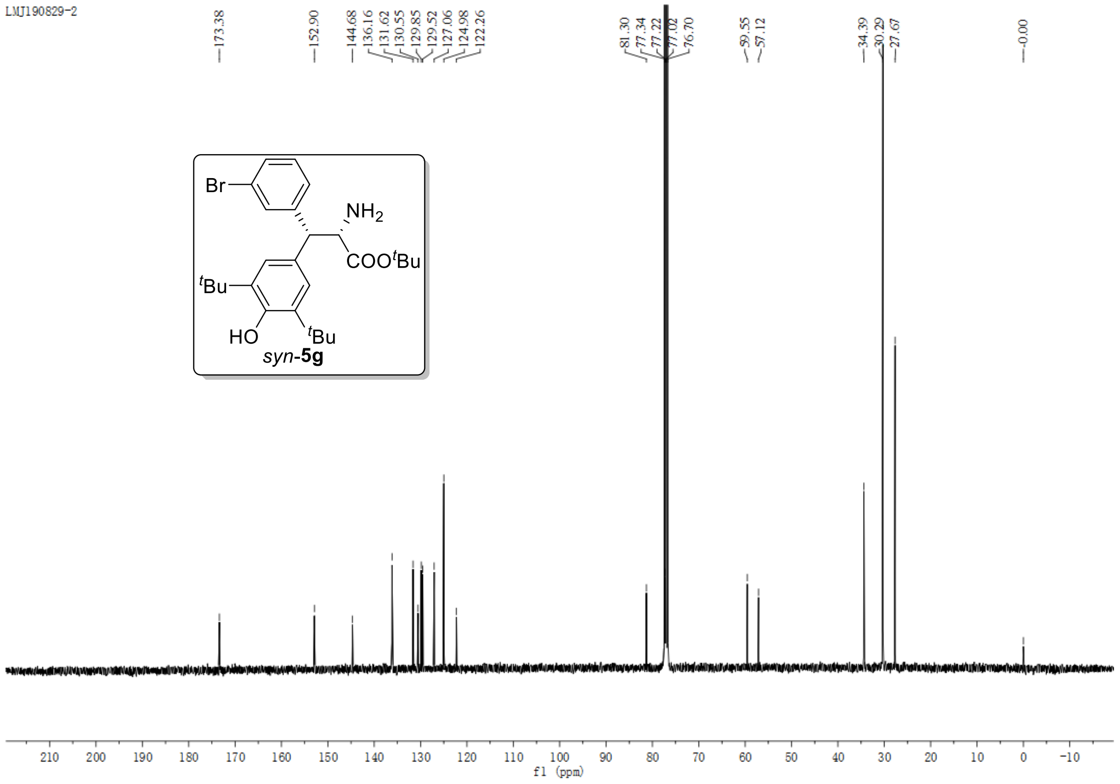
LMJ190829-2
173.38
152.90
144.68
136.16
131.62
130.55
129.85
129.52
127.06
124.98
122.26
81.30
77.34
77.22
77.02
76.70
59.55
57.12
34.39
30.29
27.67
-0.00
Br
NH2
COOtBu
tBu
HO
tBu
syn-5g
f1 (ppm)

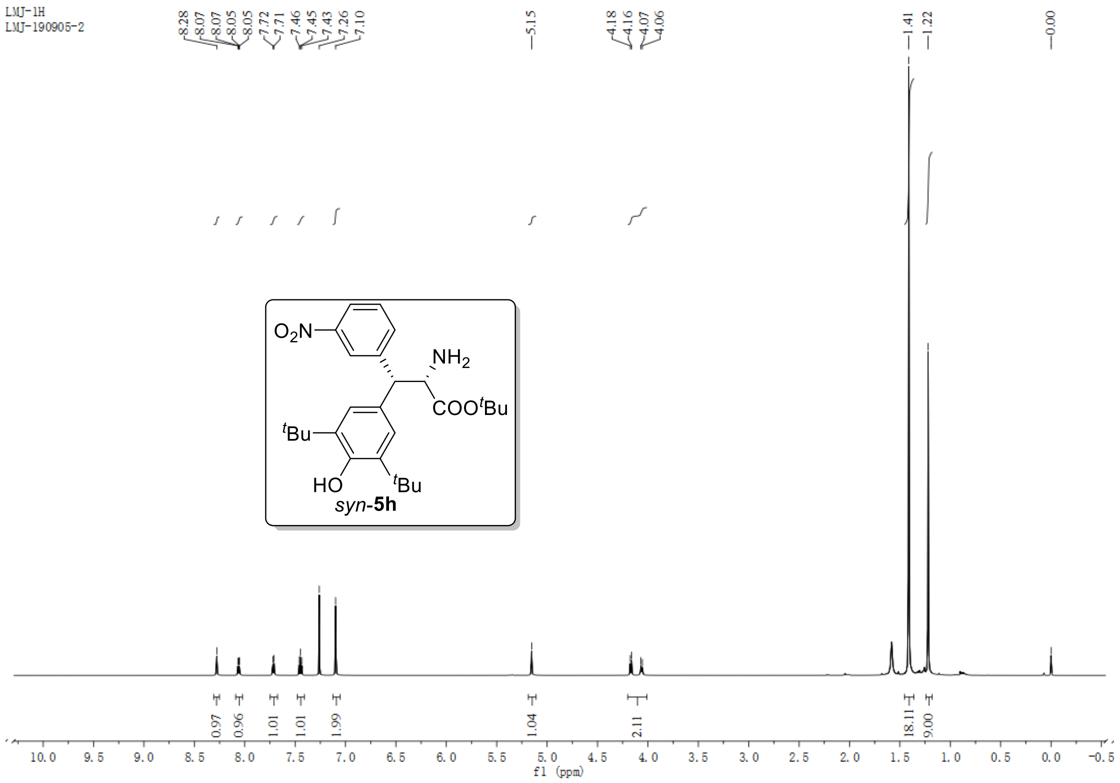
LMJ-1H
LMJ-190905-2
8.28 8.07 8.07 8.05 8.05 7.72 7.71 7.46 7.45 7.43 7.26 7.10
5.15
4.18 4.16 4.07 4.06
1.41 1.22
0.00
O2N
NH2
COOtBu
tBu
HO
tBu
syn-5h
0.97 0.96 1.01 1.01 1.99 1.04 2.11 18.11 9.00
f1 (ppm)

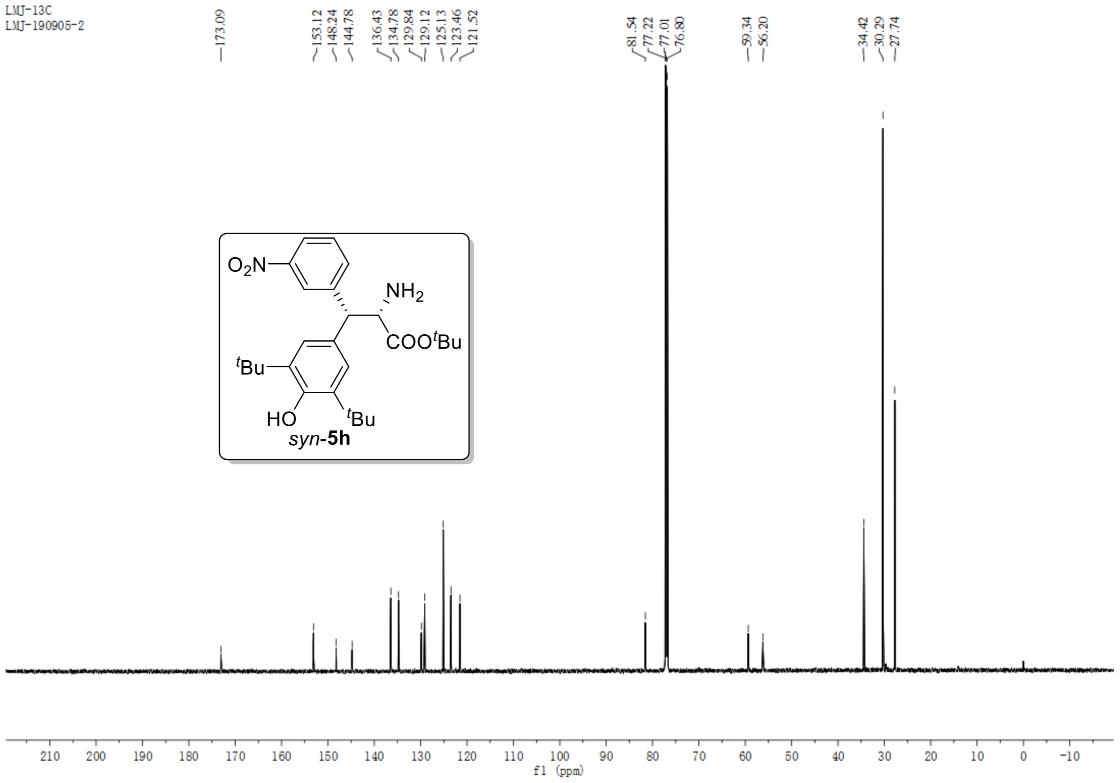
LMJ-13C
LMJ-190905-2
173.09
153.12 148.24 144.78
136.43 134.78 129.84 129.12 125.13 123.46 121.52
81.54 77.22 77.01 76.80
59.34 56.20
34.42 30.29 27.74
O2N
NH2
COOtBu
tBu
HO
tBu
syn-5h
f1 (ppm)

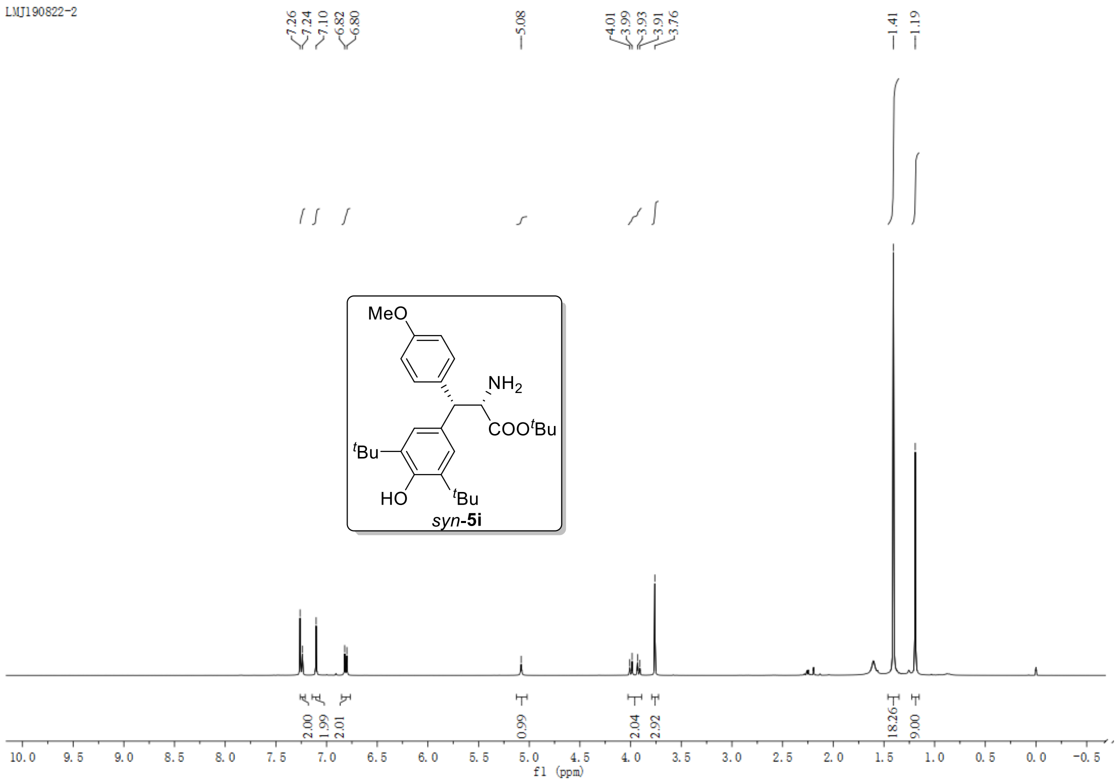
LMJ190822-2
7.26
7.24
7.10
6.82
6.80
5.08
4.01
3.99
3.93
3.91
3.76
1.41
1.19
MeO
NH2
COO^tBu
tBu
HO
tBu
syn-5i
2.00
1.99
2.01
0.99
2.04
2.92
18.26
9.00
f1 (ppm)

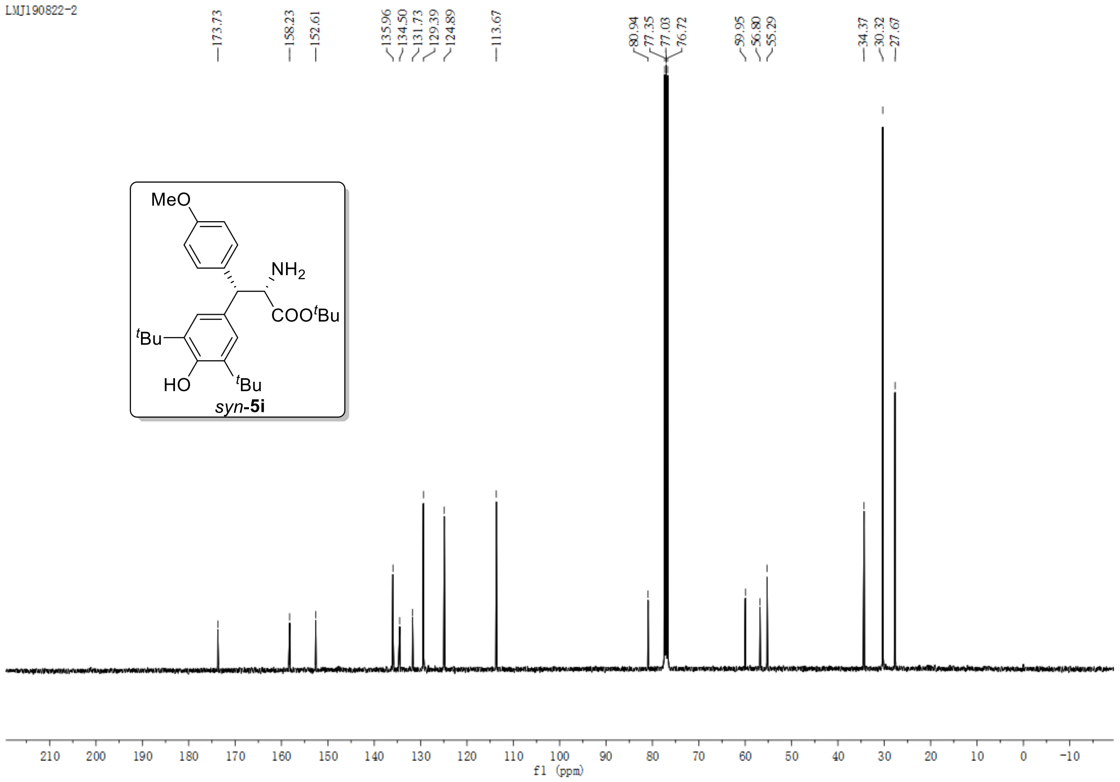
LMJ190822-2
173.73
158.23
152.61
135.96
134.50
131.73
129.39
124.89
113.67
80.94
77.35
77.03
76.72
59.95
56.80
55.29
34.37
30.32
27.67
MeO
NH2
COOtBu
tBu
HO
tBu
syn-5i
f1 (ppm)

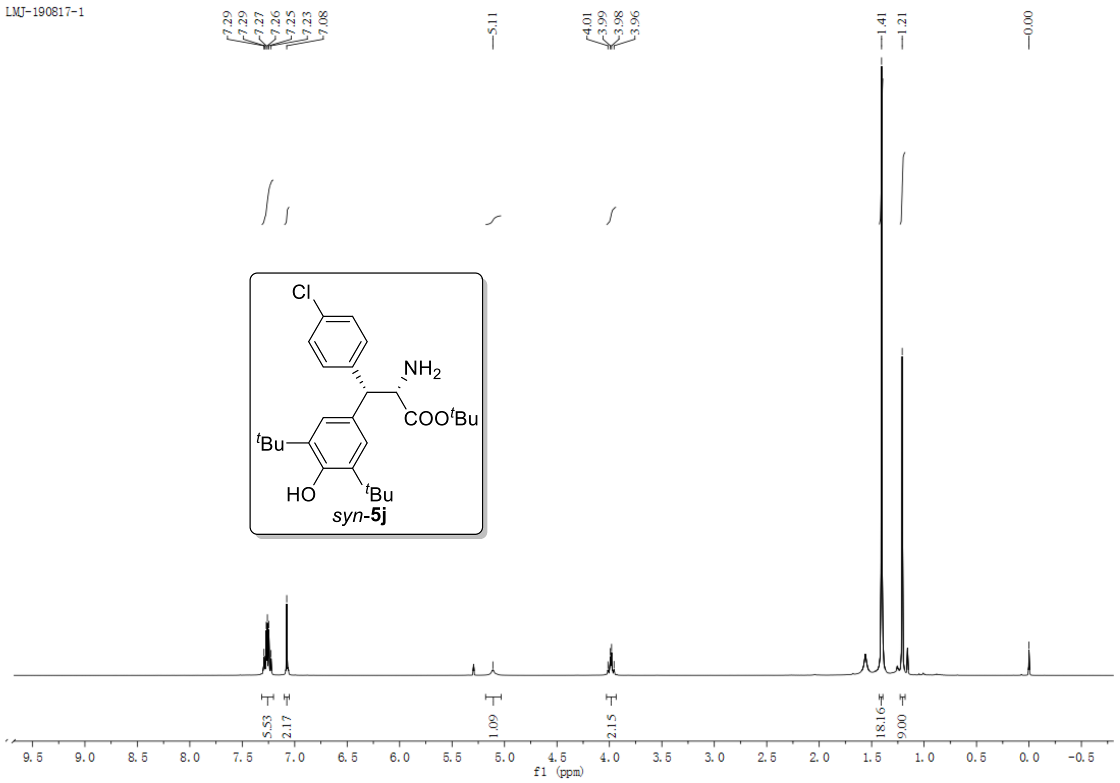
LMJ-190817-1
7.29
7.29
7.27
7.26
7.25
7.23
7.08
5.11
4.01
3.99
3.98
3.96
1.41
1.21
0.00
Cl
NH2
COOtBu
tBu
HO
tBu
syn-5j
5.53
2.17
1.09
2.15
18.16
9.00
f1 (ppm)

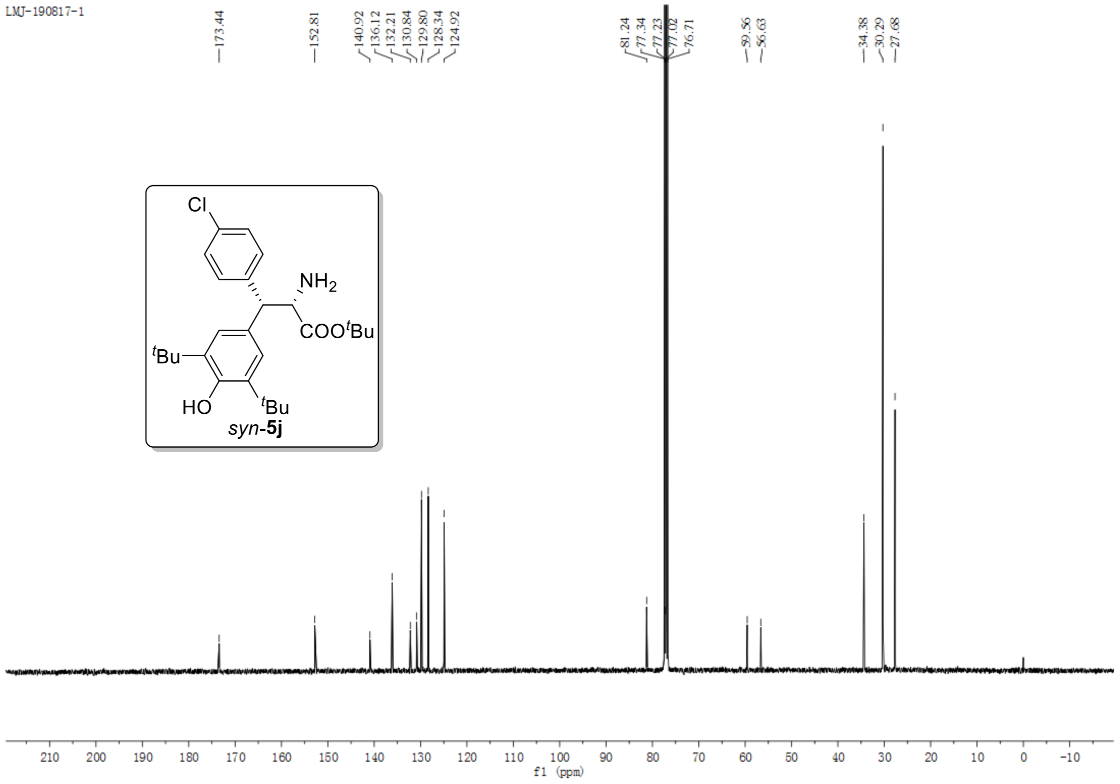
LMJ-190817-1
173.44
152.81
140.92
136.12
132.21
130.84
129.80
128.34
124.92
81.24
77.34
77.23
77.02
76.71
59.56
56.63
34.38
30.29
27.68
Cl
NH2
COOtBu
tBu
HO
tBu
syn-5j
f1 (ppm)

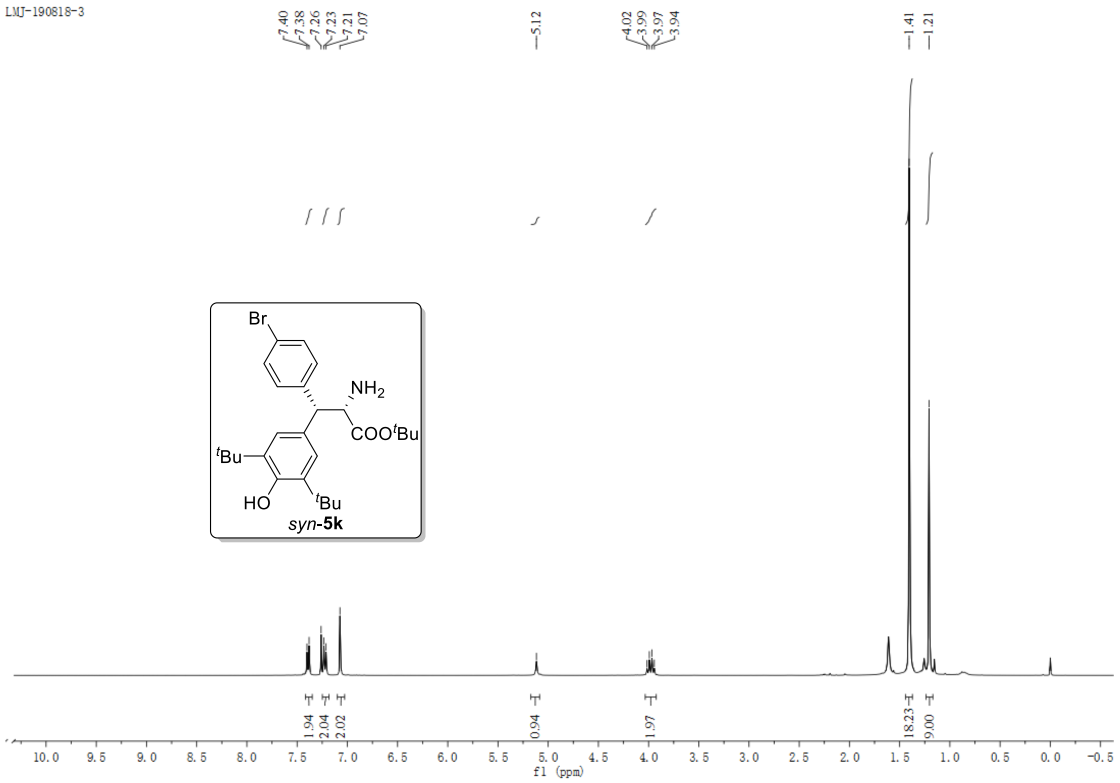
LMJ-190818-3
7.40
7.38
7.26
7.23
7.21
7.07
5.12
4.02
3.99
3.97
3.94
1.41
1.21
Br
NH2
COOtBu
tBu
HO
tBu
syn-5k
1.94
2.04
2.02
0.94
1.97
18.23
9.00
f1 (ppm)

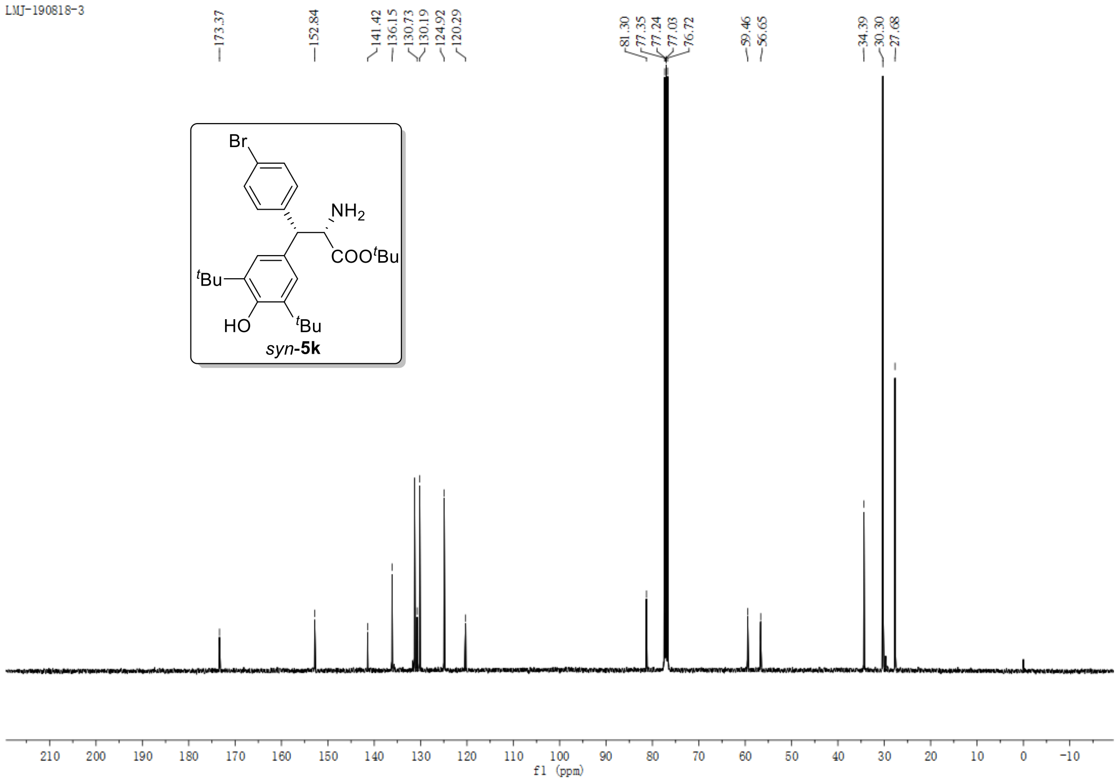
LMJ-190818-3
173.37
152.84
141.42
136.15
130.73
130.19
124.92
120.29
81.30
77.35
77.24
77.03
76.72
59.46
56.65
34.39
30.30
27.68
Br
NH2
COOtBu
tBu
HO
tBu
syn-5k
f1 (ppm)

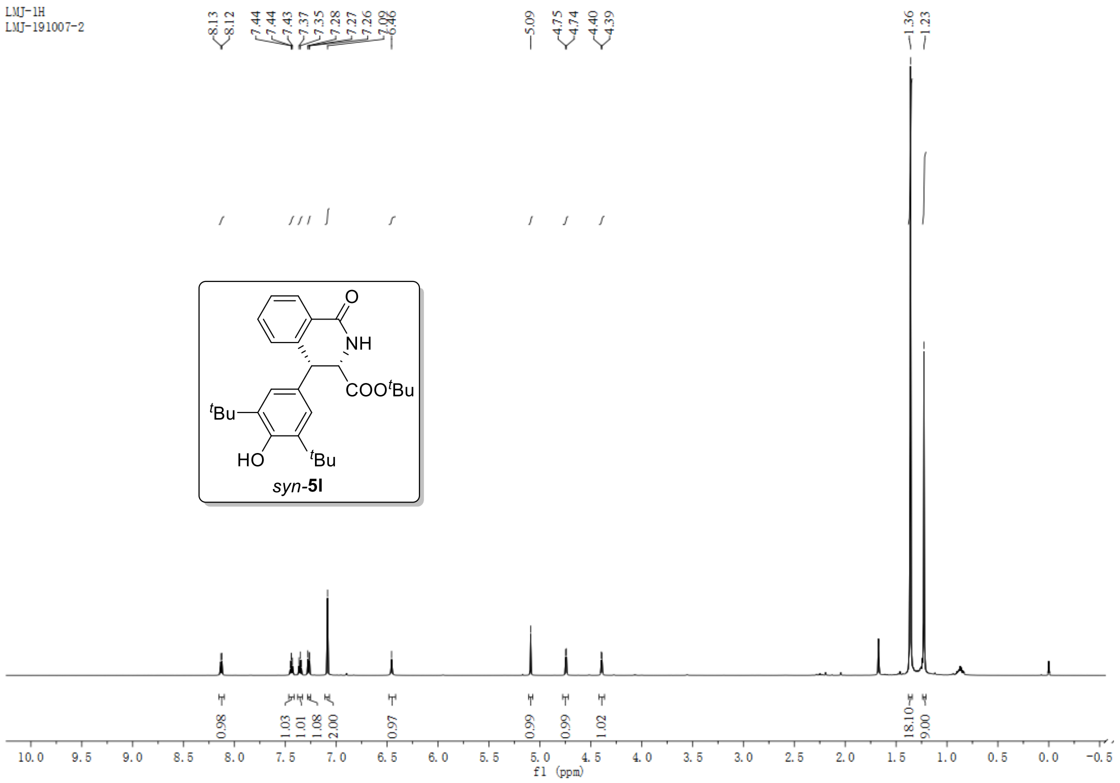
LMJ-1H
LMJ-191007-2
syn-5l
f1 (ppm)

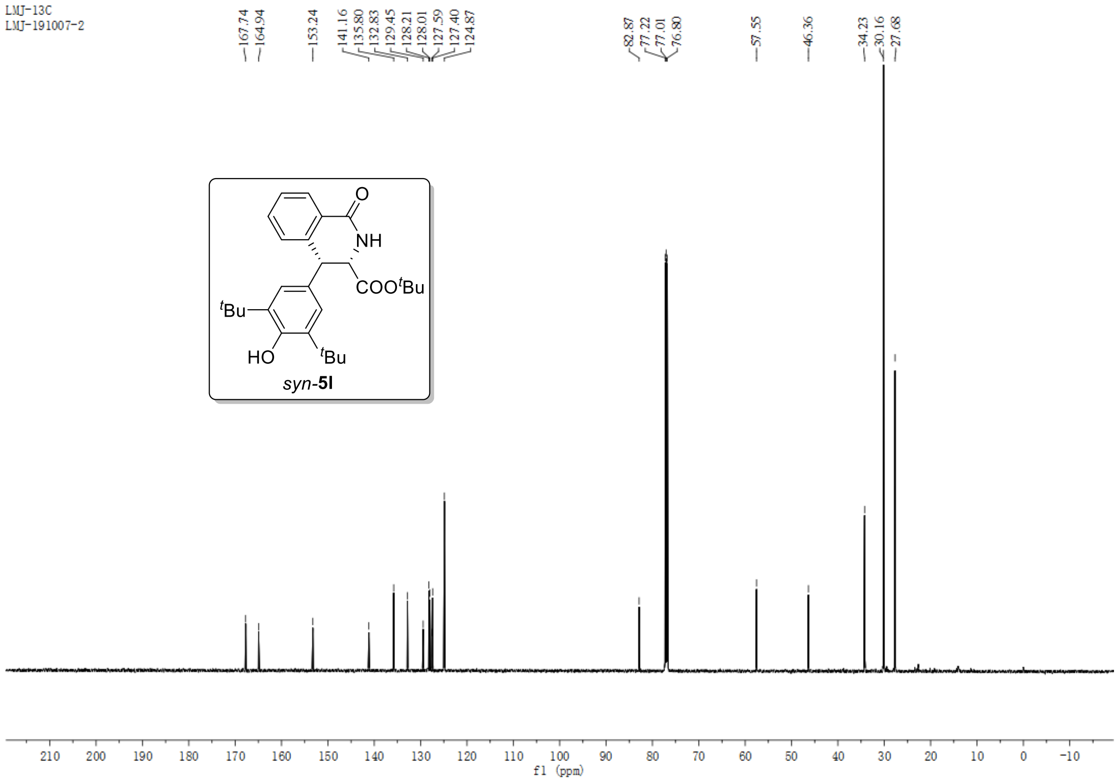
LMJ-13C
LMJ-191007-2
syn-5l
f1 (ppm)

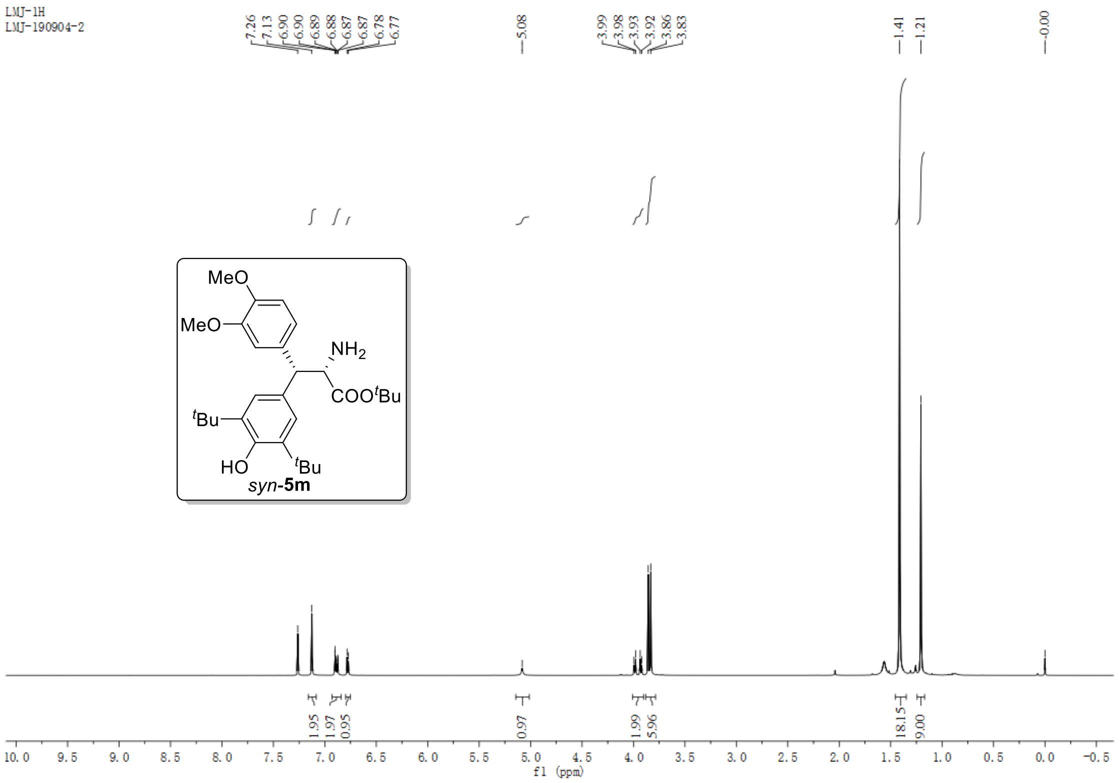
LMJ-1H
LMJ-190904-2
7.26
7.13
6.90
6.90
6.89
6.88
6.87
6.87
6.78
6.77
5.08
3.99
3.98
3.93
3.92
3.86
3.83
1.41
1.21
0.00
MeO
MeO
NH2
COOtBu
tBu
HO
tBu
syn-5m
1.95
1.97
0.95
0.97
1.99
5.96
18.15
9.00
f1 (ppm)

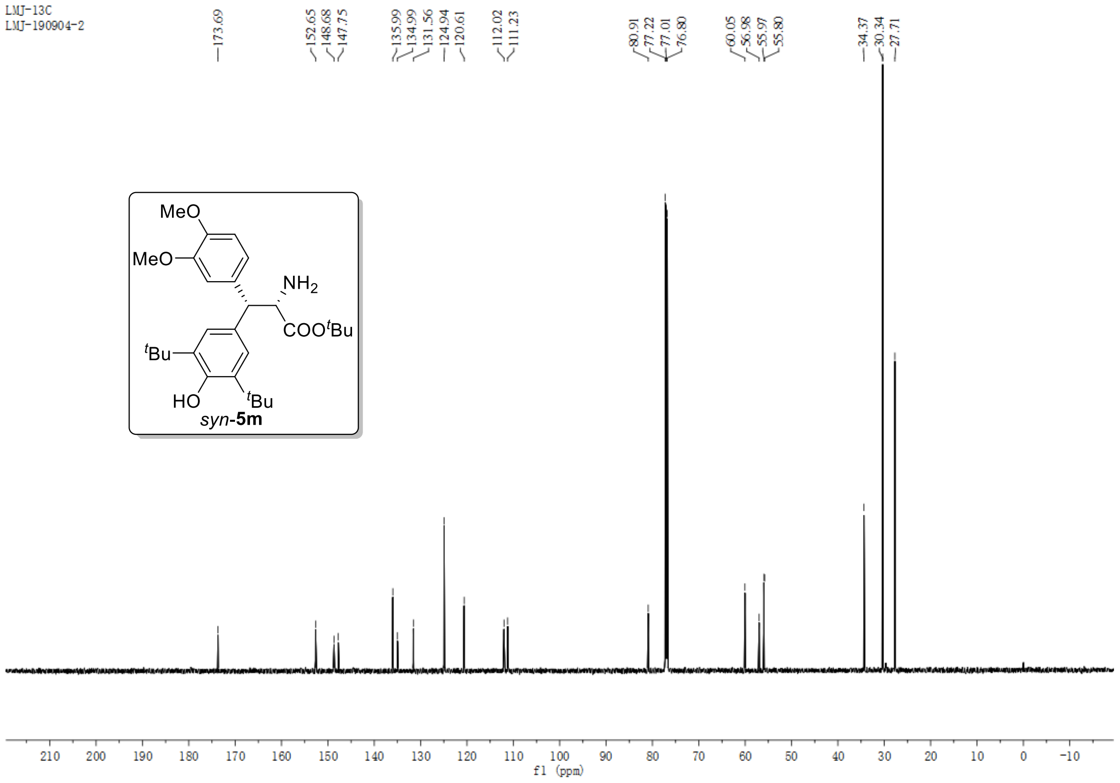
LMJ-13C
LMJ-190904-2
173.69
152.65
148.68
147.75
135.99
134.99
131.56
124.94
120.61
112.02
111.23
80.91
77.22
77.01
76.80
60.05
56.98
55.97
55.80
34.37
30.34
27.71
MeO
MeO
NH2
COOtBu
tBu
HO
tBu
syn-5m
f1 (ppm)

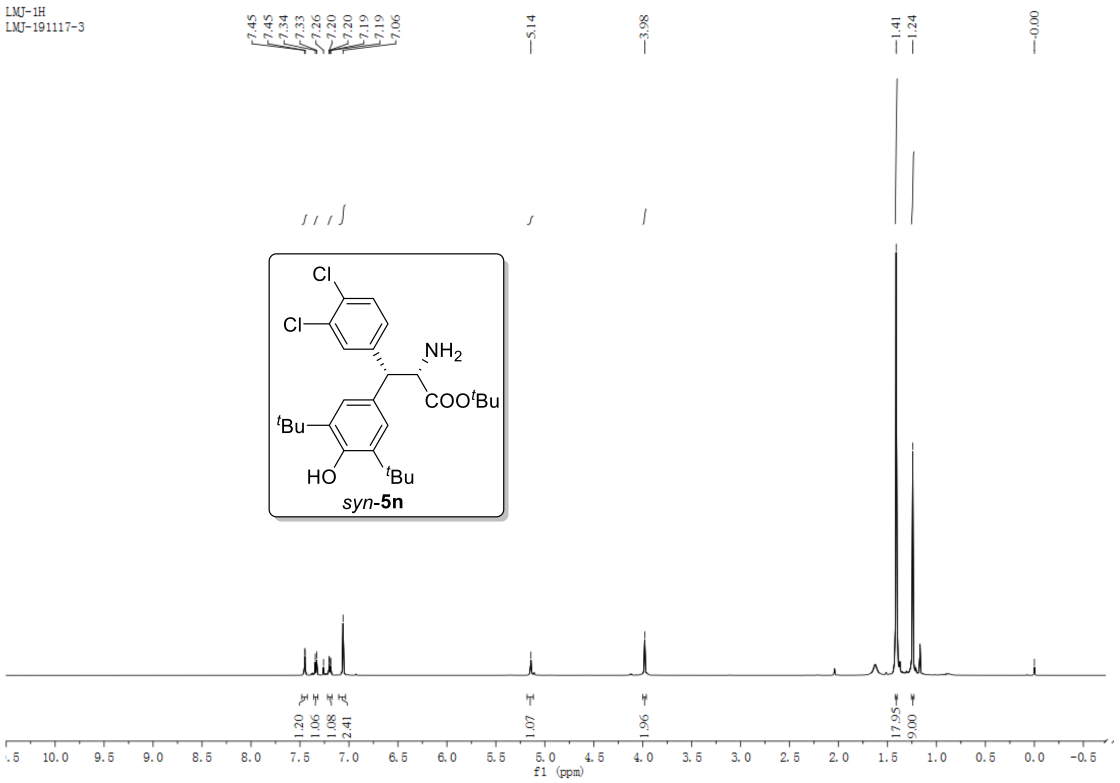
LMJ-1H
LMJ-191117-3
7.45
7.45
7.34
7.33
7.26
7.20
7.20
7.19
7.19
7.06
5.14
3.98
1.41
1.24
0.00
Cl
Cl
NH2
COOtBu
tBu
HO
tBu
syn-5n
1.20
1.06
1.08
2.41
1.07
1.96
17.95
9.00
f1 (ppm)

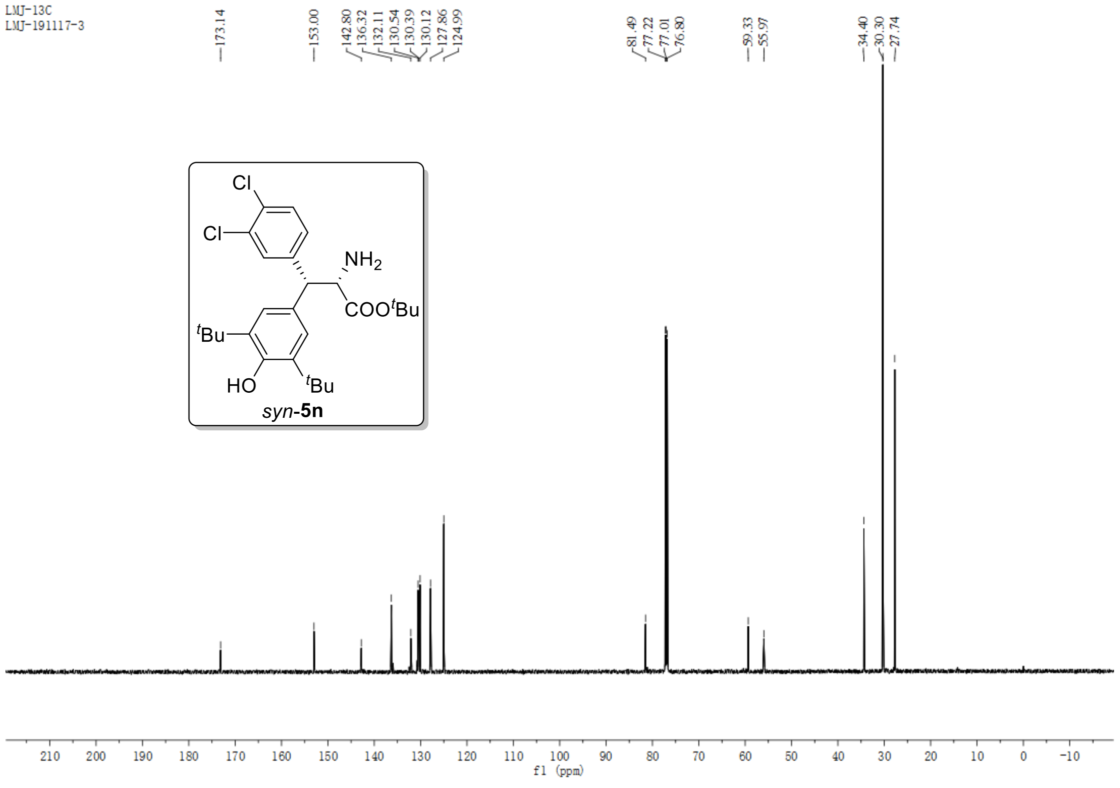
LMJ-13C
LMJ-191117-3
173.14
153.00
142.80
136.32
132.11
130.54
130.39
130.12
127.86
124.99
81.49
77.22
77.01
76.80
59.33
55.97
34.40
30.30
27.74
Cl
Cl
NH2
COOtBu
tBu
HO
tBu
syn-5n
f1 (ppm)

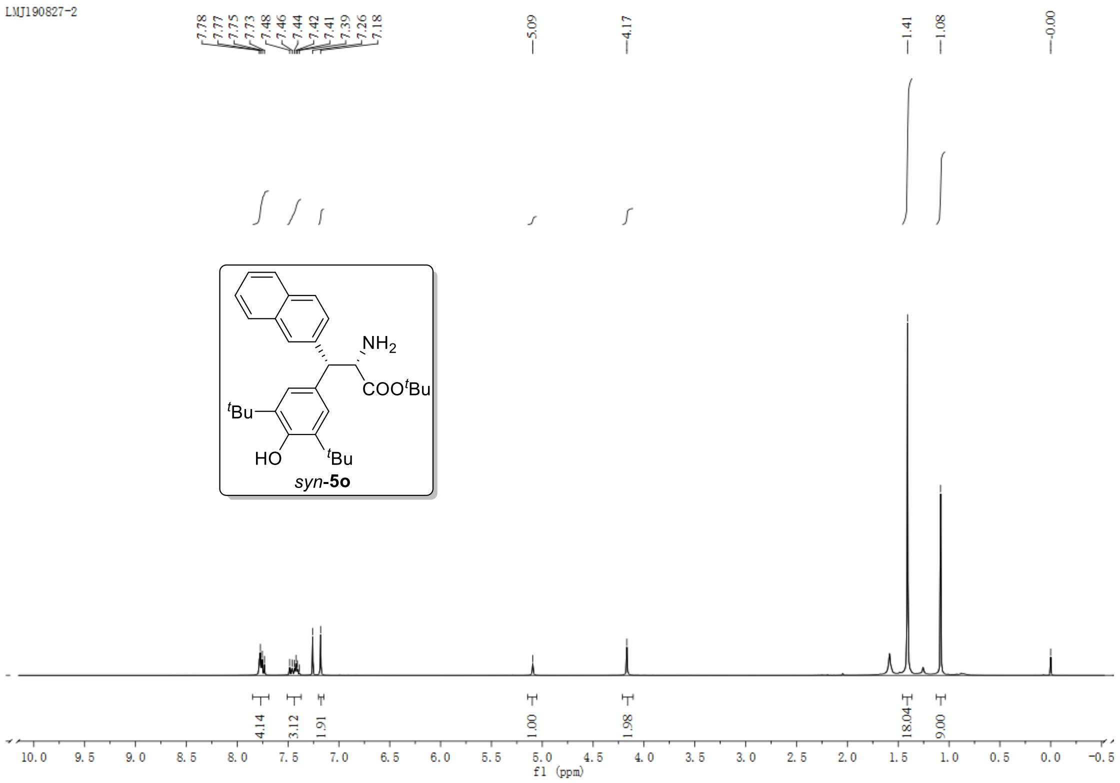
LMJ190827-2
7.78
7.77
7.75
7.73
7.48
7.46
7.44
7.42
7.41
7.39
7.26
7.18
5.09
4.17
1.41
1.08
0.00
NH2
COOtBu
tBu
HO
tBu
syn-5o
4.14
3.12
1.91
1.00
1.98
18.04
9.00
f1 (ppm)

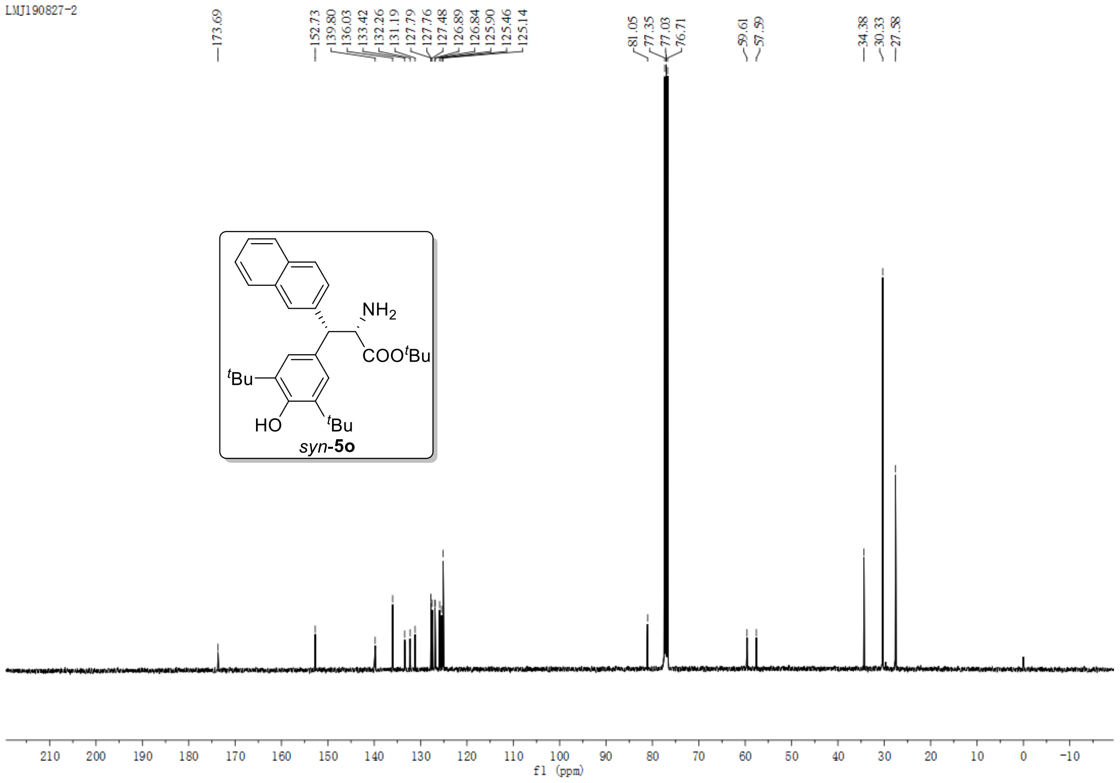
LMJ190827-2
173.69
152.73
139.80
136.03
133.42
132.26
131.19
127.79
127.76
127.48
126.89
126.84
125.90
125.46
125.14
81.05
77.35
77.03
76.71
59.61
57.59
34.38
30.33
27.58
NH2
COOtBu
tBu
HO
tBu
syn-5o
f1 (ppm)

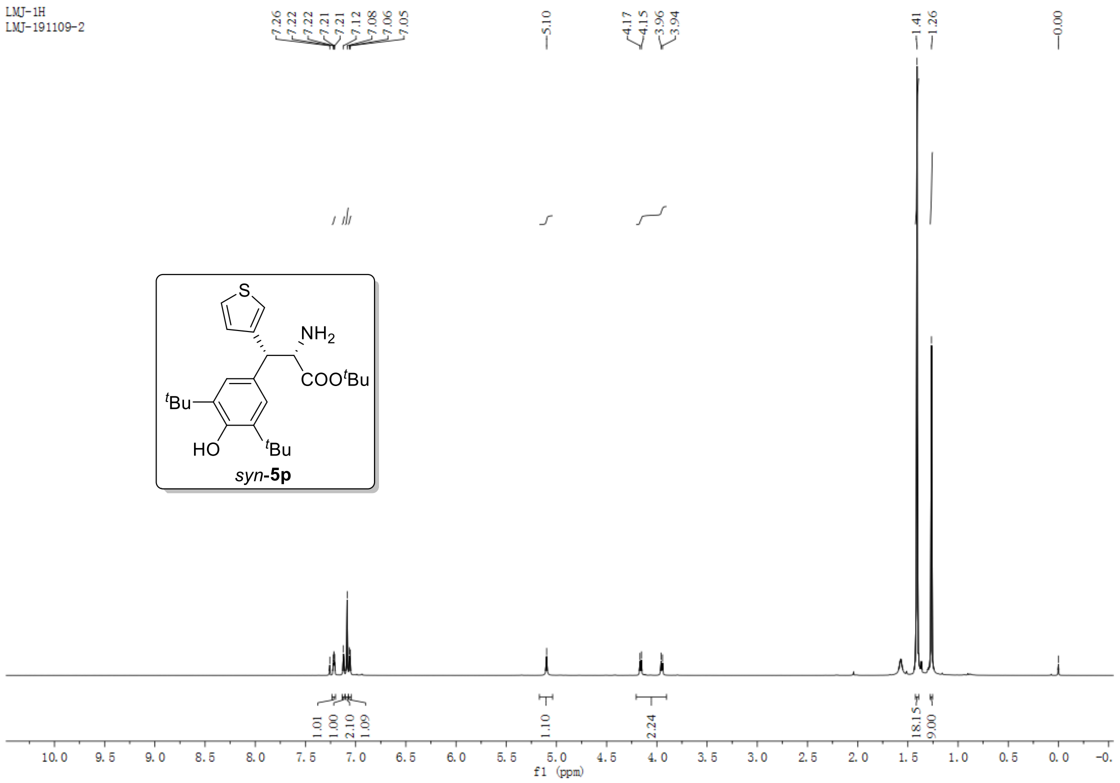
LMJ-1H
LMJ-191109-2
7.26 7.22 7.22 7.21 7.21 7.12 7.08 7.06 7.05
5.10
4.17 4.15 3.96 3.94
1.41 1.26
0.00
S
NH2
COOtBu
tBu
HO
tBu
syn-5p
1.01 1.00 2.10 1.09
1.10
2.24
18.15 9.00
f1 (ppm)

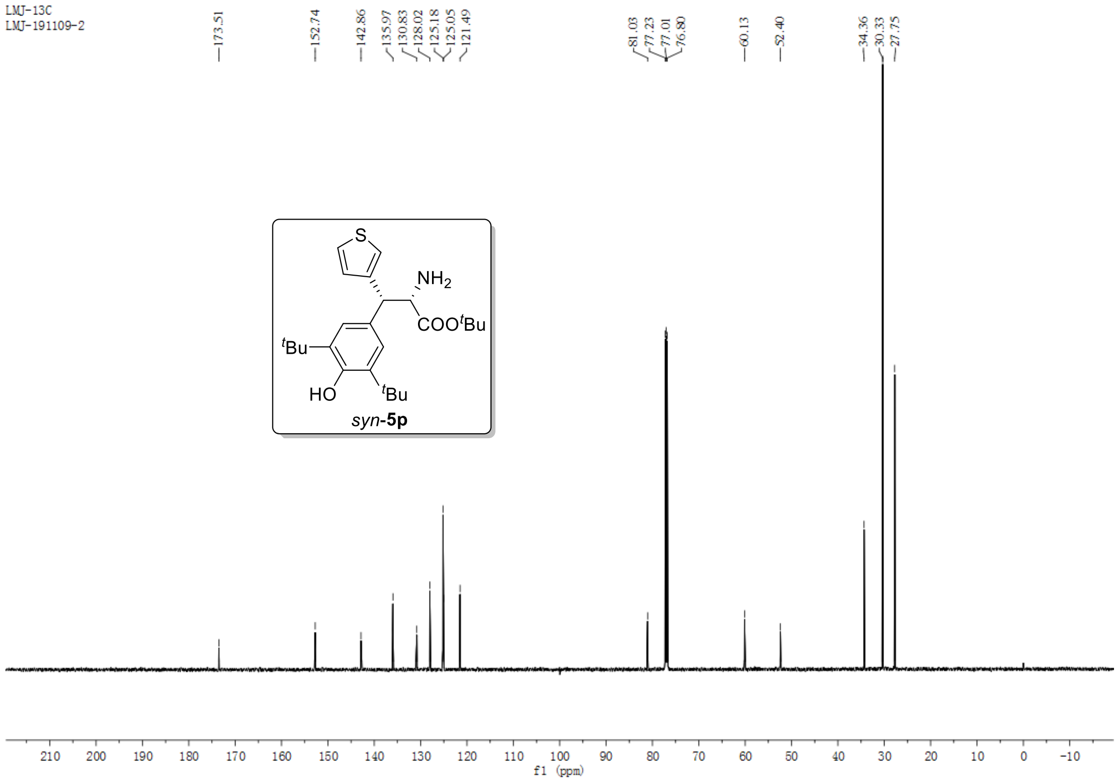
LMJ-13C
LMJ-191109-2
173.51
152.74
142.86 135.97 130.83 128.02 125.18 125.05 121.49
81.03 77.23 77.01 76.80
60.13
52.40
34.36 30.33 27.75
S
NH2
COOtBu
tBu
HO
tBu
syn-5p
f1 (ppm)

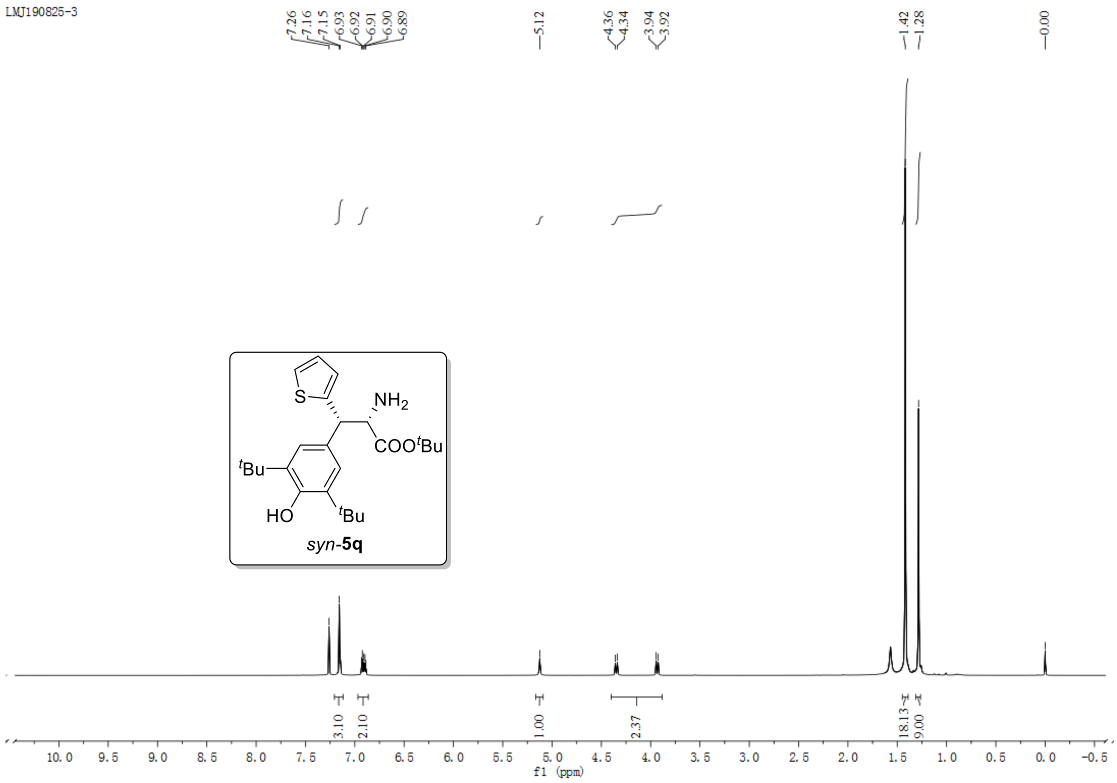
LMJ190825-3
7.26
7.16
7.15
6.93
6.92
6.91
6.90
6.89
5.12
4.36
4.34
3.94
3.92
1.42
1.28
0.00
NH2
COOtBu
tBu
HO
tBu
syn-5q
3.10
2.10
1.00
2.37
18.13
9.00
f1 (ppm)

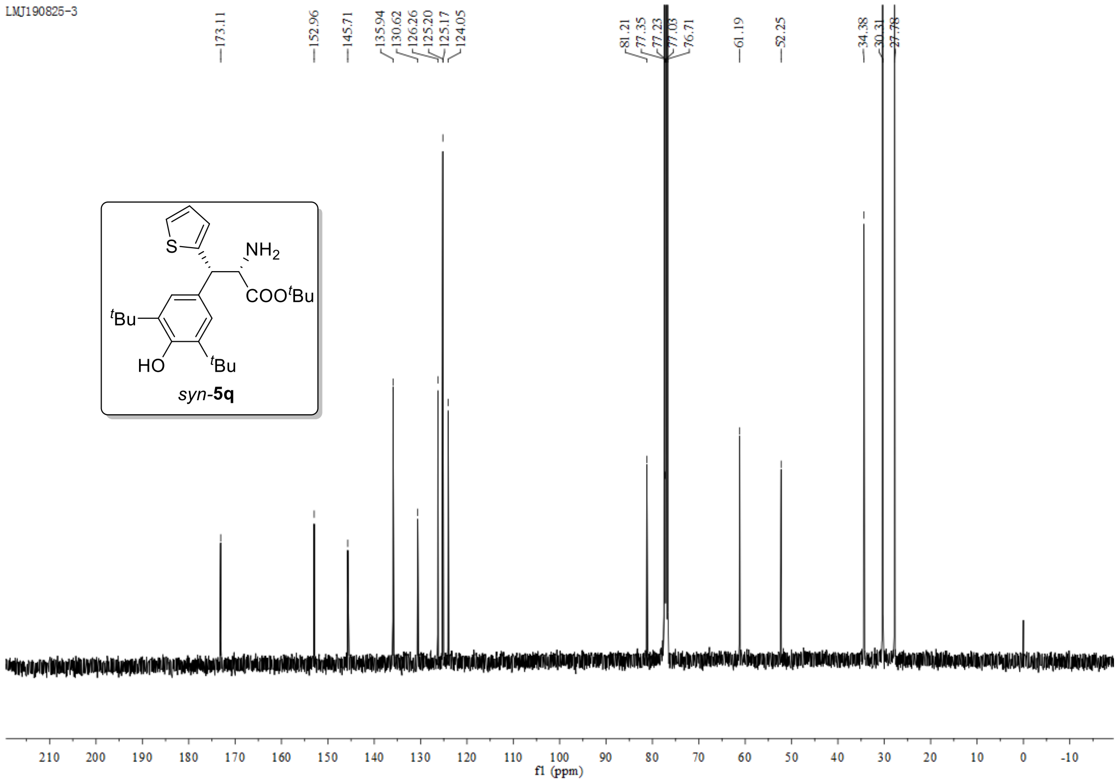
LMJ190825-3
173.11
152.96
145.71
135.94
130.62
126.26
125.20
125.17
124.05
81.21
77.35
77.23
77.03
76.71
61.19
52.25
34.38
30.31
27.78
NH2
COOtBu
tBu
HO
tBu
syn-5q
f1 (ppm)

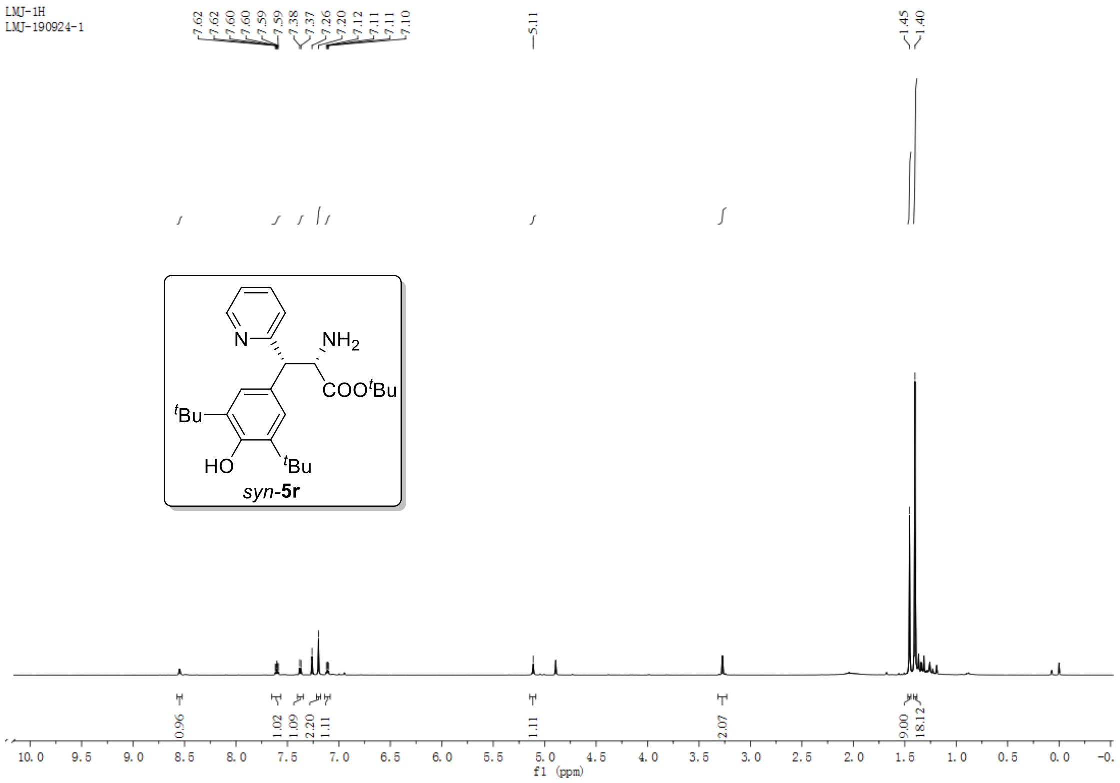
LMJ-1H
LMJ-190924-1
7.62 7.62 7.60 7.60 7.59 7.59 7.38 7.37 7.26 7.20 7.12 7.11 7.11 7.10
5.11
1.45 1.40
NH2
N
COOtBu
tBu
HO
tBu
syn-5r
0.96 1.02 1.09 2.20 1.11 1.11 2.07 9.00 18.12
f1 (ppm)

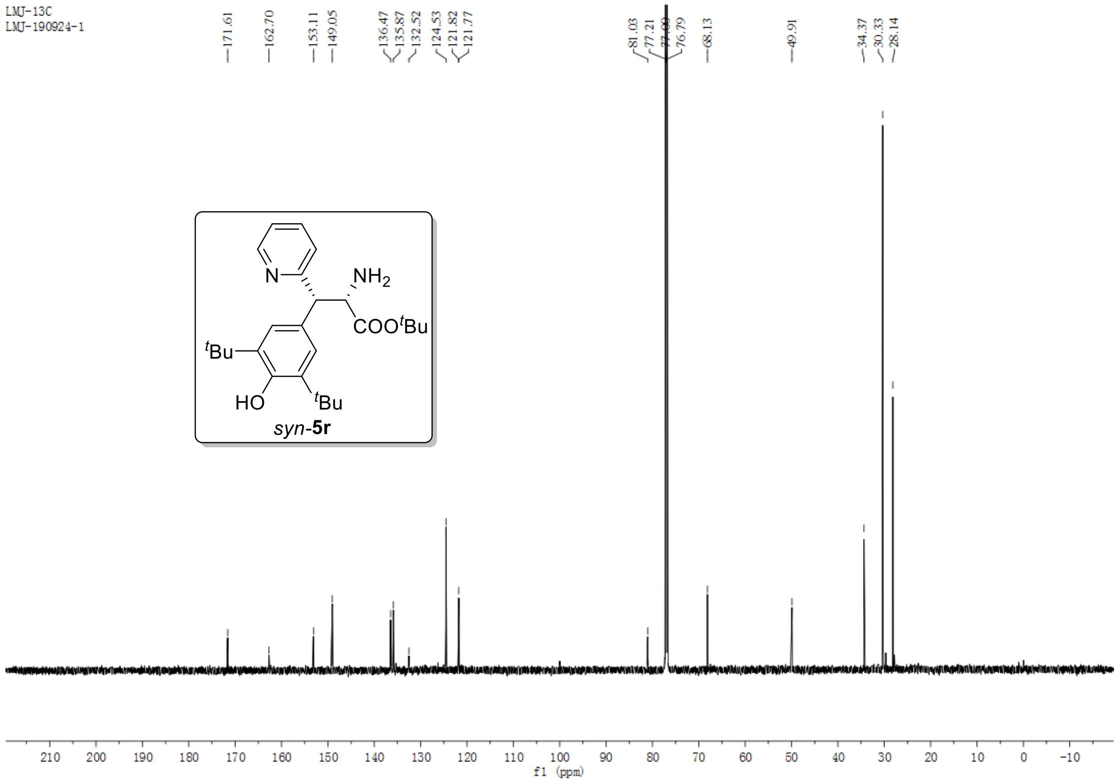
LMJ-13C
LMJ-190924-1
171.61
162.70
153.11
149.05
136.47 135.87 132.52 124.53 121.82 121.77
81.03 77.21 77.00 76.79
68.13
49.91
34.37 30.33 28.14
NH2
N
COOtBu
tBu
HO
tBu
syn-5r
f1 (ppm)

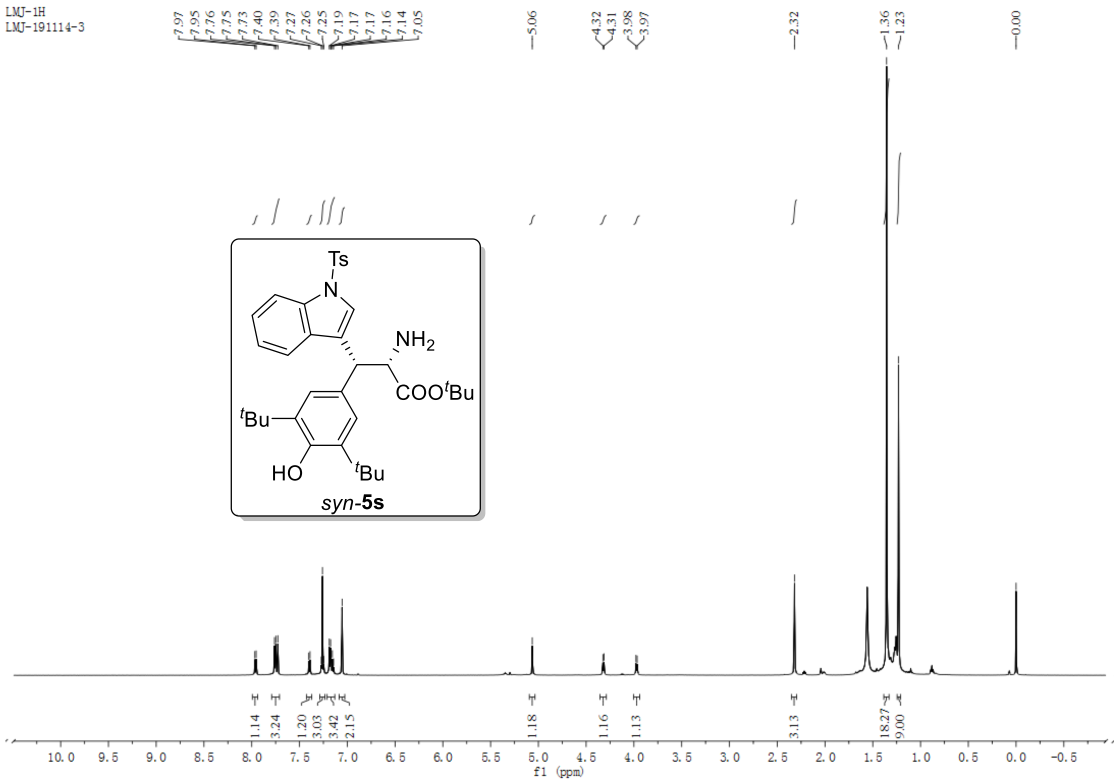
LMJ-1H
LMJ-191114-3
7.97 7.95 7.76 7.75 7.73 7.40 7.39 7.27 7.26 7.25 7.19 7.17 7.17 7.16 7.14 7.05
5.06
4.32 4.31 3.98 3.97
2.32
1.36 1.23
0.00
Ts
N
NH2
COOtBu
tBu
HO
tBu
syn-5s
1.14 3.24 1.20 3.03 3.42 2.15 1.18 1.16 1.13 3.13 18.27 9.00
f1 (ppm)

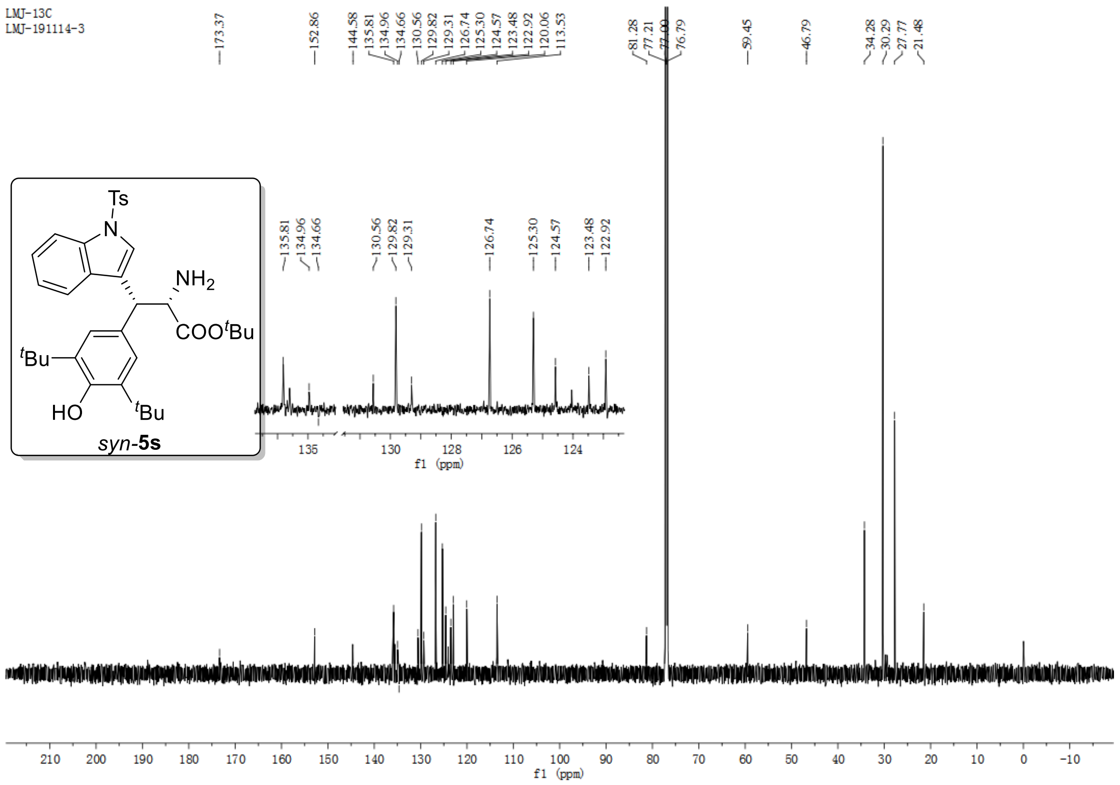
LMJ-13C
LMJ-191114-3
173.37
152.86
144.58 135.81 134.96 134.66 130.56 129.82 129.31 126.74 125.30 124.57 123.48 122.92 120.06 113.53
81.28 77.21 77.00 76.79
59.45
46.79
34.28 30.29 27.77 21.48
Ts
N
NH2
COOtBu
tBu
HO
tBu
syn-5s
135.81 134.96 134.66
130.56 129.82 129.31
126.74
125.30 124.57
123.48 122.92
f1 (ppm)

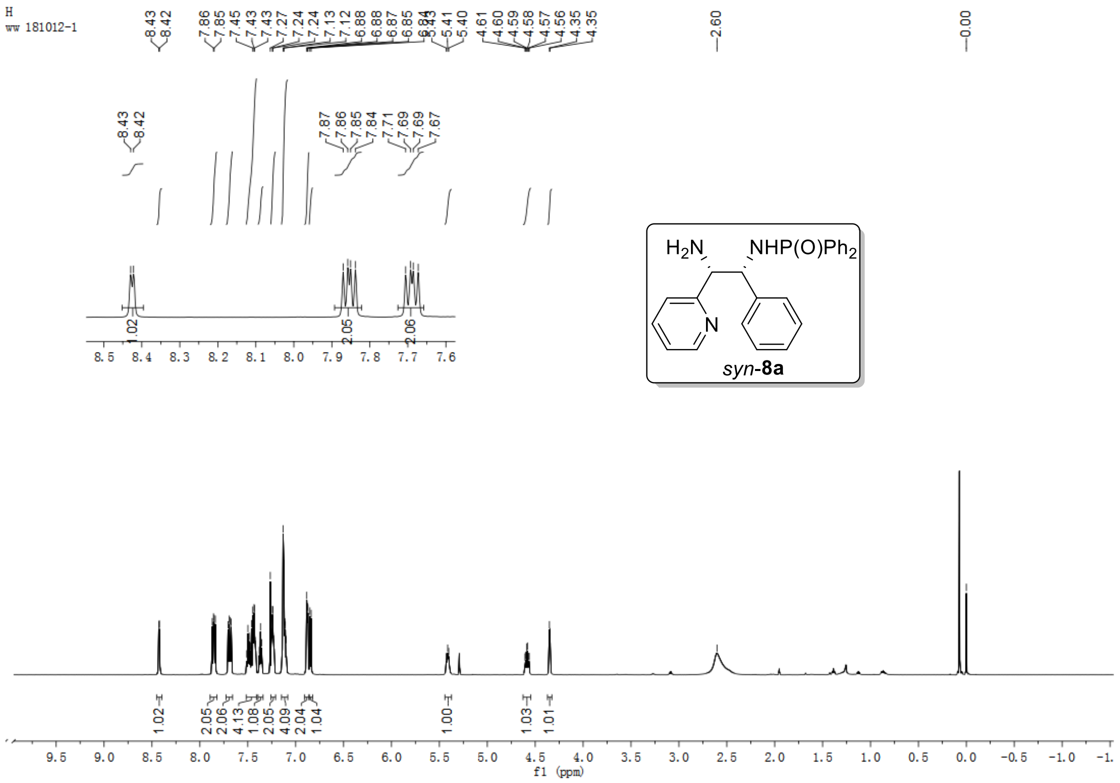

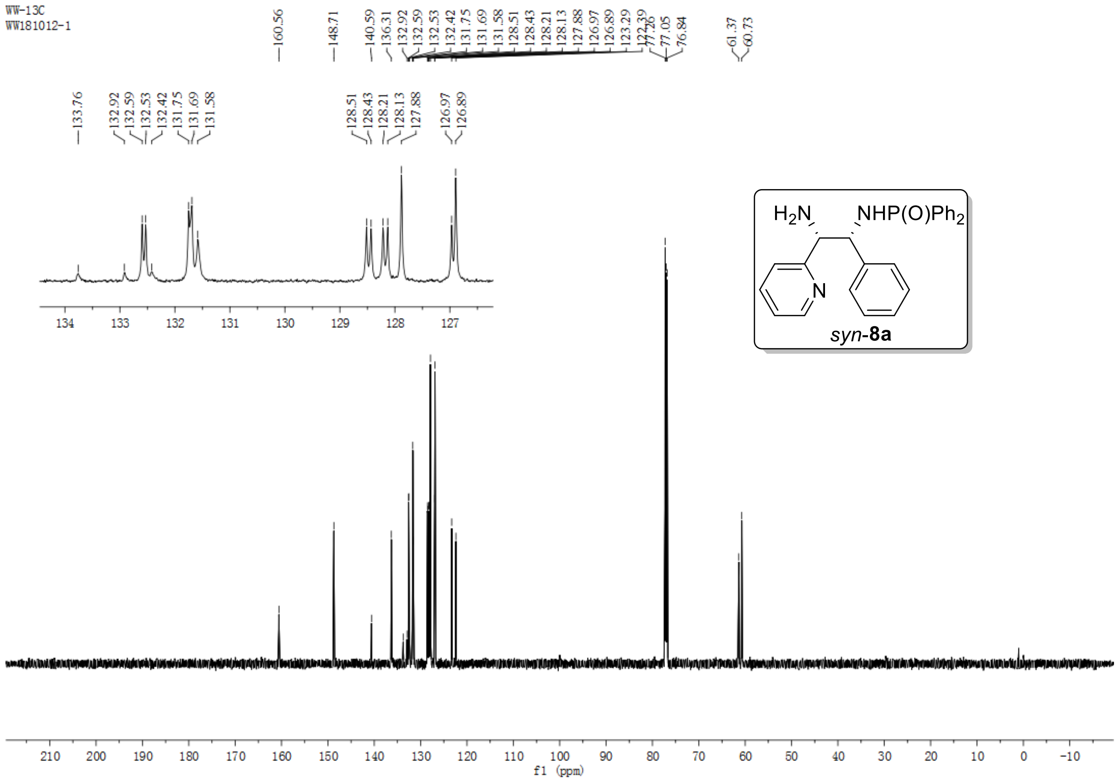

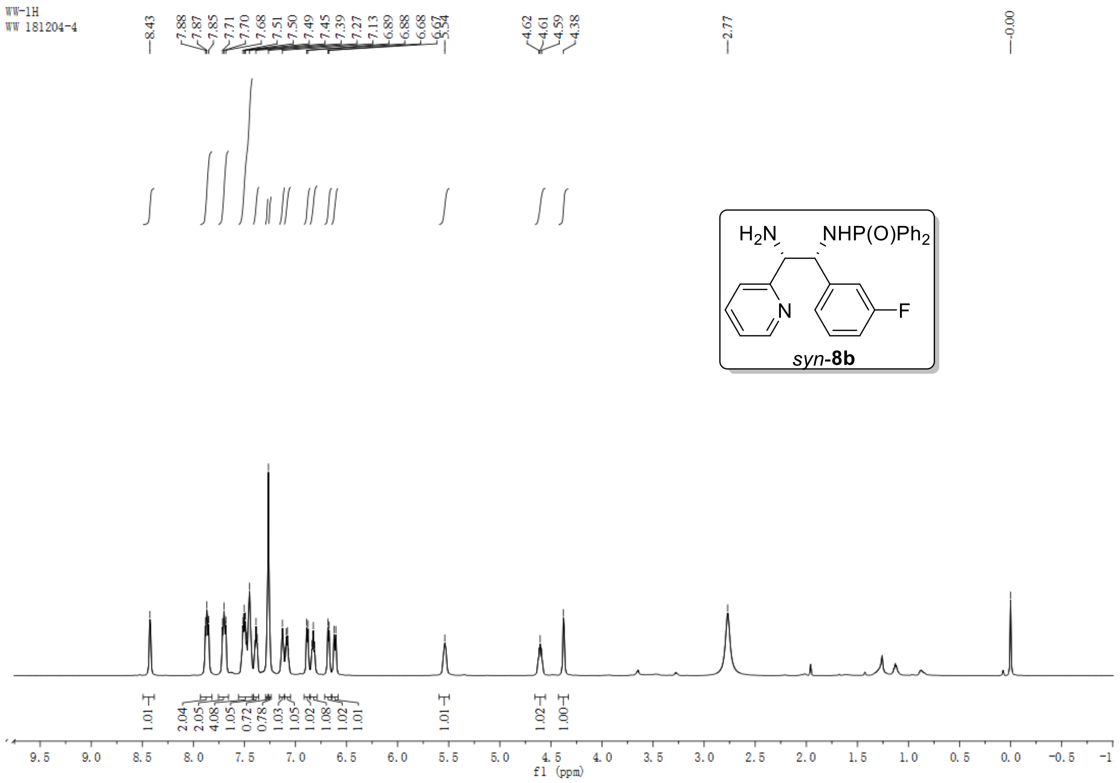

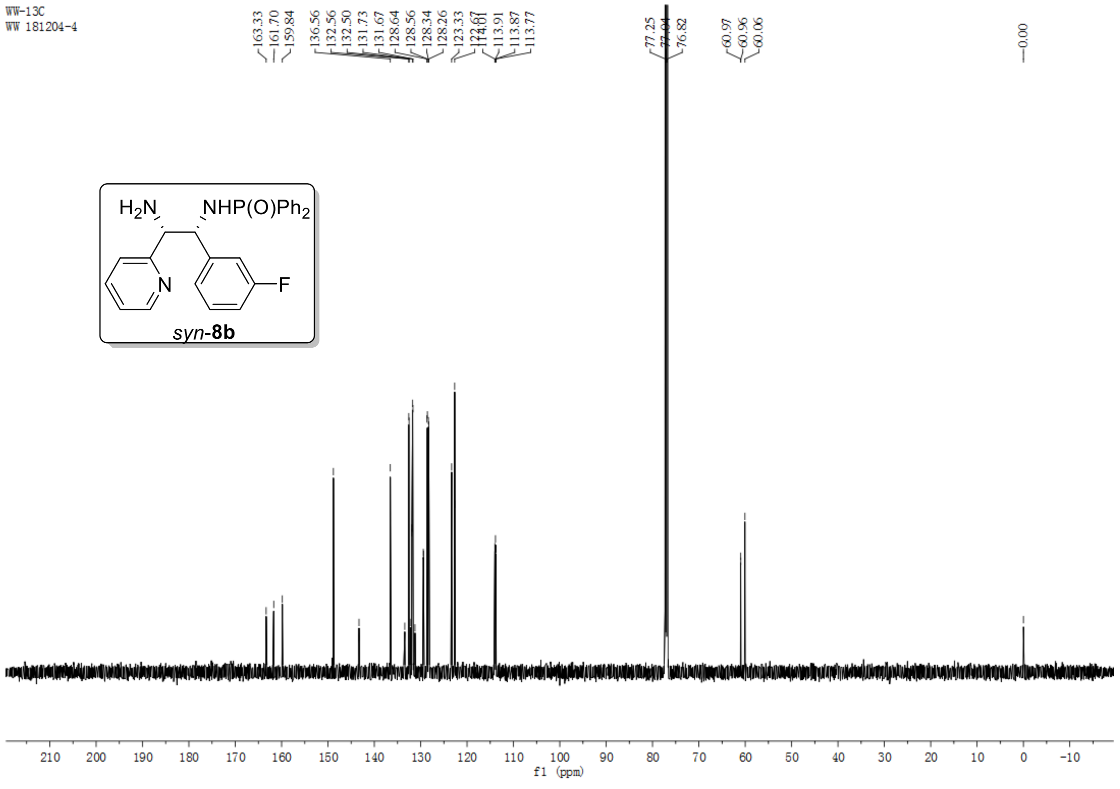

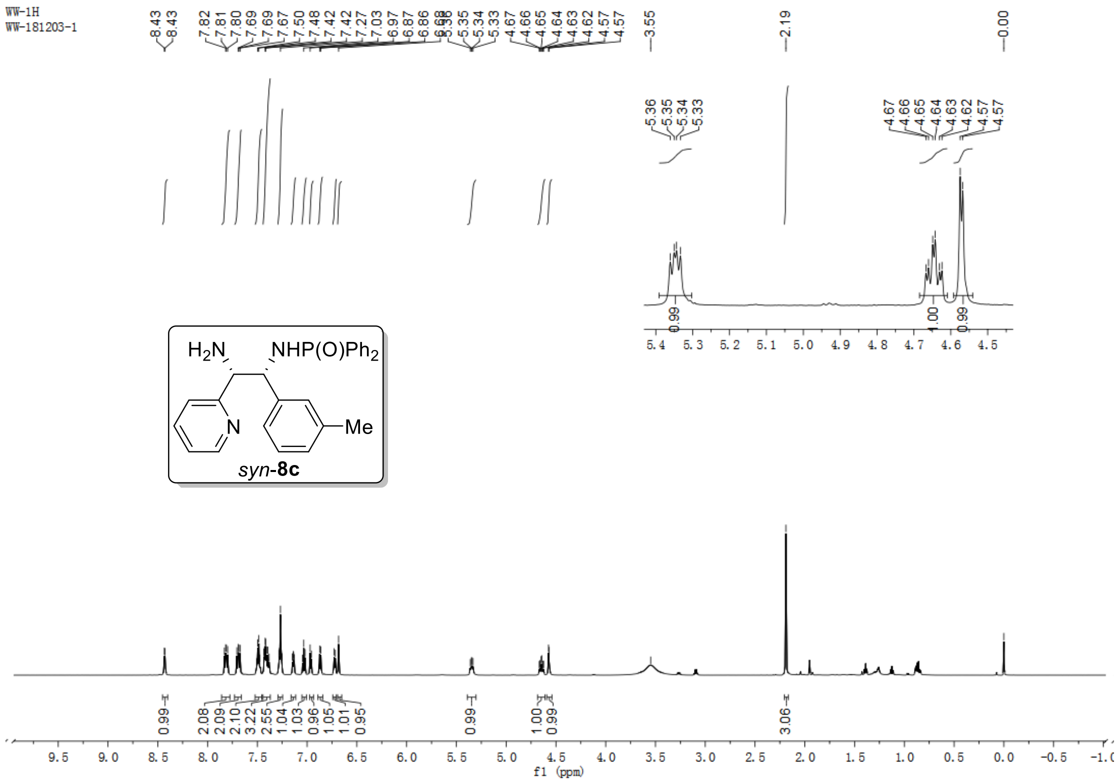
WW-1H
WW-181203-1
8.43
8.43
7.82
7.81
7.80
7.69
7.67
7.50
7.48
7.42
7.42
7.27
7.03
6.97
6.87
6.86
5.36
5.35
5.34
5.33
4.67
4.66
4.65
4.64
4.63
4.62
4.57
4.57
3.55
2.19
0.00
H2N
NHP(O)Ph2
N
Me
syn-8c
0.99
2.08
2.09
2.10
3.22
2.55
1.04
1.03
0.96
1.05
1.01
0.95
0.99
1.00
0.99
3.06
f1 (ppm)

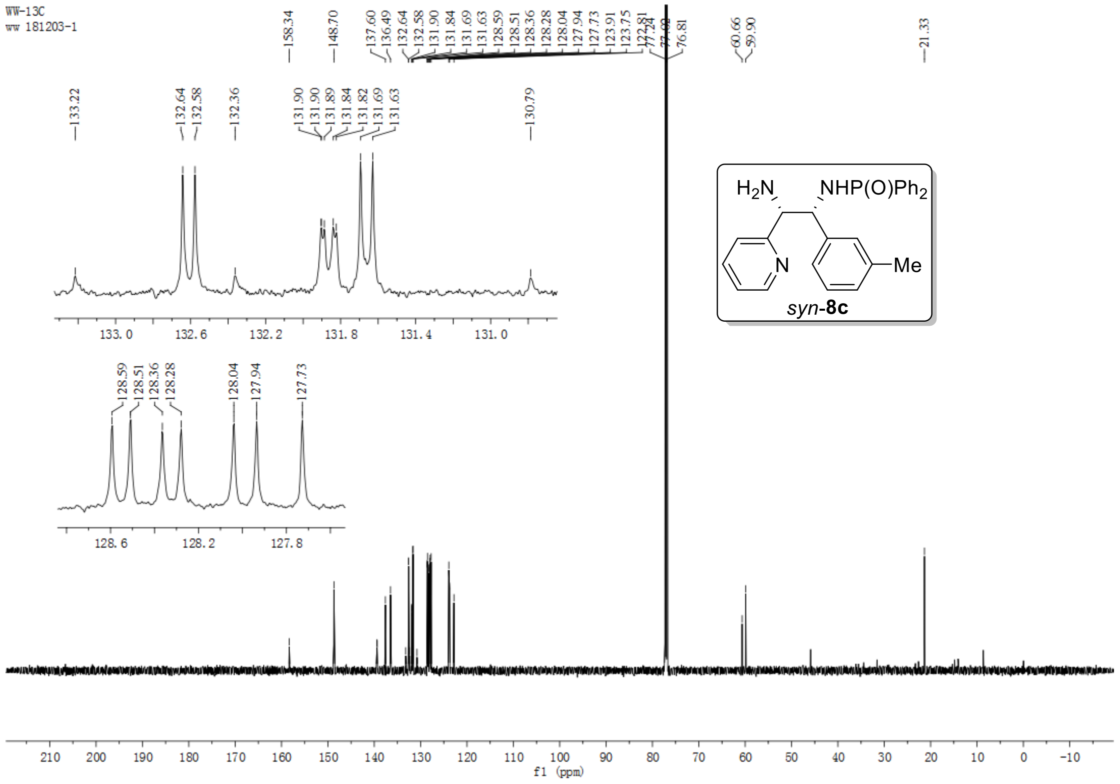
WW-13C
ww 181203-1
158.34
148.70
137.60
136.49
132.64
132.58
131.90
131.84
131.69
131.63
128.59
128.51
128.36
128.28
128.04
127.94
127.73
123.91
123.75
76.81
60.66
59.90
21.33
133.22
132.36
131.89
131.82
130.79
H2N
NHP(O)Ph2
N
Me
syn-8c
f1 (ppm)

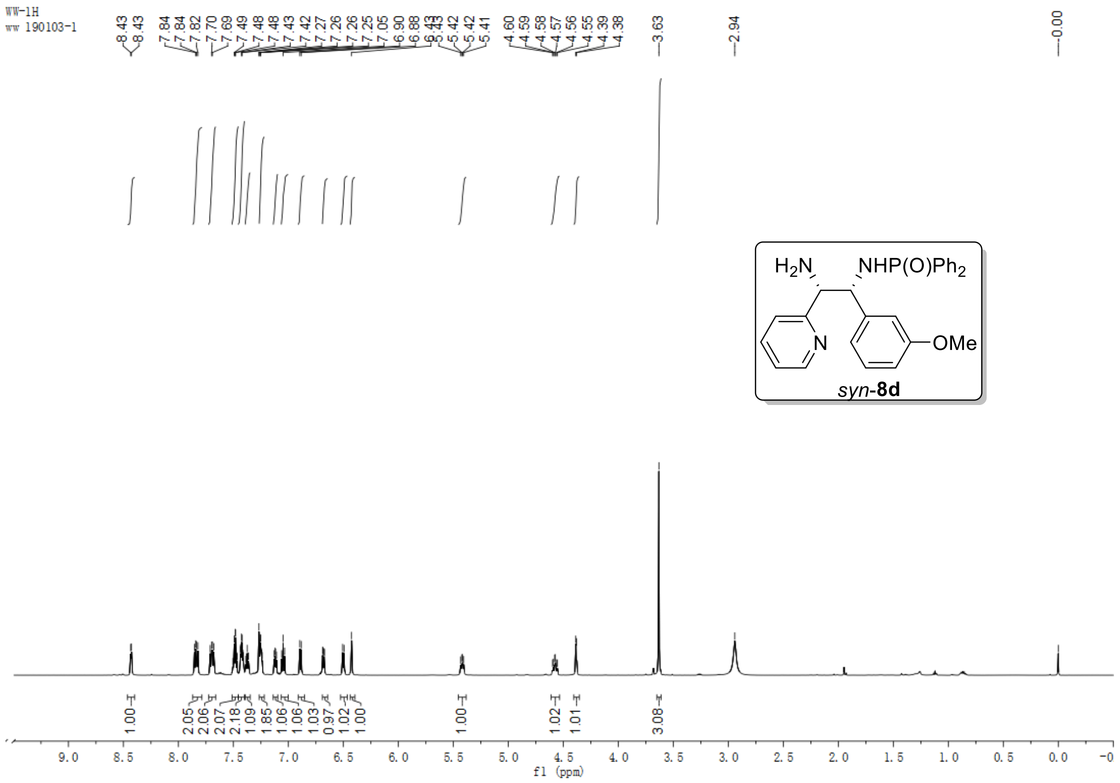
WW-1H
ww 190103-1
H2N
NHP(O)Ph2
N
OMe
syn-8d
f1 (ppm)

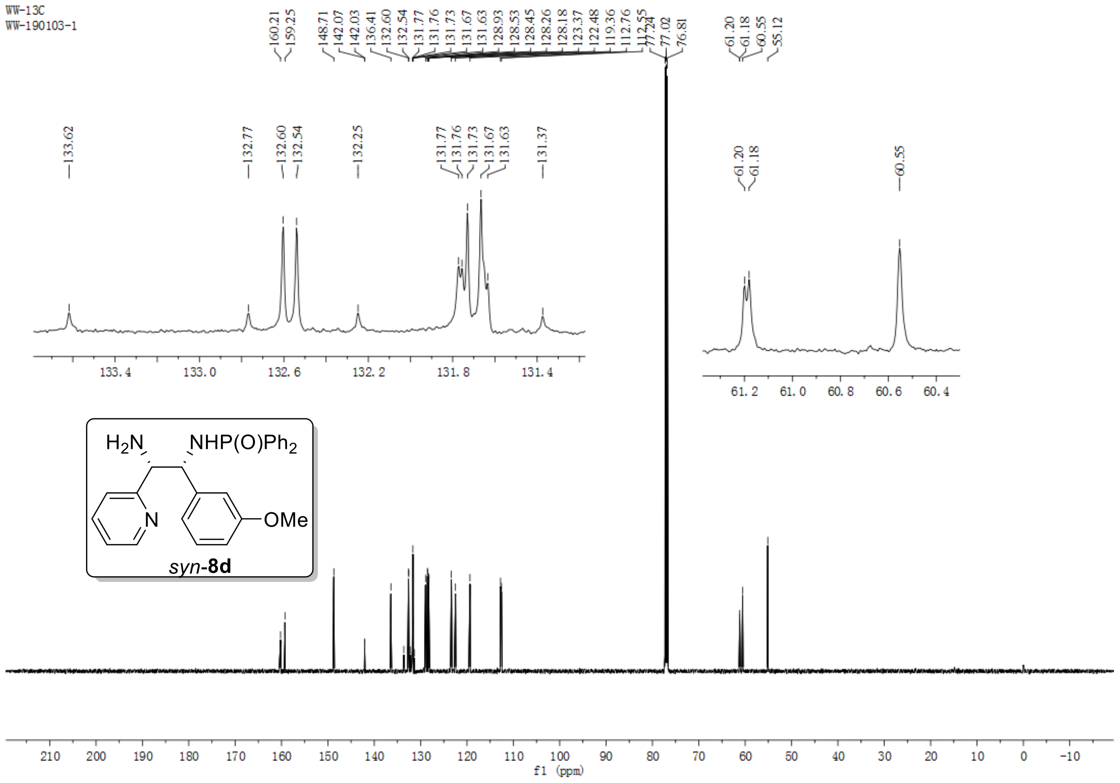
WW-13C
WW-190103-1
H2N
NHP(O)Ph2
N
OMe
syn-8d
f1 (ppm)

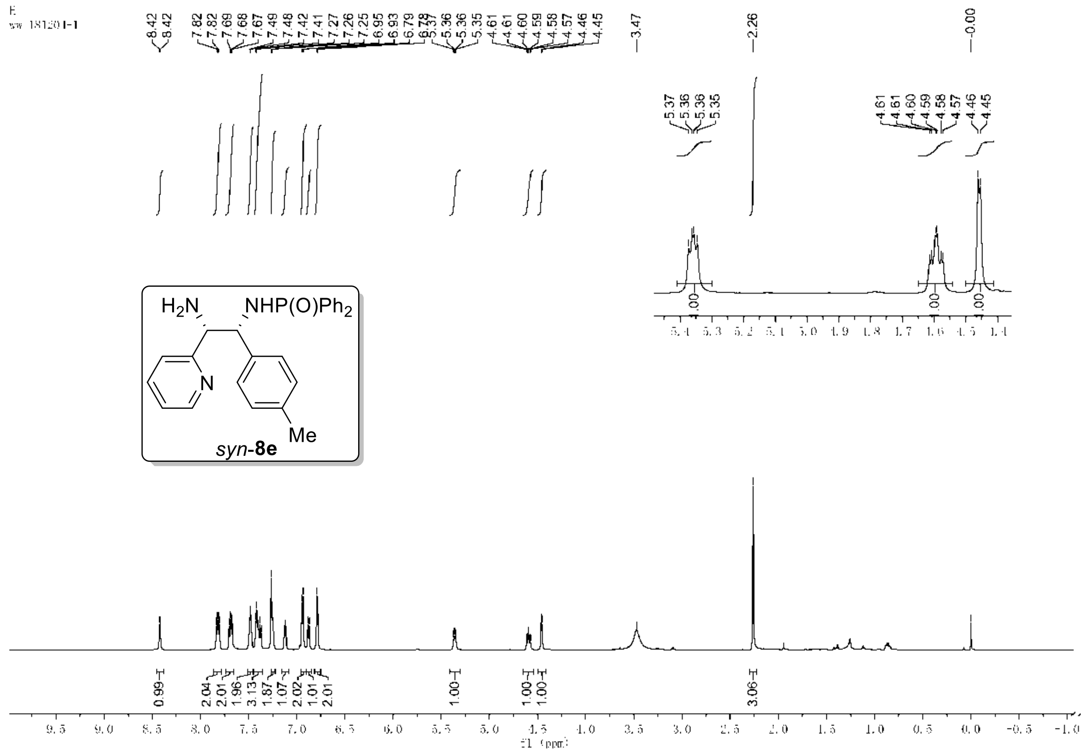
H
ww 181204-1
H2N
NHP(O)Ph2
N
Me
syn-8e
f1 (ppm)

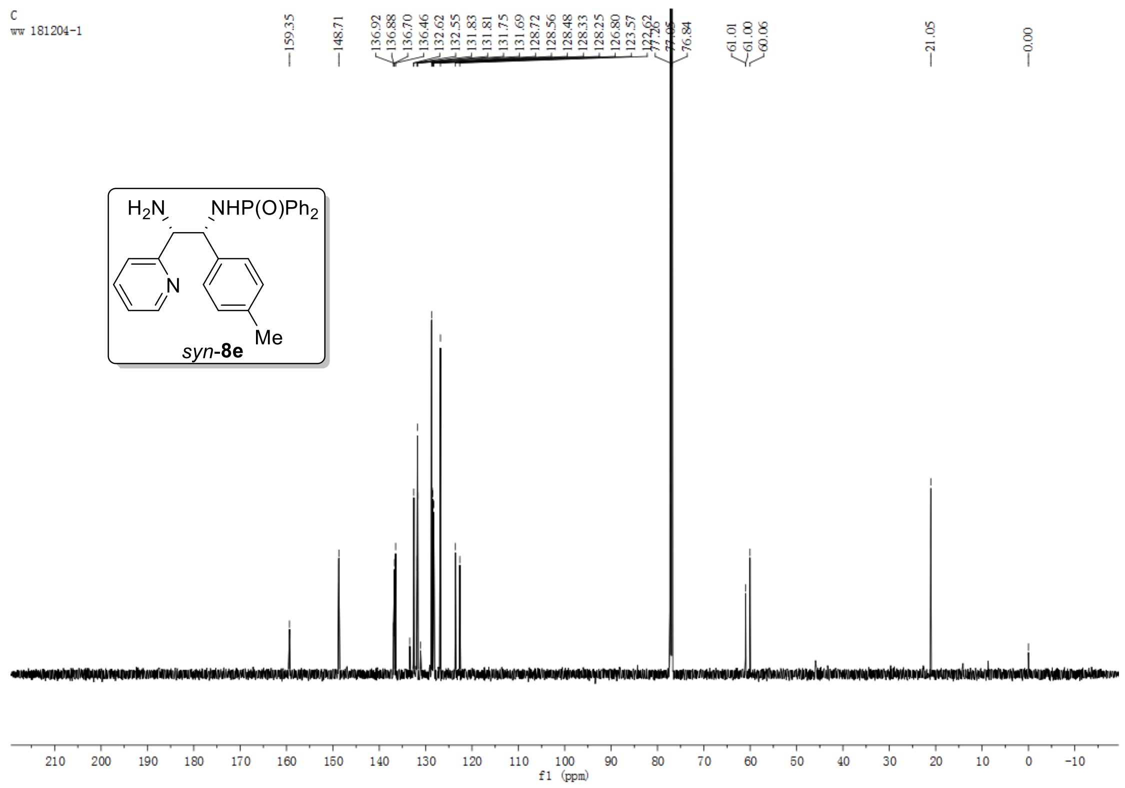
C
ww 181204-1
H2N
NHP(O)Ph2
N
Me
syn-8e
f1 (ppm)

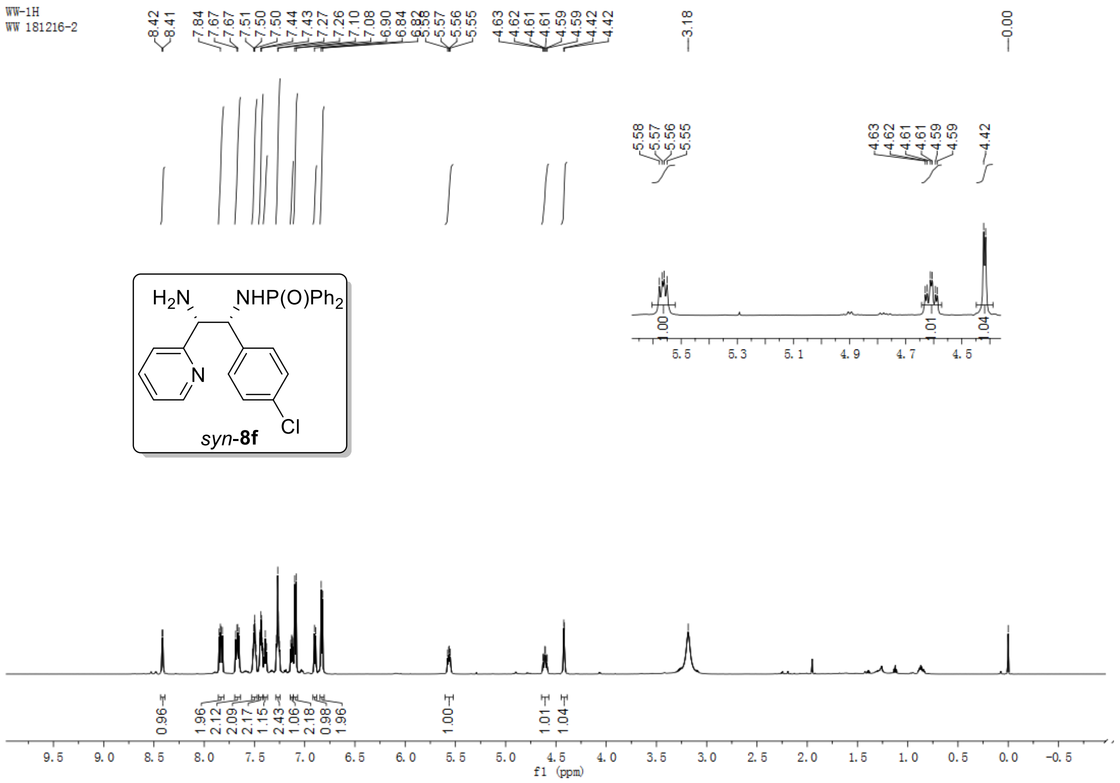
WW-1H
WW 181216-2
H2N
NHP(O)Ph2
N
Cl
syn-8f
f1 (ppm)

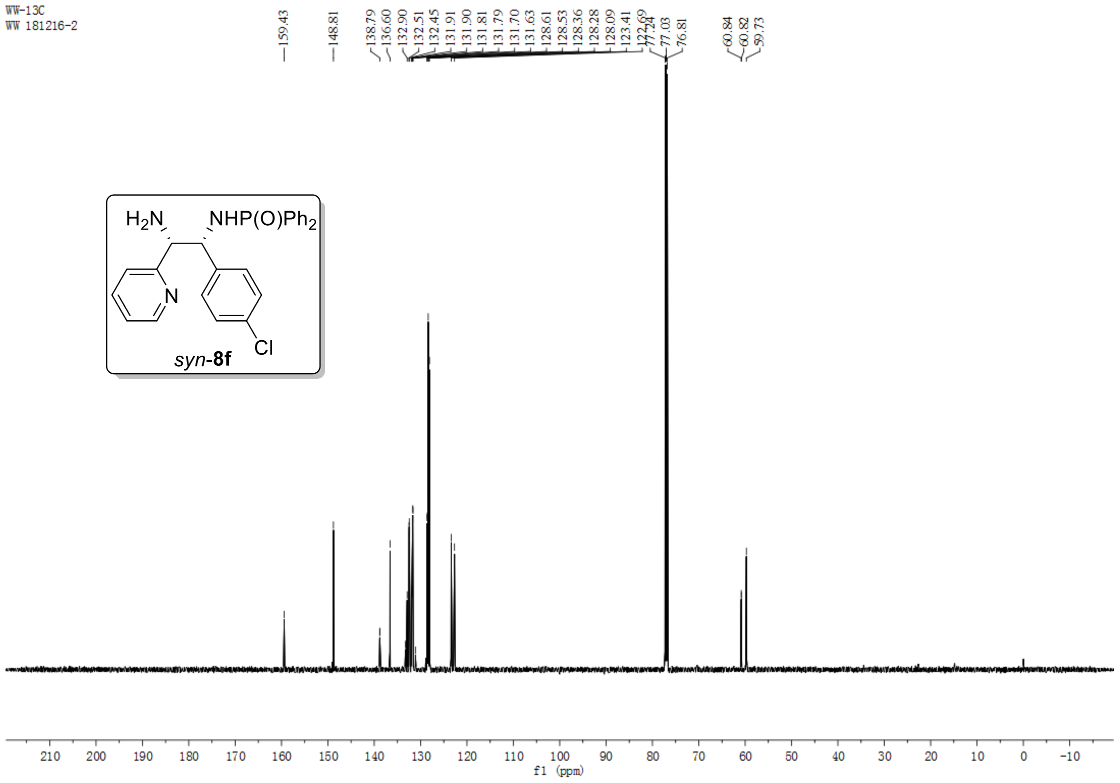
WW-13C
WW 181216-2
H2N
NHP(O)Ph2
N
Cl
syn-8f
f1 (ppm)

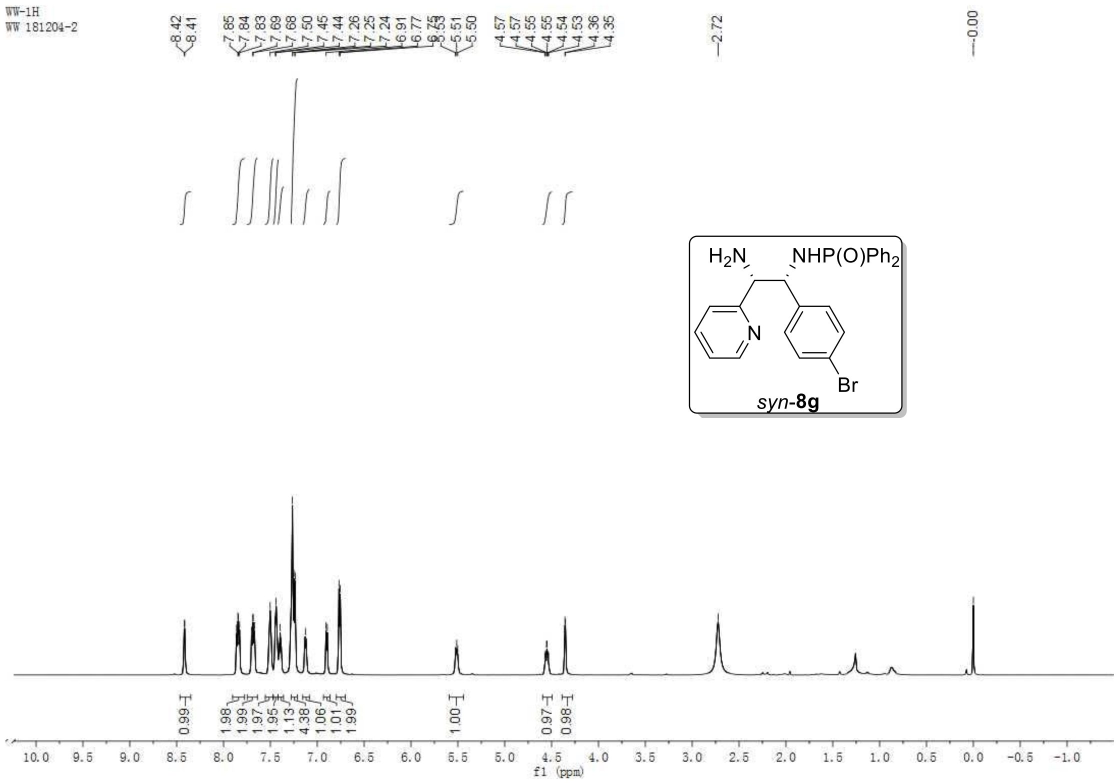
WW-1H
WW 181204-2
H2N
NHP(O)Ph2
N
Br
syn-8g
f1 (ppm)

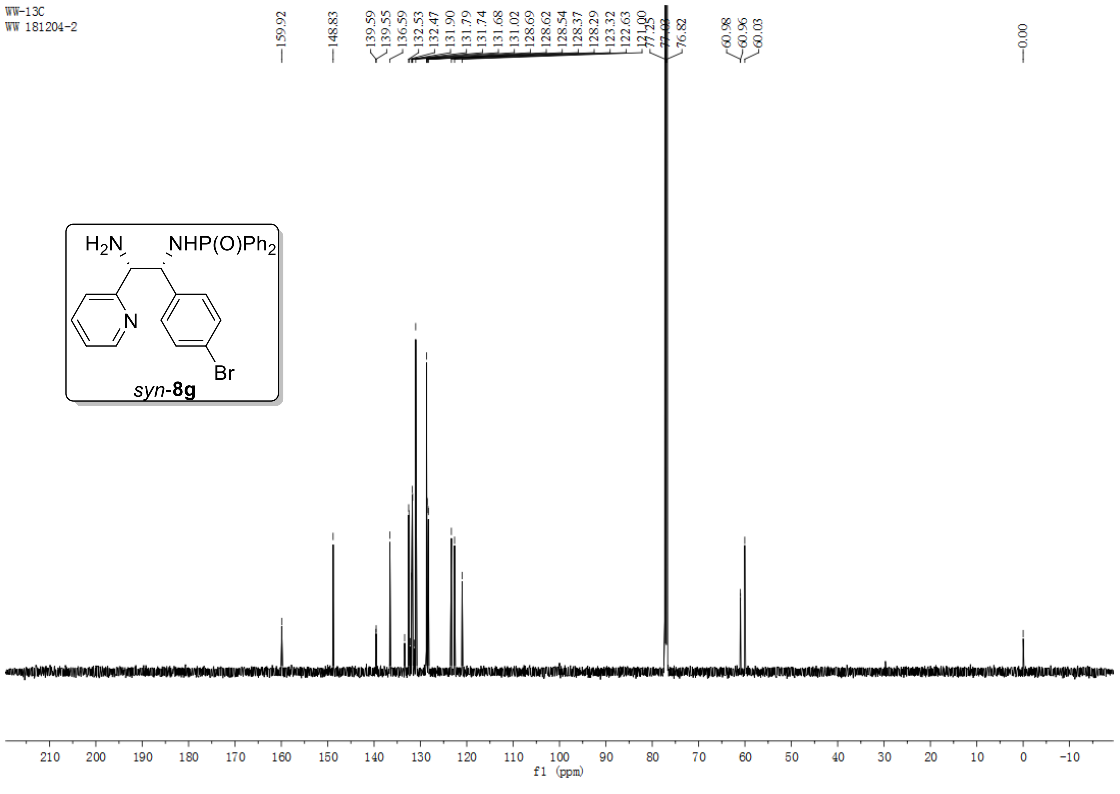
WW-13C
WW 181204-2
H2N
NHP(O)Ph2
N
Br
syn-8g
f1 (ppm)

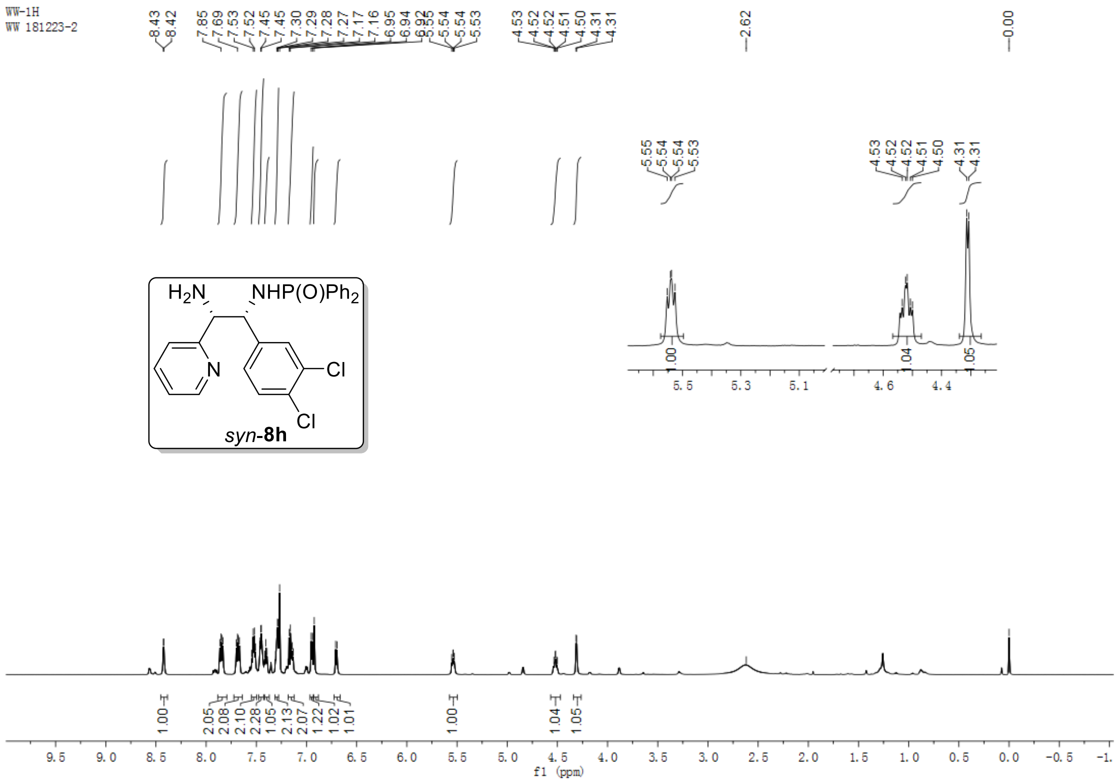
WW-1H
WW 181223-2
H2N
NHP(O)Ph2
N
Cl
Cl
syn-8h
f1 (ppm)

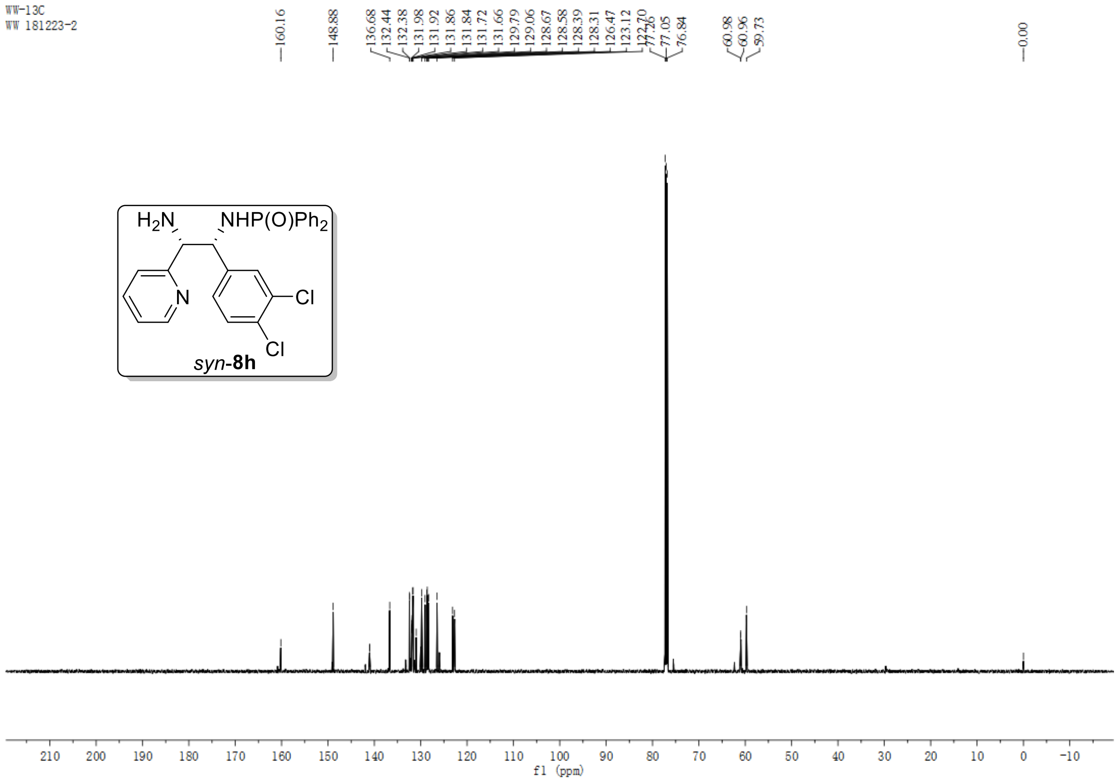
WW-13C
WW 181223-2
H2N
NHP(O)Ph2
N
Cl
Cl
syn-8h
f1 (ppm)

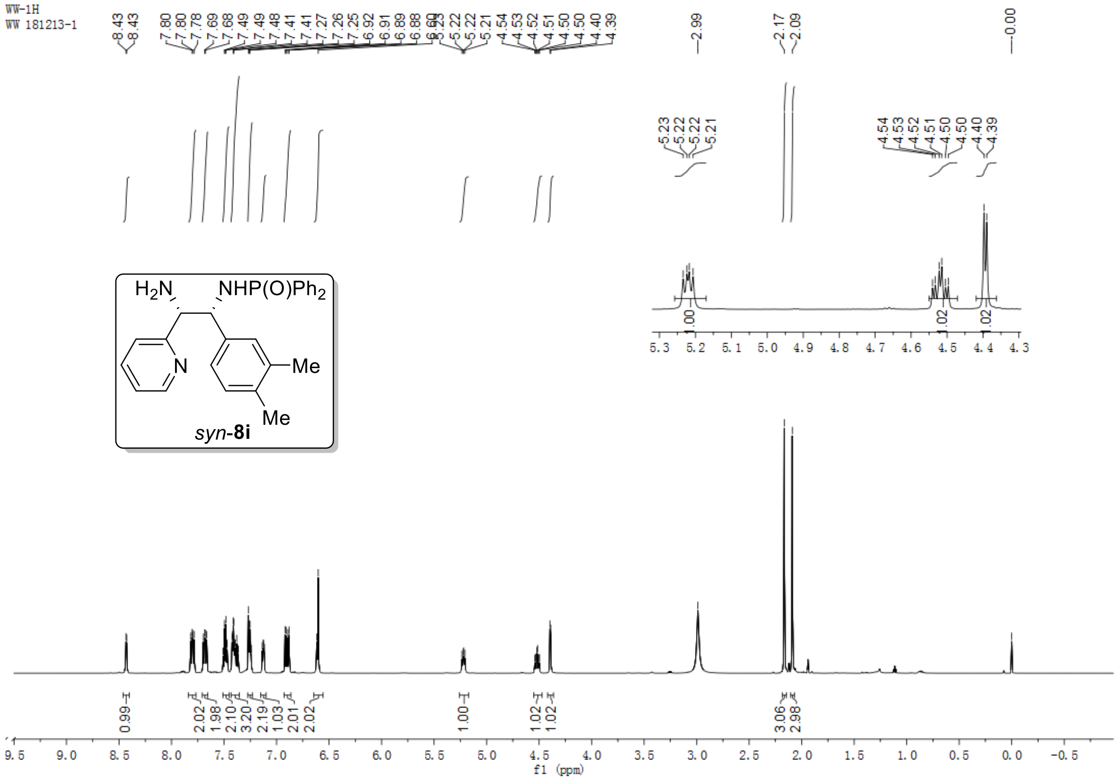
WW-1H
WW 181213-1
H2N
NHP(O)Ph2
N
Me
Me
syn-8i
f1 (ppm)

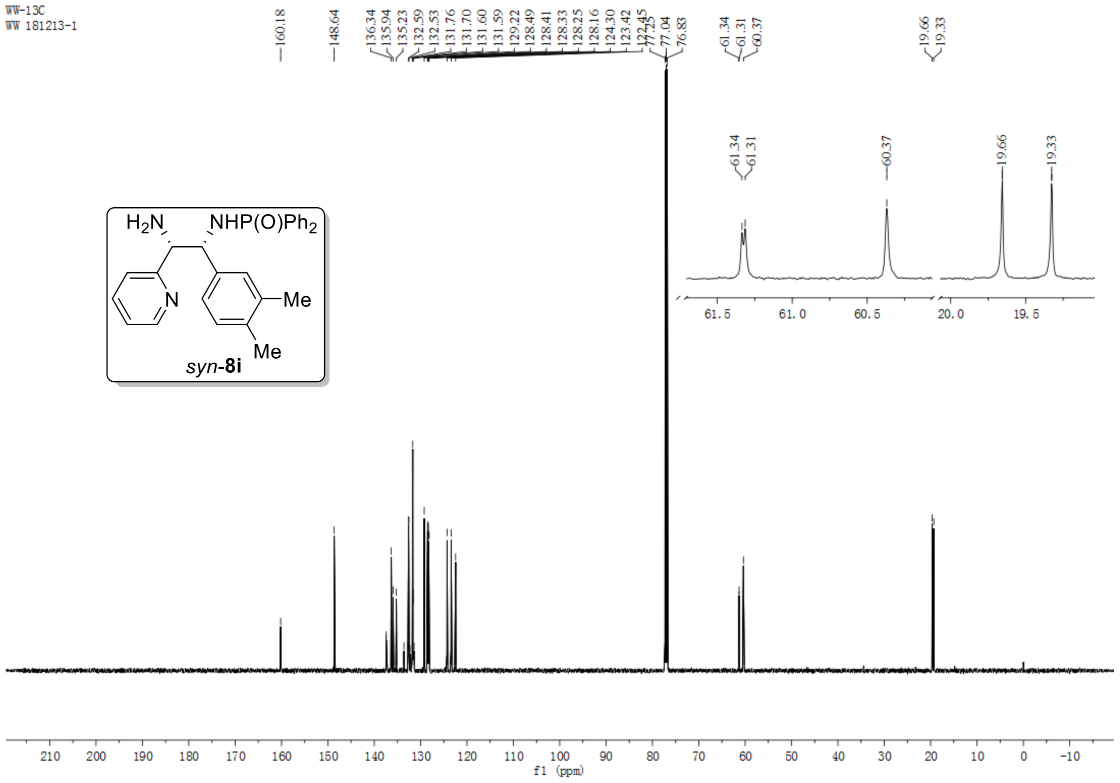
WW-13C
WW 181213-1
H2N
NHP(O)Ph2
N
Me
Me
syn-8i
f1 (ppm)

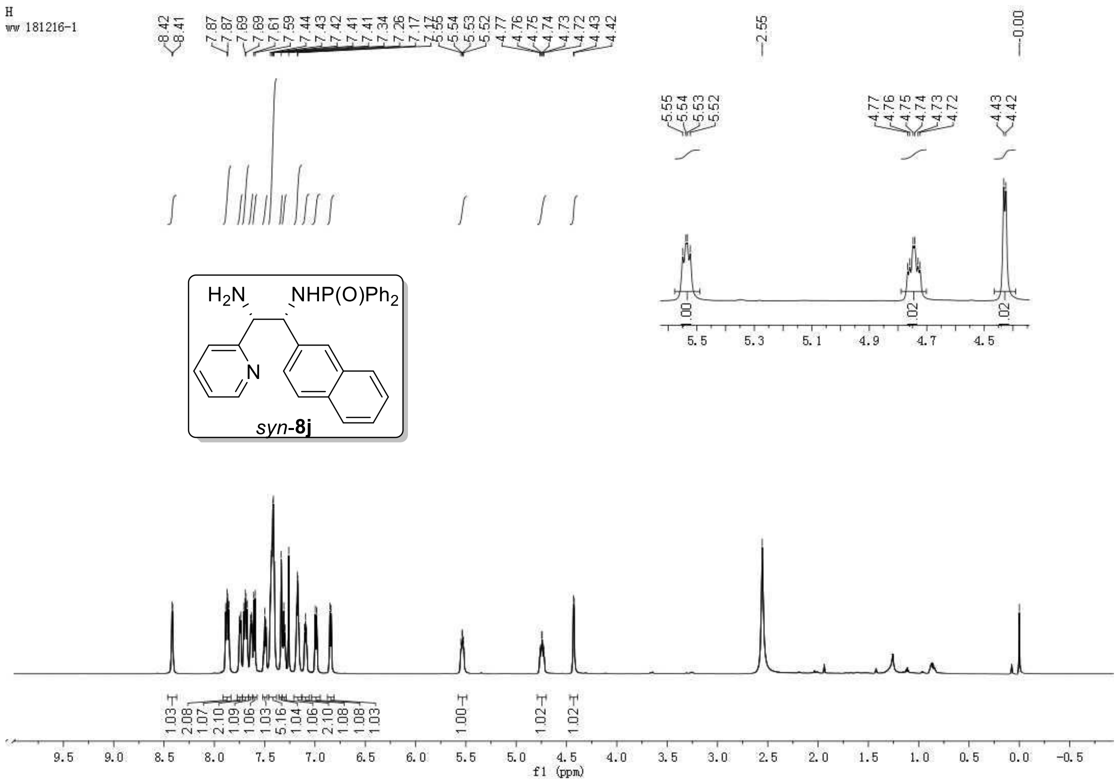

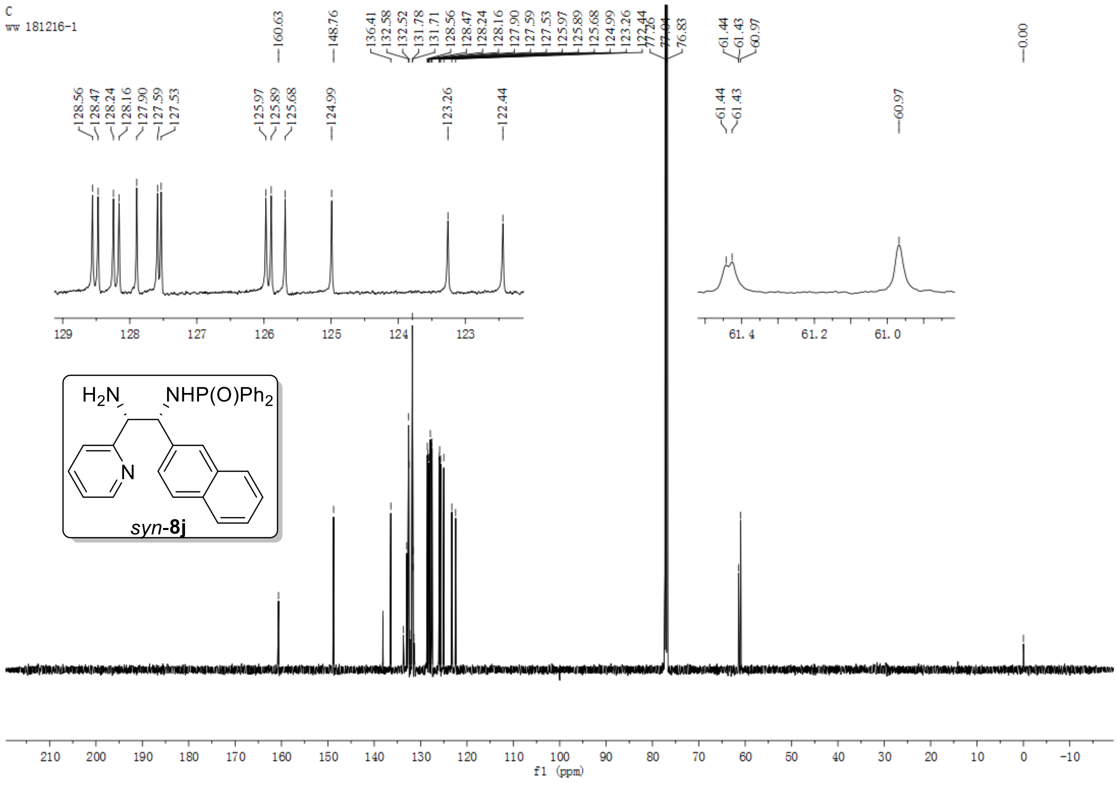

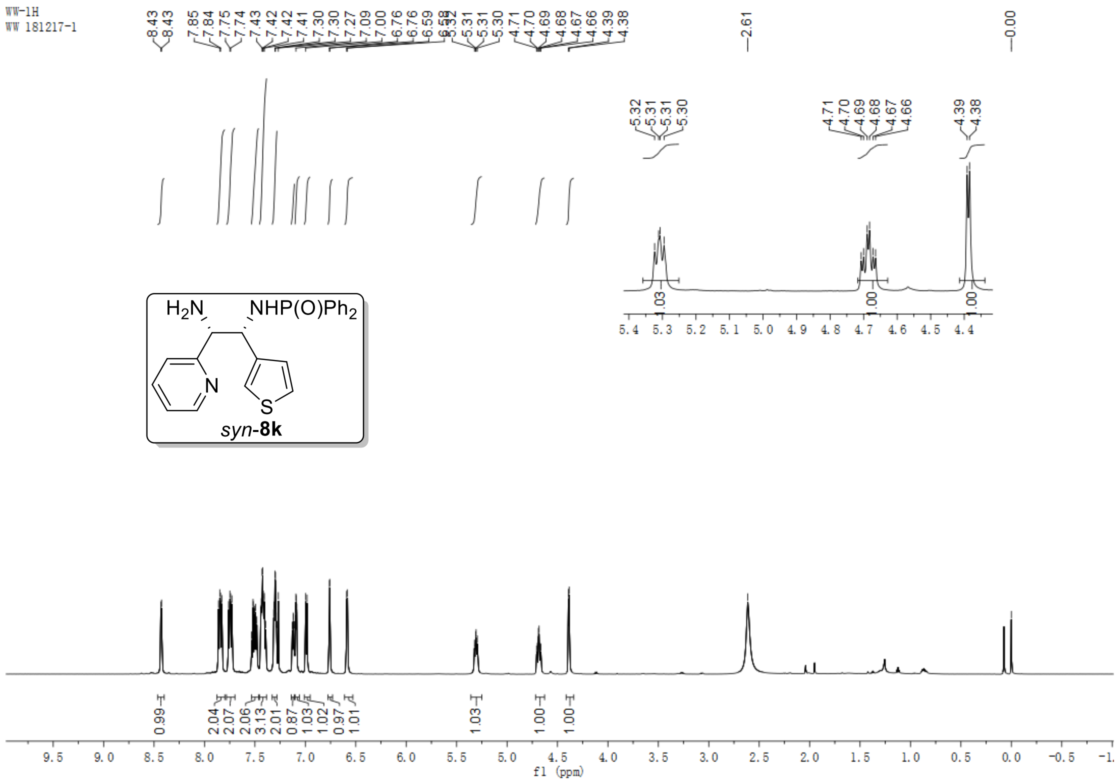
WW-1H
WW 181217-1
syn-8k
f1 (ppm)

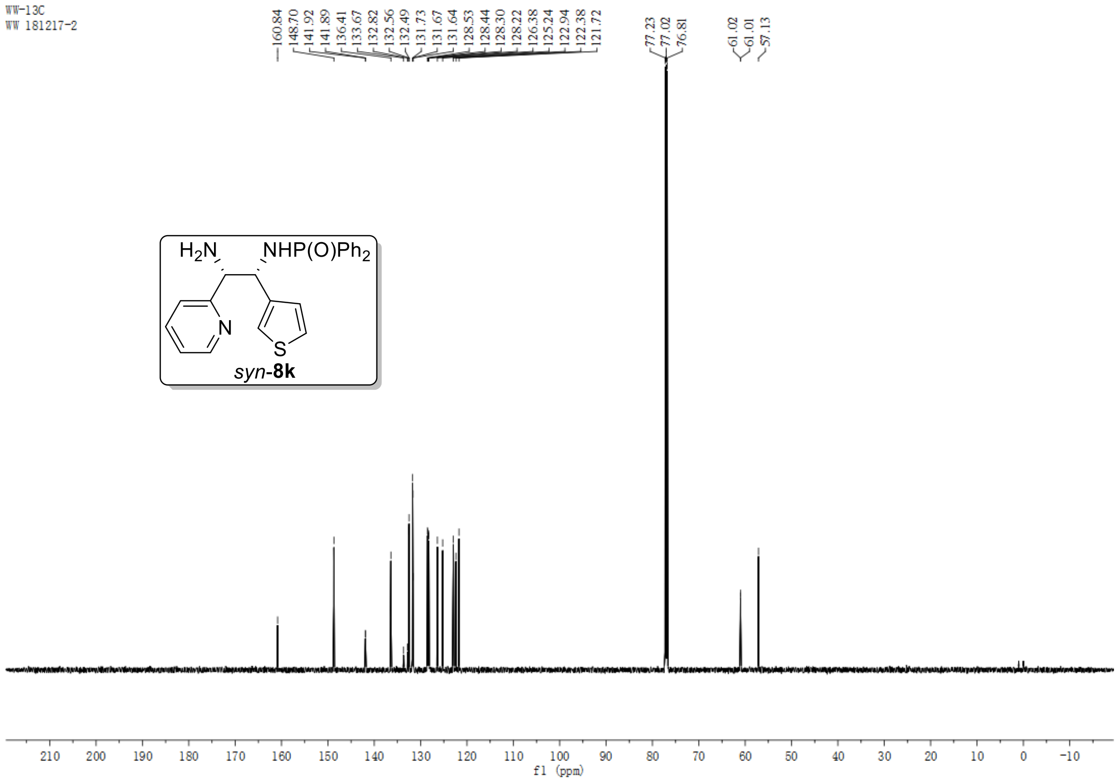
WW-13C
WW 181217-2
syn-8k
f1 (ppm)

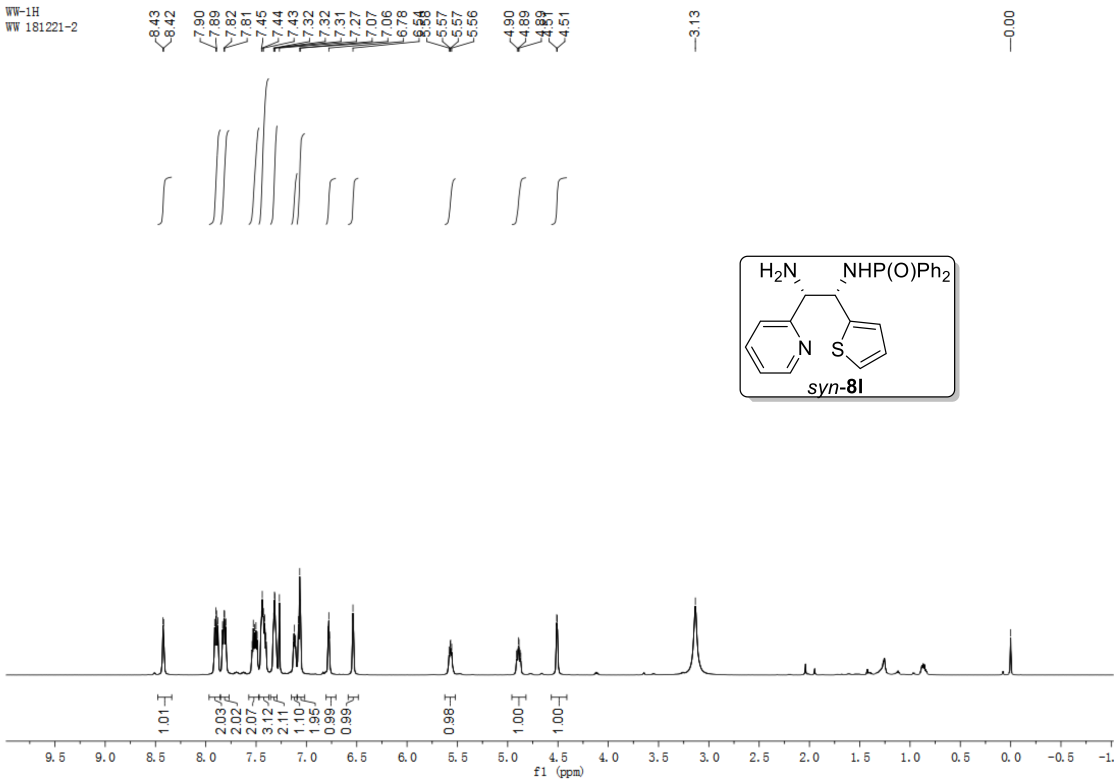
WW-1H
WW 181221-2
H2N NHP(O)Ph2
N S
syn-8l
f1 (ppm)

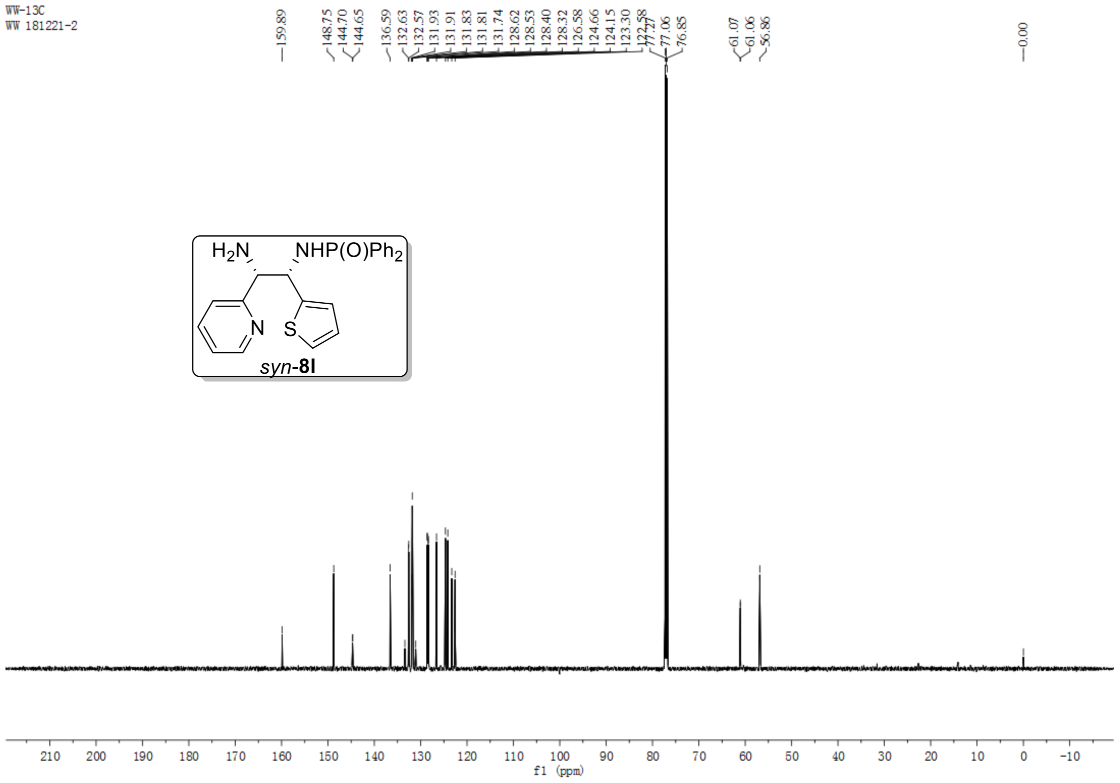
WW-13C
WW 181221-2
H2N NHP(O)Ph2
N S
syn-8l
f1 (ppm)

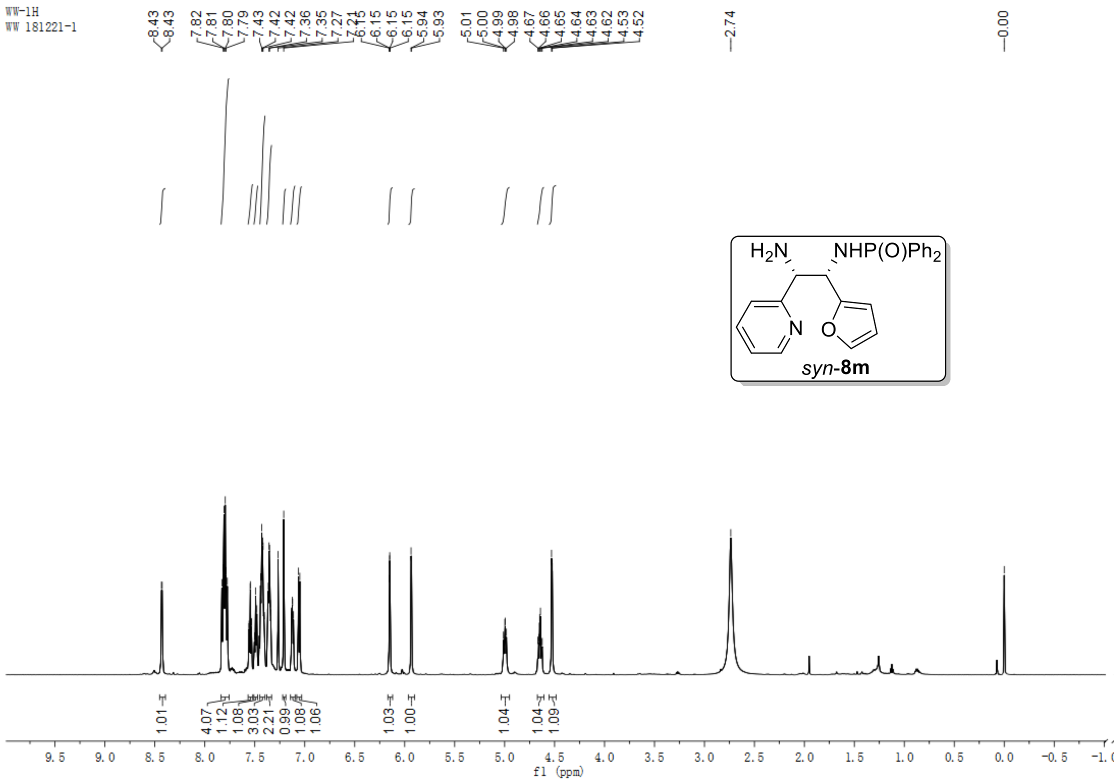
WW-1H
WW 181221-1
8.43 8.43 7.82 7.81 7.80 7.79 7.43 7.42 7.42 7.36 7.35 7.27 7.21 6.15 6.15 6.15 5.94 5.93 5.01 5.00 4.99 4.98 4.67 4.66 4.65 4.64 4.63 4.62 4.53 4.52
2.74
0.00
H2N
NHP(O)Ph2
N
O
syn-8m
1.01 4.07 1.12 1.08 3.03 2.21 0.99 1.08 1.06 1.03 1.00 1.04 1.04 1.09
f1 (ppm)

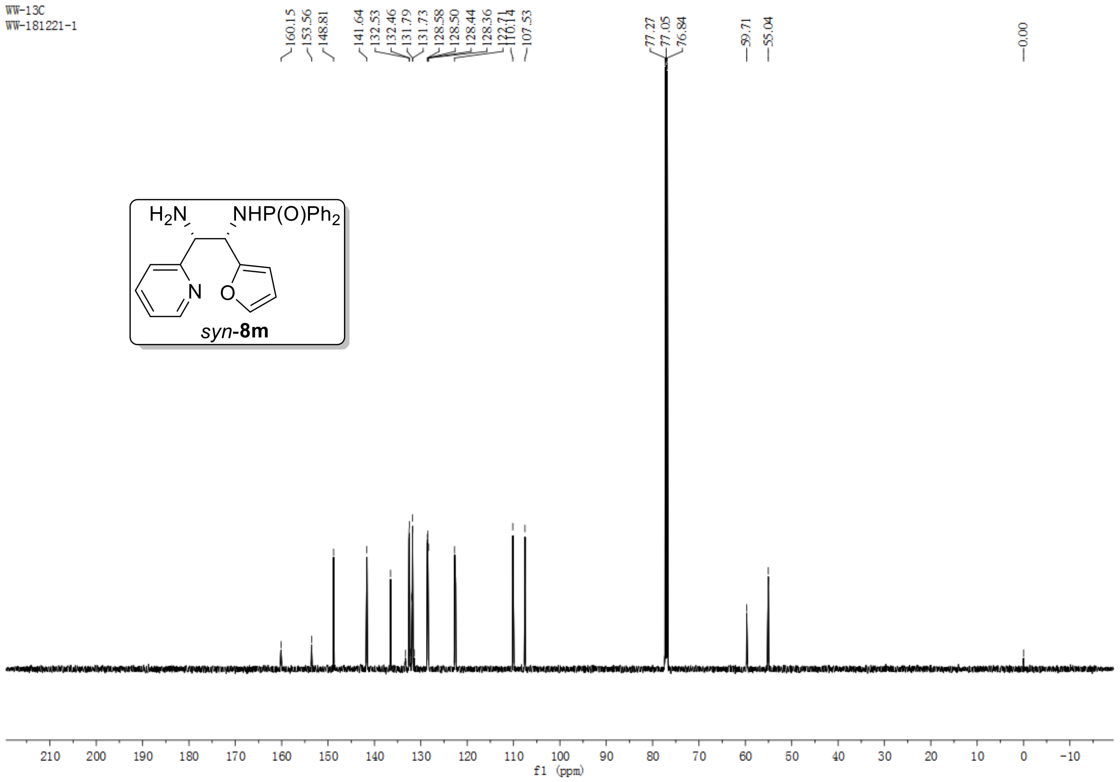
WW-13C
WW-181221-1
160.15 153.56 148.81 141.64 132.53 132.46 131.79 131.73 128.58 128.50 128.44 128.36 122.71 110.14 107.53
77.27 77.05 76.84
59.71 55.04
0.00
H2N
NHP(O)Ph2
N
O
syn-8m
f1 (ppm)

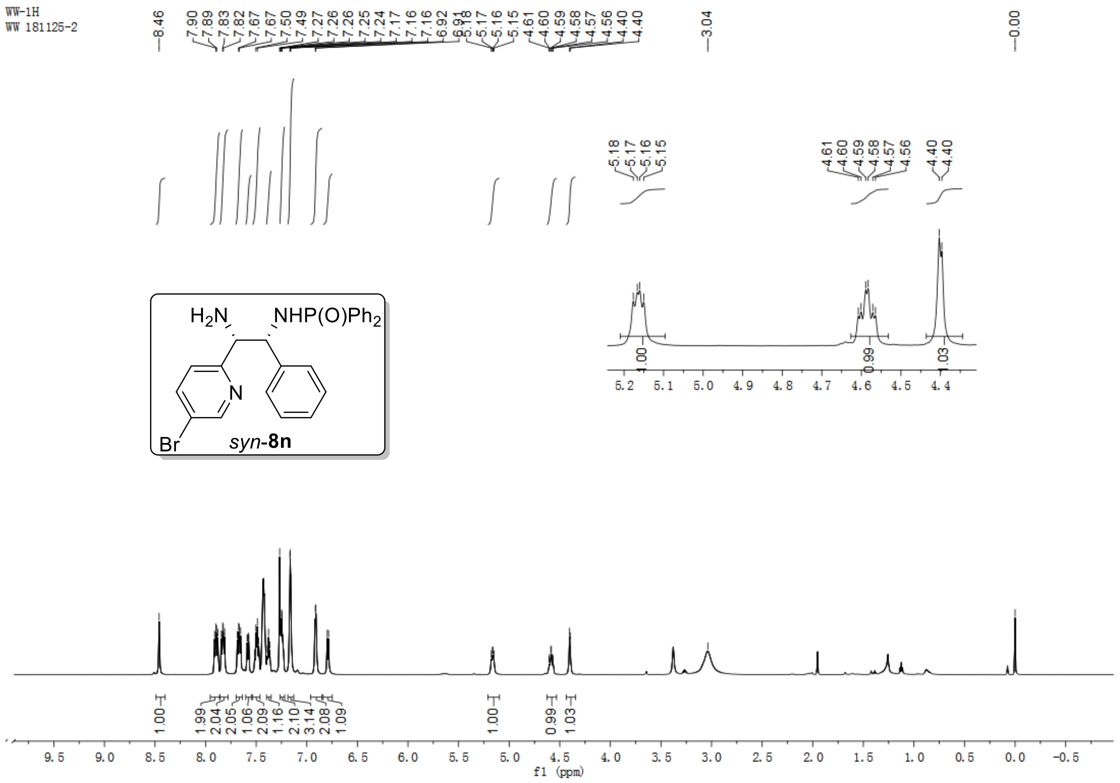
WW-1H
WW 181125-2
8.46
7.90
7.89
7.83
7.82
7.67
7.67
7.50
7.49
7.27
7.26
7.26
7.25
7.24
7.17
7.16
7.16
6.92
6.91
5.18
5.17
5.16
5.15
4.61
4.60
4.59
4.58
4.57
4.56
4.40
4.40
3.04
0.00
H2N
NHP(O)Ph2
N
Br
syn-8n
f1 (ppm)

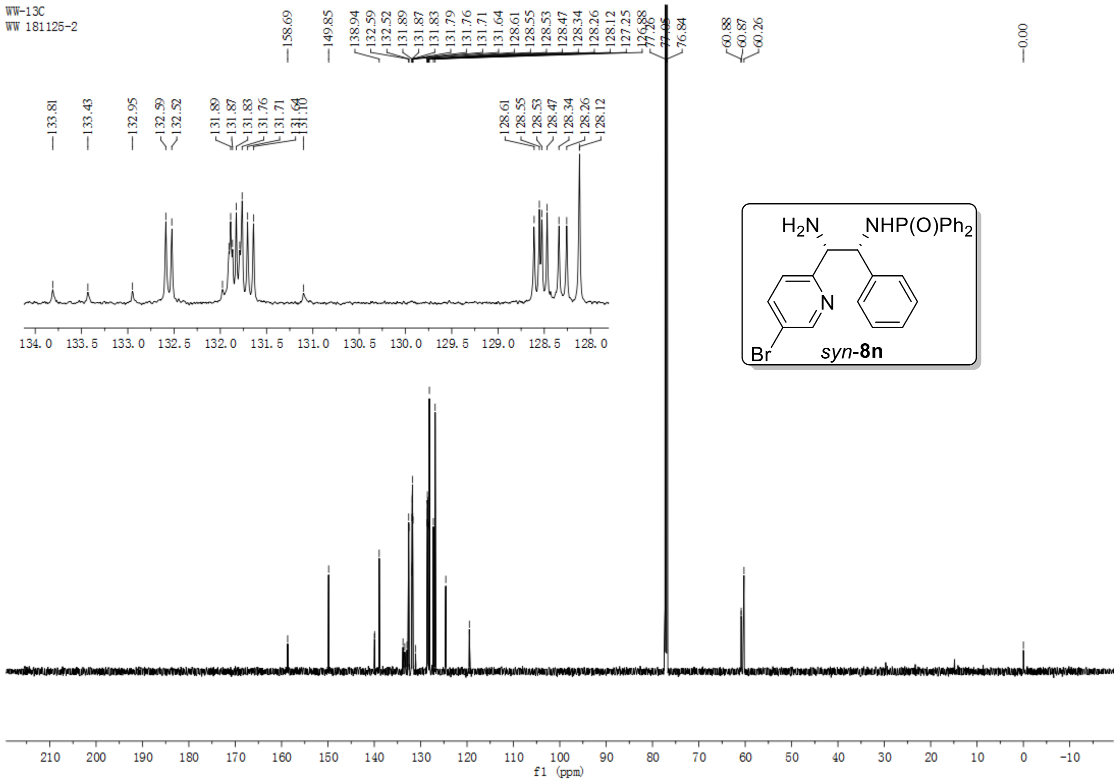
WW-13C
WW 181125-2
158.69
149.85
138.94
132.59
132.52
131.89
131.87
131.83
131.79
131.76
131.71
131.64
128.61
128.55
128.53
128.47
128.34
128.26
128.12
127.25
76.84
60.88
60.87
60.26
0.00
133.81
133.43
132.95
131.10
H2N
NHP(O)Ph2
N
Br
syn-8n
f1 (ppm)

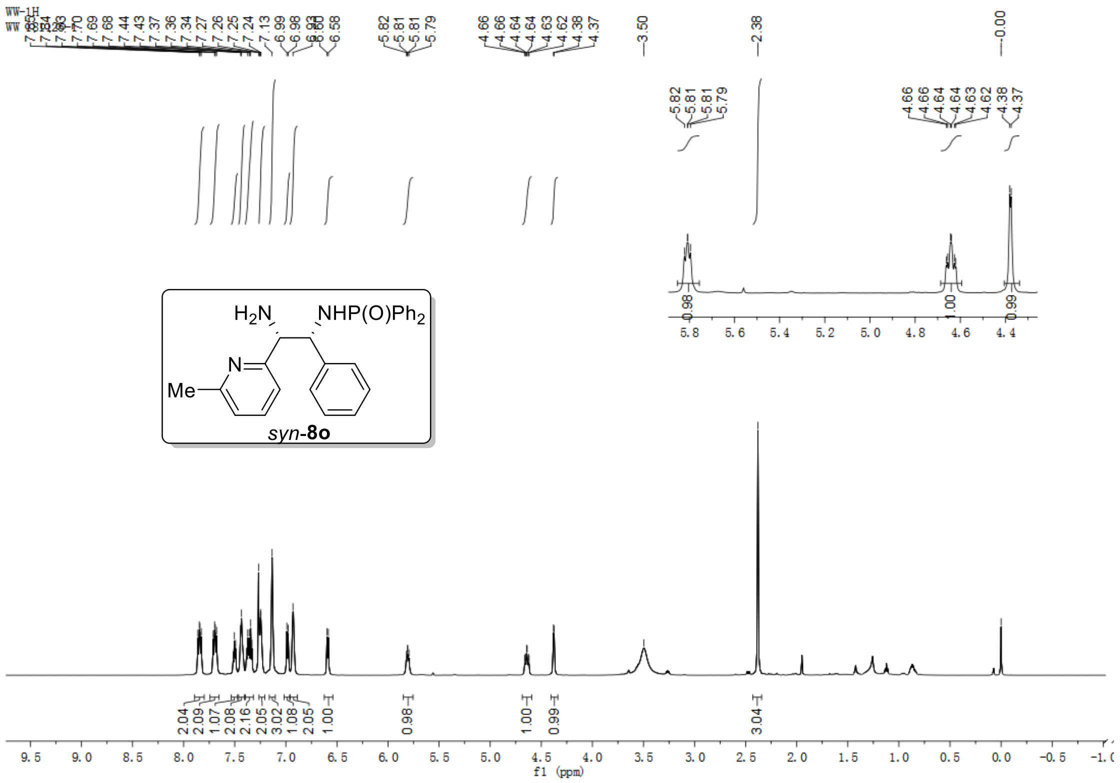

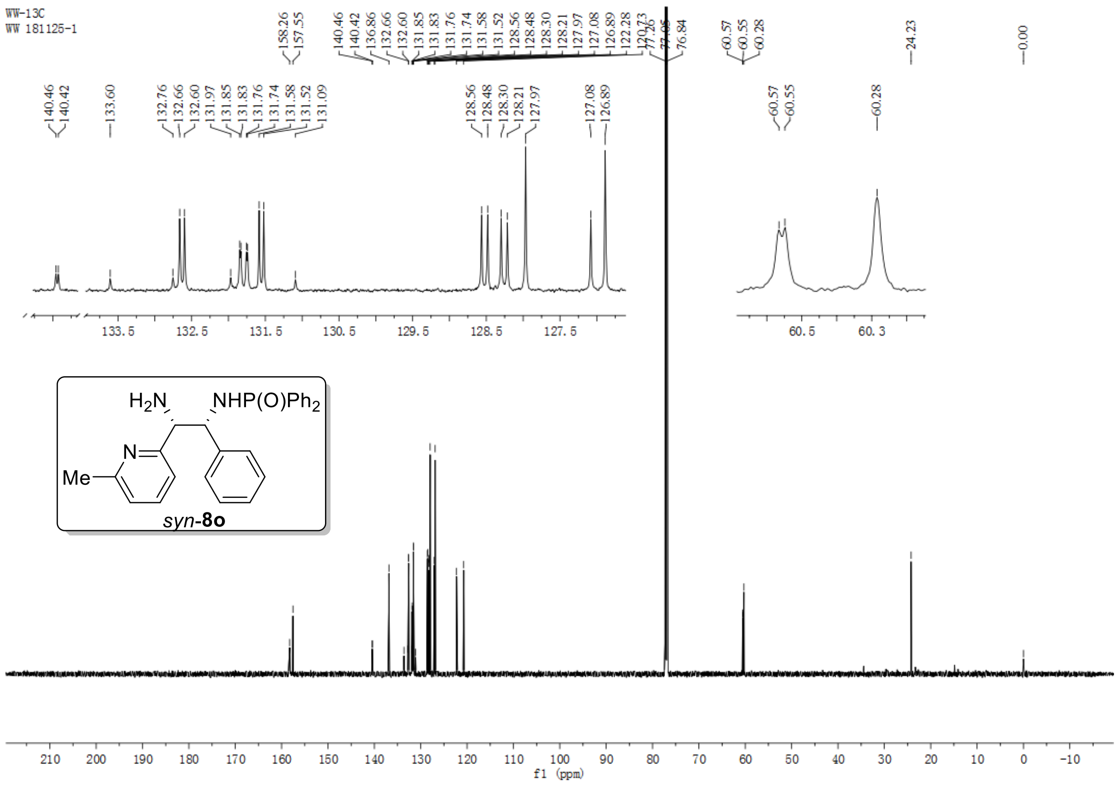

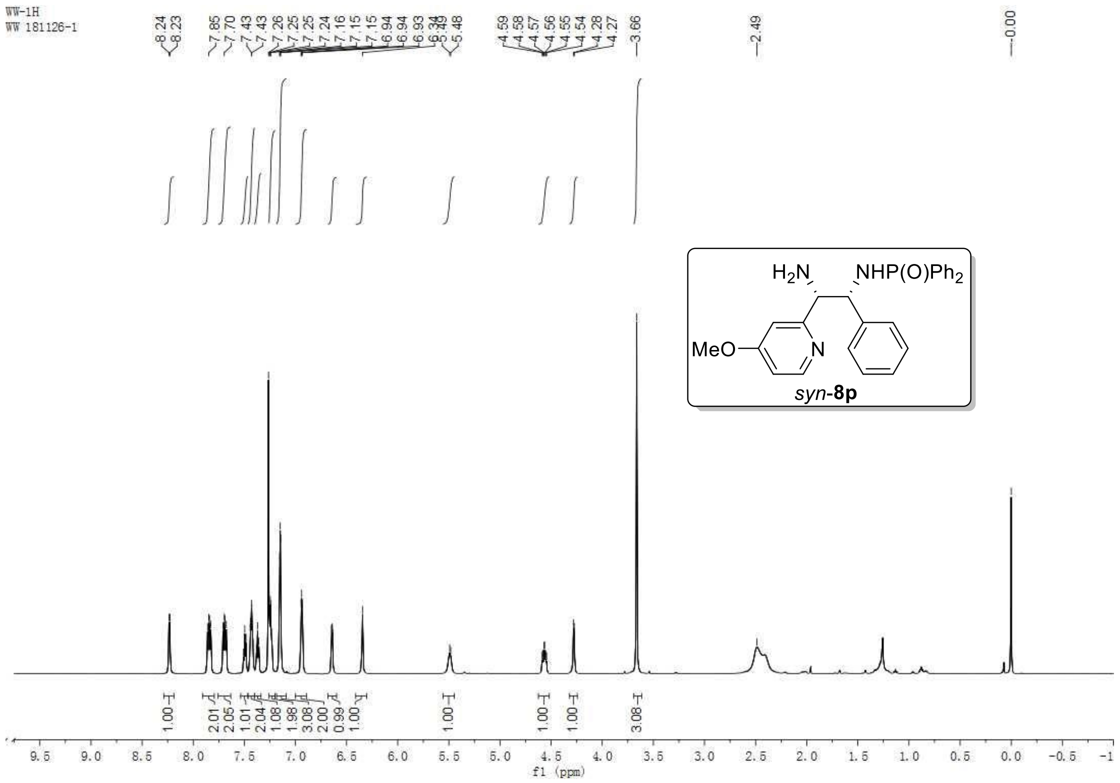
WW-1H
WW 181126-1
8.24
8.23
7.85
7.70
7.43
7.26
7.25
7.25
7.24
7.16
7.15
6.94
6.94
6.93
6.34
5.49
5.48
4.59
4.58
4.57
4.56
4.55
4.54
4.28
4.27
3.66
2.49
0.00
H2N
NHP(O)Ph2
MeO
N
syn-8p
1.00
2.01
2.05
1.01
2.04
1.08
1.98
3.08
2.00
0.99
1.00
1.00
1.00
1.00
3.08
f1 (ppm)

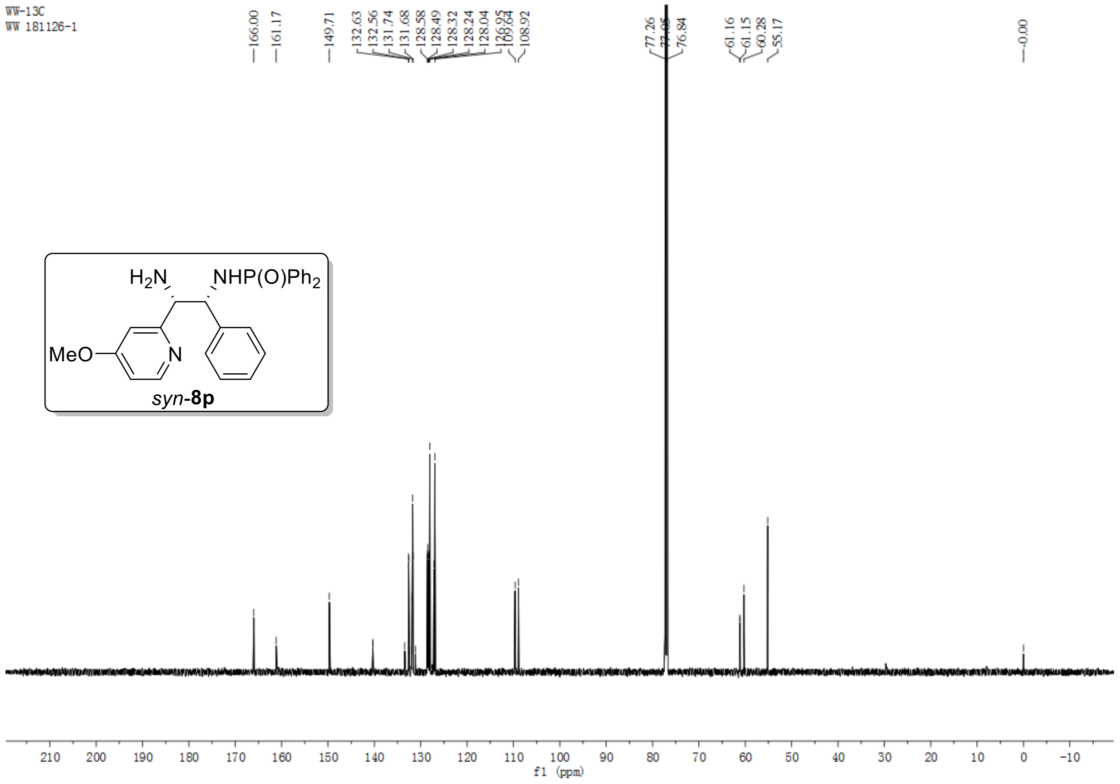
WW-13C
WW 181126-1
166.00
161.17
149.71
132.63
132.56
131.74
131.68
128.58
128.49
128.32
128.24
128.04
109.64
108.92
77.26
76.84
61.16
61.15
60.28
55.17
0.00
H2N
NHP(O)Ph2
MeO
N
syn-8p
f1 (ppm)

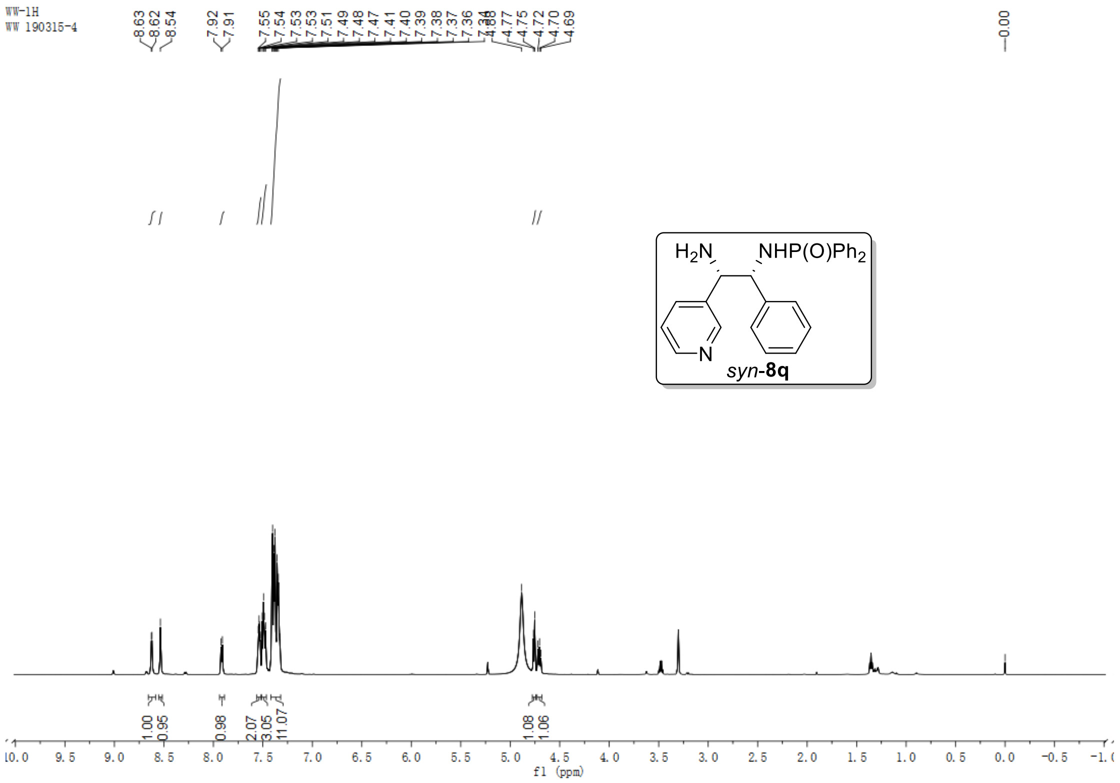
WW-1H
WW 190315-4
H2N
NHP(O)Ph2
N
syn-8q
f1 (ppm)

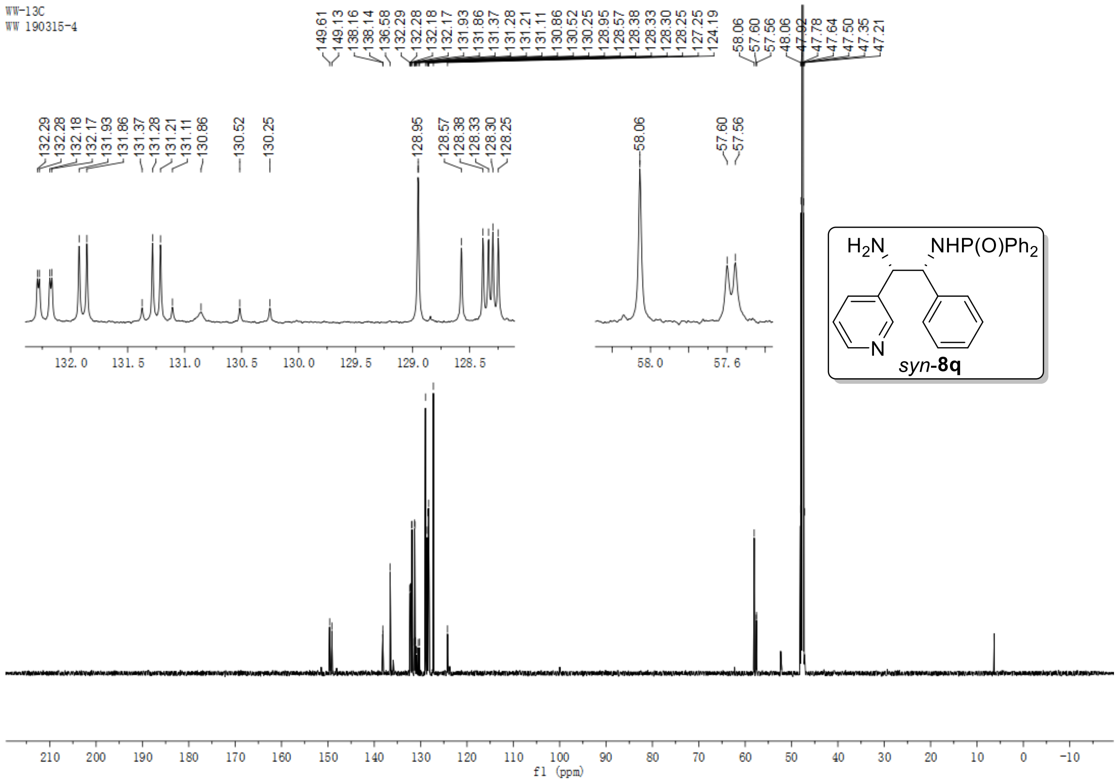
WW-13C
WW 190315-4
H2N
NHP(O)Ph2
N
syn-8q
f1 (ppm)

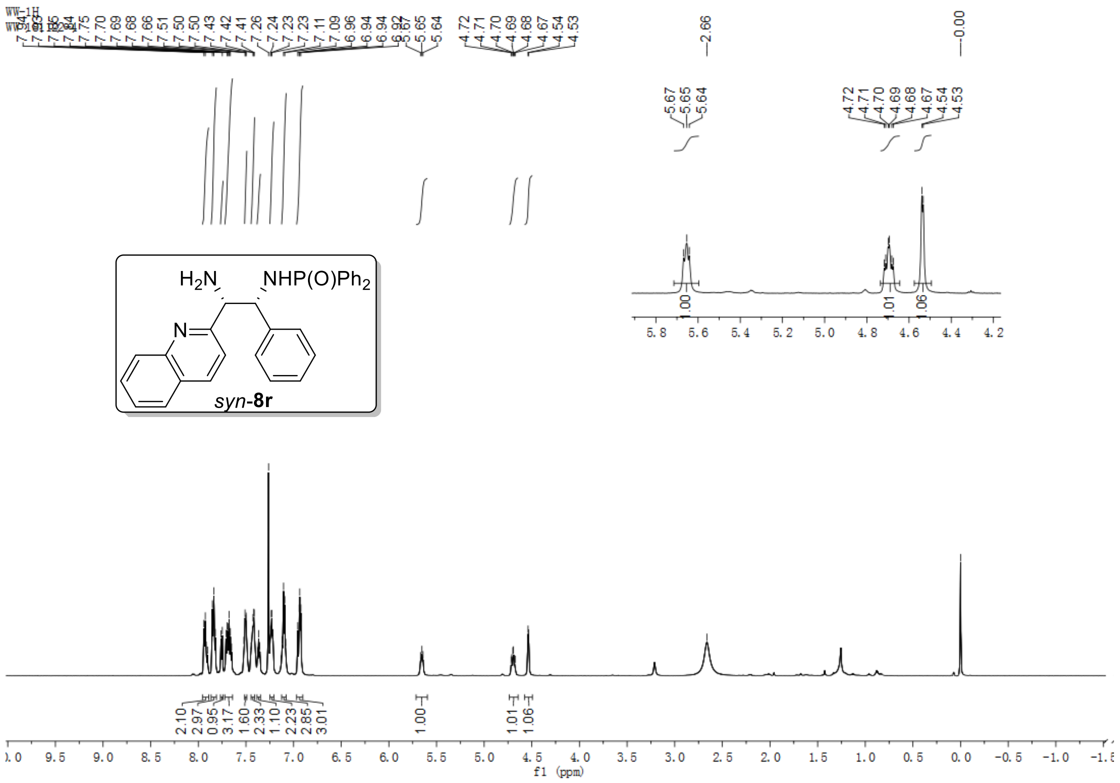

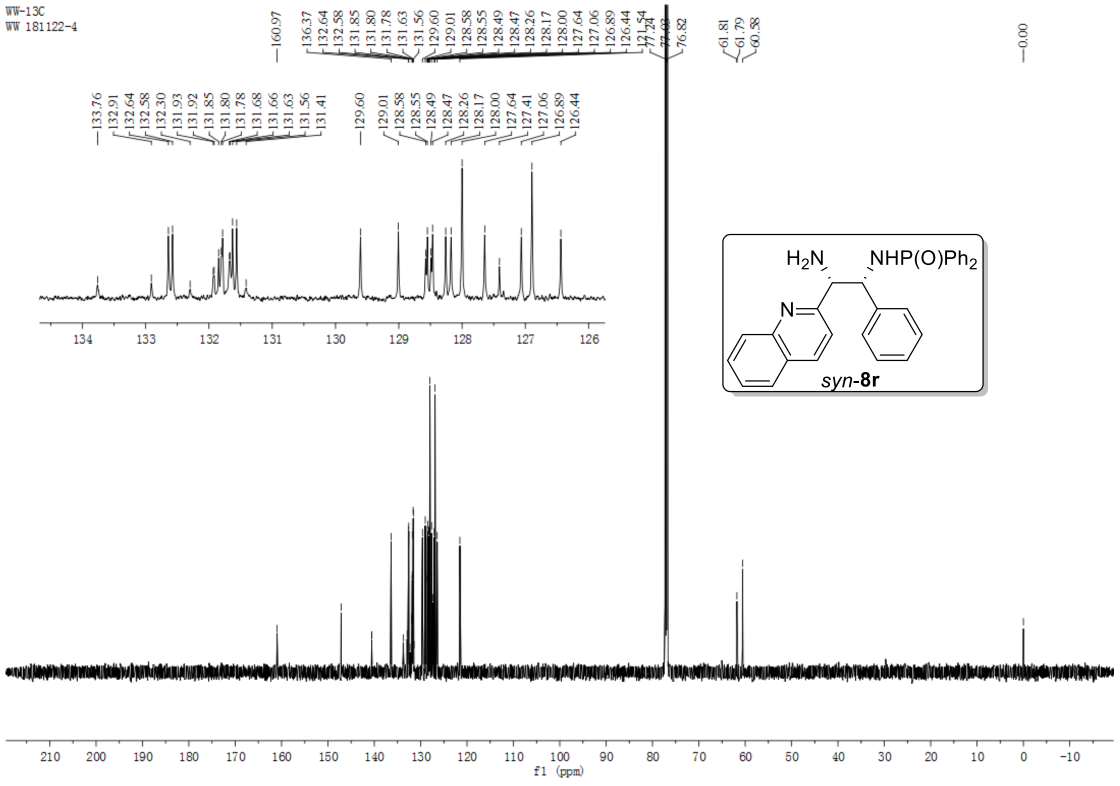

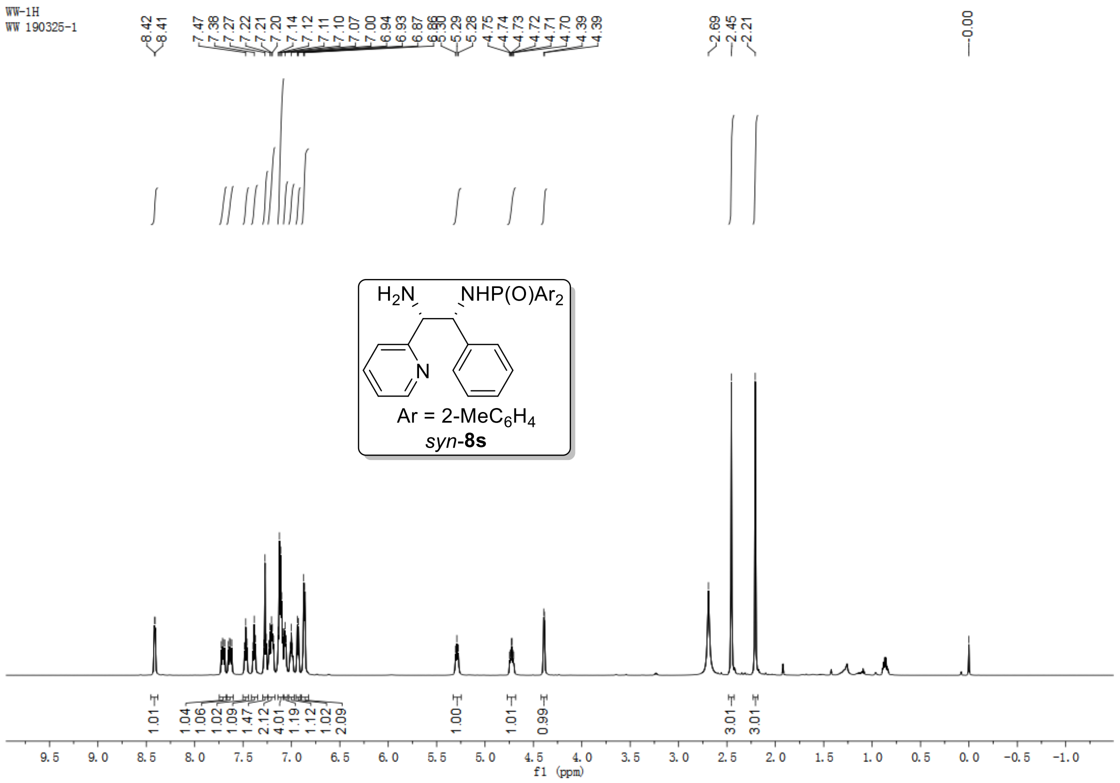
WW-1H
WW 190325-1
H2N
NHP(O)Ar2
N
Ar = 2-MeC6H4
syn-8s
f1 (ppm)

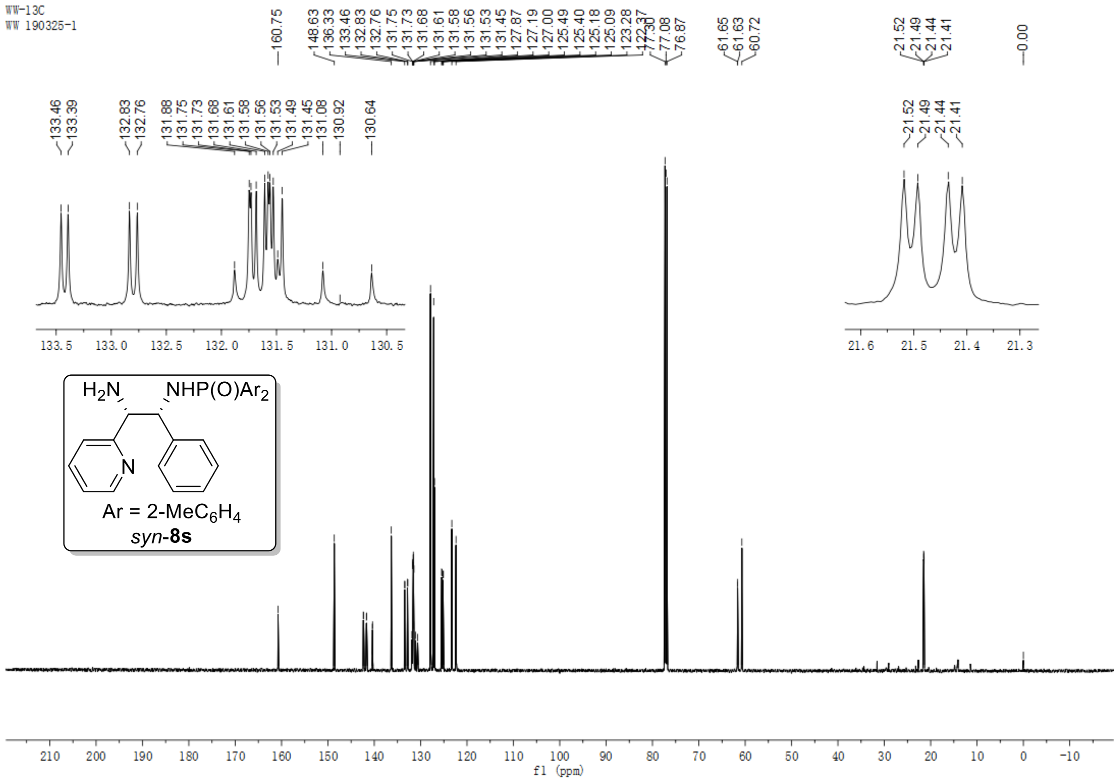
WW-13C
WW 190325-1
H2N
NHP(O)Ar2
N
Ar = 2-MeC6H4
syn-8s
f1 (ppm)

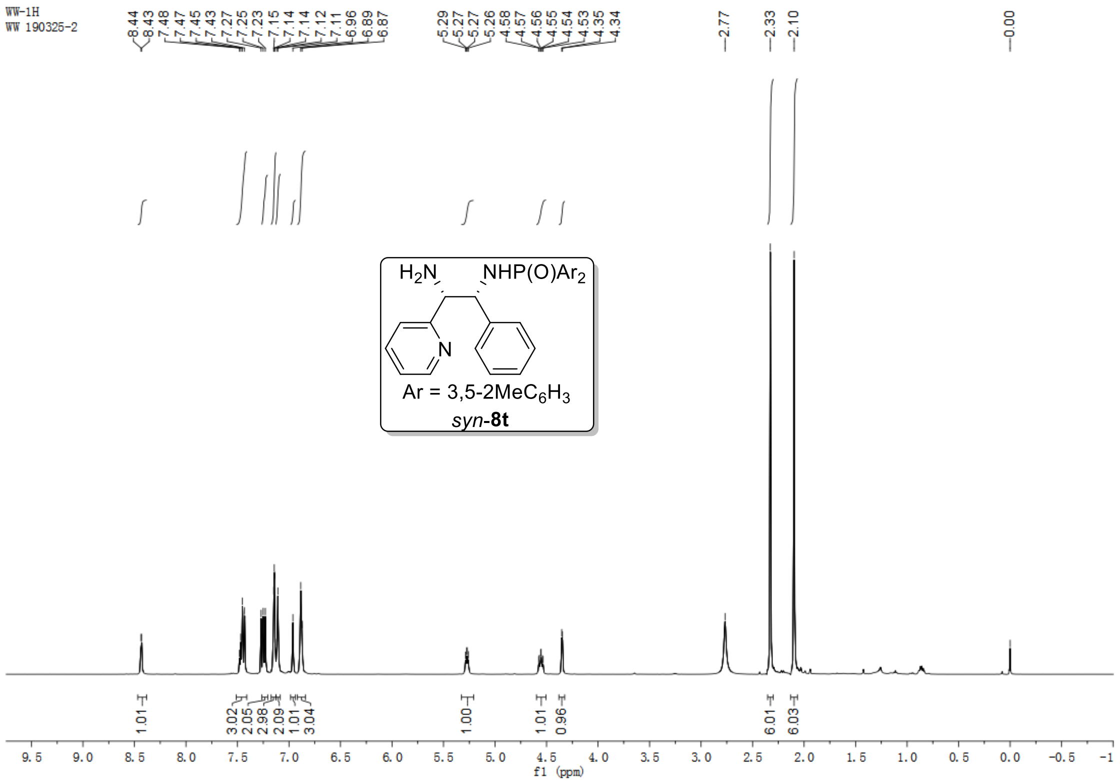
WW-1H
WW 190325-2
H2N NHP(O)Ar2
N
Ar = 3,5-2MeC6H3
syn-8t
f1 (ppm)

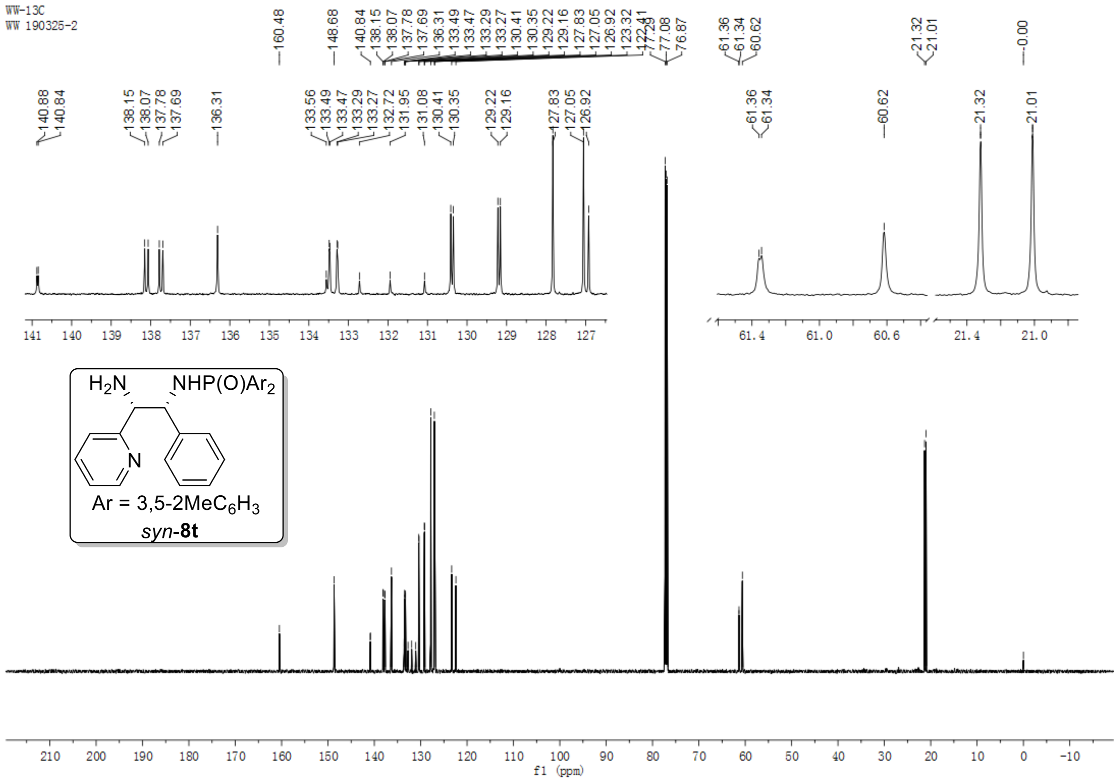
WW-13C
WW 190325-2
H2N NHP(O)Ar2
N
Ar = 3,5-2MeC6H3
syn-8t
f1 (ppm)

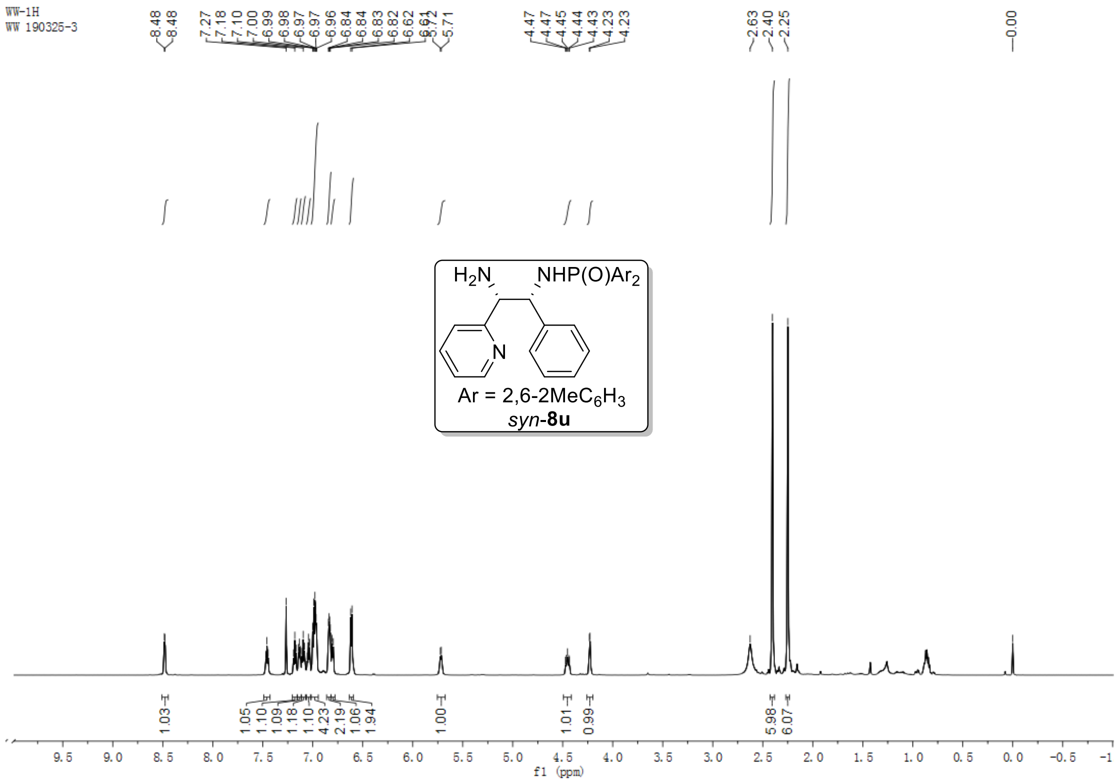
WW-1H
WW 190325-3
8.48 8.48 7.27 7.18 7.10 7.00 6.99 6.98 6.97 6.97 6.96 6.84 6.84 6.83 6.82 6.62 5.72 5.71
4.47 4.47 4.45 4.44 4.43 4.23 4.23
2.63 2.40 2.25
0.00
H2N
NHP(O)Ar2
N
Ar = 2,6-2MeC6H3
syn-8u
1.03 1.05 1.10 1.09 1.18 1.10 4.23 2.19 1.06 1.94 1.00 1.01 0.99 5.98 6.07
f1 (ppm)

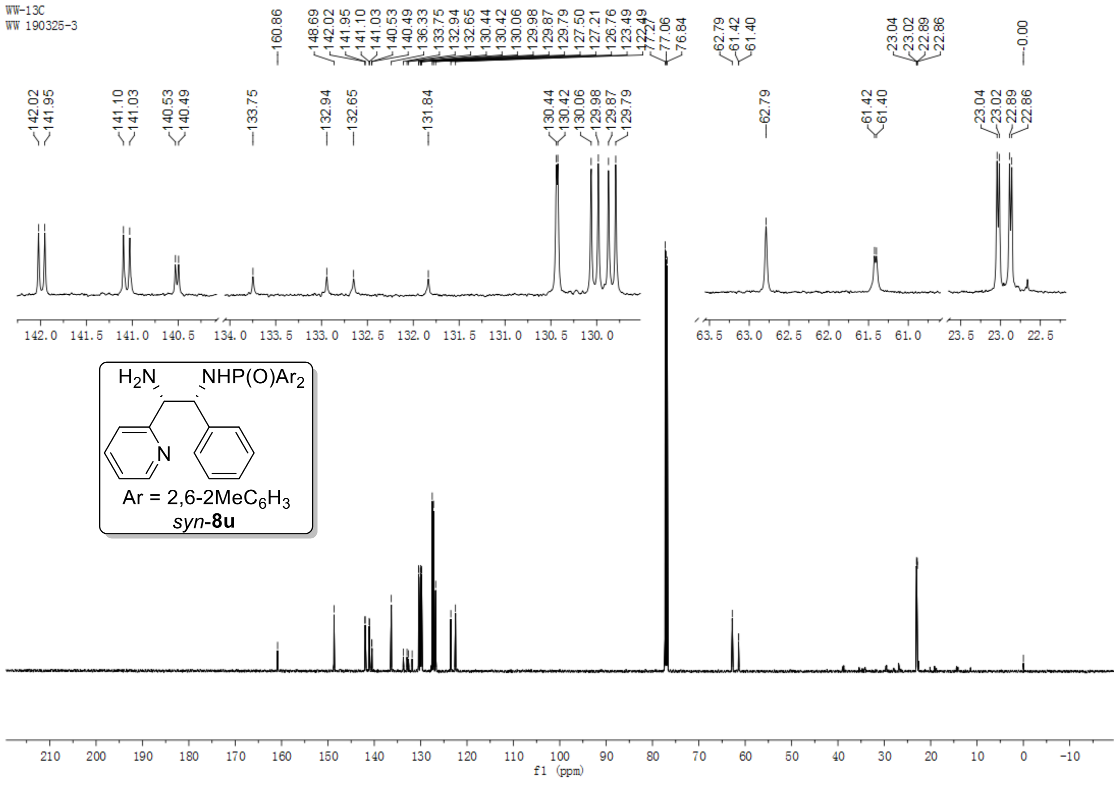
WW-13C
WW 190325-3
160.86
148.69 142.02 141.95 141.10 141.03 140.53 140.49 136.33 133.75 132.94 132.65 130.44 130.42 130.06 129.98 129.87 129.79 127.50 127.21 126.76 123.49 122.49 77.27 77.06 76.84
62.79 61.42 61.40
23.04 23.02 22.89 22.86
0.00
131.84
H2N
NHP(O)Ar2
N
Ar = 2,6-2MeC6H3
syn-8u
f1 (ppm)

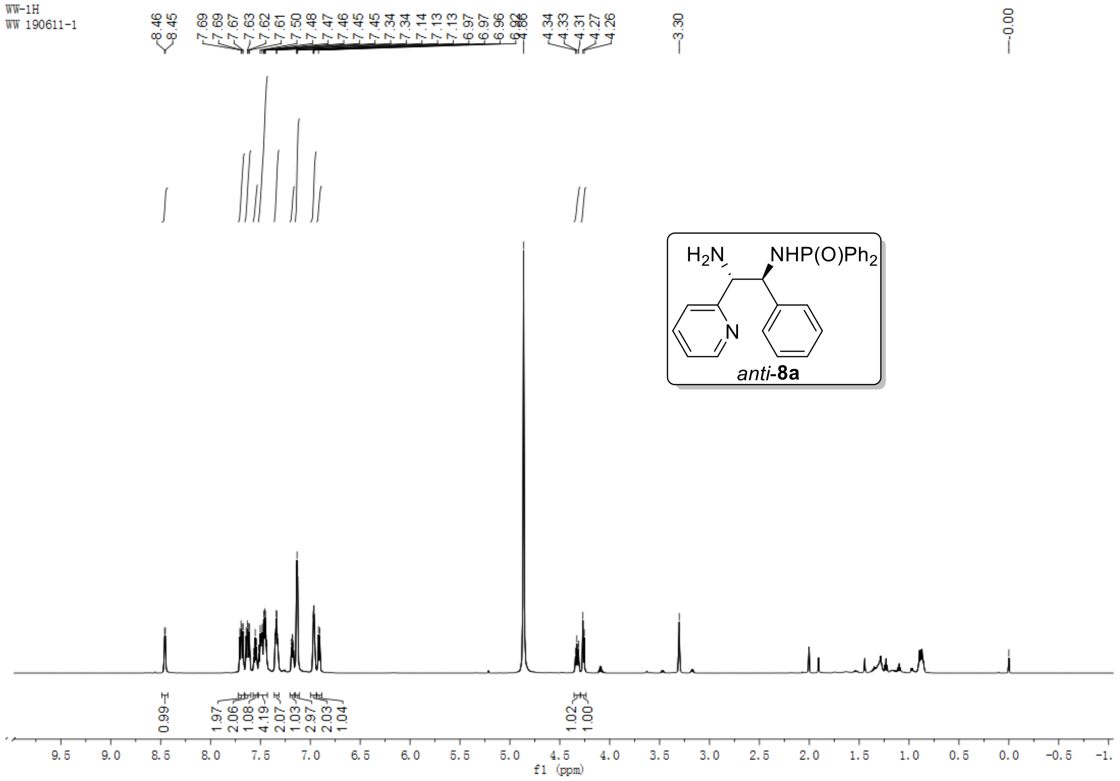
WW-1H
WW 190611-1
H2N
NHP(O)Ph2
N
anti-8a
f1 (ppm)

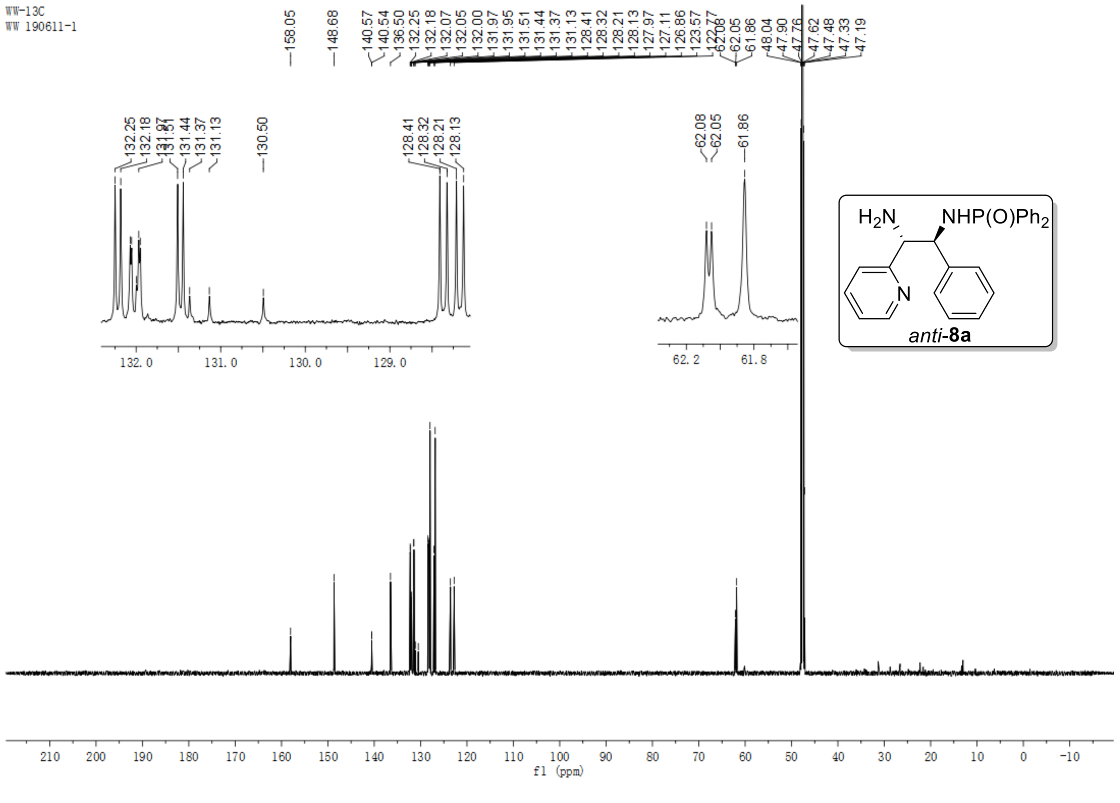
WW-13C
WW 190611-1
H2N
NHP(O)Ph2
N
anti-8a
f1 (ppm)

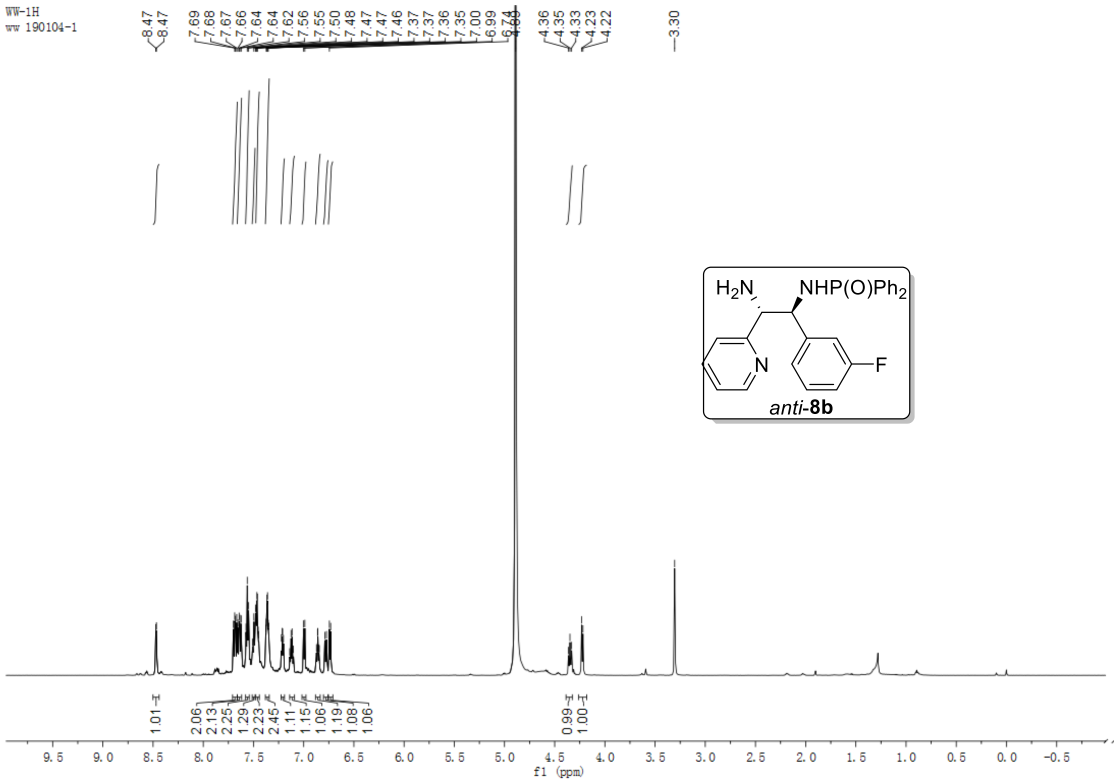
WW-1H
ww 190104-1
H2N
NHP(O)Ph2
N
F
anti-8b
f1 (ppm)

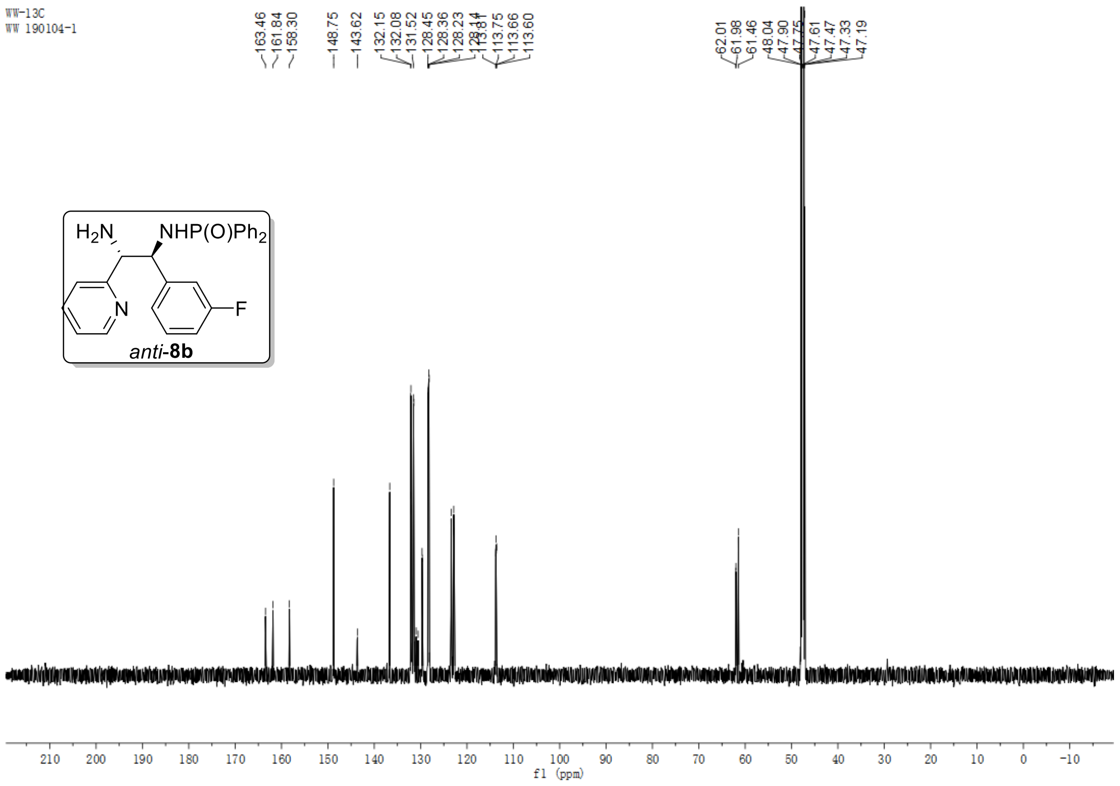
WW-13C
WW 190104-1
H2N
NHP(O)Ph2
N
F
anti-8b
f1 (ppm)

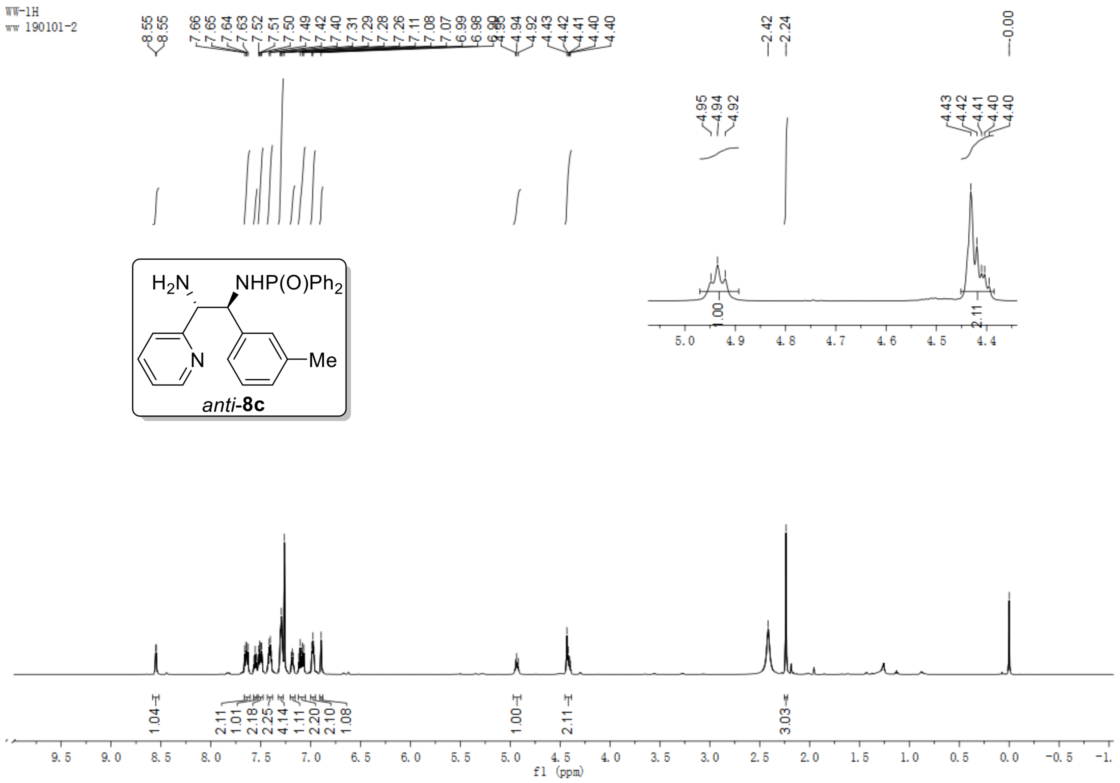
WW-1H
ww 190101-2
8.55
8.55
7.66
7.65
7.64
7.63
7.52
7.51
7.50
7.49
7.42
7.40
7.40
7.31
7.29
7.28
7.26
7.11
7.08
7.07
6.99
6.98
4.95
4.94
4.92
4.43
4.42
4.41
4.40
4.40
2.42
2.24
0.00
H2N
NHP(O)Ph2
N
Me
anti-8c
1.00
2.11
1.04
2.11
1.01
2.18
2.25
4.14
1.11
2.20
2.10
1.08
3.03
f1 (ppm)

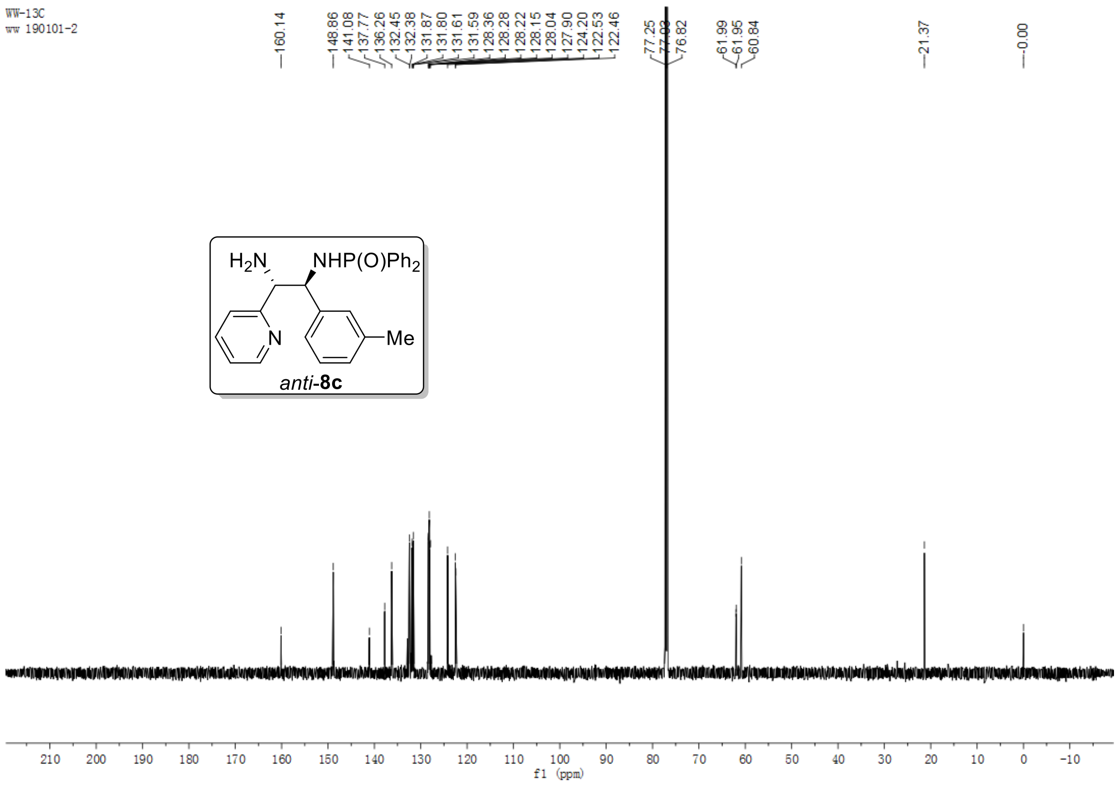
WW-13C
ww 190101-2
160.14
148.86
141.08
137.77
136.26
132.45
132.38
131.87
131.80
131.61
131.59
128.36
128.28
128.22
128.15
128.04
127.90
124.20
122.53
122.46
77.25
76.82
61.99
61.95
60.84
21.37
0.00
H2N
NHP(O)Ph2
N
Me
anti-8c
f1 (ppm)

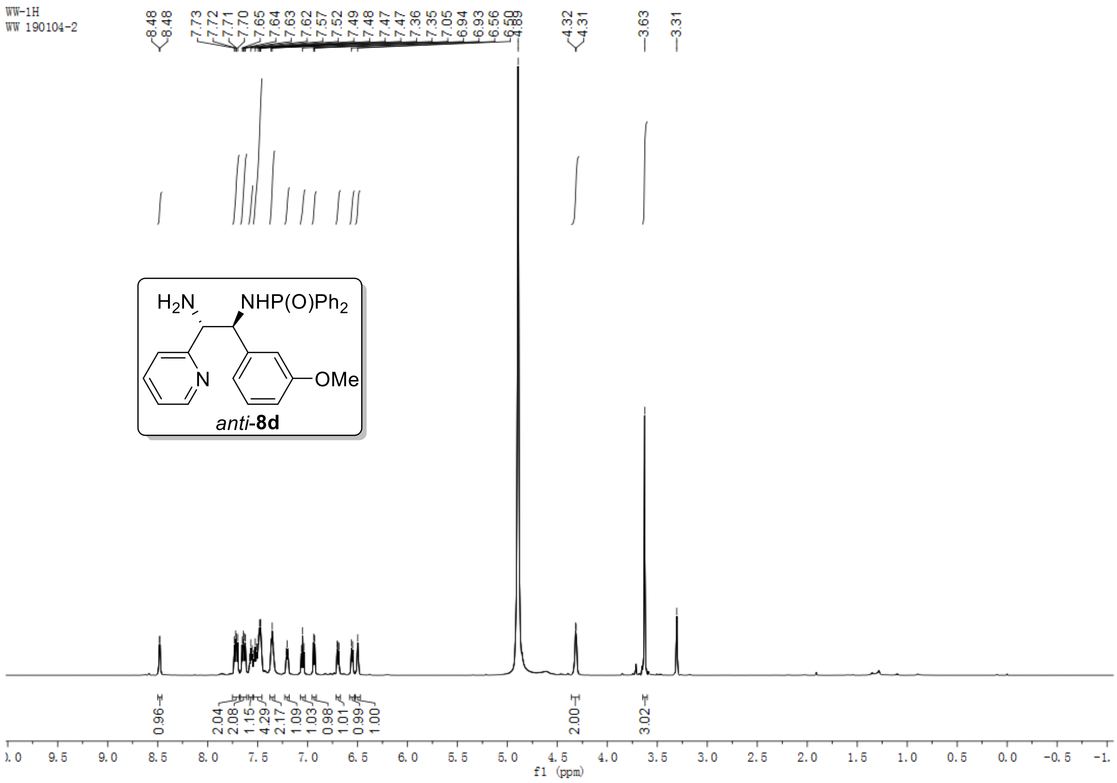
WW-1H
WW 190104-2
H2N
NHP(O)Ph2
N
OMe
anti-8d
f1 (ppm)

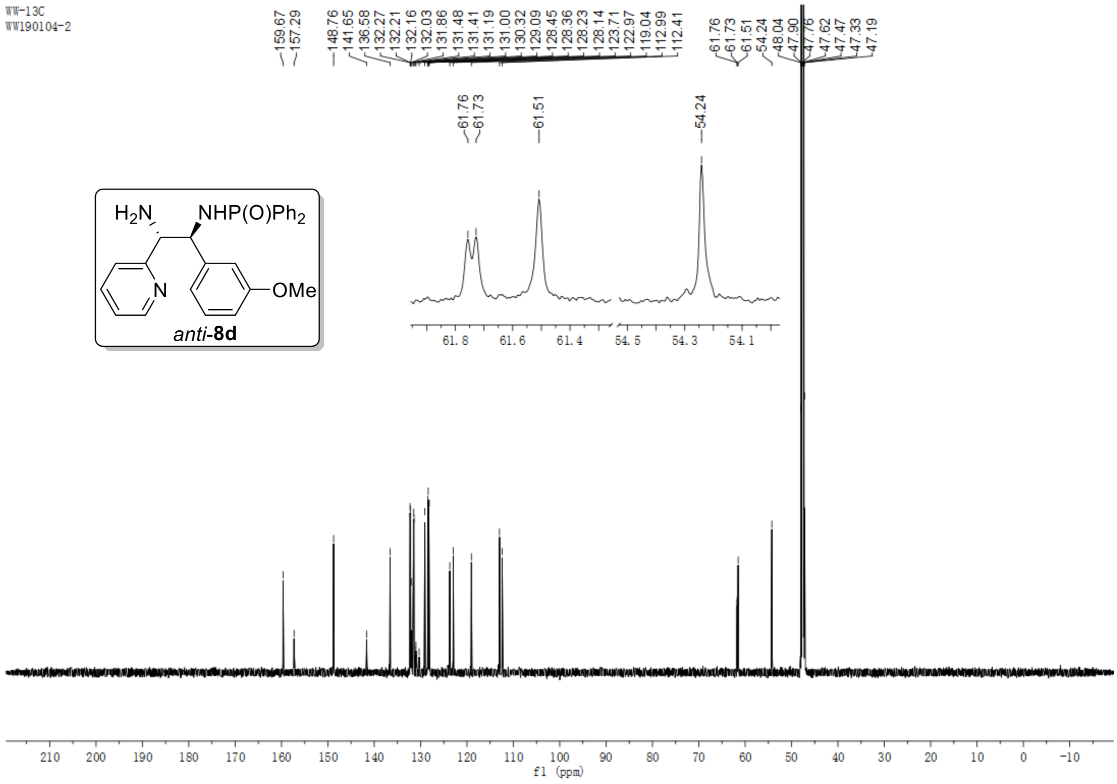
WW-13C
WW190104-2
H2N
NHP(O)Ph2
N
OMe
anti-8d
f1 (ppm)

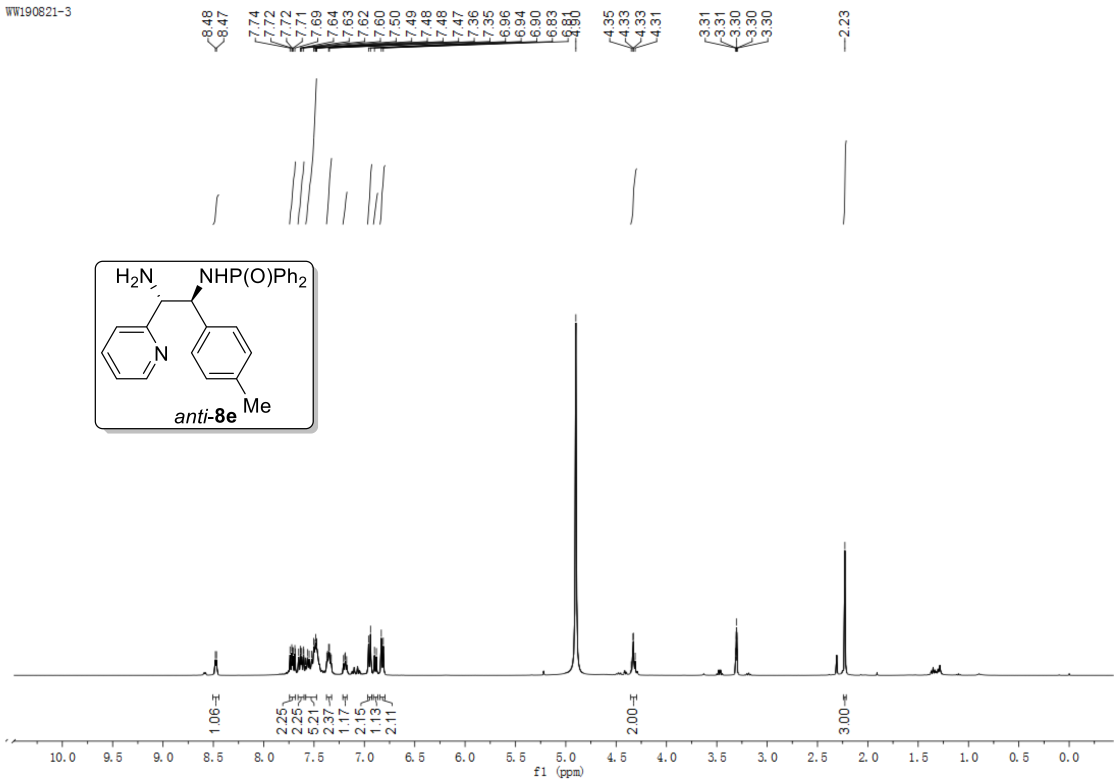

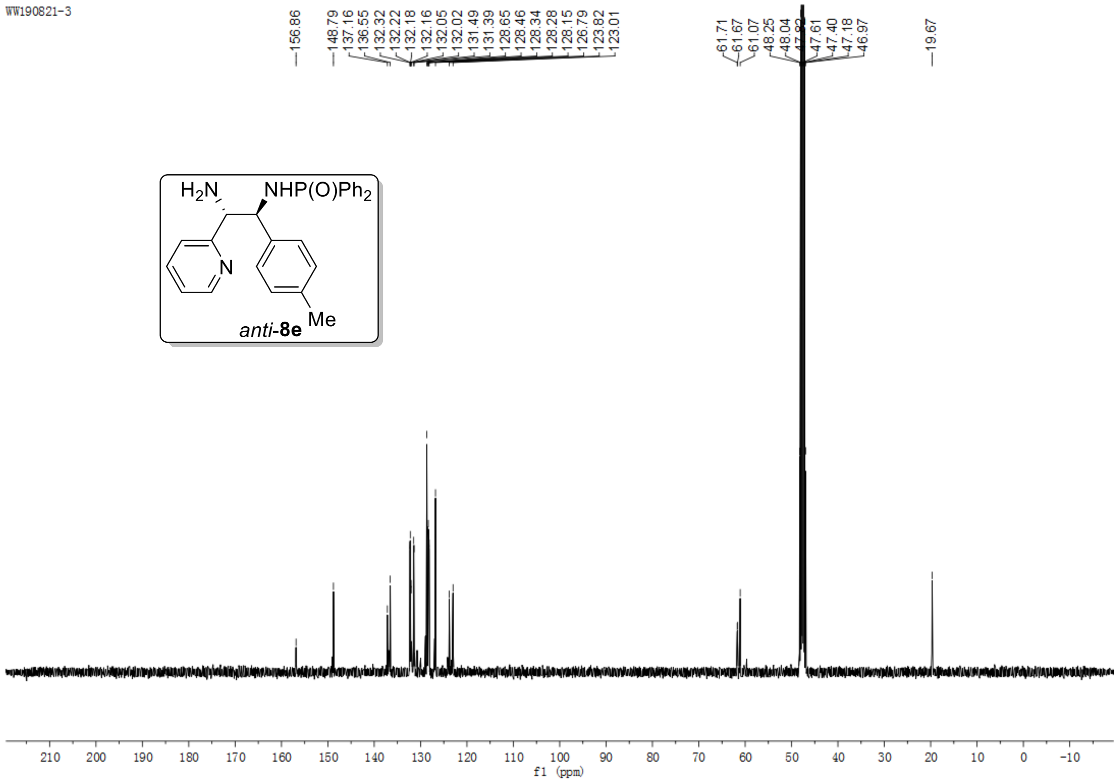

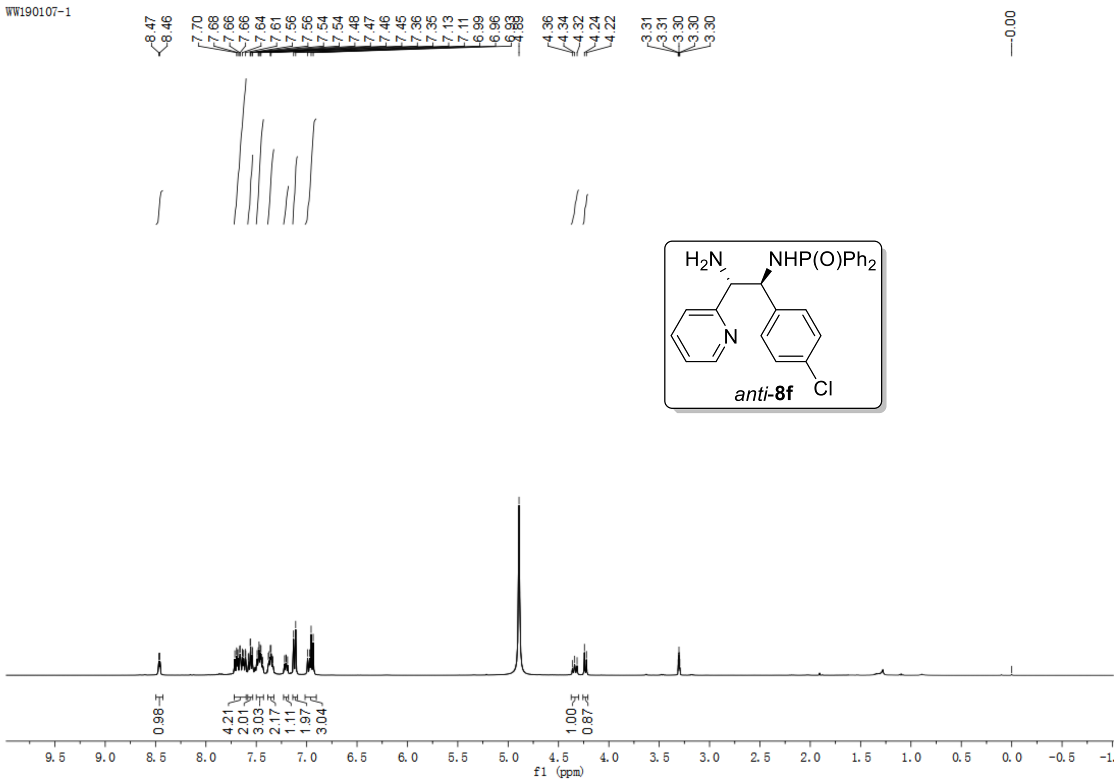

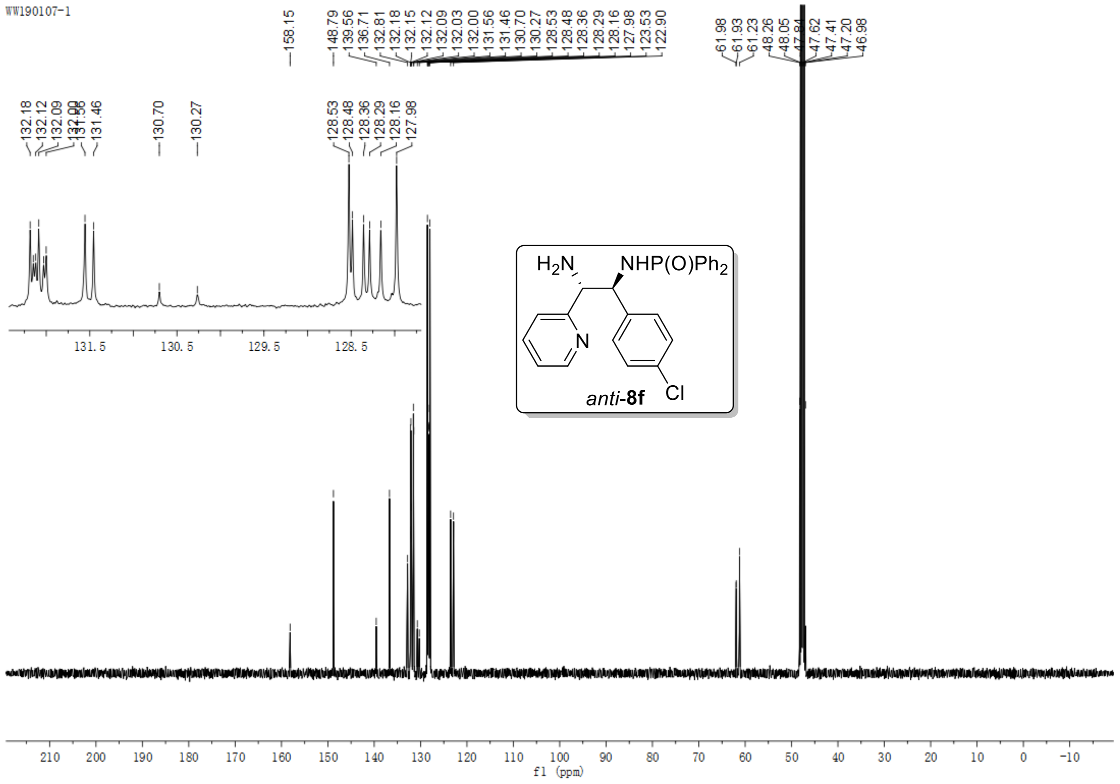

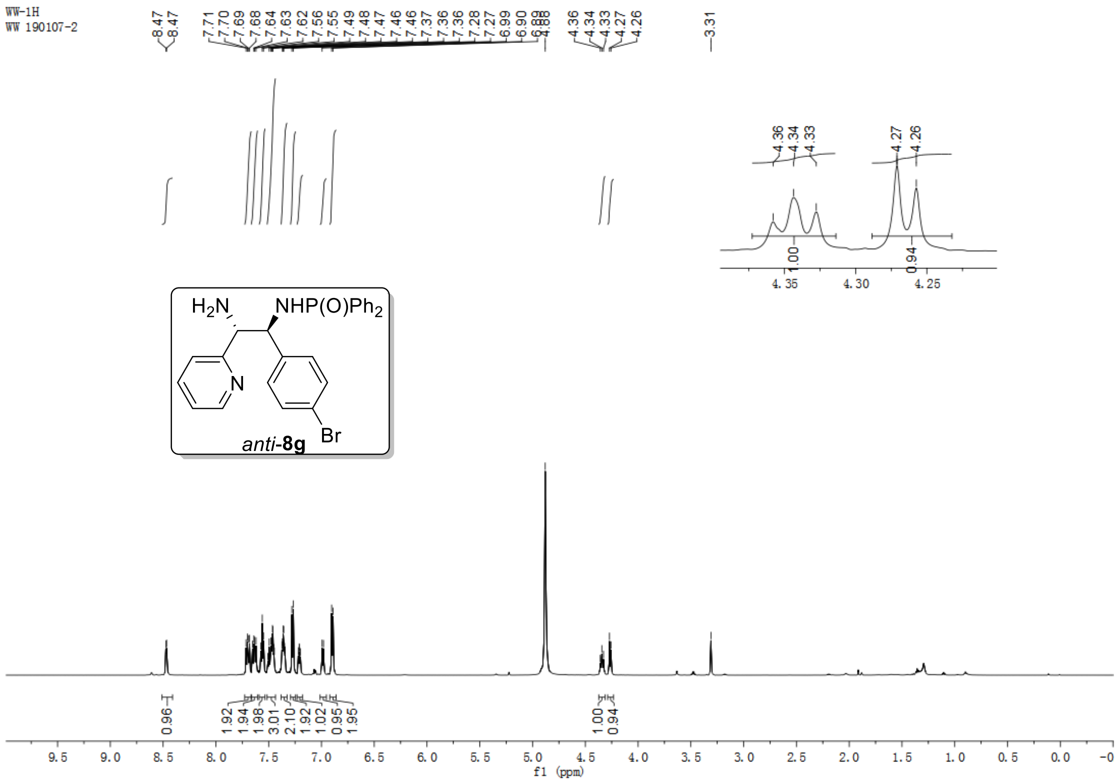
WW-1H
WW 190107-2
H2N
NHP(O)Ph2
N
Br
anti-8g
f1 (ppm)

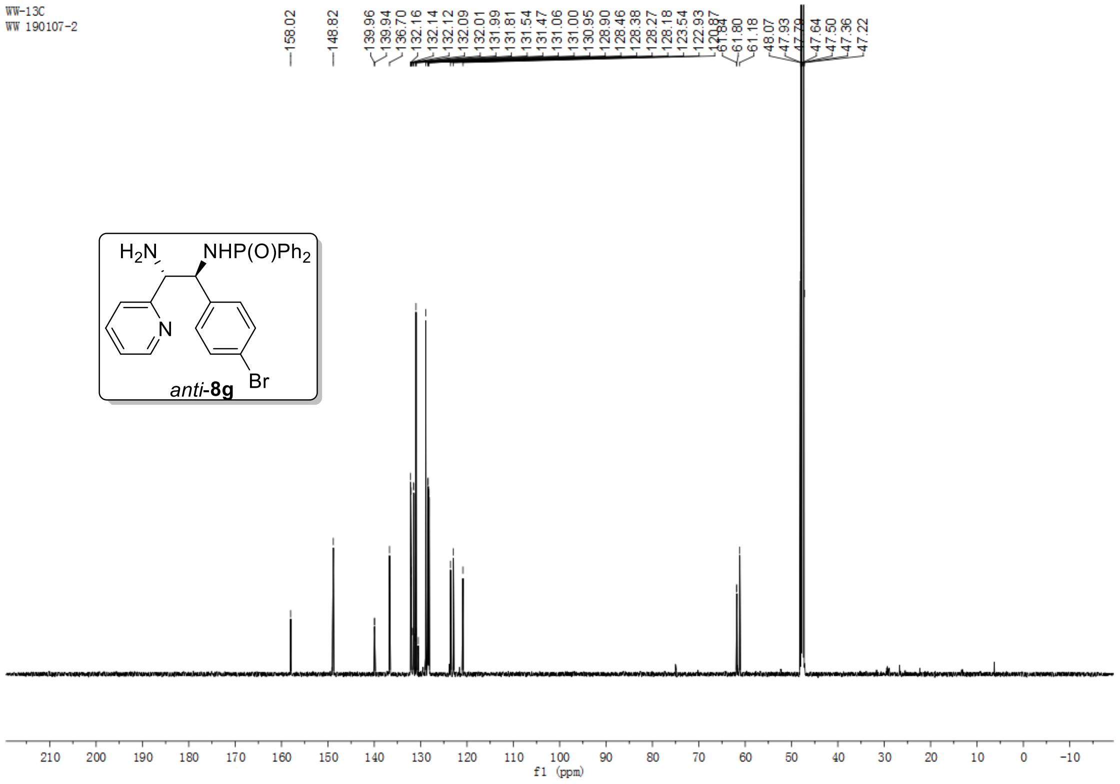
WW-13C
WW 190107-2
H2N
NHP(O)Ph2
N
Br
anti-8g
f1 (ppm)

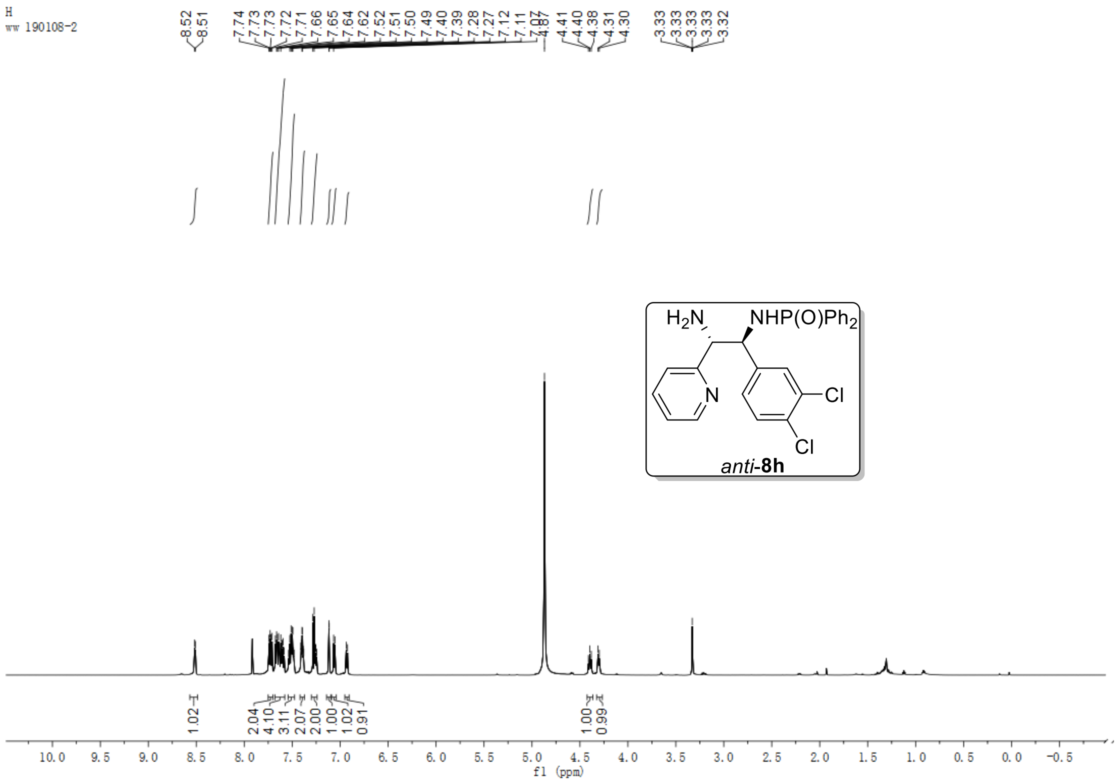

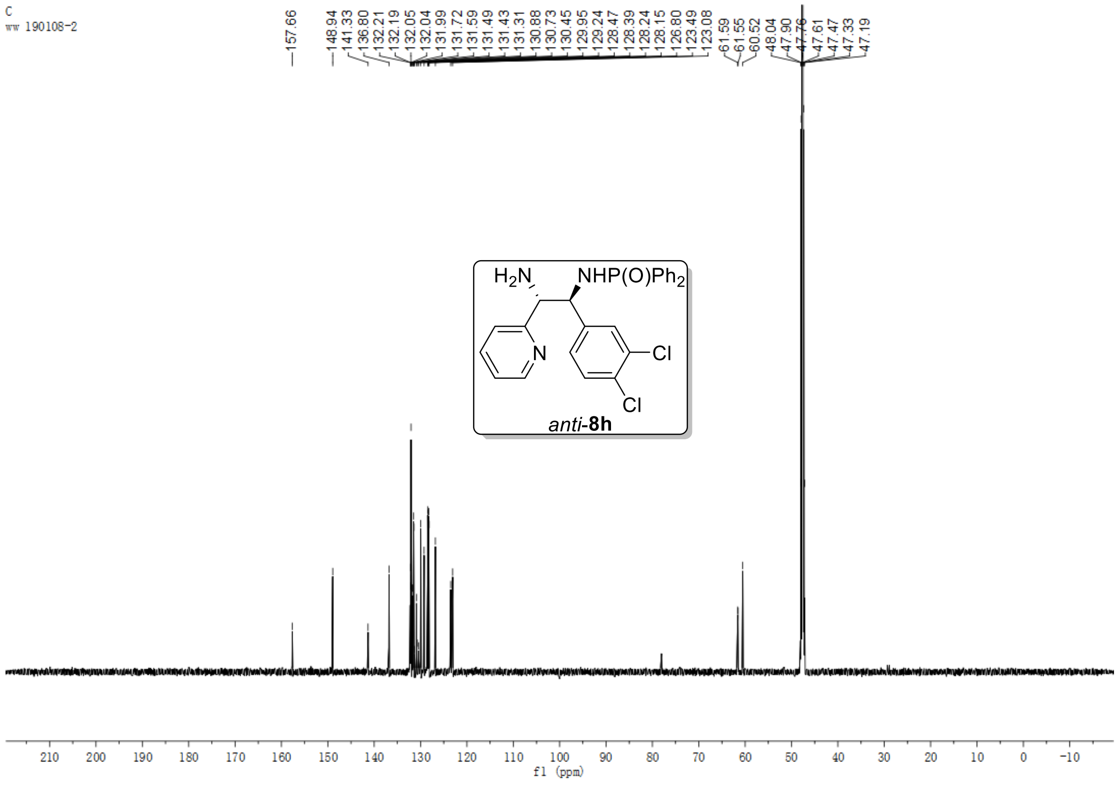

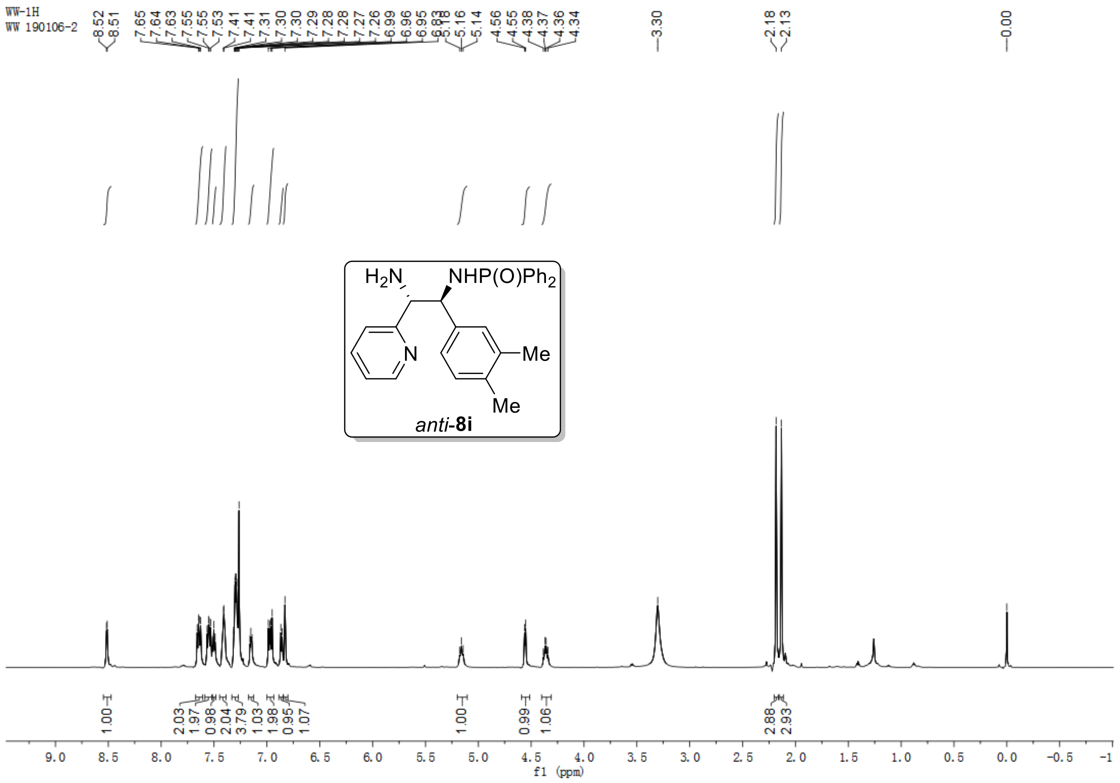
WW-1H
WW 190106-2
H2N NHP(O)Ph2
N
Me
Me
anti-8i
f1 (ppm)

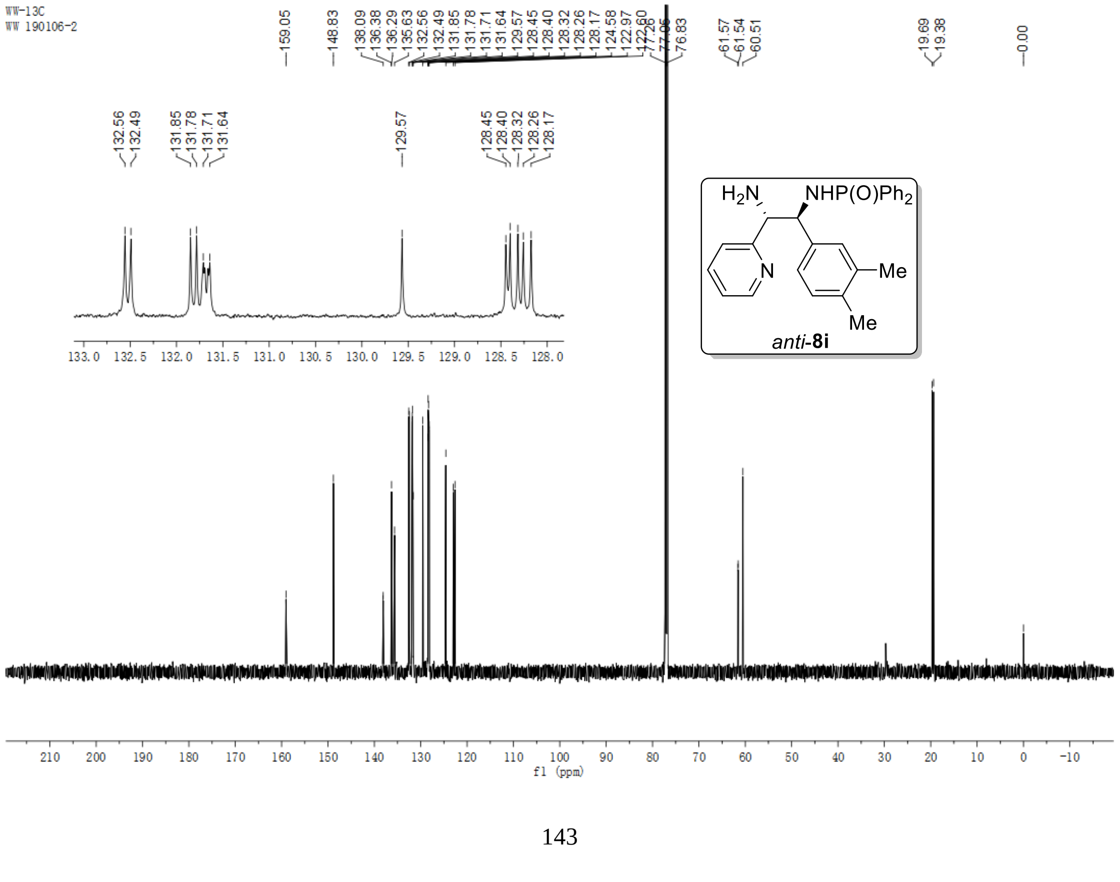
WW-13C
WW 190106-2
H2N NHP(O)Ph2
N
Me
Me
anti-8i
f1 (ppm)

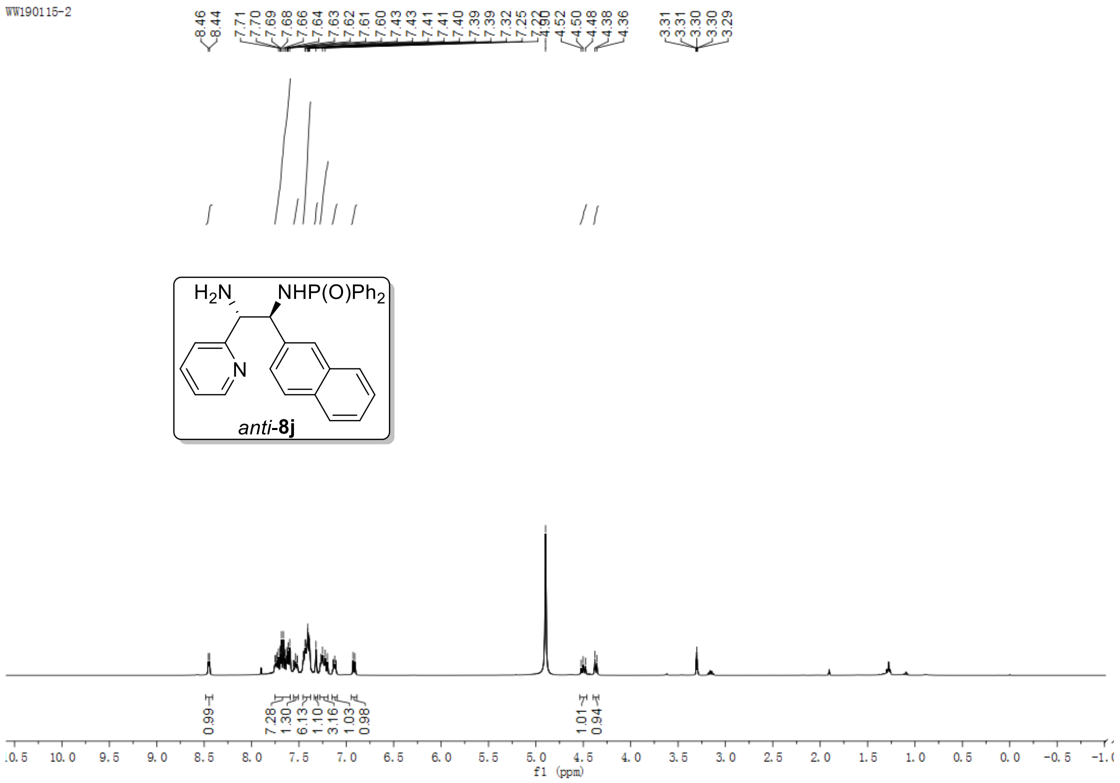
WW190115-2
8.46
8.44
7.71
7.70
7.69
7.68
7.66
7.64
7.63
7.62
7.61
7.60
7.43
7.43
7.41
7.40
7.39
7.39
7.32
7.25
4.52
4.50
4.48
4.38
4.36
3.31
3.31
3.30
3.30
3.29
H2N
NHP(O)Ph2
N
anti-8j
0.99
7.28
1.30
6.13
1.10
3.16
1.03
0.98
1.01
0.94
f1 (ppm)

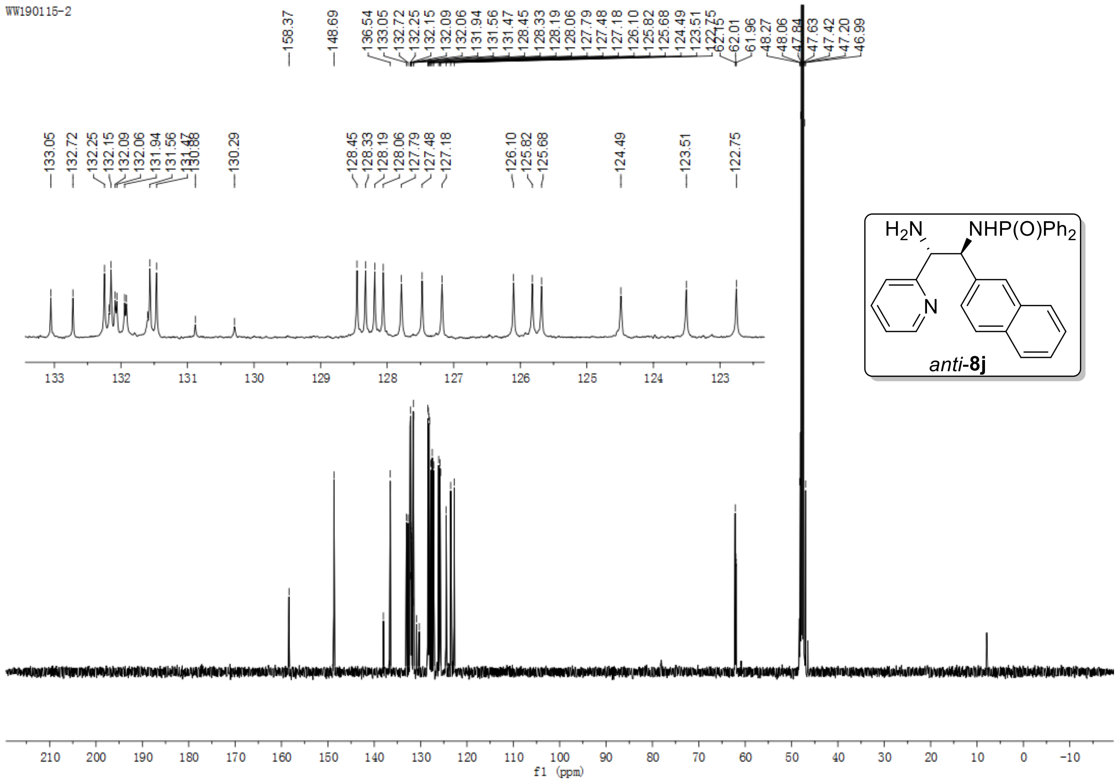
WW190115-2
158.37
148.69
136.54
133.05
132.72
132.25
132.15
132.09
132.06
131.94
131.56
131.47
128.45
128.33
128.19
128.06
127.79
127.48
127.18
126.10
125.82
125.68
124.49
123.51
122.75
62.15
62.01
61.96
48.27
48.06
47.84
47.63
47.42
47.20
46.99
130.29
H2N
NHP(O)Ph2
N
anti-8j
f1 (ppm)

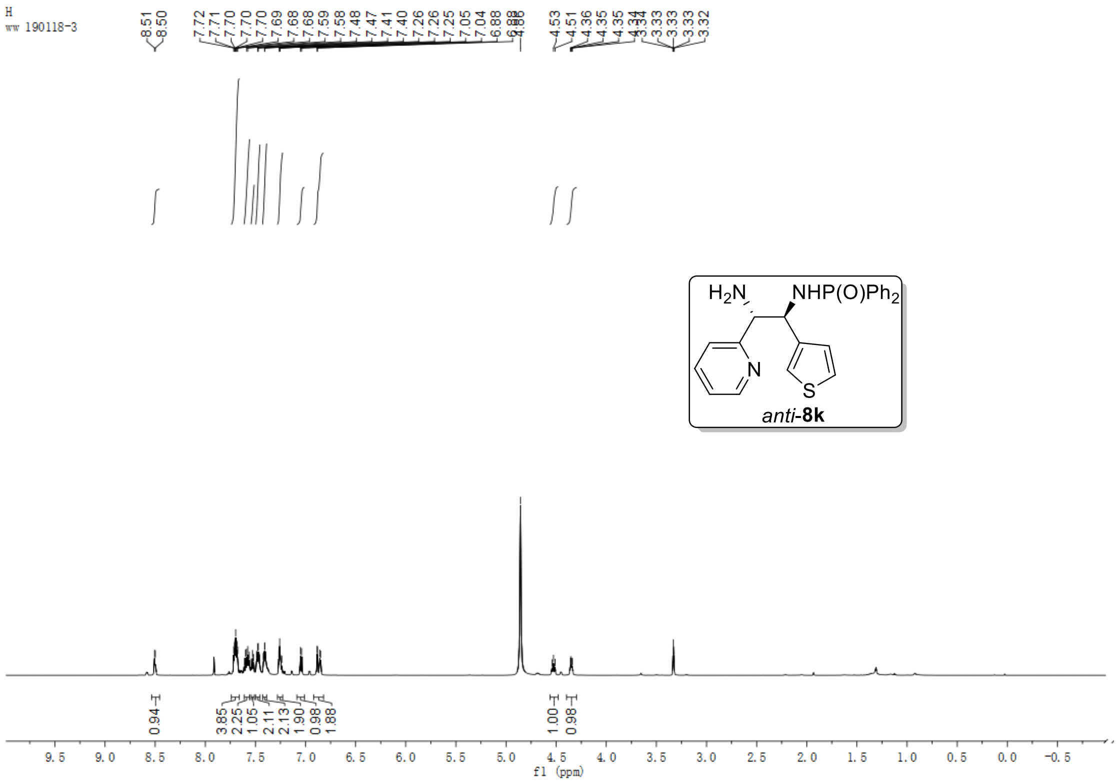
H
ww 190118-3
8.51
8.50
7.72
7.71
7.70
7.70
7.69
7.68
7.59
7.58
7.48
7.47
7.41
7.40
7.26
7.26
7.25
7.05
7.04
6.88
4.53
4.51
4.36
4.35
3.33
3.33
3.33
3.32
H2N
NHP(O)Ph2
N
S
anti-8k
0.94
3.85
2.25
1.05
2.11
2.13
1.90
0.98
1.88
1.00
0.98
9.5
9.0
8.5
8.0
7.5
7.0
6.5
6.0
5.5
5.0
4.5
4.0
3.5
3.0
2.5
2.0
1.5
1.0
0.5
0.0
-0.5
f1 (ppm)

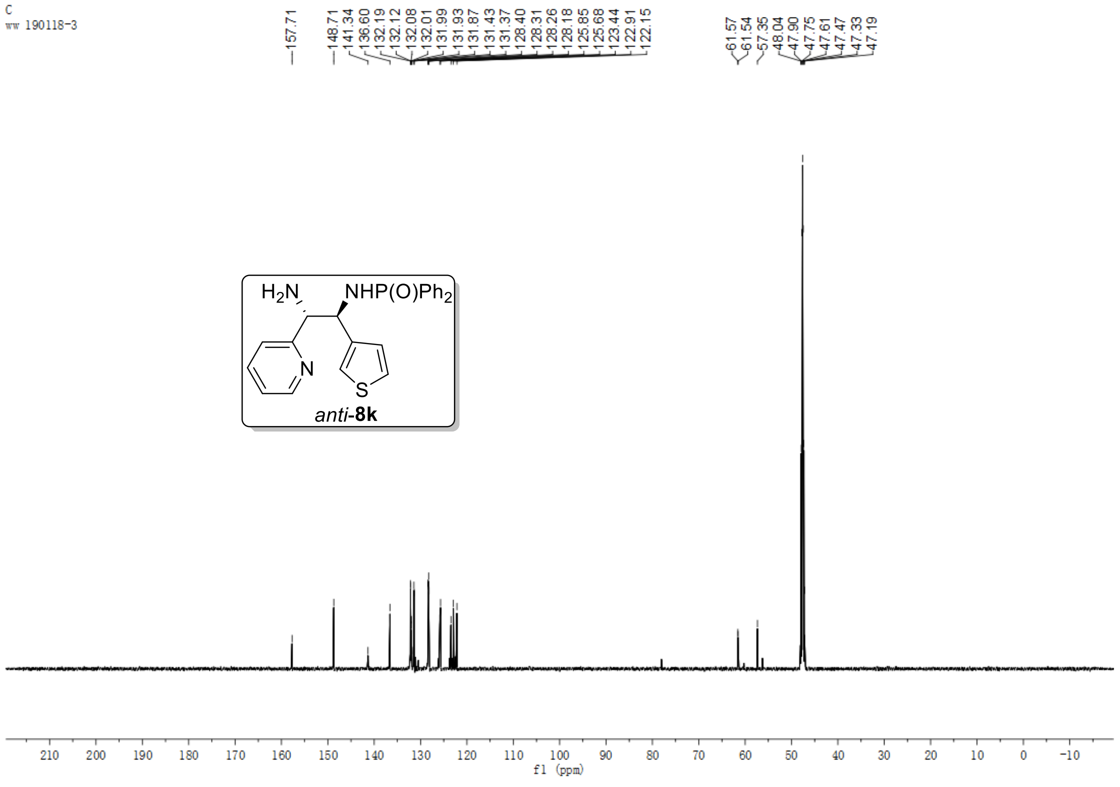
C
ww 190118-3
157.71
148.71
141.34
136.60
132.19
132.12
132.08
132.01
131.99
131.93
131.87
131.43
131.37
128.40
128.31
128.26
128.18
125.85
125.68
123.44
122.91
122.15
61.57
61.54
57.35
48.04
47.90
47.75
47.61
47.47
47.33
47.19
H2N
NHP(O)Ph2
N
S
anti-8k
210
200
190
180
170
160
150
140
130
120
110
100
90
80
70
60
50
40
30
20
10
0
-10
f1 (ppm)

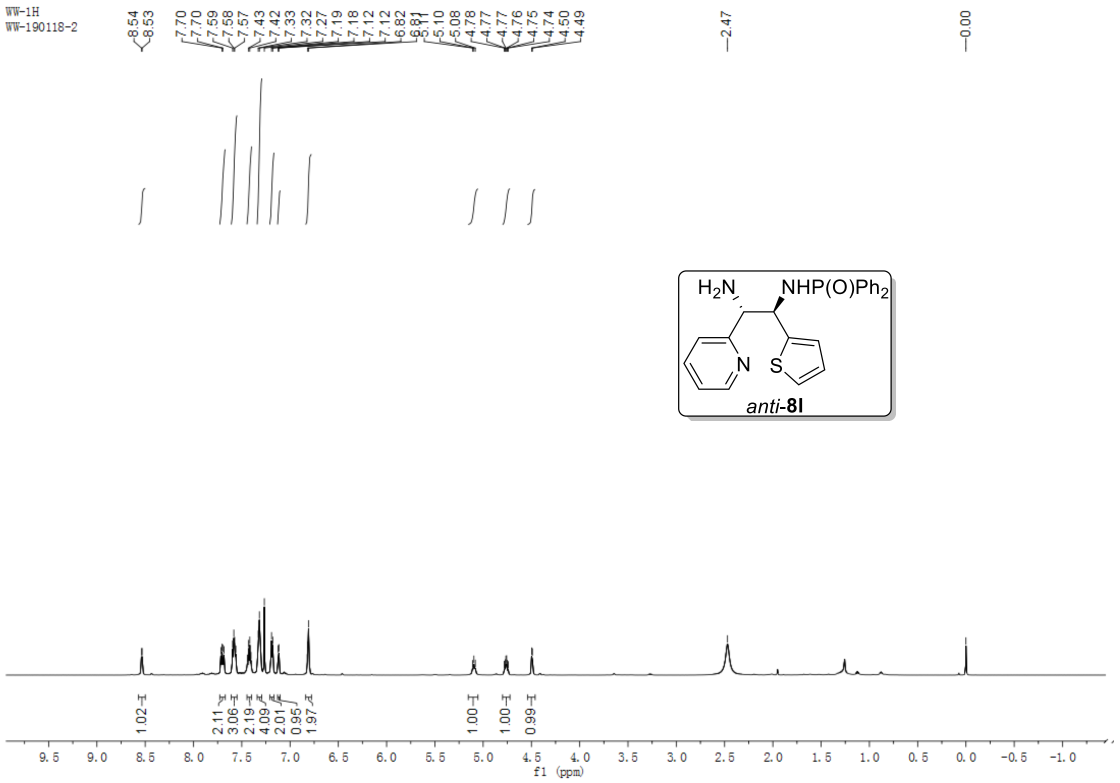
WW-1H
WW-190118-2
H2N
NHP(O)Ph2
N
S
anti-8l
f1 (ppm)

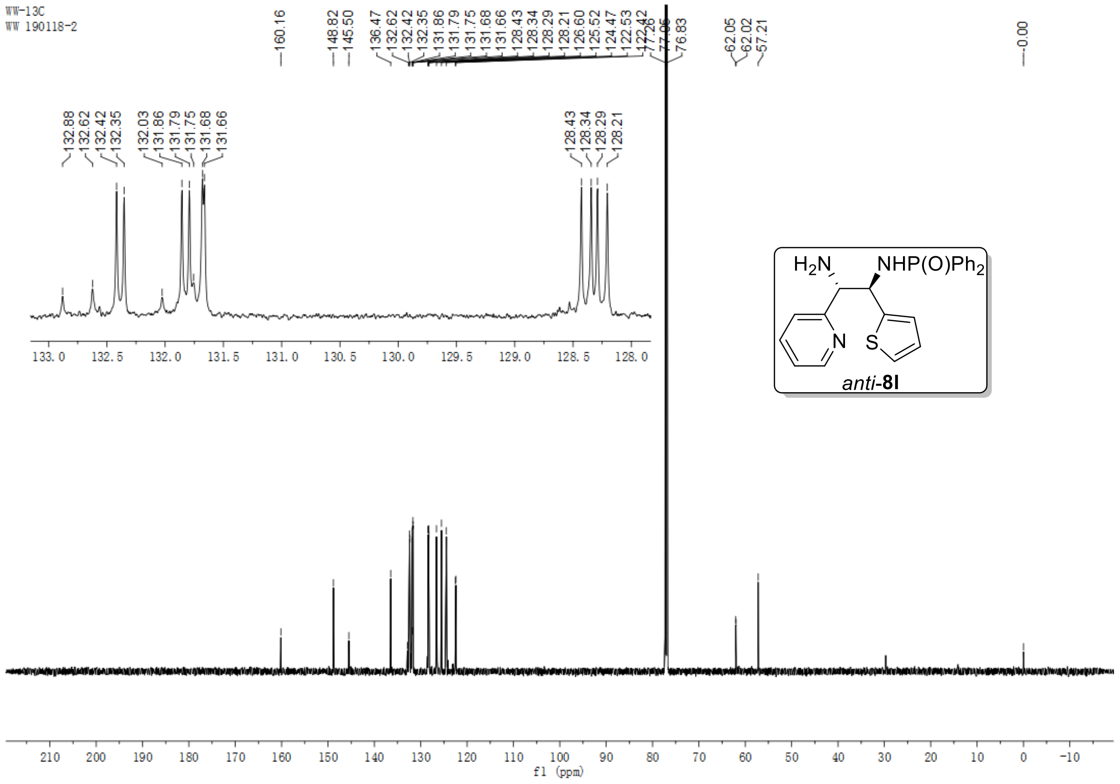
WW-13C
WW 190118-2
H2N
NHP(O)Ph2
N
S
anti-8l
f1 (ppm)

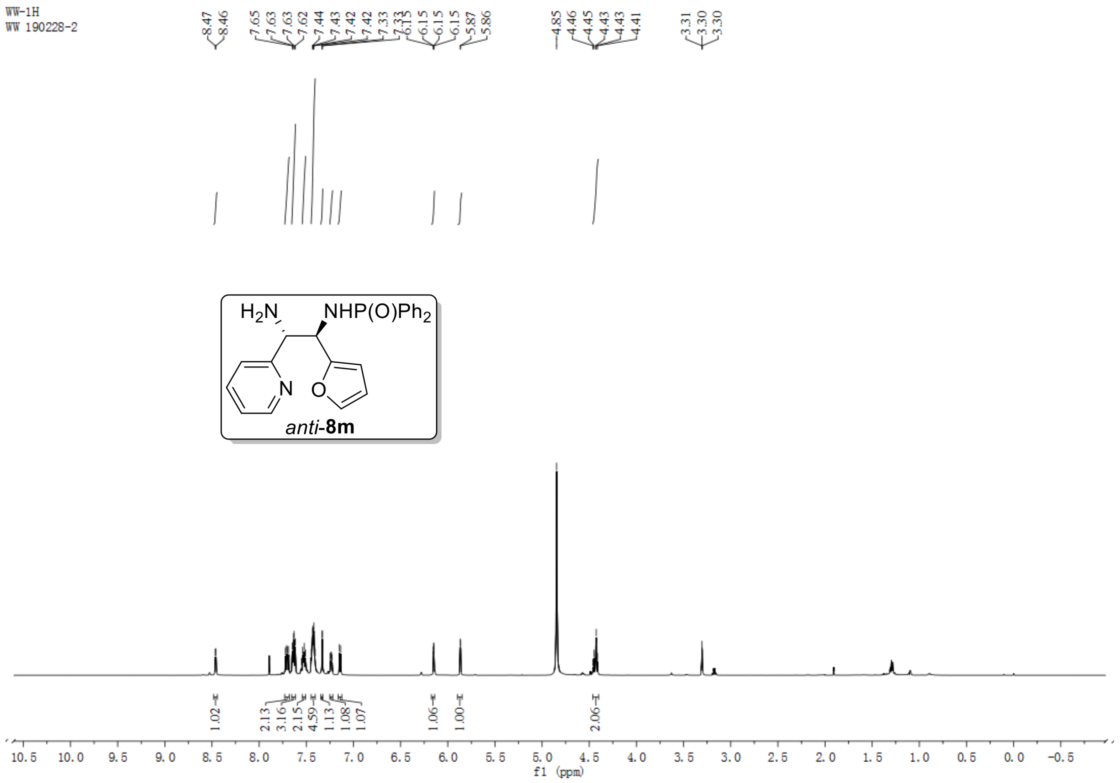
WW-1H
WW 190228-2
f1 (ppm)
H2N
NHP(O)Ph2
N
O
anti-8m

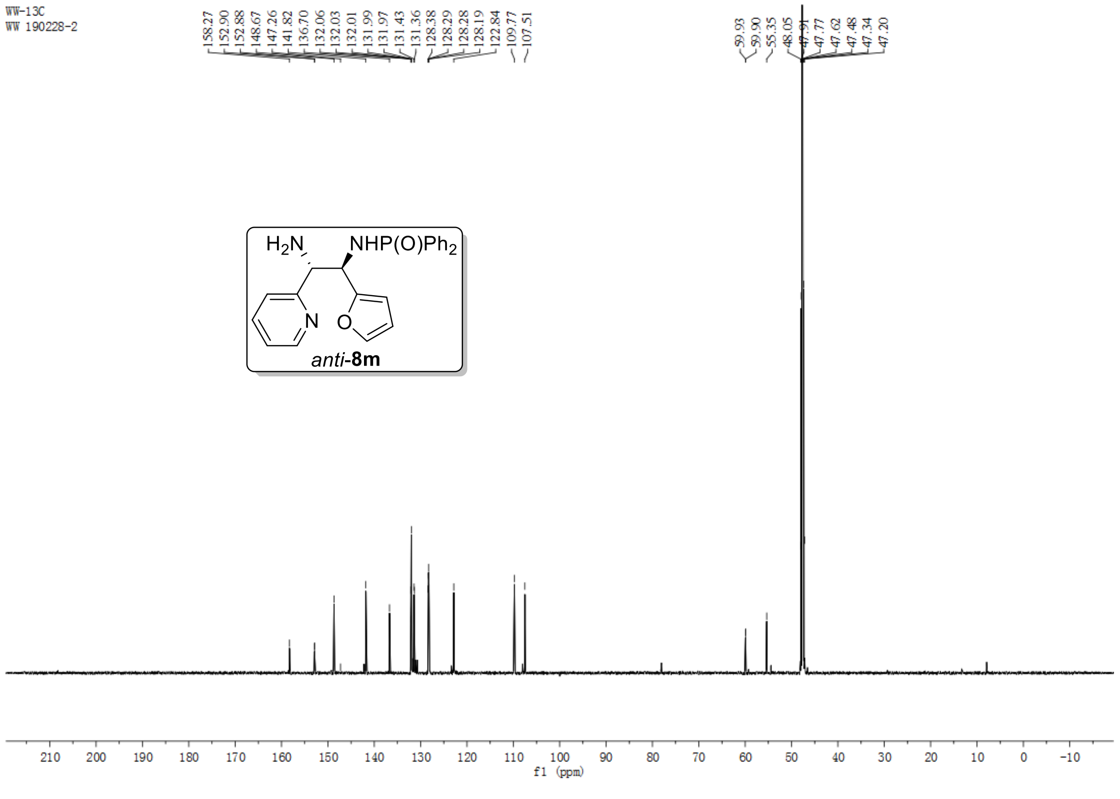
WW-13C
WW 190228-2
f1 (ppm)
H2N
NHP(O)Ph2
N
O
anti-8m

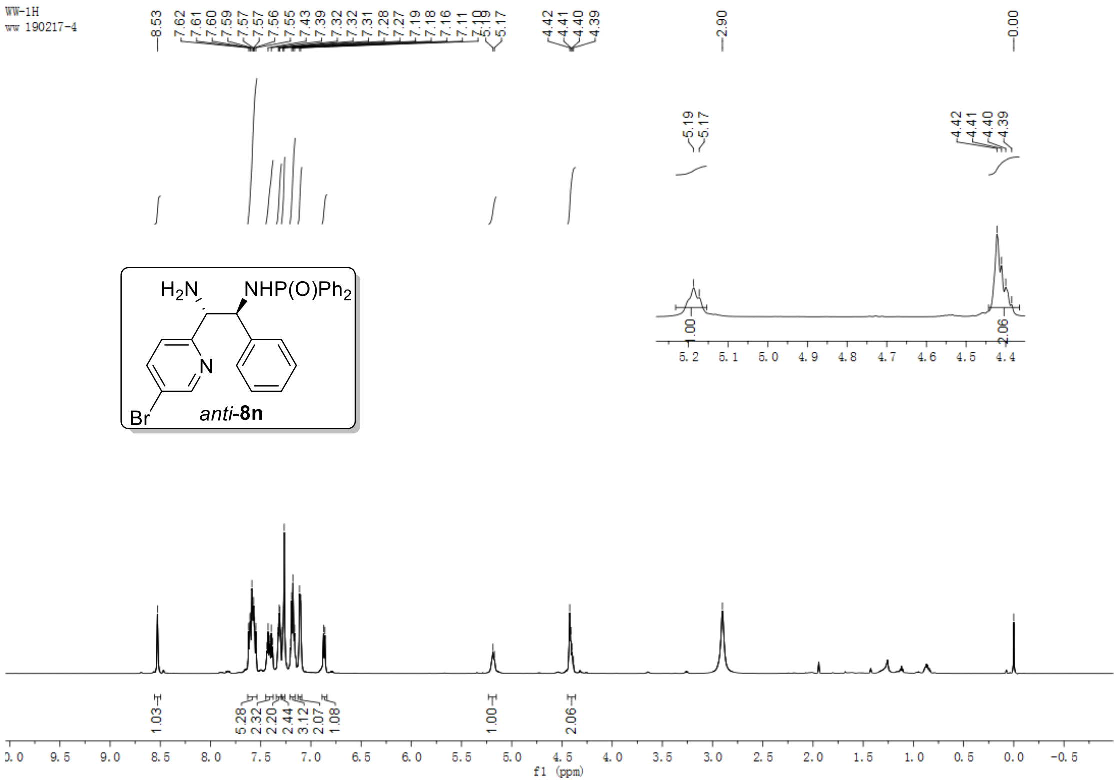
WW-1H
ww 190217-4
H2N
NHP(O)Ph2
N
Br
anti-8n
f1 (ppm)

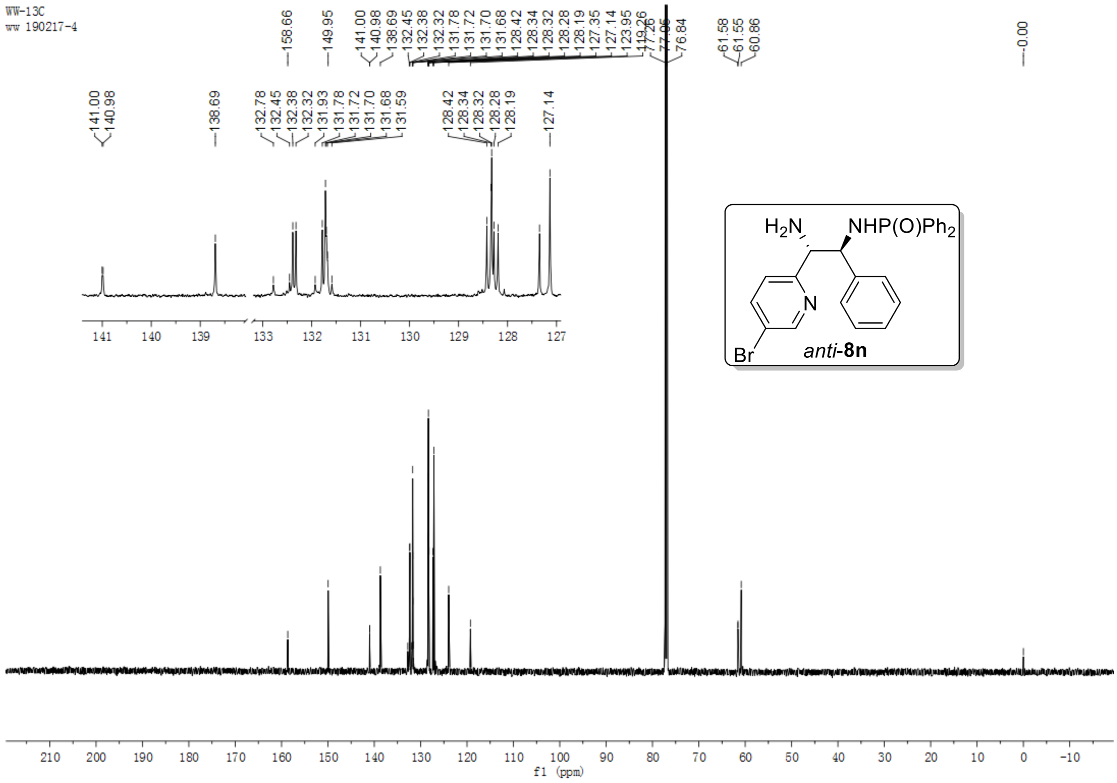
WW-13C
ww 190217-4
H2N
NHP(O)Ph2
N
Br
anti-8n
f1 (ppm)

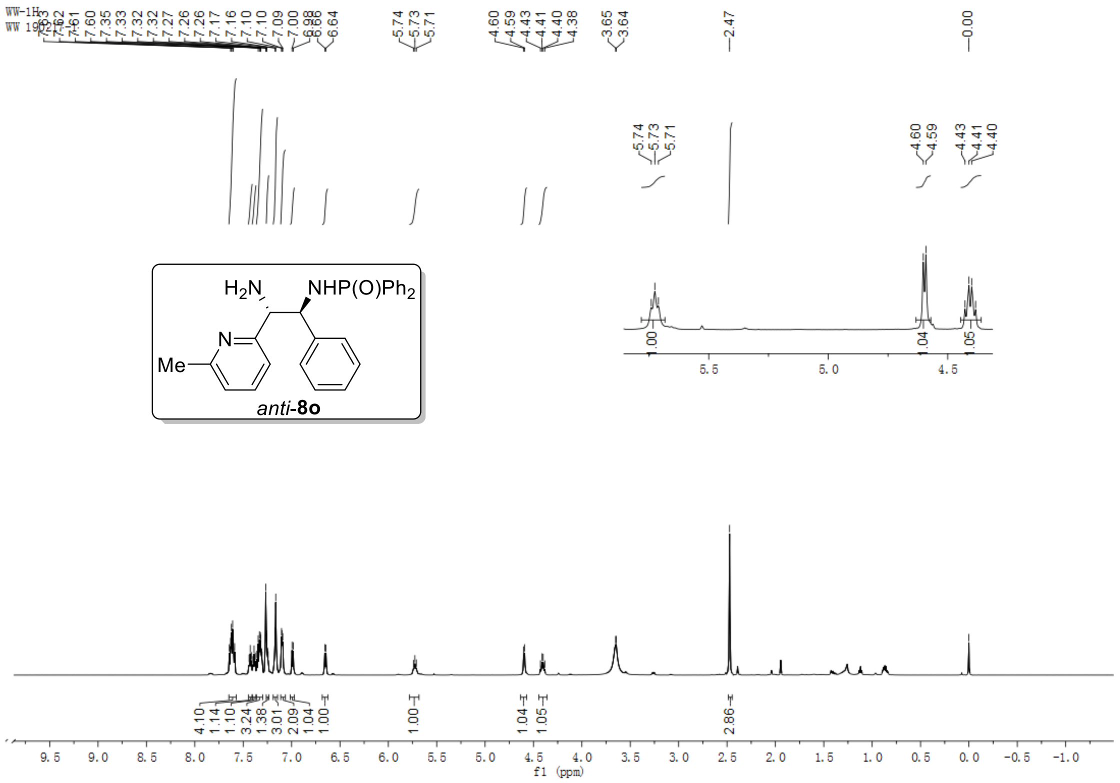
WW-1H
anti-8o
f1 (ppm)

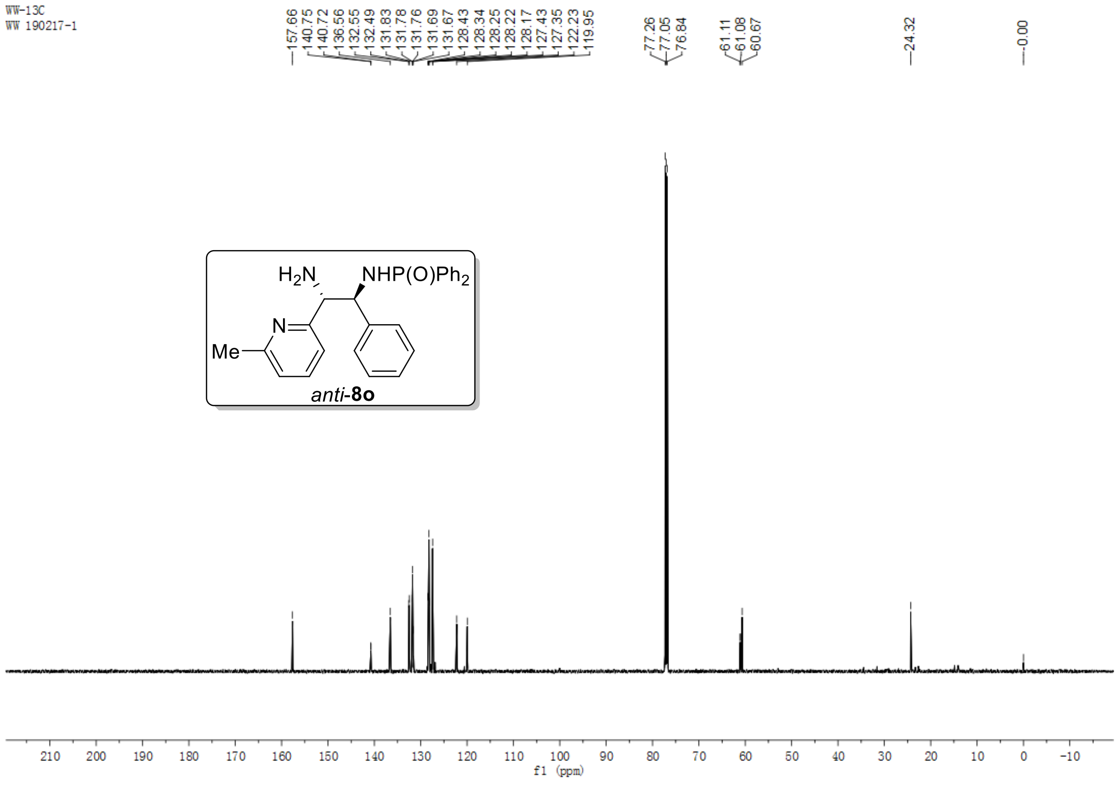
WW-13C
WW 190217-1
anti-8o
f1 (ppm)

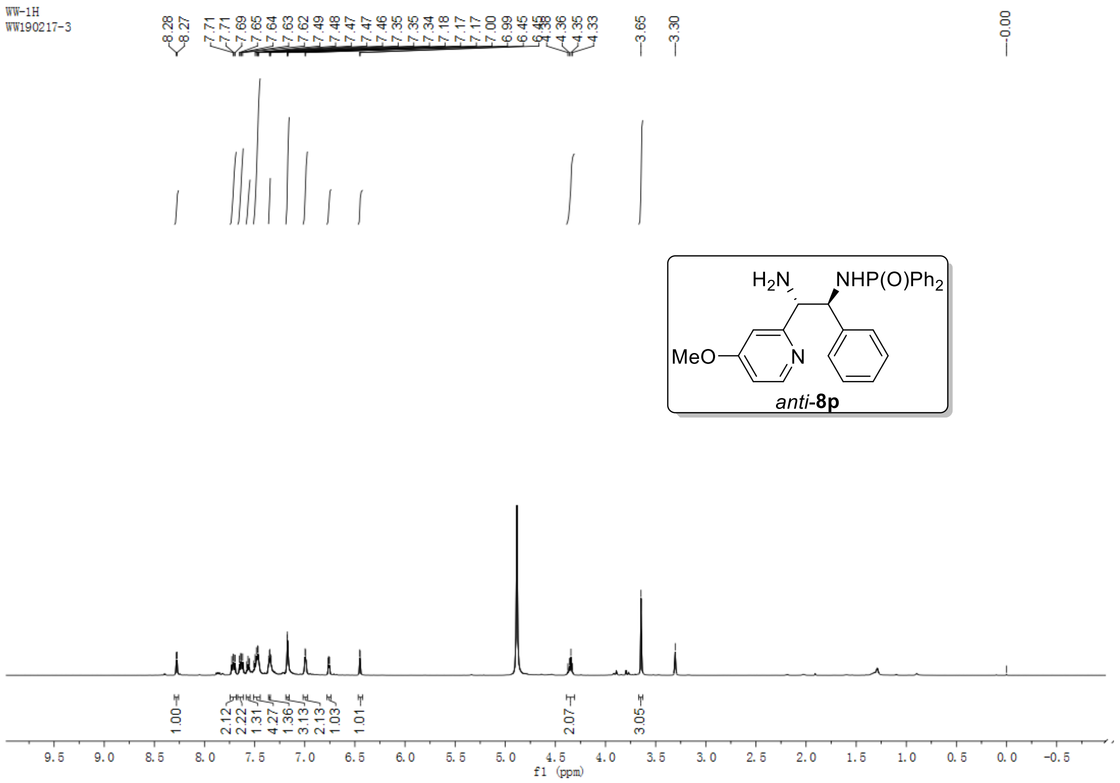
WW-1H
WW190217-3
H2N
NHP(O)Ph2
MeO
N
anti-8p
f1 (ppm)

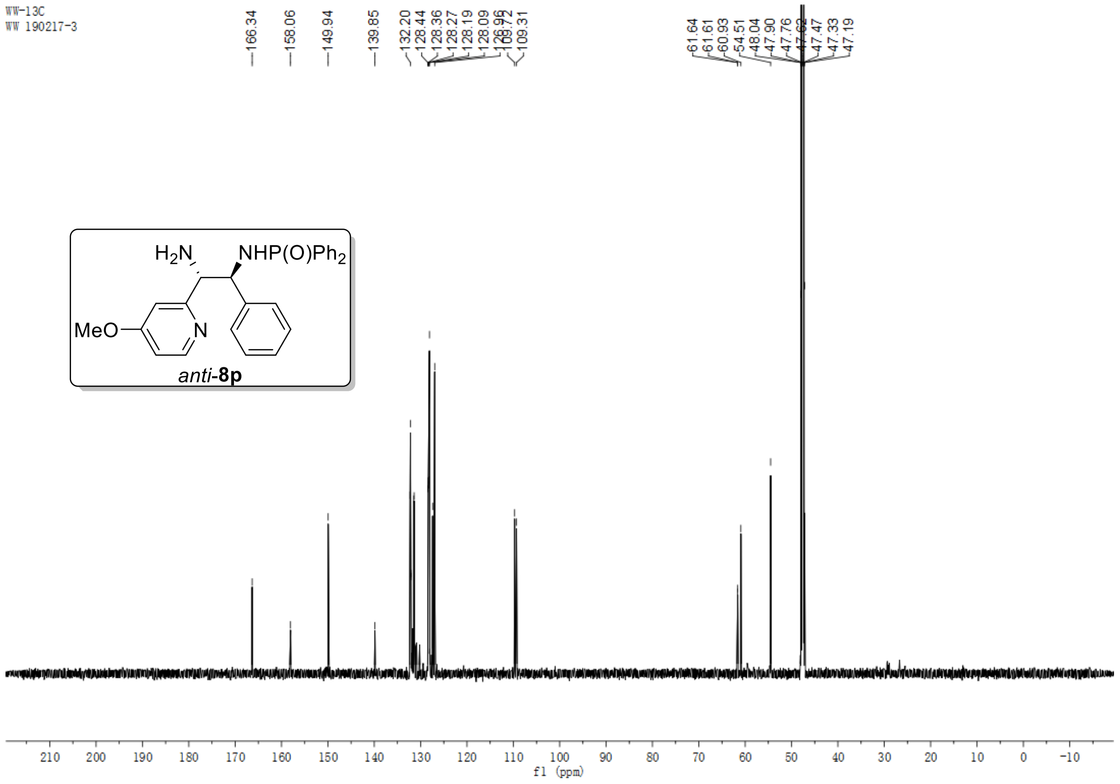
WW-13C
WW 190217-3
H2N
NHP(O)Ph2
MeO
N
anti-8p
f1 (ppm)

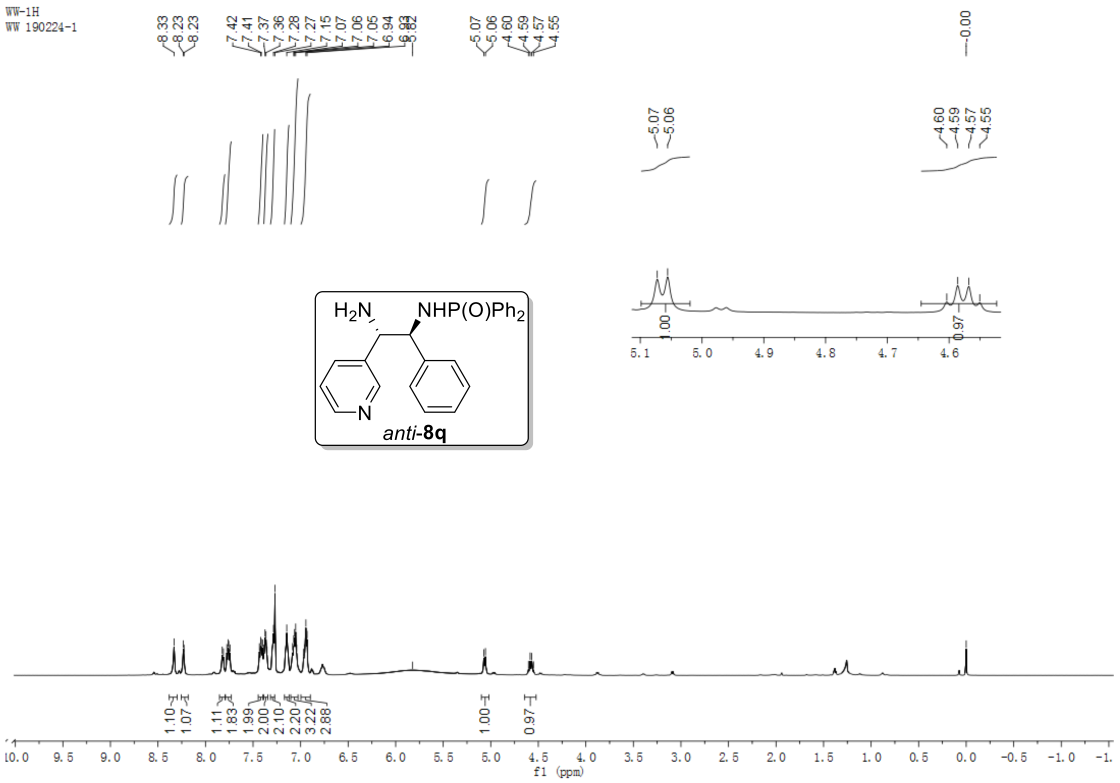
WW-1H
WW 190224-1
H2N
NHP(O)Ph2
N
anti-8q
f1 (ppm)

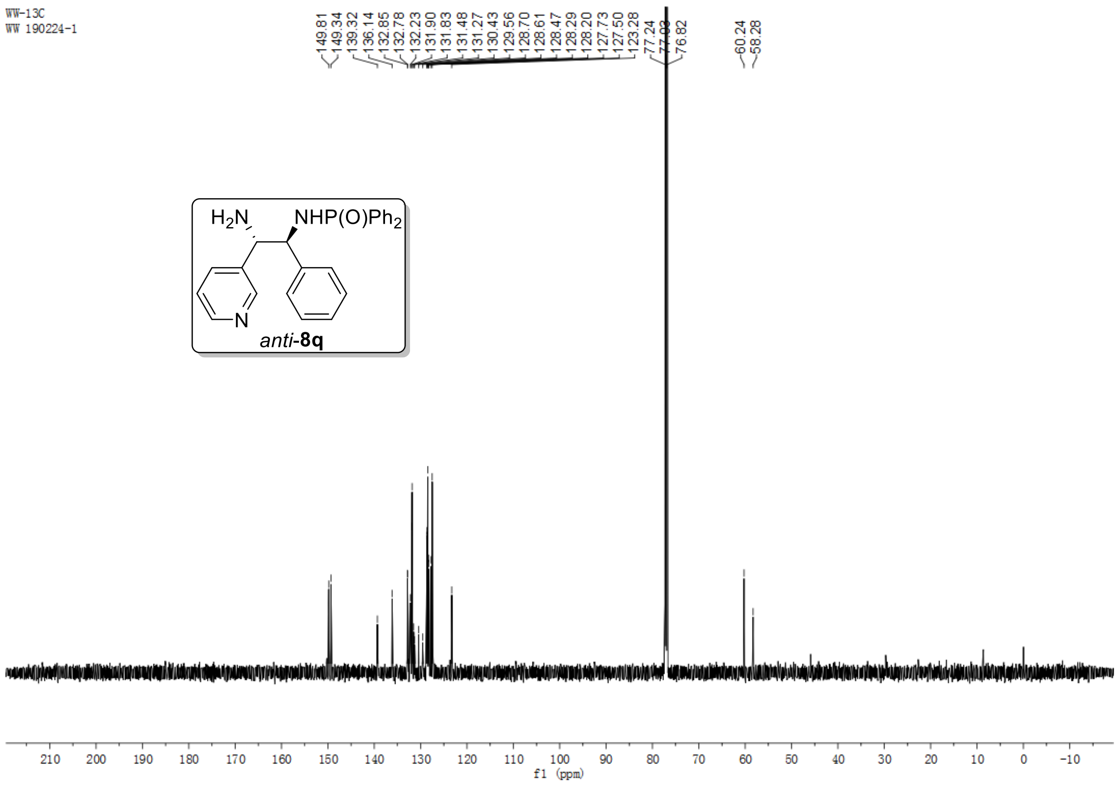
WW-13C
WW 190224-1
H2N
NHP(O)Ph2
N
anti-8q
f1 (ppm)

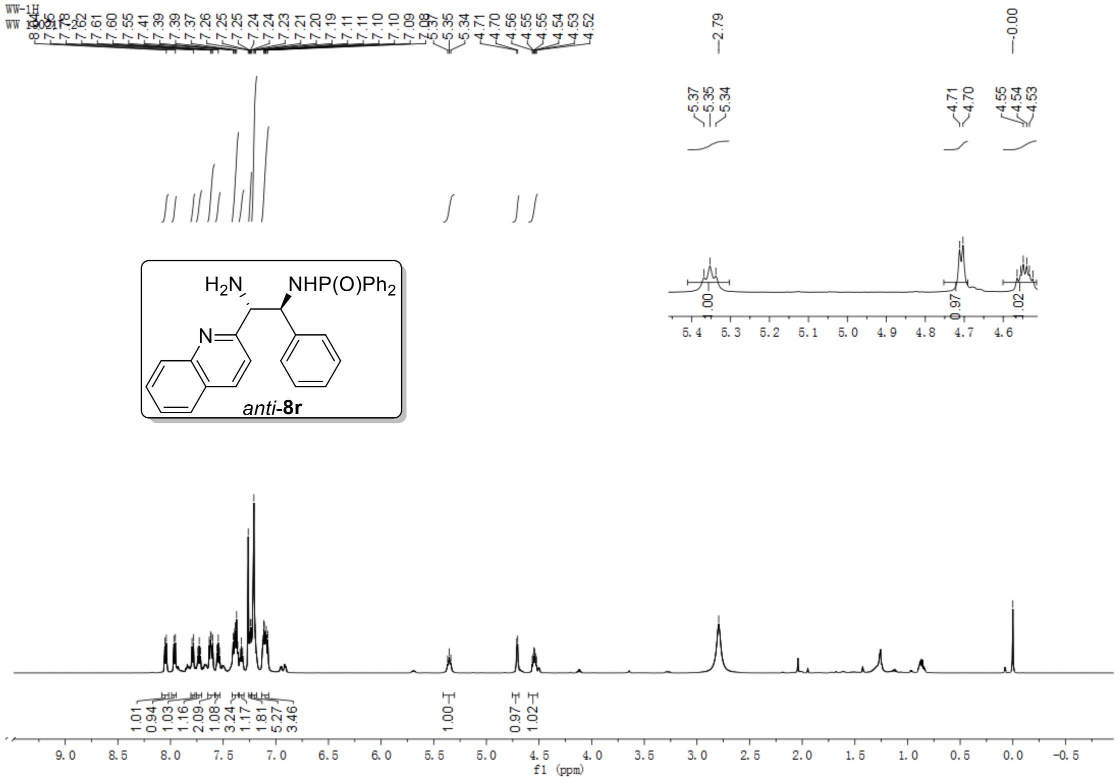
WW-1H
WW 190217-2
2.79
0.00
5.37
5.35
5.34
4.71
4.70
4.55
4.54
4.53
H2N
NHP(O)Ph2
N
anti-8r
1.00
0.97
1.02
f1 (ppm)

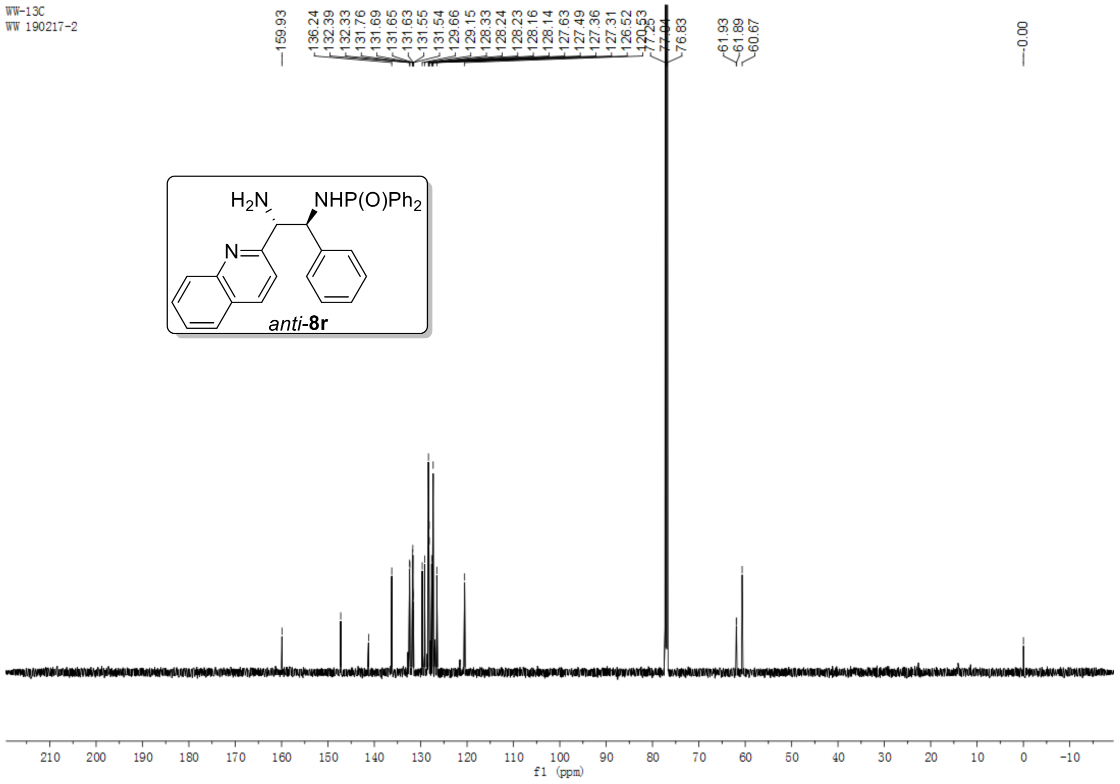
WW-13C
WW 190217-2
159.93
136.24
132.39
132.33
131.76
131.69
131.65
131.63
131.55
131.54
129.66
129.15
128.33
128.24
128.23
128.16
128.14
127.63
127.49
127.36
127.31
126.52
120.53
77.25
77.04
76.83
61.93
61.89
60.67
0.00
H2N
NHP(O)Ph2
N
anti-8r
f1 (ppm)

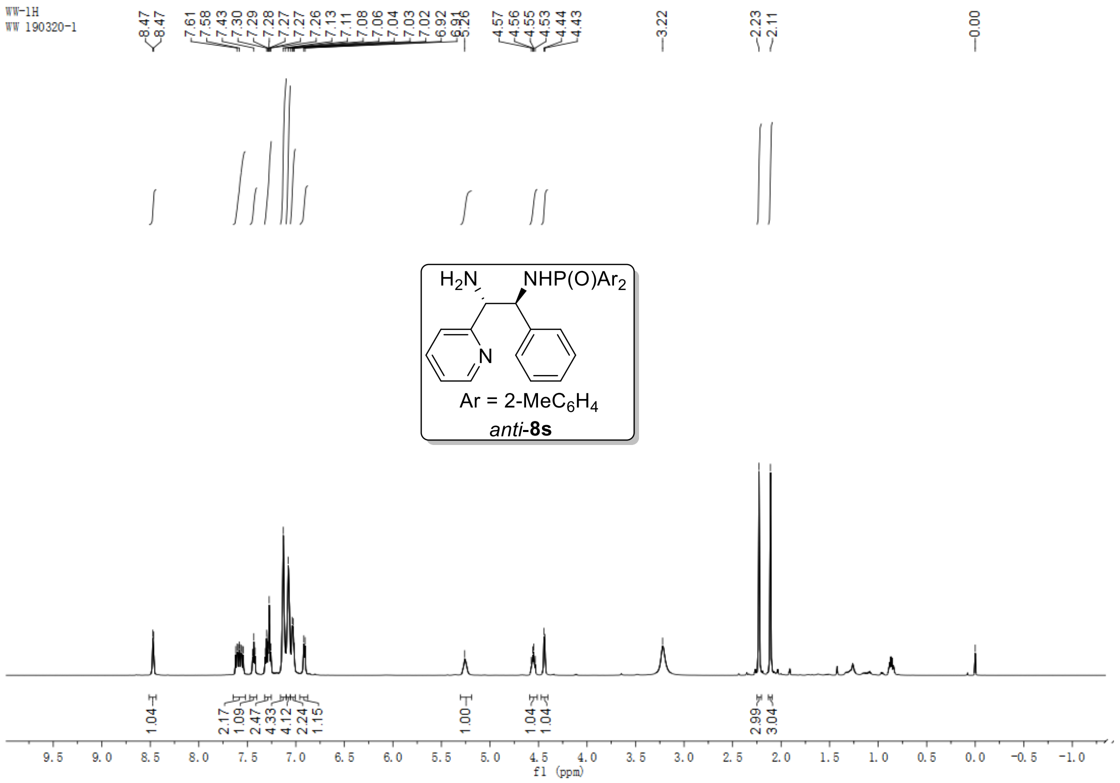
WW-1H
WW 190320-1
H_2N
NHP(O)Ar_2
N
Ar = 2-MeC_6H_4
anti-**8s**
f1 (ppm)

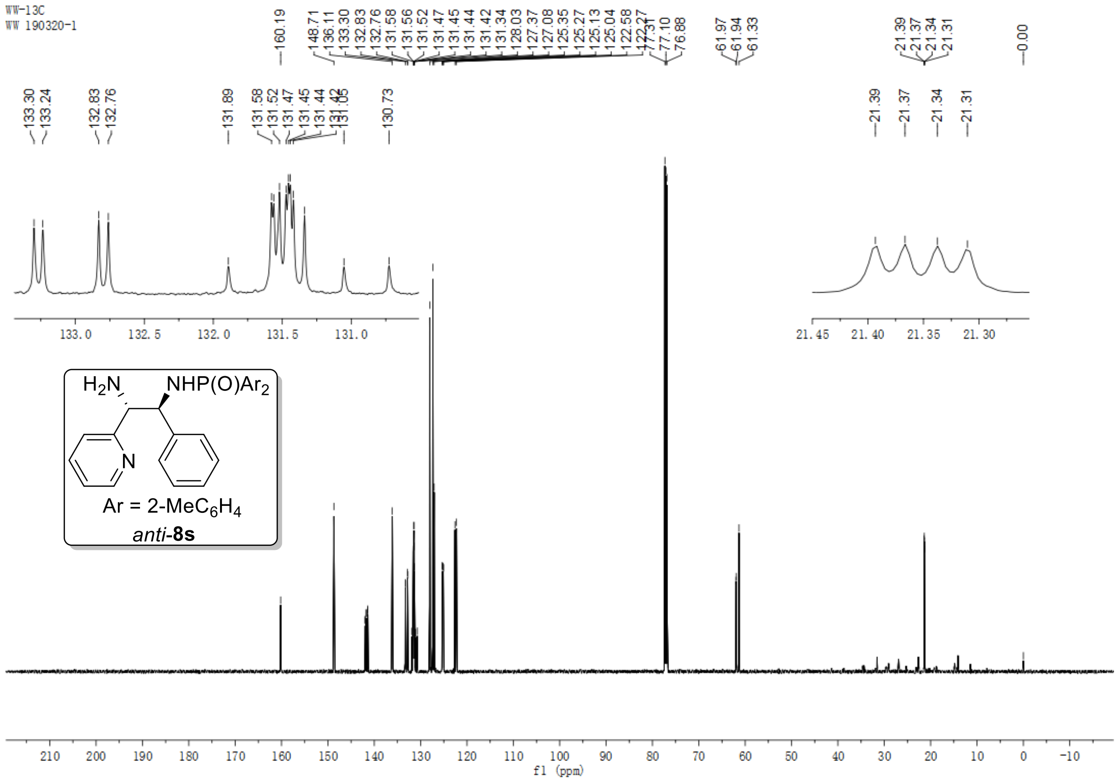
WW-13C
WW 190320-1
H_2N
NHP(O)Ar_2
N
Ar = 2-MeC_6H_4
anti-**8s**
f1 (ppm)

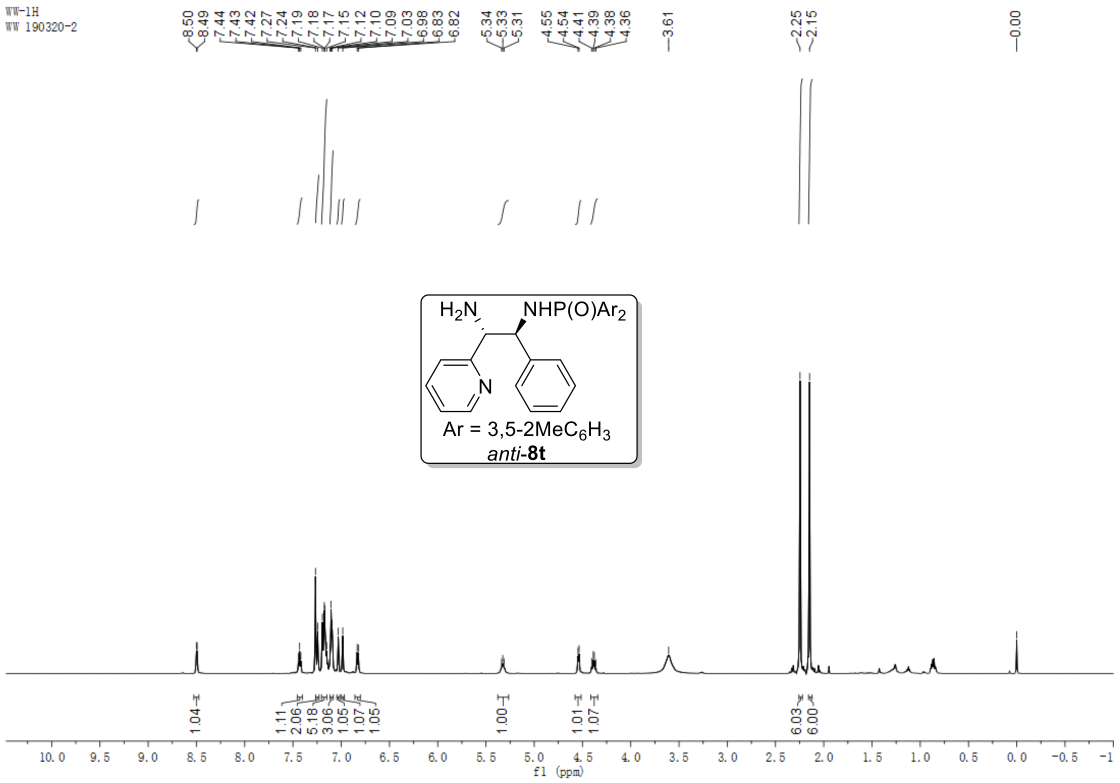
WW-1H
WW 190320-2
8.50
8.49
7.44
7.43
7.42
7.27
7.24
7.19
7.18
7.17
7.15
7.12
7.10
7.09
7.03
6.98
6.83
6.82
5.34
5.33
5.31
4.55
4.54
4.41
4.39
4.38
4.36
3.61
2.25
2.15
0.00
H2N
NHP(O)Ar2
N
Ar = 3,5-2MeC6H3
anti-8t
1.04
1.11
2.06
5.18
3.06
1.05
1.07
1.05
1.00
1.01
1.07
6.03
6.00
f1 (ppm)

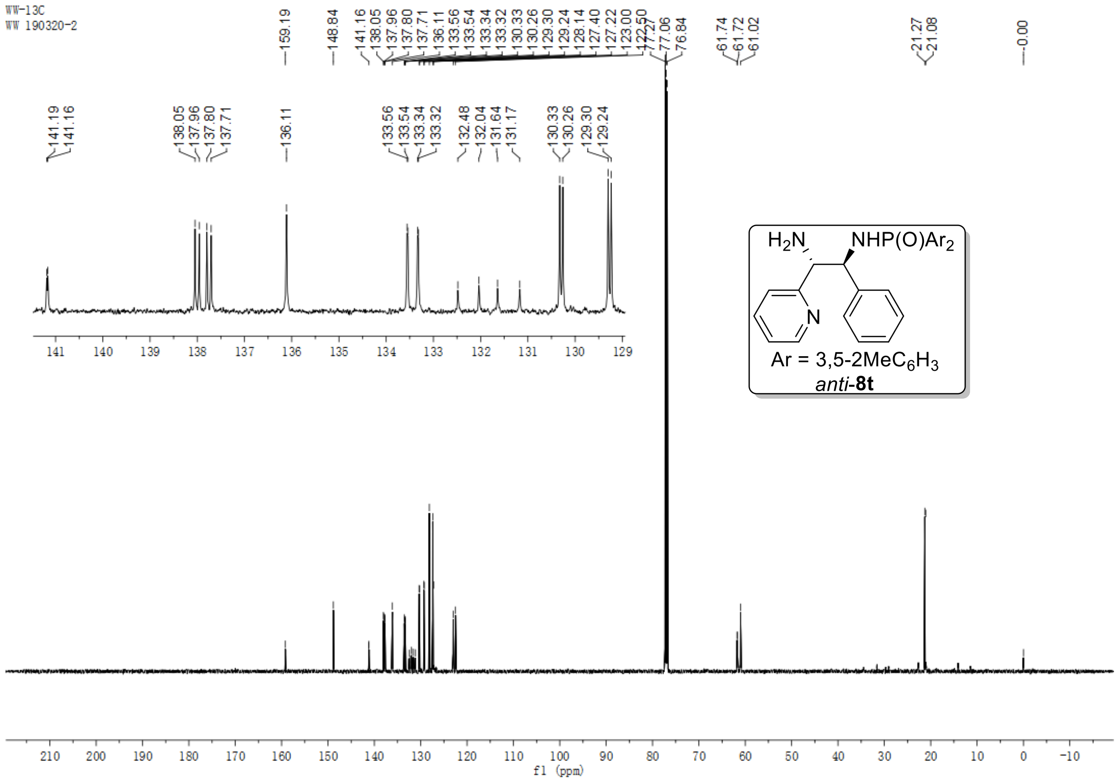
WW-13C
WW 190320-2
159.19
148.84
141.16
138.05
137.96
137.80
137.71
136.11
133.56
133.54
133.34
133.32
130.33
130.26
129.30
129.24
128.14
127.40
127.22
123.00
122.50
77.27
77.06
76.84
61.74
61.72
61.02
21.27
21.08
0.00
141.19
141.16
138.05
137.96
137.80
137.71
136.11
133.56
133.54
133.34
133.32
132.48
132.04
131.64
131.17
130.33
130.26
129.30
129.24
H2N
NHP(O)Ar2
N
Ar = 3,5-2MeC6H3
anti-8t
f1 (ppm)

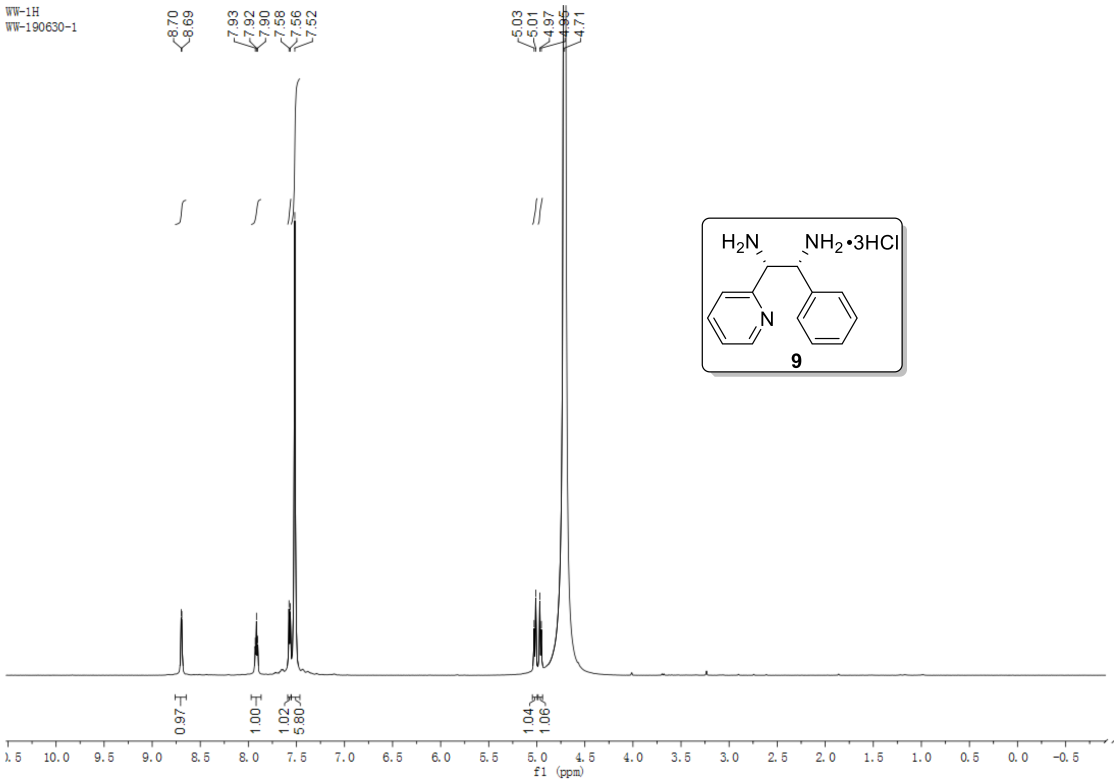
WW-1H
WW-190630-1
8.70
8.69
7.93
7.92
7.90
7.58
7.56
7.52
5.03
5.01
4.97
4.95
4.71
0.97
1.00
1.02
5.80
1.04
1.06
f1 (ppm)
H2N
NH2•3HCl
N
9

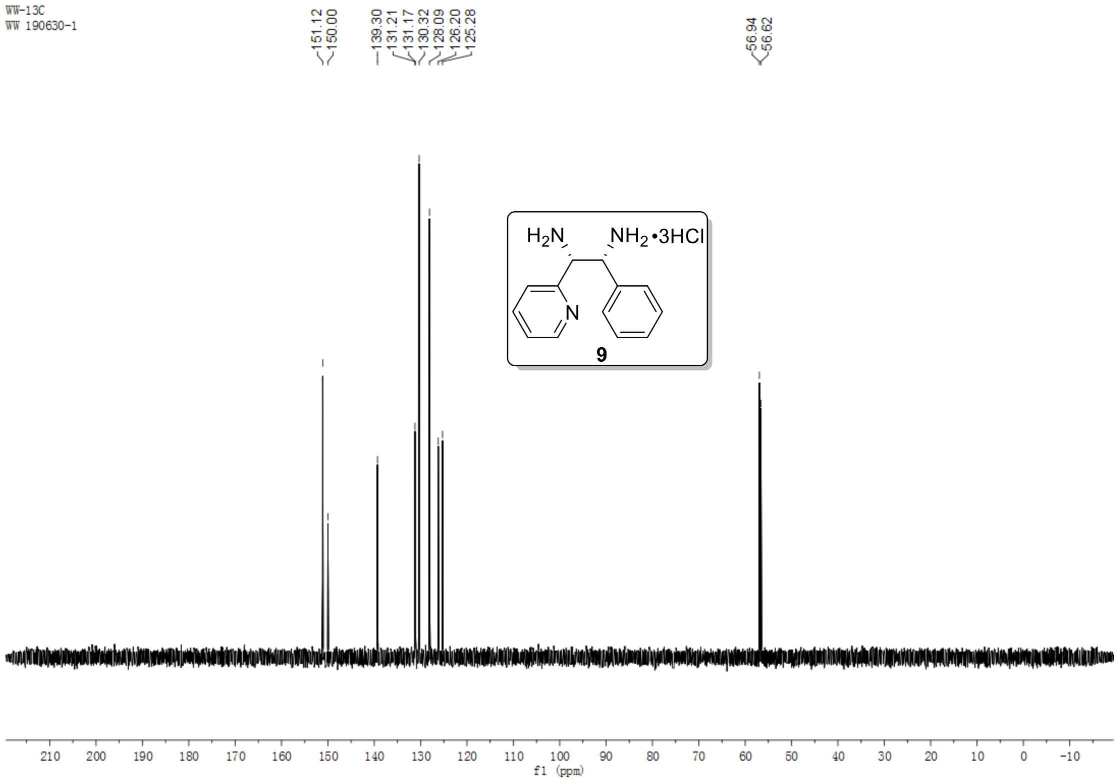
WW-13C
WW 190630-1
151.12
150.00
139.30
131.21
131.17
130.32
128.09
126.20
125.28
56.94
56.62
f1 (ppm)
H2N
NH2•3HCl
N
9

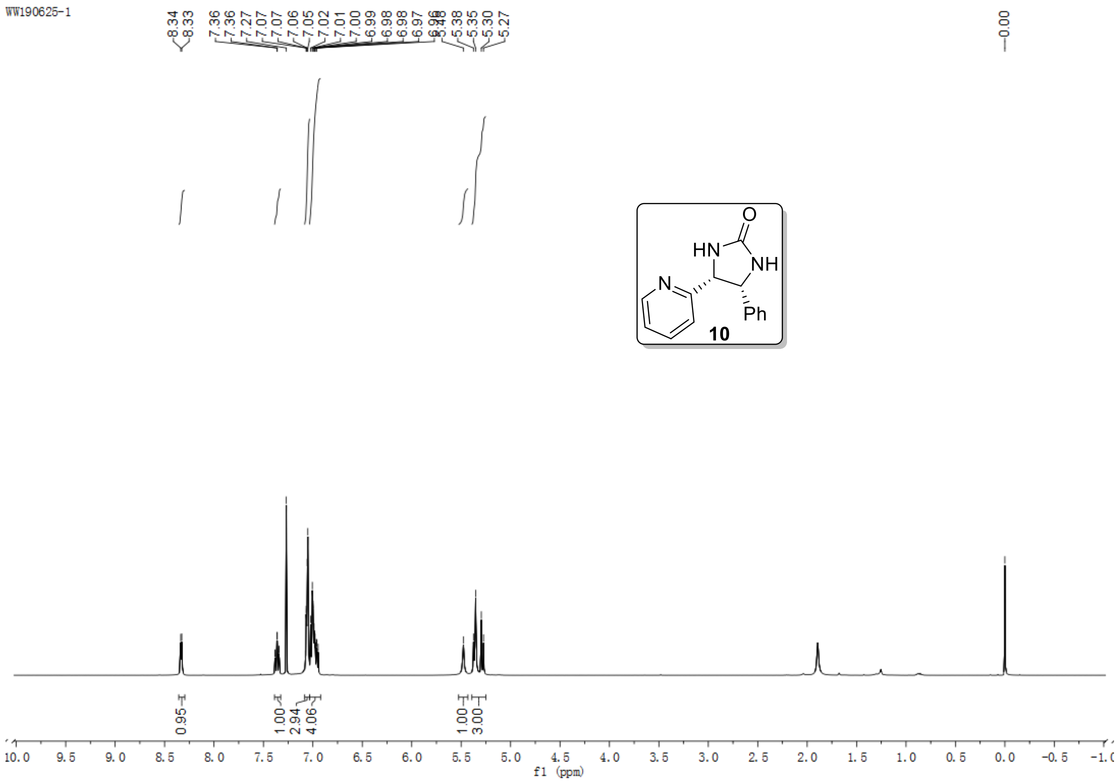
WW190625-1
8.34
8.33
7.36
7.36
7.27
7.07
7.07
7.06
7.05
7.02
7.01
7.00
6.99
6.98
6.98
6.97
5.38
5.35
5.30
5.27
0.00
O
HN
NH
N
Ph
10
0.95
1.00
2.94
4.06
1.00
3.00
f1 (ppm)

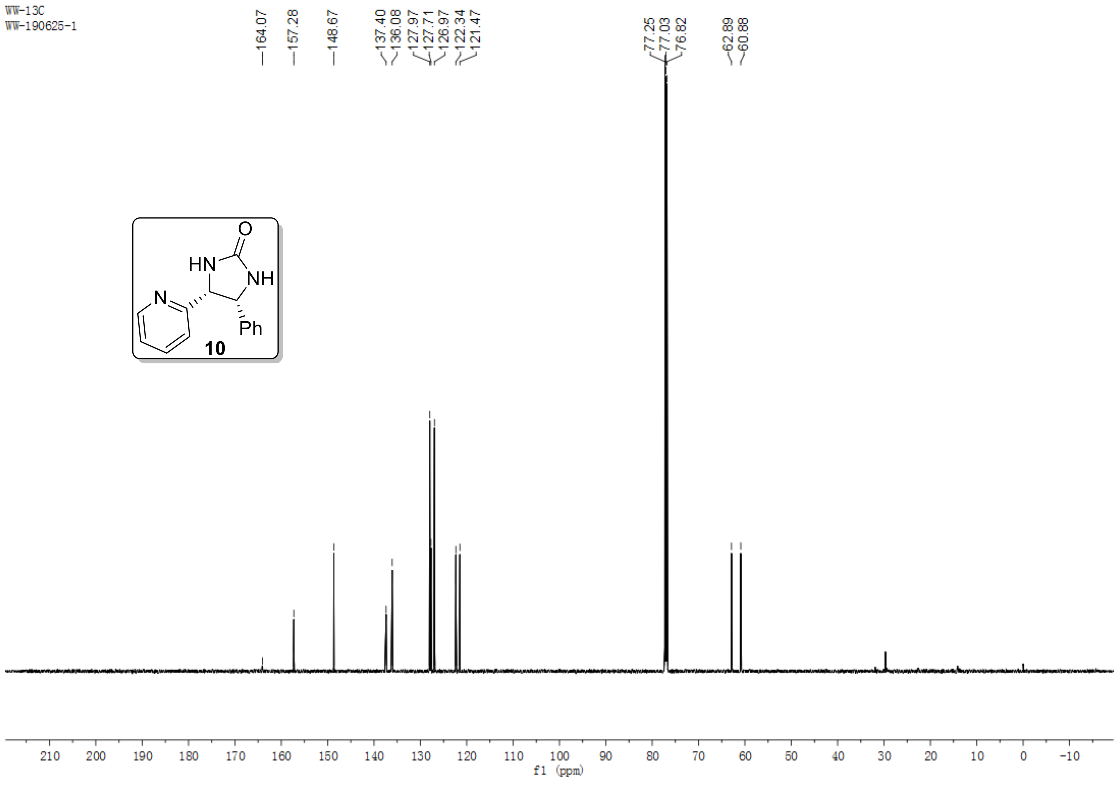
WW-13C
WW-190625-1
164.07
157.28
148.67
137.40
136.08
127.97
127.71
126.97
122.34
121.47
77.25
77.03
76.82
62.89
60.88
O
HN
NH
N
Ph
10
f1 (ppm)

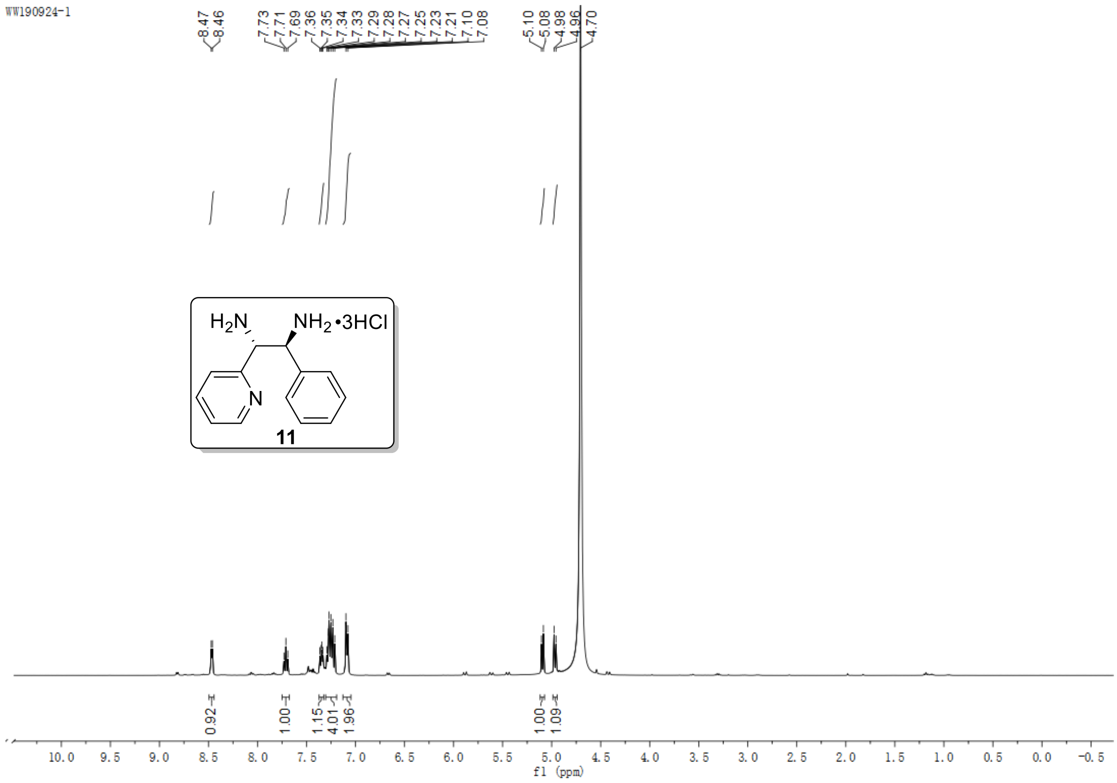
WW190924-1
H2N
NH2•3HCl
N
11
f1 (ppm)

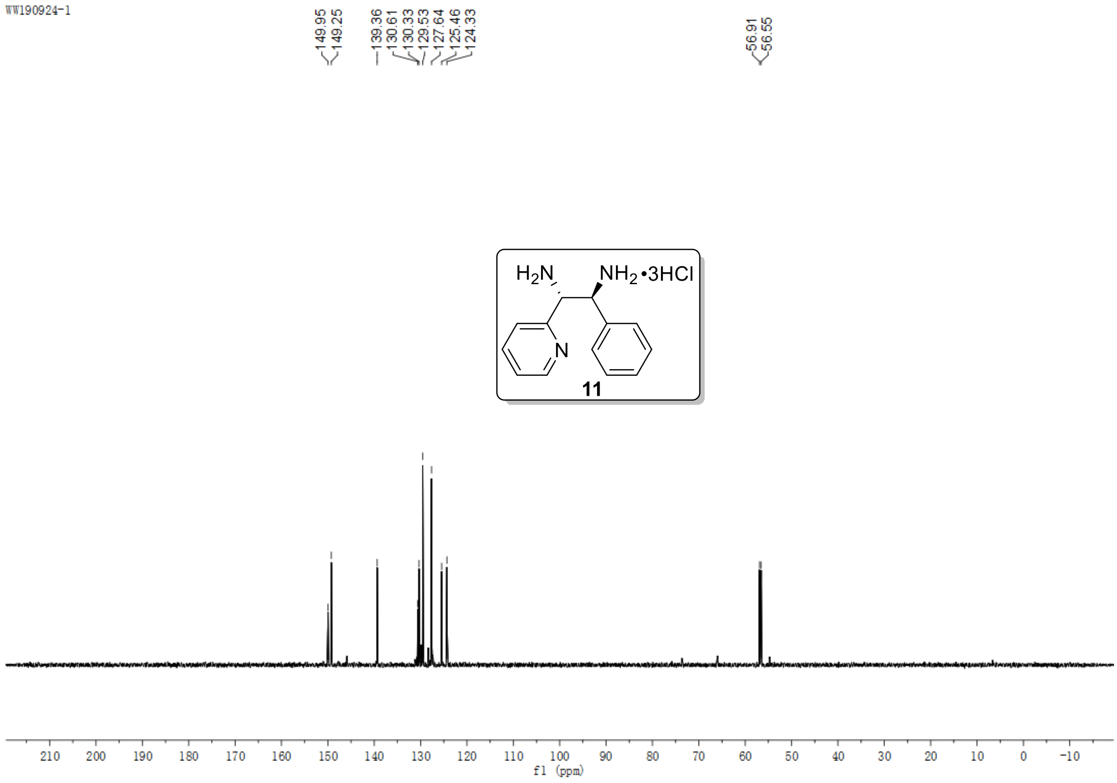
WW190924-1
H2N
NH2•3HCl
N
11
f1 (ppm)

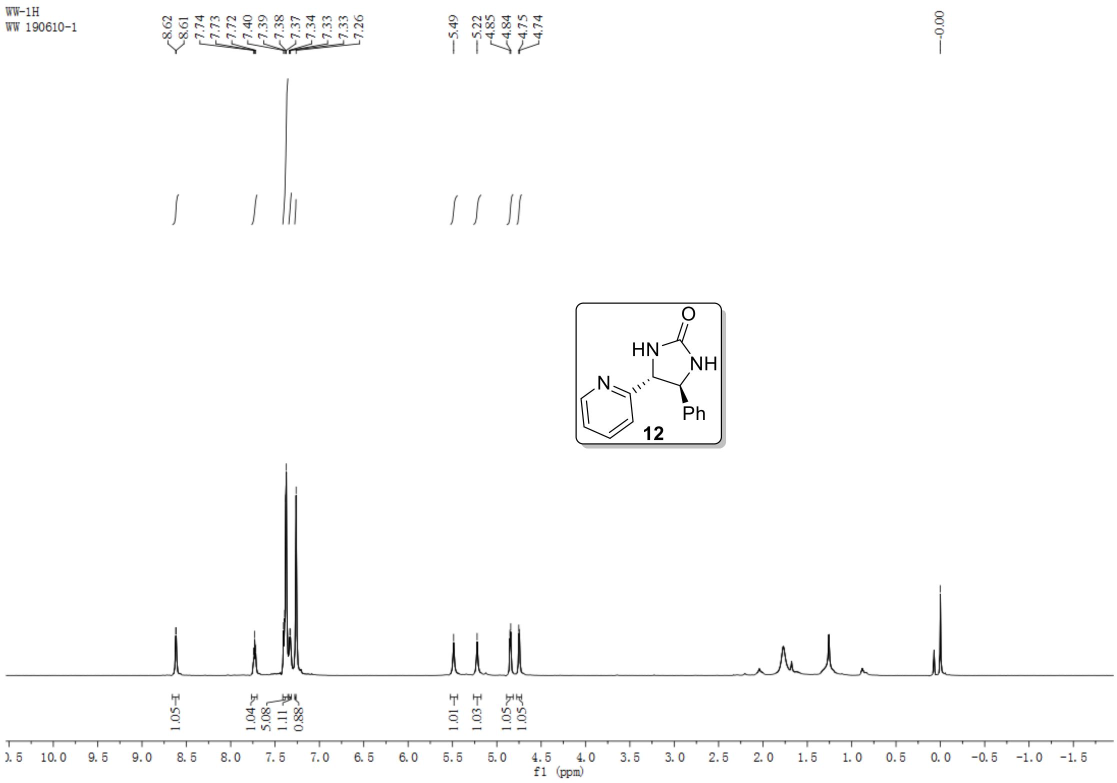
WW-1H
WW 190610-1
8.62
8.61
7.74
7.73
7.72
7.40
7.39
7.38
7.37
7.34
7.33
7.33
7.26
5.49
5.22
4.85
4.84
4.75
4.74
-0.00
HN
O
NH
N
Ph
12
1.05
1.04
5.08
1.11
0.88
1.01
1.03
1.05
1.05
f1 (ppm)

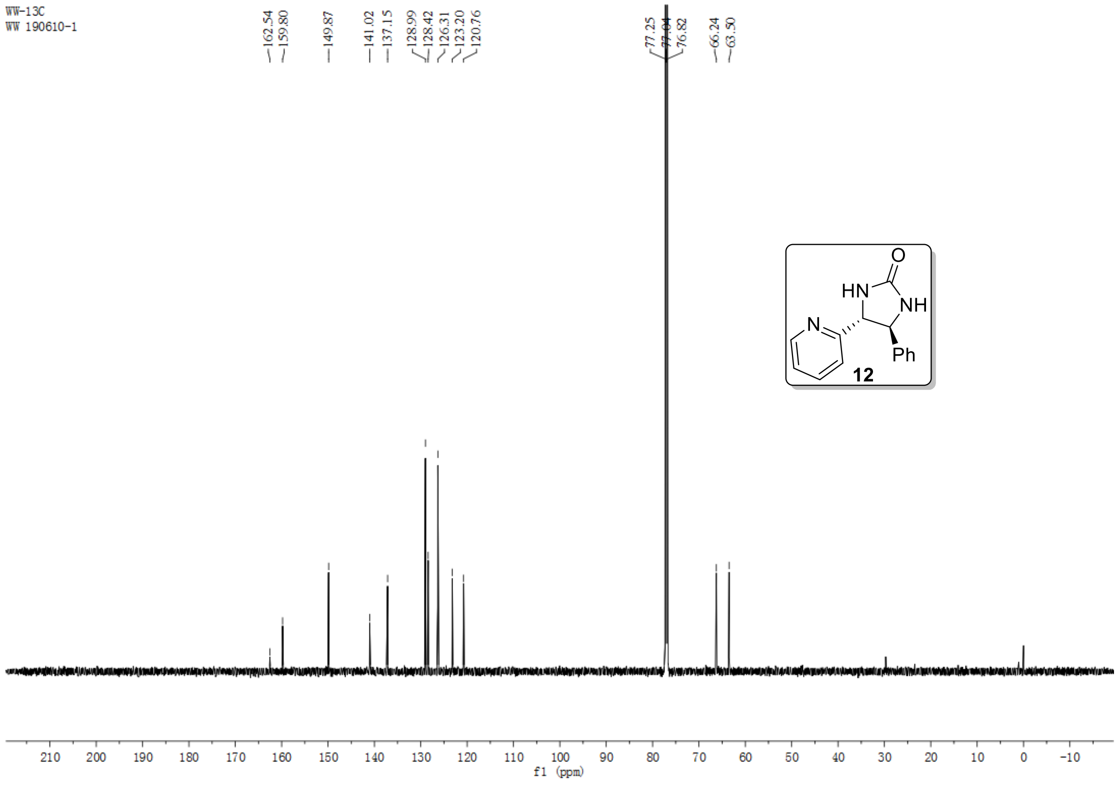
WW-13C
WW 190610-1
162.54
159.80
149.87
141.02
137.15
128.99
128.42
126.31
123.20
120.76
77.25
76.82
66.24
63.50
HN
O
NH
N
Ph
12
f1 (ppm)

9. DFT computational calculation studies.

9.1 Computational method

All calculations were carried out with the GAUSSIAN 09 packages.[9] The conformations of intermediates and transition states were generated by GA Conf. search module in SYBYL-X 2.0.[10] The recently developed M06-2x functional,[11-16] together with the standard 6-31G(d) basis set, were used for optimizing the geometry of all the minima and transition states. All the optimized structures were confirmed by frequency calculations to be either minima or transition states using the same level of theory. For transition states, intrinsic reaction coordinate analysis (IRC) was done to verify that they connect the right reactants.[17] To take solvent effects into account, solution-phase single-point calculations were performed on the gas-phase geometries.[18] The solution-phase single point energy calculations were done using M06-2x method at a larger basis set 6-31++G(d,p). Solvent effect was accounted for using self-consistent reaction field (SCRF) method, using SMD model and UAKS radii.[19-20] Solution-phase single-point energies corrected by the gas-phase Gibbs free energy corrections were used to describe all the reaction energetics. All of these energies correspond to the reference state of 1 mol/L, 298 K. All energetics reported throughout the text are in kcal/mol, and the bond lengths are in angstroms (Å). Structures were generated using GaussView5.0.8 and CYLview.

9.2 DFT computational calculation studies for the key intermediates

9.2.1 Structure of **INT1-3b-2a**

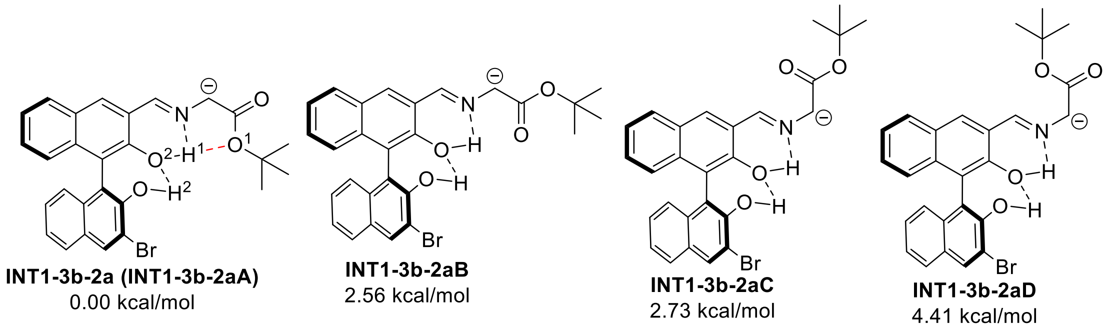

Supplementary Figure 1. Structures and energies of INT1-3b-2a with different conformations. Energies are obtained at the M06-2x/6-31++G(d, p) (mesitylene) level and are given in kcal/mol relative to **INT1-3b-2a**.

As reported in our pervious paper (JACS 2018), the conformations of the key intermediate formed by **2a** and catalyst **3b** were calculated. As shown in figure 1, different conformations was proposed and calculated as the ester group cloud rotate to different directions. The results showed that **INT1** (**INT1A**) employed the lowest energy, in which there are a special hydrogen bond

between the OH (H^1) and the O atom of ester group (O^1 in Supplementary Figure 1). Besides, the conjugation effect of the carbonyl group should be another factor for the lowest energy, since it shared a plane with C-N double bone and naphthalene. According to the initial computational explorations on the configuration of **1a**, both *Re*-face and *Si*-face attacks by different poses were considered.

9.2.2 Structure of **INT1-4f-2a**

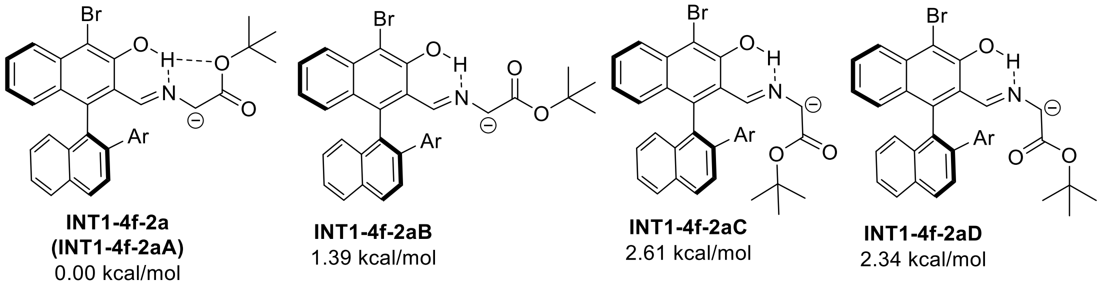

Supplementary Figure 2. Structures and energies of INT1-4f-2a with different conformations. Energies are obtained at the M06-2x/6-31++G(d, p) (toluene) level and are given in kcal/mol relative to **INT1-4f-2aA**.

We also explored the configuration of **INT1-4f** formed by **2a** and catalyst **4f**. Similarly, **INT1-4f-2a** employed the lowest energy due to the special hydrogen bond as **INT1-3b-2a**

9.2.3 Structure of **INT1-ent-3b-6a**

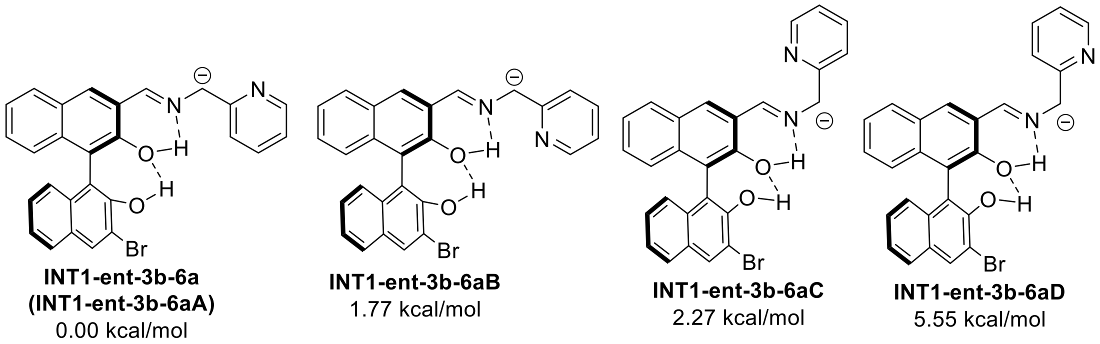

Supplementary Figure 3. Structures and energies of INT1-ent-3b-6a with different conformations. Energies are obtained at the M06-2x/6-31++G(d, p) (toluene) level and are given in kcal/mol relative to **INT1-ent-3b-6a**.

We explored the configurations of **INT1-ent-3b-6a** formed by **2a** and catalyst **3b**. Similarly, **INT1-ent-3b-6a** employed the lowest energy due to the conjugated plane. Interestingly, the rotation of pyridine ring would lead to 1.77 kcal/mol higher energy.

9.2.4. Structure of **INT1-4f-6a**

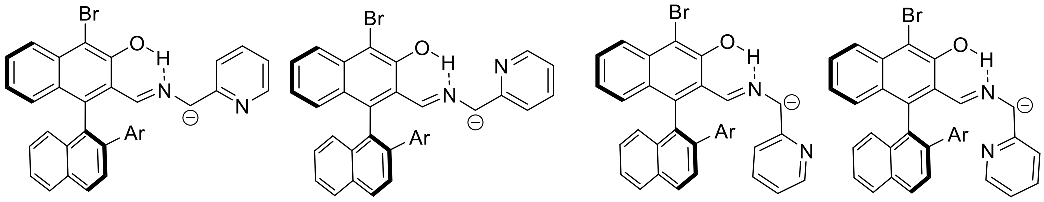

INT1-6a-4f
0.00 kcal/mol

INT1B-6a-4f
2.42 kcal/mol

INT1C-6a-4f
6.74 kcal/mol

INT1D-6a-4f
3.84 kcal/mol

Supplementary Figure 4. Structures and energies of INT1-4f-6a with different conformations. Energies are obtained at the M06-2x/6-31++G(d, p) (o-Xylene) level and are given in kcal/mol relative to **INT1-4f-6a**.

Similarly, the configurations of **INT1-6a-4f** formed by **6a** and catalyst **4f** was calculated, showing **INT1-6a-4f** employed the lowest energy (Supplementary Figure 4).

9.3 The structures and the energies of transition states (TSs) for nucleophilic attack

Since the chiral centre was formed with the formation of the C-C bond, transition states (TSs) for nucleophilic attack were individually investigated as critical process for stereoselectivity. Because both reactions could be catalysed by **3b** and **4f**, the transition states for the reactions using them as catalyst were calculated. The initial conformations of these transition structures were generated by SYBYL-X2.0 using GA.conf. module, and then calculated by Gaussian 09. The conformations and energies of INT1 were all considered when calculating the TSs. Various conformations (4-10) of transition structures was calculated to represent each of the four possibilities (RR, RS, SR, SS). The transition structure employing the lowest energy that leads to different stereoselective isomer was selected and add into the manuscript. Four transition structures with the lower energies to produce different configurations were listed following.

9.3.1 TSs to form **5a** by the 1,6-conjugate addition catalysed by **3b**

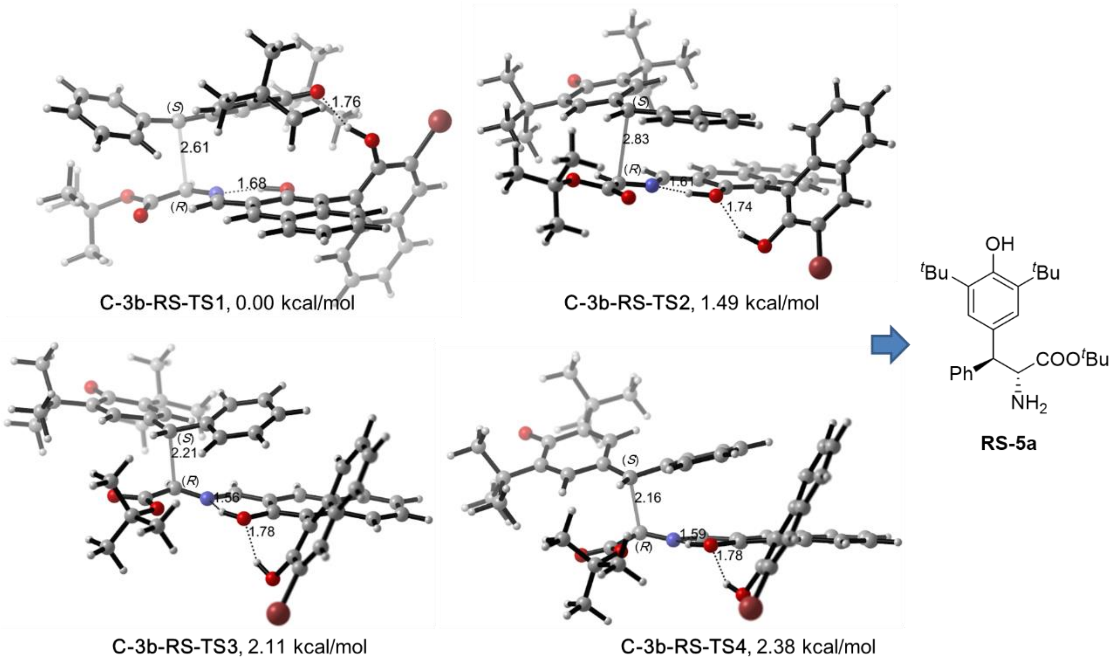

Supplementary Figure 5. Calculated transition states lead to RS-5a. The structures and energies of TSs to form **RS-5a** by the 1,6-conjugate addition catalysed by **3b** at the M06-2X/6-31(d) // M06-2X/6-31++G(d,p) level (toluene as solvent) and energies are given in kcal/mol relative energy of **C-3b-RS-TS1**.

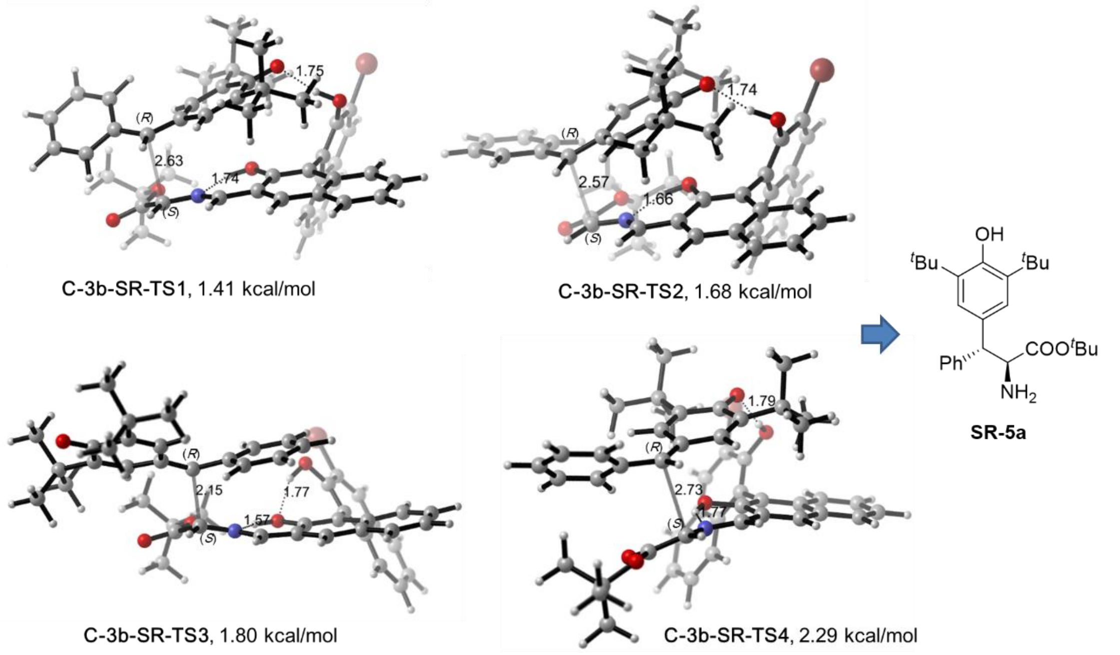

Supplementary Figure 6. The structures and energies of TSs to form **SR-5a** by the 1,6-conjugate addition catalysed by **3b** at the M06-2X/6-31(d) // M06-2X/6-31++G(d,p) level (toluene as solvent) and energies are given in kcal/mol relative energy of **C-3b-RS-TS1**.

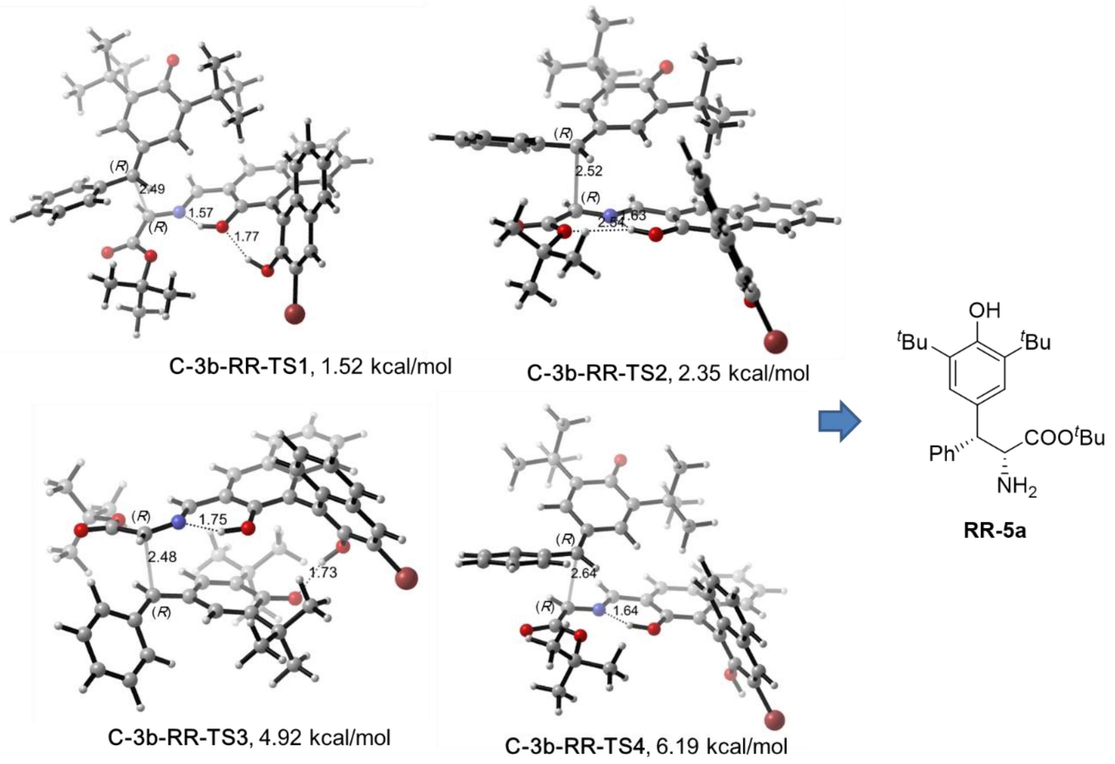

Supplementary Figure 7. The structures and energies of TSs to form **RR-5a** by the 1,6-conjugate addition catalysed by **3b** at the M06-2X/6-31(d) // M06-2X/6-31++G(d,p) level (toluene as solvent) and energies are given in kcal/mol relative energy of **C-3b-RS-TS1**.

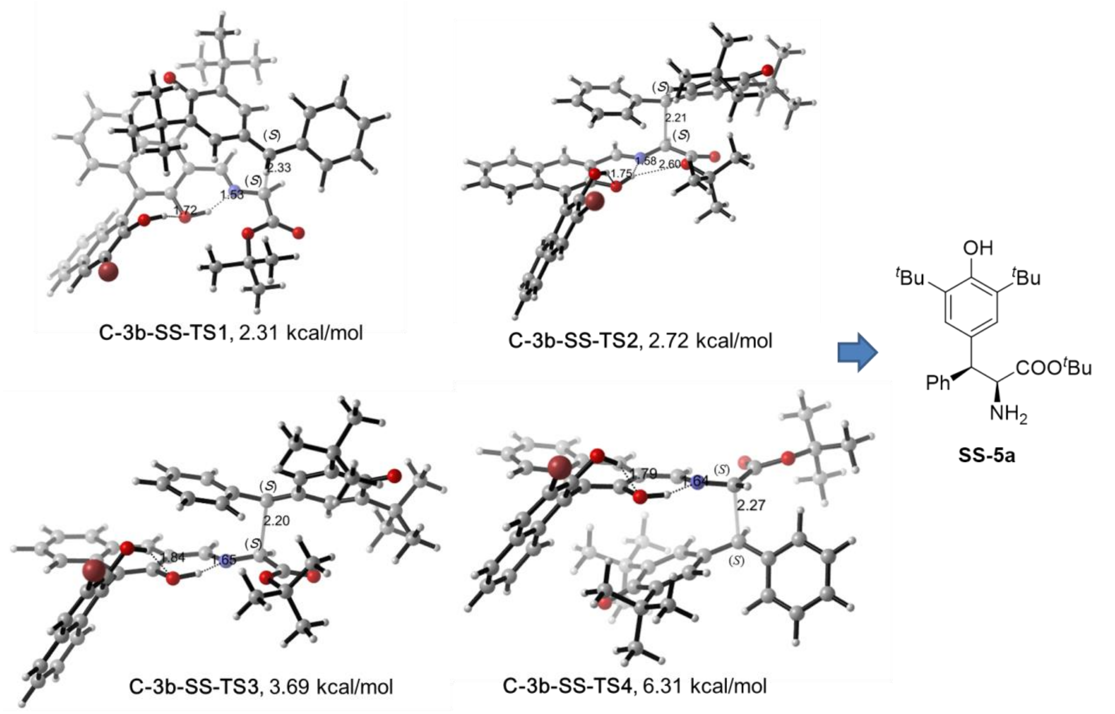

Supplementary Figure 8. The structures and energies of TSs to form **SS-5a** by the 1,6-conjugate addition catalysed by **3b** at the M06-2X/6-31(d) // M06-2X/6-31++G(d,p) level (toluene as solvent) and energies are given in kcal/mol relative energy of **C-3b-RS-TS1**.

10.3.2 TSs to form **5a** by the 1,6-conjugate addition catalysed by **4f**

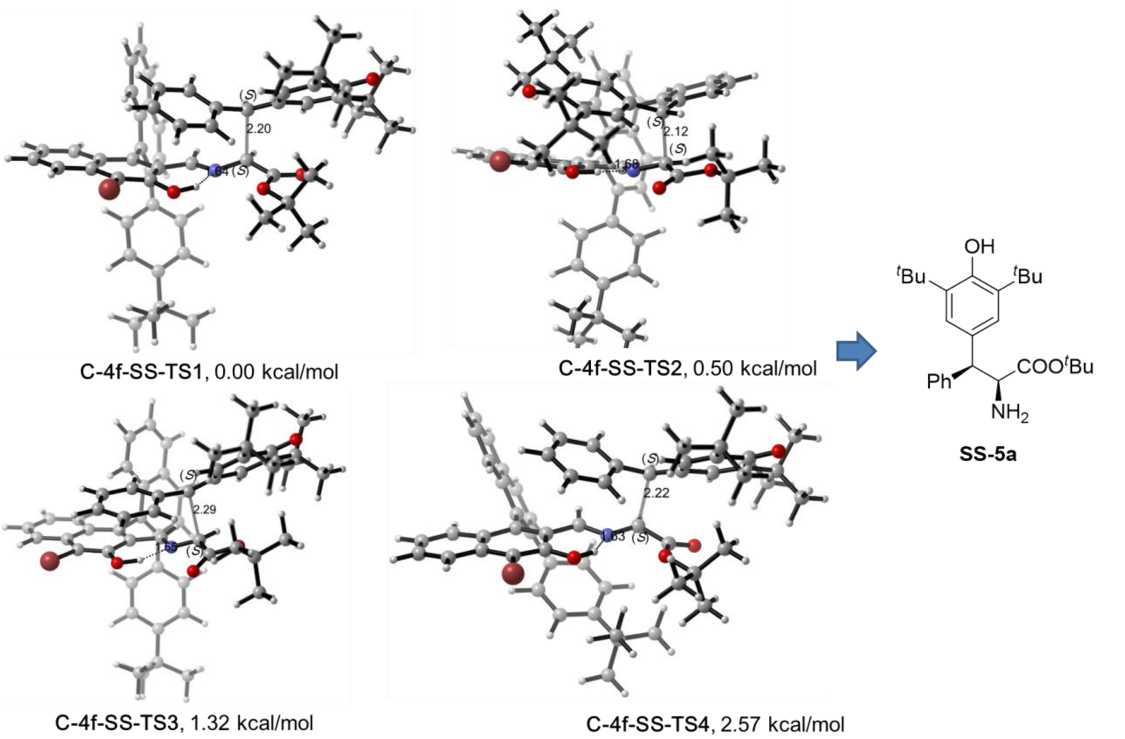

Supplementary Figure 9. The structures and energies of TSs to form **SS-5a** by the 1,6-conjugate addition catalysed by **4f** at the M06-2X/6-31(d) // M06-2X/6-31++G(d,p) level (toluene as solvent) and energies are given in kcal/mol relative energy of **C-4f-SS-TS1**.

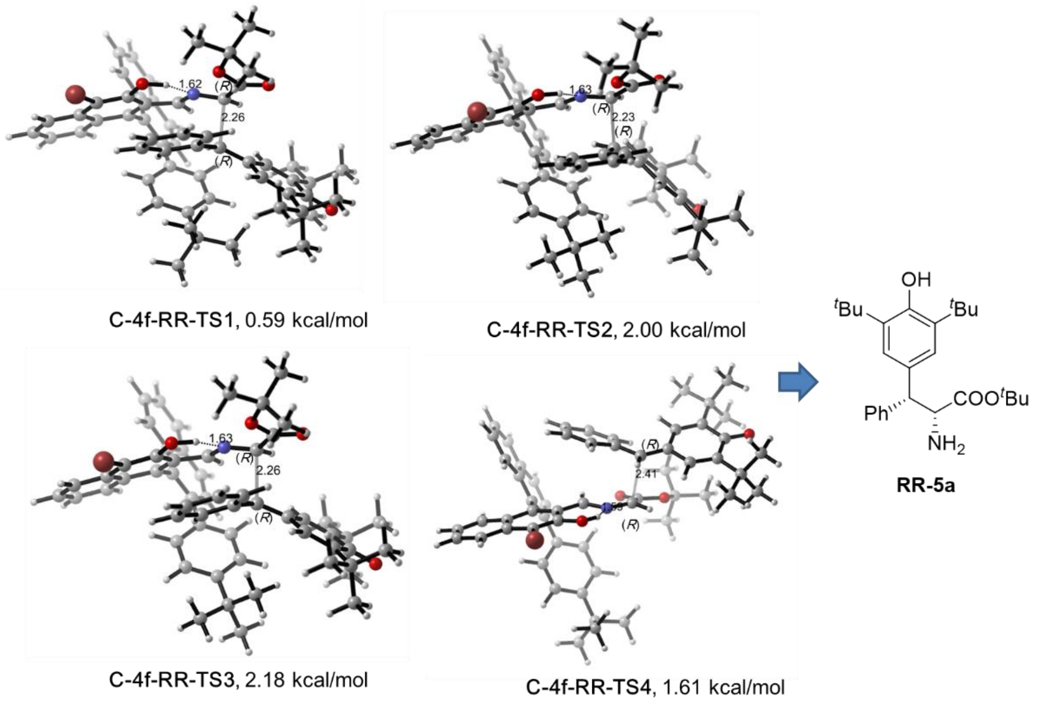

Supplementary Figure 10. The structures and energies of TSs to form **RR-5a** by the 1,6-conjugate addition catalysed by **4f** at the M06-2X/6-31(d) // M06-2X/6-31++G(d,p) level (toluene as solvent) and energies are given in kcal/mol relative energy of **C-4f-SS-TS1**.

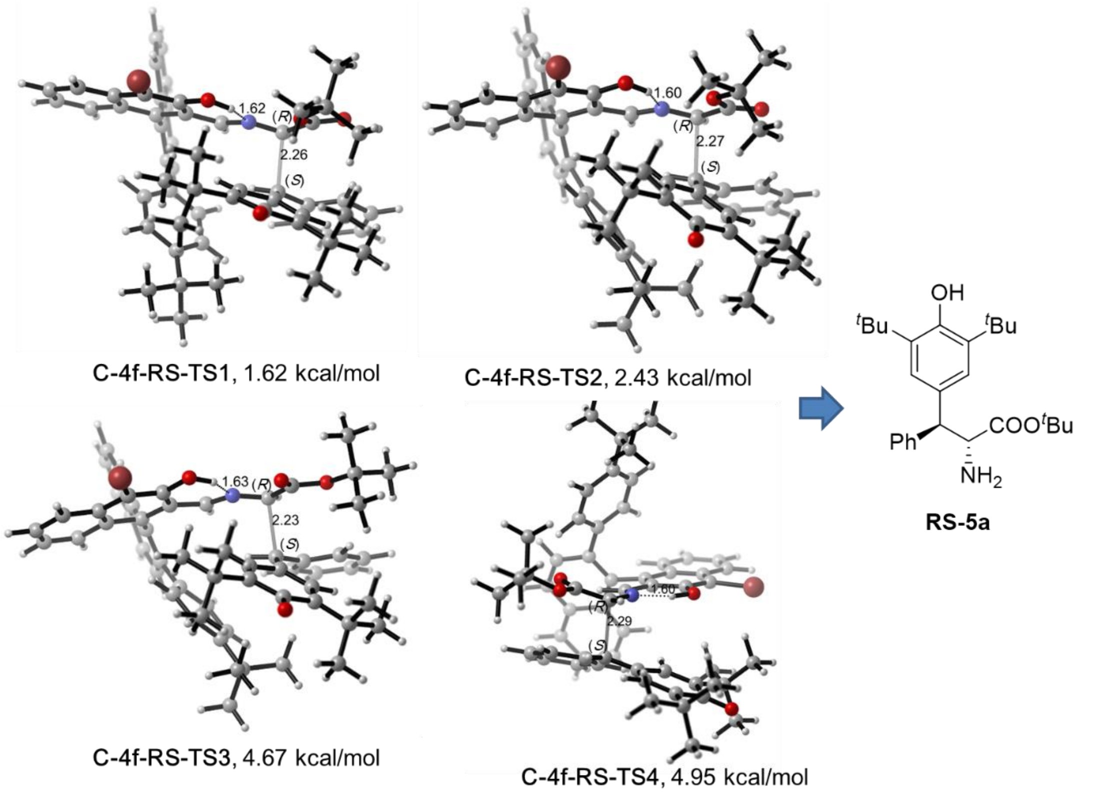

Supplementary Figure 11. The structures and energies of TSs to form **RS-5a** by the 1,6-conjugate addition catalysed by **4f** at the M06-2X/6-31(d) // M06-2X/6-31++G(d,p) level (toluene as solvent) and energies are given in kcal/mol relative energy of **C-4f-SS-TS1**.

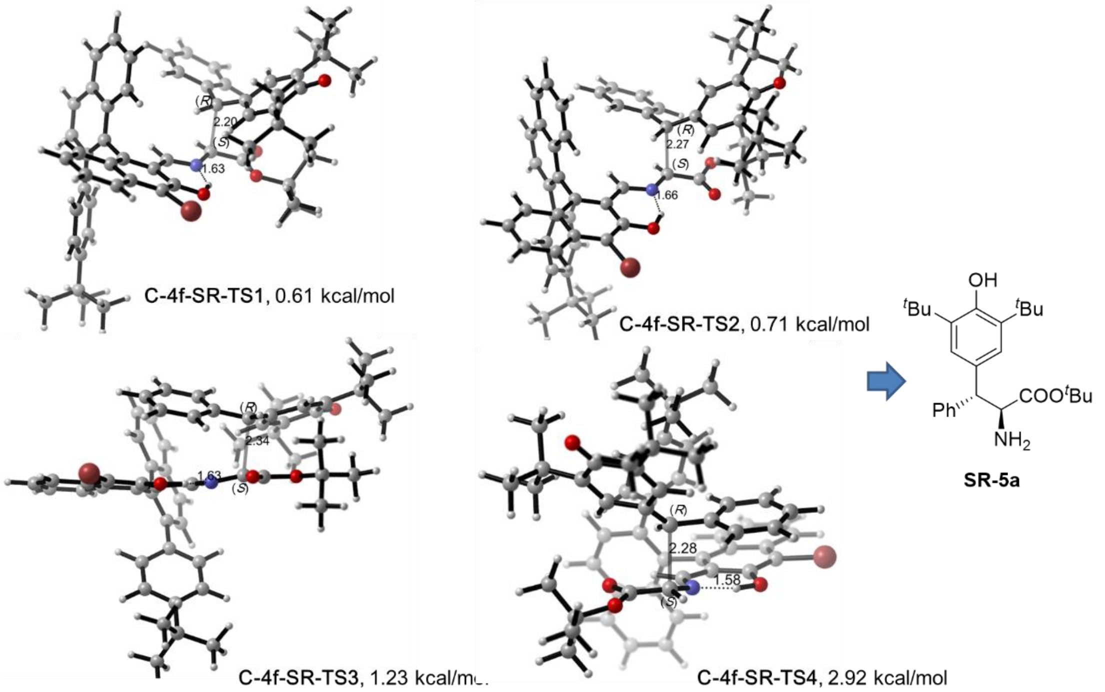

Supplementary Figure 12. The structures and energies of TSs to form **SR-5a** by the 1,6-conjugate addition catalysed by **4f** at the M06-2X/6-31(d) // M06-2X/6-31++G(d,p) level (toluene as solvent) and energies are given in kcal/mol relative energy of **C-4f-SS-TS1**.

10.3.3 TSs to form **8a** by Mannich reaction catalyzed by ***ent*-3b**

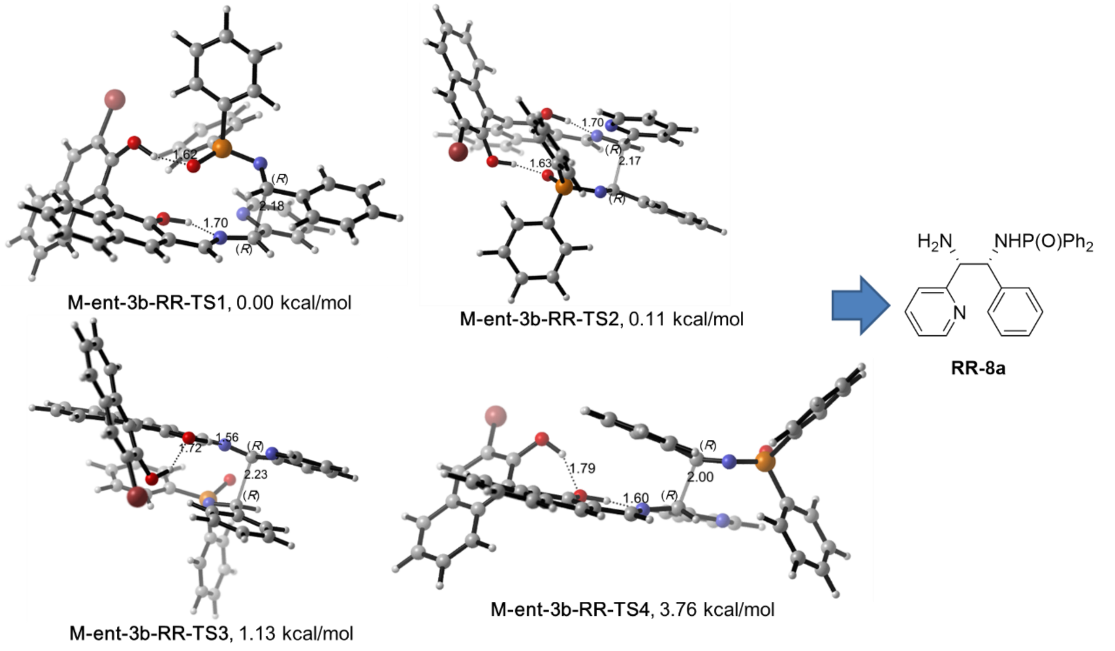

Supplementary Figure 13. The structures and energies of TSs to form **RR-8a** by Mannich reaction catalyzed by ***ent*-3b** at the M06-2X/6-31(d) // M06-2X/6-31++G(d,p) level (o-xylene as solvent) and energies are given in kcal/mol relative energy of **M-*ent*-3b-RR-TS1**.

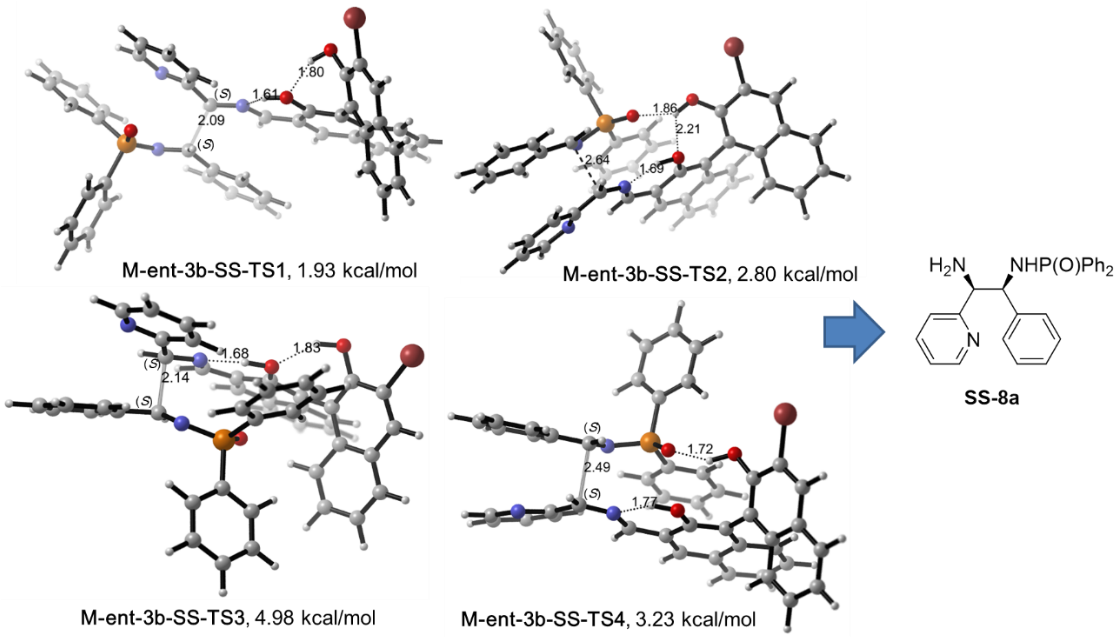

Supplementary Figure 14. The structures and energies of TSs to form **SS-8a** by Mannich reaction catalyzed by ***ent*-3b** at the M06-2X/6-31(d) // M06-2X/6-31++G(d,p) level (o-xylene as solvent) and energies are given in kcal/mol relative energy of **M-*ent*-3b-RR-TS1**.

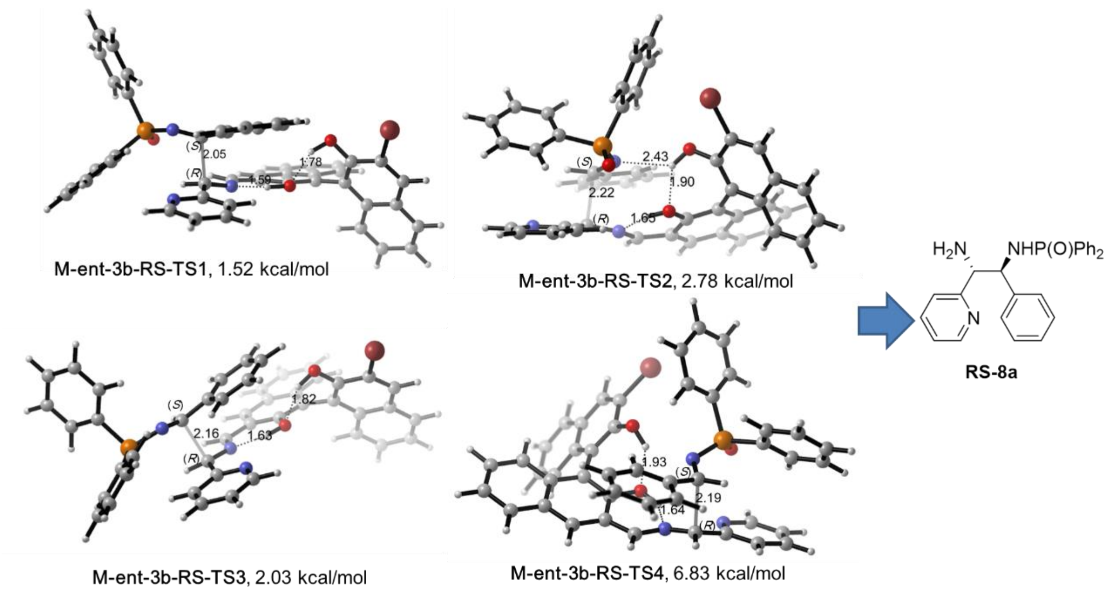

Supplementary Figure 15. The structures and energies of TSs to form **RS-8a** by Mannich reaction catalyzed by ***ent*-3b** at the M06-2X/6-31(d) // M06-2X/6-31++G(d,p) level (o-xylene as solvent) and energies are given in kcal/mol relative energy of **M-*ent*-3b-RR-TS1**.

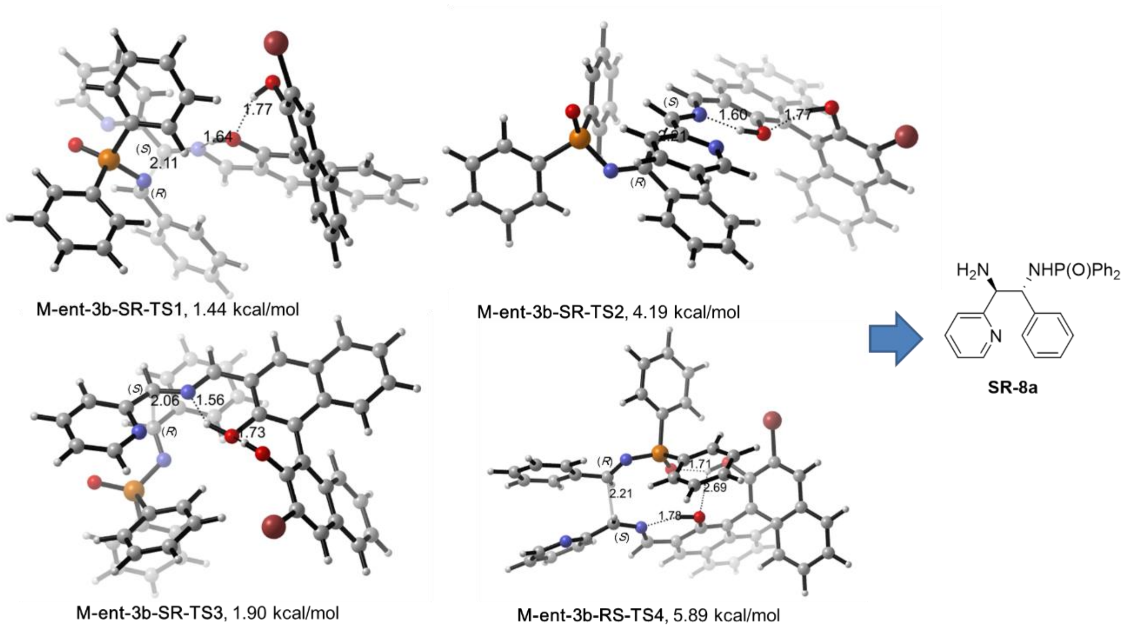

Supplementary Figure 16. The structures and energies of TSs to form **SR-8a** by Mannich reaction catalyzed by ***ent*-3b** at the M06-2X/6-31(d) // M06-2X/6-31++G(d,p) level (o-xylene as solvent) and energies are given in kcal/mol relative energy of **M-*ent*-3b-RR-TS1**.

10.3.4 TSs to form **8a** by Mannich reaction catalyzed by **4f**

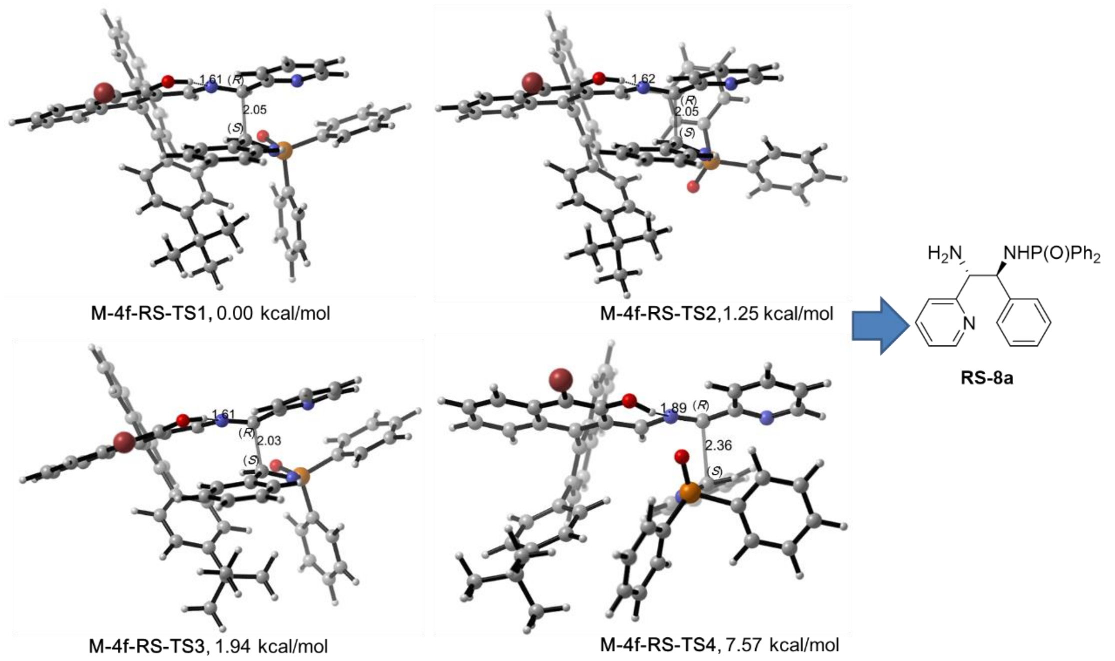

Supplementary Figure 17. The structures and energies of TSs to form **RS-8a** by Mannich reaction catalyzed by **4f** at the M06-2X/6-31(d) // M06-2X/6-31++G(d,p) level (DCM as solvent) and energies are given in kcal/mol relative energy of **M-4f-RS-TS1**.

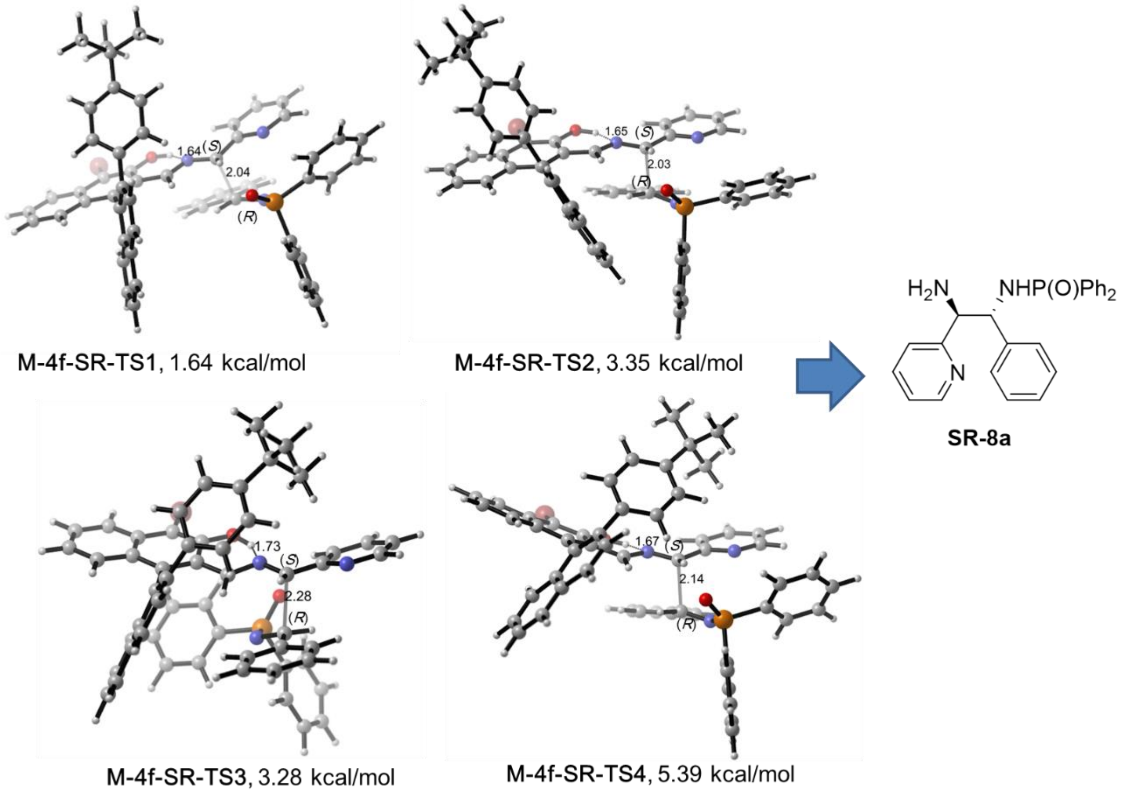

Supplementary Figure 18. The structures and energies of TSs to form **SR-8a** by Mannich reaction catalyzed by **4f** at the M06-2X/6-31(d) // M06-2X/6-31++G(d,p) level (DCM as solvent) and energies are given in kcal/mol relative energy of **M-4f-RS-TS1**.

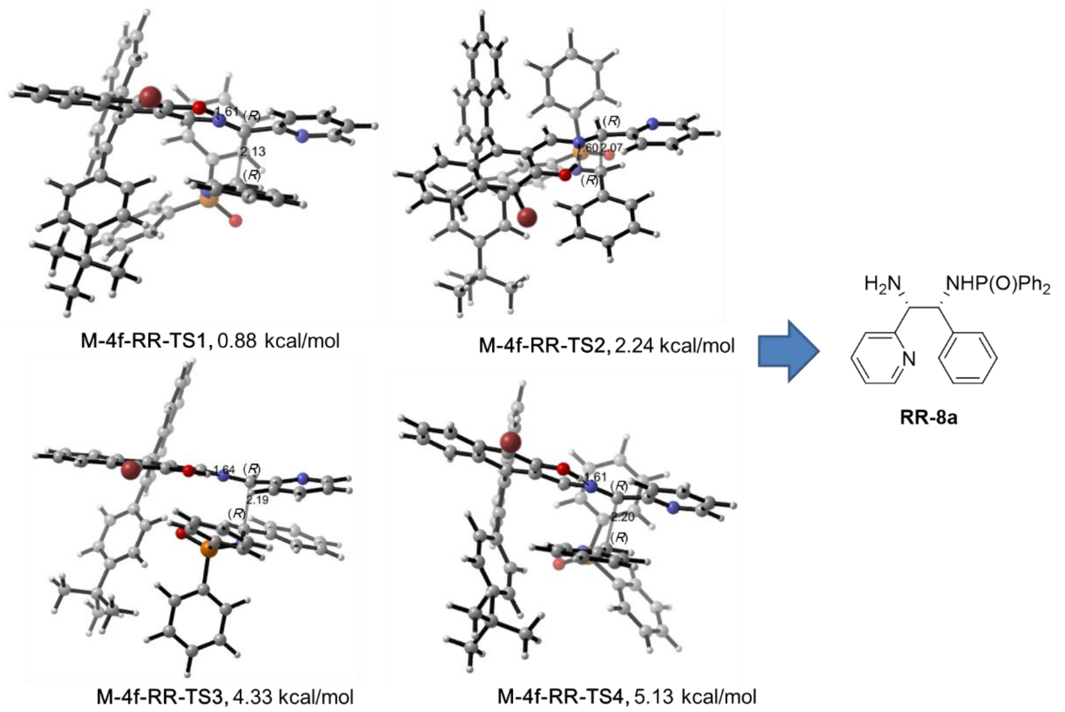

Supplementary Figure 19. The structures and energies of TSs to form **RR-8a** by Mannich reaction catalyzed by **4f** at the M06-2X/6-31(d) // M06-2X/6-31++G(d,p) level (DCM as solvent) and energies are given in kcal/mol relative energy of **M-4f-RS-TS1**.

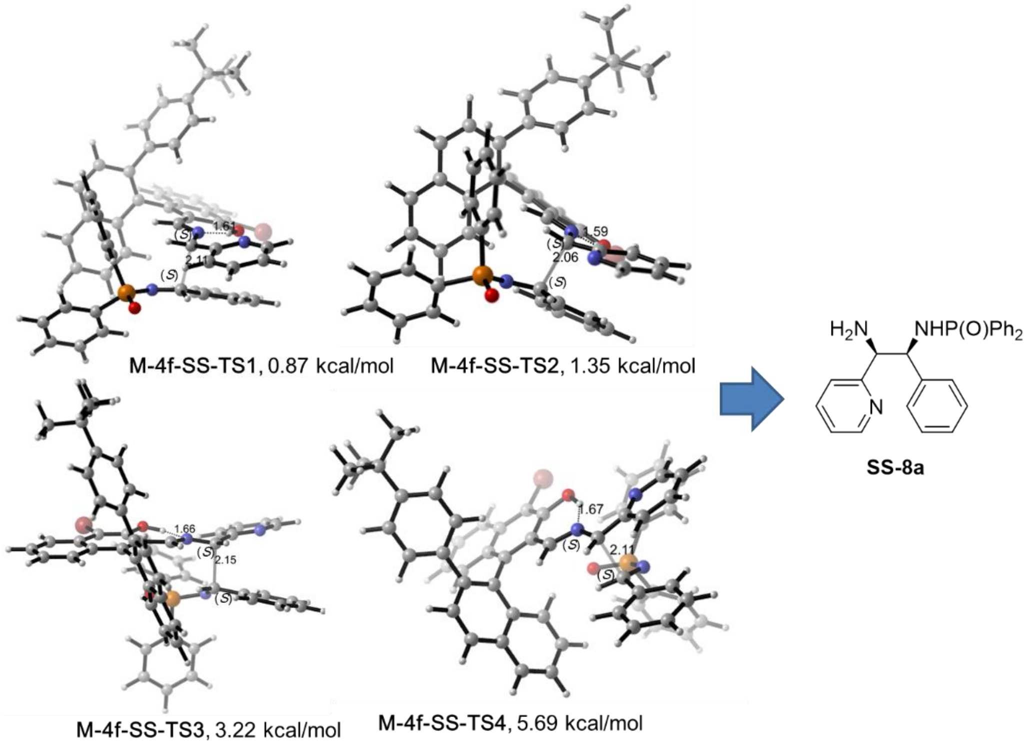

Supplementary Figure 20. The structures and energies of TSs to form **SS-8a** by Mannich reaction catalyzed by **4f** at the M06-2X/6-31(d) // M06-2X/6-31++G(d,p) level (DCM as solvent) and energies are given in kcal/mol relative energy of **M-4f-RS-TS1**.

10. Supplementary References

(1) Xu, B. *et al.* Catalytic asymmetric direct α-alkylation of amino esters by aldehydes via imine activation. *Chem. Sci.* **5**, 1988–1991 (2014).

(2) Sako, M. *et al.* Efficient enantioselective synthesis of oxahelicenes using redox/acid cooperative catalysts. *J. Am. Chem. Soc.* **138**, 11481−11484 (2016).

(3) Wen, W. *et al.* Chiral aldehyde catalysis for the catalytic asymmetric activation of glycine esters. *J. Am. Chem. Soc.* **140**, 9774−9780 (2018).

(4) Richter, D., Hampel, N., Singer, T., Ofial, A. R. & Mayr, H. Synthesis and characterization of novel quinone methides: reference electrophiles for the construction of nucleophilicity scales. *Eur. J. Org. Chem.* 3203–3211 (2009).

(5) Chu, W. *et al.* Asymmetric catalytic 1,6-conjugate addition/aromatization of *para*-quinone methides: enantioselective introduction of functionalized diarylmethine stereogenic centers. *Angew. Chem. Int. Ed.* **52**, 9229 –9233 (2013).

(6) Abell, J. P. & Yamamoto, H. Dual-activation asymmetric Strecker reaction of aldimines and ketimines catalyzed by a tethered bis(8-quinolinolato) aluminum complex. *J. Am. Chem. Soc.* **131**, 15118–15119 (2009).

(7) Le, P. Q., Nguyen, T. S. & May, J. A. A general method for the enantioselective synthesis of α-chiral heterocycles. *Org. Lett.* **14**, 6104–6107 (2012).

(8) Zhang, X.-Z. *et al.* Diastereoselective and enantioselective synthesis of unsymmetric β,β-diaryl-α-amino acid esters via organocatalytic 1,6-conjugate addition of *para*-quinone methides. *J. Org. Chem.* **81**, 5655−5662 (2016).

(9) Frisch, M. J., Trucks, G. W., Schlegel, H. B., Scuseria, G. E., Robb, M. A., Cheeseman, J. R., Scalmani, G., Barone, V., Mennucci, B., Petersson, G. A., Nakatsuji, H., Caricato, M., Li, X., Hratchian, H. P., Izmaylov, A. F., Bloino, J., Zheng, G., Sonnenberg, J. L., Hada, M., Ehara, M., Toyota, K., Fukuda, R., Hasegawa, J., Ishida, M., Nakajima, T., Honda, Y., Kitao, O., Nakai, H., Vreven, T., Montgomery Jr., J. A., Peralta, J. E., Ogliaro, F., Bearpark, M. J., Heyd, J., Brothers, E. N., Kudin, K. N., Staroverov, V. N., Kobayashi, R., Normand, J., Raghavachari, K., Rendell, A. P., Burant, J. C., Iyengar, S. S., Tomasi, J., Cossi, M., Rega, N., Millam, N. J., Klene, M., Knox, J. E., Cross, J. B., Bakken, V., Adamo, C., Jaramillo, J., Gomperts, R., Stratmann, R. E., Yazyev, O.,

Austin, A. J., Cammi, R., Pomelli, C., Ochterski, J. W., Martin, R. L., Morokuma, K., Zakrzewski, V. G., Voth, G. A., Salvador, P., Dannenberg, J. J., Dapprich, S., Daniels, A. D., Farkas, Ö., Foresman, J. B., Ortiz, J. V., Cioslowski, J., Fox, D. J. *Gaussian 09,* Gaussian, Inc.: Wallingford, CT, USA, (2009).

(10) Tripos International, (2012). Sybyl-X 2.0, Tripos International, St.Louis, MO, USA.

(11) Zhao, Y., Schultz, N. E. & Truhlar, D. G. Exchange-correlation functional with broad accuracy for metallic and nonmetallic compounds, kinetics, and noncovalent interactions. *J. Chem. Phys.* **123**, 161103 (2005).

(12) Zhao, Y., Schultz, N. E. & Truhlar, D. G. Design of density functionals by combining the method of constraint satisfaction with parametrization for thermochemistry, thermochemical kinetics, and noncovalent interactions. *J. Chem. Theory. Comput.* **2**, 364−382 (2006).

(13) Zhao, Y. & Truhlar, D. G. Assessment of model chemistries for noncovalent interactions. *J. Chem. Theory Comput.* **2**, 1009−1018 (2006).

(14) Zhao, Y. & Truhlar, D. G., Density functionals with broad applicability in chemistry. *Acc. Chem. Res.* **41**, 157−167 (2008).

(15) Zhao, Y. & Truhlar, D. G. The M06 suite of density functionals for main group thermochemistry, thermochemical kinetics, noncovalent interactions, excited states, and transition elements: two new functionals and systematic testing of four M06-class functionals and 12 other functionals. *Theor. Chem. Acc.* **120**, 215−241 (2008).

(16) Zhao, Y. & Truhlar, D. G. Applications and validations of the Minnesota density functionals. *Chem. Phys. Lett.* **502**, 1−13 (2011).

(17) Fukui, K. The path of chemical reactions−the IRC approach. *Acc. Chem. Res.* **14**, 363−368 (1981).

(18) Um, J. M., DiRocco, D. A., Noey, E. L., Rovis, T. & Houk, K. N. Quantum mechanical investigation of the effect of catalyst fluorination in the intermolecular asymmetric stetter reaction. *J. Am. Chem. Soc.* **133**, 11249−11254 (2011).

(19) Marenich, A. V., Cramer, C. J. & Truhlar, D. G. Universal solvation model based on solute electron density and on a continuum model of the solvent defined by the bulk dielectric constant and atomic surface tensions. *J. Phys. Chem. B* **113**, 6378−6396 (2009).

(20) Ribeiro, R. F., Marenich, A. V., Cramer, C. J. & Truhlar, D. G. Use of solution-phase vibrational frequencies in continuum models for the free energy of solvation. *J. Phys. Chem. B* **115**, 14556−14562 (2011).
