# Supplementary material for: Diastereodivergent chiral aldehyde catalysis for asymmetric 1,6-conjugated addition and Mannich reactions
Source: Nat Commun. 2020 Oct 23;11:5372. doi: 10.1038/s41467-020-19245-3 (PMC7584650; doi:10.1038/s41467-020-19245-3)
Supplement: Supplementary file 3 — Description of Additional Supplementary Files [file 41467_2020_19245_MOESM3_ESM.pdf]

**Description of Additional Supplementary Files**

File Name: Supplementary Data 1
Description: Computational data
