# Supplementary material for: Diastereodivergent chiral aldehyde catalysis for asymmetric 1,6-conjugated addition and Mannich reactions
Source: Nat Commun. 2020 Oct 23;11:5372. doi: 10.1038/s41467-020-19245-3 (PMC7584650; doi:10.1038/s41467-020-19245-3)
Supplement: Supplementary file 4 — Supplementary Data 1 [file 41467_2020_19245_MOESM4_ESM.zip › 252666_2_supp_4967061_qhg6yf.docx]

**Computational data**

**INT1-3b-2a**

Zero-point correction= 0.441337 (Hartree/Particle)

Thermal correction to Energy= 0.469764

Thermal correction to Enthalpy= 0.470708

Thermal correction to Gibbs Free Energy= 0.380687

E(solv) = -3969.85507042 A.U.

C -4.449153 -3.777316 -1.288546

C -4.694520 -2.388972 -1.223083

C -3.685071 -1.521137 -0.873818

C -2.381260 -1.986752 -0.570395

C -2.125439 -3.386790 -0.673456

C -3.190592 -4.257317 -1.023289

C -1.303102 -1.111814 -0.194601

C -0.029728 -1.629525 -0.053687

C 0.250954 -3.047416 -0.176832

C -0.817695 -3.877762 -0.452084

C -1.532448 0.345778 0.030667

C -2.487678 0.791151 1.004825

C -2.784102 2.177217 1.134838

C -2.084486 3.112241 0.333994

C -1.113533 2.678552 -0.520368

C -0.809543 1.296529 -0.682956

C -3.150878 -0.110104 1.884875

C -4.069628 0.335827 2.800657

C -4.386435 1.711419 2.896866

C -3.749028 2.609437 2.082040

O 0.985738 -0.774896 0.193193

C 1.598044 -3.545908 -0.018629

O 0.168219 0.978841 -1.551219

O 0.168219 0.978841 -1.551219

Br -0.116369 3.938744 -1.517688

N 2.570943 -2.689421 0.173030

C 3.874775 -2.874231 0.312786

H -5.250487 -4.459350 -1.559562

H -5.682556 -2.001164 -1.454543

H -3.882544 -0.454491 -0.838100

H -2.980976 -5.323087 -1.087530

H -0.642113 -4.948943 -0.539594

H -2.295714 4.172842 0.429318

H -2.908338 -1.165352 1.829427

H -4.553007 -0.374993 3.464601

H -5.119193 2.052169 3.622617

H -3.961313 3.673820 2.152937

H 1.857552 -1.355234 0.239988

H 1.774840 -4.623287 -0.094371

H 0.701963 0.267073 -1.118162

H 4.344005 -3.856225 0.294824

C 4.718470 -1.722203 0.457722

O 5.937534 -1.731905 0.588711

O 3.982196 -0.561445 0.423803

C 4.601950 0.727055 0.524583

C 3.418274 1.689265 0.429316

H 3.760266 2.725831 0.520349

H 2.899002 1.574820 -0.527359

H 2.697428 1.479174 1.225177

C 5.302901 0.883634 1.874813

H 4.592894 0.677874 2.682545

H 6.140625 0.189662 1.951689

H 5.667790 1.910939 1.987987

C 5.568040 0.956989 -0.638015

H 6.416705 0.275361 -0.571692

H 5.047642 0.788465 -1.586358

H 5.927812 1.992169 -0.621550

----------------------------------------------------

**INT1-3b-2aB**

Zero-point correction= 0.442914 (Hartree/Particle)

Thermal correction to Energy= 0.471289

Thermal correction to Enthalpy= 0.472233

Thermal correction to Gibbs Free Energy= 0.383257

E(solv) = -3969.85357218 A.U.

C -3.782102 -4.509082 -1.294314

C -4.389811 -3.247192 -1.117799

C -3.631031 -2.153312 -0.769138

C -2.230219 -2.255221 -0.575646

C -1.613672 -3.523454 -0.790416

C -2.424475 -4.635716 -1.137838

C -1.404998 -1.136207 -0.204140

C -0.032154 -1.288834 -0.170201

C 0.607901 -2.566845 -0.397418

C -0.210646 -3.644556 -0.674913

C -2.019092 0.185759 0.113673

C -2.987646 0.307314 1.164870

C -3.675079 1.537472 1.363580

C -3.359799 2.646361 0.540782

C -2.374041 2.537172 -0.395428

C -1.669995 1.318114 -0.612955

C -3.289427 -0.768397 2.046902

C -4.229365 -0.633167 3.036821

C -4.929408 0.584335 3.208757

C -4.650445 1.645865 2.388919

O 0.732353 -0.200477 0.070343

C 2.042161 -2.699832 -0.332086

O -0.704670 1.320168 -1.550064

Br -1.913983 4.041734 -1.444879

N 2.799098 -1.657931 -0.087954

C 4.125324 -1.647686 -0.018019

H -4.385616 -5.371176 -1.564997

H -5.460490 -3.135420 -1.263632

H -4.109130 -1.186636 -0.649459

H -1.938190 -5.597312 -1.288236

H 0.244276 -4.620064 -0.839450

H -3.881034 3.588652 0.677486

H -2.753402 -1.703728 1.929072

H -4.434449 -1.468759 3.699746

H -5.674205 0.677585 3.993787

H -5.163654 2.596602 2.513961

H 1.717465 -0.508421 0.078986

H 2.476386 -3.690510 -0.504411

H 0.049531 0.801963 -1.181021

H 4.716286 -2.550609 -0.164919

C 4.774725 -0.401951 0.241372

O 4.246061 0.688130 0.424641

O 6.146695 -0.569942 0.262483

C 7.000597 0.557192 0.492863

C 8.402398 -0.045695 0.416206

H 9.162227 0.726326 0.577157

H 8.520621 -0.822655 1.177757

H 8.562743 -0.500061 -0.566383

C 6.829595 1.613040 -0.601577

H 6.954010 1.146536 -1.584536

H 5.838248 2.062380 -0.546514

H 7.592987 2.391593 -0.488305

C 6.763961 1.148310 1.883959

H 5.766640 1.583269 1.947657

H 6.856932 0.360139 2.638661

H 7.514479 1.919396 2.093885

---------------------------------------------------

**INT1-3b-2aC**

Zero-point correction= 0.442472 (Hartree/Particle)

Thermal correction to Energy= 0.470854

Thermal correction to Enthalpy= 0.471798

Thermal correction to Gibbs Free Energy= 0.382738

E(solv) = -3969.85276442 A.U.

C 1.850942 4.896293 -1.678054

C 2.949354 4.044398 -1.432732

C 2.746327 2.755173 -0.996912

C 1.441172 2.245310 -0.778216

C 0.332215 3.097511 -1.058672

C 0.574326 4.424868 -1.498894

C 1.189957 0.908529 -0.309715

C -0.112293 0.450941 -0.247918

C -1.246204 1.304199 -0.522580

C -0.984020 2.604582 -0.901782

C 2.315567 0.023581 0.108133

C 3.207256 0.434350 1.153619

C 4.358999 -0.343602 1.459394

C 4.585270 -1.548078 0.748845

C 3.677394 -1.969025 -0.178850

C 2.516448 -1.208905 -0.500785

C 2.972919 1.603692 1.930401

C 3.841632 1.984288 2.920928

C 5.001652 1.222978 3.197722

C 5.247751 0.080958 2.481785

O -0.343491 -0.839364 0.076407

C -2.603504 0.821027 -0.373947

O 1.670191 -1.722565 -1.412528

Br 3.945998 -3.617583 -1.067789

N -2.811983 -0.426691 -0.020081

C -3.981033 -1.048702 0.202623

H 2.017125 5.915090 -2.017265

H 3.961284 4.405567 -1.593085

H 3.599706 2.107673 -0.822920

H -0.284077 5.062500 -1.699384

H -1.820792 3.270877 -1.103884

H 5.462238 -2.149467 0.967603

H 2.081661 2.189107 1.734735

H 3.632053 2.876614 3.503417

H 5.683868 1.537669 3.982078

H 6.123899 -0.528681 2.690939

H -1.392861 -0.934807 0.105516

H -3.424751 1.511593 -0.545425

H 0.757919 -1.524645 -1.094041

H -3.937482 -2.092449 0.486015

C -5.250000 -0.414990 0.129820

O -5.489508 0.758978 -0.156818

O -6.261654 -1.308672 0.442918

C -7.618291 -0.857860 0.515855

C -8.385100 -2.117809 0.914761

H -9.454948 -1.903169 1.009697

H -8.246406 -2.896970 0.158994

H -8.013469 -2.497070 1.871566

C -7.783480 0.213950 1.596364

H -7.382235 -0.157566 2.545245

H -7.247292 1.121268 1.317338

H -8.846448 0.443317 1.735663

C -8.111306 -0.360634 -0.845083

H -7.577476 0.544203 -1.134368

H -7.938275 -1.132642 -1.602250

H -9.187287 -0.154909 -0.799485

-----------------------------------------------------

**INT1-3b-2aD**

Zero-point correction= 0.442816 (Hartree/Particle)

Thermal correction to Energy= 0.471145

Thermal correction to Enthalpy= 0.472089

Thermal correction to Gibbs Free Energy= 0.383022

E(solv) = -3969.85038419 A.U.

C 0.870874 4.712042 -1.910425

C 2.103148 4.059561 -1.691000

C 2.134193 2.801910 -1.133487

C 0.943376 2.127475 -0.762970

C -0.302492 2.775152 -1.016111

C -0.300984 4.076379 -1.582874

C 0.935738 0.813442 -0.177684

C -0.271422 0.176948 0.034537

C -1.542263 0.816904 -0.220818

C -1.511350 2.103962 -0.719752

C 2.214544 0.121755 0.155908

C 3.151418 0.710155 1.067992

C 4.430604 0.115995 1.260554

C 4.750065 -1.076670 0.566342

C 3.815096 -1.667041 -0.232674

C 2.523569 -1.099576 -0.429743

C 2.845396 1.881653 1.816088

C 3.760095 2.433477 2.676513

C 5.039302 1.851569 2.843243

C 5.360724 0.714229 2.150399

O -0.273584 -1.100662 0.473895

C -2.792914 0.135468 0.035732

O 1.663706 -1.775240 -1.214011

Br 4.230948 -3.280934 -1.126565

N -2.781572 -1.089255 0.514586

C -3.768198 -1.931035 0.842685

H 0.851367 5.706961 -2.346854

H 3.032427 4.548707 -1.968943

H 3.088161 2.306812 -0.983850

H -1.260140 4.558031 -1.761024

H -2.453714 2.612620 -0.916698

H 5.727749 -1.531494 0.692009

H 1.864857 2.330708 1.701375

H 3.497065 3.323351 3.240831

H 5.755289 2.299458 3.526183

H 6.332899 0.242921 2.275334

H -1.289525 -1.348167 0.591999

H -3.714449 0.669755 -0.179882

H 0.776313 -1.699697 -0.791084

H -3.466976 -2.898120 1.227646

C -5.183296 -1.744458 0.759740

O -6.026365 -2.573706 1.093174

O -5.534256 -0.513090 0.252632

C -6.915273 -0.161124 0.087723

C -6.844549 1.249465 -0.494586

H -7.850615 1.644000 -0.672328

H -6.320425 1.915963 0.197694

H -6.296395 1.237531 -1.441868

C -7.605024 -1.100387 -0.902875

H -7.029967 -1.137925 -1.833951

H -7.674556 -2.106174 -0.488845

H -8.609856 -0.726028 -1.131008

C -7.636575 -0.135467 1.435893

H -7.699143 -1.141064 1.851824

H -7.086318 0.504694 2.133453

H -8.645062 0.275495 1.310030

----------------------------------------------------

**INT1-4f-2a**

Zero-point correction= 0.633625 (Hartree/Particle)

Thermal correction to Energy= 0.671650

Thermal correction to Enthalpy= 0.672594

Thermal correction to Gibbs Free Energy= 0.560470

E(solv) = -4282.81743300 A.U.

C -2.277813 -0.691994 -2.608880

N -1.551717 -0.855973 -1.516324

C -0.265291 -0.599965 -1.402187

C 0.417050 -0.814776 -0.153156

C -0.300757 -1.397486 0.975446

C 0.371576 -1.634708 2.152379

C 1.744416 -1.314841 2.349099

C 2.443241 -0.722567 1.253198

C 1.763528 -0.490133 0.021061

C 2.511988 0.131233 -1.113600

C 3.445611 -0.655616 -1.866884

C 4.137670 -0.080079 -2.969740

C 3.873534 1.268175 -3.319164

C 2.969530 2.001028 -2.601467

C 2.284666 1.447838 -1.484431

C 2.436983 -1.540663 3.561183

C 3.759676 -1.188268 3.707100

C 4.449899 -0.586518 2.637208

C 3.804588 -0.362132 1.443413

C 3.699266 -2.019221 -1.550036

C 4.599447 -2.756748 -2.276339

C 5.295621 -2.175795 -3.362927

C 5.065452 -0.867807 -3.700344

O -1.589679 -1.711596 0.875151

Br -0.614516 -2.440140 3.563935

H -1.851991 -0.316579 -3.539704

H 0.301274 -0.223481 -2.256638

H 4.399885 1.707311 -4.163426

H 2.770270 3.036818 -2.863681

H 1.899891 -1.998494 4.384522

H 4.266600 -1.371095 4.650357

H 5.491480 -0.299381 2.751838

H 4.338797 0.103853 0.620413

H 3.161976 -2.466773 -0.720288

H 4.777964 -3.796561 -2.018761

H 6.005536 -2.771051 -3.930242

H 5.586214 -0.409688 -4.538222

H -1.892915 -1.443746 -0.058883

C 1.303377 2.311367 -0.775612

C 0.307229 2.968115 -1.496862

C 1.352939 2.505391 0.609262

C -0.618306 3.792159 -0.859986

H 0.234692 2.806812 -2.569838

C 0.432414 3.331515 1.237001

H 2.119104 2.002398 1.192611

C -0.575712 3.991530 0.520138

H -1.388183 4.264942 -1.460245

H 0.498766 3.458541 2.314855

C -1.577556 4.877003 1.264062

C -0.825619 6.017556 1.969951

H -1.529482 6.656592 2.516961

H -0.093144 5.631752 2.685747

H -0.290683 6.636765 1.241659

C -2.612947 5.496053 0.320048

H -2.138763 6.131494 -0.436067

H -3.196530 4.725425 -0.194050

H -3.308001 6.118749 0.893989

C -2.323422 4.033081 2.310987

H -2.854113 3.204574 1.831848

H -1.635473 3.606503 3.047053

H -3.053316 4.651670 2.847236

C -3.676576 -1.008543 -2.617924

O -4.114888 -1.453821 -1.397033

O -4.419745 -0.902457 -3.588864

C -5.478842 -1.850695 -1.208413

C -5.511763 -2.267840 0.260393

H -5.234174 -1.422247 0.896373

H -6.513151 -2.612894 0.539768

H -4.791165 -3.071207 0.438432

C -5.828180 -3.041475 -2.102743

H -5.099897 -3.843672 -1.944828

H -6.823630 -3.421034 -1.844445

H -5.811816 -2.748476 -3.152887

C -6.429578 -0.675388 -1.442606

H -7.448141 -0.958717 -1.152526

H -6.119011 0.175225 -0.827000

H -6.418318 -0.377332 -2.491161

---------------------------------------------------

**INT1-4f-2aB**

Zero-point correction= 0.634129 (Hartree/Particle)

Thermal correction to Energy= 0.672202

Thermal correction to Enthalpy= 0.673146

Thermal correction to Gibbs Free Energy= 0.561086

E(solv) = -4282.81582824 A.U.

C -2.967140 -1.491066 -0.053065

N -1.862094 -1.018277 0.501646

C -0.657594 -1.117775 -0.018529

C 0.490519 -0.572757 0.657796

C 0.331655 0.063638 1.959245

C 1.439385 0.557560 2.610012

C 2.754391 0.497969 2.070604

C 2.911457 -0.111781 0.788248

C 1.772051 -0.635880 0.108785

C 1.959353 -1.266244 -1.233935

C 2.512642 -2.586345 -1.325155

C 2.678226 -3.205644 -2.596201

C 2.267489 -2.506281 -3.758891

C 1.722278 -1.256612 -3.655047

C 1.568937 -0.615250 -2.394461

C 3.895638 1.014467 2.727374

C 5.143862 0.951373 2.151197

C 5.304035 0.365410 0.880384

C 4.213653 -0.151502 0.220480

C 2.897063 -3.314699 -0.165335

C 3.429654 -4.574563 -0.267648

C 3.608125 -5.179580 -1.534754

C 3.237417 -4.508327 -2.670546

O -0.864451 0.167556 2.528569

Br 1.156750 1.350731 4.312848

H -2.953282 -2.012435 -1.010193

H -0.511243 -1.624197 -0.975251

H 2.392702 -2.979062 -4.730230

H 1.412005 -0.717406 -4.546361

H 3.766340 1.469841 3.702933

H 6.003409 1.357849 2.676799

H 6.287188 0.318706 0.420166

H 4.339728 -0.601333 -0.760128

H 2.755811 -2.852525 0.806152

H 3.714066 -5.114933 0.630323

H 4.032822 -6.177141 -1.602983

H 3.360248 -4.964228 -3.650466

H -1.544588 -0.246141 1.899303

C 0.946312 0.734693 -2.367656

C -0.276207 0.953483 -3.001411

C 1.557518 1.816052 -1.722952

C -0.880946 2.208811 -2.985741

H -0.779318 0.120655 -3.487067

C 0.952890 3.063880 -1.714027

H 2.512752 1.672594 -1.226159

C -0.281789 3.290033 -2.338886

H -1.843038 2.324367 -3.473194

H 1.453835 3.878726 -1.197117

C -0.921312 4.678336 -2.269275

C 0.032407 5.717075 -2.882222

H -0.412820 6.718002 -2.829912

H 0.988929 5.746372 -2.351709

H 0.236613 5.483872 -3.932822

C -2.249896 4.741908 -3.029256

H -2.116774 4.507099 -4.091177

H -2.984713 4.046529 -2.610976

H -2.665831 5.752924 -2.956876

C -1.189347 5.037018 -0.798044

H -1.862455 4.306084 -0.339443

H -0.264570 5.049803 -0.213719

H -1.651372 6.029317 -0.727024

C -4.210178 -1.309283 0.634270

O -5.242714 -1.870286 -0.093869

O -4.395727 -0.750147 1.706847

C -6.584999 -1.805277 0.402355

C -7.390853 -2.532297 -0.672989

H -7.038368 -3.563387 -0.774018

H -8.454605 -2.545633 -0.412755

H -7.268799 -2.031547 -1.638310

C -7.057425 -0.354268 0.514790

H -6.909682 0.154654 -0.443695

H -8.125548 -0.327346 0.760309

H -6.492882 0.171094 1.285042

C -6.717272 -2.543280 1.736297

H -7.773813 -2.607838 2.021962

H -6.323802 -3.560123 1.633660

H -6.159402 -2.025820 2.516493

-------------------------------------------------

**INT1-4f-2aC**

Zero-point correction= 0.633519 (Hartree/Particle)

Thermal correction to Energy= 0.671561

Thermal correction to Enthalpy= 0.672505

Thermal correction to Gibbs Free Energy= 0.561117

E(solv) = -4282.81391687 A.U.

C -1.736230 1.718169 2.857199

N -0.723660 1.022483 2.339833

C -0.687954 0.127645 1.373315

C 0.573454 -0.454769 0.970920

C 1.794249 -0.138965 1.697164

C 2.983924 -0.714788 1.315594

C 3.097873 -1.604985 0.212833

C 1.906706 -1.901725 -0.517956

C 0.664756 -1.318572 -0.119865

C -0.553661 -1.610636 -0.935785

C -1.194945 -2.888536 -0.845646

C -2.337486 -3.170554 -1.647626

C -2.833169 -2.165105 -2.515583

C -2.227938 -0.938435 -2.566503

C -1.081536 -0.646523 -1.780102

C 4.319022 -2.189505 -0.198236

C 4.380892 -3.025382 -1.289267

C 3.214032 -3.309387 -2.025264

C 2.012204 -2.758894 -1.646846

C -0.723295 -3.897996 0.038512

C -1.344286 -5.118337 0.110768

C -2.473274 -5.399562 -0.695815

C -2.956993 -4.445062 -1.551944

O 1.756830 0.698930 2.728614

Br 4.523459 -0.262034 2.332168

H -1.492736 2.387308 3.675167

H -1.584617 -0.178658 0.844571

H -3.706560 -2.380888 -3.126704

H -2.612967 -0.160958 -3.221787

H 5.215496 -1.958146 0.366313

H 5.331792 -3.459790 -1.585020

H 3.260176 -3.961648 -2.892918

H 1.116557 -2.978780 -2.219905

H 0.139298 -3.678672 0.659401

H -0.970528 -5.874487 0.794768

H -2.956529 -6.370188 -0.628222

H -3.828510 -4.645570 -2.171172

H 0.782919 1.015586 2.788996

C -0.485338 0.715543 -1.851621

C -1.214359 1.819937 -1.414648

C 0.821360 0.915222 -2.306252

C -0.651977 3.095411 -1.426760

H -2.206571 1.672027 -0.992971

C 1.372989 2.187982 -2.320494

H 1.410485 0.060127 -2.628115

C 0.652573 3.305358 -1.875529

H -1.244175 3.921359 -1.048089

H 2.396015 2.308549 -2.668400

C 1.323485 4.681016 -1.868970

C 1.741863 5.055587 -3.300275

H 2.230345 6.037701 -3.310163

H 2.443261 4.325885 -3.716460

H 0.868155 5.097863 -3.959568

C 0.392024 5.775046 -1.338197

H -0.510828 5.866773 -1.951865

H 0.087684 5.574406 -0.305891

H 0.911155 6.739823 -1.355478

C 2.570116 4.631870 -0.969282

H 2.298151 4.348204 0.052097

H 3.299618 3.902559 -1.333757

H 3.057110 5.614440 -0.941213

C -3.126994 1.714056 2.511482

O -3.422573 0.901045 1.441146

O -3.992812 2.365227 3.090018

C -4.790550 0.669482 1.064341

C -4.667217 -0.306846 -0.101843

H -4.097477 -1.192997 0.197627

H -5.658048 -0.621353 -0.448023

H -4.139495 0.162099 -0.937553

C -5.458871 1.962797 0.595331

H -4.849342 2.428511 -0.187151

H -6.445013 1.737046 0.172535

H -5.563789 2.662336 1.424395

C -5.561960 0.018899 2.213478

H -6.560919 -0.272840 1.869749

H -5.033829 -0.880866 2.545471

H -5.654223 0.708718 3.052649

-----------------------------------------------------

**INT1-4f-2aD**

Zero-point correction= 0.633796 (Hartree/Particle)

Thermal correction to Energy= 0.671761

Thermal correction to Enthalpy= 0.672705

Thermal correction to Gibbs Free Energy= 0.561989

E(solv) = -4282.81522871 A.U.

C -2.506838 0.441190 2.274503

N -1.187701 0.430310 2.030775

C -0.530819 -0.375073 1.226936

C 0.879219 -0.156480 0.970916

C 1.605629 0.863950 1.707479

C 2.940442 1.072095 1.444266

C 3.657667 0.346818 0.454332

C 2.940283 -0.638989 -0.290043

C 1.559583 -0.876725 -0.009190

C 0.815969 -1.887082 -0.822956

C 1.061917 -3.286917 -0.635229

C 0.336110 -4.244349 -1.398926

C -0.646124 -3.792142 -2.315524

C -0.886947 -2.454321 -2.463738

C -0.150106 -1.484608 -1.732341

C 5.025682 0.564953 0.162082

C 5.665657 -0.139447 -0.830453

C 4.958194 -1.095352 -1.586003

C 3.630881 -1.333911 -1.319804

C 2.012388 -3.762311 0.310980

C 2.241409 -5.105341 0.469091

C 1.531421 -6.052538 -0.306417

C 0.598536 -5.627480 -1.215575

O 0.983367 1.587730 2.633245

Br 3.820292 2.423146 2.449872

H -2.868660 1.174902 2.984237

H -1.038695 -1.179806 0.707171

H -1.208338 -4.525484 -2.888852

H -1.645823 -2.103383 -3.157677

H 5.563580 1.310322 0.737211

H 6.715754 0.049299 -1.035524

H 5.458593 -1.642707 -2.379867

H 3.086953 -2.068085 -1.906460

H 2.555030 -3.038696 0.910499

H 2.970654 -5.446907 1.197870

H 1.722411 -7.113563 -0.172282

H 0.035517 -6.343425 -1.810273

H -0.001621 1.306075 2.588503

C -0.487998 -0.048297 -1.921970

C -1.784842 0.393197 -1.660934

C 0.470811 0.887234 -2.320887

C -2.113402 1.742260 -1.782766

H -2.520504 -0.312065 -1.280683

C 0.133159 2.227533 -2.446242

H 1.489310 0.561297 -2.517481

C -1.162574 2.687109 -2.171559

H -3.125519 2.045804 -1.536313

H 0.904585 2.932960 -2.746772

C -1.466411 4.183979 -2.270922

C -1.171356 4.677397 -3.696753

H -1.374869 5.752212 -3.778899

H -0.124960 4.510585 -3.969760

H -1.797641 4.150453 -4.424816

C -2.929638 4.500899 -1.947277

H -3.613119 3.992021 -2.635884

H -3.186450 4.204100 -0.925370

H -3.099700 5.579625 -2.038379

C -0.576878 4.944587 -1.273201

H -0.761952 4.598169 -0.251565

H 0.485164 4.792279 -1.487579

H -0.783761 6.020966 -1.321078

C -3.464678 -0.376093 1.617003

O -4.744104 -0.069804 2.050952

O -3.267001 -1.241929 0.759695

C -5.875536 -0.737955 1.483923

C -7.057714 -0.096082 2.209142

H -7.074099 0.981702 2.020077

H -8.002350 -0.530385 1.864850

H -6.967090 -0.254496 3.288038

C -5.837497 -2.238920 1.779039

H -5.730025 -2.398489 2.857164

H -6.773168 -2.706820 1.450736

H -4.996571 -2.706842 1.267154

C -5.984743 -0.464670 -0.018701

H -6.950548 -0.822519 -0.394305

H -5.921833 0.613929 -0.199855

H -5.179789 -0.962003 -0.559627

-----------------------------------------------

**INT1-ent-3b-6a**

Zero-point correction= 0.383378 (Hartree/Particle)

Thermal correction to Energy= 0.408107

Thermal correction to Enthalpy= 0.409051

Thermal correction to Gibbs Free Energy= 0.327309

E(solv) = -3871.16123489 A.U.

C -3.186206 4.592346 -1.291504

C -3.828625 3.345571 -1.135874

C -3.099776 2.230942 -0.785184

C -1.701928 2.298106 -0.570439

C -1.046729 3.551803 -0.763919

C -1.828321 4.685955 -1.113115

C -0.908058 1.155047 -0.198050

C 0.463665 1.278304 -0.141083

C 1.148927 2.537470 -0.345825

C 0.355131 3.639546 -0.626376

C -1.554351 -0.156618 0.097739

C -2.537259 -0.268855 1.136548

C -3.248379 -1.488012 1.318391

C -2.942646 -2.596809 0.491837

C -1.945236 -2.497671 -0.433207

C -1.219805 -1.289022 -0.635139

C -2.829764 0.806236 2.022521

C -3.782838 0.680497 3.000991

C -4.505782 -0.525844 3.156570

C -4.236873 -1.586434 2.332259

O 1.209264 0.169693 0.097355

C 2.576205 2.627664 -0.255796

O -0.245730 -1.302224 -1.564567

Br -1.495377 -4.003743 -1.485881

N 3.325710 1.564946 0.003074

C 4.644706 1.575393 0.090931

C 5.403812 0.394239 0.366677

C 4.805232 -0.874881 0.588564

C 5.604555 -1.968942 0.840880

C 6.995618 -1.816154 0.878165

C 7.494453 -0.536891 0.654558

N 6.754233 0.538955 0.407588

H -3.763960 5.471775 -1.563265

H -4.899312 3.261423 -1.298235

H -3.602399 1.274817 -0.679742

H -1.318454 5.637558 -1.248001

H 0.834035 4.605543 -0.776739

H -3.480695 -3.531264 0.616763

H -2.277265 1.733256 1.915675

H -3.981175 1.515057 3.667215

H -5.260661 -0.611432 3.932803

H -4.768643 -2.528517 2.444569

H 2.189085 0.487198 0.117971

H 3.042172 3.604509 -0.414898

H 0.505084 -0.782162 -1.195907

H 5.203579 2.501907 -0.054110

H 3.725486 -0.973505 0.560264

H 5.151162 -2.943088 1.009161

H 7.661485 -2.650002 1.072982

H 8.572426 -0.367050 0.675671

--------------------------------------------------

**INT1-ent-3b-6aB**

Zero-point correction= 0.383336 (Hartree/Particle)

Thermal correction to Energy= 0.408005

Thermal correction to Enthalpy= 0.408949

Thermal correction to Gibbs Free Energy= 0.327363

E(solv) = -3871.15846989 A.U.

C -3.265738 4.533123 -1.296542

C -3.872129 3.267826 -1.144158

C -3.113446 2.173074 -0.794769

C -1.717543 2.277951 -0.576944

C -1.100486 3.550937 -0.766537

C -1.911215 4.663428 -1.115530

C -0.892976 1.157665 -0.204185

C 0.478320 1.312628 -0.147048

C 1.119628 2.597726 -0.342978

C 0.298220 3.676419 -0.622559

C -1.508434 -0.167958 0.095254

C -2.489849 -0.297933 1.133495

C -3.177344 -1.530391 1.317325

C -2.850430 -2.633629 0.491711

C -1.852992 -2.516736 -0.431312

C -1.147340 -1.295913 -0.632652

C -2.803797 0.772182 2.018169

C -3.754099 0.629369 2.997022

C -4.453707 -0.590500 3.154241

C -4.163963 -1.646570 2.331122

O 1.240035 0.221420 0.090280

C 2.545403 2.734532 -0.238850

O -0.168476 -1.291865 -1.555235

Br -1.380507 -4.014949 -1.485475

N 3.301600 1.685307 0.007856

C 4.617977 1.674203 0.128469

C 5.319250 0.456317 0.380561

C 6.733060 0.455490 0.519023

C 7.398501 -0.728199 0.759456

C 6.668565 -1.917064 0.864614

C 5.285999 -1.823417 0.715174

N 4.615860 -0.702138 0.483056

H -3.867631 5.396337 -1.567870

H -4.939820 3.153481 -1.308526

H -3.589565 1.203199 -0.692722

H -1.426956 5.628876 -1.247201

H 0.751317 4.656244 -0.765136

H -3.371983 -3.577496 0.615682

H -2.268612 1.709216 1.910981

H -3.967963 1.460822 3.662388

H -5.206753 -0.689825 3.930653

H -4.676915 -2.598921 2.444892

H 2.226527 0.527324 0.124077

H 2.982819 3.729516 -0.376194

H 0.576701 -0.768358 -1.173660

H 5.189436 2.602087 0.032236

H 7.273286 1.394718 0.432519

H 8.481335 -0.733403 0.865978

H 7.146733 -2.872527 1.050705

H 4.674490 -2.724084 0.786657

-----------------------------------------------------

**INT1-ent-3b-6aC**

Zero-point correction= 0.382771 (Hartree/Particle)

Thermal correction to Energy= 0.407479

Thermal correction to Enthalpy= 0.408423

Thermal correction to Gibbs Free Energy= 0.326492

E(solv) = -3871.15680286 A.U.

C -0.899209 4.826460 -1.799712

C -2.072414 4.070792 -1.590497

C -1.994624 2.792237 -1.085982

C -0.749021 2.198748 -0.762214

C 0.438417 2.951440 -1.007977

C 0.324327 4.271601 -1.517505

C -0.627336 0.866423 -0.229989

C 0.629446 0.314377 -0.076245

C 1.842883 1.063047 -0.323819

C 1.701160 2.366671 -0.760701

C -1.841127 0.078767 0.131472

C -2.776509 0.584112 1.094105

C -4.002731 -0.099056 1.328367

C -4.267174 -1.301509 0.627887

C -3.326943 -1.813553 -0.217998

C -2.088580 -1.153275 -0.462218

C -2.516554 1.757645 1.856738

C -3.427682 2.229577 2.766755

C -4.658176 1.561893 2.971774

C -4.932651 0.418973 2.267904

O 0.741462 -0.975829 0.306333

C 3.143954 0.474028 -0.111332

O -1.217363 -1.755107 -1.292268

Br -3.661818 -3.449519 -1.106546

N 3.226620 -0.778136 0.300706

C 4.303203 -1.522390 0.570291

C 5.675862 -1.158318 0.494427

C 6.668987 -2.124229 0.838904

C 8.001862 -1.797708 0.774081

C 8.374877 -0.506209 0.367052

C 7.348993 0.373086 0.050146

N 6.048057 0.090714 0.100850

H -0.965836 5.837044 -2.193524

H -3.042259 4.495916 -1.833254

H -2.904376 2.217735 -0.943287

H 1.239701 4.833533 -1.690976

H 2.596833 2.956311 -0.948340

H -5.203650 -1.826945 0.787704

H -1.572078 2.271294 1.716187

H -3.198308 3.122633 3.340658

H -5.373204 1.949054 3.691864

H -5.865419 -0.118743 2.422152

H 1.786423 -1.148602 0.386994

H 4.029791 1.073510 -0.294625

H -0.318952 -1.612073 -0.908738

H 4.086225 -2.537491 0.892742

H 6.350710 -3.115253 1.151066

H 8.758908 -2.534000 1.035961

H 9.413582 -0.201125 0.300958

H 7.590572 1.388045 -0.270130

--------------------------------------------------------

**INT1-ent-3b-6aD**

Zero-point correction= 0.383521 (Hartree/Particle)

Thermal correction to Energy= 0.408218

Thermal correction to Enthalpy= 0.409162

Thermal correction to Gibbs Free Energy= 0.327282

E(solv) = -3871.15236473 A.U.

C -1.016647 4.826024 -1.842794

C -2.174527 4.062923 -1.582542

C -2.064820 2.787747 -1.074431

C -0.802454 2.207738 -0.797128

C 0.368692 2.968475 -1.091153

C 0.222120 4.284316 -1.604819

C -0.646883 0.878286 -0.265302

C 0.618852 0.340312 -0.152522

C 1.817914 1.098992 -0.439874

C 1.644492 2.398731 -0.883473

C -1.839178 0.075258 0.133056

C -2.744812 0.563819 1.131698

C -3.952208 -0.137268 1.407290

C -4.228163 -1.337553 0.707587

C -3.315959 -1.830507 -0.179218

C -2.095231 -1.153532 -0.462760

C -2.473509 1.738431 1.888459

C -3.356515 2.193489 2.834149

C -4.568157 1.506400 3.082859

C -4.852868 0.362857 2.384031

O 0.751454 -0.952305 0.222829

C 3.125683 0.527231 -0.253633

O -1.249173 -1.737896 -1.331219

Br -3.667492 -3.459947 -1.072151

N 3.253336 -0.727187 0.157130

C 4.320324 -1.477436 0.401015

C 5.710123 -1.124152 0.439458

C 6.219093 0.201239 0.469729

C 7.585579 0.409890 0.531265

C 8.453525 -0.680709 0.560723

C 7.868230 -1.947655 0.565884

N 6.566795 -2.183182 0.514837

H -1.107046 5.833108 -2.240955

H -3.156786 4.479010 -1.787770

H -2.962631 2.206594 -0.889275

H 1.124237 4.854088 -1.817862

H 2.525184 2.999220 -1.106068

H -5.151295 -1.876019 0.898566

H -1.543543 2.267721 1.712863

H -3.119557 3.088297 3.402196

H -5.261212 1.880179 3.830923

H -5.771514 -0.188694 2.570630

H 1.784609 -1.120960 0.276934

H 3.973714 1.150616 -0.527796

H -0.339392 -1.592188 -0.979820

H 4.120748 -2.523121 0.614489

H 5.543024 1.044138 0.506252

H 7.974093 1.425451 0.565458

H 9.531050 -0.561268 0.596208

H 8.503198 -2.833916 0.618329

-----------------------------------------------------------

**INT1-4f-6a**

Zero-point correction= 0.574147 (Hartree/Particle)

Thermal correction to Energy= 0.608602

Thermal correction to Enthalpy= 0.609546

Thermal correction to Gibbs Free Energy= 0.504736

E(solv) = -4184.12123141 A.U.

C -3.328395 0.130078 3.544179

C -3.405844 1.285068 2.742610

C -2.410280 1.564086 1.837269

C -1.285345 0.709718 1.679143

C -1.211829 -0.457549 2.499843

C -2.250899 -0.718350 3.421077

C -0.248748 0.986413 0.742696

C 0.867543 0.153893 0.603628

C 0.936921 -1.031836 1.444029

C -0.072001 -1.297955 2.335801

C -0.376935 2.209046 -0.107167

C 0.354683 3.391982 0.242185

C 0.171221 4.591351 -0.500669

C -0.745369 4.595033 -1.582928

C -1.434677 3.459490 -1.906903

C -1.255155 2.249582 -1.179839

C 1.266209 3.404985 1.333467

C 1.957167 4.543827 1.659975

C 1.774497 5.734180 0.916915

C 0.900010 5.754527 -0.138271

H -4.114457 -0.092385 4.260289

H -4.252209 1.959846 2.839040

H -2.472669 2.460074 1.227275

H -2.183373 -1.608828 4.036423

H -0.884638 5.511506 -2.151750

H 1.406207 2.489533 1.898740

H 2.652583 4.532539 2.493887

H 2.329616 6.628607 1.185089

H 0.748374 6.663118 -0.716899

Br 0.089637 -2.877907 3.382191

O 1.979949 -1.862747 1.339765

H 2.581125 -1.478489 0.629123

H -2.125602 3.457905 -2.746120

C 1.894416 0.431608 -0.354613

H 1.797332 1.327648 -0.971667

C -2.057465 1.062504 -1.577375

C -1.459985 -0.177101 -1.812847

C -3.443135 1.166193 -1.733602

C -2.228488 -1.279371 -2.176292

H -0.382382 -0.278871 -1.712479

C -4.204634 0.061971 -2.094557

H -3.929611 2.119885 -1.541344

C -3.615382 -1.188080 -2.318400

H -1.721380 -2.223569 -2.342225

H -5.282185 0.176600 -2.188622

C -4.494998 -2.385510 -2.687410

C -3.674951 -3.661983 -2.899229

H -2.950756 -3.545225 -3.712497

H -3.130130 -3.943626 -1.992441

H -4.344290 -4.489156 -3.160509

C -5.258504 -2.082935 -3.987307

H -5.897551 -1.200388 -3.882813

H -4.559941 -1.897244 -4.810041

H -5.896431 -2.932243 -4.260187

C -5.501386 -2.643680 -1.553744

H -6.149751 -1.777550 -1.388359

H -6.137548 -3.502823 -1.798983

H -4.978379 -2.854287 -0.615599

N 2.963624 -0.348478 -0.508343

C 3.916967 -0.084734 -1.378023

H 3.863849 0.805006 -2.009893

C 5.067601 -0.921572 -1.552827

C 5.275834 -2.118318 -0.820319

C 6.412791 -2.864062 -1.051414

H 4.537263 -2.431775 -0.090109

C 7.055201 -1.246778 -2.674848

C 7.342801 -2.431088 -2.003662

H 6.581521 -3.783648 -0.495691

H 7.750261 -0.868748 -3.426377

H 8.249234 -2.987467 -2.216889

N 5.970987 -0.505098 -2.476883

---------------------------------------------------

**INT1-4f-6aB**

Zero-point correction= 0.574565 (Hartree/Particle)

Thermal correction to Energy= 0.608819

Thermal correction to Enthalpy= 0.609763

Thermal correction to Gibbs Free Energy= 0.506126

E(solv) = -4184.11875352 A.U.

C -2.894758 -0.147479 3.879129

C -3.190361 0.978965 3.086988

C -2.339276 1.350089 2.072950

C -1.152097 0.620983 1.792412

C -0.855950 -0.516677 2.605469

C -1.750906 -0.873806 3.639809

C -0.259412 0.987991 0.743224

C 0.911199 0.270952 0.478007

C 1.214231 -0.882523 1.316916

C 0.342875 -1.229312 2.323754

C -0.583136 2.190626 -0.083367

C 0.095204 3.427443 0.183709

C -0.237934 4.599691 -0.551378

C -1.250262 4.524477 -1.541558

C -1.889184 3.341288 -1.784056

C -1.561581 2.156559 -1.065155

C 1.106386 3.521714 1.179493

C 1.748832 4.709335 1.421693

C 1.416656 5.871177 0.685229

C 0.443635 5.814198 -0.278069

H -3.566312 -0.443934 4.680079

H -4.091111 1.556662 3.276368

H -2.570522 2.221734 1.467550

H -1.515959 -1.740244 4.247832

H -1.502039 5.418373 -2.107517

H 1.363568 2.629800 1.740793

H 2.522125 4.759240 2.182519

H 1.934807 6.804743 0.885735

H 0.176451 6.698773 -0.851974

Br 0.807272 -2.746835 3.369694

O 2.312443 -1.607598 1.125726

H 2.820153 -1.208350 0.344232

H -2.651550 3.279084 -2.556381

C 1.788783 0.621229 -0.606056

H 1.531196 1.471689 -1.243116

C -2.318397 0.919838 -1.395205

C -1.676514 -0.290825 -1.662065

C -3.714039 0.958231 -1.486237

C -2.410362 -1.424694 -2.001473

H -0.592152 -0.344006 -1.616165

C -4.439690 -0.175630 -1.826042

H -4.235169 1.887445 -1.267285

C -3.804289 -1.394683 -2.090009

H -1.868528 -2.343414 -2.198057

H -5.524048 -0.108137 -1.877867

C -4.642643 -2.620891 -2.460294

C -3.778845 -3.865908 -2.683717

H -3.070066 -3.722769 -3.506140

H -3.212205 -4.126721 -1.784100

H -4.420069 -4.717260 -2.937601

C -5.419788 -2.333768 -3.755931

H -6.087086 -1.473478 -3.641537

H -4.729811 -2.117231 -4.578355

H -6.029625 -3.201478 -4.035203

C -5.636830 -2.923602 -1.327167

H -6.316336 -2.083534 -1.152913

H -6.242957 -3.802222 -1.579062

H -5.105338 -3.124427 -0.391627

N 2.893111 -0.072381 -0.822631

C 3.778897 0.134774 -1.773489

H 3.651533 0.947940 -2.495053

C 4.931987 -0.709468 -1.882097

C 5.889566 -0.500098 -2.907513

C 6.991490 -1.326558 -2.995333

H 5.740711 0.311424 -3.614888

C 6.160375 -2.492809 -1.094363

C 7.148954 -2.361245 -2.068566

H 7.729429 -1.171380 -3.779509

H 6.240005 -3.284571 -0.348061

H 7.998467 -3.034949 -2.096939

N 5.091389 -1.715665 -0.984330

---------------------------------------------------

**INT1-4f-6aC**

Zero-point correction= 0.575203 (Hartree/Particle)

Thermal correction to Energy= 0.609370

Thermal correction to Enthalpy= 0.610314

Thermal correction to Gibbs Free Energy= 0.507223

E(solv) = -4184.11297941 A.U.

C 3.422251 1.711911 2.960422

C 2.739659 0.544124 3.350656

C 1.615015 0.143561 2.669880

C 1.110987 0.878584 1.563602

C 1.802881 2.069641 1.180310

C 2.956667 2.455682 1.899042

C -0.050888 0.472230 0.847303

C -0.552321 1.210879 -0.232092

C 0.151221 2.424899 -0.613063

C 1.273093 2.806847 0.080122

C -0.752725 -0.784594 1.247431

C -2.018486 -0.709487 1.920960

C -2.705102 -1.900333 2.287464

C -2.096396 -3.154399 2.025847

C -0.879444 -3.211621 1.406912

C -0.202952 -2.031383 0.988843

C -2.629150 0.540383 2.219320

C -3.861223 0.597729 2.820085

C -4.551399 -0.589786 3.161699

C -3.979762 -1.809438 2.906670

H 4.312352 2.030059 3.495945

H 3.098279 -0.041567 4.192900

H 1.086695 -0.752360 2.981345

H 3.474881 3.359162 1.596207

H -2.618702 -4.063973 2.312827

H -2.101879 1.449124 1.948716

H -4.316178 1.561760 3.026797

H -5.530530 -0.529574 3.628376

H -4.493929 -2.730520 3.172250

Br 2.142686 4.398115 -0.495096

O -0.287105 3.147437 -1.647187

H -1.127929 2.690034 -1.981200

H -0.420561 -4.172383 1.187915

C -1.719253 0.782767 -0.942746

H -2.133760 -0.181813 -0.671634

C 1.094559 -2.190607 0.279759

C 1.330595 -1.586284 -0.956242

C 2.107144 -2.981202 0.831307

C 2.545174 -1.763698 -1.612997

H 0.552384 -0.978004 -1.409939

C 3.317723 -3.151753 0.172838

H 1.947637 -3.448088 1.800744

C 3.565979 -2.544028 -1.063658

H 2.684191 -1.274540 -2.570970

H 4.087764 -3.760900 0.641139

C 4.922490 -2.742099 -1.744625

C 5.005915 -2.014433 -3.090001

H 4.250220 -2.381501 -3.792658

H 4.868918 -0.934861 -2.970728

H 5.991743 -2.181509 -3.538066

C 5.160888 -4.240730 -1.992122

H 5.148844 -4.808762 -1.056837

H 4.384610 -4.652140 -2.645902

H 6.135286 -4.397684 -2.470410

C 6.032081 -2.193855 -0.831741

H 6.049284 -2.711216 0.132550

H 7.013448 -2.323753 -1.304105

H 5.877731 -1.127936 -0.636872

N -2.254722 1.507570 -1.928089

C -3.371395 1.258053 -2.587123

H -3.565252 1.876177 -3.459637

C -4.412329 0.296406 -2.298173

C -4.610397 -0.325265 -1.038115

C -5.670486 -1.196181 -0.862003

H -3.974436 -0.080682 -0.196335

C -6.292321 -0.769438 -3.115597

C -6.532484 -1.457197 -1.926744

H -5.821700 -1.660851 0.109730

H -6.960873 -0.913533 -3.966215

H -7.365697 -2.146769 -1.839940

N -5.291685 0.078113 -3.313443

--------------------------------------------------

**INT1-4f-6aD**

Zero-point correction= 0.574779 (Hartree/Particle)

Thermal correction to Energy= 0.609064

Thermal correction to Enthalpy= 0.610008

Thermal correction to Gibbs Free Energy= 0.505728

E(solv) = -4184.11609964 A.U.

C 3.542071 2.175692 2.619538

C 2.838359 1.125758 3.241473

C 1.681421 0.643535 2.677016

C 1.163912 1.177252 1.465723

C 1.880959 2.246926 0.844934

C 3.068115 2.721269 1.448959

C -0.033670 0.686318 0.865581

C -0.540471 1.225122 -0.318996

C 0.191794 2.317157 -0.942543

C 1.347365 2.781688 -0.360339

C -0.767432 -0.430994 1.536511

C -1.939978 -0.139229 2.309953

C -2.617845 -1.183351 2.999114

C -2.109982 -2.504717 2.916295

C -0.989051 -2.767106 2.179948

C -0.308046 -1.737217 1.472221

C -2.454361 1.182205 2.414166

C -3.578452 1.444525 3.154685

C -4.252656 0.402370 3.834120

C -3.779489 -0.881266 3.756693

H 4.457659 2.557658 3.062572

H 3.208202 0.696479 4.168708

H 1.139398 -0.161830 3.163819

H 3.603009 3.531599 0.966329

H -2.630851 -3.303203 3.439675

H -1.941582 1.980176 1.887937

H -3.959560 2.459543 3.215826

H -5.145012 0.623944 4.412718

H -4.287349 -1.693167 4.272550

Br 2.242110 4.204659 -1.248728

O -0.253265 2.858803 -2.073612

H -1.110440 2.341746 -2.304266

H -0.609277 -3.782515 2.100921

C -1.737742 0.699673 -0.932835

H -2.256491 -0.128246 -0.462964

C 0.902854 -2.111150 0.694966

C 1.049303 -1.759855 -0.647900

C 1.921460 -2.858069 1.295577

C 2.185422 -2.134597 -1.360320

H 0.260157 -1.199372 -1.142220

C 3.052574 -3.227703 0.579799

H 1.831585 -3.130952 2.344716

C 3.212392 -2.871212 -0.764451

H 2.256402 -1.836856 -2.400926

H 3.830772 -3.795358 1.085201

C 4.482803 -3.284895 -1.511184

C 4.475219 -2.814457 -2.969079

H 3.633168 -3.241832 -3.523917

H 4.414085 -1.723635 -3.038722

H 5.400247 -3.132848 -3.462643

C 4.610310 -4.817000 -1.501140

H 4.665398 -5.208558 -0.480553

H 3.747997 -5.279315 -1.992924

H 5.518778 -5.126994 -2.031923

C 5.706139 -2.667650 -0.813128

H 5.790313 -3.009600 0.223119

H 6.626815 -2.948179 -1.339324

H 5.631055 -1.575731 -0.800854

N -2.189793 1.239750 -2.056872

C -3.258410 0.919035 -2.777262

H -3.408025 1.525782 -3.668333

C -4.230893 -0.100688 -2.546125

C -5.303682 -0.246100 -3.471703

C -6.256846 -1.217364 -3.274751

H -5.352067 0.420178 -4.328774

C -5.081495 -1.850305 -1.302443

C -6.158034 -2.057440 -2.155435

H -7.076664 -1.330929 -3.980745

H -4.962193 -2.477504 -0.417816

H -6.885865 -2.836927 -1.957415

N -4.141780 -0.920324 -1.466162

---------------------------------------------------

**C-3b-RS-TS1**

Zero-point correction= 0.865294 (Hartree/Particle)

Thermal correction to Energy= 0.916414

Thermal correction to Enthalpy= 0.917359

Thermal correction to Gibbs Free Energy= 0.780858

E(solv) = -4860.65281977 A.U.

C -2.374166 -0.300620 5.436673

C -3.525985 -0.125646 4.634309

C -3.412803 0.245232 3.317083

C -2.140950 0.461293 2.724627

C -0.981279 0.285657 3.535518

C -1.131664 -0.097156 4.893606

C -1.984350 0.835526 1.354725

C -0.717827 1.009725 0.821023

C 0.460725 0.835314 1.634425

C 0.293903 0.480034 2.956892

C -3.169919 1.130136 0.502276

C -3.463082 2.478359 0.119885

C -4.600325 2.753898 -0.688975

C -5.422625 1.680539 -1.112685

C -5.120078 0.404276 -0.737650

C -3.992637 0.101775 0.080141

C -2.657676 3.573569 0.535409

C -2.965557 4.857760 0.165437

C -4.094600 5.125306 -0.644948

C -4.893007 4.092297 -1.060137

O -0.598529 1.401122 -0.455761

C 1.799978 1.057952 1.110367

O -3.798811 -1.188531 0.429363

Br -6.172551 -1.032657 -1.367779

N 1.984769 1.437816 -0.123797

C 3.173348 1.638132 -0.733143

H -2.477262 -0.598140 6.476192

H -4.509948 -0.292600 5.063124

H -4.301220 0.368827 2.706700

H -0.234203 -0.231274 5.493419

H 1.177879 0.354120 3.580456

H -6.281435 1.874177 -1.747806

H -1.790937 3.375480 1.156567

H -2.334515 5.678109 0.494321

H -4.324168 6.146686 -0.934252

H -5.765672 4.277745 -1.681983

H 0.386243 1.468588 -0.647811

H 2.631025 0.949063 1.807582

H -2.924458 -1.523939 0.114641

H 3.131927 1.973645 -1.763030

C 4.428089 1.778252 -0.054199

O 4.637896 1.653974 1.149757

O 5.424819 2.075711 -0.949542

C 6.736111 2.435090 -0.481209

C 6.668211 3.702125 0.372315

H 7.681560 4.039363 0.617254

H 6.162466 4.498731 -0.183304

H 6.121274 3.511150 1.296331

C 7.396714 1.281005 0.270902

H 6.889990 1.090860 1.216655

H 7.350028 0.372767 -0.336883

H 8.449717 1.521551 0.460515

C 7.497369 2.712788 -1.775810

H 7.514365 1.812859 -2.398300

H 7.008284 3.513562 -2.338679

H 8.527885 3.011005 -1.557400

C 4.700029 -1.311996 0.653917

C 4.624805 -1.268633 -0.746220

C 5.742876 -1.688206 -1.485026

C 6.867198 -2.205679 -0.853143

C 6.913650 -2.276198 0.538476

C 5.835046 -1.809797 1.285743

C 3.423183 -0.851590 -1.481874

H 3.877292 -0.921025 1.242486

H 5.706077 -1.633556 -2.570216

H 7.710719 -2.547920 -1.446067

H 7.793107 -2.673653 1.036601

H 5.876671 -1.825462 2.370690

H 3.623057 -0.406869 -2.454534

C 2.126429 -1.274014 -1.252792

C 1.122448 -0.934445 -2.224980

C 1.712093 -2.078248 -0.140119

C -0.170828 -1.342780 -2.144125

H 1.456084 -0.329273 -3.063858

C 0.427847 -2.504572 0.027531

H 2.476416 -2.392487 0.558126

C -0.587491 -2.117791 -0.960693

O -1.780211 -2.474890 -0.826586

C 0.025271 -3.442971 1.170589

C 1.180234 -3.627777 2.163571

H 2.062439 -4.079777 1.696736

H 1.472932 -2.668195 2.607442

H 0.857413 -4.291395 2.972746

C -1.175441 -2.891139 1.962672

H -2.113066 -3.027215 1.425626

H -1.253933 -3.408144 2.927146

H -1.042877 -1.825812 2.163909

C -0.339302 -4.819068 0.586140

H 0.524206 -5.268619 0.082323

H -0.659203 -5.494902 1.389265

H -1.157401 -4.721582 -0.131319

C -1.188953 -1.080055 -3.258472

C -0.585208 -0.206933 -4.365232

H -0.290881 0.776010 -3.980150

H 0.289314 -0.676847 -4.829426

H -1.334274 -0.051388 -5.148855

C -1.594813 -2.429477 -3.879723

H -2.037496 -3.079942 -3.122565

H -2.333430 -2.265757 -4.674231

H -0.724780 -2.932614 -4.317819

C -2.449224 -0.363578 -2.739677

H -3.060983 -0.038187 -3.590971

H -3.057160 -1.031761 -2.128969

H -2.179099 0.517182 -2.147937

**C-3b-RS-TS2**

Zero-point correction= 0.865517 (Hartree/Particle)

Thermal correction to Energy= 0.916353

Thermal correction to Enthalpy= 0.917297

Thermal correction to Gibbs Free Energy= 0.780256

E(solv) = -4860.64983692 A.U.

C -2.845201 -5.069495 -2.298730

C -4.040565 -4.429258 -1.905006

C -4.020440 -3.125817 -1.466939

C -2.810592 -2.388500 -1.400452

C -1.611706 -3.025079 -1.834712

C -1.661425 -4.376358 -2.266604

C -2.749793 -1.029098 -0.932699

C -1.566444 -0.325590 -1.059303

C -0.342218 -0.959441 -1.495620

C -0.400922 -2.294336 -1.842075

C -3.942936 -0.387744 -0.307236

C -4.560393 -0.983628 0.844135

C -5.754945 -0.427161 1.382078

C -6.309460 0.729922 0.781824

C -5.674554 1.320551 -0.271199

C -4.470296 0.791484 -0.819642

C -4.015444 -2.125903 1.497688

C -4.622629 -2.676553 2.597916

C -5.819739 -2.127706 3.113470

C -6.368323 -1.024218 2.514527

O -1.558755 0.987891 -0.744677

C 0.905185 -0.228350 -1.522550

O -3.914206 1.460812 -1.845052

Br -6.392049 2.894798 -1.031292

N 0.942937 1.044281 -1.213858

C 2.063240 1.760380 -1.166483

H -2.866845 -6.101958 -2.635768

H -4.983138 -4.967014 -1.951756

H -4.946893 -2.640985 -1.175848

H -0.733421 -4.849115 -2.580895

H 0.510827 -2.794534 -2.164497

H -7.226004 1.157373 1.176258

H -3.098575 -2.561707 1.116036

H -4.176293 -3.542280 3.077951

H -6.293307 -2.575245 3.982089

H -7.282799 -0.579662 2.900259

H -0.593479 1.325700 -0.824178

H 1.815816 -0.771369 -1.799369

H -2.940209 1.460041 -1.694778

H 3.024988 1.317067 -1.423223

C 2.026004 3.100030 -0.675394

O 1.048576 3.760549 -0.348818

O 3.322314 3.574426 -0.546603

C 3.560878 4.927349 -0.132861

C 5.083690 5.037075 -0.160833

H 5.402324 6.041954 0.135060

H 5.526656 4.309652 0.527480

H 5.458428 4.828752 -1.167613

C 2.942300 5.912794 -1.125271

H 3.300382 5.690273 -2.135824

H 1.854657 5.836478 -1.109845

H 3.239758 6.936003 -0.869559

C 3.047260 5.167258 1.287928

H 1.959257 5.101577 1.316118

H 3.471686 4.417708 1.966709

H 3.362932 6.157476 1.636022

C 0.157946 -0.084187 1.765003

C 1.084401 0.963258 1.917854

C 0.617250 2.184017 2.437293

C -0.703790 2.342092 2.831846

C -1.597718 1.281695 2.705550

C -1.160477 0.073453 2.169588

C 2.479669 0.917769 1.502395

H 0.442978 -1.002873 1.268431

H 1.308913 3.018566 2.511913

H -1.041149 3.300158 3.215206

H -2.639679 1.400999 2.988540

H -1.865974 -0.738456 2.025602

H 2.946856 1.902303 1.472401

C 3.304359 -0.122533 1.154159

C 4.586033 0.234781 0.589250

C 2.986488 -1.527394 1.218522

C 5.452342 -0.663389 0.066627

H 4.793476 1.300830 0.533417

C 3.806296 -2.492807 0.730896

H 2.072592 -1.811803 1.719756

C 5.106449 -2.106831 0.125495

O 5.886913 -2.963578 -0.306783

C 3.437340 -3.976287 0.786814

C 2.048895 -4.191170 1.401090

H 2.000094 -3.836064 2.436780

H 1.266707 -3.685333 0.821764

H 1.819918 -5.262270 1.406333

C 3.403291 -4.557089 -0.639436

H 4.378462 -4.474747 -1.119628

H 3.112222 -5.614111 -0.601444

H 2.662473 -4.023697 -1.246308

C 4.461204 -4.748486 1.637885

H 4.465972 -4.368539 2.665991

H 4.191398 -5.811445 1.669831

H 5.464454 -4.651724 1.220801

C 6.762495 -0.243827 -0.600658

C 6.932397 1.280125 -0.592533

H 6.114370 1.787384 -1.116871

H 6.980151 1.674843 0.429026

H 7.870418 1.540383 -1.095372

C 7.959752 -0.858859 0.146006

H 7.909048 -1.948055 0.130378

H 8.896479 -0.538824 -0.327242

H 7.973375 -0.519737 1.188256

C 6.769667 -0.709585 -2.068125

H 7.700954 -0.392298 -2.553590

H 6.690290 -1.795484 -2.133401

H 5.931935 -0.259011 -2.612548

**C-3b-RS-TS3**

Zero-point correction= 0.866137 (Hartree/Particle)

Thermal correction to Energy= 0.916721

Thermal correction to Enthalpy= 0.917665

Thermal correction to Gibbs Free Energy= 0.779760

E(solv) = -4860.64835325 A.U.

C -4.214814 -5.319584 -1.470409

C -5.244702 -4.445498 -1.055176

C -4.975449 -3.127415 -0.777090

C -3.660923 -2.603538 -0.897332

C -2.630042 -3.481352 -1.344271

C -2.938121 -4.840680 -1.613834

C -3.341269 -1.235071 -0.606208

C -2.066261 -0.770974 -0.866268

C -1.017341 -1.649424 -1.323097

C -1.321355 -2.976656 -1.524930

C -4.364744 -0.309871 -0.040648

C -5.000238 -0.603589 1.210701

C -6.065650 0.217457 1.675053

C -6.457659 1.342692 0.908251

C -5.784876 1.654202 -0.236699

C -4.712614 0.850711 -0.719032

C -4.595404 -1.693569 2.031618

C -5.225876 -1.957684 3.221538

C -6.302531 -1.153027 3.662856

C -6.707355 -0.086989 2.903277

O -1.810914 0.541591 -0.677834

C 0.339702 -1.154195 -1.482688

O -4.097679 1.254132 -1.848158

Br -6.269698 3.191811 -1.221166

N 0.595124 0.091518 -1.236043

C 1.832607 0.634748 -1.135047

H -4.438371 -6.360621 -1.683844

H -6.260505 -4.817834 -0.959714

H -5.778551 -2.466149 -0.469232

H -2.134516 -5.493843 -1.946039

H -0.533486 -3.657375 -1.842540

H -7.273941 1.971653 1.249342

H -3.766529 -2.313214 1.704605

H -4.891781 -2.790572 3.833095

H -6.795660 -1.375168 4.604331

H -7.523142 0.552179 3.232221

H -0.801099 0.675348 -0.848254

H 1.125918 -1.867054 -1.758267

H -3.134929 1.110754 -1.714531

H 2.689865 0.151054 -1.597161

C 1.915645 2.082234 -0.973214

O 2.937015 2.735001 -1.075719

O 0.719729 2.598662 -0.569451

C 0.602564 3.980277 -0.168735

C -0.873364 4.102104 0.200700

H -1.099085 5.119967 0.534053

H -1.503628 3.863953 -0.661470

H -1.123053 3.403156 1.005025

C 1.482557 4.250082 1.050783

H 1.229219 3.549541 1.854671

H 2.539248 4.140522 0.800100

H 1.305652 5.267121 1.417074

C 0.938145 4.912879 -1.331381

H 1.996277 4.853997 -1.586723

H 0.343043 4.638185 -2.208109

H 0.689587 5.943629 -1.056194

C 1.702629 -2.098261 1.530200

C 1.542886 -0.718022 1.347712

C 0.272868 -0.168713 1.601345

C -0.798931 -0.959607 1.987945

C -0.629323 -2.337111 2.130037

C 0.624564 -2.897105 1.908159

C 2.601774 0.202479 0.895526

H 2.680626 -2.549300 1.411383

H 0.127172 0.897944 1.440509

H -1.774596 -0.506752 2.147158

H -1.467450 -2.965579 2.418632

H 0.773329 -3.965626 2.036309

H 2.424501 1.218256 1.252471

C 3.970607 -0.077572 0.666435

C 4.910775 0.975258 0.844591

C 4.460700 -1.285448 0.093494

C 6.250257 0.844329 0.599854

H 4.513475 1.921227 1.201646

C 5.784127 -1.494178 -0.185951

H 3.734453 -2.040900 -0.187885

C 6.769419 -0.440549 0.118883

O 7.994058 -0.630121 -0.056634

C 6.280424 -2.793387 -0.824139

C 5.131245 -3.759845 -1.132714

H 4.588788 -4.051225 -0.225353

H 4.412782 -3.324766 -1.837439

H 5.536296 -4.671473 -1.586581

C 7.001233 -2.488702 -2.150058

H 7.847705 -1.823406 -1.977460

H 7.362586 -3.420694 -2.604146

H 6.312017 -2.010244 -2.855543

C 7.249254 -3.508423 0.135277

H 6.733347 -3.773364 1.065697

H 7.620461 -4.433990 -0.324455

H 8.093597 -2.859987 0.371231

C 7.225574 2.005655 0.801210

C 6.504582 3.284526 1.243305

H 5.750805 3.595529 0.510623

H 6.010684 3.156908 2.213674

H 7.235683 4.094984 1.345947

C 8.262439 1.646043 1.879526

H 8.815457 0.751683 1.590039

H 8.967693 2.476489 2.017780

H 7.764262 1.459894 2.838283

C 7.946818 2.314540 -0.523540

H 8.646765 3.149022 -0.384430

H 8.498897 1.439913 -0.869850

H 7.218713 2.603028 -1.290084

**C-3b-RS-TS4**

Zero-point correction= 0.865758 (Hartree/Particle)

Thermal correction to Energy= 0.916415

Thermal correction to Enthalpy= 0.917359

Thermal correction to Gibbs Free Energy= 0.779919

E(solv) = -4860.64808749 A.U.

C -4.934548 -4.648997 -2.529965

C -5.808626 -3.822736 -1.787609

C -5.360532 -2.649414 -1.231445

C -4.012384 -2.229843 -1.384990

C -3.142039 -3.050598 -2.160208

C -3.631609 -4.263274 -2.712248

C -3.508654 -1.014796 -0.810732

C -2.224725 -0.605974 -1.117650

C -1.336575 -1.431322 -1.896374

C -1.808233 -2.632919 -2.372274

C -4.353451 -0.197408 0.106070

C -4.868699 -0.771836 1.315043

C -5.783295 -0.034443 2.117174

C -6.141876 1.279465 1.726803

C -5.573093 1.840972 0.621252

C -4.651235 1.124875 -0.194588

C -4.484799 -2.066155 1.766315

C -4.993839 -2.592934 2.926665

C -5.924325 -1.864328 3.704108

C -6.305564 -0.610378 3.304271

O -1.805270 0.594743 -0.664718

C 0.053022 -1.046756 -2.085147

O -4.129432 1.775052 -1.253272

Br -6.002477 3.619898 0.149502

N 0.477169 0.080722 -1.617584

C 1.784564 0.455991 -1.532293

H -5.298785 -5.577370 -2.959661

H -6.846690 -4.115251 -1.659315

H -6.046253 -2.020144 -0.673953

H -2.947930 -4.877391 -3.293877

H -1.137048 -3.279085 -2.935748

H -6.844158 1.849397 2.327117

H -3.766153 -2.631811 1.181914

H -4.674316 -3.577755 3.254083

H -6.322640 -2.294171 4.618084

H -7.006800 -0.026950 3.896036

H -0.815768 0.685070 -0.927157

H 0.720063 -1.756224 -2.588135

H -3.183315 1.518182 -1.309925

H 2.544990 -0.081101 -2.100760

C 2.008531 1.900877 -1.404403

O 3.018702 2.483928 -1.744345

O 0.963788 2.492942 -0.770720

C 0.974616 3.906168 -0.467245

C -0.372726 4.112440 0.219131

H -0.499122 5.162513 0.500406

H -1.191128 3.823822 -0.447457

H -0.434430 3.495513 1.120822

C 2.118598 4.236220 0.489749

H 2.088302 3.563347 1.354196

H 3.087228 4.135351 -0.002294

H 2.008714 5.263806 0.853047

C 1.052764 4.727609 -1.752469

H 2.016846 4.589766 -2.243228

H 0.254086 4.422704 -2.436312

H 0.917765 5.788548 -1.516400

C 1.540624 -2.449719 0.127219

C 1.446165 -1.165733 0.688451

C 0.271858 -0.847436 1.385679

C -0.743052 -1.780327 1.567021

C -0.613869 -3.062726 1.037421

C 0.529768 -3.388912 0.312780

C 2.428917 -0.091477 0.451470

H 2.392605 -2.701378 -0.496459

H 0.149103 0.160662 1.774039

H -1.644707 -1.496957 2.104048

H -1.408420 -3.793050 1.164221

H 0.626702 -4.373639 -0.136107

H 2.075526 0.868956 0.827568

C 3.846600 -0.241825 0.476949

C 4.650925 0.931182 0.418051

C 4.533164 -1.483481 0.561478

C 6.018625 0.910436 0.370526

H 4.126294 1.881561 0.388620

C 5.899619 -1.585790 0.543032

H 3.946996 -2.380569 0.711310

C 6.722574 -0.375976 0.408896

O 7.973426 -0.441214 0.358809

C 6.607534 -2.937246 0.671073

C 5.617999 -4.096971 0.831617

H 5.006080 -3.989191 1.734519

H 4.945186 -4.178884 -0.030563

H 6.173158 -5.038775 0.912623

C 7.442044 -3.208496 -0.594261

H 8.179056 -2.417676 -0.736329

H 7.958342 -4.173754 -0.505949

H 6.791140 -3.249328 -1.475567

C 7.531069 -2.935297 1.902986

H 6.943971 -2.783427 2.816426

H 8.049935 -3.899466 1.987426

H 8.268602 -2.136043 1.818924

C 6.842891 2.195804 0.270146

C 5.950468 3.441928 0.228265

H 5.264789 3.412999 -0.626707

H 5.355867 3.544161 1.144718

H 6.578950 4.335911 0.136809

C 7.781844 2.317598 1.483006

H 8.453090 1.458744 1.528407

H 8.376604 3.237854 1.409671

H 7.199074 2.362394 2.410673

C 7.675698 2.177764 -1.025064

H 8.278015 3.093333 -1.096745

H 8.337670 1.310964 -1.037563

H 7.013337 2.135886 -1.897126

**C-3b-SR-TS1**

Zero-point correction= 0.865300 (Hartree/Particle)

Thermal correction to Energy= 0.915879

Thermal correction to Enthalpy= 0.916824

Thermal correction to Gibbs Free Energy= 0.783301

E(solv) = -4860.65302399 A.U.

C -4.594134 3.492154 -3.107099

C -5.189588 2.358965 -2.506065

C -4.413571 1.355440 -1.980314

C -2.996825 1.425334 -2.025237

C -2.399556 2.568749 -2.631023

C -3.227678 3.589135 -3.165610

C -2.157002 0.407269 -1.476327

C -0.778350 0.527482 -1.538006

C -0.168427 1.682628 -2.151993

C -0.990523 2.662971 -2.668054

C -2.748826 -0.812369 -0.859492

C -2.628918 -2.086775 -1.500469

C -3.213178 -3.236903 -0.901006

C -3.903608 -3.101012 0.329086

C -3.999791 -1.875455 0.920675

C -3.423189 -0.707018 0.343260

C -1.946414 -2.249365 -2.736147

C -1.848014 -3.480542 -3.332243

C -2.424425 -4.622973 -2.728156

C -3.093720 -4.499253 -1.539082

O -0.023395 -0.466070 -1.044861

C 1.272647 1.813946 -2.252998

O -3.594001 0.475110 0.979270

Br -4.850848 -1.703545 2.598183

N 2.073132 0.826408 -1.978624

C 3.404880 0.913603 -2.095670

H -5.219325 4.281160 -3.515218

H -6.272073 2.284535 -2.453625

H -4.880578 0.494801 -1.513263

H -2.750932 4.455224 -3.619628

H -0.536361 3.535904 -3.135522

H -4.339561 -3.973924 0.804940

H -1.501939 -1.377013 -3.202887

H -1.319428 -3.581391 -4.275595

H -2.333691 -5.593231 -3.207910

H -3.544574 -5.365683 -1.060846

H 0.932558 -0.262759 -1.246993

H 1.660368 2.763418 -2.648178

H -2.747830 0.794738 1.384014

H 3.867317 1.777567 -2.578401

C 4.239434 -0.253463 -1.987379

O 5.399626 -0.301633 -2.384117

O 3.625584 -1.286946 -1.351818

C 4.282672 -2.566925 -1.256834

C 3.262550 -3.421445 -0.511437

H 3.606772 -4.458977 -0.449365

H 3.127823 -3.038089 0.503995

H 2.295356 -3.393425 -1.022487

C 4.533455 -3.140353 -2.651478

H 3.592447 -3.185227 -3.209534

H 5.245949 -2.522112 -3.198944

H 4.932818 -4.156978 -2.564603

C 5.570262 -2.464655 -0.439995

H 6.343775 -1.926548 -0.987444

H 5.369885 -1.929050 0.494606

H 5.926806 -3.472126 -0.193945

C 6.033209 1.125193 0.047704

C 4.874083 1.054827 0.837895

C 4.977252 0.444215 2.101753

C 6.177264 -0.110930 2.530957

C 7.307825 -0.060824 1.717811

C 7.230362 0.567326 0.477237

C 3.650421 1.736048 0.393525

H 5.975862 1.591199 -0.930528

H 4.121297 0.448537 2.767980

H 6.232247 -0.572390 3.512971

H 8.243390 -0.498915 2.053697

H 8.102555 0.614271 -0.168056

H 3.826336 2.650807 -0.170684

C 2.364799 1.552920 0.872201

C 1.925473 0.353274 1.530085

C 1.406790 2.611015 0.728870

C 0.690853 0.231063 2.089331

H 2.613037 -0.485400 1.533135

C 0.155726 2.567199 1.272540

H 1.750955 3.503683 0.214018

C -0.267506 1.344786 1.958810

O -1.423665 1.245652 2.438132

C -0.782946 3.778399 1.270174

C -0.233543 4.898325 0.377463

H 0.738871 5.264437 0.725376

H -0.130863 4.557980 -0.660230

H -0.928703 5.744600 0.390682

C -2.195224 3.423618 0.763718

H -2.801601 2.973103 1.549460

H -2.704643 4.328717 0.410159

H -2.147626 2.719939 -0.069389

C -0.889012 4.314416 2.709357

H 0.087700 4.653774 3.073566

H -1.583943 5.163341 2.744918

H -1.263803 3.532586 3.375117

C 0.269650 -1.018240 2.868216

C 1.365299 -2.088992 2.843103

H 1.538403 -2.447366 1.823272

H 2.315255 -1.723974 3.251744

H 1.047176 -2.943579 3.450159

C 0.018152 -0.628517 4.336309

H -0.761388 0.132182 4.404443

H -0.301650 -1.511033 4.904362

H 0.936138 -0.241003 4.794434

C -1.002029 -1.649627 2.277216

H -1.176221 -2.632664 2.733763

H -1.874687 -1.028601 2.478380

H -0.897499 -1.782728 1.194085

**C-3b-SR-TS2**

Zero-point correction= 0.864649 (Hartree/Particle)

Thermal correction to Energy= 0.915417

Thermal correction to Enthalpy= 0.916361

Thermal correction to Gibbs Free Energy= 0.782021

E(solv) = -4860.65131287 A.U.

C 4.594429 -3.498793 -3.133949

C 5.151980 -2.351741 -2.521952

C 4.342423 -1.386742 -1.975325

C 2.929116 -1.511489 -2.011125

C 2.368517 -2.667903 -2.628973

C 3.232018 -3.648087 -3.182817

C 2.056085 -0.534516 -1.444918

C 0.682227 -0.707666 -1.491055

C 0.105449 -1.865762 -2.141487

C 0.961367 -2.807667 -2.672634

C 2.575957 0.719792 -0.833534

C 2.337499 1.981264 -1.468919

C 2.800981 3.179204 -0.859166

C 3.514752 3.101143 0.363216

C 3.735268 1.886204 0.943299

C 3.258265 0.671788 0.367997

C 1.642531 2.085260 -2.704850

C 1.406899 3.306532 -3.283849

C 1.853326 4.497159 -2.662224

C 2.540090 4.429857 -1.478447

O -0.087941 0.243121 -0.946678

C -1.338862 -2.004802 -2.265439

O 3.520991 -0.491873 1.006728

N -2.086778 -0.987282 -1.976747

C -3.412917 -0.840618 -2.023759

H 5.245460 -4.257422 -3.558769

H 6.231253 -2.237183 -2.477627

H 4.776522 -0.514919 -1.496755

H 2.786053 -4.525304 -3.646663

H 0.538595 -3.683245 -3.164451

H 3.869048 4.008391 0.842593

H 1.295794 1.175800 -3.183912

H 0.866594 3.361749 -4.224501

H 1.652822 5.458460 -3.126147

H 2.895761 5.333544 -0.989215

H -1.041608 0.043131 -1.196898

H -1.738281 -2.942956 -2.674702

H 2.690913 -0.868192 1.396486

H -4.083582 -1.573281 -2.474245

C -3.922336 0.499450 -1.839777

O -5.041454 0.887289 -2.135507

O -2.988397 1.303827 -1.233114

C -3.007481 2.733366 -1.418620

C -1.664586 3.176380 -0.844643

H -1.568291 4.266434 -0.892067

H -1.583628 2.857679 0.199639

H -0.837353 2.717404 -1.393974

C -3.080974 3.062365 -2.909671

H -2.282810 2.529822 -3.437652

H -4.045667 2.769000 -3.328198

H -2.936706 4.137769 -3.058058

C -4.162845 3.374464 -0.648408

H -5.115578 2.949556 -0.969208

H -4.037364 3.211159 0.428389

H -4.164900 4.456433 -0.823069

C -5.092279 0.420589 0.947111

C -4.992187 -0.949711 0.661141

C -6.180753 -1.697692 0.624268

C -7.409950 -1.117510 0.908249

C -7.485982 0.238228 1.219509

C -6.322693 1.002956 1.224279

C -3.738641 -1.664487 0.390161

H -4.208479 1.042366 0.883706

H -6.127340 -2.755648 0.379006

H -8.311100 -1.723443 0.880848

H -8.445933 0.698151 1.434771

H -6.370901 2.069839 1.423873

H -3.894767 -2.615627 -0.118177

C -2.485624 -1.521357 0.964162

C -1.533219 -2.581736 0.775034

C -2.069684 -0.416470 1.781071

C -0.278692 -2.575872 1.310589

H -1.883247 -3.443227 0.214198

C -0.812843 -0.309559 2.289002

H -2.800999 0.345026 2.008932

C 0.170370 -1.373414 2.012239

O 1.351033 -1.269821 2.424117

C -0.384501 0.881919 3.148443

C -1.530451 1.884536 3.333463

H -1.861665 2.306050 2.377460

H -2.396623 1.428744 3.827086

H -1.184396 2.712346 3.961664

C 0.040167 0.398045 4.547076

H 0.861353 -0.316397 4.474367

H 0.371229 1.252743 5.150020

H -0.802841 -0.077150 5.062544

C 0.792905 1.616950 2.479451

H 0.590473 1.794313 1.415713

H 0.962577 2.583469 2.971473

H 1.707007 1.029367 2.566199

C 0.628758 -3.811801 1.292548

C 0.038776 -4.914385 0.403888

H -0.943359 -5.247541 0.757517

H -0.058386 -4.571636 -0.633253

H 0.706284 -5.782656 0.414012

C 2.049183 -3.505123 0.780203

H 2.667575 -3.057132 1.557760

H 2.533394 -4.430864 0.445176

H 2.018447 -2.818085 -0.065963

C 0.727365 -4.350684 2.731597

H 1.394993 -5.221208 2.762961

H 1.132277 -3.581406 3.394673

H -0.257304 -4.656545 3.103415

Br 4.628201 1.791479 2.605633

**C-3b-SR-TS3**

Zero-point correction= 0.866337 (Hartree/Particle)

Thermal correction to Energy= 0.916786

Thermal correction to Enthalpy= 0.917730

Thermal correction to Gibbs Free Energy= 0.781219

E(solv) = -4860.65030456 A.U.

C -4.892677 5.143969 -0.665519

C -5.716612 4.147632 -0.094212

C -5.307074 2.837042 -0.052961

C -4.048560 2.440997 -0.580696

C -3.208499 3.454492 -1.128472

C -3.663746 4.798587 -1.165166

C -3.586708 1.081695 -0.552592

C -2.298353 0.799638 -0.969202

C -1.436668 1.821555 -1.508214

C -1.919052 3.106836 -1.592394

C -4.463948 -0.012988 -0.045228

C -5.730689 -0.277851 -0.663598

C -6.618969 -1.226502 -0.084298

C -6.218462 -1.929189 1.079216

C -4.976327 -1.725255 1.604489

C -4.069500 -0.782644 1.040812

C -6.141169 0.370099 -1.862065

C -7.363526 0.108294 -2.426799

C -8.253435 -0.815339 -1.829345

C -7.881992 -1.469737 -0.684566

O -1.837899 -0.462360 -0.832121

C -0.054960 1.522497 -1.848774

O -2.858340 -0.665287 1.623209

N 0.402584 0.327898 -1.665842

C 1.714844 -0.037530 -1.699871

H -5.228572 6.176065 -0.695425

H -6.681090 4.421127 0.323394

H -5.946601 2.088214 0.401482

H -3.006710 5.552193 -1.592958

H -1.270819 3.890180 -1.981583

H -6.890897 -2.649759 1.533908

H -5.463466 1.072395 -2.334906

H -7.648777 0.611107 -3.346002

H -9.219578 -1.010313 -2.284773

H -8.544268 -2.194930 -0.218088

H -0.853616 -0.455178 -1.137073

H 0.580963 2.345123 -2.193442

H -2.200623 -0.564875 0.898006

H 2.453867 0.614908 -2.166379

C 1.946660 -1.476466 -1.880217

O 2.938957 -1.969802 -2.377313

O 0.929780 -2.191030 -1.333392

C 0.927085 -3.636035 -1.347304

C -0.389567 -3.974042 -0.653479

H -0.528512 -5.058660 -0.609697

H -0.393759 -3.577373 0.366534

H -1.228836 -3.527721 -1.194858

C 0.923577 -4.152557 -2.784731

H 0.091898 -3.704668 -3.337832

H 1.860460 -3.908705 -3.286993

H 0.791016 -5.239642 -2.782001

C 2.110199 -4.180632 -0.550012

H 3.053938 -3.980206 -1.058786

H 2.137303 -3.717353 0.442614

H 1.997883 -5.262416 -0.419501

C 1.599801 2.495950 0.461337

C 1.469597 1.139076 0.797058

C 0.309335 0.749263 1.483687

C -0.653936 1.676166 1.871346

C -0.485762 3.025090 1.563027

C 0.642053 3.427581 0.853337

C 2.406711 0.097090 0.336316

H 2.438302 2.817392 -0.148736

H 0.167009 -0.304366 1.717463

H -1.544760 1.337417 2.391683

H -1.244355 3.748899 1.845679

H 0.767805 4.471235 0.578041

H 2.027249 -0.907317 0.526689

C 3.829389 0.205603 0.365383

C 4.607408 -0.947903 0.063485

C 4.545329 1.390992 0.688057

C 5.975390 -0.946202 0.011381

H 4.063495 -1.862589 -0.152783

C 5.913432 1.466525 0.680056

H 3.979579 2.252763 1.016026

C 6.708848 0.288507 0.310488

O 7.961429 0.330978 0.280422

C 6.650648 2.752212 1.065738

C 5.685788 3.885654 1.434217

H 5.022453 4.141422 0.599266

H 5.063801 3.627698 2.298837

H 6.261705 4.782381 1.690730

C 7.557812 2.496171 2.282780

H 8.277858 1.709076 2.056816

H 8.097907 3.413771 2.551278

H 6.955633 2.190159 3.146133

C 7.506755 3.236616 -0.119250

H 6.867221 3.463351 -0.980421

H 8.046767 4.152558 0.155267

H 8.224369 2.467070 -0.405154

C 6.772452 -2.199295 -0.359155

C 5.854579 -3.388683 -0.664115

H 5.253906 -3.671113 0.209304

H 5.173886 -3.163984 -1.493417

H 6.464602 -4.255744 -0.944034

C 7.609169 -1.925552 -1.622740

H 8.294568 -1.094804 -1.451529

H 8.186056 -2.820318 -1.891926

H 6.950288 -1.678698 -2.462915

C 7.703908 -2.597677 0.799164

H 8.283433 -3.489914 0.527258

H 8.389367 -1.781588 1.031300

H 7.116194 -2.833684 1.694107

Br -4.425101 -2.709954 3.120156

**C-3b-SR-TS4**

Zero-point correction= 0.865592 (Hartree/Particle)

Thermal correction to Energy= 0.916385

Thermal correction to Enthalpy= 0.917329

Thermal correction to Gibbs Free Energy= 0.783169

E(solv) = -4860.65148869 A.U.

C -4.264713 4.992730 -0.278669

C -4.931636 3.817308 -0.695112

C -4.235811 2.650262 -0.894688

C -2.831964 2.589621 -0.692862

C -2.162018 3.777427 -0.278727

C -2.908872 4.966883 -0.076203

C -2.073688 1.391123 -0.889205

C -0.701942 1.394461 -0.694659

C -0.018418 2.594829 -0.277500

C -0.764869 3.736675 -0.071328

C -2.743859 0.149600 -1.367122

C -2.498623 -0.348323 -2.687485

C -3.185870 -1.506592 -3.145398

C -4.106539 -2.150907 -2.282192

C -4.319930 -1.659860 -1.027719

C -3.639998 -0.505823 -0.540900

C -1.595328 0.288703 -3.581185

C -1.384815 -0.203819 -4.843563

C -2.063405 -1.362704 -5.289846

C -2.946137 -1.996542 -4.455881

O -0.013012 0.264937 -0.922052

C 1.418118 2.616303 -0.082911

O -3.940675 -0.081132 0.707218

N 2.199755 1.697492 -0.573682

C 3.528479 1.738053 -0.408179

H -4.826840 5.908406 -0.119327

H -6.006095 3.835832 -0.853182

H -4.759146 1.753364 -1.208250

H -2.377709 5.860132 0.245232

H -0.255700 4.646523 0.244192

H -4.628850 -3.042215 -2.615682

H -1.069490 1.175838 -3.246817

H -0.684481 0.296532 -5.505493

H -1.882157 -1.745115 -6.290070

H -3.479669 -2.886582 -4.781042

H 0.957358 0.485808 -0.901143

H 1.837011 3.496186 0.425291

H -3.138849 -0.058671 1.281230

H 3.991893 2.568947 0.127214

C 4.376484 0.911579 -1.217385

O 4.041705 0.028707 -1.995416

O 5.690100 1.258095 -1.000147

C 6.723563 0.773215 -1.871540

C 7.973479 1.479194 -1.349461

H 8.849881 1.193403 -1.940218

H 7.846836 2.564656 -1.405720

H 8.147036 1.204815 -0.304390

C 6.889969 -0.738510 -1.743769

H 7.006585 -1.005376 -0.689016

H 6.015673 -1.256364 -2.138017

H 7.785361 -1.060703 -2.289018

C 6.447106 1.192082 -3.316298

H 5.553088 0.694322 -3.693710

H 6.299650 2.276046 -3.367657

H 7.301816 0.927916 -3.948984

C 4.106350 -1.920294 0.247415

C 4.357388 -1.014983 1.291162

C 5.554539 -1.145403 2.012035

C 6.425854 -2.200752 1.766956

C 6.129469 -3.133248 0.773896

C 4.979319 -2.975175 0.004132

C 3.393853 0.014406 1.702885

H 3.243803 -1.764952 -0.391710

H 5.776416 -0.429018 2.799117

H 7.334533 -2.298086 2.354309

H 6.806324 -3.960865 0.582372

H 4.765717 -3.667041 -0.805171

H 3.825940 0.924204 2.113139

C 2.039459 -0.174725 1.881721

C 1.243408 0.884280 2.449997

C 1.377472 -1.432822 1.647393

C -0.082022 0.752631 2.734193

H 1.768627 1.807361 2.678578

C 0.055549 -1.628170 1.878557

H 1.989186 -2.261595 1.320040

C -0.757397 -0.500312 2.373256

O -1.990826 -0.629431 2.533935

C -0.611434 -2.990174 1.671452

C 0.387460 -4.028885 1.146363

H 0.798947 -3.735407 0.173995

H 1.219986 -4.187447 1.841307

H -0.127647 -4.986907 1.018024

C -1.171696 -3.503275 3.010132

H -1.900512 -2.800483 3.417634

H -1.666164 -4.470694 2.858637

H -0.364144 -3.642701 3.738455

C -1.753878 -2.877019 0.646286

H -1.409841 -2.365102 -0.260399

H -2.109731 -3.877432 0.368202

H -2.595312 -2.325757 1.066151

C -0.877487 1.822007 3.490741

C -0.063387 3.114705 3.629185

H 0.864261 2.959300 4.191214

H 0.187476 3.527193 2.644771

H -0.656403 3.859948 4.169931

C -2.209142 2.171631 2.794191

H -2.991182 1.453920 3.041733

H -2.542532 3.170089 3.102947

H -2.090601 2.181303 1.710004

C -1.180815 1.286306 4.902049

H -1.779534 2.016808 5.460761

H -1.747249 0.353368 4.838072

H -0.254539 1.101331 5.458081

Br -5.498726 -2.562088 0.141331

**C-3b-RR-TS1**

Zero-point correction= 0.864838 (Hartree/Particle)

Thermal correction to Energy= 0.915875

Thermal correction to Enthalpy= 0.916819

Thermal correction to Gibbs Free Energy= 0.779160

E(solv) = -4860.64869841 A.U.

C -2.682420 -5.023460 -2.654548

C -3.805349 -4.509030 -1.968815

C -3.765538 -3.259134 -1.399608

C -2.602656 -2.448702 -1.483256

C -1.488651 -2.952850 -2.215225

C -1.553640 -4.254651 -2.777131

C -2.515973 -1.146286 -0.881197

C -1.397579 -0.368978 -1.116906

C -0.263040 -0.868518 -1.859861

C -0.338184 -2.144775 -2.371567

C -3.609596 -0.639994 0.000640

C -3.985304 -1.377983 1.172283

C -5.108264 -0.970746 1.944326

C -5.824489 0.189149 1.558631

C -5.401223 0.926586 0.491448

C -4.272774 0.544546 -0.289977

C -3.253315 -2.513695 1.615397

C -3.625018 -3.204852 2.739734

C -4.759227 -2.810828 3.487855

C -5.481183 -1.714385 3.095057

O -1.378636 0.894084 -0.631963

C 0.937032 -0.064329 -2.010913

O -3.918238 1.355089 -1.306611

Br -6.331535 2.503175 0.017163

N 0.923281 1.172879 -1.607834

C 1.951314 2.028040 -1.612864

H -2.719860 -6.018204 -3.088840

H -4.710111 -5.105292 -1.892552

H -4.637990 -2.875091 -0.881420

H -0.681840 -4.627377 -3.310233

H 0.513194 -2.542725 -2.921130

H -6.691525 0.505113 2.130195

H -2.380234 -2.824446 1.055188

H -3.036418 -4.059735 3.059823

H -5.046706 -3.369418 4.373628

H -6.348121 -1.386277 3.663835

H -0.481758 1.310232 -0.923931

H 1.816891 -0.517248 -2.478521

H -2.934985 1.379404 -1.317060

H 2.887804 1.800385 -2.120467

C 1.706024 3.412806 -1.277597

O 2.487606 4.329945 -1.474584

O 0.497855 3.568792 -0.658443

C -0.035239 4.882292 -0.399152

C -1.429401 4.585009 0.146829

H -1.940718 5.515929 0.412427

H -2.030282 4.053194 -0.597255

H -1.358833 3.953367 1.037296

C 0.806421 5.608075 0.648530

H 0.846055 5.016037 1.568623

H 1.823997 5.759697 0.285299

H 0.349690 6.577302 0.879810

C -0.137050 5.687106 -1.695124

H 0.852651 5.939601 -2.077096

H -0.673438 5.103593 -2.450455

H -0.698554 6.609157 -1.509645

C 4.700796 2.821527 0.087823

C 3.583612 2.464075 0.856575

C 3.198690 3.319749 1.896355

C 3.927619 4.466104 2.193566

C 5.052318 4.790541 1.441224

C 5.428844 3.966852 0.382482

C 2.756085 1.266686 0.620480

H 4.968412 2.222685 -0.777370

H 2.316981 3.070335 2.481328

H 3.608101 5.111124 3.007374

H 5.619747 5.689294 1.663992

H 6.282676 4.231085 -0.234246

H 1.709963 1.391671 0.899178

C 3.193914 -0.039421 0.479342

C 4.578484 -0.391096 0.303784

C 2.256688 -1.127149 0.625481

C 5.016517 -1.673919 0.239342

H 5.293521 0.419818 0.258011

C 2.627561 -2.438241 0.626834

H 1.217917 -0.849903 0.790688

C 4.043313 -2.783694 0.388925

O 4.422968 -3.965296 0.346691

C 1.647060 -3.579142 0.912877

C 0.231476 -3.062087 1.186264

H 0.194450 -2.404583 2.063034

H -0.177634 -2.517202 0.328349

H -0.424011 -3.919169 1.383341

C 1.571835 -4.544007 -0.284081

H 2.547937 -4.985780 -0.487281

H 0.851130 -5.344265 -0.073031

H 1.225088 -4.010217 -1.174957

C 2.105243 -4.351501 2.165108

H 2.139028 -3.683366 3.033493

H 1.391640 -5.155907 2.383794

H 3.093622 -4.787267 2.015618

C 6.496156 -2.017970 0.057415

C 7.360697 -0.759143 -0.080768

H 7.060027 -0.153243 -0.943385

H 7.314163 -0.129049 0.814534

H 8.405540 -1.055119 -0.227380

C 7.010219 -2.807382 1.274593

H 6.453587 -3.736957 1.395293

H 8.073950 -3.043027 1.143000

H 6.904907 -2.209621 2.187289

C 6.686738 -2.855132 -1.220627

H 7.749928 -3.091917 -1.355041

H 6.119904 -3.784247 -1.160970

H 6.351542 -2.290941 -2.098652

-------------------------------------------

**C-3b-RR-TS2**

Zero-point correction= 0.865984 (Hartree/Particle)

Thermal correction to Energy= 0.917077

Thermal correction to Enthalpy= 0.918021

Thermal correction to Gibbs Free Energy= 0.780935

E(solv) = -4860.64914637 A.U.

C -2.863715 -5.119937 -1.642557

C -3.900904 -4.513448 -0.899136

C -3.820887 -3.189371 -0.539017

C -2.700148 -2.396539 -0.904426

C -1.652614 -3.012578 -1.650200

C -1.765200 -4.381375 -2.004214

C -2.582611 -1.011194 -0.550606

C -1.447258 -0.293428 -0.901325

C -0.364029 -0.927569 -1.628254

C -0.512034 -2.249088 -1.993056

C -3.674234 -0.314623 0.188702

C -3.490712 0.182073 1.514717

C -4.558955 0.848558 2.175602

C -5.796303 1.003806 1.503927

C -5.955472 0.522363 0.236394

C -4.895399 -0.137070 -0.440160

C -2.263602 0.025429 2.212641

C -2.111835 0.505241 3.487849

C -3.176057 1.173730 4.140388

C -4.372855 1.339212 3.494463

O -1.368326 0.989446 -0.540829

C 0.852450 -0.200008 -1.936539

O -5.144019 -0.573805 -1.695234

N 0.938623 1.063176 -1.631383

C 2.005466 1.845840 -1.807203

H -2.934036 -6.168095 -1.917643

H -4.766641 -5.099182 -0.604039

H -4.615760 -2.736727 0.046545

H -0.950346 -4.837495 -2.562128

H 0.291327 -2.731393 -2.547766

H -6.621005 1.506419 1.999510

H -1.444570 -0.482161 1.715808

H -1.163919 0.372463 4.001227

H -3.040440 1.553189 5.148786

H -5.201940 1.848585 3.979973

H -0.480338 1.343479 -0.877693

H 1.668420 -0.736527 -2.432520

H -4.329680 -0.987636 -2.030415

H 2.870570 1.526371 -2.388008

C 1.897170 3.258325 -1.519281

O 2.696428 4.111183 -1.873132

O 0.806150 3.525888 -0.740287

C 0.366412 4.880449 -0.526849

C -0.955385 4.695996 0.213778

H -1.397675 5.668819 0.452986

H -1.657404 4.123416 -0.399360

H -0.793191 4.141238 1.142820

C 1.367271 5.641971 0.339801

H 1.490067 5.131060 1.300269

H 2.338084 5.697341 -0.155354

H 0.992502 6.653812 0.533334

C 0.128026 5.586022 -1.862469

H 1.068822 5.759984 -2.385664

H -0.522188 4.970099 -2.492124

H -0.369799 6.545782 -1.686479

C 5.026596 2.438971 -0.548072

C 3.987142 2.304520 0.384112

C 3.819492 3.320067 1.333803

C 4.682997 4.408781 1.384488

C 5.727569 4.512714 0.471505

C 5.889343 3.525895 -0.498928

C 3.025697 1.187888 0.401584

H 5.126513 1.709145 -1.345692

H 3.000707 3.242826 2.044631

H 4.530026 5.182119 2.132169

H 6.397927 5.366585 0.500447

H 6.678333 3.616549 -1.239611

H 2.037236 1.458677 0.771485

C 3.310264 -0.164691 0.353927

C 4.626439 -0.684295 0.085870

C 2.289064 -1.123082 0.706142

C 4.924746 -2.006994 0.127088

H 5.411453 0.032732 -0.115048

C 2.523560 -2.460148 0.821047

H 1.304668 -0.715189 0.925478

C 3.868180 -2.980519 0.499289

O 4.126356 -4.192586 0.568578

C 1.470080 -3.449853 1.329881

C 0.139476 -2.754013 1.633854

H 0.253068 -1.997361 2.420315

H -0.280402 -2.274111 0.743015

H -0.589558 -3.493037 1.984627

C 1.202120 -4.551643 0.289247

H 2.106116 -5.128975 0.094447

H 0.415067 -5.223735 0.654049

H 0.851753 -4.107021 -0.646844

C 1.968869 -4.095923 2.636733

H 2.135402 -3.329425 3.402588

H 1.213692 -4.796069 3.015431

H 2.900980 -4.638599 2.471158

C 6.334816 -2.531092 -0.153318

C 7.299992 -1.398391 -0.521588

H 6.969454 -0.859409 -1.417048

H 7.411326 -0.674230 0.293166

H 8.288765 -1.821845 -0.730455

C 6.896241 -3.236924 1.094232

H 6.266454 -4.080163 1.378440

H 7.910880 -3.602483 0.890743

H 6.950132 -2.535608 1.934856

C 6.300555 -3.516860 -1.335931

H 7.313152 -3.887067 -1.541074

H 5.650448 -4.363307 -1.113731

H 5.933659 -3.014894 -2.238726

Br -7.615432 0.728704 -0.646616

**C-3b-RR-TS3**

Zero-point correction= 0.865393 (Hartree/Particle)

Thermal correction to Energy= 0.916206

Thermal correction to Enthalpy= 0.917150

Thermal correction to Gibbs Free Energy= 0.781423

E(solv) = -4860.64553748 A.U.

C 2.745378 5.481630 -0.528259

C 3.805345 4.571176 -0.306947

C 3.552846 3.287454 0.107993

C 2.224097 2.837770 0.328848

C 1.158799 3.757319 0.105353

C 1.451317 5.076971 -0.325532

C 1.924710 1.507715 0.755261

C 0.610455 1.121110 0.959493

C -0.471469 2.041415 0.724930

C -0.169626 3.318556 0.302229

C 3.021890 0.540013 1.030154

C 3.328133 0.151061 2.372512

C 4.404390 -0.745231 2.620062

C 5.150954 -1.246020 1.524923

C 4.832428 -0.865416 0.253830

C 3.760600 0.032849 -0.023910

C 2.596277 0.642762 3.487101

C 2.916368 0.262560 4.765389

C 3.985257 -0.633009 5.005984

C 4.711891 -1.123488 3.952953

O 0.359242 -0.117238 1.408747

C -1.867553 1.671130 0.922450

O 3.538048 0.381448 -1.309438

Br 5.772487 -1.606073 -1.207483

N -2.203649 0.574343 1.530697

C -3.455130 0.102236 1.771952

H 2.959443 6.493141 -0.861182

H 4.830347 4.889774 -0.474267

H 4.371684 2.593418 0.265675

H 0.622087 5.760083 -0.496220

H -0.985300 4.018292 0.122772

H 5.962323 -1.946372 1.698070

H 1.774549 1.327954 3.308041

H 2.341227 0.648517 5.602006

H 4.225335 -0.928787 6.023175

H 5.535989 -1.813565 4.118380

H -0.625827 -0.188073 1.548006

H -2.600800 2.401947 0.580211

H 2.660650 0.049861 -1.636474

H -3.509880 -0.721687 2.475406

C -4.714975 0.782847 1.578207

O -5.774132 0.466897 2.112183

O -4.644185 1.783042 0.648551

C -5.826596 2.485704 0.215474

C -5.273106 3.495507 -0.787569

H -6.087931 4.070938 -1.238460

H -4.586745 4.188680 -0.290557

H -4.724282 2.978437 -1.581209

C -6.788715 1.524986 -0.481612

H -6.279745 1.024178 -1.312464

H -7.157317 0.772804 0.216652

H -7.638146 2.084378 -0.889068

C -6.491908 3.213233 1.383245

H -6.932610 2.504115 2.083583

H -5.747042 3.817333 1.911499

H -7.270542 3.883221 1.001726

C -5.300869 -2.518637 1.072479

C -4.296630 -2.491384 0.092806

C -3.998598 -3.690212 -0.576627

C -4.645416 -4.877978 -0.249401

C -5.617879 -4.894878 0.745271

C -5.945198 -3.707439 1.396377

C -3.640027 -1.224351 -0.317459

H -5.572278 -1.604607 1.591977

H -3.267645 -3.681140 -1.378992

H -4.391384 -5.789315 -0.783409

H -6.123783 -5.820977 1.003401

H -6.710763 -3.702821 2.167467

H -4.313177 -0.408781 -0.575787

C -2.351920 -1.146408 -0.838057

C -1.359594 -2.152777 -0.607042

C -1.966597 -0.018684 -1.629386

C -0.115623 -2.109496 -1.160905

H -1.632307 -2.974067 0.045100

C -0.736600 0.096131 -2.217270

H -2.728822 0.740564 -1.784401

C 0.263834 -0.940019 -1.968431

O 1.423039 -0.846968 -2.451339

C -0.403425 1.227901 -3.195196

C -1.501302 2.300194 -3.190673

H -2.469166 1.901450 -3.515576

H -1.622125 2.737333 -2.191633

H -1.226241 3.103464 -3.882849

C 0.933186 1.920451 -2.859146

H 1.783208 1.358483 -3.245680

H 0.954422 2.927276 -3.295061

H 1.060456 2.020684 -1.780034

C -0.311597 0.634015 -4.612222

H -1.272478 0.204709 -4.919051

H -0.035941 1.414826 -5.332982

H 0.451486 -0.148279 -4.640777

C 0.883875 -3.255865 -0.985061

C 0.317464 -4.362871 -0.087370

H 0.123404 -3.997448 0.927294

H -0.612815 -4.783606 -0.487354

H 1.048624 -5.175632 -0.016995

C 1.182862 -3.871109 -2.364360

H 1.585221 -3.114598 -3.040609

H 1.919601 -4.677621 -2.260660

H 0.270981 -4.293899 -2.803096

C 2.195413 -2.765474 -0.349919

H 2.820742 -3.623170 -0.069403

H 2.759789 -2.157104 -1.056419

H 1.991406 -2.173322 0.550080

-------------------------------------------------

**C-3b-RR-TS4**

Zero-point correction= 0.866097 (Hartree/Particle)

Thermal correction to Energy= 0.917119

Thermal correction to Enthalpy= 0.918063

Thermal correction to Gibbs Free Energy= 0.782091

E(solv) = -4860.64418252 A.U.

C -2.798382 -5.153703 -1.624049

C -3.835909 -4.559624 -0.870349

C -3.776153 -3.229945 -0.529060

C -2.678771 -2.419823 -0.917920

C -1.631531 -3.021669 -1.673129

C -1.722805 -4.395875 -2.013452

C -2.572803 -1.038695 -0.563621

C -1.453438 -0.304980 -0.921035

C -0.373466 -0.918515 -1.668929

C -0.509402 -2.240417 -2.037372

C -3.661825 -0.351538 0.184065

C -3.466018 0.142668 1.508762

C -4.520626 0.812271 2.190570

C -5.765431 0.976033 1.540448

C -5.931987 0.492484 0.274570

C -4.891247 -0.171555 -0.428509

C -2.228909 -0.020894 2.190709

C -2.056791 0.454710 3.463709

C -3.108548 1.127286 4.134759

C -4.313546 1.299619 3.508764

O -1.383943 0.979386 -0.547597

C 0.827189 -0.171829 -1.995430

O -5.066759 -0.639958 -1.685355

N 0.908711 1.088680 -1.677982

C 1.951014 1.895595 -1.883871

H -2.851031 -6.206525 -1.886030

H -4.685226 -5.159784 -0.555784

H -4.572940 -2.783467 0.058649

H -0.908368 -4.841133 -2.581135

H 0.291177 -2.709634 -2.607583

H -6.582098 1.481830 2.045578

H -1.421284 -0.532211 1.679976

H -1.102215 0.316357 3.962954

H -2.955440 1.503432 5.141911

H -5.133175 1.811498 4.007458

H -0.507286 1.345772 -0.895122

H 1.637559 -0.693978 -2.516206

H -5.967945 -0.433281 -1.979047

H 2.818412 1.590676 -2.469388

C 1.849792 3.288152 -1.535077

O 2.639535 4.162026 -1.865229

O 0.770583 3.525201 -0.721249

C 0.299060 4.865396 -0.499298

C -1.012605 4.645275 0.250297

H -1.476429 5.605437 0.500559

H -1.704726 4.058695 -0.360838

H -0.828737 4.087954 1.173878

C 1.284726 5.652207 0.362911

H 1.419012 5.148958 1.326031

H 2.253250 5.722876 -0.134693

H 0.890148 6.657516 0.552237

C 0.033287 5.570701 -1.830133

H 0.966685 5.766692 -2.358966

H -0.606457 4.941153 -2.457114

H -0.485367 6.518277 -1.647394

C 4.979690 2.463377 -0.549353

C 3.941924 2.325975 0.384491

C 3.749008 3.357526 1.311674

C 4.590205 4.464260 1.342152

C 5.634459 4.571362 0.429250

C 5.818430 3.569385 -0.521625

C 3.009395 1.188282 0.423882

H 5.093891 1.719391 -1.331809

H 2.929180 3.278099 2.020933

H 4.418887 5.249848 2.072988

H 6.286152 5.439978 0.441636

H 6.605396 3.662990 -1.264110

H 1.999972 1.445579 0.743458

C 3.315355 -0.152901 0.370062

C 4.649714 -0.648757 0.133303

C 2.298051 -1.132205 0.684938

C 4.966847 -1.965516 0.164777

H 5.426593 0.084569 -0.038083

C 2.551479 -2.464082 0.795366

H 1.300662 -0.741121 0.872889

C 3.914327 -2.959052 0.501235

O 4.188959 -4.166011 0.562332

C 1.499186 -3.478072 1.255661

C 0.155913 -2.802065 1.547882

H 0.248299 -2.067854 2.358364

H -0.249086 -2.300178 0.662881

H -0.574512 -3.556473 1.861127

C 1.269646 -4.552125 0.177285

H 2.182181 -5.120894 -0.004759

H 0.473984 -5.236271 0.497788

H 0.946863 -4.082336 -0.756350

C 1.972919 -4.153518 2.556919

H 2.116701 -3.405214 3.345188

H 1.212380 -4.865846 2.900341

H 2.911430 -4.687671 2.401584

C 6.388816 -2.467640 -0.093556

C 7.344326 -1.317235 -0.431063

H 7.022078 -0.771308 -1.325258

H 7.431128 -0.602263 0.394661

H 8.342392 -1.724444 -0.627603

C 6.936755 -3.180078 1.156114

H 6.316095 -4.037240 1.417860

H 7.960974 -3.526497 0.967912

H 6.962782 -2.489109 2.006526

C 6.389334 -3.440410 -1.287776

H 7.410958 -3.792647 -1.479103

H 5.748351 -4.299321 -1.086885

H 6.030491 -2.933673 -2.191061

Br -7.612961 0.713081 -0.596517

----------------------------------------------

**C-3b-SS-TS1**

Zero-point correction= 0.865698 (Hartree/Particle)

Thermal correction to Energy= 0.915982

Thermal correction to Enthalpy= 0.916926

Thermal correction to Gibbs Free Energy= 0.781265

E(solv) = -4860.64955320 A.U.

C 1.821041 -5.254007 -0.604177

C 3.042129 -4.619991 -0.288336

C 3.171220 -3.254487 -0.381454

C 2.084652 -2.442011 -0.796674

C 0.850353 -3.085374 -1.098398

C 0.747993 -4.495491 -0.998229

C 2.160633 -1.006917 -0.844082

C 1.008553 -0.284453 -1.091845

C -0.230006 -0.929061 -1.456570

C -0.274945 -2.302613 -1.453117

C 3.435327 -0.290608 -0.547929

C 4.609505 -0.530652 -1.336058

C 5.848562 0.066665 -0.969740

C 5.896443 0.930665 0.152179

C 4.753662 1.209277 0.842425

C 3.503058 0.621070 0.498586

C 4.587135 -1.346728 -2.502027

C 5.725993 -1.573218 -3.232248

C 6.959833 -1.000075 -2.844389

C 7.013052 -0.193794 -1.738201

O 1.050642 1.062322 -0.982096

C -1.410904 -0.139973 -1.770335

O 2.436180 0.971941 1.242204

Br 4.803337 2.405717 2.305201

N -1.368888 1.140550 -1.572125

C -2.392263 2.012698 -1.648065

H 1.728570 -6.332286 -0.515531

H 3.887341 -5.215404 0.045349

H 4.109518 -2.783207 -0.108804

H -0.211283 -4.956545 -1.218219

H -1.208502 -2.803135 -1.696699

H 6.836652 1.388923 0.443220

H 3.645277 -1.784886 -2.813848

H 5.679349 -2.195823 -4.120883

H 7.855290 -1.192926 -3.427824

H 7.948032 0.268859 -1.430732

H 0.081479 1.397026 -1.153073

H -2.291176 -0.653026 -2.167277

H 1.679489 1.087061 0.620044

H -3.254552 1.837426 -2.288829

C -2.026938 3.402180 -1.380226

O -2.676389 4.390621 -1.690551

O -0.873233 3.462376 -0.666973

C -0.262594 4.718121 -0.303775

C 1.003683 4.278919 0.427838

H 1.576962 5.152042 0.755397

H 0.748444 3.678615 1.306692

H 1.631770 3.667641 -0.227317

C 0.092997 5.515855 -1.557123

H 0.708927 4.901322 -2.221315

H -0.807839 5.825711 -2.088805

H 0.669270 6.403784 -1.275928

C -1.179957 5.504246 0.631026

H -2.086431 5.817384 0.111557

H -1.455988 4.881586 1.488453

H -0.652236 6.388481 1.004723

C -6.035293 0.863041 0.381397

C -4.992376 1.675576 -0.094178

C -5.342567 2.834993 -0.803410

C -6.675250 3.142670 -1.057368

C -7.696204 2.309879 -0.606291

C -7.367581 1.169975 0.120886

C -3.575940 1.360928 0.250588

H -5.794999 -0.006766 0.984167

H -4.566590 3.500956 -1.168537

H -6.914395 4.043256 -1.616131

H -8.735570 2.551870 -0.810068

H -8.149445 0.518298 0.501247

H -3.052228 2.154899 0.781602

C -3.125902 0.059048 0.506766

C -2.021926 -0.161080 1.394534

C -3.755285 -1.107852 -0.039722

C -1.608522 -1.398704 1.791715

H -1.547360 0.728977 1.802269

C -3.386349 -2.380924 0.283261

H -4.568041 -0.943487 -0.741042

C -2.250893 -2.594879 1.205820

O -1.869631 -3.745204 1.491702

C -4.163212 -3.601793 -0.220704

C -5.324323 -3.202039 -1.137900

H -6.043824 -2.551564 -0.626894

H -4.969807 -2.680281 -2.034574

H -5.856620 -4.103362 -1.462715

C -3.257417 -4.564084 -1.008140

H -2.389520 -4.842694 -0.408190

H -3.816752 -5.470610 -1.273363

H -2.922354 -4.099358 -1.943697

C -4.756804 -4.346178 0.990637

H -5.431111 -3.685183 1.547586

H -5.333334 -5.216979 0.652713

H -3.962267 -4.682057 1.658478

C -0.544172 -1.598815 2.874439

C -0.065429 -0.264271 3.458319

H 0.422564 0.361022 2.703977

H -0.889303 0.301220 3.910040

H 0.677055 -0.460234 4.239845

C -1.142233 -2.420271 4.032544

H -1.453006 -3.407639 3.689956

H -0.392274 -2.537580 4.824875

H -2.009948 -1.903328 4.460256

C 0.684948 -2.319505 2.299184

H 1.399478 -2.538380 3.103467

H 0.405570 -3.255135 1.809785

H 1.187640 -1.670615 1.574583

--------------------------------------------------

**C-3b-SS-TS2**

Zero-point correction= 0.864975 (Hartree/Particle)

Thermal correction to Energy= 0.914648

Thermal correction to Enthalpy= 0.915593

Thermal correction to Gibbs Free Energy= 0.782793

E(solv) = -4860.65041925 A.U.

C -6.037767 -4.009052 -1.697701

C -6.494924 -2.672467 -1.659780

C -5.736591 -1.688110 -1.073329

C -4.474862 -1.978156 -0.488624

C -4.004243 -3.322180 -0.558451

C -4.814958 -4.319025 -1.161121

C -3.654130 -0.974029 0.131308

C -2.378013 -1.312273 0.543207

C -1.894356 -2.668578 0.474500

C -2.723032 -3.632988 -0.050324

C -4.160728 0.419539 0.301766

C -5.365930 0.666697 1.041166

C -5.928723 1.973042 1.064899

C -5.264814 3.025936 0.387651

C -4.074591 2.791143 -0.235470

C -3.488237 1.493041 -0.266132

C -6.031129 -0.353814 1.776273

C -7.191594 -0.094255 2.460788

C -7.761428 1.200044 2.453766

C -7.135634 2.210516 1.772467

O -1.564211 -0.338060 1.006502

C -0.541688 -2.998301 0.886703

O -2.297979 1.369311 -0.887028

N 0.270533 -2.058042 1.250166

C 1.598575 -2.220862 1.482343

H -6.647107 -4.779651 -2.160552

H -7.452327 -2.417921 -2.105074

H -6.097458 -0.665343 -1.065662

H -4.435655 -5.337771 -1.195956

H -2.362946 -4.658109 -0.119185

H -5.692041 4.023937 0.388964

H -5.599248 -1.348326 1.800001

H -7.674653 -0.891020 3.018643

H -8.682884 1.391214 2.995457

H -7.547128 3.217005 1.767615

H -0.650709 -0.773410 1.195688

H -0.224606 -4.045595 0.818711

H -1.754445 0.752267 -0.344229

H 1.969609 -3.209611 1.755508

C 2.286219 -1.107961 2.139910

O 3.257628 -1.233431 2.860280

O 1.742228 0.069418 1.771745

C 2.187146 1.323947 2.346946

C 1.378099 2.351014 1.561281

H 1.577094 3.359594 1.936787

H 1.659832 2.310295 0.503196

H 0.306060 2.145206 1.646247

C 1.816502 1.346004 3.827464

H 0.741738 1.176797 3.950796

H 2.366888 0.572202 4.367360

H 2.068196 2.321385 4.256841

C 3.679260 1.549819 2.125926

H 4.279583 0.867646 2.726862

H 3.928192 1.395361 1.072468

H 3.927410 2.584339 2.390393

C 1.003032 -3.336649 -1.917299

C 1.419831 -2.135903 -1.331657

C 0.596441 -1.007623 -1.484421

C -0.571444 -1.073545 -2.235588

C -0.961729 -2.276179 -2.826711

C -0.172233 -3.408393 -2.662853

C 2.620545 -2.133469 -0.479223

H 1.612474 -4.227763 -1.781445

H 0.856645 -0.091659 -0.959011

H -1.201736 -0.194522 -2.335534

H -1.891165 -2.329902 -3.385851

H -0.476087 -4.354587 -3.102434

H 2.982386 -3.135400 -0.252790

C 3.646628 -1.151867 -0.521883

C 4.840771 -1.397121 0.216440

C 3.603345 0.037709 -1.302629

C 5.921368 -0.559855 0.211182

H 4.853479 -2.296804 0.825982

C 4.651305 0.917732 -1.374983

H 2.722236 0.217902 -1.905845

C 5.884144 0.655429 -0.612962

O 6.863449 1.432708 -0.672227

C 4.579079 2.186350 -2.228956

C 3.239198 2.314628 -2.962898

H 3.070261 1.481074 -3.654222

H 2.393716 2.357433 -2.265980

H 3.235162 3.241212 -3.548291

C 4.732798 3.422566 -1.323773

H 5.677590 3.381956 -0.780448

H 4.704017 4.338595 -1.928300

H 3.908957 3.467779 -0.600660

C 5.694291 2.180715 -3.289932

H 5.567619 1.328603 -3.968294

H 5.648595 3.100559 -3.887690

H 6.672203 2.110313 -2.812747

C 7.173416 -0.850689 1.041801

C 7.021029 -2.133270 1.867749

H 6.160831 -2.073408 2.544011

H 6.897897 -3.015149 1.227659

H 7.923505 -2.284113 2.472020

C 8.390500 -1.018558 0.115546

H 8.555839 -0.107913 -0.461594

H 9.288412 -1.237700 0.708534

H 8.228145 -1.852662 -0.577385

C 7.432588 0.303960 2.026151

H 8.353379 0.111248 2.592794

H 7.534277 1.248720 1.490869

H 6.605927 0.384157 2.740787

Br -3.171160 4.206866 -1.098387

--------------------------------------------

**C-3b-SS-TS3**

Zero-point correction= 0.866343 (Hartree/Particle)

Thermal correction to Energy= 0.916919

Thermal correction to Enthalpy= 0.917863

Thermal correction to Gibbs Free Energy= 0.781979

E(solv) = -4860.64806208 A.U.

C -5.876694 -4.298119 -2.047826

C -6.501839 -3.043910 -1.862591

C -5.841147 -2.017843 -1.231843

C -4.515194 -2.181901 -0.749567

C -3.879033 -3.439380 -0.963606

C -4.591339 -4.483831 -1.609071

C -3.795702 -1.132171 -0.085747

C -2.462918 -1.324494 0.233946

C -1.817112 -2.596689 0.034147

C -2.542742 -3.615513 -0.540162

C -4.471447 0.155134 0.250569

C -5.623907 0.168257 1.104883

C -6.337729 1.381296 1.313565

C -5.870705 2.573354 0.706887

C -4.728621 2.556467 -0.038119

C -4.002244 1.353476 -0.267107

C -6.089526 -0.997573 1.774426

C -7.203419 -0.959022 2.574419

C -7.923786 0.244665 2.754993

C -7.492486 1.389550 2.138511

O -1.767118 -0.286749 0.744514

C -0.425814 -2.800003 0.402857

O -2.888837 1.442904 -1.019916

N 0.287282 -1.833563 0.884678

C 1.623779 -1.946029 1.115422

H -6.409646 -5.103310 -2.544666

H -7.512439 -2.887112 -2.228205

H -6.330402 -1.057630 -1.107673

H -4.088014 -5.436771 -1.755236

H -2.065452 -4.581582 -0.696179

H -6.406543 3.504612 0.861598

H -5.541835 -1.925621 1.652911

H -7.531982 -1.862502 3.079600

H -8.806117 0.259656 3.387602

H -8.021010 2.329759 2.276382

H -0.814939 -0.599295 0.903108

H 0.000769 -3.797125 0.232201

H -2.214852 0.839225 -0.640435

H 2.046957 -2.940758 1.263638

C 2.289903 -0.913898 1.905591

O 3.342325 -1.097946 2.487086

O 1.648533 0.273481 1.839415

C 2.220076 1.450865 2.462900

C 1.154045 2.514402 2.215591

H 1.479364 3.476063 2.624695

H 0.976970 2.633536 1.142159

H 0.210042 2.227201 2.688908

C 2.405713 1.237111 3.965125

H 1.477146 0.855921 4.403205

H 3.211437 0.531662 4.166456

H 2.640052 2.197167 4.437618

C 3.524708 1.824563 1.766052

H 4.263070 1.021730 1.838832

H 3.343086 2.022951 0.704655

H 3.938415 2.732699 2.219190

C 0.992723 0.228989 -1.391369

C 1.424577 -1.093084 -1.578131

C 0.650118 -1.935432 -2.383508

C -0.510062 -1.474415 -3.002182

C -0.918221 -0.157398 -2.820463

C -0.154787 0.695089 -2.017646

C 2.576920 -1.636991 -0.838016

H 1.540369 0.865459 -0.702900

H 0.957708 -2.970765 -2.512875

H -1.108632 -2.152328 -3.603849

H -1.836742 0.205732 -3.270920

H -0.482836 1.718688 -1.856711

H 2.633571 -2.725218 -0.865540

C 3.823747 -0.964769 -0.722336

C 4.115560 0.285807 -1.336755

C 4.867996 -1.570828 0.032954

C 5.330336 0.907905 -1.222435

H 3.353131 0.725314 -1.969120

C 6.088740 -0.990407 0.234835

H 4.632581 -2.517058 0.513049

C 6.390592 0.296362 -0.407385

O 7.508556 0.844212 -0.274831

C 7.135450 -1.616752 1.157222

C 6.635676 -2.927686 1.775029

H 5.718431 -2.768436 2.352943

H 6.437267 -3.686828 1.008760

H 7.403002 -3.326824 2.448891

C 8.430965 -1.912298 0.382186

H 8.827228 -0.995524 -0.056078

H 9.181969 -2.346164 1.055725

H 8.237390 -2.634298 -0.420255

C 7.429100 -0.644802 2.314780

H 6.510895 -0.460255 2.884982

H 8.174137 -1.080252 2.994015

H 7.808054 0.302722 1.929207

C 5.624935 2.234896 -1.926133

C 4.419010 2.743581 -2.724216

H 4.130329 2.046145 -3.518751

H 3.545889 2.907650 -2.080666

H 4.672506 3.701067 -3.193929

C 5.979470 3.312647 -0.884695

H 6.841150 3.001269 -0.293824

H 6.210206 4.260520 -1.388954

H 5.131491 3.485932 -0.211462

C 6.800254 2.063464 -2.904877

H 7.018299 3.017326 -3.403107

H 7.689783 1.726385 -2.371076

H 6.547759 1.326208 -3.676091

Br -4.072148 4.162998 -0.784895

----------------------------------------------

**C-3b-SS-TS4**

Zero-point correction= 0.865949 (Hartree/Particle)

Thermal correction to Energy= 0.916601

Thermal correction to Enthalpy= 0.917545

Thermal correction to Gibbs Free Energy= 0.781559

E(solv) = -4860.64346148 A.U.

C 3.359524 4.694249 -2.460423

C 4.348724 3.723095 -2.183057

C 3.998444 2.457388 -1.782113

C 2.635371 2.083319 -1.633778

C 1.642471 3.055217 -1.950310

C 2.035431 4.358952 -2.348282

C 2.232989 0.770801 -1.216149

C 0.886896 0.453885 -1.207230

C -0.120662 1.426524 -1.522722

C 0.277341 2.697549 -1.864279

C 3.249432 -0.260065 -0.861134

C 4.162373 -0.044229 0.222640

C 5.202998 -0.982847 0.465010

C 5.299792 -2.138313 -0.349983

C 4.374005 -2.362401 -1.325801

C 3.321566 -1.440501 -1.586134

C 4.040292 1.064228 1.104873

C 4.918527 1.237199 2.144169

C 5.974864 0.320368 2.357927

C 6.108124 -0.768115 1.536551

O 0.514562 -0.821096 -0.950094

C -1.541305 1.094546 -1.490474

O 2.445256 -1.758077 -2.563613

N -1.929592 -0.136931 -1.384767

C -3.237164 -0.543558 -1.303194

H 3.650353 5.694671 -2.766670

H 5.398918 3.979256 -2.289914

H 4.770472 1.722542 -1.582186

H 1.256761 5.086635 -2.564865

H -0.479920 3.443376 -2.100317

H 6.091171 -2.860106 -0.172965

H 3.207857 1.748765 0.980014

H 4.780458 2.072002 2.824424

H 6.663598 0.470384 3.184174

H 6.898127 -1.497360 1.699882

H -0.500214 -0.847972 -1.015102

H -2.246504 1.916859 -1.612319

H 1.552370 -1.529804 -2.227144

H -3.373062 -1.618964 -1.350081

C -4.314433 0.252321 -1.860942

O -4.352462 1.473128 -1.940679

O -5.353315 -0.538822 -2.269959

C -6.639938 0.041787 -2.572837

C -7.516042 -1.182964 -2.823295

H -8.531993 -0.876143 -3.091309

H -7.101315 -1.783960 -3.638015

H -7.563776 -1.805095 -1.923403

C -7.172828 0.830796 -1.376756

H -7.139724 0.208694 -0.475185

H -6.583539 1.732657 -1.207523

H -8.214998 1.113817 -1.561173

C -6.555616 0.901093 -3.833352

H -5.934091 1.779228 -3.656534

H -6.121445 0.316608 -4.651008

H -7.560992 1.219306 -4.130820

C -4.260577 -1.634923 2.741557

C -4.388575 -1.252132 1.396307

C -5.328703 -1.939552 0.615575

C -6.093435 -2.971651 1.150410

C -5.942426 -3.348367 2.482112

C -5.020705 -2.671444 3.274740

C -3.610767 -0.079193 0.888773

H -3.562502 -1.098509 3.375970

H -5.452910 -1.675652 -0.428330

H -6.806545 -3.490660 0.514930

H -6.536793 -4.157447 2.897224

H -4.893221 -2.943507 4.318848

H -4.219645 0.764891 0.561053

C -2.360272 0.256749 1.431829

C -1.898537 1.607927 1.467655

C -1.464261 -0.746215 1.917804

C -0.710670 1.976686 2.039880

H -2.566573 2.359059 1.052010

C -0.263514 -0.463116 2.497034

H -1.779894 -1.778730 1.803740

C 0.156957 0.947546 2.633656

O 1.204263 1.251414 3.240688

C 0.644147 -1.564523 3.047568

C 0.068430 -2.960514 2.783400

H -0.907911 -3.102184 3.262625

H -0.043333 -3.149230 1.709732

H 0.753295 -3.714250 3.187957

C 2.026856 -1.496571 2.379705

H 2.509555 -0.541615 2.590024

H 2.667389 -2.306208 2.754132

H 1.927882 -1.611019 1.293903

C 0.793210 -1.393979 4.570584

H -0.183587 -1.480483 5.062327

H 1.447930 -2.178429 4.971937

H 1.221176 -0.417881 4.802744

C -0.279929 3.441447 2.154188

C -1.206659 4.364759 1.354625

H -1.221925 4.085993 0.293886

H -2.235305 4.342828 1.731364

H -0.847286 5.397364 1.430292

C -0.325168 3.871756 3.631153

H 0.321880 3.225435 4.228024

H 0.016023 4.910491 3.735383

H -1.348534 3.806959 4.018932

C 1.150123 3.642123 1.618363

H 1.381258 4.713545 1.555657

H 1.874050 3.156343 2.272702

H 1.243630 3.216111 0.614247

Br 4.463540 -3.939294 -2.367306

---------------------------------------------

**C-4f-SS-TS1**

Zero-point correction= 1.056658 (Hartree/Particle)

Thermal correction to Energy= 1.116688

Thermal correction to Enthalpy= 1.117632

Thermal correction to Gibbs Free Energy= 0.958814

E(solv) = -5173.60884742 A.U.

C -1.140345 -1.074774 0.745331

N -0.004532 -0.733442 0.083305

C 1.012674 -0.192511 0.676138

C 2.078246 0.430211 -0.096145

C 1.963120 0.495781 -1.537801

C 2.823166 1.316335 -2.235803

C 3.903627 1.998677 -1.619745

C 4.078714 1.840603 -0.211528

C 3.139928 1.066576 0.532056

C 3.273284 0.963548 2.018736

C 2.875754 2.072099 2.835732

C 2.929283 1.964086 4.253282

C 3.372825 0.747853 4.832478

C 3.738272 -0.303242 4.038500

C 3.697614 -0.210946 2.618895

C 4.819332 2.814311 -2.331800

C 5.869835 3.426744 -1.693877

C 6.060898 3.253970 -0.306083

C 5.183662 2.480362 0.412615

C 2.396360 3.283762 2.267122

C 2.000655 4.327885 3.064156

C 2.067368 4.221413 4.473856

C 2.522491 3.064858 5.051676

O 1.032944 -0.192372 -2.191050

Br 2.476349 1.545648 -4.082817

H -1.096568 -1.222287 1.826211

H 1.042760 -0.097680 1.766463

H 3.413998 0.664966 5.915773

H 4.082599 -1.233548 4.482028

H 4.675773 2.939789 -3.399051

H 6.558944 4.043585 -2.263413

H 6.896949 3.734788 0.192761

H 5.323320 2.348665 1.481938

H 2.340755 3.362355 1.186771

H 1.630492 5.243286 2.611409

H 1.752284 5.055681 5.093404

H 2.571336 2.966582 6.133511

H 0.398663 -0.572075 -1.501501

C 4.076779 -1.412565 1.829258

C 3.482333 -2.643540 2.103885

C 5.034378 -1.355167 0.810228

C 3.821219 -3.783887 1.377255

H 2.722173 -2.707235 2.878878

C 5.372088 -2.494508 0.095396

H 5.516803 -0.408927 0.581103

C 4.771695 -3.734213 0.357356

H 3.318041 -4.715104 1.613580

H 6.119131 -2.411718 -0.690025

C 5.168067 -4.957283 -0.472865

C 6.671189 -5.228384 -0.295186

H 6.973477 -6.096284 -0.893026

H 7.273706 -4.372246 -0.613627

H 6.905696 -5.434128 0.754674

C 4.399977 -6.214319 -0.052690

H 4.594179 -6.473340 0.993831

H 3.320194 -6.087386 -0.182956

H 4.715247 -7.061024 -0.671754

C 4.870515 -4.682864 -1.956496

H 3.805604 -4.479658 -2.106576

H 5.430409 -3.818541 -2.326199

H 5.147509 -5.551635 -2.565283

C -2.105790 -1.948452 0.082837

O -2.009927 -1.890271 -1.264468

O -2.943165 -2.593483 0.684792

C -2.960220 -2.598007 -2.098220

C -4.348965 -1.991956 -1.925357

H -4.333960 -0.933246 -2.203296

H -5.063412 -2.510414 -2.575091

H -4.691688 -2.064630 -0.889558

C -2.941286 -4.097907 -1.802212

H -3.395397 -4.315079 -0.835869

H -3.493508 -4.624858 -2.587921

H -1.908926 -4.463584 -1.802927

C -2.430302 -2.338805 -3.505633

H -3.093985 -2.793942 -4.247665

H -2.374029 -1.263005 -3.698030

H -1.426439 -2.759373 -3.619537

C -0.778439 2.209631 -2.325459

C -1.543626 1.437251 -1.460309

C -1.519251 1.666907 -0.077840

C -0.698687 2.690015 0.412337

C 0.046652 3.483358 -0.457237

C 0.020705 3.237881 -1.827889

H -0.756715 1.972598 -3.384764

H -2.115379 0.591575 -1.835052

H -0.642492 2.863153 1.485030

H 0.666383 4.283448 -0.060457

H 0.647246 3.813779 -2.503615

C -2.273450 0.804520 0.856651

H -1.900125 0.847902 1.880053

C -3.668757 0.571210 0.727089

C -4.472762 1.101317 -0.320945

C -4.327193 -0.224345 1.707687

C -5.822778 0.884583 -0.406120

H -3.988568 1.744958 -1.046667

C -5.658633 -0.527619 1.663830

H -3.700792 -0.642249 2.491141

C -6.492345 0.040454 0.595301

O -7.724608 -0.177263 0.545231

C -6.671886 1.511341 -1.515729

C -7.737196 2.435287 -0.898026

H -8.356261 2.880534 -1.688304

H -8.375654 1.870662 -0.217145

H -7.256655 3.249292 -0.342602

C -5.827431 2.348904 -2.482787

H -5.331356 3.183082 -1.974230

H -5.056578 1.744737 -2.976546

H -6.476542 2.766844 -3.261149

C -7.367004 0.410281 -2.337886

H -7.992853 -0.206698 -1.692615

H -7.989880 0.863843 -3.120512

H -6.622658 -0.229157 -2.826959

C -6.299958 -1.484930 2.669767

C -7.421025 -0.777260 3.450084

H -8.181973 -0.402105 2.764857

H -7.884360 -1.475539 4.159869

H -7.011666 0.065231 4.020484

C -5.276888 -2.018650 3.679065

H -4.460359 -2.546715 3.174403

H -4.847832 -1.212227 4.285864

H -5.772647 -2.720367 4.360327

C -6.874560 -2.696167 1.912397

H -7.340656 -3.396513 2.618403

H -7.621666 -2.371257 1.186743

H -6.068144 -3.222307 1.387858

**C-4f-SS-TS2**

Zero-point correction= 1.057837 (Hartree/Particle)

Thermal correction to Energy= 1.117695

Thermal correction to Enthalpy= 1.118639

Thermal correction to Gibbs Free Energy= 0.962845

E(solv) = -5173.61208306 A.U.

C 1.496323 2.186296 0.830872

N 0.686114 1.404177 0.061738

C -0.369871 0.841362 0.541002

C -1.046334 -0.237606 -0.172764

C -0.480785 -0.746764 -1.400086

C -1.050216 -1.857832 -1.992218

C -2.208424 -2.484391 -1.468526

C -2.786891 -1.955201 -0.275374

C -2.177698 -0.836349 0.363900

C -2.765821 -0.316804 1.638015

C -2.558419 -1.053251 2.850913

C -3.111275 -0.577514 4.072962

C -3.834338 0.642158 4.070022

C -3.998419 1.345882 2.909728

C -3.475283 0.873436 1.673023

C -2.808697 -3.623468 -2.067254

C -3.935877 -4.194349 -1.532731

C -4.528269 -3.655190 -0.369000

C -3.963488 -2.564391 0.241140

C -1.796553 -2.255003 2.876889

C -1.614115 -2.949914 4.046291

C -2.190807 -2.487989 5.253753

C -2.919463 -1.326096 5.263739

O 0.573265 -0.178238 -1.968082

Br -0.179475 -2.550344 -3.511748

H 1.126121 2.586122 1.775243

H -0.754375 1.120173 1.528147

H -4.253670 1.007583 5.004254

H -4.558265 2.277054 2.907674

H -2.350053 -4.033417 -2.959713

H -4.375726 -5.065748 -2.009250

H -5.425285 -4.105978 0.044917

H -4.413908 -2.152024 1.139242

H -1.355715 -2.618018 1.952733

H -1.019553 -3.858560 4.041600

H -2.043568 -3.049615 6.171541

H -3.355553 -0.951787 6.187008

H 0.922681 0.538031 -1.358561

C -3.682827 1.711663 0.462252

C -3.362790 3.068642 0.494480

C -4.206099 1.186175 -0.725158

C -3.543815 3.878179 -0.625321

H -2.938035 3.493018 1.401183

C -4.387090 1.997610 -1.834737

H -4.470802 0.133851 -0.774932

C -4.056458 3.360053 -1.814918

H -3.261449 4.923351 -0.558343

H -4.788730 1.553919 -2.742134

C -4.248767 4.203024 -3.077250

C -5.727232 4.166389 -3.498502

H -5.876796 4.761124 -4.407489

H -6.060979 3.145235 -3.706486

H -6.366132 4.576126 -2.708735

C -3.846881 5.665200 -2.862441

H -4.445819 6.136734 -2.075432

H -2.789314 5.754346 -2.593307

H -4.005703 6.227644 -3.788822

C -3.381202 3.624374 -4.207423

H -2.323021 3.637099 -3.928508

H -3.653288 2.588989 -4.432820

H -3.507229 4.215338 -5.122500

C 2.450359 3.015789 0.100878

O 3.107796 3.834635 0.972048

O 2.709068 2.947175 -1.084899

C 4.223998 4.628945 0.518334

C 3.766217 5.657844 -0.514646

H 2.933089 6.242553 -0.110763

H 4.590640 6.342640 -0.742021

H 3.443882 5.165547 -1.432336

C 5.339318 3.735195 -0.024733

H 5.038421 3.248216 -0.953429

H 6.234899 4.338478 -0.210528

H 5.592356 2.965692 0.713278

C 4.686054 5.330077 1.792785

H 5.541719 5.978864 1.580986

H 3.875029 5.937103 2.206283

H 4.981235 4.590945 2.543987

C 0.602162 0.093339 4.631692

C 1.110535 0.034311 3.337514

C 2.294113 0.709666 2.993405

C 2.930965 1.461130 3.992111

C 2.429846 1.513499 5.287368

C 1.261949 0.826830 5.614349

H -0.315097 -0.440593 4.865647

H 0.570193 -0.529094 2.585275

H 3.829987 2.015833 3.733939

H 2.949353 2.096373 6.043245

H 0.866250 0.868889 6.625199

C 2.838587 0.747870 1.616531

H 3.676856 1.439903 1.525288

C 2.891579 -0.376416 0.743682

C 2.343361 -1.658255 1.017638

C 3.524527 -0.208067 -0.522727

C 2.352027 -2.685781 0.108836

H 1.976950 -1.848393 2.018246

C 3.531952 -1.165713 -1.498232

H 3.985917 0.758187 -0.710423

C 2.874078 -2.458469 -1.245726

O 2.807026 -3.343234 -2.127571

C 1.844046 -4.084562 0.471532

C 2.928203 -5.139016 0.180226

H 2.566940 -6.135924 0.465973

H 3.185037 -5.138088 -0.879019

H 3.831643 -4.923881 0.763566

C 1.491242 -4.201049 1.959588

H 2.362433 -4.013352 2.598286

H 0.704342 -3.495392 2.249946

H 1.126580 -5.214419 2.167861

C 0.577134 -4.403114 -0.338543

H 0.753693 -4.261429 -1.406902

H 0.257839 -5.438956 -0.159426

H -0.240027 -3.737557 -0.031994

C 4.200341 -0.929643 -2.854177

C 5.319061 -1.965429 -3.065675

H 4.910572 -2.976223 -3.015023

H 5.789307 -1.817355 -4.047063

H 6.093028 -1.854842 -2.296483

C 4.823400 0.469480 -2.942957

H 4.070625 1.255276 -2.811841

H 5.605552 0.612826 -2.186897

H 5.285363 0.597432 -3.929171

C 3.168846 -1.055160 -3.990051

H 3.652754 -0.831643 -4.951061

H 2.759449 -2.064781 -4.019116

H 2.342807 -0.353105 -3.838649

**C-4f-SS-TS3**

Zero-point correction= 1.056679 (Hartree/Particle)

Thermal correction to Energy= 1.115923

Thermal correction to Enthalpy= 1.116867

Thermal correction to Gibbs Free Energy= 0.962545

E(solv) = -5173.61047162 A.U.

C -1.046241 0.863927 0.909175

N 0.167198 0.282719 0.943795

C 1.013030 0.340938 -0.041172

C 2.174322 -0.534972 -0.074828

C 2.345574 -1.535515 0.961575

C 3.334648 -2.485585 0.810531

C 4.258366 -2.476104 -0.267521

C 4.120697 -1.453712 -1.253317

C 3.058627 -0.508147 -1.143795

C 2.843260 0.473533 -2.250709

C 2.197176 0.016997 -3.447010

C 1.932943 0.932608 -4.503041

C 2.312275 2.289897 -4.344612

C 2.911421 2.710805 -3.190585

C 3.187516 1.808966 -2.123797

C 5.302375 -3.424359 -0.413023

C 6.177945 -3.361280 -1.469908

C 6.052980 -2.344181 -2.439817

C 5.045430 -1.417558 -2.330946

C 1.780178 -1.334050 -3.605214

C 1.144759 -1.746067 -4.749404

C 0.896102 -0.834452 -5.802357

C 1.283788 0.474290 -5.678671

O 1.551820 -1.578854 2.022311

Br 3.393236 -3.870853 2.102725

H -1.282794 1.632347 0.172725

H 0.833278 0.997475 -0.898798

H 2.113920 2.989578 -5.152837

H 3.203132 3.750780 -3.072422

H 5.399027 -4.202536 0.335426

H 6.970995 -4.098377 -1.556917

H 6.748810 -2.295781 -3.272048

H 4.942886 -0.635738 -3.078207

H 1.972730 -2.039281 -2.802545

H 0.827766 -2.779852 -4.847359

H 0.391787 -1.173538 -6.702267

H 1.091679 1.188438 -6.475838

H 0.823179 -0.889818 1.899981

C 3.794963 2.361174 -0.884010

C 3.242148 3.495264 -0.289025

C 4.926007 1.791310 -0.288383

C 3.791648 4.042655 0.868389

H 2.351377 3.942879 -0.723158

C 5.470146 2.342731 0.862170

H 5.383837 0.913258 -0.734729

C 4.916327 3.477707 1.470507

H 3.315913 4.915572 1.302293

H 6.346377 1.870159 1.298916

C 5.540025 4.028726 2.754291

C 7.016429 4.377183 2.502647

H 7.472947 4.771692 3.418097

H 7.590867 3.498884 2.193126

H 7.108139 5.134744 1.717073

C 4.826684 5.291750 3.246785

H 4.871570 6.094494 2.502507

H 3.775240 5.094206 3.479411

H 5.309234 5.653253 4.161262

C 5.448488 2.960236 3.856561

H 4.404634 2.696785 4.053742

H 5.976180 2.044968 3.572113

H 5.893611 3.334775 4.786058

C -1.836261 0.825110 2.129775

O -2.996003 1.500938 1.947569

O -1.540577 0.230977 3.153013

C -3.983020 1.573527 2.999670

C -3.426493 2.391073 4.164022

H -3.099818 3.373371 3.807113

H -4.205374 2.539109 4.920066

H -2.578871 1.877155 4.621063

C -4.431626 0.183750 3.451896

H -3.636618 -0.336854 3.986193

H -5.304392 0.283550 4.107825

H -4.729036 -0.400866 2.575176

C -5.144508 2.297945 2.327467

H -5.970700 2.430078 3.033709

H -4.821259 3.281500 1.972028

H -5.498995 1.719471 1.467706

C -1.312423 -3.494836 2.075109

C -1.936745 -2.391927 1.504550

C -1.542913 -1.925724 0.241761

C -0.502651 -2.594566 -0.420242

C 0.119337 -3.700071 0.150608

C -0.281246 -4.150132 1.404984

H -1.610249 -3.821433 3.066848

H -2.690045 -1.848699 2.066011

H -0.171492 -2.222440 -1.387500

H 0.947107 -4.184096 -0.361441

H 0.236032 -4.982085 1.872975

C -2.100089 -0.706873 -0.373457

H -1.434628 -0.274639 -1.124185

C -3.459222 -0.381838 -0.503724

C -4.530468 -1.140221 0.054199

C -3.810720 0.786236 -1.247819

C -5.844677 -0.786990 -0.084241

H -4.274115 -2.054835 0.576960

C -5.093754 1.210401 -1.428544

H -2.985728 1.354127 -1.672402

C -6.197418 0.427996 -0.840736

O -7.386555 0.778879 -0.986696

C -6.976779 -1.624762 0.516379

C -7.913486 -2.124871 -0.598106

H -8.722185 -2.727736 -0.164627

H -8.346560 -1.282859 -1.139163

H -7.360067 -2.754757 -1.304246

C -6.445702 -2.852289 1.265834

H -5.889772 -3.525458 0.603582

H -5.788777 -2.570944 2.097527

H -7.290098 -3.413219 1.682913

C -7.780389 -0.777062 1.520291

H -8.191543 0.106975 1.032162

H -8.603551 -1.372386 1.936579

H -7.140513 -0.457694 2.351535

C -5.433838 2.476562 -2.216682

C -6.330718 2.126171 -3.417786

H -7.256006 1.658573 -3.079529

H -6.573164 3.035676 -3.982495

H -5.809806 1.436060 -4.091811

C -4.177234 3.168194 -2.757026

H -3.503372 3.471955 -1.947853

H -3.620000 2.521383 -3.444356

H -4.468290 4.070188 -3.307401

C -6.160973 3.479310 -1.302793

H -6.420260 4.383178 -1.869458

H -7.073139 3.038400 -0.898727

H -5.510300 3.773248 -0.471138

**C-4f-SS-TS4**

Zero-point correction= 1.057575 (Hartree/Particle)

Thermal correction to Energy= 1.116076

Thermal correction to Enthalpy= 1.117020

Thermal correction to Gibbs Free Energy= 0.965671

E(solv) = -5173.61161243 A.U.

C -0.936014 0.647053 -1.167637

N 0.146748 0.611795 -0.343085

C 1.270344 0.075436 -0.701248

C 2.383271 -0.023371 0.230028

C 2.157484 0.277666 1.627015

C 3.183703 0.083778 2.526298

C 4.494141 -0.279797 2.123952

C 4.737676 -0.481213 0.730995

C 3.654447 -0.387340 -0.199045

C 3.936115 -0.626716 -1.652729

C 4.314585 -1.941990 -2.087280

C 4.634524 -2.174004 -3.454950

C 4.551801 -1.098242 -4.374322

C 4.147507 0.137997 -3.954645

C 3.835365 0.394866 -2.591545

C 5.572161 -0.420016 3.035880

C 6.839410 -0.711556 2.597936

C 7.096176 -0.867928 1.219237

C 6.070719 -0.753411 0.314973

C 4.363079 -3.047215 -1.191702

C 4.725317 -4.296379 -1.628195

C 5.062517 -4.516140 -2.984857

C 5.014542 -3.475852 -3.874971

O 0.975956 0.705942 2.063898

Br 2.784077 0.358852 4.356788

H -0.766742 0.593961 -2.244495

H 1.408085 -0.330335 -1.706969

H 4.801441 -1.277173 -5.417295

H 4.066716 0.960026 -4.660686

H 5.374926 -0.279675 4.092374

H 7.648092 -0.810946 3.316156

H 8.103699 -1.077064 0.872519

H 6.274088 -0.870101 -0.744198

H 4.094577 -2.888227 -0.152482

H 4.750734 -5.125170 -0.926745

H 5.350876 -5.509112 -3.316580

H 5.259289 -3.630179 -4.923187

H 0.376710 0.809002 1.254863

C 3.254031 1.713878 -2.229282

C 2.101631 2.131033 -2.897052

C 3.691431 2.463027 -1.133031

C 1.332687 3.187103 -2.414953

H 1.747173 1.558242 -3.751830

C 2.925419 3.516656 -0.659738

H 4.597819 2.171209 -0.608982

C 1.702857 3.864301 -1.252872

H 0.403413 3.426989 -2.920482

H 3.261289 4.046091 0.228079

C 0.804398 4.892196 -0.567318

C 1.540230 6.235146 -0.438428

H 0.902395 6.966759 0.071251

H 2.465161 6.137656 0.138836

H 1.797868 6.632971 -1.426203

C -0.498767 5.121251 -1.338312

H -0.306746 5.519149 -2.341530

H -1.081679 4.198288 -1.436311

H -1.118754 5.848942 -0.802858

C 0.456394 4.354020 0.832762

H -0.076886 3.398596 0.757627

H 1.353658 4.186673 1.436889

H -0.180338 5.071367 1.365412

C -2.062841 1.498932 -0.791037

O -2.155325 1.652812 0.545927

O -2.857544 1.955570 -1.595410

C -3.257352 2.387960 1.144770

C -4.586855 1.716506 0.827384

H -4.559105 0.662419 1.116158

H -5.387263 2.211002 1.389888

H -4.810572 1.774643 -0.238139

C -3.248934 3.845839 0.690421

H -3.448774 3.918710 -0.379906

H -4.027307 4.392236 1.234270

H -2.284610 4.312264 0.910103

C -2.947182 2.288673 2.634452

H -3.705735 2.824041 3.214094

H -2.943481 1.239146 2.945476

H -1.963252 2.718358 2.849612

C 0.410466 -2.381060 1.732528

C -0.650497 -1.784246 1.063228

C -0.881576 -2.041412 -0.297883

C -0.000056 -2.896411 -0.966380

C 1.053943 -3.512978 -0.294913

C 1.262649 -3.255804 1.056984

H 0.607267 -2.115983 2.768094

H -1.266632 -1.045916 1.570520

H -0.142407 -3.076925 -2.029525

H 1.724606 -4.172827 -0.837772

H 2.100067 -3.712012 1.578440

C -1.929517 -1.340420 -1.061182

H -1.778561 -1.381645 -2.138553

C -3.296370 -1.298355 -0.674396

C -3.791674 -1.823736 0.552525

C -4.257708 -0.798052 -1.600396

C -5.126925 -1.881581 0.852353

H -3.074091 -2.241013 1.248013

C -5.605291 -0.802342 -1.365294

H -3.866833 -0.376069 -2.522080

C -6.116547 -1.384851 -0.117295

O -7.345436 -1.468198 0.108286

C -5.629101 -2.460775 2.177344

C -6.521163 -3.687530 1.917168

H -6.887704 -4.095888 2.868235

H -7.372159 -3.411903 1.293658

H -5.947428 -4.471504 1.409115

C -4.476137 -2.903338 3.085726

H -3.879000 -3.700544 2.628703

H -3.806384 -2.068973 3.326107

H -4.883925 -3.288275 4.027477

C -6.434027 -1.389090 2.936100

H -7.277334 -1.050147 2.333041

H -6.809320 -1.800283 3.882511

H -5.795307 -0.528354 3.169855

C -6.605476 -0.224930 -2.370014

C -7.532195 -1.340079 -2.885665

H -8.069994 -1.798653 -2.054423

H -8.256801 -0.928962 -3.601158

H -6.947481 -2.112554 -3.398939

C -5.898736 0.408307 -3.574316

H -5.206206 1.199166 -3.263419

H -5.334527 -0.335145 -4.149641

H -6.646916 0.849049 -4.243741

C -7.454191 0.872030 -1.700317

H -8.194461 1.258220 -2.413570

H -7.971769 0.472833 -0.826893

H -6.819935 1.709203 -1.387527

**C-4f-RR-TS1**

Zero-point correction= 1.057552 (Hartree/Particle)

Thermal correction to Energy= 1.117252

Thermal correction to Enthalpy= 1.118196

Thermal correction to Gibbs Free Energy= 0.964134

E(solv) = -5173.61322975 A.U.

C -0.561753 0.777137 2.176403

N 0.696022 0.945451 1.718588

C 1.475465 -0.028438 1.354302

C 2.648285 0.245565 0.533930

C 2.914174 1.610749 0.113319

C 3.886138 1.840262 -0.836818

C 4.695774 0.807214 -1.377673

C 4.483808 -0.522164 -0.903145

C 3.452356 -0.779397 0.053336

C 3.318210 -2.173114 0.587308

C 4.283513 -2.591454 1.567307

C 4.238305 -3.912406 2.093485

C 3.225394 -4.794715 1.640210

C 2.302683 -4.373809 0.726764

C 2.327370 -3.055107 0.182965

C 5.698053 1.032873 -2.355006

C 6.458502 -0.001043 -2.843208

C 6.253443 -1.317227 -2.378549

C 5.290819 -1.566131 -1.431828

C 5.296220 -1.714613 2.048725

C 6.207787 -2.133328 2.984410

C 6.164666 -3.452090 3.494050

C 5.198075 -4.319054 3.056228

O 2.237262 2.635930 0.615997

H -0.907674 -0.189705 2.542419

H 1.221232 -1.070246 1.570082

H 3.195583 -5.808725 2.031082

H 1.534270 -5.056236 0.376135

H 5.851524 2.044698 -2.712304

H 7.219379 0.197148 -3.592485

H 6.853707 -2.132990 -2.769823

H 5.127347 -2.579667 -1.078909

H 5.335989 -0.699553 1.668891

H 6.968904 -1.444948 3.338903

H 6.893511 -3.770689 4.233344

H 5.145223 -5.334237 3.442153

H 1.461866 2.257251 1.148186

C 1.232908 -2.695845 -0.758955

C -0.077353 -3.076917 -0.451107

C 1.446939 -2.017115 -1.964699

C -1.138792 -2.789516 -1.304706

H -0.276378 -3.587410 0.489292

C 0.380489 -1.726157 -2.808486

H 2.453691 -1.724021 -2.248101

C -0.936305 -2.096265 -2.499419

H -2.139546 -3.083232 -1.005638

H 0.583690 -1.186829 -3.730739

C -2.083038 -1.738197 -3.447648

C -1.815290 -2.368442 -4.824867

H -2.631910 -2.124453 -5.514130

H -0.882853 -1.999947 -5.264644

H -1.745280 -3.458825 -4.746436

C -3.433812 -2.247023 -2.931789

H -3.441282 -3.338412 -2.828817

H -3.693382 -1.803090 -1.963634

H -4.220733 -1.974547 -3.643348

C -2.170327 -0.209739 -3.590029

H -2.393466 0.250785 -2.622707

H -1.234306 0.222314 -3.959898

H -2.970770 0.054599 -4.291774

C -1.276590 1.932871 2.702697

O -0.611825 3.092153 2.459545

O -2.358688 1.871431 3.258805

C -1.049750 4.339466 3.034613

C 0.019202 5.318533 2.557856

H 0.063378 5.311264 1.464305

H -0.209066 6.331955 2.904115

H 1.001509 5.022977 2.937735

C -1.049617 4.237090 4.559896

H -0.075903 3.875128 4.905228

H -1.226193 5.226288 4.995844

H -1.826523 3.551424 4.901694

C -2.421727 4.759553 2.507480

H -2.701808 5.718765 2.957217

H -2.382869 4.893409 1.422631

H -3.177372 4.013101 2.754754

C 0.560891 2.673237 -2.028524

C 0.081416 1.541663 -1.376905

C -1.052008 1.606151 -0.555562

C -1.687157 2.845207 -0.391441

C -1.214029 3.974649 -1.049395

C -0.085987 3.894288 -1.865513

H 1.470201 2.611954 -2.619993

H 0.596411 0.590313 -1.487487

H -2.530712 2.919266 0.289384

H -1.714954 4.929078 -0.907626

H 0.297742 4.783479 -2.357079

C -1.489490 0.390889 0.155791

H -0.726914 -0.391060 0.179575

C -2.821253 -0.053945 0.247089

C -3.941339 0.625526 -0.320495

C -3.076466 -1.321787 0.854207

C -5.207761 0.113250 -0.314450

H -3.750086 1.558529 -0.835889

C -4.311996 -1.897154 0.914548

H -2.213846 -1.835834 1.273186

C -5.465784 -1.185066 0.331402

O -6.611469 -1.677589 0.365249

C -6.372380 0.834043 -0.997590

C -7.473502 1.150793 0.030801

H -8.307755 1.667614 -0.461167

H -7.842009 0.233419 0.490553

H -7.080844 1.807139 0.815673

C -5.931968 2.158005 -1.633503

H -5.526059 2.851636 -0.888174

H -5.171888 2.003878 -2.408262

H -6.797661 2.638520 -2.103394

C -6.946747 -0.052213 -2.117660

H -7.292533 -1.004452 -1.713794

H -7.787642 0.456233 -2.606944

H -6.177947 -0.245388 -2.875562

C -4.545091 -3.270225 1.547745

C -5.537302 -3.151067 2.718732

H -6.489707 -2.749593 2.371573

H -5.705377 -4.137948 3.169047

H -5.133105 -2.487303 3.491167

C -3.247765 -3.875982 2.095430

H -2.507126 -4.033258 1.301417

H -2.796389 -3.242532 2.867014

H -3.464504 -4.851844 2.544365

C -5.104004 -4.240220 0.490504

H -5.274886 -5.227737 0.938247

H -6.043599 -3.863357 0.085246

H -4.387391 -4.360827 -0.331991

Br 4.096677 3.628883 -1.427771

**C-4f-RR-TS2**

Zero-point correction= 1.058062 (Hartree/Particle)

Thermal correction to Energy= 1.117515

Thermal correction to Enthalpy= 1.118460

Thermal correction to Gibbs Free Energy= 0.964914

E(solv) = -5173.61176590 A.U.

C -0.476518 -0.755756 -2.527470

N 0.777644 -0.820800 -2.025926

C 1.409360 0.209483 -1.546180

C 2.602990 0.027871 -0.728340

C 3.092770 -1.314449 -0.473340

C 4.140442 -1.486956 0.406771

C 4.824776 -0.401387 1.012309

C 4.353562 0.917961 0.741041

C 3.210349 1.103025 -0.093859

C 2.655162 2.484334 -0.261044

C 3.269052 3.360212 -1.216153

C 2.754153 4.672211 -1.411543

C 1.631723 5.086278 -0.651079

C 1.057456 4.237345 0.251617

C 1.556204 2.919058 0.468363

C 5.936402 -0.564762 1.877572

C 6.556545 0.519653 2.447265

C 6.091050 1.826115 2.186861

C 5.014103 2.015334 1.357028

C 4.387591 2.953909 -1.996200

C 4.959706 3.806115 -2.906435

C 4.447357 5.111890 -3.091621

C 3.367730 5.531256 -2.358903

O 2.535292 -2.377185 -1.043599

H -0.890167 0.186150 -2.888278

H 1.028420 1.226336 -1.686754

H 1.236164 6.088510 -0.795592

H 0.204669 4.564710 0.839446

H 6.289114 -1.569531 2.080958

H 7.407138 0.368622 3.105754

H 6.581287 2.680092 2.644375

H 4.648213 3.018681 1.161260

H 4.783962 1.952679 -1.860822

H 5.812501 3.476320 -3.492224

H 4.908581 5.776142 -3.816520

H 2.957740 6.529304 -2.493376

H 1.707168 -2.062106 -1.536163

C 0.810535 2.067813 1.437878

C -0.589761 2.064553 1.399310

C 1.432024 1.283459 2.414066

C -1.337739 1.273770 2.261037

H -1.111807 2.651764 0.647459

C 0.676799 0.502113 3.286788

H 2.513697 1.285359 2.503870

C -0.719839 0.462147 3.220215

H -2.420806 1.275959 2.148385

H 1.205903 -0.098880 4.018958

C -1.577792 -0.403083 4.146772

C -2.430231 0.519626 5.034539

H -3.058768 -0.076963 5.706418

H -1.794613 1.172473 5.642960

H -3.088094 1.153067 4.430649

C -2.510228 -1.303165 3.317637

H -3.132995 -0.718851 2.631981

H -1.938470 -2.023271 2.722233

H -3.177260 -1.860411 3.987549

C -0.724147 -1.303473 5.044576

H -0.097014 -1.972767 4.444819

H -0.078002 -0.720985 5.710984

H -1.376940 -1.922528 5.669381

C -1.045482 -1.955301 -3.134831

O -0.344260 -3.066480 -2.801280

O -2.052118 -1.962692 -3.820753

C -0.627628 -4.339893 -3.417963

C 0.406970 -5.258179 -2.774806

H 0.271338 -5.262316 -1.688605

H 0.300622 -6.279022 -3.156683

H 1.418021 -4.898750 -2.988219

C -0.403287 -4.238226 -4.926166

H 0.600383 -3.848898 -5.125197

H -0.488044 -5.230495 -5.382183

H -1.140511 -3.572838 -5.379055

C -2.039892 -4.829350 -3.097968

H -2.182561 -5.822696 -3.538648

H -2.169700 -4.914118 -2.015484

H -2.788723 -4.145787 -3.498736

C -1.461252 -4.155583 0.557863

C -1.863442 -2.977937 -0.063254

C -1.109689 -1.805056 0.082255

C 0.081090 -1.865472 0.819430

C 0.505534 -3.051955 1.408110

C -0.275848 -4.198843 1.293628

H -2.063542 -5.053969 0.447448

H -2.747874 -2.969217 -0.694744

H 0.678304 -0.963713 0.928007

H 1.464512 -3.082304 1.919692

H 0.045568 -5.128480 1.754561

C -1.470067 -0.521647 -0.542186

H -0.662326 0.207259 -0.487571

C -2.755202 0.054615 -0.547797

C -2.863453 1.413192 -0.974881

C -3.916545 -0.529833 0.036314

C -3.984152 2.173849 -0.816677

H -1.975048 1.846273 -1.431015

C -5.076274 0.165228 0.243867

H -3.840232 -1.546451 0.400689

C -5.166661 1.577293 -0.166333

O -6.194755 2.251844 0.047004

C -4.041687 3.639369 -1.252038

C -4.255661 4.536357 -0.019679

H -4.290193 5.590932 -0.322620

H -5.188289 4.278742 0.483272

H -3.426581 4.413135 0.688554

C -2.745485 4.088223 -1.937678

H -1.877155 4.028420 -1.269414

H -2.529954 3.490309 -2.830298

H -2.847181 5.133767 -2.250514

C -5.192776 3.849934 -2.252195

H -6.144949 3.564113 -1.804419

H -5.238387 4.904649 -2.552846

H -5.027903 3.246612 -3.152265

C -6.279067 -0.468940 0.947200

C -6.564648 0.277224 2.263195

H -6.771581 1.330084 2.067391

H -7.429423 -0.170466 2.770037

H -5.701126 0.202702 2.936387

C -6.032059 -1.943094 1.290559

H -5.825289 -2.538391 0.393615

H -5.193502 -2.068154 1.985877

H -6.927001 -2.356145 1.769916

C -7.518803 -0.404258 0.036422

H -8.379494 -0.859485 0.543516

H -7.756516 0.630191 -0.212985

H -7.336791 -0.959706 -0.890780

Br 4.628514 -3.271979 0.816083

**C-4f-RR-TS3**

Zero-point correction= 1.058543 (Hartree/Particle)

Thermal correction to Energy= 1.117949

Thermal correction to Enthalpy= 1.118893

Thermal correction to Gibbs Free Energy= 0.965802

E(solv) = -5173.61235613 A.U.

C 0.586409 0.801971 -2.209375

N -0.662747 0.981433 -1.733901

C -1.453506 0.009864 -1.387086

C -2.628931 0.278844 -0.568937

C -2.870546 1.632588 -0.099635

C -3.853293 1.845696 0.843790

C -4.710825 0.815661 1.311529

C -4.525686 -0.496521 0.781802

C -3.466004 -0.744084 -0.143734

C -3.303717 -2.129040 -0.691343

C -4.189115 -2.540931 -1.743727

C -4.071317 -3.843352 -2.303635

C -3.067508 -4.713461 -1.808788

C -2.225965 -4.301718 -0.815031

C -2.328817 -3.003367 -0.235678

C -5.736160 1.027448 2.267685

C -6.547926 -0.001582 2.678022

C -6.373297 -1.299270 2.152971

C -5.385396 -1.535721 1.229101

C -5.192064 -1.675332 -2.262414

C -6.028164 -2.087039 -3.269270

C -5.911048 -3.386092 -3.817033

C -4.951198 -4.241748 -3.342612

O -2.159962 2.660286 -0.548047

Br -4.015644 3.605936 1.526397

H 0.915338 -0.165597 -2.589171

H -1.212903 -1.029639 -1.629814

H -2.978525 -5.710984 -2.231922

H -1.463770 -4.974250 -0.432170

H -5.866356 2.025092 2.671246

H -7.326168 0.185674 3.412241

H -7.016330 -2.110855 2.479968

H -5.244977 -2.535235 0.828870

H -5.285772 -0.676382 -1.849844

H -6.784736 -1.408522 -3.651610

H -6.578386 -3.699267 -4.614560

H -4.843471 -5.241119 -3.757461

H -1.398953 2.286152 -1.102468

C -1.326289 -2.642213 0.802499

C 0.025891 -2.931210 0.574629

C -1.669998 -2.053297 2.020669

C 0.998036 -2.623665 1.516889

H 0.321427 -3.377474 -0.373513

C -0.689753 -1.747332 2.963359

H -2.711377 -1.836524 2.240578

C 0.663843 -2.010431 2.730887

H 2.038539 -2.837936 1.282908

H -1.001754 -1.276670 3.889917

C 1.764888 -1.672207 3.738180

C 2.367933 -2.982550 4.271691

H 3.163078 -2.766629 4.994909

H 1.603155 -3.590653 4.767776

H 2.802507 -3.576051 3.460575

C 2.867291 -0.845941 3.055095

H 3.345777 -1.381829 2.228091

H 2.460108 0.087718 2.652031

H 3.647338 -0.597475 3.785104

C 1.224334 -0.858725 4.918380

H 0.769239 0.076768 4.573617

H 0.479372 -1.418513 5.494987

H 2.047415 -0.605133 5.594959

C 1.322161 1.950586 -2.718306

O 0.667204 3.118259 -2.483809

O 2.412826 1.878155 -3.256920

C 1.135153 4.360706 -3.044594

C 0.062677 5.351258 -2.601276

H -0.019745 5.341930 -1.509930

H 0.314859 6.362967 -2.936003

H -0.909466 5.068840 -3.016098

C 1.181816 4.261854 -4.569274

H 0.219153 3.900125 -4.944706

H 1.370339 5.252500 -4.997141

H 1.968415 3.577127 -4.889366

C 2.494291 4.764849 -2.473244

H 2.809910 5.711741 -2.925649

H 2.419296 4.916508 -1.392029

H 3.244453 4.000875 -2.682431

C -0.510319 2.518914 2.111231

C -0.015537 1.423568 1.410214

C 1.082043 1.553934 0.548032

C 1.665668 2.818720 0.395942

C 1.173858 3.912434 1.099150

C 0.082430 3.768059 1.954879

H -1.393288 2.408637 2.735508

H -0.497412 0.453044 1.505259

H 2.477750 2.943226 -0.314721

H 1.630794 4.889426 0.961851

H -0.318455 4.629926 2.480395

C 1.528632 0.365992 -0.204058

H 0.770518 -0.418456 -0.238386

C 2.855573 -0.078550 -0.305848

C 3.989456 0.627699 0.197345

C 3.086334 -1.391962 -0.819316

C 5.248735 0.099951 0.213324

H 3.816280 1.598393 0.644816

C 4.310834 -1.993057 -0.836926

H 2.210567 -1.922743 -1.188764

C 5.481012 -1.249331 -0.331052

O 6.620504 -1.756320 -0.344078

C 6.435595 0.858420 0.811500

C 7.508535 1.089338 -0.268258

H 8.357221 1.639722 0.158575

H 7.863201 0.136749 -0.663283

H 7.095573 1.682827 -1.091844

C 6.018674 2.227839 1.359933

H 5.602978 2.870550 0.575256

H 5.272707 2.134501 2.157862

H 6.896841 2.733270 1.777757

C 7.040110 0.053228 1.976160

H 7.383811 -0.922222 1.629883

H 7.888077 0.598829 2.410334

H 6.290735 -0.093249 2.763255

C 4.517084 -3.418977 -1.349706

C 5.488242 -3.416792 -2.544004

H 6.450238 -2.994615 -2.251438

H 5.639924 -4.442136 -2.905710

H 5.075613 -2.822138 -3.366861

C 3.203048 -4.055205 -1.815522

H 2.480520 -4.136161 -0.993857

H 2.738972 -3.486689 -2.629159

H 3.400954 -5.068327 -2.183576

C 5.084802 -4.299706 -0.221564

H 5.222033 -5.328056 -0.580106

H 6.042893 -3.910122 0.123325

H 4.388047 -4.326349 0.626125

**C-4f-RR-TS4**

Zero-point correction= 1.057795 (Hartree/Particle)

Thermal correction to Energy= 1.117610

Thermal correction to Enthalpy= 1.118554

Thermal correction to Gibbs Free Energy= 0.962932

E(solv) = -5173.61039283 A.U.

C -1.072300 0.947830 0.297060

N 0.195107 0.764491 0.742381

C 1.080528 -0.059306 0.252233

C 2.425763 -0.101658 0.826523

C 2.705129 0.612830 2.055726

C 3.976617 0.584211 2.587141

C 5.055038 -0.085174 1.954229

C 4.787104 -0.771854 0.732193

C 3.460036 -0.791042 0.201415

C 3.186029 -1.555603 -1.055215

C 3.206152 -2.989913 -1.026932

C 2.854051 -3.722697 -2.194861

C 2.477673 -3.015989 -3.365142

C 2.483839 -1.649637 -3.379042

C 2.858701 -0.898486 -2.231225

C 6.378906 -0.088494 2.464690

C 7.397175 -0.718373 1.793518

C 7.144119 -1.375960 0.570561

C 5.870840 -1.400188 0.059012

C 3.567490 -3.722002 0.139541

C 3.598690 -5.093567 0.135069

C 3.252772 -5.817469 -1.031547

C 2.882259 -5.142395 -2.165721

O 1.741506 1.281495 2.683193

H -1.639448 1.721552 0.801272

H 0.855570 -0.690427 -0.603705

H 2.193974 -3.578709 -4.250984

H 2.210191 -1.106476 -4.278913

H 6.573182 0.426928 3.398225

H 8.402944 -0.703420 2.203367

H 7.954079 -1.861624 0.034776

H 5.674969 -1.903608 -0.882679

H 3.821425 -3.176994 1.042266

H 3.882636 -5.629215 1.036039

H 3.275633 -6.903189 -1.021872

H 2.606139 -5.683076 -3.067923

H 0.937932 1.264343 2.057328

C 2.855391 0.585237 -2.332433

C 1.722211 1.251703 -2.798228

C 3.975102 1.344399 -1.975027

C 1.702564 2.642238 -2.891340

H 0.826527 0.681567 -3.027668

C 3.949879 2.727532 -2.079191

H 4.869124 0.844371 -1.611494

C 2.812503 3.409709 -2.534627

H 0.790066 3.118955 -3.233602

H 4.836190 3.287029 -1.790238

C 2.826507 4.938644 -2.607730

C 3.946251 5.396173 -3.557442

H 3.971694 6.490910 -3.615285

H 4.927709 5.053607 -3.215607

H 3.783404 5.000838 -4.565701

C 1.499431 5.504609 -3.122968

H 1.273854 5.144516 -4.132450

H 0.666154 5.233639 -2.466670

H 1.556637 6.597891 -3.160248

C 3.082773 5.512448 -1.204055

H 2.300500 5.193383 -0.508395

H 4.043792 5.177616 -0.802369

H 3.092518 6.608505 -1.237895

C -1.548486 0.572903 -1.010775

O -2.804138 1.059013 -1.196957

O -0.958689 -0.108818 -1.845620

C -3.487868 0.909422 -2.457297

C -4.835145 1.574848 -2.192312

H -4.692227 2.626955 -1.927028

H -5.472220 1.516101 -3.080927

H -5.340232 1.074835 -1.359310

C -3.698015 -0.561649 -2.816054

H -4.200685 -1.065721 -1.983842

H -4.345276 -0.631340 -3.698057

H -2.748626 -1.056122 -3.025572

C -2.722086 1.651059 -3.553218

H -3.311157 1.659088 -4.476646

H -2.546055 2.687337 -3.246032

H -1.762406 1.168195 -3.747867

C 0.454193 -3.260941 1.436868

C -0.378416 -2.222799 1.842521

C -1.481040 -1.830036 1.065630

C -1.671171 -2.463872 -0.175582

C -0.829401 -3.488210 -0.585982

C 0.225099 -3.905186 0.224636

H 1.300017 -3.548923 2.054484

H -0.177185 -1.697935 2.773525

H -2.446940 -2.111803 -0.844992

H -0.980861 -3.948179 -1.558082

H 0.885625 -4.701918 -0.104869

C -2.302594 -0.714247 1.533567

H -1.856691 -0.158949 2.356080

C -3.672336 -0.502310 1.333527

C -4.545935 -1.393172 0.638006

C -4.258002 0.688352 1.868317

C -5.875153 -1.139585 0.449351

H -4.134472 -2.338258 0.305194

C -5.566491 1.029688 1.700096

H -3.587671 1.353535 2.407620

C -6.456774 0.116139 0.959045

O -7.660916 0.388965 0.783175

C -6.797377 -2.130351 -0.267115

C -7.399362 -1.472512 -1.523292

H -8.076144 -2.176048 -2.025362

H -7.954612 -0.572976 -1.255283

H -6.606754 -1.202652 -2.231459

C -6.048801 -3.391427 -0.715650

H -5.239288 -3.160344 -1.418389

H -5.620246 -3.934592 0.134014

H -6.748674 -4.063651 -1.224865

C -7.931609 -2.571608 0.676083

H -8.518496 -1.710818 0.998024

H -8.590757 -3.283046 0.162212

H -7.517198 -3.069575 1.560279

C -6.149174 2.336209 2.243075

C -6.680828 3.189927 1.077243

H -7.448999 2.648223 0.523956

H -7.108077 4.125074 1.461742

H -5.863117 3.444450 0.393254

C -5.096327 3.166918 2.985470

H -4.689678 2.629138 3.849396

H -4.263781 3.445788 2.329977

H -5.557849 4.090431 3.352781

C -7.292111 2.036867 3.230249

H -7.706324 2.976208 3.618470

H -8.085328 1.473224 2.738437

H -6.916938 1.454430 4.079729

Br 4.254571 1.515461 4.215066

**C-4f-RS-TS1**

Zero-point correction= 1.057778 (Hartree/Particle)

Thermal correction to Energy= 1.117378

Thermal correction to Enthalpy= 1.118322

Thermal correction to Gibbs Free Energy= 0.965807

E(solv) = -5173.61326670 A.U.

C -0.611509 1.728026 -2.084938

N 0.252024 0.732348 -1.790960

C 1.417505 0.901580 -1.243983

C 2.235037 -0.251938 -0.892955

C 1.746390 -1.585839 -1.179549

C 2.504049 -2.676473 -0.806001

C 3.765756 -2.553398 -0.169780

C 4.255994 -1.238919 0.092695

C 3.469960 -0.105036 -0.272934

C 4.003633 1.261540 0.019224

C 5.035487 1.792031 -0.824893

C 5.563149 3.088947 -0.572139

C 5.043212 3.840562 0.512023

C 4.047226 3.327242 1.293632

C 3.510423 2.027300 1.064115

C 4.550129 -3.667659 0.225231

C 5.759838 -3.497167 0.850656

C 6.246787 -2.199496 1.119153

C 5.510481 -1.102253 0.749113

C 5.549304 1.060507 -1.931870

C 6.535889 1.585470 -2.727158

C 7.066620 2.870415 -2.463368

C 6.587144 3.603274 -1.409345

O 0.566071 -1.784434 -1.762386

H -0.273336 2.764991 -2.107504

H 1.813762 1.902930 -1.050936

H 5.449318 4.829450 0.709943

H 3.656480 3.902224 2.128392

H 4.168710 -4.662470 0.025840

H 6.341582 -4.365933 1.144563

H 7.201122 -2.068936 1.620395

H 5.881225 -0.104332 0.961813

H 5.144345 0.076120 -2.141432

H 6.912061 1.011317 -3.568362

H 7.848348 3.273334 -3.100411

H 6.979077 4.595327 -1.198174

H 0.132119 -0.878368 -1.899730

C 2.398159 1.580657 1.945712

C 1.326075 2.449942 2.181576

C 2.382354 0.339858 2.584685

C 0.277105 2.087834 3.015772

H 1.312707 3.416056 1.680867

C 1.336660 -0.009450 3.437371

H 3.202171 -0.355801 2.431357

C 0.259246 0.850275 3.671354

H -0.548582 2.782082 3.153952

H 1.373972 -0.979567 3.921156

C -0.896949 0.512934 4.616583

C -0.948931 1.569162 5.734921

H -1.758192 1.333634 6.435739

H -0.005816 1.595175 6.291872

H -1.133955 2.570751 5.334415

C -2.226958 0.522324 3.843846

H -2.396310 1.475689 3.332148

H -2.247461 -0.263947 3.082654

H -3.060640 0.355163 4.536423

C -0.723850 -0.862736 5.268192

H -0.687151 -1.661885 4.520590

H 0.188204 -0.911013 5.874139

H -1.576338 -1.063890 5.925790

C -1.731213 1.410582 -2.960727

O -2.069517 0.107844 -2.867952

O -2.326487 2.230579 -3.643473

C -3.123289 -0.455861 -3.680881

C -3.233188 -1.880282 -3.146246

H -3.548215 -1.866158 -2.097606

H -3.967300 -2.447954 -3.727412

H -2.262640 -2.382400 -3.205246

C -2.684571 -0.444870 -5.143587

H -1.737305 -0.982193 -5.253172

H -3.440165 -0.941642 -5.761657

H -2.558417 0.581809 -5.494579

C -4.448049 0.281456 -3.483717

H -5.258055 -0.318318 -3.914774

H -4.640057 0.393653 -2.411440

H -4.434799 1.264103 -3.954983

C -3.763238 4.858597 -1.117885

C -3.293645 3.551078 -1.057530

C -2.221738 3.214269 -0.216421

C -1.621474 4.240692 0.526523

C -2.094592 5.547015 0.470257

C -3.174836 5.860930 -0.350166

H -4.588315 5.096024 -1.782786

H -3.731345 2.795344 -1.699858

H -0.774253 3.998012 1.161402

H -1.615994 6.320190 1.064760

H -3.546007 6.880339 -0.401957

C -1.674136 1.844169 -0.096601

H -0.652140 1.808038 0.284566

C -2.442170 0.694924 0.155222

C -1.775589 -0.526460 0.499905

C -3.870680 0.669527 0.130596

C -2.437178 -1.697962 0.740044

H -0.691426 -0.477575 0.595613

C -4.601491 -0.464860 0.339078

H -4.385818 1.609410 -0.025909

C -3.903574 -1.740658 0.580755

O -4.535183 -2.811097 0.674876

C -1.719658 -2.985899 1.154806

C -1.800373 -4.004770 0.004193

H -1.305318 -4.940751 0.295686

H -2.843496 -4.214242 -0.243399

H -1.283237 -3.612835 -0.878161

C -0.246284 -2.725770 1.473491

H 0.289128 -2.341317 0.605589

H -0.141220 -2.000052 2.289242

H 0.240779 -3.661776 1.771241

C -2.359018 -3.577611 2.425837

H -3.391342 -3.876580 2.248684

H -1.780353 -4.451161 2.751864

H -2.344419 -2.841483 3.240309

C -6.132749 -0.458829 0.350214

C -6.687367 -1.380363 -0.752802

H -6.330753 -2.400788 -0.612950

H -7.785003 -1.373560 -0.724826

H -6.373178 -1.028432 -1.741777

C -6.700776 0.945486 0.112345

H -6.376124 1.652671 0.883938

H -6.404001 1.344922 -0.865022

H -7.795651 0.901162 0.135479

C -6.636836 -0.945733 1.721162

H -7.734285 -0.940617 1.743123

H -6.282471 -1.958550 1.918159

H -6.275669 -0.282756 2.516164

Br 1.754342 -4.383202 -1.124984

**C-4f-RS-TS2**

Zero-point correction= 1.057224 (Hartree/Particle)

Thermal correction to Energy= 1.116919

Thermal correction to Enthalpy= 1.117863

Thermal correction to Gibbs Free Energy= 0.965328

E(solv) = -5173.61148545 A.U.

C 0.494875 0.727769 2.720309

N -0.488217 0.034264 2.114438

C -1.546733 0.498001 1.525467

C -2.375614 -0.423540 0.755144

C -2.074510 -1.844932 0.776239

C -2.830390 -2.704804 0.007309

C -3.872191 -2.258979 -0.845584

C -4.170159 -0.863922 -0.862293

C -3.424257 0.030451 -0.030554

C -3.874730 1.457911 -0.012023

C -5.090680 1.726522 0.710063

C -5.698167 3.009369 0.641340

C -5.084814 4.004936 -0.158378

C -3.906097 3.747896 -0.794948

C -3.249037 2.480608 -0.713958

C -4.617747 -3.130772 -1.679864

C -5.608328 -2.651678 -2.499624

C -5.901423 -1.271693 -2.529117

C -5.199829 -0.404834 -1.729953

C -5.719171 0.734184 1.513901

C -6.876893 1.006932 2.198334

C -7.481777 2.282256 2.113700

C -6.900784 3.260459 1.349514

O -1.091590 -2.339321 1.524851

H 0.351068 1.746763 3.075612

H -1.816223 1.558951 1.548960

H -5.566501 4.974484 -0.259208

H -3.464300 4.516753 -1.419769

H -4.380379 -4.188027 -1.662138

H -6.163460 -3.337695 -3.132901

H -6.678186 -0.893945 -3.187148

H -5.423178 0.656677 -1.762700

H -5.262903 -0.247048 1.589951

H -7.332859 0.235267 2.811232

H -8.401014 2.483079 2.656032

H -7.348749 4.248558 1.274363

H -0.580528 -1.548664 1.909548

C -1.908494 2.384461 -1.363785

C -1.103226 3.534377 -1.381685

C -1.379813 1.235730 -1.966286

C 0.145408 3.543346 -1.984337

H -1.447763 4.438913 -0.888209

C -0.110465 1.244502 -2.545995

H -1.961958 0.321109 -2.001494

C 0.681779 2.397181 -2.578467

H 0.722241 4.464463 -1.967579

H 0.260619 0.319237 -2.976300

C 2.052328 2.468902 -3.255510

C 1.924336 3.343980 -4.514759

H 2.893420 3.416502 -5.022756

H 1.199129 2.915309 -5.214961

H 1.594643 4.358357 -4.263967

C 3.084254 3.099630 -2.305381

H 2.823958 4.127183 -2.031422

H 3.170958 2.512626 -1.386637

H 4.066538 3.123836 -2.792559

C 2.560865 1.084199 -3.662725

H 2.629131 0.417511 -2.796815

H 1.909346 0.617864 -4.410529

H 3.560701 1.170800 -4.101805

C 1.555879 -0.052180 3.346154

O 1.486414 -1.345988 2.962404

O 2.430235 0.406848 4.064025

C 2.509540 -2.305843 3.304722

C 1.967391 -3.590223 2.686350

H 1.786673 -3.428111 1.619579

H 2.685498 -4.407308 2.810241

H 1.016872 -3.869463 3.151060

C 2.636879 -2.446834 4.820544

H 1.650684 -2.631215 5.259414

H 3.281432 -3.302264 5.051024

H 3.060140 -1.545149 5.263923

C 3.829040 -1.917440 2.644947

H 4.583840 -2.684658 2.852017

H 3.696354 -1.844566 1.560883

H 4.187797 -0.957311 3.023415

C 3.761966 3.888554 2.803690

C 3.287314 2.688038 2.287128

C 2.167169 2.667992 1.440742

C 1.512132 3.880272 1.182202

C 1.991594 5.080563 1.695431

C 3.127162 5.091117 2.501566

H 4.627865 3.880057 3.459101

H 3.751552 1.752997 2.578885

H 0.619039 3.867537 0.565697

H 1.472450 6.008230 1.470261

H 3.503973 6.026378 2.905817

C 1.616781 1.420159 0.868152

H 0.590964 1.503732 0.498913

C 2.400296 0.421792 0.253770

C 1.759655 -0.695362 -0.377134

C 3.818030 0.521260 0.104701

C 2.433806 -1.627801 -1.118423

H 0.681649 -0.774785 -0.245810

C 4.559641 -0.357634 -0.630521

H 4.307941 1.379981 0.544108

C 3.893831 -1.487013 -1.301356

O 4.535719 -2.276610 -2.021759

C 1.723705 -2.776456 -1.842680

C 2.322175 -4.134603 -1.430947

H 1.793966 -4.940849 -1.955285

H 3.383421 -4.183651 -1.674428

H 2.195447 -4.299522 -0.355463

C 0.230467 -2.814326 -1.512599

H 0.062713 -2.964000 -0.441675

H -0.281675 -1.890987 -1.815034

H -0.247993 -3.646839 -2.041088

C 1.875726 -2.598156 -3.364555

H 2.928366 -2.545425 -3.648484

H 1.405914 -3.442312 -3.885269

H 1.371346 -1.681395 -3.693510

C 6.067665 -0.179268 -0.815096

C 6.805767 -1.429554 -0.303450

H 6.481559 -2.315402 -0.850548

H 7.889218 -1.304620 -0.429470

H 6.601704 -1.578089 0.763824

C 6.600256 1.027778 -0.033922

H 6.154092 1.967637 -0.380111

H 6.405713 0.931379 1.040923

H 7.684718 1.100603 -0.175465

C 6.389265 0.045463 -2.303274

H 7.471539 0.167474 -2.441968

H 6.045041 -0.799755 -2.900336

H 5.895040 0.957255 -2.660904

Br -2.407349 -4.546462 0.106808

**C-4f-RS-TS3**

Zero-point correction= 1.056725 (Hartree/Particle)

Thermal correction to Energy= 1.116553

Thermal correction to Enthalpy= 1.117498

Thermal correction to Gibbs Free Energy= 0.963930

E(solv) = -5173.60651604 A.U.

C -0.647772 1.096429 -2.337800

N 0.422677 0.343093 -1.998899

C 1.506823 0.732458 -1.407178

C 2.452902 -0.277756 -0.938942

C 2.235579 -1.671855 -1.280073

C 3.086252 -2.630948 -0.769357

C 4.167209 -2.318779 0.093231

C 4.389798 -0.946922 0.415406

C 3.526935 0.057158 -0.128557

C 3.869954 1.476467 0.198753

C 4.997186 2.038094 -0.497752

C 5.509798 3.310140 -0.124868

C 4.897929 3.994904 0.953960

C 3.803463 3.466955 1.572618

C 3.233210 2.212492 1.190348

C 5.023085 -3.303081 0.652108

C 6.048233 -2.954152 1.494259

C 6.268840 -1.599825 1.823911

C 5.459369 -0.625770 1.296785

C 5.631413 1.353952 -1.573236

C 6.702171 1.906950 -2.229677

C 7.209962 3.169895 -1.846683

C 6.623135 3.853526 -0.813994

O 1.242695 -2.052690 -2.077695

Br 2.745021 -4.427725 -1.253330

H -0.566188 2.168186 -2.509994

H 1.720544 1.786878 -1.200263

H 5.315045 4.943169 1.284249

H 3.365842 3.994685 2.412981

H 4.842632 -4.342606 0.404142

H 6.688606 -3.725320 1.912677

H 7.076421 -1.327645 2.496809

H 5.627233 0.413253 1.560827

H 5.249594 0.385413 -1.878448

H 7.165014 1.369237 -3.051660

H 8.060701 3.593858 -2.372115

H 6.998059 4.826375 -0.504913

H 0.630429 -1.257519 -2.212415

C 1.960921 1.829445 1.872965

C 1.093758 2.863551 2.258495

C 1.548924 0.522338 2.167275

C -0.100925 2.614023 2.915547

H 1.342078 3.892290 2.013073

C 0.335617 0.276758 2.812289

H 2.178561 -0.322545 1.909731

C -0.514727 1.312698 3.213440

H -0.729068 3.459153 3.183484

H 0.055270 -0.755958 2.997586

C -1.813215 1.092807 3.992650

C -1.589753 1.568472 5.439003

H -2.505596 1.429724 6.025920

H -0.784184 0.999861 5.916321

H -1.320267 2.630060 5.470651

C -2.957768 1.906219 3.366458

H -2.772709 2.984473 3.408757

H -3.101338 1.621417 2.320580

H -3.890146 1.708539 3.908971

C -2.227680 -0.380541 4.013626

H -2.360563 -0.772137 2.999611

H -1.488106 -1.000802 4.532825

H -3.179601 -0.488383 4.544966

C -1.696795 0.331959 -3.003293

O -2.657069 1.137633 -3.526335

O -1.748378 -0.882887 -3.023709

C -3.831641 0.550033 -4.133037

C -4.615540 1.767558 -4.616143

H -4.032977 2.326738 -5.354828

H -5.558281 1.451262 -5.073901

H -4.838625 2.435861 -3.778544

C -4.644441 -0.234051 -3.102203

H -4.998882 0.425430 -2.302228

H -5.522567 -0.673157 -3.589496

H -4.049713 -1.030323 -2.651418

C -3.442413 -0.322945 -5.327408

H -4.343282 -0.592172 -5.889619

H -2.776090 0.237891 -5.991410

H -2.936234 -1.230982 -5.000621

C -3.804512 4.286835 -1.321950

C -3.351328 2.982087 -1.163608

C -2.135503 2.713039 -0.516405

C -1.370494 3.807803 -0.088179

C -1.822590 5.113379 -0.244641

C -3.049615 5.360298 -0.854622

H -4.750941 4.464953 -1.825375

H -3.922563 2.160920 -1.578338

H -0.406372 3.617862 0.372201

H -1.210039 5.940292 0.104384

H -3.406969 6.378296 -0.980119

C -1.585395 1.349921 -0.335627

H -0.539936 1.340835 -0.015505

C -2.338230 0.220231 0.053663

C -1.655618 -1.015615 0.295149

C -3.741938 0.241690 0.308347

C -2.282324 -2.148646 0.732373

H -0.584051 -1.023060 0.105110

C -4.437481 -0.841602 0.764664

H -4.257942 1.188272 0.206635

C -3.739204 -2.121947 0.966555

O -4.355662 -3.136633 1.350452

C -1.527507 -3.451393 1.007580

C -2.066479 -4.567576 0.093474

H -1.510338 -5.496352 0.275210

H -3.126634 -4.742645 0.280156

H -1.927626 -4.288299 -0.956838

C -0.029015 -3.314358 0.727691

H 0.161522 -3.089039 -0.325012

H 0.434104 -2.529636 1.341491

H 0.477874 -4.258370 0.958123

C -1.699502 -3.857150 2.482551

H -2.754732 -3.957407 2.740904

H -1.193727 -4.814103 2.664336

H -1.241686 -3.107002 3.140039

C -5.924489 -0.756457 1.114297

C -6.729178 -1.732653 0.237581

H -6.371909 -2.752833 0.380607

H -7.794949 -1.682457 0.497344

H -6.623265 -1.465964 -0.821067

C -6.488811 0.651101 0.888405

H -5.980058 1.397614 1.510030

H -6.402128 0.961825 -0.159975

H -7.552520 0.661449 1.153069

C -6.126085 -1.103991 2.600660

H -7.191693 -1.054204 2.860696

H -5.751995 -2.106835 2.811172

H -5.587179 -0.384903 3.230275

**C-4f-RS-TS4**

Zero-point correction= 1.057674 (Hartree/Particle)

Thermal correction to Energy= 1.117617

Thermal correction to Enthalpy= 1.118561

Thermal correction to Gibbs Free Energy= 0.962585

E(solv) = -5173.60473713 A.U.

C 0.909171 2.118632 -0.442971

N 0.433324 0.845191 -0.550898

C -0.557860 0.329459 0.114508

C -0.963974 -1.054753 -0.148479

C -0.214485 -1.859394 -1.089121

C -0.591317 -3.167194 -1.318780

C -1.741216 -3.744743 -0.725493

C -2.499463 -2.942603 0.178258

C -2.073259 -1.612869 0.476367

C -2.800190 -0.836988 1.527744

C -2.613927 -1.209267 2.901002

C -3.218080 -0.432301 3.928187

C -3.986899 0.704339 3.570307

C -4.166191 1.032462 2.256219

C -3.589853 0.256370 1.212139

C -2.171639 -5.071855 -0.987585

C -3.306254 -5.576630 -0.404158

C -4.072501 -4.780007 0.475120

C -3.672776 -3.498535 0.758531

C -1.827852 -2.333741 3.282246

C -1.673603 -2.673285 4.602928

C -2.277367 -1.897159 5.621115

C -3.027117 -0.799094 5.286260

O 0.863390 -1.373665 -1.697145

H 1.694071 2.373798 -1.144892

H -1.126020 0.911921 0.834192

H -4.434930 1.304888 4.358205

H -4.766712 1.894944 1.981700

H -1.583907 -5.679443 -1.666084

H -3.617796 -6.593731 -0.623680

H -4.974293 -5.181150 0.927668

H -4.257557 -2.883607 1.436252

H -1.341622 -2.922920 2.512259

H -1.071064 -3.536110 4.869861

H -2.139335 -2.170022 6.663102

H -3.491702 -0.188295 6.057097

H 0.960963 -0.408067 -1.390396

C -3.834534 0.677934 -0.192605

C -3.608194 2.005680 -0.572408

C -4.301645 -0.215948 -1.156570

C -3.832410 2.412223 -1.880523

H -3.195983 2.703192 0.150394

C -4.529778 0.201855 -2.465384

H -4.489372 -1.250901 -0.883382

C -4.296337 1.522274 -2.857550

H -3.621500 3.445694 -2.145442

H -4.889441 -0.528092 -3.182502

C -4.510086 2.016279 -4.290516

C -5.546942 3.151978 -4.291543

H -5.705403 3.521207 -5.311817

H -6.506678 2.799672 -3.898794

H -5.219290 3.994369 -3.674849

C -3.177489 2.542589 -4.850032

H -2.790423 3.372922 -4.251626

H -2.420985 1.751780 -4.852864

H -3.311999 2.899215 -5.878245

C -5.014134 0.905031 -5.216701

H -4.299069 0.078199 -5.276705

H -5.976209 0.505323 -4.878352

H -5.152418 1.304012 -6.227429

C 0.106542 3.221348 0.026662

O 0.740370 4.413011 -0.221853

O -0.969471 3.160257 0.607170

C 0.036287 5.657583 -0.048940

C 1.021983 6.690565 -0.589438

H 1.226205 6.502465 -1.647492

H 0.613748 7.700445 -0.480738

H 1.966308 6.630585 -0.040552

C -0.255011 5.931487 1.427298

H 0.681584 5.985080 1.992782

H -0.765729 6.895925 1.527134

H -0.883359 5.144032 1.845989

C -1.246007 5.674868 -0.884495

H -1.653382 6.691303 -0.911308

H -1.020857 5.364499 -1.910378

H -1.994446 5.000029 -0.467694

C 0.117413 0.480699 3.916953

C 1.037258 0.477290 2.869361

C 1.485138 1.683967 2.307322

C 0.975602 2.878861 2.848897

C 0.052381 2.883001 3.881368

C -0.386028 1.674782 4.420563

H -0.206578 -0.462548 4.344653

H 1.430552 -0.470940 2.526310

H 1.309894 3.820357 2.420455

H -0.325320 3.828095 4.262092

H -1.112509 1.657957 5.228392

C 2.462102 1.826007 1.218171

H 2.777869 2.863785 1.104675

C 3.386888 0.906504 0.693328

C 4.573881 1.446154 0.107165

C 3.221720 -0.512031 0.616552

C 5.573763 0.684551 -0.422382

H 4.669416 2.529603 0.122734

C 4.172728 -1.345694 0.098909

H 2.272356 -0.932199 0.923643

C 5.430311 -0.780823 -0.426637

O 6.333205 -1.513742 -0.878646

C 6.847195 1.296840 -1.010698

C 6.986879 0.896870 -2.490960

H 7.902033 1.332648 -2.912861

H 7.028779 -0.188178 -2.589665

H 6.133417 1.271953 -3.067275

C 6.832170 2.828308 -0.942649

H 5.990552 3.250579 -1.503298

H 6.772618 3.188347 0.090771

H 7.757667 3.219167 -1.380861

C 8.077745 0.805192 -0.226784

H 8.144681 -0.282358 -0.268938

H 8.992325 1.239632 -0.651400

H 8.008038 1.116093 0.822155

C 3.966438 -2.861142 0.011806

C 3.998340 -3.299913 -1.463855

H 4.963533 -3.059261 -1.912424

H 3.828225 -4.382423 -1.534571

H 3.199546 -2.797971 -2.019282

C 2.619875 -3.294586 0.601716

H 2.539206 -3.030642 1.664082

H 1.783645 -2.845182 0.061442

H 2.519393 -4.382537 0.513996

C 5.071591 -3.589134 0.799923

H 4.914169 -4.673767 0.739156

H 6.056230 -3.347225 0.399974

H 5.037063 -3.300992 1.857591

Br 0.517848 -4.188346 -2.460312

**C-4f-SR-TS1**

Zero-point correction= 1.056709 (Hartree/Particle)

Thermal correction to Energy= 1.116956

Thermal correction to Enthalpy= 1.117900

Thermal correction to Gibbs Free Energy= 0.960508

E(solv) = -5173.60957631 A.U.

C -0.836317 -1.129282 -1.905986

N 0.025017 -0.260750 -1.327824

C 0.976560 -0.647584 -0.533327

C 1.648737 0.312380 0.329412

C 1.302291 1.716565 0.249169

C 1.847379 2.594135 1.162700

C 2.746931 2.180214 2.180748

C 3.081245 0.794798 2.257760

C 2.528252 -0.116506 1.313378

C 2.783177 -1.584216 1.455101

C 1.919290 -2.328156 2.326696

C 2.058434 -3.740172 2.425948

C 3.064849 -4.384270 1.661832

C 3.870631 -3.661148 0.828433

C 3.738236 -2.247580 0.702961

C 3.312326 3.068610 3.130910

C 4.162277 2.615156 4.109542

C 4.489897 1.244499 4.192735

C 3.957242 0.359901 3.288527

C 0.883718 -1.697136 3.071694

C 0.045957 -2.430398 3.874849

C 0.195983 -3.833737 3.979218

C 1.181907 -4.470738 3.269858

O 0.454434 2.165444 -0.675055

Br 1.310045 4.403981 1.040499

H -0.557936 -2.173104 -2.044840

H 1.216818 -1.709463 -0.420875

H 3.178667 -5.462407 1.744506

H 4.640583 -4.158746 0.245340

H 3.054889 4.119942 3.070546

H 4.580903 3.316538 4.825372

H 5.158153 0.890332 4.971928

H 4.202552 -0.697175 3.347303

H 0.757395 -0.622092 2.990069

H -0.746033 -1.931371 4.424230

H -0.477640 -4.403164 4.612389

H 1.304392 -5.549531 3.335186

H 0.068347 1.360742 -1.152818

C 4.595360 -1.556460 -0.297566

C 4.701880 -2.075585 -1.589026

C 5.323588 -0.399706 0.004080

C 5.499980 -1.460795 -2.551213

H 4.130510 -2.961501 -1.854783

C 6.115785 0.208471 -0.958714

H 5.276156 0.021522 1.003181

C 6.223463 -0.303753 -2.258700

H 5.539746 -1.896717 -3.543882

H 6.661239 1.108407 -0.686063

C 7.092284 0.418784 -3.290106

C 8.530130 0.543820 -2.759837

H 9.159503 1.060986 -3.493629

H 8.567751 1.112636 -1.826123

H 8.961539 -0.444911 -2.570886

C 7.139682 -0.325638 -4.627947

H 7.551664 -1.334029 -4.511189

H 6.145392 -0.408741 -5.078571

H 7.779716 0.220911 -5.328971

C 6.516223 1.823330 -3.536197

H 5.493365 1.758906 -3.920604

H 6.489974 2.412616 -2.614627

H 7.129132 2.362632 -4.268329

C -1.846664 -0.612146 -2.822388

O -1.921859 0.729764 -2.766314

O -2.580514 -1.318352 -3.496127

C -2.920586 1.462610 -3.515061

C -2.738727 1.227053 -5.013624

H -2.987152 0.198943 -5.279384

H -3.389409 1.910840 -5.569711

H -1.701064 1.430266 -5.299282

C -2.599113 2.908643 -3.154145

H -1.595385 3.176883 -3.498316

H -3.327611 3.588197 -3.607900

H -2.631357 3.025531 -2.067045

C -4.321161 1.093920 -3.037412

H -4.408023 1.272134 -1.961258

H -5.058096 1.719798 -3.553778

H -4.539692 0.043505 -3.238942

C -3.009061 -5.010068 -1.339406

C -2.959179 -3.622235 -1.264372

C -2.196287 -2.996490 -0.267735

C -1.455482 -3.795244 0.613938

C -1.513036 -5.183238 0.538679

C -2.297164 -5.796710 -0.434469

H -3.598598 -5.481315 -2.120854

H -3.460982 -3.011261 -2.008878

H -0.834642 -3.318529 1.370029

H -0.936676 -5.779889 1.240938

H -2.342191 -6.880310 -0.499486

C -2.082172 -1.526500 -0.135081

H -1.187858 -1.226820 0.414463

C -3.174303 -0.646940 0.008753

C -2.911717 0.691337 0.441971

C -4.538569 -1.025040 -0.156442

C -3.894973 1.583961 0.763078

H -1.864862 0.982542 0.516607

C -5.579170 -0.183580 0.120891

H -4.743012 -2.038879 -0.479200

C -5.305853 1.166138 0.646525

O -6.236588 1.924798 0.988936

C -3.584195 2.984770 1.299292

C -4.096761 3.096226 2.747476

H -3.880204 4.096485 3.144459

H -5.173103 2.921534 2.787989

H -3.592578 2.361290 3.385754

C -2.080418 3.281450 1.303276

H -1.522716 2.596289 1.954317

H -1.636938 3.219447 0.303747

H -1.910041 4.296683 1.679903

C -4.272409 4.060367 0.438570

H -5.352054 3.909428 0.423164

H -4.053208 5.055563 0.846562

H -3.896136 4.030497 -0.590077

C -7.036387 -0.606354 -0.077846

C -7.788869 -0.589219 1.264951

H -7.757201 0.407699 1.705687

H -8.835707 -0.884293 1.112824

H -7.333202 -1.300888 1.963424

C -7.148632 -2.020191 -0.659925

H -6.640518 -2.099234 -1.628112

H -6.724283 -2.773992 0.013037

H -8.206289 -2.264874 -0.812489

C -7.718587 0.357827 -1.065784

H -8.769975 0.073283 -1.205048

H -7.672474 1.381689 -0.692826

H -7.221453 0.312323 -2.042244

**C-4f-SR-TS2**

Zero-point correction= 1.057087 (Hartree/Particle)

Thermal correction to Energy= 1.117483

Thermal correction to Enthalpy= 1.118427

Thermal correction to Gibbs Free Energy= 0.958885

E(solv) = -5173.60778620 A.U.

C 0.918877 -0.519940 1.321977

N -0.075495 0.276414 0.870587

C -1.036866 -0.176425 0.121626

C -2.041537 0.720621 -0.429509

C -2.032724 2.124198 -0.070700

C -2.999025 2.956484 -0.597906

C -3.991748 2.503813 -1.506068

C -3.979166 1.126095 -1.878640

C -3.002803 0.254110 -1.315780

C -2.962304 -1.176109 -1.751462

C -2.277355 -1.492955 -2.970243

C -2.178303 -2.846900 -3.395005

C -2.755731 -3.862459 -2.590559

C -3.382008 -3.543803 -1.418625

C -3.496649 -2.193991 -0.979764

C -4.983990 3.349318 -2.065872

C -5.915876 2.863238 -2.949339

C -5.904986 1.501739 -3.322334

C -4.956954 0.658762 -2.798609

C -1.649216 -0.488495 -3.758835

C -0.961758 -0.817054 -4.899990

C -0.873546 -2.164404 -5.324231

C -1.475281 -3.153069 -4.589683

O -1.114814 2.623718 0.750277

Br -2.948655 4.776742 -0.075658

H 0.769372 -1.593748 1.398663

H -1.107444 -1.242405 -0.114384

H -2.679056 -4.897675 -2.914659

H -3.816270 -4.323307 -0.798900

H -4.991048 4.395200 -1.781471

H -6.664705 3.532018 -3.363532

H -6.642888 1.122880 -4.023035

H -4.943372 -0.390049 -3.081193

H -1.710326 0.545475 -3.435502

H -0.477012 -0.038462 -5.480561

H -0.324625 -2.410803 -6.227921

H -1.410767 -4.193294 -4.901430

H -0.473711 1.885950 0.994945

C -4.129304 -1.941265 0.342302

C -3.690620 -2.650286 1.461162

C -5.165591 -1.016344 0.514204

C -4.257379 -2.438068 2.716398

H -2.873531 -3.359664 1.353373

C -5.728760 -0.813352 1.765633

H -5.534995 -0.457383 -0.340306

C -5.287262 -1.513784 2.896877

H -3.870292 -3.000626 3.559034

H -6.529845 -0.084751 1.860873

C -5.930523 -1.237656 4.257935

C -7.435104 -1.547582 4.184853

H -7.910397 -1.349284 5.152722

H -7.936013 -0.932334 3.431341

H -7.601794 -2.598872 3.927283

C -5.317656 -2.093564 5.370888

H -5.448997 -3.163230 5.174791

H -4.247863 -1.892549 5.488579

H -5.808727 -1.863766 6.322471

C -5.731744 0.243066 4.623089

H -4.666647 0.489125 4.675008

H -6.191867 0.905192 3.883714

H -6.185623 0.456728 5.598000

C 1.910094 0.099861 2.185218

O 2.801704 -0.834965 2.605575

O 1.986115 1.285492 2.448749

C 3.873630 -0.485248 3.511238

C 4.763091 0.620213 2.950269

H 4.234566 1.571764 2.911261

H 5.651770 0.721459 3.584278

H 5.088853 0.358104 1.940131

C 4.668154 -1.784476 3.607311

H 5.068371 -2.041487 2.619945

H 5.504297 -1.669919 4.304291

H 4.027025 -2.602157 3.952185

C 3.278942 -0.103986 4.865617

H 4.082590 0.073759 5.588133

H 2.680898 0.804621 4.771236

H 2.644683 -0.913653 5.241669

C 2.261829 -4.511998 -0.052020

C 2.492048 -3.153191 0.116288

C 1.910258 -2.214368 -0.752031

C 1.060047 -2.675682 -1.765123

C 0.833587 -4.041004 -1.932407

C 1.437561 -4.962824 -1.083778

H 2.716695 -5.222886 0.632015

H 3.086820 -2.789228 0.950665

H 0.583136 -1.961617 -2.434415

H 0.179358 -4.377698 -2.730811

H 1.257780 -6.026111 -1.214447

C 2.136719 -0.763490 -0.579398

H 1.346012 -0.138631 -0.994170

C 3.412037 -0.180497 -0.493203

C 3.506021 1.246069 -0.425316

C 4.635854 -0.916906 -0.505198

C 4.691191 1.919972 -0.400974

H 2.564227 1.785215 -0.358864

C 5.862057 -0.314391 -0.502510

H 4.571463 -1.996334 -0.573141

C 5.951388 1.159273 -0.484658

O 7.055748 1.737041 -0.537559

C 4.756208 3.444452 -0.286574

C 5.404336 4.036482 -1.550874

H 5.444979 5.130723 -1.473564

H 6.416270 3.649599 -1.679047

H 4.811577 3.780622 -2.436732

C 3.359784 4.057471 -0.128299

H 2.732311 3.874459 -1.007843

H 2.842003 3.658359 0.751051

H 3.452926 5.142805 -0.007696

C 5.581836 3.847245 0.949345

H 6.590488 3.436453 0.892962

H 5.644023 4.941327 1.011990

H 5.098492 3.484373 1.862954

C 7.161375 -1.123499 -0.522554

C 7.978037 -0.794877 -1.785546

H 8.219248 0.267850 -1.817454

H 8.908883 -1.376903 -1.792630

H 7.406906 -1.056183 -2.683958

C 6.896886 -2.633805 -0.518255

H 6.330822 -2.945002 0.367886

H 6.341439 -2.953058 -1.407197

H 7.853589 -3.168505 -0.510048

C 7.991598 -0.791752 0.731541

H 8.931548 -1.358854 0.721888

H 8.218086 0.274561 0.768230

H 7.437599 -1.067265 1.637546

**C-4f-SR-TS3**

Zero-point correction= 1.057247 (Hartree/Particle)

Thermal correction to Energy= 1.117199

Thermal correction to Enthalpy= 1.118144

Thermal correction to Gibbs Free Energy= 0.961973

E(solv) = -5173.61004576 A.U.

C 1.177785 1.601217 -0.229793

N -0.121892 1.340669 -0.473448

C -0.718492 0.273318 -0.032037

C -2.047544 -0.081373 -0.498729

C -2.676741 0.721173 -1.527178

C -3.846130 0.272573 -2.102438

C -4.527218 -0.888397 -1.655210

C -3.951555 -1.625626 -0.575724

C -2.699841 -1.213959 -0.025911

C -2.096562 -2.002048 1.093992

C -1.526624 -3.291553 0.832366

C -0.951007 -4.040639 1.896518

C -0.920861 -3.477117 3.197272

C -1.442144 -2.234140 3.423917

C -2.048717 -1.482683 2.378779

C -5.752279 -1.337099 -2.212071

C -6.395285 -2.443137 -1.714219

C -5.846965 -3.157309 -0.627230

C -4.656234 -2.755064 -0.075881

C -1.504776 -3.850642 -0.474965

C -0.957028 -5.087757 -0.701783

C -0.401749 -5.837618 0.362422

C -0.399304 -5.321362 1.631486

O -2.134389 1.858409 -1.949359

H 1.708568 1.078202 0.562392

H -0.207652 -0.413818 0.649755

H -0.467188 -4.044978 4.005759

H -1.419585 -1.801504 4.420192

H -6.177693 -0.780450 -3.039307

H -7.334153 -2.764593 -2.155693

H -6.364722 -4.024332 -0.228305

H -4.232100 -3.304431 0.758856

H -1.919026 -3.273915 -1.295349

H -0.947346 -5.496601 -1.708234

H 0.030615 -6.814344 0.167499

H 0.040829 -5.876122 2.456563

H -1.244560 1.965847 -1.477067

C -2.579907 -0.133398 2.709152

C -1.760976 0.798709 3.345380

C -3.893705 0.241251 2.404895

C -2.230543 2.073192 3.658459

H -0.730751 0.534461 3.572223

C -4.356689 1.508876 2.723470

H -4.552294 -0.468833 1.912314

C -3.536465 2.457044 3.351922

H -1.550236 2.770846 4.134536

H -5.381271 1.766835 2.467767

C -4.089569 3.850627 3.658997

C -5.304615 3.724385 4.593293

H -5.715616 4.715932 4.816976

H -6.099687 3.124717 4.139799

H -5.019356 3.247872 5.537207

C -3.050810 4.746936 4.340430

H -2.719022 4.324628 5.295138

H -2.172198 4.897044 3.704784

H -3.490699 5.729446 4.542431

C -4.522357 4.525335 2.346372

H -3.674348 4.617667 1.660883

H -5.302057 3.951316 1.836959

H -4.917355 5.528032 2.548529

C 1.685320 2.884041 -0.678992

O 2.901510 3.116641 -0.122077

O 1.153096 3.623352 -1.493157

C 3.683011 4.268009 -0.497325

C 4.047201 4.210833 -1.980297

H 3.149931 4.260564 -2.599014

H 4.703596 5.051230 -2.231173

H 4.585319 3.282253 -2.198206

C 4.932553 4.117141 0.365092

H 5.363594 3.123076 0.212169

H 5.675293 4.878638 0.106075

H 4.677438 4.215871 1.424645

C 2.944056 5.560222 -0.149861

H 2.621991 5.529661 0.896009

H 3.619505 6.413365 -0.277679

H 2.068742 5.691681 -0.786404

C -0.730868 -0.144263 -3.752782

C 0.356655 0.462089 -3.140758

C 1.313967 -0.292062 -2.437728

C 1.164872 -1.686607 -2.412839

C 0.081760 -2.296159 -3.041411

C -0.879152 -1.530772 -3.695218

H -1.494592 0.467021 -4.226322

H 0.453342 1.546234 -3.150611

H 1.918454 -2.301471 -1.933067

H -0.007515 -3.378351 -3.011506

H -1.739227 -2.005496 -4.159700

C 2.434908 0.412628 -1.799847

H 2.730885 1.315688 -2.336925

C 3.442454 -0.164205 -1.001663

C 4.766058 0.360693 -1.038491

C 3.180696 -1.200710 -0.057668

C 5.797507 -0.148500 -0.297878

H 4.950165 1.165020 -1.746424

C 4.138871 -1.716720 0.768127

H 2.151542 -1.534533 0.037293

C 5.525958 -1.228552 0.663971

O 6.434248 -1.707595 1.376703

C 7.237961 0.350348 -0.445672

C 8.150149 -0.817464 -0.867297

H 9.184041 -0.464669 -0.975779

H 8.119708 -1.613373 -0.122723

H 7.825487 -1.221810 -1.833091

C 7.366989 1.438952 -1.518284

H 7.047190 1.076858 -2.502079

H 6.781067 2.332703 -1.275527

H 8.416784 1.743993 -1.597617

C 7.739475 0.933716 0.887704

H 7.685658 0.179413 1.673729

H 8.778800 1.271719 0.783294

H 7.130362 1.796178 1.182477

C 3.812946 -2.788899 1.809863

C 4.611929 -4.069972 1.507715

H 5.682343 -3.861895 1.518836

H 4.385924 -4.838655 2.258170

H 4.335925 -4.464829 0.523104

C 2.324117 -3.154205 1.811915

H 1.687626 -2.294132 2.054959

H 1.991683 -3.562000 0.849263

H 2.140778 -3.922599 2.572964

C 4.170392 -2.279367 3.218208

H 3.936173 -3.049255 3.964796

H 5.230898 -2.033240 3.278547

H 3.583766 -1.384816 3.457112

Br -4.520157 1.280180 -3.559605

**C-4f-SR-TS4**

Zero-point correction= 1.057871 (Hartree/Particle)

Thermal correction to Energy= 1.117449

Thermal correction to Enthalpy= 1.118393

Thermal correction to Gibbs Free Energy= 0.965911

E(solv) = -5173.61128387 A.U.

C -0.477549 0.704202 -2.561612

N 0.790305 0.332202 -2.241949

C 1.535051 0.801986 -1.282676

C 2.741318 0.070657 -0.889570

C 3.064736 -1.180324 -1.549959

C 4.079516 -1.965544 -1.046412

C 4.894323 -1.561438 0.041346

C 4.622603 -0.296272 0.644029

C 3.525572 0.491737 0.176168

C 3.262923 1.815629 0.825837

C 4.082103 2.929243 0.441604

C 3.858911 4.208304 1.022935

C 2.810483 4.359601 1.964907

C 2.030932 3.291785 2.307964

C 2.248776 1.998163 1.752219

C 5.959076 -2.347114 0.550717

C 6.726731 -1.906754 1.600259

C 6.465456 -0.655827 2.198741

C 5.438668 0.125538 1.728798

C 5.122210 2.800251 -0.520485

C 5.898893 3.875893 -0.869182

C 5.680793 5.142932 -0.278760

C 4.680323 5.301622 0.644394

O 2.386763 -1.585287 -2.615188

H -0.895065 0.219900 -3.437392

H 1.250836 1.688947 -0.720908

H 2.637214 5.337486 2.407384

H 1.231067 3.408709 3.033439

H 6.156138 -3.308301 0.089784

H 7.537962 -2.526642 1.971235

H 7.073608 -0.312150 3.030105

H 5.232856 1.086362 2.191450

H 5.291619 1.832619 -0.981091

H 6.685574 3.754901 -1.607777

H 6.302101 5.986973 -0.563565

H 4.494818 6.270987 1.101063

H 1.597812 -0.944811 -2.704521

C 1.332686 0.907962 2.184362

C -0.051576 1.120473 2.157060

C 1.798988 -0.315637 2.670539

C -0.933193 0.134106 2.581191

H -0.438109 2.056945 1.756503

C 0.907709 -1.300913 3.093398

H 2.868393 -0.500523 2.722512

C -0.476188 -1.106350 3.041995

H -2.001754 0.323226 2.515274

H 1.314387 -2.242827 3.446805

C -1.490983 -2.177073 3.448412

C -2.203810 -1.726741 4.733829

H -2.952651 -2.469324 5.034034

H -1.487906 -1.604023 5.554409

H -2.716364 -0.770720 4.584991

C -2.530542 -2.365132 2.328294

H -3.128642 -1.464090 2.151284

H -2.042820 -2.629752 1.383379

H -3.222572 -3.171437 2.599721

C -0.814545 -3.527646 3.703472

H -0.279358 -3.872716 2.811398

H -0.105995 -3.477539 4.537756

H -1.573294 -4.276230 3.955401

C -1.055829 1.984673 -2.210952

O -2.131639 2.238437 -2.999827

O -0.676704 2.730507 -1.315889

C -2.773550 3.530224 -2.993299

C -3.380301 3.844635 -1.629766

H -2.598934 4.064505 -0.901565

H -4.050365 4.708428 -1.713028

H -3.958778 2.984340 -1.278066

C -3.877307 3.368498 -4.034479

H -4.572341 2.582705 -3.724600

H -4.430444 4.306113 -4.148206

H -3.446815 3.090990 -5.001419

C -1.777253 4.602955 -3.430927

H -1.350103 4.343016 -4.405097

H -2.288640 5.567335 -3.522116

H -0.970767 4.697187 -2.701619

C -0.400372 -3.726576 -2.493223

C -1.071992 -2.534001 -2.244398

C -0.739880 -1.750079 -1.132839

C 0.286378 -2.189452 -0.287438

C 0.952204 -3.387190 -0.531803

C 0.615296 -4.155994 -1.641116

H -0.655625 -4.311537 -3.372250

H -1.833690 -2.182481 -2.935990

H 0.579425 -1.563493 0.551344

H 1.775763 -3.687790 0.111069

H 1.163653 -5.067834 -1.857530

C -1.387558 -0.454574 -0.816591

H -0.738413 0.233644 -0.272259

C -2.757559 -0.320478 -0.547784

C -3.198484 0.812296 0.208675

C -3.719944 -1.344869 -0.806497

C -4.461543 0.927473 0.718224

H -2.450861 1.578299 0.409016

C -5.014633 -1.275949 -0.383231

H -3.375749 -2.230221 -1.327027

C -5.466225 -0.102122 0.388269

O -6.647625 0.001684 0.772400

C -4.865008 2.055215 1.673376

C -5.327152 1.441420 3.008891

H -5.598168 2.238108 3.713590

H -6.189061 0.790580 2.858187

H -4.514748 0.855246 3.457613

C -3.692812 2.992743 1.986668

H -2.864404 2.460871 2.470709

H -3.305330 3.484183 1.089342

H -4.032157 3.773695 2.676839

C -6.007601 2.899645 1.080811

H -6.869212 2.269831 0.856690

H -6.307053 3.676141 1.796807

H -5.682078 3.399023 0.161372

C -6.014062 -2.404064 -0.649733

C -6.537792 -2.967382 0.684171

H -7.034852 -2.186532 1.260905

H -7.249324 -3.781558 0.494863

H -5.707503 -3.371607 1.275295

C -5.374375 -3.560894 -1.425495

H -5.001224 -3.234905 -2.403136

H -4.540560 -4.009571 -0.873406

H -6.125235 -4.340960 -1.595003

C -7.193553 -1.873438 -1.484543

H -7.914496 -2.680086 -1.671304

H -7.696262 -1.059733 -0.960885

H -6.836997 -1.505002 -2.453467

Br 4.330460 -3.658721 -1.858839

**M-ent-3b-RR-TS1**

Zero-point correction= 0.688215 (Hartree/Particle)

Thermal correction to Energy= 0.731859

Thermal correction to Enthalpy= 0.732803

Thermal correction to Gibbs Free Energy= 0.609719

E(solv) = -5075.90255177 A.U.

C 4.178717 -4.750693 -2.705906

C 4.929208 -3.592844 -2.397451

C 4.416525 -2.628412 -1.564065

C 3.126376 -2.769520 -0.987255

C 2.367610 -3.933360 -1.306325

C 2.924878 -4.909660 -2.170553

C 2.570959 -1.793959 -0.107654

C 1.316069 -1.982168 0.445546

C 0.508958 -3.116253 0.057637

C 1.060730 -4.060592 -0.777336

C 3.288267 -0.529073 0.215891

C 4.384786 -0.508952 1.128035

C 5.039029 0.717801 1.434375

C 4.556876 1.915003 0.850015

C 3.498539 1.874434 -0.011302

C 2.851140 0.652957 -0.368316

C 4.866727 -1.696411 1.745970

C 5.936843 -1.662570 2.603575

C 6.587774 -0.440888 2.899120

C 6.142020 0.722037 2.326458

O 0.878488 -1.089377 1.337026

C -0.887717 -3.209242 0.467617

O 1.875446 0.739301 -1.275638

Br 2.810192 3.481752 -0.732257

N -1.424838 -2.258495 1.148302

C -2.757226 -2.117472 1.402232

C -3.136146 -1.060721 2.317433

C -4.425808 -1.008997 2.884964

C -4.782041 0.090612 3.645793

C -3.859562 1.122350 3.828352

C -2.613609 0.988419 3.222745

N -2.248816 -0.061321 2.491114

H 4.592316 -5.504694 -3.369464

H 5.918412 -3.462835 -2.827614

H 4.995078 -1.738532 -1.334434

H 2.330400 -5.789682 -2.407115

H 0.459764 -4.920165 -1.071677

H 5.021633 2.864587 1.097403

H 4.366168 -2.634082 1.525831

H 6.287533 -2.582198 3.063341

H 7.434397 -0.428277 3.579315

H 6.625940 1.671178 2.545935

H -0.113426 -1.218186 1.442259

H -1.464737 -4.077332 0.113261

H 1.084307 0.135556 -1.128563

H -3.404753 -2.996343 1.334910

H -5.121832 -1.825086 2.710327

H -5.771700 0.148371 4.092962

H -4.098543 2.004330 4.413153

H -1.863257 1.773946 3.319426

C -5.142644 -2.834474 -0.767145

C -4.765359 -1.532608 -0.417535

C -5.754067 -0.625968 -0.022309

C -7.088276 -1.019869 0.031619

C -7.454791 -2.318427 -0.314493

C -6.474695 -3.226324 -0.717888

C -3.338949 -1.121624 -0.442617

N -3.081357 0.179159 -0.417146

P -1.581952 0.726038 -0.783780

C -1.773058 1.718442 -2.308893

C -0.659646 2.428848 -2.774828

C -2.971860 1.763018 -3.019390

C -0.753054 3.172354 -3.945948

C -3.061145 2.510068 -4.193510

C -1.953017 3.213297 -4.656541

C -1.163233 2.028141 0.418061

C 0.047590 1.996087 1.106663

C -2.040400 3.100662 0.602477

C 0.391274 3.046688 1.957340

C -1.704261 4.138566 1.464196

C -0.480715 4.117244 2.134926

O -0.472942 -0.286322 -0.976642

H -4.374261 -3.541371 -1.075846

H -5.444545 0.380686 0.240797

H -7.847251 -0.308569 0.346890

H -8.496921 -2.623159 -0.273743

H -6.751942 -4.239512 -0.995982

H -2.653579 -1.821445 -0.939683

H 0.274978 2.406327 -2.214565

H -3.827949 1.211211 -2.638859

H 0.112363 3.722133 -4.305475

H -3.996630 2.543288 -4.745533

H -2.022021 3.796535 -5.571143

H 0.702921 1.137474 0.998273

H -2.987427 3.115352 0.068020

H 1.347588 3.023872 2.473090

H -2.391202 4.968078 1.608255

H -0.209819 4.936771 2.795388

**M-ent-3b-RR-TS2**

Zero-point correction= 0.688282 (Hartree/Particle)

Thermal correction to Energy= 0.731925

Thermal correction to Enthalpy= 0.732869

Thermal correction to Gibbs Free Energy= 0.610048

E(solv) = -5075.90270472 A.U.

C 4.225654 4.764260 2.681034

C 4.971174 3.601192 2.378763

C 4.448397 2.627592 1.562519

C 3.152973 2.764238 0.996403

C 2.398509 3.932567 1.311173

C 2.966134 4.918086 2.158204

C 2.589415 1.783272 0.127652

C 1.334173 1.974970 -0.423566

C 0.530187 3.111085 -0.035801

C 1.087518 4.058099 0.792137

C 3.299714 0.514388 -0.195180

C 4.394571 0.487631 -1.109740

C 5.038264 -0.743767 -1.420796

C 4.547531 -1.938982 -0.838881

C 3.489868 -1.892154 0.022700

C 2.852370 -0.666428 0.384300

C 4.883495 1.672851 -1.726931

C 5.950951 1.632884 -2.587325

C 6.591524 0.406587 -2.887684

C 6.138728 -0.754198 -2.316433

O 0.892843 1.083937 -1.314747

C -0.865564 3.207816 -0.446041

O 1.876555 -0.749687 1.292174

Br 2.786897 -3.495319 0.739931

N -1.404438 2.261665 -1.131982

C -2.736761 2.133809 -1.393312

C -3.123082 1.088548 -2.317085

C -4.412115 1.056886 -2.888568

C -4.781539 -0.034572 -3.654412

C -3.872506 -1.078332 -3.838227

C -2.625922 -0.962974 -3.230010

N -2.248086 0.078833 -2.493756

H 4.648487 5.526874 3.328611

H 5.965150 3.475279 2.798786

H 5.023148 1.734195 1.337208

H 2.375296 5.802082 2.388775

H 0.490133 4.920588 1.084976

H 5.004712 -2.891364 -1.089386

H 4.390335 2.613417 -1.503146

H 6.306547 2.550401 -3.047214

H 7.434632 0.388895 -3.572070

H 6.613935 -1.706458 -2.540856

H -0.098086 1.219553 -1.423227

H -1.440508 4.076825 -0.091290

H 1.085622 -0.147380 1.144730

H -3.375978 3.018201 -1.320651

H -5.097042 1.881782 -2.711880

H -5.770740 -0.076425 -4.104086

H -4.121345 -1.955085 -4.426685

H -1.886165 -1.758349 -3.327798

C -5.129114 2.838244 0.779699

C -4.757808 1.540806 0.407948

C -5.749102 0.647000 -0.010004

C -7.081150 1.049104 -0.061378

C -7.442055 2.342707 0.308667

C -6.458957 3.237989 0.732931

C -3.334134 1.121501 0.436463

N -3.082554 -0.179938 0.394974

P -1.592179 -0.738637 0.779454

C -1.815795 -1.733363 2.297494

C -0.719255 -2.457541 2.782281

C -3.025082 -1.758449 2.990937

C -0.840003 -3.194556 3.955442

C -3.141761 -2.498254 4.167273

C -2.050225 -3.214614 4.649392

C -1.160670 -2.032161 -0.426248

C 0.051854 -1.979363 -1.110898

C -2.026337 -3.111339 -0.628492

C 0.407654 -3.012166 -1.977719

C -1.677004 -4.133029 -1.504765

C -0.453040 -4.088364 -2.174048

O -0.480564 0.266638 0.993561

H -4.357838 3.534489 1.104215

H -5.442870 -0.355878 -0.290624

H -7.842734 0.348093 -0.392429

H -8.482412 2.653337 0.270062

H -6.732433 4.246921 1.029305

H -2.648420 1.811672 0.945997

H 0.223548 -2.449102 2.235813

H -3.867683 -1.197086 2.595457

H 0.012030 -3.754290 4.330708

H -4.085395 -2.515467 4.705804

H -2.140748 -3.791873 5.565765

H 0.700930 -1.118594 -0.986926

H -2.974631 -3.144625 -0.097332

H 1.364096 -2.971012 -2.491751

H -2.354106 -4.968088 -1.662207

H -0.173369 -4.893875 -2.847830

**M-ent-3b-RR-TS3**

Zero-point correction= 0.689847 (Hartree/Particle)

Thermal correction to Energy= 0.733099

Thermal correction to Enthalpy= 0.734043

Thermal correction to Gibbs Free Energy= 0.611346

E(solv) = -5075.90237256 A.U.

C 2.358787 5.447196 -0.283169

C 3.445145 4.714365 0.245802

C 3.470917 3.342779 0.165159

C 2.413871 2.618693 -0.449963

C 1.298388 3.359675 -0.938820

C 1.308269 4.776454 -0.855737

C 2.408929 1.187776 -0.566553

C 1.284149 0.554509 -1.068540

C 0.126576 1.295799 -1.503511

C 0.177241 2.669963 -1.455840

C 3.565123 0.375304 -0.092411

C 4.885789 0.579705 -0.610171

C 5.991639 -0.103151 -0.029135

C 5.763981 -1.020741 1.026377

C 4.488801 -1.270332 1.443231

C 3.369511 -0.592633 0.884877

C 5.142237 1.442983 -1.711768

C 6.416911 1.635849 -2.179954

C 7.515709 0.979664 -1.576173

C 7.301420 0.125528 -0.526663

O 1.269124 -0.793295 -1.112948

C -1.076592 0.599267 -1.934922

O 2.147959 -0.912621 1.354908

Br 4.171065 -2.553223 2.796189

N -1.064758 -0.698121 -2.028018

C -2.183958 -1.419725 -2.241552

C -2.056026 -2.839643 -2.499698

C -3.185644 -3.588459 -2.890316

C -3.078123 -4.962574 -3.018601

C -1.851857 -5.574419 -2.759520

C -0.789301 -4.750628 -2.391927

N -0.865743 -3.430776 -2.266388

H 2.352839 6.531832 -0.222871

H 4.266255 5.237966 0.727387

H 4.307383 2.797474 0.589273

H 0.448494 5.314428 -1.247973

H -0.693563 3.244597 -1.770811

H 6.600250 -1.545424 1.477950

H 4.305014 1.942340 -2.187266

H 6.584392 2.295141 -3.026643

H 8.520489 1.144305 -1.954108

H 8.130194 -0.401388 -0.059498

H 0.352537 -1.084050 -1.509468

H -1.951312 1.191269 -2.220945

H 1.567840 -1.030815 0.561968

H -3.078125 -0.905318 -2.601436

H -4.125346 -3.075617 -3.078025

H -3.940608 -5.553943 -3.316677

H -1.718550 -6.647933 -2.841823

H 0.187844 -5.185270 -2.181910

C -2.549726 -3.724789 0.433994

C -2.123104 -2.393211 0.456153

C -0.883374 -2.086444 1.028653

C -0.076587 -3.095358 1.547683

C -0.507104 -4.421689 1.505297

C -1.746915 -4.734108 0.952855

C -2.957440 -1.337809 -0.154847

N -2.777489 -0.079594 0.218110

P -3.891268 1.048711 -0.226607

C -5.210307 0.968298 1.044291

C -6.510249 1.331231 0.686834

C -4.943178 0.562225 2.354118

C -7.532677 1.299384 1.631978

C -5.965077 0.530548 3.298795

C -7.259370 0.902369 2.938955

C -3.031604 2.617225 0.100391

C -3.439768 3.749803 -0.607621

C -1.984840 2.714387 1.021129

C -2.818830 4.976834 -0.382922

C -1.362853 3.939849 1.241952

C -1.785685 5.072094 0.547596

O -4.520743 1.047489 -1.592749

H -3.510082 -3.967095 -0.015044

H -0.576979 -1.043036 1.059945

H 0.894746 -2.851027 1.970430

H 0.126926 -5.209205 1.903453

H -2.086330 -5.765874 0.914626

H -3.915373 -1.689779 -0.560328

H -6.704778 1.618410 -0.343242

H -3.934064 0.256110 2.618119

H -8.544153 1.578102 1.348929

H -5.754148 0.210545 4.315402

H -8.056838 0.875055 3.676841

H -4.231045 3.647954 -1.345998

H -1.641744 1.815218 1.524940

H -3.135136 5.856327 -0.937445

H -0.528364 4.008506 1.933719

H -1.290924 6.024934 0.716682

**M-ent-3b-RR-TS4**

Zero-point correction= 0.689656 (Hartree/Particle)

Thermal correction to Energy= 0.733415

Thermal correction to Enthalpy= 0.734359

Thermal correction to Gibbs Free Energy= 0.608625

E(solv) = -5075.89546061 A.U.

C 4.508736 5.159706 -0.269166

C 5.480015 4.200654 0.096577

C 5.193534 2.858042 0.048394

C 3.917769 2.391561 -0.367752

C 2.931730 3.363601 -0.705762

C 3.260927 4.743043 -0.655444

C 3.584016 0.996161 -0.429150

C 2.281032 0.633392 -0.712143

C 1.281480 1.605641 -1.059651

C 1.632584 2.934355 -1.063119

C 4.621419 -0.046810 -0.187146

C 5.796124 -0.095198 -1.009331

C 6.847820 -0.998560 -0.692463

C 6.695403 -1.875746 0.409418

C 5.534442 -1.876477 1.126386

C 4.469827 -0.976640 0.831835

C 5.952115 0.726991 -2.159855

C 7.091474 0.672042 -2.921644

C 8.147459 -0.205319 -2.581448

C 8.020846 -1.025772 -1.491557

O 1.933516 -0.671134 -0.647155

C -0.091454 1.202752 -1.322803

O 3.358503 -1.060951 1.591306

Br 5.312005 -3.105691 2.542874

N -0.467261 -0.028628 -1.216270

C -1.809168 -0.360520 -1.191889

C -2.157042 -1.756139 -1.494769

C -1.241724 -2.818140 -1.391607

C -1.700799 -4.113115 -1.571449

C -3.053058 -4.328540 -1.833915

C -3.880577 -3.213772 -1.912941

N -3.452749 -1.963338 -1.768094

H 4.748148 6.218226 -0.231564

H 6.461714 4.527845 0.426425

H 5.946554 2.136271 0.346623

H 2.492682 5.464630 -0.923780

H 0.877710 3.680488 -1.304770

H 7.491303 -2.569260 0.661888

H 5.145335 1.395444 -2.439036

H 7.179887 1.305041 -3.799632

H 9.046211 -0.234583 -3.190089

H 8.813261 -1.720181 -1.222573

H 0.927608 -0.708778 -0.841027

H -0.814429 1.997377 -1.539280

H 2.587148 -0.936882 0.994297

H -2.492132 0.369936 -1.631256

H -0.196038 -2.621449 -1.179107

H -1.010846 -4.950143 -1.500304

H -3.455730 -5.327321 -1.964536

H -4.947810 -3.322750 -2.100386

C -0.343589 0.150378 1.745355

C -1.530918 0.709117 1.258949

C -1.669139 2.098201 1.269822

C -0.640616 2.910332 1.744124

C 0.540033 2.344923 2.218598

C 0.682734 0.956484 2.227911

C -2.577341 -0.152737 0.641164

N -3.809839 0.376869 0.509602

P -5.164573 -0.523643 0.464162

C -6.307433 0.406070 1.562592

C -7.277539 -0.305119 2.270403

C -6.242970 1.795023 1.699485

C -8.177905 0.362526 3.098413

C -7.143586 2.463998 2.522695

C -8.114578 1.748162 3.222140

C -5.939178 -0.236183 -1.174379

C -6.853544 -1.171961 -1.659998

C -5.655151 0.900177 -1.933050

C -7.472539 -0.978180 -2.893264

C -6.273734 1.096697 -3.164413

C -7.183845 0.157498 -3.646638

O -5.213060 -1.981513 0.820547

H -0.224944 -0.932538 1.727217

H -2.600537 2.516185 0.898163

H -0.758991 3.991138 1.737043

H 1.349402 2.977739 2.571435

H 1.602322 0.509078 2.596559

H -2.488040 -1.208429 0.932149

H -7.298520 -1.387378 2.171866

H -5.465682 2.338057 1.168865

H -8.927656 -0.197798 3.651091

H -7.085724 3.544454 2.626888

H -8.816195 2.270834 3.867212

H -7.055203 -2.059203 -1.064179

H -4.927979 1.613391 -1.553144

H -8.177961 -1.714357 -3.270365

H -6.040663 1.979162 -3.754670

H -7.663095 0.308423 -4.610388

**M-ent-3b-SS-TS1**

Zero-point correction= 0.689103 (Hartree/Particle)

Thermal correction to Energy= 0.733124

Thermal correction to Enthalpy= 0.734068

Thermal correction to Gibbs Free Energy= 0.606327

E(solv) = -5075.89608106 A.U.

C -5.612040 4.261823 -2.186316

C -6.404756 3.308667 -1.507617

C -5.839051 2.170078 -0.987039

C -4.447224 1.915047 -1.112308

C -3.657636 2.861880 -1.828703

C -4.269354 4.034442 -2.344594

C -3.820214 0.741601 -0.570036

C -2.486182 0.509726 -0.844490

C -1.685168 1.448380 -1.583379

C -2.281221 2.605853 -2.029912

C -4.598678 -0.226147 0.255324

C -5.245916 0.200820 1.461831

C -6.108472 -0.691236 2.158089

C -6.284196 -2.008642 1.666498

C -5.593552 -2.422495 0.565540

C -4.720115 -1.549717 -0.144965

C -5.049623 1.498579 2.011333

C -5.690050 1.886061 3.160782

C -6.568515 1.003198 3.832159

C -6.766453 -0.259607 3.339339

O -1.921588 -0.645553 -0.422659

C -0.272158 1.201117 -1.823673

O -4.070188 -2.057959 -1.210395

Br -5.787876 -4.198797 -0.050450

N 0.318222 0.137007 -1.374232

C 1.670946 -0.049903 -1.502395

C 2.180717 -1.413104 -1.350063

C 1.539999 -2.368747 -0.540842

C 2.123617 -3.610772 -0.382875

C 3.333864 -3.879827 -1.023013

C 3.898337 -2.870136 -1.788696

N 3.347862 -1.665931 -1.962367

H -6.068670 5.161076 -2.588881

H -7.472795 3.474104 -1.400678

H -6.463431 1.443111 -0.479049

H -3.644701 4.746584 -2.878639

H -1.676608 3.338735 -2.561062

H -6.946515 -2.695135 2.184628

H -4.370110 2.180354 1.511736

H -5.515247 2.879136 3.563665

H -7.071286 1.323949 4.739492

H -7.424343 -0.959364 3.848960

H -0.934036 -0.600985 -0.686525

H 0.280542 1.960286 -2.389241

H -3.156396 -1.700179 -1.189875

H 2.188464 0.536525 -2.264780

H 0.615472 -2.117288 -0.031130

H 1.651751 -4.360256 0.246394

H 3.834224 -4.836487 -0.916158

H 4.856809 -3.023169 -2.285649

C 0.805995 2.547665 0.426248

C 2.046463 2.223595 -0.129081

C 2.683340 3.165571 -0.949078

C 2.083806 4.388454 -1.215787

C 0.840730 4.703290 -0.656530

C 0.209264 3.783119 0.171911

C 2.649484 0.888004 0.090764

N 3.965196 0.748832 -0.093377

P 4.784631 -0.398777 0.735380

C 6.050070 -1.024255 -0.422866

C 6.759734 -2.170411 -0.058932

C 6.287437 -0.430104 -1.662587

C 7.707013 -2.715330 -0.921667

C 7.233117 -0.975863 -2.527197

C 7.944685 -2.116722 -2.157727

C 5.801568 0.539229 1.944402

C 6.075657 -0.044120 3.182551

C 6.307048 1.810000 1.657744

C 6.855264 0.629261 4.121079

C 7.087419 2.482728 2.593380

C 7.364939 1.891197 3.825444

O 4.091063 -1.519657 1.452026

H 0.299281 1.818278 1.054245

H 3.651911 2.903826 -1.365027

H 2.584906 5.107242 -1.858867

H 0.373399 5.661449 -0.866065

H -0.762431 4.009528 0.601927

H 2.136737 0.281540 0.848819

H 6.537282 -2.647089 0.892661

H 5.689935 0.430084 -1.948479

H 8.251134 -3.612042 -0.637561

H 7.407156 -0.519507 -3.497855

H 8.677969 -2.543794 -2.836790

H 5.651137 -1.021158 3.398002

H 6.063873 2.269226 0.703039

H 7.061163 0.172670 5.085591

H 7.474917 3.472471 2.367130

H 7.971804 2.418346 4.556840

**M-ent-3b-SS-TS2**

Zero-point correction= 0.688906 (Hartree/Particle)

Thermal correction to Energy= 0.732684

Thermal correction to Enthalpy= 0.733628

Thermal correction to Gibbs Free Energy= 0.610656

E(solv) = -5075.89903046 A.U.

C -3.345585 -3.227975 3.655940

C -4.333735 -2.424852 3.041626

C -4.068668 -1.775325 1.858182

C -2.802132 -1.885007 1.225276

C -1.796363 -2.666947 1.865614

C -2.107031 -3.340606 3.074793

C -2.488141 -1.225667 -0.006362

C -1.206542 -1.298486 -0.522145

C -0.155935 -2.004565 0.172301

C -0.493244 -2.693049 1.319437

C -3.517774 -0.447477 -0.746374

C -4.677705 -1.093964 -1.279401

C -5.709552 -0.325474 -1.887738

C -5.553052 1.078588 -1.981289

C -4.413629 1.669502 -1.517990

C -3.360734 0.923484 -0.908214

C -4.846209 -2.506543 -1.224105

C -5.972983 -3.106040 -1.726336

C -7.003015 -2.333308 -2.314461

C -6.867081 -0.972265 -2.394131

O -0.954147 -0.677809 -1.682155

C 1.220153 -1.999341 -0.293060

O -2.308737 1.627846 -0.468095

Br -4.214998 3.545463 -1.676676

N 1.518766 -1.486720 -1.463427

C 2.721448 -1.480141 -2.060444

C 3.908467 -2.200750 -1.701545

C 4.977611 -2.263909 -2.638723

C 6.145708 -2.910439 -2.309283

C 6.261316 -3.517343 -1.050242

C 5.173271 -3.411938 -0.196895

N 4.034574 -2.776818 -0.481181

H -3.565599 -3.743623 4.586627

H -5.310055 -2.319694 3.506517

H -4.833392 -1.158320 1.396551

H -1.324198 -3.930799 3.546953

H 0.284964 -3.240646 1.848480

H -6.333002 1.681360 -2.436106

H -4.057596 -3.106202 -0.782478

H -6.074123 -4.186317 -1.676916

H -7.890933 -2.820745 -2.706405

H -7.641864 -0.361119 -2.851472

H 0.035155 -0.771021 -1.844620

H 1.962051 -2.506992 0.318609

H -1.438784 1.173228 -0.569451

H 2.725430 -1.043497 -3.058057

H 4.858588 -1.785188 -3.606356

H 6.969412 -2.945854 -3.018396

H 7.160270 -4.042858 -0.746222

H 5.217011 -3.864288 0.794615

C 5.225303 1.025750 -2.384899

C 4.751485 0.600412 -1.140651

C 5.649742 0.056416 -0.215275

C 6.995463 -0.060625 -0.536883

C 7.466203 0.378806 -1.774705

C 6.576603 0.921127 -2.699458

C 3.327110 0.758233 -0.808967

N 2.897558 0.619515 0.404246

P 1.397023 1.263464 0.766110

C 0.790196 0.353561 2.196811

C -0.570119 0.465245 2.502951

C 1.636468 -0.407119 3.004157

C -1.077431 -0.169203 3.632819

C 1.120251 -1.044506 4.130067

C -0.230618 -0.917361 4.447946

C 1.847566 2.914935 1.428672

C 2.592468 3.777541 0.616925

C 1.429602 3.360391 2.684044

C 2.908772 5.059983 1.048357

C 1.748864 4.645938 3.118821

C 2.487380 5.496847 2.303613

O 0.388253 1.396077 -0.337473

H 4.522481 1.435439 -3.107730

H 5.262057 -0.279878 0.740725

H 7.682826 -0.503792 0.178028

H 8.520441 0.288548 -2.021010

H 6.934952 1.260360 -3.667435

H 2.686759 1.159013 -1.604183

H -1.230144 1.018506 1.837206

H 2.681939 -0.509124 2.728175

H -2.141070 -0.121466 3.850109

H 1.772075 -1.647375 4.756269

H -0.636454 -1.430708 5.315300

H 2.931280 3.440504 -0.359678

H 0.852239 2.702281 3.326732

H 3.487932 5.718683 0.407855

H 1.418001 4.980427 4.097838

H 2.736649 6.498037 2.643594

**M-ent-3b-SS-TS3**

Zero-point correction= 0.689131 (Hartree/Particle)

Thermal correction to Energy= 0.733046

Thermal correction to Enthalpy= 0.733991

Thermal correction to Gibbs Free Energy= 0.609966

E(solv) = -5075.89485791 A.U.

C -3.905170 5.633344 -0.620479

C -4.729021 4.522285 -0.329223

C -4.236883 3.242988 -0.424745

C -2.895883 2.999700 -0.821888

C -2.068440 4.121662 -1.119153

C -2.604690 5.430303 -1.007216

C -2.355402 1.679087 -0.942309

C -1.078780 1.514697 -1.433619

C -0.202607 2.635116 -1.634534

C -0.719651 3.901659 -1.487669

C -3.107075 0.495431 -0.445458

C -3.416630 0.404775 0.952035

C -4.220430 -0.666494 1.431275

C -4.643032 -1.668794 0.523143

C -4.240655 -1.619301 -0.780374

C -3.450993 -0.549167 -1.287724

C -2.893306 1.329028 1.899514

C -3.209427 1.220923 3.230712

C -4.057794 0.186417 3.694187

C -4.543246 -0.742282 2.811655

O -0.651916 0.269903 -1.723112

C 1.215716 2.429507 -1.876103

O -3.074691 -0.603899 -2.583666

Br -4.702108 -3.034307 -1.947678

N 1.742225 1.248736 -1.934162

C 3.100268 1.074325 -1.915982

C 3.662217 -0.202084 -2.312392

C 2.943730 -1.404310 -2.157318

C 3.575044 -2.604965 -2.431350

C 4.903569 -2.595359 -2.858206

C 5.530067 -1.358506 -2.981331

N 4.948392 -0.190317 -2.720809

H -4.303093 6.640610 -0.539234

H -5.760998 4.680868 -0.029694

H -4.879117 2.395467 -0.206562

H -1.956443 6.273564 -1.234956

H -0.067718 4.761409 -1.632740

H -5.245733 -2.500068 0.875361

H -2.193051 2.083966 1.560378

H -2.781464 1.922896 3.940414

H -4.299503 0.116786 4.750779

H -5.166832 -1.564637 3.153793

H 0.352379 0.315247 -1.808270

H 1.845761 3.327019 -1.937801

H -2.142255 -0.304084 -2.623437

H 3.727354 1.943754 -2.131216

H 1.915679 -1.381837 -1.814108

H 3.033898 -3.539247 -2.303041

H 5.437555 -3.512023 -3.086676

H 6.568750 -1.303222 -3.307771

C 5.401703 2.394185 0.099388

C 4.817669 1.130572 0.221624

C 5.644776 0.010061 0.339118

C 7.027621 0.154305 0.333879

C 7.602950 1.418376 0.219313

C 6.784188 2.540424 0.102408

C 3.340436 0.975123 0.210013

N 2.836784 -0.138370 0.726316

P 1.277820 -0.239704 1.230863

C 0.717546 -1.866863 0.617533

C -0.623691 -2.034207 0.271418

C 1.606644 -2.937514 0.478125

C -1.072938 -3.252607 -0.237779

C 1.154672 -4.160115 -0.008565

C -0.183274 -4.314569 -0.374438

C 1.390818 -0.501904 3.039140

C 0.218944 -0.363642 3.789076

C 2.585738 -0.831382 3.680382

C 0.240935 -0.561031 5.166145

C 2.607428 -1.024880 5.060563

C 1.436019 -0.893408 5.803107

O 0.273546 0.842388 0.950490

H 4.758287 3.266837 0.002996

H 5.174566 -0.965950 0.421032

H 7.661515 -0.724639 0.415077

H 8.683758 1.529641 0.217845

H 7.224804 3.529699 0.013881

H 2.771050 1.914929 0.255688

H -1.297329 -1.188684 0.369959

H 2.656148 -2.788174 0.718415

H -2.106565 -3.361629 -0.555270

H 1.848776 -4.989438 -0.119012

H -0.530176 -5.263425 -0.774779

H -0.703182 -0.085854 3.282982

H 3.492714 -0.914719 3.087205

H -0.673945 -0.451440 5.742691

H 3.540519 -1.275079 5.558195

H 1.454965 -1.045182 6.879143

**M-ent-3b-SS-TS4**

Zero-point correction= 0.689317 (Hartree/Particle)

Thermal correction to Energy= 0.733040

Thermal correction to Enthalpy= 0.733984

Thermal correction to Gibbs Free Energy= 0.610882

E(solv) = -5075.89856085 A.U.

C -3.346900 -3.651199 3.275459

C -4.321958 -2.775505 2.745244

C -4.046599 -2.013448 1.633490

C -2.781527 -2.079110 0.991931

C -1.791805 -2.942587 1.545228

C -2.110425 -3.726190 2.683737

C -2.457985 -1.305447 -0.168699

C -1.188311 -1.367739 -0.713300

C -0.148470 -2.148543 -0.086679

C -0.492112 -2.936532 0.991967

C -3.476965 -0.422533 -0.799624

C -4.629216 -0.978083 -1.437393

C -5.652653 -0.124313 -1.936328

C -5.497485 1.277365 -1.807594

C -4.369424 1.788546 -1.234561

C -3.324788 0.956119 -0.732734

C -4.800639 -2.382961 -1.588454

C -5.920876 -2.898310 -2.188883

C -6.939808 -2.043649 -2.674299

C -6.802335 -0.685962 -2.550424

O -0.941551 -0.680536 -1.835638

C 1.234142 -2.102632 -0.525457

O -2.281957 1.578496 -0.166582

Br -4.174617 3.666458 -1.085209

N 1.607764 -1.429608 -1.583119

C 2.856597 -1.309894 -2.067739

C 4.081589 -1.938608 -1.626012

C 4.355252 -2.346551 -0.297916

C 5.601663 -2.870228 0.012564

C 6.575004 -2.968492 -0.976424

C 6.239543 -2.486194 -2.242667

N 5.054269 -1.997023 -2.574187

H -3.575268 -4.252695 4.150840

H -5.296870 -2.703169 3.219112

H -4.801416 -1.341949 1.236049

H -1.337657 -4.374158 3.092219

H 0.280878 -3.535774 1.471197

H -6.271347 1.943877 -2.175388

H -4.019774 -3.042409 -1.224546

H -6.026117 -3.973850 -2.297400

H -7.821946 -2.465500 -3.146948

H -7.570608 -0.012374 -2.923146

H 0.046866 -0.690311 -1.968813

H 1.910885 -2.751562 0.030234

H -1.404458 1.135751 -0.304449

H 2.922828 -0.886309 -3.066572

H 3.626922 -2.182148 0.484881

H 5.815896 -3.182096 1.032304

H 7.561431 -3.373948 -0.777441

H 6.983585 -2.495672 -3.040262

C 4.998456 1.257664 -2.600963

C 4.665991 0.899015 -1.291389

C 5.688643 0.580145 -0.391184

C 7.015562 0.602354 -0.803339

C 7.339679 0.964232 -2.110165

C 6.326234 1.296455 -3.007576

C 3.255351 0.847132 -0.879217

N 2.923435 0.636465 0.367129

P 1.383954 1.072257 0.814623

C 0.929125 -0.004168 2.192125

C -0.412697 -0.004403 2.584248

C 1.856003 -0.806875 2.857608

C -0.824348 -0.800480 3.647848

C 1.437525 -1.607124 3.918618

C 0.101190 -1.599940 4.315145

C 1.575003 2.721908 1.571283

C 0.500369 3.611131 1.483879

C 2.742393 3.102650 2.237574

C 0.593796 4.871283 2.069940

C 2.832913 4.363777 2.819017

C 1.757192 5.246676 2.737895

O 0.309262 1.153728 -0.234290

H 4.201751 1.487068 -3.304834

H 5.415761 0.304392 0.622249

H 7.801695 0.331959 -0.103829

H 8.377847 0.981323 -2.429962

H 6.571174 1.572560 -4.029016

H 2.525771 1.217937 -1.606649

H -1.139417 0.585427 2.028115

H 2.891263 -0.805809 2.526969

H -1.874807 -0.834174 3.924010

H 2.154263 -2.240903 4.433696

H -0.227671 -2.237312 5.131468

H -0.395731 3.311375 0.944780

H 3.579722 2.411050 2.280081

H -0.241140 5.562308 1.999533

H 3.743529 4.661053 3.331394

H 1.828839 6.231329 3.191742

**M-ent-3b-RS-TS1**

Zero-point correction= 0.690258 (Hartree/Particle)

Thermal correction to Energy= 0.734099

Thermal correction to Enthalpy= 0.735043

Thermal correction to Gibbs Free Energy= 0.608021

E(solv) = -5075.89843605 A.U.

C -4.275608 -5.526750 0.771576

C -5.290311 -4.551250 0.899724

C -5.041262 -3.236134 0.590943

C -3.764348 -2.816768 0.130906

C -2.732842 -3.797488 0.038889

C -3.024478 -5.150096 0.356548

C -3.468555 -1.454924 -0.210345

C -2.174853 -1.115708 -0.558904

C -1.116922 -2.089142 -0.613033

C -1.427856 -3.400877 -0.334250

C -4.507184 -0.388919 -0.121446

C -5.709128 -0.444704 -0.898655

C -6.728760 0.528042 -0.698578

C -6.526746 1.560019 0.251619

C -5.345293 1.637843 0.929935

C -4.309573 0.681109 0.739434

C -5.923536 -1.440481 -1.891697

C -7.085506 -1.475901 -2.619815

C -8.104467 -0.518754 -2.401254

C -7.923658 0.462030 -1.461796

O -1.887294 0.183320 -0.801748

C 0.249640 -1.686491 -0.916057

O -3.167605 0.851621 1.438438

Br -5.046733 3.041393 2.158610

N 0.524539 -0.466156 -1.262794

C 1.823379 -0.053308 -1.411387

C 2.090821 1.200384 -2.100074

C 1.113641 2.199115 -2.284820

C 1.484477 3.404945 -2.850889

C 2.816886 3.607661 -3.216409

C 3.709550 2.565987 -2.989930

N 3.373717 1.393409 -2.456488

H -4.485235 -6.564279 1.014427

H -6.275489 -4.842478 1.252366

H -5.827039 -2.498091 0.709624

H -2.224463 -5.881618 0.270853

H -0.639919 -4.150569 -0.374784

H -7.301027 2.302636 0.416402

H -5.144035 -2.172847 -2.072814

H -7.221922 -2.242197 -3.377029

H -9.019745 -0.558019 -2.984236

H -8.689421 1.214475 -1.289404

H -0.892798 0.220871 -1.050941

H 1.043957 -2.436671 -0.829603

H -2.426677 0.724808 0.803873

H 2.573000 -0.832290 -1.562598

H 0.091481 2.024191 -1.964503

H 0.744710 4.187972 -2.995224

H 3.152175 4.539783 -3.659545

H 4.762003 2.672103 -3.257731

C 0.296629 1.441753 1.275362

C 1.624776 1.531310 0.843180

C 2.196870 2.795484 0.671969

C 1.455044 3.944735 0.919304

C 0.131346 3.846629 1.343307

C -0.449744 2.592600 1.524906

C 2.415703 0.312808 0.515350

N 3.746562 0.416469 0.564921

P 4.616085 -0.965652 0.413940

C 5.795056 -0.668772 -0.949515

C 6.321261 -1.772177 -1.620969

C 6.192814 0.618423 -1.317128

C 7.248803 -1.594557 -2.645625

C 7.122898 0.795843 -2.336419

C 7.653286 -0.310391 -2.999889

C 5.699591 -0.990351 1.888895

C 6.146325 -2.223045 2.367846

C 6.091383 0.184211 2.535543

C 6.988072 -2.282487 3.476061

C 6.933753 0.124747 3.642148

C 7.385502 -1.108080 4.110973

O 3.934486 -2.295735 0.242979

H -0.144584 0.457185 1.426466

H 3.225439 2.846021 0.325780

H 1.908548 4.921491 0.772051

H -0.449326 4.744456 1.536746

H -1.480841 2.518078 1.860543

H 1.926609 -0.634331 0.792626

H 5.978597 -2.765432 -1.342094

H 5.738742 1.469392 -0.817303

H 7.651858 -2.456577 -3.170502

H 7.430090 1.798664 -2.622273

H 8.377239 -0.169230 -3.798146

H 5.807558 -3.128164 1.870187

H 5.709811 1.135076 2.173747

H 7.330201 -3.244197 3.848898

H 7.232981 1.039710 4.146346

H 8.040207 -1.152298 4.977308

**M-ent-3b-RS-TS2**

Zero-point correction= 0.689032 (Hartree/Particle)

Thermal correction to Energy= 0.732750

Thermal correction to Enthalpy= 0.733694

Thermal correction to Gibbs Free Energy= 0.609765

E(solv) = -5075.89817522 A.U.

C 5.669815 -3.770093 -1.556399

C 5.999729 -2.402699 -1.411747

C 5.130598 -1.533465 -0.798845

C 3.880875 -1.977346 -0.286852

C 3.536079 -3.350374 -0.468522

C 4.459926 -4.224869 -1.099348

C 2.950455 -1.103914 0.363836

C 1.722947 -1.595636 0.768641

C 1.334339 -2.957783 0.510902

C 2.255147 -3.799095 -0.072502

C 3.222304 0.348682 0.537618

C 4.346068 0.809679 1.296676

C 4.648020 2.199165 1.344385

C 3.801512 3.112737 0.669548

C 2.691566 2.662191 0.014741

C 2.369123 1.274366 -0.050062

C 5.181600 -0.079138 2.029080

C 6.261541 0.383506 2.737224

C 6.572657 1.763894 2.757964

C 5.776945 2.648546 2.078365

O 0.861676 -0.743643 1.350211

C -0.053000 -3.365233 0.671076

O 1.259593 0.932512 -0.740457

Br 1.524806 3.879334 -0.829178

N -0.923669 -2.586138 1.234101

C -2.261256 -2.744246 1.064493

C -3.184539 -2.123048 1.997826

C -2.777070 -1.146581 2.926993

C -3.716010 -0.615576 3.794779

C -5.040311 -1.046196 3.718150

C -5.356774 -1.977605 2.731911

N -4.474919 -2.512216 1.892587

H 6.366166 -4.449467 -2.039359

H 6.946480 -2.033479 -1.795452

H 5.392449 -0.484833 -0.708322

H 4.177835 -5.268437 -1.220454

H 1.966587 -4.825549 -0.292824

H 4.022251 4.175245 0.699123

H 4.943618 -1.137607 2.026281

H 6.879865 -0.315334 3.292949

H 7.431711 2.118002 3.320225

H 5.990017 3.714792 2.095369

H -0.024733 -1.219328 1.442718

H -0.358895 -4.306407 0.196468

H 0.676365 0.364013 -0.187243

H -2.629822 -3.653983 0.585735

H -1.746805 -0.813354 2.949890

H -3.415654 0.141660 4.513881

H -5.808294 -0.658843 4.380187

H -6.386213 -2.316158 2.612935

C -2.594199 -3.376445 -2.049694

C -1.809167 -2.307757 -1.593591

C -0.478993 -2.218729 -2.013973

C 0.060067 -3.185344 -2.863369

C -0.725443 -4.243158 -3.306820

C -2.061050 -4.333557 -2.900617

C -2.366369 -1.323850 -0.643826

N -1.669292 -0.223221 -0.391288

P -2.331422 1.151933 0.235477

C -1.830416 2.439959 -0.955678

C -2.030439 3.775522 -0.596619

C -1.324560 2.136319 -2.220642

C -1.750393 4.796528 -1.499837

C -1.040328 3.157834 -3.124127

C -1.259787 4.486498 -2.768122

C -4.148199 1.075723 -0.014945

C -4.974464 1.134127 1.105153

C -4.721030 0.935539 -1.283903

C -6.358397 1.038097 0.965582

C -6.101431 0.847571 -1.426078

C -6.922062 0.895472 -0.298244

O -2.019476 1.558513 1.642519

H -3.625208 -3.453072 -1.709127

H 0.123704 -1.380060 -1.675489

H 1.101804 -3.111185 -3.162820

H -0.305536 -4.997887 -3.966055

H -2.681111 -5.155273 -3.248254

H -3.459175 -1.343077 -0.551395

H -2.393230 4.002445 0.402547

H -1.134460 1.097065 -2.474984

H -1.905295 5.833417 -1.214356

H -0.633181 2.917297 -4.101984

H -1.036079 5.282363 -3.473666

H -4.513073 1.249990 2.080621

H -4.078631 0.896405 -2.161852

H -6.992784 1.067565 1.847534

H -6.540063 0.738274 -2.413998

H -8.000416 0.819757 -0.409235

**M-ent-3b-RS-TS3**

Zero-point correction= 0.689185 (Hartree/Particle)

Thermal correction to Energy= 0.733102

Thermal correction to Enthalpy= 0.734046

Thermal correction to Gibbs Free Energy= 0.607085

E(solv) = -5075.89667730 A.U.

C 4.941946 5.266597 0.098678

C 5.822009 4.194552 0.369600

C 5.406063 2.892996 0.225123

C 4.085817 2.583080 -0.198721

C 3.193364 3.667387 -0.441787

C 3.654984 5.001093 -0.293414

C 3.617624 1.235912 -0.363502

C 2.282341 1.010014 -0.653462

C 1.375984 2.103889 -0.911632

C 1.857910 3.389339 -0.814505

C 4.555626 0.087070 -0.206576

C 5.714394 -0.028695 -1.043695

C 6.676235 -1.046698 -0.789027

C 6.453254 -1.961600 0.269954

C 5.305581 -1.884736 1.002468

C 4.328086 -0.876253 0.765443

C 5.945464 0.841062 -2.146053

C 7.069536 0.718888 -2.922663

C 8.034734 -0.278260 -2.646640

C 7.834849 -1.143191 -1.603299

O 1.839985 -0.260770 -0.687410

C -0.011883 1.854244 -1.272635

O 3.228589 -0.890275 1.541264

Br 4.981918 -3.140937 2.379219

N -0.453777 0.642447 -1.360177

C -1.755894 0.319029 -1.575581

C -2.076229 -1.065187 -1.861522

C -3.377686 -1.387651 -2.293064

C -3.742749 -2.718697 -2.403952

C -2.796272 -3.702494 -2.119667

C -1.521271 -3.285469 -1.739531

N -1.154662 -2.014771 -1.599404

H 5.282093 6.291697 0.212132

H 6.835606 4.400097 0.701396

H 6.090182 2.081638 0.449597

H 2.957084 5.811399 -0.491582

H 1.184402 4.221312 -1.013444

H 7.183076 -2.737877 0.477062

H 5.207185 1.602215 -2.373198

H 7.217088 1.391196 -3.762599

H 8.921837 -0.361615 -3.267325

H 8.556253 -1.926572 -1.383559

H 0.850610 -0.250318 -0.945508

H -0.656659 2.722456 -1.461783

H 2.446046 -0.710285 0.972182

H -2.431101 1.075473 -1.983339

H -4.069897 -0.585825 -2.532905

H -4.755466 -2.981277 -2.702206

H -3.030310 -4.759438 -2.194979

H -0.748317 -4.023311 -1.524861

C -0.507967 0.639877 1.713187

C -1.577680 -0.100857 1.198948

C -1.625011 -1.479912 1.430497

C -0.605473 -2.107148 2.135888

C 0.469485 -1.367666 2.629319

C 0.509565 0.011352 2.424743

C -2.627264 0.548792 0.383804

N -3.839902 0.004327 0.389434

P -5.115381 0.735880 -0.327508

C -6.214616 -0.669524 -0.712668

C -7.084820 -0.551022 -1.797221

C -6.174918 -1.862001 0.014531

C -7.917155 -1.612222 -2.148814

C -7.009648 -2.919501 -0.333595

C -7.882773 -2.795265 -1.414315

C -5.993194 1.645719 0.998805

C -6.758487 2.761439 0.653609

C -5.914254 1.250691 2.336623

C -7.448317 3.469963 1.634410

C -6.604345 1.958542 3.317025

C -7.374252 3.066237 2.965859

O -4.961135 1.628105 -1.528897

H -0.473933 1.713712 1.548539

H -2.461497 -2.040773 1.023069

H -0.637564 -3.182491 2.287781

H 1.287768 -1.859665 3.146877

H 1.349668 0.589678 2.799207

H -2.475110 1.627782 0.230438

H -7.076474 0.370197 -2.374146

H -5.458699 -1.957818 0.825465

H -8.586698 -1.519348 -2.999456

H -6.970188 -3.848510 0.228250

H -8.529001 -3.624632 -1.688828

H -6.785085 3.071007 -0.387758

H -5.291705 0.399276 2.599055

H -8.038448 4.340926 1.362744

H -6.536398 1.651691 4.356982

H -7.910061 3.619783 3.732213

**M-ent-3b-RS-TS4**

Zero-point correction= 0.688406 (Hartree/Particle)

Thermal correction to Energy= 0.732074

Thermal correction to Enthalpy= 0.733018

Thermal correction to Gibbs Free Energy= 0.609746

E(solv) = -5075.89169006 A.U.

C 5.777331 -3.524618 -1.654431

C 6.032561 -2.141021 -1.512159

C 5.136623 -1.325461 -0.865336

C 3.932066 -1.841867 -0.312861

C 3.661731 -3.231803 -0.492430

C 4.611282 -4.049350 -1.159627

C 2.977765 -1.026706 0.376036

C 1.792515 -1.585548 0.825553

C 1.473706 -2.966787 0.556962

C 2.419070 -3.751517 -0.061808

C 3.169663 0.440190 0.537264

C 4.289779 0.973444 1.252637

C 4.510077 2.378903 1.280601

C 3.590448 3.235185 0.626493

C 2.487603 2.714038 0.013089

C 2.243329 1.309416 -0.025281

C 5.201800 0.141552 1.960190

C 6.275596 0.672908 2.628262

C 6.503866 2.069621 2.630550

C 5.634525 2.900019 1.972994

O 0.921621 -0.782837 1.451263

C 0.106569 -3.440561 0.733735

O 1.131678 0.895254 -0.671454

Br 1.229826 3.850368 -0.813589

N -0.761075 -2.679139 1.308614

C -2.108363 -2.806763 1.211092

C -2.919928 -2.080739 2.174721

C -4.294723 -2.362780 2.317793

C -5.041077 -1.629680 3.222016

C -4.419681 -0.609570 3.945238

C -3.070122 -0.371031 3.698448

N -2.327082 -1.087537 2.858649

H 6.494577 -4.160727 -2.164954

H 6.943056 -1.716195 -1.925130

H 5.341572 -0.264082 -0.777676

H 4.384559 -5.106729 -1.278103

H 2.176738 -4.788752 -0.288495

H 3.750929 4.308807 0.637895

H 5.026942 -0.929176 1.971194

H 6.953183 0.016450 3.166360

H 7.358553 2.478742 3.161349

H 5.784259 3.977155 1.976303

H 0.060330 -1.296636 1.612425

H -0.171513 -4.390918 0.257260

H 0.579253 0.329461 -0.082540

H -2.525460 -3.732629 0.801910

H -4.744851 -3.156684 1.727287

H -6.099234 -1.839847 3.357310

H -4.967177 -0.002384 4.658871

H -2.553454 0.449055 4.191714

C -2.441191 -3.503607 -1.903072

C -1.710614 -2.394634 -1.455877

C -0.397131 -2.228974 -1.903839

C 0.178734 -3.160666 -2.767389

C -0.553101 -4.261160 -3.199891

C -1.872502 -4.427837 -2.768581

C -2.309083 -1.442522 -0.493205

N -1.684850 -0.294574 -0.260909

P -2.445116 1.093125 0.208025

C -1.993972 2.274875 -1.109764

C -2.271217 3.627547 -0.893671

C -1.415868 1.876560 -2.315462

C -1.990269 4.570009 -1.878293

C -1.127820 2.819903 -3.299425

C -1.419171 4.164602 -3.084641

C -4.251119 0.872785 -0.060649

C -5.097787 1.038563 1.033694

C -4.797569 0.544786 -1.306283

C -6.473827 0.862707 0.893515

C -6.170659 0.374878 -1.450291

C -7.010633 0.531216 -0.346830

O -2.215391 1.702511 1.554130

H -3.463691 -3.636668 -1.553337

H 0.166117 -1.360697 -1.571613

H 1.208779 -3.026395 -3.085698

H -0.104046 -4.988213 -3.870837

H -2.453483 -5.280299 -3.109934

H -3.401326 -1.517789 -0.414011

H -2.687035 3.931047 0.063529

H -1.166141 0.828698 -2.458431

H -2.203823 5.620975 -1.702810

H -0.660396 2.506715 -4.228583

H -1.189171 4.899949 -3.851200

H -4.655710 1.300058 1.990649

H -4.140028 0.424945 -2.165813

H -7.125398 0.984042 1.755079

H -6.588333 0.121042 -2.420683

H -8.083234 0.396198 -0.458725

**M-ent-3b-SR-TS1**

Zero-point correction= 0.688910 (Hartree/Particle)

Thermal correction to Energy= 0.732766

Thermal correction to Enthalpy= 0.733710

Thermal correction to Gibbs Free Energy= 0.608721

E(solv) = -5075.89925257 A.U.

C -5.721166 4.143236 -0.761245

C -6.127795 2.817383 -0.488229

C -5.203546 1.804091 -0.409877

C -3.818070 2.053870 -0.599172

C -3.416217 3.385902 -0.910009

C -4.393587 4.413560 -0.971730

C -2.823079 1.022271 -0.507083

C -1.518159 1.321015 -0.852079

C -1.105307 2.656694 -1.193733

C -2.052744 3.654081 -1.175778

C -3.183249 -0.352151 -0.053793

C -3.796361 -0.555488 1.228469

C -4.253920 -1.850776 1.600659

C -4.044042 -2.939848 0.718821

C -3.381974 -2.742105 -0.456762

C -2.927383 -1.453098 -0.860975

C -3.952640 0.497572 2.173047

C -4.553988 0.279456 3.386391

C -5.038043 -1.003501 3.734267

C -4.882819 -2.044950 2.858299

O -0.606306 0.323681 -0.848376

C 0.288488 2.950144 -1.496701

O -2.276277 -1.372032 -2.037595

Br -3.001397 -4.215710 -1.578067

N 1.146447 2.000270 -1.686753

C 2.488493 2.223866 -1.786682

C 3.313968 1.216207 -2.443843

C 2.833958 -0.067191 -2.745452

C 3.708409 -0.998197 -3.286230

C 5.032057 -0.637491 -3.506498

C 5.413609 0.659179 -3.170069

N 4.594684 1.573578 -2.664224

H -6.459330 4.937899 -0.815515

H -7.181126 2.595583 -0.343943

H -5.531591 0.789587 -0.209902

H -4.062614 5.423763 -1.201154

H -1.749152 4.675116 -1.399083

H -4.375720 -3.934885 0.997949

H -3.573587 1.482885 1.925946

H -4.651422 1.099498 4.091449

H -5.514637 -1.161358 4.696985

H -5.227276 -3.043960 3.114035

H 0.293665 0.723125 -1.114152

H 0.582508 4.005831 -1.552951

H -1.497340 -0.788588 -1.896376

H 2.833639 3.253771 -1.907717

H 1.795839 -0.318618 -2.556264

H 3.356039 -1.999815 -3.517505

H 5.754867 -1.336919 -3.913040

H 6.443969 0.979735 -3.321641

C 2.945235 4.320050 0.605769

C 2.370886 3.050220 0.745360

C 1.105734 2.945566 1.332003

C 0.432257 4.083506 1.774242

C 1.013511 5.339607 1.635031

C 2.277327 5.454161 1.049450

C 3.049436 1.847289 0.207391

N 2.608644 0.650189 0.591694

P 3.581693 -0.669157 0.552632

C 3.825901 -1.101409 2.319798

C 4.990635 -1.781320 2.681842

C 2.888206 -0.775285 3.302855

C 5.211599 -2.142559 4.008864

C 3.107345 -1.137584 4.629189

C 4.267747 -1.824490 4.982915

C 2.491177 -2.001807 -0.052851

C 3.050244 -3.047480 -0.789493

C 1.112625 -1.975606 0.170251

C 2.235284 -4.048398 -1.315962

C 0.298593 -2.972426 -0.360097

C 0.857439 -4.007735 -1.106600

O 4.908760 -0.677417 -0.146792

H 3.922711 4.406234 0.135092

H 0.670271 1.954561 1.430733

H -0.556561 3.985476 2.214168

H 0.488817 6.227272 1.977303

H 2.739759 6.431535 0.941068

H 4.104466 1.992103 -0.061043

H 5.722285 -2.001709 1.909101

H 1.999923 -0.217649 3.018142

H 6.122050 -2.667667 4.285569

H 2.375923 -0.878998 5.390043

H 4.438851 -2.104927 6.018902

H 4.121904 -3.036930 -0.974336

H 0.675132 -1.148822 0.721264

H 2.672816 -4.853150 -1.901435

H -0.773830 -2.943663 -0.195888

H 0.207938 -4.769403 -1.529679

**M-ent-3b-SR-TS2**

Zero-point correction= 0.689237 (Hartree/Particle)

Thermal correction to Energy= 0.733065

Thermal correction to Enthalpy= 0.734009

Thermal correction to Gibbs Free Energy= 0.608008

E(solv) = -5075.89416443 A.U.

C -3.292372 5.579110 -0.846420

C -4.413690 4.806099 -0.468893

C -4.306820 3.444141 -0.321176

C -3.074746 2.772136 -0.540668

C -1.954134 3.551799 -0.953416

C -2.093671 4.958334 -1.086832

C -2.924295 1.352064 -0.387573

C -1.728421 0.755501 -0.745886

C -0.589949 1.537486 -1.165843

C -0.729132 2.906226 -1.236641

C -4.049662 0.521534 0.130714

C -4.610217 0.783197 1.424635

C -5.778623 0.089117 1.847029

C -6.350795 -0.885302 0.992087

C -5.751978 -1.182386 -0.196973

C -4.577546 -0.507270 -0.638392

C -4.029505 1.716060 2.329159

C -4.591312 1.957462 3.557041

C -5.769802 1.284878 3.958160

C -6.344569 0.367434 3.118657

O -1.644064 -0.590706 -0.699433

C 0.679491 0.899606 -1.466072

O -4.048792 -0.887682 -1.816335

Br -6.488408 -2.524833 -1.306345

N 0.785293 -0.393367 -1.393081

C 1.953631 -1.066280 -1.483590

C 1.924597 -2.516251 -1.467561

C 3.132638 -3.230781 -1.601753

C 3.108234 -4.606908 -1.446506

C 1.898016 -5.243402 -1.167831

C 0.759850 -4.448070 -1.061166

N 0.751390 -3.126015 -1.204135

H -3.385896 6.655621 -0.955758

H -5.370627 5.291044 -0.299314

H -5.179917 2.863645 -0.042954

H -1.222957 5.533010 -1.394519

H 0.131030 3.504900 -1.532732

H -7.247972 -1.414083 1.298527

H -3.120453 2.229939 2.036433

H -4.122268 2.667693 4.231404

H -6.206101 1.488065 4.931540

H -7.240853 -0.172748 3.414132

H -0.681062 -0.861460 -0.948841

H 1.529144 1.539952 -1.734904

H -3.071564 -0.904025 -1.699108

H 2.816559 -0.581365 -1.945145

H 4.053710 -2.686955 -1.799958

H 4.026872 -5.181236 -1.537461

H 1.834678 -6.318700 -1.036426

H -0.205852 -4.903073 -0.842201

C 0.530581 -1.125183 1.590362

C 1.839826 -1.532778 1.318822

C 2.255438 -2.810697 1.707239

C 1.365433 -3.670030 2.340310

C 0.055034 -3.263607 2.592424

C -0.361383 -1.987743 2.217594

C 2.743514 -0.664059 0.538167

N 4.045895 -0.906470 0.569496

P 5.098332 -0.077429 -0.378426

C 4.411410 1.588263 -0.736312

C 4.359707 2.004469 -2.068506

C 3.878012 2.412049 0.259863

C 3.783482 3.229105 -2.402141

C 3.290627 3.629385 -0.071922

C 3.244293 4.038810 -1.404806

C 6.516626 0.236692 0.718932

C 7.749031 0.549610 0.139895

C 6.398707 0.190357 2.109130

C 8.849763 0.830556 0.943668

C 7.501045 0.471426 2.913579

C 8.724468 0.795409 2.331641

O 5.540757 -0.691872 -1.675660

H 0.207253 -0.132178 1.286620

H 3.272064 -3.112594 1.475399

H 1.688611 -4.668204 2.623253

H -0.643083 -3.942685 3.074251

H -1.383689 -1.666210 2.393594

H 2.347751 0.342220 0.337122

H 4.767676 1.343544 -2.828680

H 3.911109 2.089004 1.298717

H 3.744537 3.546619 -3.440255

H 2.863194 4.256587 0.704813

H 2.784557 4.988812 -1.663756

H 7.833784 0.550659 -0.943642

H 5.443986 -0.091083 2.545110

H 9.807311 1.070760 0.490233

H 7.407562 0.429977 3.995107

H 9.583965 1.012794 2.959918

**M-ent-3b-SR-TS3**

Zero-point correction= 0.688225 (Hartree/Particle)

Thermal correction to Energy= 0.731995

Thermal correction to Enthalpy= 0.732940

Thermal correction to Gibbs Free Energy= 0.607624

E(solv) = -5075.89742200 A.U.

C -5.842901 4.019338 -0.493173

C -6.198370 2.685536 -0.187700

C -5.251894 1.690356 -0.190444

C -3.892536 1.965612 -0.500314

C -3.545284 3.306140 -0.840138

C -4.543465 4.314792 -0.815793

C -2.877952 0.950785 -0.500270

C -1.612878 1.267378 -0.967285

C -1.253272 2.621440 -1.309475

C -2.210949 3.601811 -1.208024

C -3.160529 -0.422934 0.007674

C -3.637864 -0.610899 1.349550

C -4.006471 -1.910336 1.798434

C -3.835406 -3.017557 0.931300

C -3.296923 -2.830635 -0.307007

C -2.942008 -1.537950 -0.791885

C -3.740421 0.461262 2.280270

C -4.214271 0.256985 3.551244

C -4.615760 -1.031068 3.976048

C -4.504803 -2.090600 3.115173

O -0.702627 0.283859 -1.080953

C 0.120658 2.938504 -1.686524

O -2.418529 -1.469653 -2.030617

Br -2.965285 -4.322776 -1.419158

N 0.958903 1.978858 -1.868338

C 2.308594 2.101496 -2.044482

C 2.967059 0.934004 -2.633284

C 4.271755 1.024242 -3.145256

C 4.899040 -0.124845 -3.589578

C 4.217326 -1.338910 -3.518742

C 2.930272 -1.330575 -2.995988

N 2.308204 -0.233083 -2.564414

H -6.598202 4.799543 -0.482302

H -7.230942 2.443582 0.046767

H -5.540671 0.669471 0.036334

H -4.252338 5.331543 -1.069596

H -1.939778 4.631882 -1.433032

H -4.096326 -4.015679 1.268473

H -3.421469 1.451568 1.975754

H -4.272263 1.092809 4.242057

H -4.991903 -1.178283 4.983935

H -4.784076 -3.093839 3.427738

H 0.181559 0.701430 -1.440874

H 0.400970 3.995436 -1.784709

H -1.651701 -0.851183 -1.985417

H 2.698898 3.082946 -2.332566

H 4.775800 1.986851 -3.167051

H 5.913428 -0.081629 -3.976794

H 4.673822 -2.268133 -3.842517

H 2.365209 -2.257372 -2.897982

C 2.855720 4.449712 0.068054

C 2.322940 3.192562 0.375165

C 1.112204 3.128672 1.070851

C 0.448808 4.294548 1.449291

C 0.986704 5.539949 1.138277

C 2.198443 5.614176 0.447550

C 2.979908 1.946605 -0.100074

N 2.601540 0.788981 0.446802

P 3.644668 -0.471899 0.511920

C 3.971872 -0.701478 2.304035

C 5.249231 -1.094215 2.705815

C 2.980614 -0.499630 3.268285

C 5.533828 -1.289720 4.056196

C 3.262553 -0.697547 4.616509

C 4.539864 -1.094786 5.011831

C 2.659218 -1.948162 0.091196

C 3.331595 -3.129793 -0.229059

C 1.267124 -1.900578 0.007730

C 2.616937 -4.255885 -0.632245

C 0.553677 -3.019859 -0.412813

C 1.226830 -4.198227 -0.731325

O 4.954416 -0.449163 -0.223544

H 3.795183 4.505814 -0.479218

H 0.710930 2.145247 1.299872

H -0.499834 4.226347 1.974800

H 0.469315 6.449329 1.431121

H 2.629932 6.582444 0.208198

H 4.025063 2.083484 -0.414450

H 6.011329 -1.226198 1.942290

H 1.993976 -0.172523 2.950348

H 6.531657 -1.591394 4.364104

H 2.488326 -0.538246 5.362148

H 4.759673 -1.247162 6.065283

H 4.418488 -3.143785 -0.196311

H 0.753438 -0.970482 0.226424

H 3.143749 -5.171644 -0.888110

H -0.526736 -2.973987 -0.513355

H 0.660931 -5.064511 -1.064658

**M-ent-3b-SR-TS4**

Zero-point correction= 0.689081 (Hartree/Particle)

Thermal correction to Energy= 0.732965

Thermal correction to Enthalpy= 0.733909

Thermal correction to Gibbs Free Energy= 0.609753

E(solv) = -5075.89319437 A.U.

C -3.134870 -5.025110 3.163167

C -4.130500 -4.109779 2.750561

C -3.834494 -3.109545 1.856358

C -2.526742 -2.969652 1.320457

C -1.522030 -3.886609 1.746244

C -1.859657 -4.908390 2.670126

C -2.187715 -1.955877 0.373531

C -0.901836 -1.871152 -0.125417

C 0.136276 -2.743315 0.357416

C -0.205683 -3.733431 1.251060

C -3.199025 -0.966723 -0.087348

C -4.241109 -1.345018 -0.985653

C -5.235538 -0.401077 -1.367192

C -5.159491 0.915930 -0.852074

C -4.140915 1.260545 -0.011671

C -3.129568 0.335595 0.386900

C -4.331281 -2.662730 -1.515152

C -5.348477 -3.013850 -2.365317

C -6.336653 -2.071151 -2.739097

C -6.275918 -0.792740 -2.249134

O -0.646302 -0.973274 -1.082392

C 1.526818 -2.589690 -0.051345

O -2.207369 0.784832 1.245538

Br -4.042957 3.035841 0.644029

N 1.934172 -1.583435 -0.755841

C 3.231373 -1.262434 -1.067415

C 4.424401 -2.062963 -0.844112

C 4.648556 -2.893558 0.278451

C 5.880374 -3.509658 0.443492

C 6.889783 -3.286423 -0.486525

C 6.597286 -2.420799 -1.541257

N 5.423733 -1.836409 -1.734002

H -3.380418 -5.811716 3.870714

H -5.138300 -4.196558 3.146731

H -4.603177 -2.407472 1.548281

H -1.079225 -5.598433 2.983565

H 0.569283 -4.408678 1.611281

H -5.907091 1.651961 -1.131589

H -3.571766 -3.385805 -1.235880

H -5.395084 -4.024515 -2.760328

H -7.135711 -2.362655 -3.414325

H -7.023662 -0.053881 -2.528164

H 0.342438 -0.911553 -1.170083

H 2.193666 -3.387731 0.281271

H -1.294124 0.387703 1.149628

H 3.324421 -0.615822 -1.935870

H 3.889860 -3.002566 1.043141

H 6.054154 -4.145962 1.308006

H 7.869013 -3.744178 -0.395705

H 7.365211 -2.186133 -2.278882

C 5.781824 -0.028856 1.060619

C 4.898713 0.618137 0.192246

C 5.418712 1.412896 -0.834021

C 6.791842 1.536075 -1.002243

C 7.667815 0.869844 -0.145079

C 7.157929 0.089296 0.889025

C 3.432012 0.441491 0.328718

N 2.657740 1.431653 -0.079214

P 1.079876 1.566438 0.327128

C 0.935223 3.170611 1.187079

C -0.217680 3.436780 1.934644

C 1.940489 4.136093 1.098657

C -0.366022 4.666336 2.571075

C 1.793172 5.360143 1.746669

C 0.638462 5.627738 2.478863

C 0.221891 1.849518 -1.256875

C -1.075928 2.363707 -1.247230

C 0.798091 1.463255 -2.467621

C -1.807854 2.459867 -2.424526

C 0.068181 1.565531 -3.649886

C -1.235966 2.055010 -3.628983

O 0.410151 0.494163 1.162036

H 5.383478 -0.638337 1.866750

H 4.720341 1.916139 -1.496104

H 7.184043 2.146567 -1.811039

H 8.741784 0.958953 -0.283622

H 7.831371 -0.434669 1.561601

H 3.105293 -0.239458 1.127260

H -0.997740 2.682321 2.014108

H 2.836906 3.907247 0.529298

H -1.266833 4.870085 3.143261

H 2.580659 6.105892 1.681143

H 0.522826 6.584717 2.980766

H -1.523983 2.685223 -0.313039

H 1.814749 1.079043 -2.471282

H -2.824930 2.842323 -2.387019

H 0.516732 1.254789 -4.589244

H -1.805826 2.120544 -4.551758

**M-4f-RS-TS1**

Zero-point correction= 0.881640 (Hartree/Particle)

Thermal correction to Energy= 0.934378

Thermal correction to Enthalpy= 0.935323

Thermal correction to Gibbs Free Energy= 0.792499

E(solv) = -5388.82895663 A.U.

C 7.002242 0.059418 1.338836

C 6.518696 1.385161 1.317204

C 5.304975 1.661036 0.738941

C 4.513619 0.635910 0.152722

C 5.004577 -0.703808 0.184123

C 6.262106 -0.957571 0.789273

C 3.236843 0.912602 -0.425203

C 2.477850 -0.096041 -1.002950

C 2.990578 -1.451208 -1.000401

C 4.198339 -1.717781 -0.392340

C 2.732973 2.322095 -0.413314

C 3.257393 3.235429 -1.387114

C 2.792521 4.579007 -1.421340

C 1.802673 4.983366 -0.490639

C 1.306826 4.096194 0.421732

C 1.765923 2.748259 0.485187

C 4.233653 2.837414 -2.342445

C 4.722740 3.726894 -3.265344

C 4.264330 5.064927 -3.288582

C 3.318649 5.476817 -2.385950

H 7.962935 -0.159156 1.796353

H 7.104183 2.185878 1.759023

H 4.928903 2.679318 0.726264

H 6.627913 -1.977803 0.809775

H 1.441765 6.008536 -0.518287

H 4.586600 1.811441 -2.334707

H 5.465594 3.399687 -3.986793

H 4.658533 5.759361 -4.024754

H 2.949363 6.499609 -2.396209

Br 4.749235 -3.530544 -0.338486

O 2.303122 -2.439611 -1.563104

H 1.406265 -2.049302 -1.830243

H 0.547433 4.410072 1.131596

C 1.134762 0.161499 -1.518022

H 0.737087 1.177744 -1.442835

C 1.128415 1.855730 1.492428

C -0.267182 1.867789 1.625365

C 1.861912 1.017286 2.336813

C -0.896452 1.046059 2.551514

H -0.879077 2.463331 0.948332

C 1.220001 0.198100 3.264298

H 2.946458 1.007068 2.278429

C -0.172586 0.182754 3.382439

H -1.982637 1.058233 2.600715

H 1.830428 -0.443214 3.891880

C -0.928829 -0.723851 4.356657

C -1.851700 -1.673506 3.571037

H -2.479032 -1.131564 2.854450

H -1.269177 -2.408928 3.006609

H -2.508494 -2.211600 4.266851

C -1.780410 0.149385 5.294337

H -1.150245 0.850008 5.853994

H -2.525267 0.728074 4.738395

H -2.315938 -0.483760 6.012468

C 0.020393 -1.573435 5.205726

H 0.682319 -0.951960 5.819928

H -0.563083 -2.212778 5.877695

H 0.636690 -2.224013 4.575791

N 0.388270 -0.804774 -1.962595

C -0.955497 -0.624521 -2.149873

H -1.325647 0.395321 -2.279049

C -1.734193 -1.656089 -2.815274

C -1.285696 -2.985654 -2.951217

C -2.139423 -3.928981 -3.493509

H -0.292070 -3.259718 -2.611700

C -3.782641 -2.213752 -3.693047

C -3.425417 -3.544699 -3.879118

H -1.811741 -4.959420 -3.604943

H -4.778932 -1.866500 -3.968147

H -4.128659 -4.252872 -4.305346

N -2.974910 -1.285281 -3.184290

C 0.201648 -2.167965 0.719812

C -1.106979 -2.045818 0.241578

C -1.924545 -3.179512 0.204409

C -1.436916 -4.410266 0.631757

C -0.122424 -4.528270 1.084478

C 0.700214 -3.403730 1.121873

C -1.621932 -0.728592 -0.215355

N -2.943719 -0.533730 -0.150182

P -3.510889 0.993550 -0.303588

C -4.968834 0.880036 -1.392151

C -5.410027 2.034748 -2.038322

C -5.665739 -0.317641 -1.562703

C -6.547126 1.997486 -2.842237

C -6.804635 -0.353445 -2.362192

C -7.247982 0.804616 -3.000103

C -4.247009 1.358185 1.339339

C -4.196689 2.666869 1.822803

C -4.782518 0.347988 2.143585

C -4.675831 2.964775 3.097129

C -5.262937 0.644852 3.415830

C -5.210109 1.954544 3.893644

O -2.614247 2.137940 -0.696400

H 0.831649 -1.283643 0.782152

H -2.941226 -3.067650 -0.162369

H -2.080160 -5.285932 0.602383

H 0.260573 -5.494404 1.401682

H 1.737852 -3.486151 1.435796

H -0.928412 0.098621 -0.021672

H -4.839342 2.952367 -1.919861

H -5.284163 -1.217840 -1.088342

H -6.882749 2.897101 -3.351051

H -7.342115 -1.288587 -2.497046

H -8.135383 0.774693 -3.626787

H -3.747702 3.433923 1.196743

H -4.784582 -0.674009 1.773217

H -4.621867 3.982357 3.474440

H -5.664050 -0.146484 4.043358

H -5.574712 2.183635 4.891421

**M-4f-RS-TS2**

Zero-point correction= 0.881595 (Hartree/Particle)

Thermal correction to Energy= 0.934330

Thermal correction to Enthalpy= 0.935274

Thermal correction to Gibbs Free Energy= 0.793032

E(solv) = -5388.82750083 A.U.

C 7.024589 0.064286 0.662045

C 6.465622 1.238595 1.210812

C 5.159019 1.562637 0.945130

C 4.347037 0.741131 0.116460

C 4.914473 -0.450120 -0.428222

C 6.267465 -0.759049 -0.133124

C 2.979158 1.058016 -0.144459

C 2.211231 0.269202 -0.988643

C 2.783560 -0.940328 -1.539624

C 4.084285 -1.274521 -1.230100

C 2.370573 2.246568 0.533255

C 2.607404 3.547241 -0.022236

C 2.040831 4.697392 0.593785

C 1.243230 4.526164 1.754013

C 1.017952 3.277469 2.260264

C 1.573991 2.107218 1.662211

C 3.391169 3.730378 -1.195005

C 3.598346 4.981982 -1.717594

C 3.034341 6.122598 -1.099880

C 2.272584 5.979002 0.030905

H 8.058696 -0.192596 0.873294

H 7.067702 1.883088 1.844131

H 4.724582 2.461936 1.370432

H 6.692581 -1.665015 -0.549834

H 0.810373 5.402354 2.230880

H 3.823017 2.858853 -1.676556

H 4.196978 5.098047 -2.616245

H 3.202523 7.107291 -1.526057

H 1.827082 6.844959 0.515024

Br 4.733656 -2.918011 -1.911260

O 2.064248 -1.737854 -2.323776

H 1.122852 -1.365592 -2.326620

H 0.407911 3.154228 3.150525

C 0.809899 0.588126 -1.249933

H 0.402716 1.508552 -0.818945

C 1.200367 0.797301 2.267405

C -0.131375 0.591326 2.651905

C 2.106653 -0.243962 2.492516

C -0.549485 -0.616580 3.190442

H -0.892656 1.350771 2.497430

C 1.684320 -1.449508 3.051916

H 3.155571 -0.116105 2.243653

C 0.346901 -1.671150 3.396377

H -1.608952 -0.716035 3.414141

H 2.424595 -2.229225 3.198958

C -0.160623 -2.982428 4.000000

C -1.322199 -3.541802 3.159604

H -2.110783 -2.800663 2.998203

H -0.970243 -3.868879 2.176825

H -1.764260 -4.405616 3.672183

C -0.657001 -2.699725 5.428417

H 0.149587 -2.291070 6.047501

H -1.479130 -1.977254 5.423578

H -1.017807 -3.624630 5.894817

C 0.935027 -4.050164 4.061763

H 1.769052 -3.744464 4.703735

H 0.519869 -4.977994 4.470827

H 1.325094 -4.267547 3.061150

N 0.044667 -0.229222 -1.905017

C -1.317404 -0.062100 -1.906153

H -1.697014 0.944733 -1.739388

C -2.134337 -0.889383 -2.780456

C -1.666327 -2.100113 -3.331491

C -2.531973 -2.883026 -4.072636

H -0.645309 -2.418910 -3.147398

C -4.228320 -1.266099 -3.652102

C -3.852212 -2.464160 -4.247405

H -2.186131 -3.820887 -4.499403

H -5.251763 -0.902591 -3.752962

H -4.566638 -3.048247 -4.818122

N -3.411419 -0.488613 -2.941432

C 0.103658 -2.401106 0.090248

C -1.256027 -2.139188 -0.111713

C -2.110038 -3.199957 -0.426214

C -1.606864 -4.490810 -0.561804

C -0.243551 -4.736696 -0.398038

C 0.613237 -3.686134 -0.070489

C -1.783284 -0.748246 -0.026659

N -3.084950 -0.605093 0.248356

P -3.626965 0.789054 0.918225

C -3.160053 2.199527 -0.160045

C -2.072081 2.990548 0.220338

C -3.754403 2.414439 -1.406991

C -1.559431 3.957208 -0.643284

C -3.259347 3.395066 -2.261509

C -2.154303 4.158192 -1.887157

C -5.430763 0.642172 0.729010

C -6.258530 1.310244 1.632220

C -5.994690 -0.109926 -0.305238

C -7.642918 1.241021 1.496326

C -7.379105 -0.173465 -0.442603

C -8.202897 0.502494 0.455921

O -3.241962 1.130563 2.335157

H 0.765760 -1.589801 0.382276

H -3.165962 -2.984189 -0.563913

H -2.279324 -5.307632 -0.810843

H 0.149753 -5.741960 -0.522777

H 1.681489 -3.854766 0.042062

H -1.039533 -0.036968 0.361747

H -1.628524 2.844134 1.201266

H -4.578691 1.784371 -1.727135

H -0.695015 4.545890 -0.345998

H -3.721850 3.547091 -3.232643

H -1.754217 4.905951 -2.566383

H -5.800441 1.865559 2.446664

H -5.330527 -0.641477 -0.983199

H -8.286110 1.758022 2.203407

H -7.817788 -0.757474 -1.247499

H -9.283224 0.448348 0.348688

**M-4f-RS-TS3**

Zero-point correction= 0.881061 (Hartree/Particle)

Thermal correction to Energy= 0.933038

Thermal correction to Enthalpy= 0.933983

Thermal correction to Gibbs Free Energy= 0.793403

E(solv) = -5388.82676340 A.U.

C 6.988676 -0.148261 1.485346

C 6.580686 1.199058 1.386341

C 5.402135 1.512118 0.756028

C 4.572672 0.504440 0.192982

C 4.990376 -0.855944 0.297371

C 6.211959 -1.148625 0.956091

C 3.326895 0.820616 -0.432813

C 2.526041 -0.172720 -0.979048

C 2.973946 -1.549386 -0.917218

C 4.150307 -1.850239 -0.264801

C 2.918252 2.258076 -0.509755

C 3.520744 3.081056 -1.519284

C 3.163605 4.454495 -1.618880

C 2.202151 4.979072 -0.718080

C 1.627729 4.175334 0.224503

C 1.979265 2.800174 0.352378

C 4.468943 2.564423 -2.445979

C 5.031410 3.369877 -3.403585

C 4.680602 4.737384 -3.491030

C 3.765554 5.263431 -2.616706

H 7.919954 -0.396954 1.986179

H 7.195994 1.987032 1.810101

H 5.083612 2.547441 0.685579

H 6.520113 -2.185022 1.034828

H 1.926087 6.027916 -0.794435

H 4.739915 1.515206 -2.388707

H 5.750403 2.953984 -4.103071

H 5.133056 5.364136 -4.254008

H 3.478641 6.310746 -2.675497

Br 4.601005 -3.683837 -0.117690

O 2.257622 -2.523200 -1.467290

H 1.370980 -2.108470 -1.732675

H 0.887897 4.576487 0.910718

C 1.191714 0.121953 -1.504742

H 0.843581 1.158912 -1.480258

C 1.248064 2.009237 1.381066

C -0.145707 2.080816 1.404700

C 1.886370 1.218000 2.346882

C -0.883620 1.368205 2.346979

H -0.686248 2.637473 0.640010

C 1.140705 0.521172 3.290913

H 2.971425 1.154124 2.360539

C -0.262495 0.567555 3.304936

H -1.965581 1.426489 2.289875

H 1.667154 -0.088298 4.021977

C -1.062082 -0.242348 4.329616

C -0.713247 -1.734779 4.202656

H -0.989852 -2.093911 3.207014

H 0.356967 -1.921050 4.340818

H -1.262032 -2.311886 4.956766

C -2.570931 -0.105236 4.098647

H -2.919687 0.929898 4.194489

H -2.846570 -0.463351 3.099961

H -3.112525 -0.709337 4.835905

C -0.718710 0.254879 5.742648

H -0.966947 1.316273 5.852037

H -1.285524 -0.310966 6.491921

H 0.347540 0.132360 5.961823

N 0.397094 -0.833405 -1.877149

C -0.946713 -0.626361 -2.057518

H -1.285001 0.396053 -2.245903

C -1.733283 -1.656455 -2.721483

C -1.346411 -3.011615 -2.752266

C -2.204318 -3.938255 -3.317111

H -0.399217 -3.316386 -2.318651

C -3.727795 -2.155624 -3.745462

C -3.429442 -3.510545 -3.833916

H -1.926347 -4.988714 -3.349245

H -4.670604 -1.764411 -4.126999

H -4.129553 -4.204564 -4.287762

N -2.916801 -1.245302 -3.208559

C 0.279316 -1.842039 0.893917

C -1.035998 -1.878192 0.416372

C -1.746897 -3.081500 0.474671

C -1.134123 -4.231492 0.964835

C 0.195328 -4.197185 1.386939

C 0.902860 -2.997597 1.351964

C -1.651761 -0.649541 -0.152230

N -2.990724 -0.553500 -0.120749

P -3.639135 0.936060 -0.312190

C -5.009587 0.748630 -1.501472

C -5.422056 1.861959 -2.232737

C -5.676610 -0.468374 -1.658887

C -6.499280 1.763876 -3.110914

C -6.760517 -0.563172 -2.526579

C -7.173838 0.553721 -3.252790

C -4.536062 1.262127 1.258181

C -4.442573 2.533405 1.826827

C -5.279219 0.273301 1.909100

C -5.082896 2.815032 3.033100

C -5.924557 0.554754 3.109061

C -5.827250 1.827212 3.672645

O -2.773166 2.127204 -0.629253

H 0.800959 -0.887964 0.902201

H -2.774610 -3.090052 0.123028

H -1.690978 -5.164230 1.003848

H 0.677575 -5.102973 1.744028

H 1.945411 -2.964515 1.660512

H -1.045999 0.249400 0.001845

H -4.875587 2.794571 -2.118066

H -5.311830 -1.340450 -1.122254

H -6.810058 2.630461 -3.688238

H -7.275155 -1.513001 -2.647156

H -8.016712 0.477748 -3.934704

H -3.846940 3.284604 1.313775

H -5.325660 -0.725010 1.480855

H -4.998258 3.804288 3.474952

H -6.494520 -0.220632 3.613701

H -6.326056 2.043928 4.613446

**M-4f-RS-TS4**

Zero-point correction= 0.880841 (Hartree/Particle)

Thermal correction to Energy= 0.934168

Thermal correction to Enthalpy= 0.935112

Thermal correction to Gibbs Free Energy= 0.790680

E(solv) = -5388.81507730 A.U.

C -5.798984 0.646744 2.448778

C -5.738096 1.441548 1.284936

C -4.522009 1.738273 0.722092

C -3.307630 1.262295 1.285672

C -3.375074 0.463296 2.466228

C -4.645584 0.171673 3.023192

C -2.035347 1.580984 0.724200

C -0.849947 1.156463 1.315847

C -0.920727 0.297865 2.479089

C -2.153394 -0.007066 3.015768

C -2.000165 2.389894 -0.534645

C -1.704872 3.790841 -0.483872

C -1.744251 4.567449 -1.675434

C -2.098940 3.937178 -2.894979

C -2.387972 2.600724 -2.924077

C -2.332279 1.805071 -1.745947

C -1.375000 4.440895 0.736926

C -1.090762 5.782205 0.765405

C -1.120734 6.549464 -0.423430

C -1.441825 5.952703 -1.614532

H -6.760861 0.410744 2.895509

H -6.651675 1.820856 0.836146

H -4.474607 2.356359 -0.169372

H -4.688885 -0.437932 3.918614

H -2.129717 4.532578 -3.804864

H -1.344852 3.851170 1.647501

H -0.834796 6.259690 1.706441

H -0.886283 7.609449 -0.388041

H -1.468136 6.529971 -2.535841

Br -2.172675 -1.114579 4.555410

O 0.169936 -0.206167 3.050892

H 0.951342 -0.074063 2.458954

H -2.654584 2.113897 -3.858954

C 0.429712 1.598975 0.784579

H 0.405383 2.223329 -0.113362

C -2.728163 0.373082 -1.841699

C -1.888543 -0.674652 -1.443449

C -3.998790 0.059161 -2.323367

C -2.334695 -1.990350 -1.509881

H -0.881780 -0.463739 -1.081181

C -4.442403 -1.258749 -2.374717

H -4.664598 0.864628 -2.626011

C -3.624738 -2.310547 -1.958246

H -1.655744 -2.778534 -1.192150

H -5.450056 -1.451936 -2.727596

C -4.088124 -3.769239 -2.015527

C -3.724818 -4.495770 -0.710686

H -2.644095 -4.527380 -0.547625

H -4.182978 -3.998128 0.150187

H -4.087276 -5.530306 -0.747579

C -3.387456 -4.459851 -3.196817

H -3.670347 -3.984976 -4.142949

H -2.301241 -4.389821 -3.092894

H -3.665259 -5.520750 -3.243975

C -5.604034 -3.885269 -2.213639

H -5.921925 -3.484171 -3.181766

H -5.897209 -4.940612 -2.182749

H -6.149536 -3.356001 -1.424957

N 1.561297 1.348148 1.376116

C 2.720115 1.803061 0.861772

H 2.731327 2.654640 0.184600

C 3.956507 1.471215 1.531419

C 3.977290 0.767767 2.759950

C 5.191717 0.455647 3.337526

H 3.037360 0.491064 3.222353

C 6.260648 1.492830 1.478362

C 6.377376 0.815708 2.687196

H 5.221659 -0.072866 4.287523

H 7.157156 1.788371 0.932623

H 7.353223 0.584305 3.101786

N 5.105234 1.826582 0.906419

C 3.613736 2.549938 -2.301456

C 2.603177 1.608220 -2.066046

C 1.386064 1.719246 -2.746984

C 1.188507 2.755494 -3.655383

C 2.198488 3.685092 -3.891626

C 3.412033 3.580678 -3.209802

C 2.836131 0.510278 -1.105607

N 1.939107 -0.446087 -1.009649

P 2.275967 -1.717381 0.005623

C 1.295393 -3.064962 -0.719563

C 0.831292 -4.073481 0.127543

C 1.042716 -3.139288 -2.092081

C 0.146242 -5.166335 -0.398225

C 0.371962 -4.239903 -2.618431

C -0.070687 -5.256149 -1.772311

C 4.023629 -2.182791 -0.295657

C 4.938228 -1.970635 0.737193

C 4.479556 -2.645226 -1.533928

C 6.296474 -2.207528 0.532050

C 5.832740 -2.898098 -1.733079

C 6.743288 -2.674415 -0.700446

O 2.034295 -1.593847 1.480656

H 4.536085 2.477204 -1.727800

H 0.611414 0.981338 -2.555498

H 0.238738 2.838820 -4.174969

H 2.039729 4.493191 -4.600523

H 4.197732 4.311008 -3.382667

H 3.878334 0.384045 -0.791440

H 1.003816 -3.975768 1.196062

H 1.354515 -2.317485 -2.731785

H -0.222540 -5.945920 0.262469

H 0.176390 -4.297714 -3.685777

H -0.604914 -6.109300 -2.183045

H 4.571806 -1.607734 1.692605

H 3.773320 -2.809812 -2.344194

H 7.001346 -2.016961 1.336400

H 6.180600 -3.262734 -2.695581

H 7.801560 -2.861357 -0.861869

**M-4f-SR-TS1**

Zero-point correction= 0.881168 (Hartree/Particle)

Thermal correction to Energy= 0.934613

Thermal correction to Enthalpy= 0.935557

Thermal correction to Gibbs Free Energy= 0.786342

E(solv) = -5388.82017960 A.U.

C 6.733447 2.400665 -1.088207

C 6.242060 2.904035 0.135673

C 4.994752 2.535832 0.573742

C 4.176913 1.649168 -0.178726

C 4.686882 1.126983 -1.405522

C 5.975788 1.534179 -1.836201

C 2.875807 1.268280 0.267347

C 2.107957 0.362190 -0.453063

C 2.635721 -0.191511 -1.681308

C 3.873449 0.218379 -2.130027

C 2.325035 1.885253 1.514183

C 1.882972 3.249457 1.475809

C 1.328359 3.843363 2.643865

C 1.202963 3.062404 3.820506

C 1.608978 1.757902 3.831510

C 2.184844 1.152224 2.680582

C 1.959250 4.035327 0.292368

C 1.529711 5.337742 0.282326

C 0.997497 5.929893 1.451501

C 0.897362 5.194832 2.603273

H 7.718008 2.696258 -1.439100

H 6.848551 3.581432 0.729295

H 4.613501 2.922086 1.514131

H 6.354608 1.141186 -2.772754

H 0.769038 3.515316 4.708298

H 2.359296 3.586753 -0.610906

H 1.590802 5.917939 -0.633438

H 0.658283 6.961233 1.428338

H 0.475113 5.630037 3.505726

Br 4.470383 -0.487746 -3.784027

O 1.933441 -1.070028 -2.390063

H 1.062792 -1.218698 -1.899781

H 1.508943 1.157553 4.731445

C 0.771545 -0.016694 -0.007182

H 0.359102 0.490985 0.869277

C 2.571669 -0.280970 2.766000

C 3.858941 -0.720614 2.439325

C 1.638031 -1.226764 3.189201

C 4.190734 -2.063785 2.539297

H 4.602317 -0.001104 2.105906

C 1.975623 -2.575812 3.278871

H 0.624024 -0.909157 3.421655

C 3.257641 -3.023336 2.956789

H 5.200702 -2.370500 2.278170

H 1.210149 -3.277446 3.592027

C 3.668195 -4.495668 3.033596

C 4.819204 -4.651124 4.041371

H 5.691193 -4.058228 3.748647

H 4.506799 -4.322282 5.038229

H 5.129688 -5.700947 4.104741

C 4.137096 -4.966982 1.647020

H 3.337293 -4.852512 0.908893

H 4.999726 -4.391409 1.298237

H 4.427986 -6.023689 1.683892

C 2.512108 -5.395333 3.481223

H 1.668649 -5.341143 2.785343

H 2.851395 -6.436235 3.518502

H 2.153468 -5.123303 4.479833

N 0.070745 -0.918610 -0.624521

C -1.228576 -1.137467 -0.249519

H -1.530559 -0.810884 0.748766

C -1.933038 -2.314316 -0.730843

C -1.540283 -3.049146 -1.865681

C -2.356160 -4.075080 -2.309590

H -0.621998 -2.794272 -2.385585

C -3.860047 -3.554463 -0.531697

C -3.549826 -4.341733 -1.636067

H -2.073606 -4.655825 -3.184101

H -4.786612 -3.709714 0.022841

H -4.220593 -5.131286 -1.959248

N -3.079002 -2.577300 -0.076186

C -0.633355 0.714958 -3.063315

C -1.844057 0.202983 -2.583857

C -2.651145 -0.543941 -3.449181

C -2.248878 -0.781724 -4.758816

C -1.029810 -0.287445 -5.220830

C -0.220998 0.460621 -4.367685

C -2.254847 0.373251 -1.162321

N -3.559600 0.252493 -0.903700

P -4.148786 0.482203 0.603243

C -5.111503 2.044291 0.506186

C -5.388594 2.729989 1.691023

C -5.560847 2.566065 -0.709064

C -6.118060 3.915557 1.664772

C -6.291412 3.751976 -0.736330

C -6.573953 4.425263 0.450401

C -5.458683 -0.782181 0.768238

C -5.572800 -1.470928 1.974640

C -6.330557 -1.079377 -0.280990

C -6.557159 -2.444544 2.135897

C -7.318607 -2.046543 -0.118877

C -7.435043 -2.728866 1.091863

O -3.267999 0.516330 1.820312

H -0.002874 1.303590 -2.400468

H -3.585051 -0.938606 -3.058648

H -2.881347 -1.371020 -5.417900

H -0.707937 -0.489017 -6.238952

H 0.742576 0.829560 -4.707579

H -1.618366 1.072179 -0.594368

H -5.003818 2.329891 2.625567

H -5.307763 2.043858 -1.628184

H -6.326320 4.447272 2.589496

H -6.635111 4.156051 -1.684873

H -7.142158 5.351497 0.428215

H -4.864331 -1.245373 2.767102

H -6.214813 -0.562836 -1.230417

H -6.637377 -2.985135 3.075386

H -7.993340 -2.276155 -0.939301

H -8.203945 -3.486627 1.217798

**M-4f-SR-TS2**

Zero-point correction= 0.881132 (Hartree/Particle)

Thermal correction to Energy= 0.934304

Thermal correction to Enthalpy= 0.935248

Thermal correction to Gibbs Free Energy= 0.789520

E(solv) = -5388.82063245 A.U.

C -5.894739 0.598695 -2.971339

C -5.409117 -0.726241 -2.924869

C -4.248096 -1.004846 -2.250271

C -3.516326 0.014559 -1.583748

C -4.001672 1.356266 -1.649349

C -5.206059 1.612368 -2.354159

C -2.319351 -0.268938 -0.863030

C -1.613689 0.745109 -0.222913

C -2.075476 2.108701 -0.329037

C -3.240791 2.373014 -1.016613

C -1.783189 -1.666165 -0.818277

C -0.602842 -1.977421 -1.573047

C -0.009411 -3.264580 -1.453491

C -0.653426 -4.244236 -0.655526

C -1.814785 -3.951356 0.003214

C -2.383599 -2.647936 -0.047711

C 0.025847 -1.023569 -2.418557

C 1.205803 -1.314011 -3.056540

C 1.819187 -2.577901 -2.894454

C 1.213575 -3.532945 -2.119943

H -6.815481 0.819990 -3.503637

H -5.952458 -1.523148 -3.423737

H -3.868048 -2.020916 -2.214558

H -5.572253 2.631711 -2.397494

H -0.199290 -5.227746 -0.565241

H -0.437146 -0.050087 -2.545127

H 1.686799 -0.556848 -3.669365

H 2.773275 -2.783585 -3.372397

H 1.678331 -4.506741 -1.984948

Br -3.793372 4.184052 -1.106884

O -1.387888 3.105249 0.226743

H -0.549925 2.703553 0.619885

H -2.298500 -4.700087 0.624855

C -0.389494 0.450496 0.515460

H -0.080787 -0.595071 0.603833

C -3.624856 -2.393729 0.732025

C -3.725278 -1.346915 1.648757

C -4.737441 -3.224136 0.559467

C -4.906377 -1.125268 2.352497

H -2.869500 -0.699425 1.816614

C -5.913579 -2.996424 1.260590

H -4.680868 -4.043396 -0.153836

C -6.028045 -1.936948 2.168926

H -4.938280 -0.296202 3.050891

H -6.763439 -3.651904 1.085265

C -7.353935 -1.704720 2.897658

C -7.284911 -0.514159 3.859146

H -6.525061 -0.665761 4.633152

H -7.056539 0.416813 3.330136

H -8.252356 -0.388624 4.357480

C -7.723347 -2.957474 3.708972

H -7.824507 -3.837778 3.066635

H -6.953908 -3.172870 4.457755

H -8.677804 -2.806517 4.227318

C -8.455434 -1.424558 1.861546

H -8.582166 -2.266527 1.173599

H -9.414738 -1.249059 2.363052

H -8.206959 -0.540257 1.266137

N 0.372831 1.388710 0.994407

C 1.632609 1.050509 1.419816

H 1.808908 0.010566 1.702863

C 2.459739 2.021030 2.118262

C 2.212707 3.406779 2.090993

C 3.128873 4.261218 2.678482

H 1.325984 3.786420 1.592422

C 4.443417 2.348847 3.230608

C 4.279023 3.730182 3.264990

H 2.958119 5.334746 2.667189

H 5.330424 1.885865 3.663443

H 5.026141 4.365410 3.730014

N 3.566643 1.506706 2.687676

C 1.298906 2.591084 -1.604672

C 2.468786 2.192386 -0.948556

C 3.502580 3.121196 -0.792849

C 3.365201 4.418052 -1.277048

C 2.189094 4.810100 -1.914294

C 1.152405 3.892472 -2.073925

C 2.597834 0.831344 -0.356418

N 3.840563 0.378298 -0.158520

P 4.046639 -1.160677 0.357146

C 4.678824 -2.080706 -1.104326

C 4.585993 -3.474931 -1.106146

C 5.187732 -1.426806 -2.227440

C 5.006760 -4.208704 -2.211779

C 5.606223 -2.159155 -3.337684

C 5.518553 -3.550214 -3.330082

C 5.491482 -1.059805 1.467681

C 5.654370 -2.038922 2.447810

C 6.423299 -0.024858 1.363713

C 6.747433 -1.993505 3.310486

C 7.516899 0.019139 2.223510

C 7.681572 -0.966849 3.196077

O 2.935880 -1.964495 0.974244

H 0.490991 1.875146 -1.733218

H 4.399000 2.803327 -0.267325

H 4.172882 5.132708 -1.141457

H 2.077538 5.827188 -2.280144

H 0.222124 4.188667 -2.550650

H 1.786743 0.143430 -0.640670

H 4.146572 -3.968554 -0.242665

H 5.225153 -0.340448 -2.225706

H 4.926494 -5.292803 -2.209540

H 5.994609 -1.644858 -4.212829

H 5.840434 -4.120791 -4.197328

H 4.898705 -2.814556 2.541738

H 6.259778 0.751955 0.622218

H 6.866448 -2.753188 4.078333

H 8.238198 0.828179 2.142909

H 8.533863 -0.929319 3.869480

**M-4f-SR-TS3**

Zero-point correction= 0.881345 (Hartree/Particle)

Thermal correction to Energy= 0.934170

Thermal correction to Enthalpy= 0.935114

Thermal correction to Gibbs Free Energy= 0.792108

E(solv) = -5388.82334299 A.U.

C -6.038141 1.786128 -0.842702

C -5.738678 1.937395 0.527145

C -4.459488 1.720598 0.976414

C -3.413477 1.346540 0.090524

C -3.714945 1.237962 -1.301198

C -5.048765 1.447015 -1.732820

C -2.083225 1.088807 0.550058

C -1.061545 0.814097 -0.349671

C -1.348883 0.779018 -1.763315

C -2.649800 0.932569 -2.186458

C -1.816808 1.116572 2.025351

C -2.391385 0.087172 2.845808

C -2.203586 0.114000 4.256956

C -1.416302 1.146206 4.826748

C -0.824862 2.082411 4.026265

C -1.016313 2.086046 2.617000

C -3.127636 -0.994873 2.286632

C -3.676637 -1.961194 3.090600

C -3.519037 -1.909718 4.495304

C -2.790438 -0.896784 5.061775

H -7.051776 1.946839 -1.199013

H -6.518176 2.223767 1.227064

H -4.232362 1.835974 2.031269

H -5.269855 1.344055 -2.789324

H -1.275838 1.167994 5.905098

H -3.237919 -1.060699 1.208508

H -4.230834 -2.777789 2.638115

H -3.962575 -2.680836 5.118812

H -2.640760 -0.852947 6.138158

Br -2.992264 0.630241 -4.025206

O -0.395441 0.525203 -2.654982

H 0.472281 0.402028 -2.169625

H -0.196993 2.857133 4.457926

C 0.296017 0.594003 0.115316

H 0.443381 0.512656 1.191171

C -0.212048 3.035590 1.804301

C -0.744168 3.809469 0.774744

C 1.177016 3.033124 1.982909

C 0.095402 4.494869 -0.100025

H -1.819826 3.832608 0.620914

C 2.007861 3.713409 1.105143

H 1.612874 2.408303 2.761706

C 1.486114 4.432290 0.022903

H -0.356966 5.053290 -0.912651

H 3.084012 3.619593 1.229513

C 2.444060 5.034780 -1.005371

C 1.704369 5.805919 -2.102434

H 1.032383 5.151862 -2.667343

H 1.117207 6.633940 -1.688403

H 2.430512 6.226827 -2.806353

C 3.230803 3.883375 -1.659167

H 3.843297 3.343975 -0.929387

H 2.548156 3.154295 -2.109371

H 3.896013 4.271821 -2.440438

C 3.421496 5.998069 -0.313381

H 4.002585 5.490567 0.462123

H 4.127262 6.410212 -1.044418

H 2.884025 6.830397 0.154848

N 1.327105 0.627695 -0.682936

C 2.551355 0.519282 -0.115545

H 2.697187 0.920300 0.885007

C 3.734331 0.496509 -0.953603

C 3.681747 0.045135 -2.290810

C 4.843855 0.050982 -3.040020

H 2.743395 -0.336560 -2.681509

C 5.998853 0.860636 -1.119526

C 6.040119 0.475151 -2.454111

H 4.825823 -0.286992 -4.073150

H 6.911830 1.179705 -0.615665

H 6.973650 0.492239 -3.007071

N 4.893194 0.882552 -0.374674

C 4.049418 -0.953985 2.462642

C 2.715671 -1.203330 2.118246

C 1.718477 -1.031886 3.088698

C 2.051707 -0.608145 4.371328

C 3.382490 -0.361917 4.707535

C 4.379161 -0.539085 3.747581

C 2.349640 -1.582764 0.733851

N 1.107094 -1.978931 0.514428

P 0.747788 -2.628195 -0.966710

C 1.421629 -4.334293 -0.900535

C 1.688918 -4.984410 -2.107962

C 1.684279 -4.990439 0.304972

C 2.197194 -6.280070 -2.111668

C 2.193361 -6.287003 0.301382

C 2.446388 -6.933766 -0.906275

C -1.059023 -2.841326 -0.916258

C -1.828304 -2.266033 -1.928037

C -1.683792 -3.568674 0.100822

C -3.218192 -2.383597 -1.903464

C -3.065460 -3.726475 0.097528

C -3.835529 -3.121699 -0.897713

O 1.187270 -1.972720 -2.243891

H 4.819048 -1.049874 1.700930

H 0.686621 -1.232379 2.811547

H 1.266110 -0.468689 5.109364

H 3.642401 -0.031684 5.709571

H 5.417679 -0.341854 3.999350

H 3.181931 -1.860243 0.076988

H 1.511709 -4.449236 -3.037172

H 1.507526 -4.468539 1.242424

H 2.406943 -6.778783 -3.054016

H 2.400610 -6.790247 1.241792

H 2.846021 -7.944354 -0.908187

H -1.331372 -1.707881 -2.718949

H -1.085105 -4.011365 0.893184

H -3.805264 -1.881413 -2.668151

H -3.544970 -4.310938 0.878247

H -4.917421 -3.223632 -0.883805

**M-4f-SR-TS4**

Zero-point correction= 0.880826 (Hartree/Particle)

Thermal correction to Energy= 0.934053

Thermal correction to Enthalpy= 0.934997

Thermal correction to Gibbs Free Energy= 0.788837

E(solv) = -5388.81670942 A.U.

C 7.501703 0.571795 -0.022437

C 6.947836 1.544192 0.836457

C 5.585067 1.670822 0.938496

C 4.702495 0.839349 0.195832

C 5.270184 -0.164609 -0.646413

C 6.681793 -0.262076 -0.741242

C 3.279909 0.969956 0.291530

C 2.443475 0.077818 -0.369394

C 3.018815 -0.967779 -1.186532

C 4.385247 -1.036181 -1.334058

C 2.711726 2.079047 1.125400

C 2.901189 3.436202 0.690697

C 2.404105 4.510485 1.481063

C 1.694958 4.218766 2.673052

C 1.480434 2.922821 3.046795

C 1.989800 1.834043 2.286572

C 3.556758 3.756932 -0.531531

C 3.728973 5.060073 -0.923583

C 3.257748 6.123546 -0.119042

C 2.606571 5.848958 1.054598

H 6.580142 0.474390 -0.109586

H 7.597096 2.190724 1.419321

H 5.161155 2.416337 1.603144

H 7.103445 -1.019541 -1.392096

H 1.309961 5.040840 3.271363

H 3.917439 2.951365 -1.161732

H 4.228510 5.276583 -1.863151

H 3.402920 7.151051 -0.439556

H 2.221618 6.652484 1.678056

Br 5.066326 -2.373840 -2.491026

O 2.237478 -1.841439 -1.822216

H 1.310324 -1.729373 -1.448403

H 0.916481 2.694520 3.946584

C 0.999853 0.124808 -0.190180

H 0.566358 1.006074 0.287350

C 1.625617 0.459095 2.720880

C 2.570065 -0.548883 2.903222

C 0.269330 0.139017 2.865514

C 2.167040 -1.850077 3.195377

H 3.627231 -0.321212 2.790216

C -0.125220 -1.164185 3.133825

H -0.490616 0.897837 2.681993

C 0.815214 -2.189397 3.299857

H 2.932411 -2.608682 3.319947

H -1.189900 -1.385830 3.161360

C 0.331418 -3.620534 3.540558

C 1.495292 -4.600807 3.719055

H 2.125380 -4.641084 2.824448

H 2.124937 -4.329370 4.574083

H 1.101416 -5.607569 3.896755

C -0.505450 -4.072451 2.331624

H -1.398772 -3.453502 2.199208

H 0.079611 -4.001884 1.408880

H -0.829650 -5.112541 2.461211

C -0.535719 -3.665700 4.809314

H -1.405577 -3.007949 4.721950

H -0.900557 -4.685472 4.981167

H 0.040713 -3.352358 5.686927

N 0.253637 -0.908366 -0.456771

C -1.066411 -0.876707 -0.130048

H -1.394675 -0.142418 0.605830

C -1.862599 -2.079601 -0.176662

C -1.524003 -3.210531 -0.948525

C -2.394969 -4.283962 -0.974856

H -0.597341 -3.217733 -1.514801

C -3.859184 -3.043317 0.444414

C -3.596899 -4.211302 -0.264060

H -2.150152 -5.168930 -1.556980

H -4.791428 -2.923745 0.998449

H -4.309699 -5.029457 -0.267174

N -3.021934 -2.008970 0.508535

C -0.328217 -0.371594 -3.420454

C -1.603646 -0.501175 -2.856885

C -2.487354 -1.446626 -3.389252

C -2.102386 -2.243765 -4.462071

C -0.826512 -2.118174 -5.008511

C 0.062180 -1.182014 -4.480885

C -1.997908 0.285473 -1.663765

N -3.289095 0.384108 -1.382827

P -3.804090 1.213948 -0.061657

C -4.642137 2.700219 -0.734201

C -4.816350 3.800910 0.107776

C -5.103536 2.762580 -2.051010

C -5.456424 4.946707 -0.355999

C -5.744010 3.908939 -2.515319

C -5.924381 4.999796 -1.667460

C -5.190096 0.208185 0.570062

C -5.319981 0.041155 1.947616

C -6.105642 -0.400703 -0.290033

C -6.364670 -0.722120 2.464728

C -7.153360 -1.157663 0.225876

C -7.286058 -1.316795 1.604975

O -2.869300 1.620080 1.044053

H 0.365770 0.355860 -3.007184

H -3.465863 -1.547495 -2.928392

H -2.795670 -2.978664 -4.862791

H -0.522265 -2.749682 -5.838690

H 1.064598 -1.087908 -4.887534

H -1.272832 1.056820 -1.362925

H -4.424428 3.750037 1.120368

H -4.933024 1.914246 -2.708838

H -5.584118 5.802033 0.302079

H -6.096099 3.955310 -3.542311

H -6.421018 5.895223 -2.031772

H -4.577194 0.496841 2.596722

H -5.977189 -0.294788 -1.364164

H -6.458444 -0.857695 3.538911

H -7.862777 -1.631715 -0.447084

H -8.102017 -1.911107 2.007821

**M-4f-RR-TS1**

Zero-point correction= 0.881305 (Hartree/Particle)

Thermal correction to Energy= 0.934000

Thermal correction to Enthalpy= 0.934944

Thermal correction to Gibbs Free Energy= 0.793308

E(solv) = -5388.82836804 A.U.

C 5.329714 0.591164 2.982662

C 4.871433 -0.742896 2.939653

C 3.573417 -1.008764 2.584446

C 2.664326 0.034010 2.258583

C 3.131465 1.381750 2.312851

C 4.480646 1.624949 2.677181

C 1.313734 -0.233495 1.876443

C 0.425040 0.799355 1.608782

C 0.895390 2.166980 1.678185

C 2.213330 2.414166 1.995501

C 0.912045 -1.669414 1.723736

C 0.213145 -2.340379 2.778305

C -0.021770 -3.742960 2.688918

C 0.402173 -4.440616 1.528895

C 1.059602 -3.782009 0.526046

C 1.338682 -2.391097 0.618597

C -0.235055 -1.652287 3.939273

C -0.866364 -2.322499 4.956509

C -1.082461 -3.718931 4.873020

C -0.671927 -4.408966 3.761440

H 6.358732 0.803024 3.259183

H 5.544644 -1.558448 3.186583

H 3.221330 -2.035096 2.553487

H 4.829379 2.650861 2.710840

H 0.188548 -5.503565 1.445626

H -0.067946 -0.581808 4.005696

H -1.202835 -1.777943 5.833629

H -1.580743 -4.237779 5.686726

H -0.842792 -5.479653 3.677500

Br 2.770672 4.227089 1.990124

O 0.075865 3.182415 1.420576

H -0.805301 2.770992 1.133523

H 1.363941 -4.306569 -0.375546

C -0.950104 0.526340 1.195271

H -1.268881 -0.513508 1.123109

C 2.123868 -1.754648 -0.470233

C 1.615304 -0.655326 -1.157050

C 3.367284 -2.263779 -0.852996

C 2.336478 -0.065851 -2.191471

H 0.606121 -0.313685 -0.934622

C 4.083163 -1.672214 -1.887096

H 3.780890 -3.121319 -0.326622

C 3.586517 -0.557413 -2.576196

H 1.887134 0.777639 -2.707127

H 5.052366 -2.085400 -2.157199

C 4.408633 0.061784 -3.709005

C 3.711892 1.276315 -4.330264

H 2.745727 1.006503 -4.768944

H 3.542048 2.064707 -3.589650

H 4.339546 1.690434 -5.127104

C 4.625699 -0.987602 -4.812025

H 5.154484 -1.867125 -4.430806

H 3.665465 -1.320935 -5.220421

H 5.219403 -0.563545 -5.630868

C 5.772102 0.515655 -3.161855

H 6.336872 -0.322949 -2.742495

H 6.373093 0.961991 -3.963458

H 5.640767 1.261041 -2.371013

N -1.769885 1.497167 0.916224

C -2.989560 1.266127 0.357106

H -3.425921 0.272360 0.446647

C -3.948691 2.356534 0.274308

C -3.608605 3.698973 0.537956

C -4.555094 4.687204 0.333879

H -2.611172 3.946901 0.884387

C -6.067588 2.985570 -0.363053

C -5.823430 4.335670 -0.129199

H -4.305166 5.727067 0.528451

H -7.042605 2.662276 -0.727435

H -6.594794 5.078036 -0.305147

N -5.175564 2.016244 -0.174612

C -2.492530 3.344401 -2.372437

C -1.757833 2.247871 -1.911042

C -0.395892 2.418689 -1.637710

C 0.211903 3.658268 -1.798117

C -0.526534 4.742489 -2.271508

C -1.878701 4.579915 -2.562427

C -2.434173 0.948916 -1.672182

N -1.712332 -0.176631 -1.721957

P -2.580679 -1.562693 -2.018713

C -1.285397 -2.808192 -2.329523

C -0.105955 -2.434646 -2.974663

C -1.502425 -4.158686 -2.036927

C 0.860866 -3.385617 -3.292735

C -0.549723 -5.115283 -2.374441

C 0.636962 -4.728506 -2.998003

C -3.370924 -2.097279 -0.457728

C -4.747548 -1.889643 -0.344289

C -2.646790 -2.537946 0.654225

C -5.389418 -2.095701 0.874736

C -3.287093 -2.742875 1.873445

C -4.658807 -2.515452 1.983081

O -3.626212 -1.510188 -3.094227

H -3.556291 3.224830 -2.569465

H 0.171716 1.575594 -1.259580

H 1.255561 3.783045 -1.520683

H -0.052841 5.711745 -2.399675

H -2.464759 5.421418 -2.922357

H -3.492265 0.940919 -1.965114

H 0.054862 -1.386420 -3.203503

H -2.420341 -4.458373 -1.536901

H 1.793362 -3.070015 -3.754631

H -0.727794 -6.162244 -2.143430

H 1.387802 -5.473933 -3.248015

H -5.293591 -1.543193 -1.217353

H -1.572930 -2.704371 0.568167

H -6.456718 -1.913427 0.962300

H -2.715721 -3.064327 2.739334

H -5.155000 -2.661497 2.938779

**M-4f-RR-TS2**

Zero-point correction= 0.881694 (Hartree/Particle)

Thermal correction to Energy= 0.934509

Thermal correction to Enthalpy= 0.935454

Thermal correction to Gibbs Free Energy= 0.792219

E(solv) = -5388.82509954 A.U.

C 5.534919 1.120968 2.503334

C 5.028050 -0.133065 2.904476

C 3.698202 -0.421616 2.728474

C 2.804012 0.518792 2.148643

C 3.318935 1.793906 1.767665

C 4.700341 2.059610 1.951084

C 1.422513 0.217569 1.927485

C 0.551832 1.174590 1.424483

C 1.062051 2.493468 1.107073

C 2.412262 2.741024 1.228346

C 0.966064 -1.177368 2.226876

C 0.218152 -1.453974 3.417284

C -0.065706 -2.803048 3.775489

C 0.373097 -3.850249 2.927065

C 1.071316 -3.567718 1.785429

C 1.380495 -2.227927 1.420190

C -0.239971 -0.418280 4.277806

C -0.940969 -0.709100 5.420475

C -1.213467 -2.051448 5.777326

C -0.781206 -3.072923 4.972017

H 6.589018 1.347612 2.636185

H 5.689143 -0.869463 3.351771

H 3.310479 -1.385480 3.042539

H 5.084038 3.027164 1.648016

H 0.137153 -4.877577 3.192993

H -0.029269 0.612280 4.011951

H -1.289541 0.097407 6.058278

H -1.766219 -2.267636 6.686807

H -0.988682 -4.109331 5.227935

Br 3.027312 4.430418 0.631031

O 0.252901 3.453564 0.673942

H -0.648983 3.011646 0.519089

H 1.388909 -4.368853 1.123290

C -0.846144 0.855060 1.126351

H -1.192641 -0.168954 1.279218

C 2.144399 -1.998856 0.165227

C 1.624369 -1.173353 -0.831579

C 3.360176 -2.646263 -0.073865

C 2.295645 -1.000295 -2.036861

H 0.645229 -0.717823 -0.692751

C 4.033131 -2.459052 -1.275300

H 3.787710 -3.285707 0.695562

C 3.515232 -1.635523 -2.283619

H 1.830622 -0.371927 -2.789524

H 4.982580 -2.966881 -1.428373

C 4.276071 -1.477095 -3.601485

C 3.548105 -0.546197 -4.576159

H 2.557944 -0.932805 -4.837976

H 3.422497 0.457310 -4.156453

H 4.129993 -0.455907 -5.500095

C 4.425557 -2.854786 -4.268640

H 4.996903 -3.544999 -3.639409

H 3.442845 -3.300957 -4.453725

H 4.949686 -2.758722 -5.227181

C 5.669371 -0.890774 -3.321684

H 6.253442 -1.538394 -2.660195

H 6.226422 -0.773717 -4.259212

H 5.585293 0.090650 -2.844022

N -1.641695 1.767646 0.658557

C -2.872154 1.476860 0.141833

H -3.355060 0.545969 0.438858

C -3.786815 2.585119 -0.114309

C -3.394616 3.936653 -0.040627

C -4.296948 4.921111 -0.404805

H -2.396938 4.195164 0.295382

C -5.872237 3.195843 -0.865663

C -5.572132 4.554754 -0.834539

H -4.007870 5.967941 -0.357431

H -6.856969 2.861291 -1.191062

H -6.309364 5.292330 -1.134226

N -5.023043 2.231467 -0.521251

C -2.076469 3.025249 -2.766993

C -1.492811 1.892082 -2.190864

C -0.105097 1.865880 -2.020638

C 0.678637 2.956844 -2.379346

C 0.087064 4.083508 -2.947589

C -1.291168 4.109492 -3.149645

C -2.350480 0.767536 -1.730785

N -1.791778 -0.446152 -1.569991

P -2.871397 -1.702299 -1.651684

C -1.843862 -3.154065 -2.039372

C -0.622134 -3.404878 -1.415852

C -2.291653 -4.014919 -3.042766

C 0.159158 -4.490274 -1.799038

C -1.520200 -5.113320 -3.416726

C -0.293150 -5.348774 -2.799737

C -3.539539 -1.971349 0.037499

C -4.905276 -1.726943 0.200904

C -2.755770 -2.254585 1.161879

C -5.479793 -1.752862 1.469859

C -3.329303 -2.278502 2.430159

C -4.691816 -2.024136 2.584046

O -4.024397 -1.613376 -2.609449

H -3.155746 3.054818 -2.903544

H 0.346462 0.990386 -1.570548

H 1.746670 2.943649 -2.174190

H 0.696118 4.940137 -3.222089

H -1.760834 4.982888 -3.594334

H -3.389122 0.840117 -2.078806

H -0.269007 -2.729302 -0.647041

H -3.238095 -3.793427 -3.528284

H 1.130430 -4.645954 -1.334621

H -1.869881 -5.779928 -4.200528

H 0.314720 -6.196664 -3.104386

H -5.493443 -1.490643 -0.681000

H -1.687264 -2.441493 1.057438

H -6.539916 -1.546686 1.588701

H -2.712959 -2.484539 3.300235

H -5.133529 -2.031761 3.577022

**M-4f-RR-TS3**

Zero-point correction= 0.880906 (Hartree/Particle)

Thermal correction to Energy= 0.934035

Thermal correction to Enthalpy= 0.934979

Thermal correction to Gibbs Free Energy= 0.790689

E(solv) = -5388.82025415 A.U.

C -5.496014 -0.808371 2.657729

C -5.828879 -0.018066 1.537120

C -4.851554 0.693541 0.888118

C -3.498158 0.655330 1.317306

C -3.168663 -0.137398 2.458540

C -4.200001 -0.864083 3.106304

C -2.469852 1.377066 0.638509

C -1.152319 1.326957 1.072253

C -0.825594 0.558949 2.251393

C -1.817916 -0.148620 2.891372

C -2.838093 2.210594 -0.549823

C -2.941000 3.633263 -0.391180

C -3.329914 4.443538 -1.494781

C -3.618947 3.820559 -2.735021

C -3.527824 2.463584 -2.863942

C -3.134337 1.633371 -1.775457

C -2.673142 4.274665 0.850269

C -2.775149 5.636629 0.978194

C -3.150442 6.438128 -0.125375

C -3.422757 5.850082 -1.332877

H -6.268425 -1.375559 3.169617

H -6.855810 0.024700 1.186493

H -5.105570 1.299645 0.024327

H -3.941882 -1.469686 3.967565

H -3.906424 4.440287 -3.581045

H -2.381995 3.667089 1.700313

H -2.563138 6.104884 1.934673

H -3.221891 7.515913 -0.011444

H -3.714380 6.451368 -2.190867

Br -1.306574 -1.157417 4.416964

O 0.412945 0.549220 2.735121

H 0.989757 1.025006 2.060426

H -3.732210 1.989662 -3.820052

C -0.092066 2.002995 0.334671

H -0.374669 2.576860 -0.553913

C -3.086317 0.165436 -2.007954

C -1.994027 -0.611438 -1.620814

C -4.152385 -0.468894 -2.658355

C -1.967367 -1.980166 -1.877245

H -1.138917 -0.177419 -1.111412

C -4.120606 -1.833337 -2.909140

H -5.023871 0.113310 -2.950338

C -3.025028 -2.619894 -2.527881

H -1.086260 -2.521100 -1.546073

H -4.970982 -2.294128 -3.406876

C -3.031666 -4.120032 -2.830776

C -1.750552 -4.805392 -2.348779

H -0.860098 -4.383108 -2.825076

H -1.628505 -4.707399 -1.264538

H -1.789803 -5.874221 -2.590498

C -3.151428 -4.334604 -4.348845

H -4.076183 -3.902248 -4.744653

H -2.309700 -3.867562 -4.870563

H -3.151707 -5.406102 -4.583242

C -4.230236 -4.779899 -2.129051

H -5.180011 -4.359983 -2.475664

H -4.242610 -5.857948 -2.331457

H -4.171496 -4.630770 -1.046136

N 1.153355 1.915501 0.689592

C 2.164559 2.434073 -0.061237

H 1.921522 3.176820 -0.824960

C 3.491664 2.522427 0.511698

C 3.911599 1.632941 1.522650

C 5.225515 1.675074 1.952691

H 3.210494 0.920538 1.942539

C 5.595751 3.434876 0.389383

C 6.101790 2.598723 1.380485

H 5.563245 0.985908 2.723388

H 6.245635 4.168661 -0.088354

H 7.140113 2.669100 1.688266

N 4.338771 3.412399 -0.047356

C 2.832562 2.206489 -3.340972

C 3.357547 1.385565 -2.339195

C 4.743131 1.225058 -2.248132

C 5.584562 1.872220 -3.145397

C 5.054127 2.681440 -4.148097

C 3.673341 2.847384 -4.243197

C 2.461755 0.702925 -1.375823

N 2.938748 -0.338522 -0.714928

P 1.933734 -1.429076 -0.009642

C 2.236115 -2.980426 -0.936399

C 1.374400 -4.056893 -0.706066

C 3.277437 -3.118830 -1.855553

C 1.555749 -5.261383 -1.379212

C 3.455496 -4.323199 -2.533897

C 2.598507 -5.394997 -2.294502

C 2.658359 -1.768838 1.624901

C 1.794240 -2.058325 2.679938

C 4.039646 -1.742404 1.840402

C 2.305453 -2.312485 3.950692

C 4.549160 -2.011147 3.106471

C 3.680803 -2.295829 4.162151

O 0.452066 -1.195557 0.094325

H 1.754119 2.337822 -3.407454

H 5.135436 0.597624 -1.452421

H 6.661040 1.750135 -3.058855

H 5.713138 3.185415 -4.849848

H 3.252518 3.478633 -5.020996

H 1.384987 0.788855 -1.584208

H 0.552989 -3.933579 -0.003008

H 3.928951 -2.268842 -2.040845

H 0.877602 -6.091393 -1.198250

H 4.261840 -4.424344 -3.255203

H 2.739239 -6.333368 -2.824094

H 0.721943 -2.036900 2.509224

H 4.699439 -1.477251 1.017835

H 1.620188 -2.500276 4.772528

H 5.622508 -1.987318 3.276105

H 4.081487 -2.490522 5.153608

**M-4f-RR-TS4**

Zero-point correction= 0.881654 (Hartree/Particle)

Thermal correction to Energy= 0.934376

Thermal correction to Enthalpy= 0.935320

Thermal correction to Gibbs Free Energy= 0.793542

E(solv) = -5388.82182048 A.U.

C -6.700651 1.167075 -0.248256

C -5.968665 2.105333 -1.006072

C -4.600281 2.149181 -0.902967

C -3.894597 1.270461 -0.037747

C -4.638905 0.312163 0.714313

C -6.050843 0.291303 0.586033

C -2.470119 1.304650 0.070166

C -1.799806 0.480809 0.960782

C -2.548447 -0.521442 1.692656

C -3.916638 -0.585126 1.543146

C -1.704060 2.232979 -0.819587

C -1.579564 3.608128 -0.431886

C -0.908197 4.529729 -1.282723

C -0.351233 4.052056 -2.496164

C -0.442489 2.730563 -2.827852

C -1.127573 1.787698 -2.003168

C -2.105211 4.089477 0.799299

C -1.974485 5.408281 1.156603

C -1.316538 6.323308 0.301164

C -0.795396 5.888747 -0.891182

H -7.783535 1.132023 -0.327843

H -6.485585 2.791204 -1.670678

H -4.033508 2.866045 -1.489143

H -6.610975 -0.436016 1.162646

H 0.174002 4.747356 -3.146197

H -2.609469 3.390761 1.459614

H -2.377818 5.753308 2.104056

H -1.221537 7.365080 0.593932

H -0.277411 6.576699 -1.555521

Br -4.827220 -1.946010 2.498789

O -1.927552 -1.379506 2.496387

H -0.941100 -1.153511 2.440889

H 0.015137 2.374162 -3.744106

C -0.365490 0.625474 1.187302

H 0.160443 1.436646 0.679852

C -1.150868 0.371784 -2.475821

C -2.268789 -0.463357 -2.380996

C 0.003267 -0.151482 -3.076762

C -2.224206 -1.778977 -2.840819

H -3.197924 -0.090848 -1.965434

C 0.034380 -1.460554 -3.534948

H 0.905176 0.453017 -3.128606

C -1.070193 -2.312207 -3.420144

H -3.118832 -2.384911 -2.737205

H 0.960741 -1.827257 -3.970124

C -0.983837 -3.747316 -3.944560

C -2.237749 -4.560694 -3.609070

H -2.397981 -4.612417 -2.526574

H -3.134225 -4.133931 -4.071534

H -2.123478 -5.584194 -3.983182

C 0.228231 -4.464705 -3.326817

H 1.165496 -3.954372 -3.567118

H 0.141962 -4.508047 -2.236641

H 0.293374 -5.488446 -3.715992

C -0.823207 -3.706782 -5.473805

H 0.078916 -3.157511 -5.761457

H -0.746982 -4.723767 -5.878240

H -1.681828 -3.211780 -5.940548

N 0.257088 -0.162167 2.014768

C 1.600365 -0.089611 2.188915

H 2.117210 0.818081 1.893490

C 2.236914 -0.872336 3.224318

C 1.549241 -1.845722 3.982121

C 2.253689 -2.621949 4.883414

H 0.481069 -1.976739 3.849076

C 4.222264 -1.445910 4.241723

C 3.629411 -2.430199 5.026595

H 1.736267 -3.377117 5.469804

H 5.293689 -1.259927 4.320317

H 4.221898 -3.020201 5.717772

N 3.570464 -0.684724 3.366178

C 1.854099 -3.360688 1.258053

C 1.428948 -2.278333 0.480865

C 0.160132 -2.316423 -0.114462

C -0.681838 -3.399555 0.109237

C -0.253848 -4.476245 0.887328

C 1.020511 -4.459029 1.450734

C 2.277103 -1.074367 0.339122

N 2.022523 -0.252518 -0.666253

P 3.033199 0.932519 -1.199494

C 3.127084 2.230685 0.077248

C 3.962519 2.191154 1.197418

C 2.148055 3.225675 -0.017330

C 3.813220 3.137278 2.210108

C 1.984272 4.156286 1.003991

C 2.822203 4.112252 2.117752

C 4.719727 0.219972 -1.308328

C 5.847749 1.045632 -1.273656

C 4.887685 -1.147283 -1.545328

C 7.121002 0.513331 -1.454368

C 6.160710 -1.681014 -1.729735

C 7.278560 -0.852428 -1.680761

O 2.607655 1.540871 -2.503608

H 2.836058 -3.326422 1.726971

H -0.154840 -1.478294 -0.728968

H -1.680358 -3.392819 -0.321881

H -0.914842 -5.321167 1.059580

H 1.359893 -5.294033 2.057896

H 3.275110 -1.150483 0.795571

H 4.695194 1.395635 1.310175

H 1.509513 3.247023 -0.896956

H 4.453210 3.091204 3.086331

H 1.200243 4.906187 0.929156

H 2.695348 4.833214 2.920712

H 5.724216 2.111919 -1.099018

H 4.013240 -1.791717 -1.585748

H 7.990792 1.163385 -1.420556

H 6.279789 -2.745713 -1.909330

H 8.271865 -1.270180 -1.820102

**M-4f-SS-TS1**

Zero-point correction= 0.880300 (Hartree/Particle)

Thermal correction to Energy= 0.933356

Thermal correction to Enthalpy= 0.934300

Thermal correction to Gibbs Free Energy= 0.790104

E(solv) = -5388.82517120 A.U.

C -4.566855 -0.271665 -3.990193

C -4.108029 -1.547716 -3.600056

C -3.152056 -1.659945 -2.621742

C -2.606952 -0.515103 -1.981275

C -3.078755 0.773495 -2.376343

C -4.063690 0.857644 -3.394258

C -1.586784 -0.616481 -0.985248

C -1.054179 0.514111 -0.381868

C -1.594000 1.818581 -0.718401

C -2.537630 1.907805 -1.722319

C -1.087695 -1.986065 -0.637993

C 0.129506 -2.460670 -1.225182

C 0.573718 -3.788662 -0.970673

C -0.236385 -4.639697 -0.174957

C -1.400509 -4.173897 0.372341

C -1.833965 -2.834149 0.164389

C 0.931299 -1.624423 -2.047226

C 2.135880 -2.062728 -2.534402

C 2.599561 -3.367250 -2.240917

C 1.824143 -4.215377 -1.491183

H -5.321735 -0.179454 -4.766112

H -4.507121 -2.439314 -4.074587

H -2.791391 -2.640896 -2.328698

H -4.413568 1.839247 -3.692643

H 0.095716 -5.658711 0.010804

H 0.582044 -0.619820 -2.259809

H 2.752513 -1.397608 -3.130028

H 3.575405 -3.681729 -2.601428

H 2.165626 -5.221509 -1.259454

Br -3.082514 3.653556 -2.219762

O -1.194776 2.919331 -0.101725

H -0.443185 2.665415 0.534288

H -2.008811 -4.818451 1.001507

C 0.083379 0.430698 0.537177

H 0.572128 -0.536218 0.686155

C -3.110385 -2.405109 0.794930

C -3.182598 -1.268504 1.601471

C -4.275568 -3.156093 0.609729

C -4.383047 -0.893057 2.198521

H -2.288126 -0.675802 1.771755

C -5.470795 -2.773841 1.203431

H -4.244279 -4.038078 -0.025890

C -5.552734 -1.633127 2.011532

H -4.389480 -0.002515 2.817305

H -6.360557 -3.372430 1.022720

C -6.894603 -1.244559 2.636585

C -6.782005 0.004155 3.516626

H -6.077036 -0.148418 4.340789

H -6.453698 0.874853 2.939989

H -7.760945 0.237430 3.949729

C -7.410690 -2.401301 3.508254

H -7.556892 -3.314026 2.922213

H -6.700444 -2.626057 4.310866

H -8.372600 -2.134532 3.962169

C -7.910387 -0.958340 1.517976

H -8.063349 -1.835939 0.881749

H -8.879524 -0.677397 1.947782

H -7.562655 -0.138941 0.880948

N 0.521802 1.507854 1.105930

C 1.712243 1.605453 1.754975

H 2.199972 0.688108 2.091021

C 1.945323 2.789163 2.574079

C 3.164005 2.898413 3.278838

C 3.433278 4.056158 3.984278

H 3.886027 2.085889 3.221651

C 1.324937 4.891802 3.250218

C 2.492568 5.088921 3.979744

H 4.370303 4.161253 4.524990

H 0.566325 5.673626 3.213099

H 2.659984 6.016736 4.516684

N 1.040527 3.785863 2.563684

C 2.045303 4.041676 -0.502075

C 2.237222 2.681598 -0.766138

C 1.608590 2.110476 -1.875704

C 0.768956 2.870262 -2.684877

C 0.559374 4.217080 -2.399182

C 1.210714 4.801875 -1.313949

C 3.054786 1.833483 0.136875

N 3.364839 0.600681 -0.280485

P 4.539033 -0.195142 0.549138

C 3.732601 -1.379433 1.687200

C 4.173700 -1.446055 3.009140

C 2.657143 -2.169081 1.273622

C 3.537601 -2.293829 3.915041

C 2.015865 -3.008932 2.178514

C 2.458527 -3.070164 3.500682

C 5.249724 -1.282135 -0.741052

C 5.819781 -2.513911 -0.412787

C 5.272484 -0.854815 -2.071054

C 6.403069 -3.308560 -1.396945

C 5.856232 -1.646089 -3.056972

C 6.422716 -2.874915 -2.721126

O 5.565108 0.585394 1.324132

H 2.537850 4.494899 0.355038

H 1.804336 1.061614 -2.075314

H 0.259084 2.407307 -3.526292

H -0.124351 4.801580 -3.007458

H 1.051636 5.852499 -1.088297

H 3.759501 2.384268 0.772624

H 5.008511 -0.817190 3.307341

H 2.319257 -2.101506 0.242493

H 3.877824 -2.341451 4.946020

H 1.164895 -3.603064 1.856271

H 1.954582 -3.721162 4.210156

H 5.796202 -2.856742 0.619048

H 4.804279 0.094404 -2.318808

H 6.839751 -4.267544 -1.131671

H 5.866149 -1.306881 -4.089379

H 6.875910 -3.494678 -3.490342

**M-4f-SS-TS2**

Zero-point correction= 0.880902 (Hartree/Particle)

Thermal correction to Energy= 0.933885

Thermal correction to Enthalpy= 0.934829

Thermal correction to Gibbs Free Energy= 0.790833

E(solv) = -5388.82514749 A.U.

C -4.554507 -0.516845 -4.017172

C -4.091369 -1.765780 -3.551117

C -3.127859 -1.816962 -2.575534

C -2.577658 -0.633582 -2.013118

C -3.050625 0.627003 -2.488728

C -4.045284 0.647764 -3.499993

C -1.555023 -0.674734 -1.014924

C -1.018464 0.491140 -0.488723

C -1.553926 1.768077 -0.914700

C -2.499433 1.800954 -1.917085

C -1.058476 -2.020385 -0.583444

C 0.155304 -2.528146 -1.148291

C 0.583135 -3.850323 -0.841292

C -0.229697 -4.654414 -0.001199

C -1.383018 -4.151116 0.535813

C -1.807453 -2.819885 0.265179

C 0.965975 -1.734347 -2.003651

C 2.154855 -2.214237 -2.488611

C 2.595994 -3.517208 -2.156317

C 1.818257 -4.320086 -1.361119

H -5.316822 -0.474324 -4.790157

H -4.493947 -2.685301 -3.965457

H -2.765369 -2.777596 -2.223842

H -4.398163 1.608759 -3.856560

H 0.091869 -5.667932 0.227652

H 0.640012 -0.728970 -2.247868

H 2.778000 -1.582000 -3.111944

H 3.558957 -3.863365 -2.520761

H 2.145107 -5.322438 -1.094692

Br -3.038906 3.514355 -2.518749

O -1.136665 2.902968 -0.367363

H -0.364980 2.652657 0.243899

H -1.989669 -4.759459 1.201448

C 0.106052 0.462506 0.452018

H 0.602998 -0.490513 0.653367

C -3.074712 -2.349193 0.883953

C -3.136583 -1.156439 1.605885

C -4.240014 -3.116152 0.780662

C -4.326719 -0.740689 2.196439

H -2.240648 -0.552136 1.716789

C -5.424363 -2.695971 1.370749

H -4.217583 -4.042881 0.211885

C -5.496304 -1.496809 2.090588

H -4.325108 0.192853 2.748474

H -6.312646 -3.313714 1.259242

C -6.823128 -1.070728 2.723346

C -6.711793 0.274838 3.446709

H -5.981119 0.233969 4.261297

H -6.417891 1.075908 2.760659

H -7.682656 0.540509 3.879126

C -7.264882 -2.133052 3.743648

H -7.398913 -3.111162 3.271032

H -6.516895 -2.242188 4.535927

H -8.217751 -1.845959 4.204386

C -7.894609 -0.940833 1.628048

H -8.058984 -1.891612 1.111853

H -8.848575 -0.625078 2.067161

H -7.594129 -0.199388 0.880881

N 0.521848 1.562620 0.996062

C 1.699279 1.668474 1.683040

H 2.150471 0.758773 2.080444

C 1.872384 2.847466 2.530807

C 0.980710 3.938428 2.521435

C 1.270944 5.050497 3.294334

H 0.078382 3.895660 1.921580

C 3.247018 3.935144 4.018318

C 2.433145 5.063797 4.064954

H 0.594294 5.901256 3.299686

H 4.162845 3.896577 4.607339

H 2.699677 5.914662 4.683105

N 2.990683 2.856819 3.283488

C 2.161756 4.091947 -0.556244

C 2.318010 2.721278 -0.785468

C 1.703094 2.148657 -1.903961

C 0.914845 2.919258 -2.753237

C 0.739312 4.277988 -2.501402

C 1.376262 4.863271 -1.408638

C 3.078577 1.860133 0.162671

N 3.390064 0.627609 -0.269069

P 4.545808 -0.198812 0.557058

C 3.688281 -1.268466 1.767520

C 3.990495 -1.124224 3.121379

C 2.666581 -2.135956 1.371864

C 3.271232 -1.843419 4.075376

C 1.947201 -2.852697 2.323017

C 2.251460 -2.703707 3.677095

C 5.155620 -1.381130 -0.701206

C 5.654387 -2.632228 -0.331463

C 5.226576 -0.991891 -2.040900

C 6.212454 -3.482811 -1.283237

C 5.785539 -1.838513 -2.995095

C 6.280627 -3.086256 -2.617332

O 5.661079 0.537002 1.239704

H 2.650648 4.548071 0.301586

H 1.868117 1.089054 -2.076622

H 0.412716 2.455619 -3.598915

H 0.094696 4.871549 -3.143009

H 1.251676 5.924789 -1.212240

H 3.774485 2.395091 0.822152

H 4.774152 -0.426592 3.404586

H 2.431492 -2.230621 0.313729

H 3.498885 -1.722685 5.131015

H 1.142510 -3.512641 2.009707

H 1.684054 -3.254330 4.422846

H 5.598881 -2.943750 0.709078

H 4.818241 -0.023905 -2.321305

H 6.593162 -4.455595 -0.983859

H 5.833826 -1.526836 -4.035255

H 6.716030 -3.748546 -3.361080

**M-4f-SS-TS3**

Zero-point correction= 0.880100 (Hartree/Particle)

Thermal correction to Energy= 0.933472

Thermal correction to Enthalpy= 0.934416

Thermal correction to Gibbs Free Energy= 0.787105

E(solv) = -5388.81843861 A.U.

C 4.287224 -2.705148 -2.965027

C 4.445546 -1.343286 -3.296402

C 3.706536 -0.390079 -2.641615

C 2.780154 -0.738713 -1.623198

C 2.616869 -2.119822 -1.299235

C 3.392852 -3.081754 -1.994291

C 2.002447 0.244472 -0.936689

C 1.086513 -0.123321 0.036887

C 0.922450 -1.518547 0.368212

C 1.671897 -2.461203 -0.297994

C 2.147381 1.681647 -1.331330

C 1.111942 2.289782 -2.117857

C 1.260221 3.634345 -2.560054

C 2.445787 4.339980 -2.230455

C 3.419900 3.745911 -1.477590

C 3.280061 2.410654 -1.003916

C -0.080972 1.589505 -2.450175

C -1.077593 2.210373 -3.162127

C -0.930688 3.548323 -3.597673

C 0.217191 4.240959 -3.307478

H 4.869499 -3.461814 -3.483430

H 5.146107 -1.048799 -4.072314

H 3.819646 0.656434 -2.905846

H 3.261172 -4.128506 -1.745163

H 2.558597 5.366482 -2.571642

H -0.235470 0.568279 -2.111745

H -1.989688 1.660582 -3.377784

H -1.729948 4.025995 -4.157213

H 0.344888 5.270100 -3.635592

Br 1.368552 -4.279410 0.159281

O 0.074806 -1.896299 1.321721

H -0.460312 -1.083101 1.573016

H 4.313803 4.299514 -1.202667

C 0.269381 0.878601 0.711391

H 0.474381 1.932871 0.497023

C 4.381601 1.844571 -0.180329

C 4.140225 1.225679 1.047209

C 5.709102 1.950565 -0.609960

C 5.188414 0.721297 1.812229

H 3.121194 1.143121 1.413250

C 6.750544 1.445862 0.156527

H 5.920203 2.417893 -1.569109

C 6.514818 0.816396 1.384952

H 4.950055 0.247009 2.758114

H 7.767571 1.538026 -0.217465

C 7.692964 0.269402 2.194555

C 7.239418 -0.391658 3.499595

H 6.721532 0.319213 4.152152

H 6.568424 -1.235501 3.308705

H 8.112435 -0.771203 4.041810

C 8.650761 1.420574 2.543200

H 9.037323 1.907391 1.642288

H 8.139236 2.180586 3.143192

H 9.505713 1.043844 3.117376

C 8.443319 -0.781222 1.359042

H 8.828416 -0.354703 0.427650

H 9.293867 -1.178944 1.925691

H 7.780614 -1.612557 1.098617

N -0.704184 0.552338 1.501872

C -1.580175 1.483960 1.982273

H -1.287406 2.536426 1.951001

C -2.485352 1.113565 3.052055

C -2.905869 -0.221437 3.223870

C -3.872182 -0.504981 4.172247

H -2.483322 -1.005295 2.605596

C -3.923471 1.818900 4.697027

C -4.402709 0.534603 4.937952

H -4.213214 -1.529205 4.305860

H -4.311153 2.661930 5.269759

H -5.161073 0.359941 5.694372

N -2.998582 2.120411 3.788841

C -3.195556 3.936320 0.305111

C -3.687571 2.678011 0.662732

C -4.899239 2.589509 1.353243

C -5.603161 3.742506 1.681237

C -5.111670 4.994676 1.315955

C -3.904595 5.088739 0.625139

C -2.925246 1.457319 0.307852

N -3.581984 0.306362 0.223166

P -2.974569 -0.916203 -0.688019

C -3.168423 -2.426197 0.311303

C -2.202954 -3.423687 0.176940

C -4.228455 -2.599412 1.206985

C -2.290800 -4.588333 0.935820

C -4.321589 -3.768830 1.955064

C -3.351638 -4.763944 1.818459

C -4.202739 -1.113815 -2.033074

C -3.784053 -1.701446 -3.229348

C -5.527139 -0.690358 -1.899934

C -4.683208 -1.876208 -4.278237

C -6.426006 -0.866508 -2.948695

C -6.006066 -1.462609 -4.136460

O -1.596501 -0.853071 -1.288766

H -2.253477 4.000956 -0.237768

H -5.260613 1.603246 1.632401

H -6.539335 3.666057 2.228032

H -5.665625 5.894151 1.570570

H -3.517482 6.061241 0.333405

H -2.041175 1.634598 -0.321468

H -1.362718 -3.261934 -0.492607

H -4.951966 -1.797949 1.336644

H -1.513464 -5.341673 0.847613

H -5.142111 -3.901530 2.655517

H -3.418462 -5.670431 2.414425

H -2.743241 -1.999069 -3.327886

H -5.831648 -0.200833 -0.978636

H -4.351141 -2.329264 -5.208470

H -7.454063 -0.530485 -2.843678

H -6.708944 -1.596433 -4.954470

**M-4f-SS-TS4**

Zero-point correction= 0.880450 (Hartree/Particle)

Thermal correction to Energy= 0.933654

Thermal correction to Enthalpy= 0.934599

Thermal correction to Gibbs Free Energy= 0.788707

E(solv) = -5388.81610195 A.U.

C -4.261501 2.747115 -2.960491

C -4.448692 1.386630 -3.284152

C -3.719611 0.423758 -2.632886

C -2.776213 0.761007 -1.626367

C -2.582174 2.140143 -1.310290

C -3.349717 3.112501 -2.002305

C -2.008611 -0.230911 -0.941993

C -1.078064 0.126137 0.020581

C -0.874696 1.519684 0.343261

C -1.618226 2.470096 -0.324564

C -2.174165 -1.667440 -1.332792

C -1.148648 -2.288003 -2.123487

C -1.313889 -3.630660 -2.565608

C -2.505426 -4.323284 -2.229692

C -3.468999 -3.718363 -1.472070

C -3.313303 -2.383946 -1.000165

C 0.051866 -1.602425 -2.458754

C 1.038559 -2.234387 -3.174729

C 0.873502 -3.569218 -3.612422

C -0.281390 -4.248623 -3.318273

H -4.835833 3.511898 -3.475984

H -5.163374 1.101661 -4.050728

H -3.854287 -0.621915 -2.890392

H -3.196302 4.157932 -1.760437

H -2.631415 -5.348554 -2.570160

H 0.223454 -0.585108 -2.117637

H 1.956981 -1.695007 -3.389676

H 1.664679 -4.055692 -4.175921

H -0.422790 -5.275828 -3.647057

Br -1.274162 4.286487 0.110505

O -0.012492 1.900057 1.276968

H 0.541691 1.101620 1.542971

H -4.367264 -4.262559 -1.192467

C -0.272702 -0.889162 0.692311

H -0.497140 -1.942868 0.487847

C -4.406408 -1.807110 -0.172785

C -4.155099 -1.179459 1.048352

C -5.737504 -1.913412 -0.591529

C -5.196789 -0.668272 1.817760

H -3.133274 -1.094268 1.405665

C -6.772441 -1.402346 0.179372

H -5.956840 -2.386501 -1.545972

C -6.526636 -0.765129 1.401827

H -4.950241 -0.186747 2.757885

H -7.792335 -1.495533 -0.186557

C -7.697827 -0.211412 2.216891

C -7.233571 0.456991 3.514356

H -6.710904 -0.250363 4.166949

H -6.563584 1.299227 3.313264

H -8.102116 0.840193 4.061192

C -8.654657 -1.358946 2.579876

H -9.048561 -1.850905 1.684981

H -8.139788 -2.115887 3.180928

H -9.504827 -0.977314 3.157946

C -8.452990 0.835094 1.380569

H -8.845160 0.403480 0.454483

H -9.299020 1.237218 1.950873

H -7.791238 1.664065 1.110428

N 0.704904 -0.556985 1.464197

C 1.594062 -1.470304 1.958897

H 1.291265 -2.522220 1.962910

C 2.479392 -1.039021 3.023943

C 3.168200 -1.989143 3.807036

C 4.086896 -1.554450 4.744602

H 2.977531 -3.048038 3.652788

C 3.588146 0.676996 4.069374

C 4.310598 -0.184080 4.890085

H 4.628093 -2.273617 5.354653

H 3.733739 1.755107 4.140051

H 5.020184 0.205274 5.612847

N 2.699659 0.282195 3.160579

C 3.067754 -4.015591 0.463534

C 3.646474 -2.763402 0.697859

C 4.899207 -2.700070 1.313751

C 5.554962 -3.867130 1.694764

C 4.971116 -5.110754 1.462033

C 3.723569 -5.181675 0.841241

C 2.921138 -1.522880 0.319800

N 3.621014 -0.399399 0.206125

P 3.007463 0.838579 -0.680469

C 3.194302 2.346407 0.318103

C 2.279910 3.379694 0.116008

C 4.220063 2.498133 1.253788

C 2.388190 4.561342 0.844208

C 4.337726 3.686026 1.968442

C 3.421455 4.718483 1.762881

C 4.234056 1.057070 -2.026753

C 3.786057 1.551173 -3.253843

C 5.585220 0.740497 -1.865707

C 4.679893 1.736832 -4.306162

C 6.479089 0.929382 -2.915921

C 6.027920 1.430225 -4.136433

O 1.634383 0.764791 -1.293328

H 2.095367 -4.064785 -0.024849

H 5.333073 -1.719379 1.486141

H 6.526079 -3.805655 2.179094

H 5.485385 -6.020826 1.758740

H 3.265151 -6.147869 0.648515

H 2.041842 -1.691658 -0.319623

H 1.460107 3.234380 -0.582149

H 4.898917 1.668871 1.436302

H 1.648286 5.344130 0.704427

H 5.134163 3.803362 2.699161

H 3.506815 5.640275 2.332759

H 2.727603 1.769264 -3.370229

H 5.919524 0.323872 -0.919371

H 4.323988 2.117138 -5.259889

H 7.528637 0.678596 -2.786780

H 6.727109 1.573888 -4.956008
